# Mining the Prognostic Role of DNA Methylation Heterogeneity in Lung Adenocarcinoma

**DOI:** 10.1155/2022/9389372

**Published:** 2022-05-28

**Authors:** Hongying Liao, Xiaolong Luo, Yisheng Huang, Xingping Yang, Yuzhen Zheng, Xianyu Qin, Jian Tan, Piao Shen, Renjiang Tian, Weijie Cai, Xiaoshun Shi, Xiaofang Deng

**Affiliations:** ^1^Department of Thoracic Surgery, Thoracic Cancer Center, The Sixth Affiliated Hospital of Sun Yat-sen University, Guangzhou, China; ^2^Department of Thoracic Surgery, Zhanjiang Central People's Hospital, Chikan District, Zhanjiang City, Guangdong Province, China; ^3^Department of Oncology, Maoming People's Hospital, Maoming, China; ^4^Department of Thoracic Surgery, Affiliated Cancer Hospital and Institute of Guangzhou Medical University, Guangzhou, China; ^5^Department of Thoracic Surgery, Nanfang Hospital, Southern Medical University, Guangzhou, China; ^6^Department of Internal Medicine, Affiliated Cancer Hospital & Institute of Guangzhou Medical University, Guangzhou Key Laboratory of Translational Medicine on Malignant Tumor Treatment, Guangzhou, China

## Abstract

**Purpose:**

DNA methylation heterogeneity is a type of tumor heterogeneity in the tumor microenvironment, but studies on the identification of the molecular heterogeneity of the lung adenocarcinoma genome with respect to DNA methylation sites and their roles in lung cancer progression and prognosis are scarce.

**Methods:**

Prognosis-associated DNA methylation subtypes were filtered by the Cox proportional hazards model and then established by unsupervised cluster analysis. Association analysis of these subtypes with clinical features and functional analysis of annotated genes potentially affected by methylation sites were performed. The robustness of the model was further tested by a Bayesian network classifier.

**Results:**

Over 7 thousand methylation sites were associated with lung adenocarcinoma prognosis. We identified seven molecular methylation subtypes, including 630 methylation sites. The subtypes yielded the most stable results for differentiating methylation profiles, prognosis, and gene expression patterns. The annotated genes potentially affected by these methylation sites are enriched in biological processes such as morphogenesis and cell adhesion, but their individual impact on the tumor microenvironment and prognosis is multifaceted. *Discussion*. We revealed that DNA methylation heterogeneity could be clustered and associated with the clinical features and prognosis of lung adenocarcinoma, which could lead to the development of a novel molecular tool for clinical evaluation.

## 1. Introduction

DNA methylation, which occurs when methyl groups are added to the DNA molecule by DNA methyltransferases, can modify chromatin structure, DNA stability, and DNA-protein interactions, allowing gene expression to be controlled without changing the DNA sequence [1]. Due to the demethylation of tissue-specific genes, CpG sites (5′-C-phosphate-G-3′) of imprinted genes, and DNA repeats, the overall methylation level in tumor tissues is lower than that in normal tissues, while the hypermethylation status of CpG islands on tumor suppressor genes inhibits protective gene transcription [2]. Methylation is known to be crucial to the internal and external microenvironments of cancer, but DNA methylation heterogeneity has yet to be systemically clarified.

For a long time, lung cancer epigenetic research has shown an abnormal methylation status in a variety of lung cancer patient samples, such as sputum [3], bronchoalveolar lavage [4], and cancer tissue [5]. In addition, many tumor-associated genes, including oncogenes and tumor suppressor genes, have altered methylation states in the early stages of lung cancer [6]. DNA methylation can be used to track recurrence after early-stage lung adenocarcinoma (LUAD) surgery [7]. Therefore, altered methylation states have translational potential in pulmonary oncology and can be used to discover biomarkers to assist in tumor detection and the prediction of cancer prognosis.

At the infant stage of lung cancer methylation studies, clinical epigenetic researchers focused on single or a few relatively well-defined tumor suppressor genes, and these studies were limited to observations of the methylation differences in tumor and normal tissue pairs and their association with clinicopathological parameters and cancer prognosis [8, 9]. In the next stage, biomarker studies attempted to detect the methylation state of multiple genes to find genomic regions with greater methylation frequency in lung cancer [10, 11]. With advances in methylation detection techniques in recent years, oncologic studies have transitioned from focusing on single or multiple genes to whole-genome DNA methylation research. For example, a comprehensive molecular profiling study showed that a large number of abnormal DNA methylation sites are present in LUAD [12]. However, the occurrence of cancer is not caused solely by a single gene but by interacting networks composed of multiple genes. Systematic analysis of methylation status is a promising way to identify potential biomarkers for NSCLC diagnosis [13]. Therefore, studying the methylation status of individual genes rather than systemic methyl typing of the methylation profile is not conducive to understanding a thorough function of DNA methylation heterogeneity in the cancer microenvironment.

We used the high-throughput methylation profile and gene expression data of LUAD patients to uncover survival-associated DNA methylation sites, as well as the effect of variation in DNA methylation on LUAD gene expression, putative biological function, and prognosis. This study could improve LUAD postoperative survival assessments based on DNA methylation heterogeneity in the LUAD microenvironment.

## 2. Material and Methods

### 2.1. Accession of Clinical, RNA Sequencing, and Methylation Data

We obtained clinical and RNA sequencing (RNA-seq) data from the Genomic Data Commons application programming interface of The Cancer Genome Atlas (TCGA) on August 31, 2018. A total of 594 samples were sequenced, and 522 clinicopathological information and follow-up data samples were accessed. Then, using the UCSC Cancer Browser, 492 methylation data generated by Infinium HumanMethylation450 were downloaded (https://xena.ucsc.edu/).

### 2.2. Curation of Methylation Sites

Data with a follow-up period of fewer than 30 days were first removed. Then, TCGA dataset and methylation data in the UCSC dataset were matched; 438 cases were selected for further analysis. Then, the removal of CpG sites with a ratio of not assigned (NA) values greater than 70% in all samples was performed in over 450,000 methylation sites from the 450 k platform. As previously reported, the genome's cross-reactive CpG sites were screened [14].

We utilized the *k*-nearest neighbors (KNN) in R (version 3.5.1) to deal with missing data in the methylation profile, further removing the unstable genomic methylation sites and single nucleotides in the sex chromosome, resulting in 208021 methylation sites.

### 2.3. Data Grouping

The 438 clinical samples with RNA-seq and methylation data were split into two groups: 219 samples for training and 219 samples for validation. (a) The training and validation sets were assigned at random, and (b) the age, clinical stage, follow-up period, and patient death rate distributions were similar in both groups.

### 2.4. Screening of Confident Methylation Sites

Using the “coxph” function in the R “survival” package, a univariate Cox proportional hazards regression model was run on the above curated methylation site, age, stage (T, N, and M), sex, and smoking history with survival data. A *p* cutoff value of 0.05 was used, yielding 13200 methylation sites. Significant methylation sites were chosen for future multivariate Cox proportional hazards regression analysis based on the results of the univariate Cox model, resulting in a reduction in the number of methylation sites to 7336 for cluster analysis.

### 2.5. Cluster Analysis

To identify molecular subgroups, the R package “ConsensusClusterPlus” was used to perform consistent clustering on significant methylation sites filtered by univariate and multivariate Cox regression. The Euclidean distance was used to determine how similar the samples were, and *K*-means was utilized to cluster them. Eighty percent of the samples were resampled 100 times. The CDF was used to determine the ideal number of clusters.

We employed the EpiDiff analysis tool to find cluster-specific methylation sites to identify the methylated molecular types of LUAD [15]. The mean methylation level of each methylation site in the 7336 sites was calculated for each cluster, resulting in a 73367 matrix that was input into EpiDiff software at the cutoff of 4.18, which is a value calculated by entropy comparisons to minimize within-group variation while maximizing between-group variation. Cluster-specific methylation sites comprised a total of 630 methylation sites.

### 2.6. Gene Function Analysis

We used g:Profiler [16] to perform the Kyoto Encyclopedia of Genes and Genomes (KEGG), Gene Ontology (GO), and transcription factor enrichment analyses. The EnrichmentMap plugin in Cytoscape was used to visualize the correlation between the enriched GO terms, KEGG pathways, and transcription factors.

### 2.7. Model Validation

To test the discriminatory ability, the naive Bayes classifier with tenfold cross-validation was applied to the 630 methylation sites by R package e1071. The ROCR package in R visualized the positive and false-positive rates.

## 3. Results

### 3.1. Curation of LUAD Survival-Associated Methylation Sites

A total of 208021 methylation sites were found after the methylation sites were screened.

To evaluate LUAD survival-related methylation sites, we used univariate Cox regression analysis on each methylation site. We defined *p* < 0.05 as the cutoff value, and a total of 13200 methylation sites associated with LUAD survival are shown in Table S1. In the univariate Cox regression analysis, TNM staging, N staging (lymph node metastasis), and T status (tumor size) were significant prognostic indicators with log-rank *p* values of 2.936 × 10^−6^, 7.153 × 10^−7^, and 0.0413, respectively. Following the univariate Cox model, the significant methylation sites were selected and subjected to the multivariate Cox regression model with T status, N status, TNM staging, and age as covariates. For further LUAD prognostic modeling, a total of 7336 significant methylation sites were obtained (Table S1). The top 10 methylation sites associated with LUAD survival are listed in Table 1.

### 3.2. Prognosis-Associated Methylation Profile and Identification of DNA Methylation Subtypes

**Figure 1:**
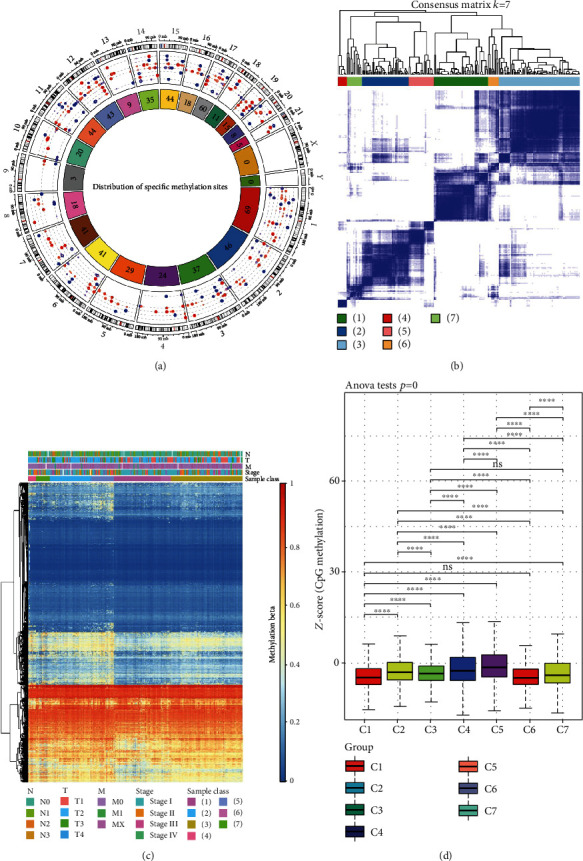
Landscape of LUAD prognosis-associated methylation sites. (a) The distribution of differentially methylated sites on chromosomes. (b) The 7336 methylation sites in 7 methylation clusters based on consensus clustering (*k* = 7). (c) Heatmap of the DNA methylation level incorporating DNA methylation subtypes, clinicopathological stage, and TNM stage. (d) The average number of methylation sites per cluster.

We first observed the distribution of differential methylation sites, which were equally distributed in the human genome (Figure 1(a)). We next hypothesized that the methylation sites might function in groups rather than working individually, so the resulting methylation locations were subjected to cluster analysis, aimed at mining potential molecular subtypes. Based on the cumulative distribution function (CFD) curve, we observed that the clustering was stable at 6 or 7 clusters (Figure S1A). Then, we selected seven subtypes based on the CFD delta area that had the most stable clustering results (Figure S1B). The 219 tumor samples were then assigned to seven subgroups based on the consensus matrix: 49 to subgroup 1, 42 to subgroup 2, 73 to subgroup 3, 8 to subgroup 4, 23 to subgroup 5, 10 to subgroup 6, and 14 to subgroup 7 (Figure 1(b)). Next, visualization by heatmap incorporating TNM staging, N category, M category, and T category of the 7336 filtered methylation sites showed that most of the methylation sites were of low abundance (Figure 1(c)). In addition, the level of differential methylation measured by the Z score per cluster revealed that cluster 1 had a lower methylation level and that the methylation abundances were significantly different among the seven clusters (Figure 1(d)). These results confirm that DNA methylation subgroups associated with LUAD prognostic value exist.

### 3.3. Functional Analysis of Cluster-Specific Methylation Sites

**Figure 2:**
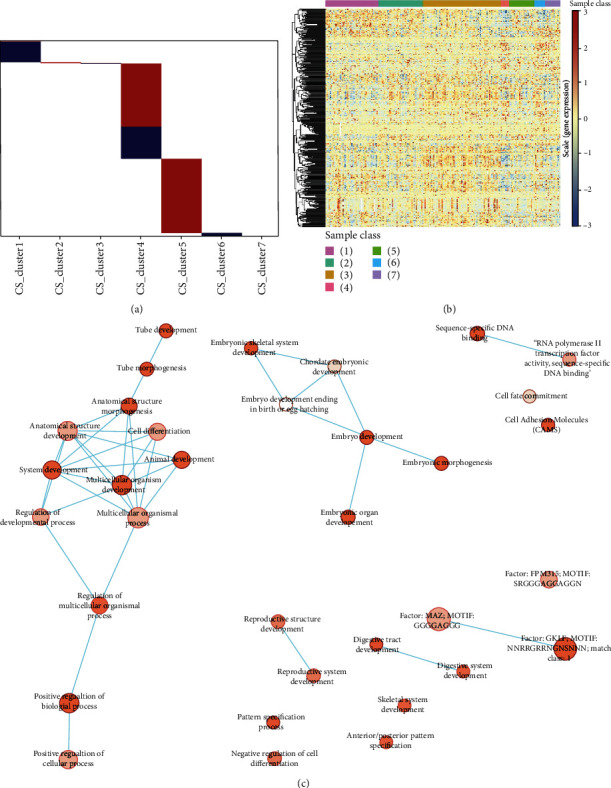
Identification of LUAD-specific methylation sites and functional analysis: (a) methylation-specific site distribution; (b) expression profiling of annotated methylation site-regulated genes; (c) functional analysis using KEGG, GO, and transcription factor enrichment analyses.

Next, we hypothesized that certain cluster-specific methylation sites could systematically play crucial roles in gene expression, thereby affecting biological function. We found 630 cluster-specific methylation sites, with more particular methylation sites in clusters 4 and 5, the majority of which were hypermethylated (Figure 2(a)). The other subclusters only have a few distinct methylation sites, most of which are hypomethylated.

We identified a total of 459 genes close to the 630 methylation sites to observe how individual methylation sites in the subgroups affect the related gene expression levels. Table S2 lists the gene annotations and related methylation subgroups of cluster-specific methylation sites. In addition, we used the training set to extract RNA-seq expression data for 359 genes corresponding to 218 samples. The heatmap expression profile (Figure 2(b)) shows that these subgroups have cluster-specific expression patterns, implying that the DNA methylation levels of these genes are linked to altered mRNA expression in LUAD.

Functional analysis showed that these genes with methylation sites were enriched in multiple Gene Ontology (GO) terms and transcription factors (Table S3) and were mainly enriched in biological processes involved in cell activity and embryo development (Figure 2(c)). Notably, these genes are enriched in the cell adhesion molecule pathway, implying that they are linked to tumor metastatic transformation in general. To further explore the specific signaling pathways enriched in annotated genes from each subgroup (Figure S2), we observed that signaling pathways were affected in a methylation subgroup-specific manner. Based on this observation, we performed biological process enrichment analysis using genes annotated with specific methylation sites in the seven subgroups, of which only C1, C3, C5, and C7 had significantly enriched pathways (Figure S3A-D). This finding suggests that systematic alteration of methylation sites affects different biological functions in LUAD subgroups, participating in the biological mechanism of different survival outcomes of LUAD.

### 3.4. Translational Implication of Methylation Subtypes on LUAD Survival Assessment

**Figure 3:**
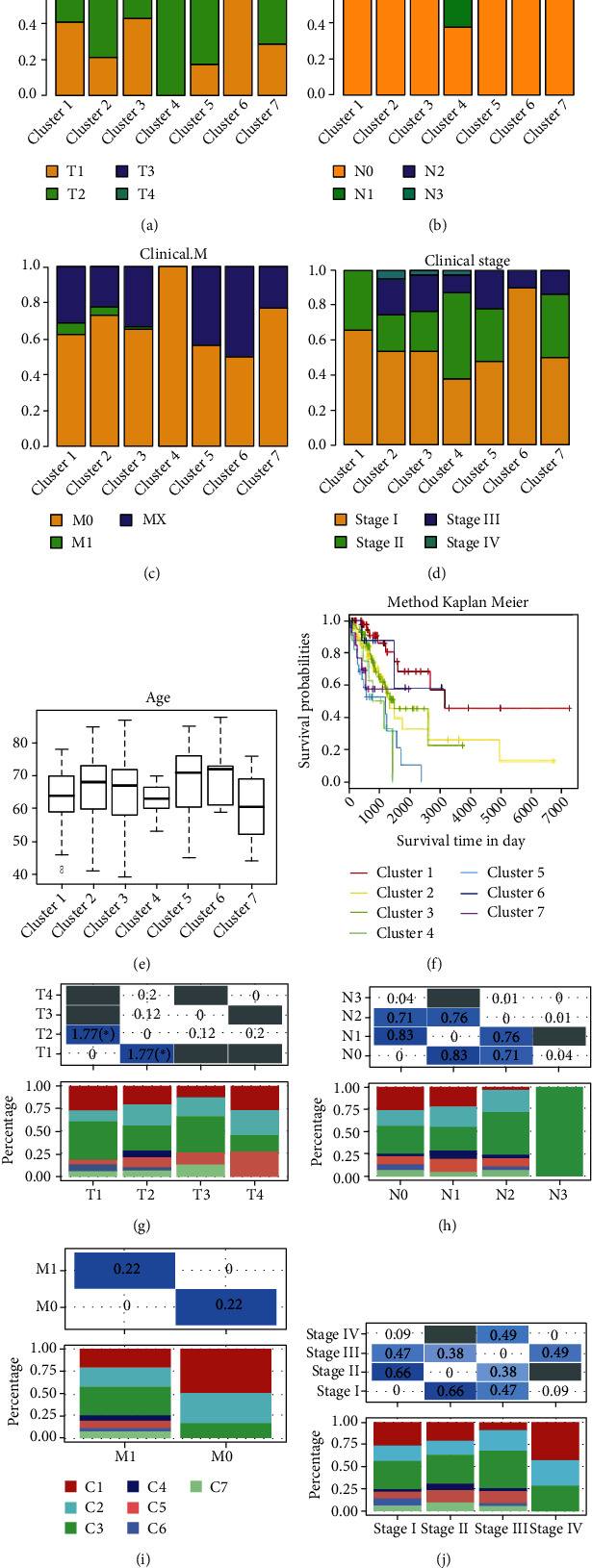
Clinical and prognostic features of the seven methylation clusters.The distributions of the seven subgroups in the T stage (a), N stage (b), M stage (c), and TNM stage (d) in the training set. (e) The age distribution in the seven methylation subtypes. (f) Prognostic differences among the seven methylation subtypes. The comparisons of the seven subgroups in T stage (g), N stage (h), M stage (i), and TNM stage (j) were visualized. The gray area represents NA, and the values in the table are −log10 (*p* value). The ANOVA test was used.

We evaluated the distribution of each of the seven molecular subtypes according to T status, N status, M status, TNM staging, and prognosis to determine the clinical importance of the methylation subtypes. The distributions of TNM staging, N stage, M stage, and T stage of the seven subgroups were plotted and compared between clusters. As shown in Figure 3(a), methylation clusters 2 and 5 were distributed in larger LUADs. Lymph node metastasis more easily occurred in cluster 4 (Figure 3(b)), and a higher proportion of distant metastasis was found in clusters 5 and 6 (Figure 3(c)).  Figure 3(d) shows that patients in cluster 2 and cluster 3 are associated with advanced TNM stages. Then, age variances (Figure 3(e)) and sex differences in cluster 3 and cluster 6 (Figure S4A) among the seven methylation subtypes were analyzed. However, the association between methylation clusters and treatment response (Figure S4B), as well as other common parameters such as BMI and comorbidities, is not clear due to missing data. Furthermore, there were prognostic disparities among the methylation subtypes, with patients in cluster 1 having the greatest prognosis and patients in clusters 4 and 5 having the worst prognosis. These findings suggest that hypomethylated LUAD samples had a better prognosis than hypermethylated LUAD samples (Figure 3(f)). Detailed clinical parameters and survival analyses of each cluster comparison in the training set are shown in Figures 3(g)–3(j) and Figure S5.

### 3.5. Validation of the Prognostic-Associated Methylation Subtype

**Figure 4:**
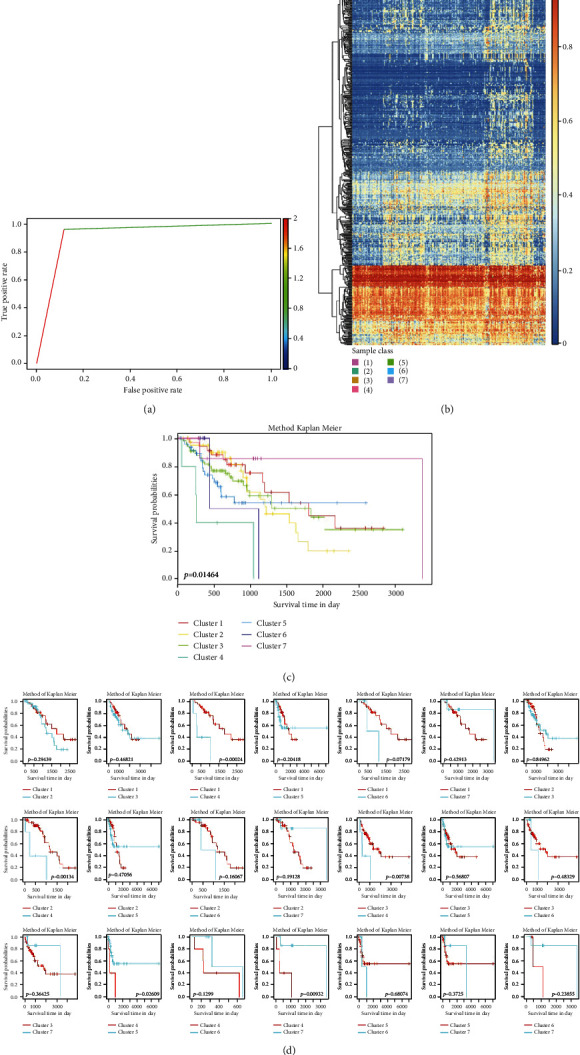
The LUAD DNA methylation subtype-specific prognostic model has been validated: (a) the validation set's area under the curve; (b) the validation set's methylation profile of subtype-specific methylation sites; (c) the validation set's prognosis differences; (d) a detailed survival analysis of each cluster comparison.

To identify subtype-specific methylation sites, Bayesian network classifiers were constructed by using 630 specific methylation sites identified by EpiDiff. The model established using the training set had a classification accuracy of 93.61 percent. The receiver operating characteristic curve's area under the curve was 0.9227 (Figure 4(a)). We used the validation set to assess the model's stability and reliability after selecting 630 CpG methylation sites from the test set. The methylation profile of the seven subtype-specific clusters also showed distinct methylation patterns, as previously shown (Figure 4(b)). The number of samples in each subgroup in the validation set was 35 in subgroup 1, 46 in subgroup 2, 69 in subgroup 3, 5 in subgroup 4, 51 in subgroup 5, 5 in subgroup 6, and 8 in subgroup 7. The clinical stage and age distributions in the validation set were then found to be consistent with those in the training set (Figure S6A-D). Figure S6E-H depicts the distributions and comparisons of the seven subgroups in the T, N, M, and TNM stages. As shown in Figure 4(c), significant prognostic differences were classified by the subtype-specific cluster model with a *p* value of 0.015. Patients in cluster 1 have a better prognosis than patients in other subtypes, which is consistent with the training set results. Detailed survival analysis of each cluster comparison in the validation set is shown in Figure 4(d). These results confirmed that the methylation clusters are distributed differently in clinically defined LUAD subgroups and affect their prognosis.

## 4. Discussion

Whether it is improper hypermethylation or hypomethylation, abnormal DNA methylation is linked to the occurrence and progression of cancer. Understanding the DNA methylation changes in cancer tissues represents a promising tactic for enhancing cancer postoperative recurrence control and treatment. The presenting integrative analysis, although with potential limitations such as batch effects in the generation of RNA-seq together with DNA methylation data, not at the single-cell level, and lack of multiple testing, shows that methylation site subtypes exist in LUAD, and most of the methylation sites were at low abundance. Furthermore, we discovered that the expression profiles of the seven methylation subtypes were different. Taking methylation cluster 1 as an example, the methylation level is significantly lower than that in the other subtypes, suggesting that there could be a unique biological meaning in this downregulated methylation block, and the assessment of LUAD prognosis could be further subdivided based on DNA methylation subgroups.

Altered methylation status of a single gene has been found to be linked to the prognosis of patients with non-small-cell lung cancer (NSCLC). For example, *SHOX2* was discovered by Dietrich et al. to be an independent predictor of prognosis as well as a biological indicator for the early diagnosis of NSCLC [17]. A similar example is that the promoter methylation of *TMEM88* plays a prognostic predictor role in NSCLC [18]. A meta-analysis indicated that *RASSF1A* methylation status can be applied to predict NSCLC prognosis [19]. Adding to these findings, our study found that there is a systematic change in gene expression associated with abnormal methylation sites in LUAD tissue. We found that the gene expression patterns in LUAD tissues differ among methylation subtypes, suggesting that DNA methylation, in cancer tissues, can cause a systematic alteration in gene expression. As a result, research into the combined effect of methylation sites on gene expression is needed.

Previous findings showed that abnormal gene methylation could affect cancer prognosis. For example, hypomethylation of cytoplasmic polyadenylation element-binding protein 1 (*CPEB1*), in our gene list (Table S2), can be used as a potential glioma prognostic marker [20]. Another example is repulsive guidance molecule member A (*RGMA*), which is a gene in a prognostic mRNA signature for breast cancer [21] whose methylation frequency can be used in evaluating colon cancer prognosis [22]. In terms of lung cancer, the aberrant methylation status of the *APC* and *CDH13* promoters was associated with lung cancer risk [23, 24]. Therefore, by providing potential candidates for methylation sites and target genes, the prognostic roles of identified genes with altered methylation sites in this study are candidates worthy of further study.

It is worth mentioning that, as reported in a previous in vitro study, epithelial gene expression is enriched in cell adhesion functions, whereas mesenchymal genes are enriched in regulators of transcription [25]. Our study shows that the methylation-influenced genes in our model were enriched in the KEGG enrichment pathway of cell adhesion as well as in the transcription factor GKLF. *GKLF*, also named *KLF4*, in lung cancer tissues was found to regulate lung tumor-initiating cells at a considerably lower level than that in normal lung tissues [26]. The abnormal methylation of cell adhesion molecules is involved in multiple cancer development processes [27], such as tumor angiogenesis [28], and is one of the consequential steps in metastasis [29]. Therefore, obtaining an understanding of how methylation sites systematically affect cell adhesion would have great translational value in the development of broad-spectrum DNA methylation-targeted agents for both LUAD prevention and treatment.

## 5. Conclusions

This study systematically summarized the methylation sites of LUAD and, for the first time, proposed seven DNA methylation subtypes that are closely related to LUAD prognosis. Abnormal DNA methylation clusters in LUAD could affect changes in gene expression levels in a cluster manner. Different methylation subtypes are associated with clinical characteristics and prognosis, suggesting that DNA methylation may play a role in cancer formation and intrinsic malignancy, providing important bioinformatics hints for the further development of epigenetic biomarkers and therapeutic targets for LUAD.

## Figures and Tables

**Table 1:** Top 10 methylation sites associated with LUAD survival.

CpGs	Univariate Cox regression				Multivariate Cox regression			
	*p* value	HR	Lower 95% CI	Upper 95% CI	*p* value	HR	Lower 95% CI	Upper 95% CI
cg07219542	1.16*E* − 05	33.410512	6.96169503	160.343466	7.08*E* − 07	103.155098	16.5088788	644.560682
cg02337836	4.05*E* − 05	49.5013782	7.68221983	318.96854	5.42*E* − 06	148.052666	17.1811057	1275.79636
cg24237439	5.75*E* − 07	67.9228467	12.9953428	355.012805	6.10*E* − 06	118.317374	14.9524332	936.235643
cg10463708	6.44*E* − 06	25.0215951	6.17787667	101.342298	7.19*E* − 06	52.062116	9.26602794	292.516269
cg02709432	0.00035404	12.7481225	3.15438224	51.5202711	7.38*E* − 06	38.448822	7.79584763	189.628118
cg14565265	3.17*E* − 06	10.0647825	3.81051651	26.5842821	8.61*E* − 06	14.0577266	4.38807397	45.0356301
cg24073738	0.00517384	61853009.6	213.657681	1.7906*E* + 13	1.64*E* − 05	1.1925*E* + 16	581566476	2.45*E* + 23
cg06498232	0.00014643	54.6599825	6.92974791	431.1432	2.06*E* − 05	171.311278	16.0608167	1827.27657
cg02156680	0.00242903	9.69422363	2.23261927	42.0931473	2.32*E* − 05	43.7945623	7.60669604	252.141492
cg02874942	0.00025906	26.0385172	4.53017875	149.663935	2.50*E* − 05	84.0269719	10.7028099	659.689566

## Data Availability

We collected clinical and RNA sequencing (RNA-seq) data from the Genomic Data Commons application programming interface of The Cancer Genome Atlas (TCGA, August 31, 2018). The data on methylation were obtained from the UCSC Cancer Browser (https://xena.ucsc.edu/).

## Abbreviations

CpG: 5′-C-Phosphate-G-3′
CFD: Cumulative distribution function
GO: Gene Ontology
KEGG: Kyoto Encyclopedia of Genes and Genomes
LUAD: Lung adenocarcinoma
TCGA: The Cancer Genome Atlas.

## Supplementary Materials

Supplementary 1 Supplementary Figure 1: the selection of methylation clusters linked to prognosis. Curve of the cumulative distribution function (a). (b) Consensus clustering delta area curve, showing the relative change in the area under the CDF curve for each category number *k* compared to *k*–1, yielding 7 clusters.

Supplementary 2 Supplementary Figure 2: specific pathways of each subtype. Gene expression values were used to calculate the score of each sample in the KEGG pathway, and the differences between pathway scores in each subgroup were analyzed.

Supplementary 3 Supplementary Figure 3: the biological processes of the seven subgroups were compared. Biological process enrichment analysis was performed on the genes annotated with specific methylation sites in the seven subgroups, and it was discovered that cluster 1 (A), cluster 3 (B), cluster 5 (C), and cluster 7 (D) had enriched pathways.

Supplementary 4 Supplementary Figure 4: detailed analysis of sex and treatment response differences among clusters.

Supplementary 5 Supplementary Figure 5: detailed survival analysis of each cluster comparison in the training set.

Supplementary 6 Supplementary Figure 6: the clinical value of the LUAD methylation subtype-specific prognostic model in the validation dataset. The proportions of different tumor sizes (a), lymph node metastasis statuses (b), and TNM stages (c) in subtype-specific classification. The age distributions in subtype-specific classification (d). In the ANOVA test, the distributions of the seven subgroups in T stage (e), N stage (f), M stage (g), and TNM stage (h) in the validation set were pairwise compared. The gray area represents NA, and the values in the table are −log10 (*p* value).

Supplementary 7 Supplementary Table 1: the discovered methylation sites linked to LUAD survival by univariate and multivariate Cox regression.

Supplementary 8 Supplementary Table 2: methylation sites and annotated genes in each subgroup.

Supplementary 9 Supplementary Table 3: functional analysis of methylation-regulated genes.
