# Supplementary figures and images for: Mining the Prognostic Role of DNA Methylation Heterogeneity in Lung Adenocarcinoma
Source: Dis Markers. 2022 May 28;2022:9389372. doi: 10.1155/2022/9389372 (PMC9168807; doi:10.1155/2022/9389372)

## Slide 1
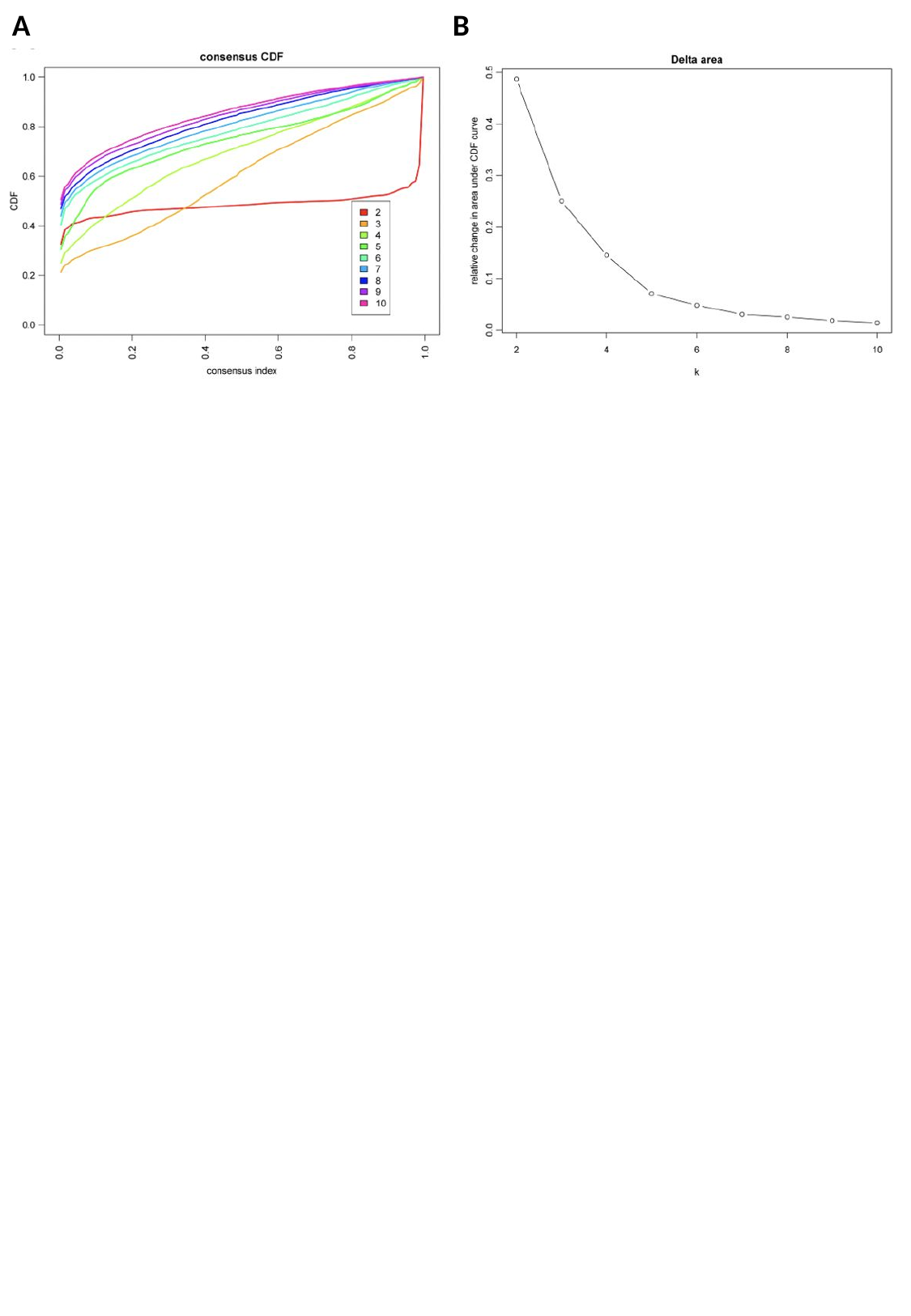

A
B

Supplement: Supplementary 1 — Supplementary Figure 1: the selection of methylation clusters linked to prognosis. Curve of the cumulative distribution function (a). (b) Consensus clustering delta area curve, showing the relative change in the area under the CDF curve for each category number k compared to k–1, yielding 7 clusters. [file 9389372.f1.pptx]

## Slide 1
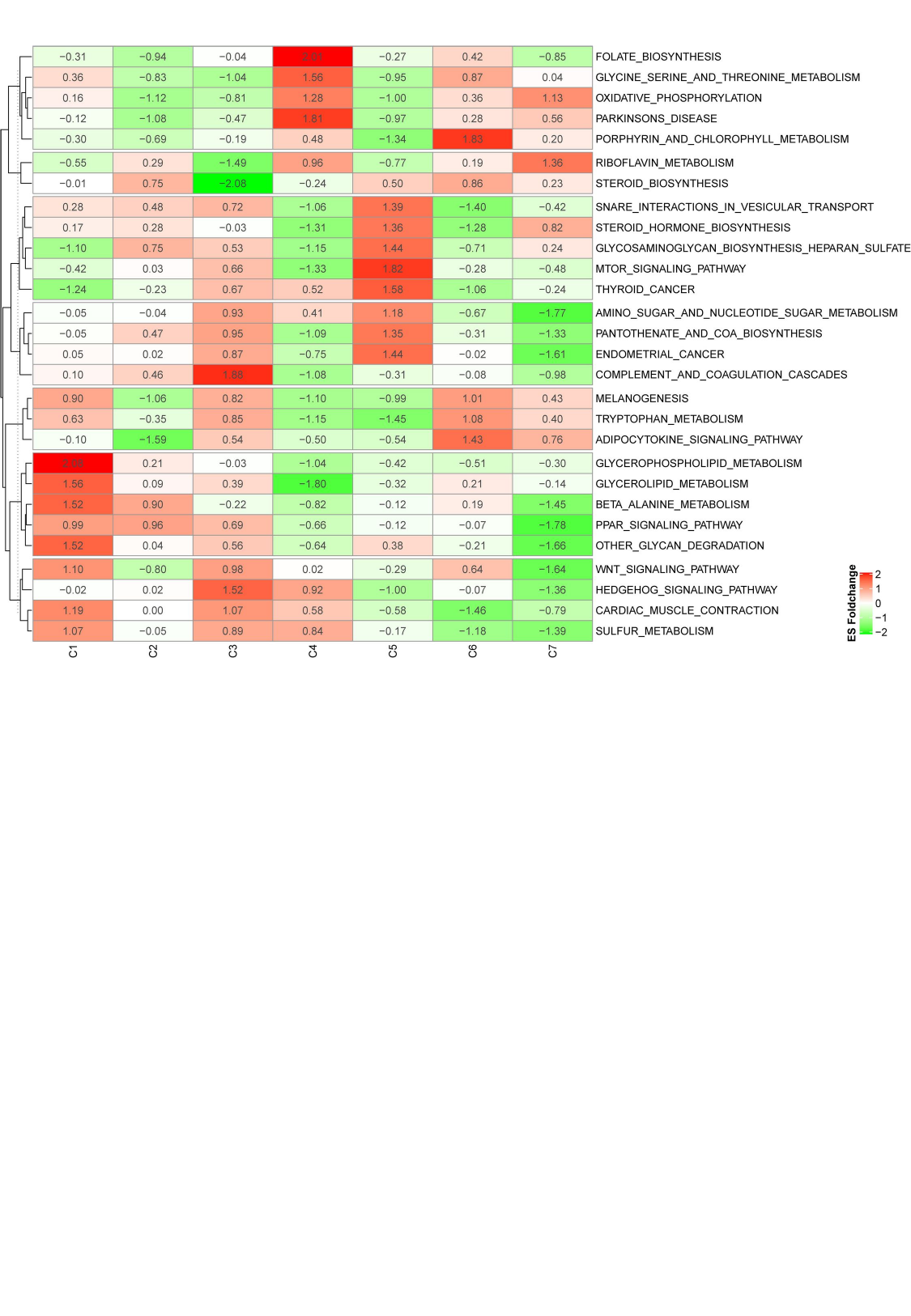

Supplement: Supplementary 2 — Supplementary Figure 2: specific pathways of each subtype. Gene expression values were used to calculate the score of each sample in the KEGG pathway, and the differences between pathway scores in each subgroup were analyzed. [file 9389372.f2.pptx]

## Slide 1
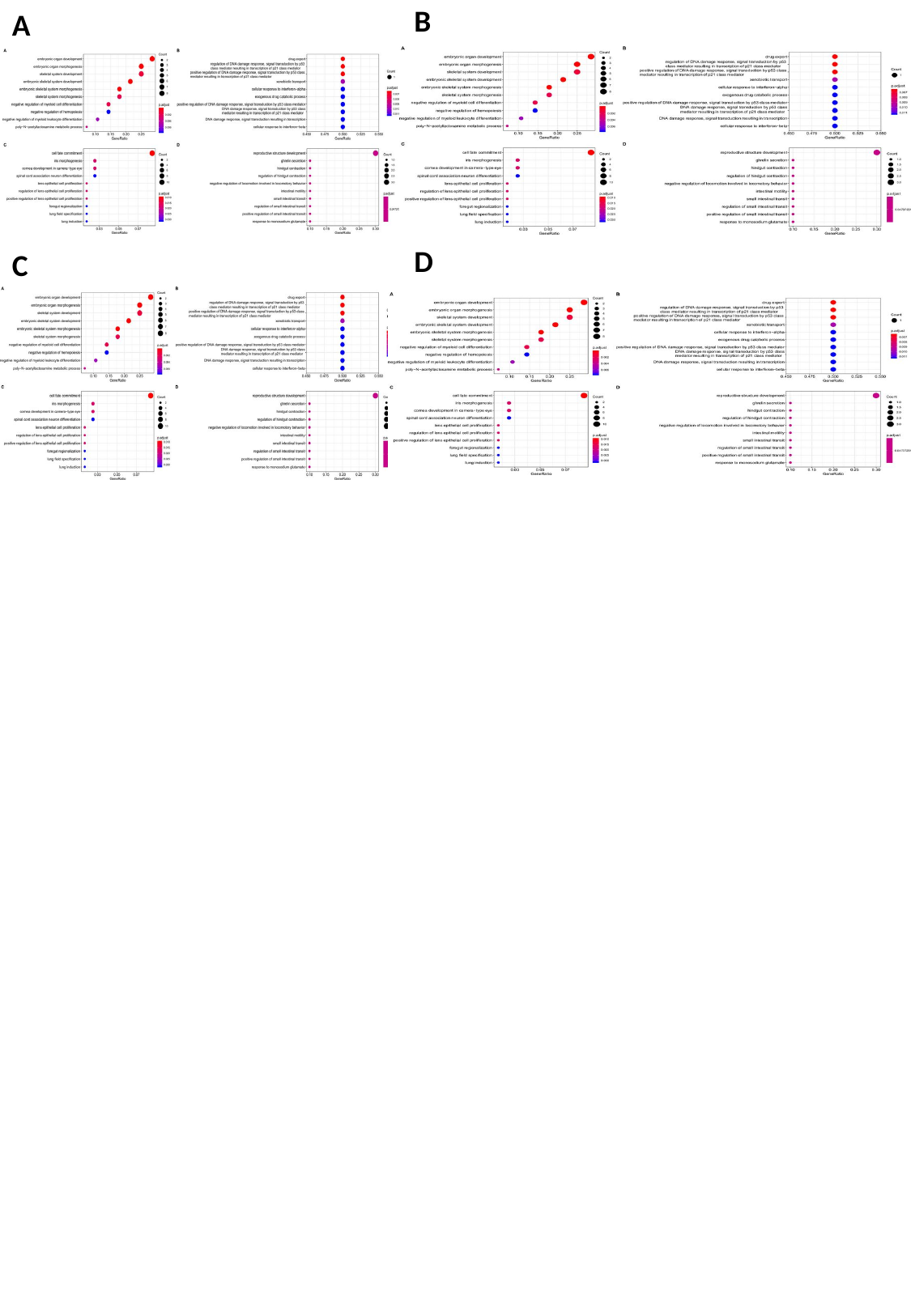

A
B
D
C

Supplement: Supplementary 3 — Supplementary Figure 3: the biological processes of the seven subgroups were compared. Biological process enrichment analysis was performed on the genes annotated with specific methylation sites in the seven subgroups, and it was discovered that cluster 1 (A), cluster 3 (B), cluster 5 (C), and cluster 7 (D) had enriched pathways. [file 9389372.f3.pptx]

## Slide 1
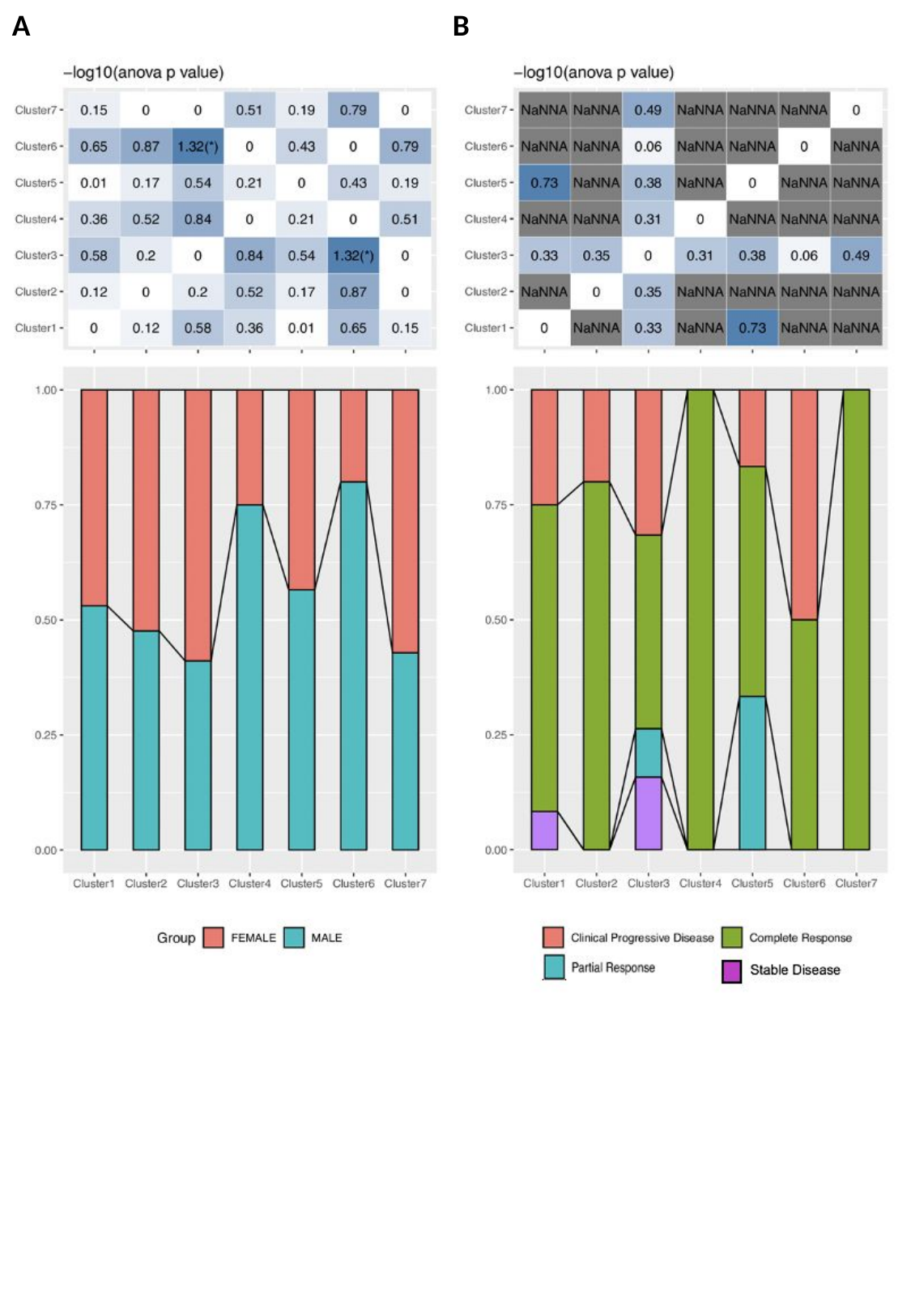

A
B

Supplement: Supplementary 4 — Supplementary Figure 4: detailed analysis of sex and treatment response differences among clusters. [file 9389372.f4.pptx]

## Slide 1
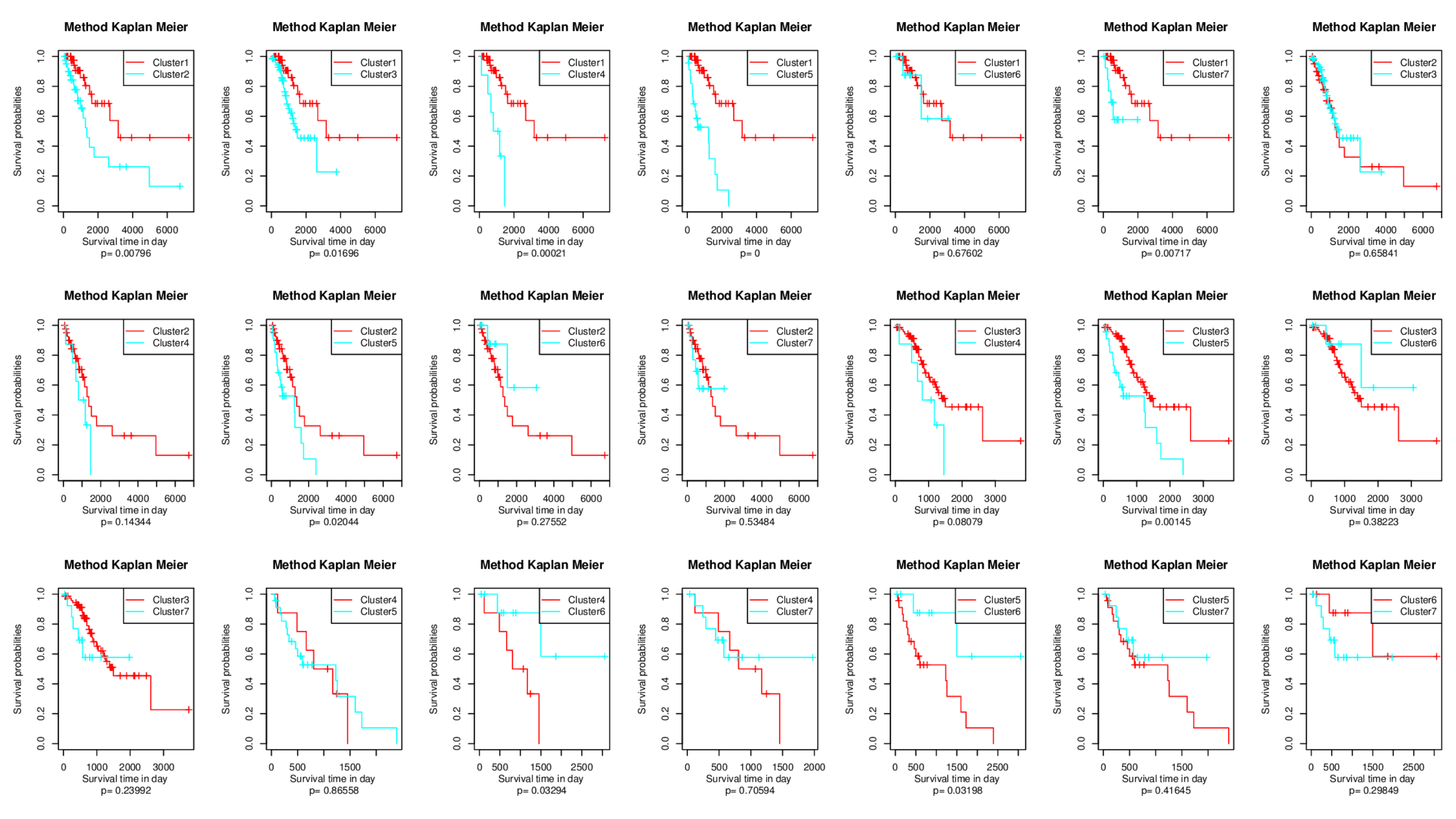

Supplement: Supplementary 5 — Supplementary Figure 5: detailed survival analysis of each cluster comparison in the training set. [file 9389372.f5.pptx]

## Slide 1
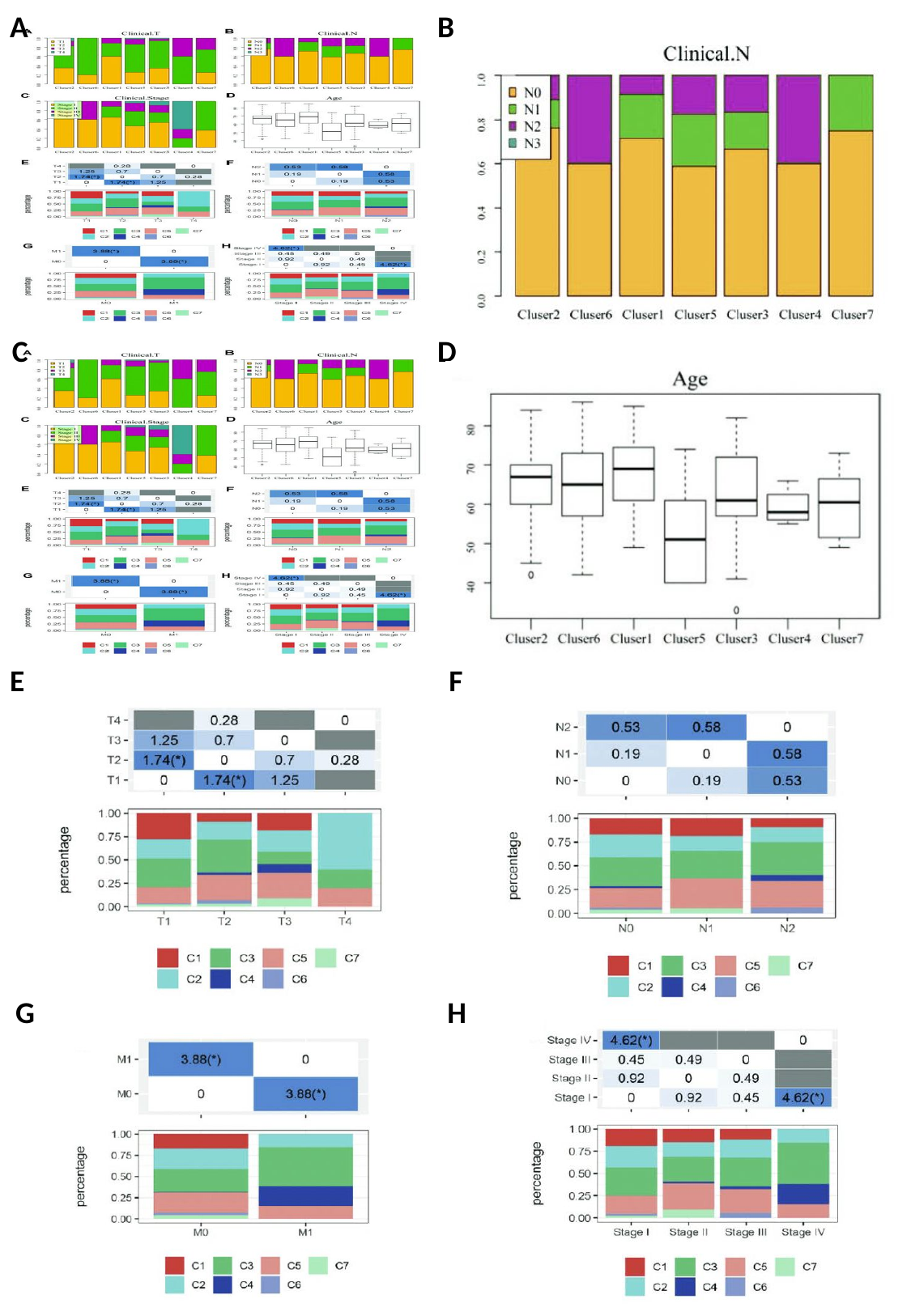

A
B
C
D
E
F
G
H

## Slide 2
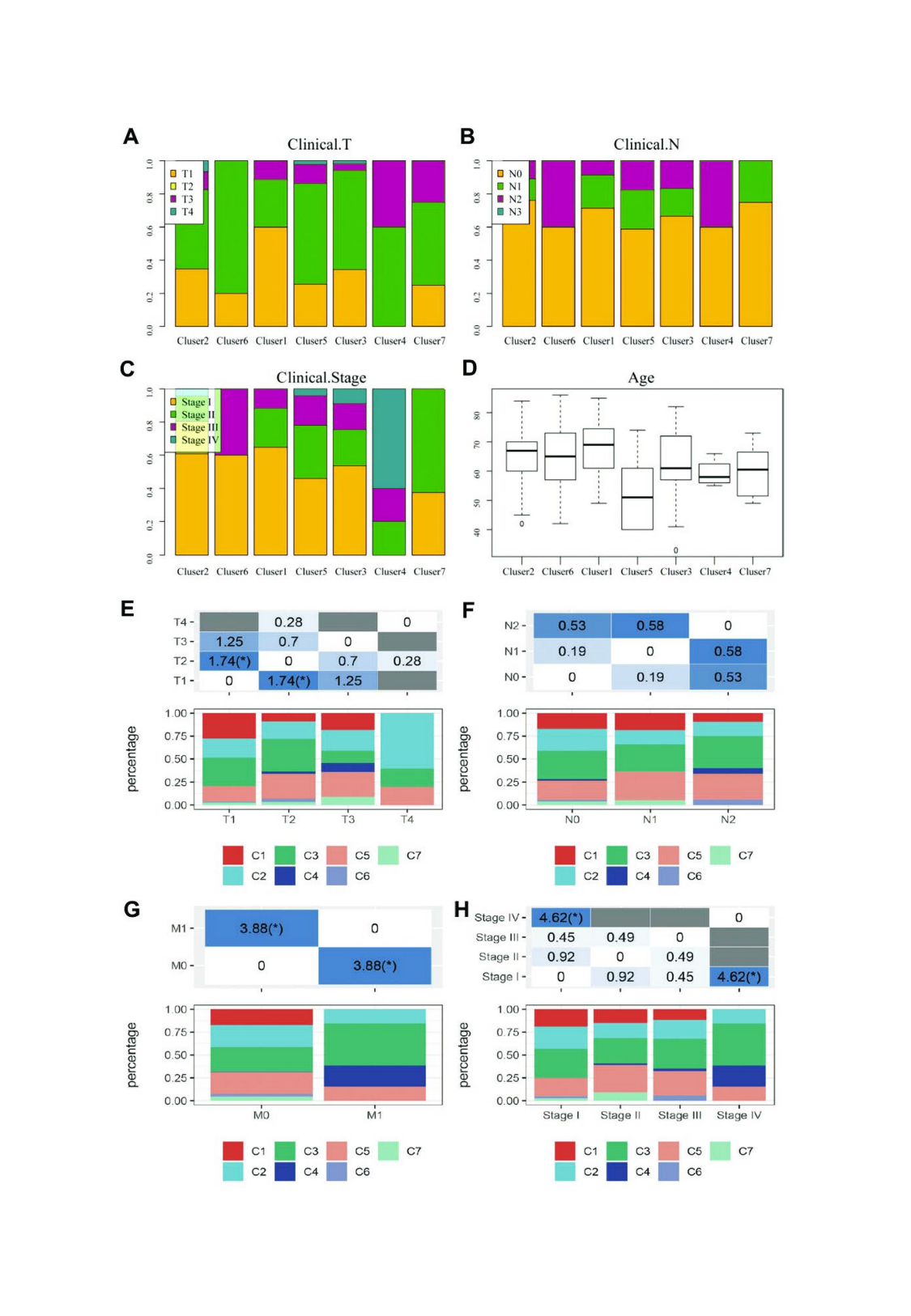

## Slide 3
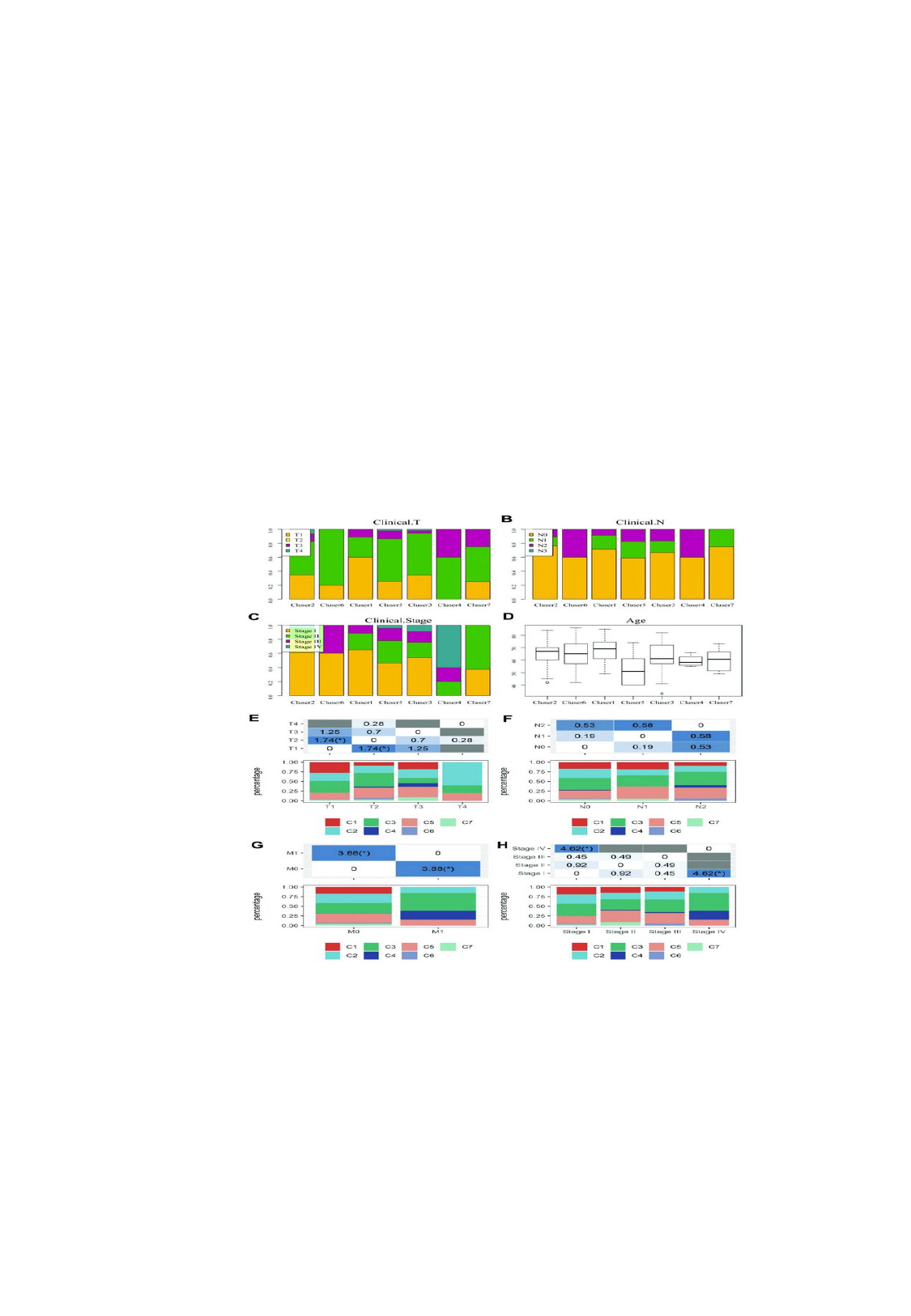

Supplement: Supplementary 6 — Supplementary Figure 6: the clinical value of the LUAD methylation subtype-specific prognostic model in the validation dataset. The proportions of different tumor sizes (a), lymph node metastasis statuses (b), and TNM stages (c) in subtype-specific classification. The age distributions in subtype-specific classification (d). In the ANOVA test, the distributions of the seven subgroups in T stage (e), N stage (f), M stage (g), and TNM stage (h) in the validation set were pairwise compared. The gray area represents NA, and the values in the table are −log10 (p value). [file 9389372.f6.pptx]
