# Supplementary material for: Mining the Prognostic Role of DNA Methylation Heterogeneity in Lung Adenocarcinoma
Source: Dis Markers. 2022 May 28;2022:9389372. doi: 10.1155/2022/9389372 (PMC9168807; doi:10.1155/2022/9389372)
Supplement: Supplementary 7 — Supplementary Table 1: the discovered methylation sites linked to LUAD survival by univariate and multivariate Cox regression. [file 9389372.f7.pdf]

Table S1

Univariate Cox regression				
	p.value	HR	Low 95%CI	High 95%CI
cg26697158	2.00E-07	0.00087066	6.11E-05	0.01240095
cg03025013	3.00E-07	2521.5923	126.01745	50456.7246
cg23389061	5.39E-07	37667.9174	611.543789	2320147.84
cg24237439	5.75E-07	67.9228467	12.9953428	355.012805
cg24436578	1.28E-06	328.472085	31.4849662	3426.83903
cg20354848	1.31E-06	16940.577	327.894513	875230.109
cg08785274	1.60E-06	52.8963191	10.4599376	267.498784
cg14038647	2.49E-06	4.79E-05	7.63E-07	0.00300663
cg03942855	2.61E-06	29.3302564	7.16691879	120.032607
cg10959711	2.72E-06	172758.688	1120.19674	26643145.3
cg07113947	2.79E-06	2.5903E+13	63528109	1.0562E+19
cg20121803	2.79E-06	7.97E+21	5.496E+12	1.16E+31
cg02336869	3.13E-06	1126.14751	58.7157095	21599.1296
cg14565265	3.17E-06	10.0647825	3.81051651	26.5842821
cg19224203	3.27E-06	17471558.8	15548.3149	1.9633E+10
cg09306340	3.38E-06	99.9174582	14.3258266	696.888127
cg21437481	3.57E-06	86.1388136	13.0889978	566.880316
cg09655116	3.71E-06	235100.178	1247.20422	44316795
cg25144930	4.08E-06	1560.82115	68.3606783	35636.9003
cg13300301	4.71E-06	0.07383721	0.02419168	0.22536402
cg08279757	5.47E-06	452321.427	1648.46508	124112228
cg07871500	5.61E-06	5002.34912	126.581329	197687.108
cg10463708	6.44E-06	25.0215951	6.17787667	101.342298
cg04006913	6.98E-06	7278691548	364111.686	1.455E+14
cg22434226	7.35E-06	31672.0622	341.272159	2939353.52
cg17150246	7.48E-06	48.4359812	8.86851771	264.536234
cg15696662	7.59E-06	12.1956257	4.07972401	36.4567029

Multivariate Cox regression				
	p.value	HR	Low 95%CI	High 95%CI
cg07219542	7.08E-07	103.155098	16.5088788	644.560682
cg02337836	5.42E-06	148.052666	17.1811057	1275.79636
cg24237439	6.10E-06	118.317374	14.9524332	936.235643
cg10463708	7.19E-06	52.062116	9.26602794	292.516269
cg02709432	7.38E-06	38.448822	7.79584763	189.628118
cg14565265	8.61E-06	14.0577266	4.38807397	45.0356301
cg24073738	1.64E-05	1.1925E+16	581566476	2.45E+23
cg06498232	2.06E-05	171.311278	16.0608167	1827.27657
cg02156680	2.32E-05	43.7945623	7.60669604	252.141492
cg02874942	2.50E-05	84.0269719	10.7028099	659.689566
cg04452959	2.91E-05	53.3203522	8.26603691	343.944745
cg04436714	2.98E-05	36.0558989	6.70015273	194.029584
cg03942855	3.46E-05	25.8621444	5.54716859	120.575119
cg10305451	3.93E-05	16.3234086	4.31184297	61.7957724
cg06998282	4.53E-05	13.1375517	3.810909	45.2897891
cg07696485	5.13E-05	118.216366	11.7364863	1190.74047
cg03070194	5.26E-05	617.384777	27.4136285	13904.1778
cg08440349	6.42E-05	1.2921E+10	142241.814	1.1737E+15
cg05305140	7.73E-05	56.4678447	7.64089681	417.309324
cg09992998	7.86E-05	2.9297E+11	596014.796	1.4401E+17
cg02288341	8.89E-05	21.2734946	4.61079224	98.1526708
cg24148025	9.04E-05	1.2228E+11	344039.101	4.3463E+16
cg17380238	9.54E-05	71.2501564	8.35898237	607.320911
cg23924887	9.80E-05	8881.20335	91.6013792	861076.259
cg14071579	0.00010196	17.3375997	4.1121408	73.0987527
cg09257526	0.00010352	103.702374	9.95629097	1080.13943
cg20121803	0.00010654	2.44E+25	3.5273E+12	1.69E+38

cg09578291	8.23E-06	178.799758	18.295696	1747.37018
cg23651323	8.76E-06	233.960494	21.1184139	2591.93294
cg01803049	9.63E-06	0.00122641	6.30E-05	0.02388142
cg04852280	9.89E-06	1252308.74	2475.08844	633624703
cg17132124	1.00E-05	43.9233371	8.19895258	235.30561
cg25320115	1.01E-05	31.0879147	6.76284271	142.90713
cg14647739	1.08E-05	92100.4529	566.853608	14964169.4
cg07219542	1.16E-05	33.410512	6.96169503	160.343466
cg02541231	1.18E-05	9136.54827	154.536657	540172.9
cg11248254	1.37E-05	1434.94455	54.2089138	37983.8984
cg03188198	1.40E-05	4.1769E+11	2381455.66	7.3261E+16
cg23755113	1.44E-05	3866730.36	4077.99739	3666408350
cg19604470	1.48E-05	31359.0235	289.559276	3396155.6
cg04670377	1.57E-05	4813912.94	4461.34231	5194346498
cg06218124	1.58E-05	832861.993	1707.54051	406232880
cg11641045	1.71E-05	153.160517	15.4507665	1518.25115
cg10801634	1.81E-05	1.7141E+12	4370314.1	6.7232E+17
cg13666340	1.87E-05	6373.29239	115.37138	352070.469
cg15240082	1.95E-05	1834.34566	58.2978902	57717.7663
cg23574449	1.99E-05	70895.7854	418.683077	12004813.8
cg27131958	2.04E-05	2457.06813	67.6894634	89189.4172
cg10293354	2.05E-05	173453.409	673.415033	44676883.5
cg06208248	2.11E-05	12787274.1	6784.08321	2.4103E+10
cg07099627	2.20E-05	446320.957	1097.56957	181494096
cg06993761	2.21E-05	6866514.04	4771.52539	9881329589
cg20384132	2.26E-05	53.8148821	8.51786477	339.996186
cg00246980	2.27E-05	33034.6878	267.980585	4072274.86
cg12614442	2.31E-05	93184841	19022.2491	4.5649E+11
cg04021962	2.42E-05	21866497	8554.70353	5.5892E+10
cg15152115	2.52E-05	595.051155	30.4676418	11621.7028

cg22965432	0.00011239	29.2308853	5.27177	162.079274
cg06033194	0.00011936	84381.9691	261.049354	27275749.2
cg12208770	0.00012031	793.638474	26.4087014	23850.5491
cg20063141	0.00012214	77.7895731	8.43785362	717.151299
cg13300301	0.00012365	0.08354381	0.02352213	0.29672347
cg00069002	0.00014938	901.084248	26.7656321	30335.6491
cg00818480	0.00015018	491530859	15765.2216	1.5325E+13
cg10616515	0.00015108	9756012778	66443.0258	1.4325E+15
cg16601494	0.00015171	7.44442537	2.63489997	21.032855
cg08599448	0.00015655	300.766949	15.6097774	5795.13437
cg02336869	0.00015941	863.630665	25.8328742	28872.4329
cg17929951	0.00017417	30.08471	5.08699067	177.922437
cg03317642	0.00019072	294.811019	14.8678113	5845.75197
cg03180980	0.00019652	1082.37781	27.3608277	42818.212
cg20384132	0.0002026	80.665001	7.96084801	817.355434
cg02318139	0.00020836	29.8276812	4.95802608	179.444511
cg02501882	0.00021126	4023.02871	49.8665784	324561.27
cg16306898	0.00021144	9.88173369	2.94138794	33.1981578
cg18811550	0.00021328	304.988599	14.7666657	6299.19085
cg08254307	0.00021502	631.805145	20.7649714	19223.6114
cg08785274	0.00021989	48.4007751	6.18222081	378.930986
cg26410550	0.00022162	0.027789	0.00414953	0.18610008
cg23098599	0.00022194	9.2408E+11	411707.413	2.0741E+18
cg00536924	0.00022366	39.5174634	5.60790892	278.469201
cg15696662	0.00022566	9.87807331	2.9249553	33.3599396
cg00350296	0.0002333	59.4180473	6.74703079	523.267857
cg13939204	0.00023332	21.9844823	4.23923805	114.010456
cg26709300	0.00023554	86.7292439	8.03887267	935.698581
cg09937039	0.00023839	33.6949614	5.16118454	219.978652
cg01111358	0.00023955	28.0334512	4.73385677	166.011442

cg24692861	2.57E-05	34452.3003	265.653426	4468080.9
cg17114257	2.58E-05	528.258707	28.4757894	9799.80775
cg06892635	2.65E-05	1.5769E+12	3217329.04	7.7291E+17
cg11827182	2.75E-05	139.62962	13.8813643	1404.50394
cg08446657	2.81E-05	264541.345	767.131686	91225697.4
cg21116657	2.87E-05	2023649.54	2247.68817	1821941985
cg04436714	2.88E-05	18.858582	4.76270686	74.673106
cg08511084	2.88E-05	186908.356	632.940537	55194337.6
cg20240860	2.90E-05	2863.13637	68.6151833	119471.369
cg00170536	2.95E-05	113755.647	482.745805	26805716.6
cg12208770	3.02E-05	595.560644	29.5983498	11983.5222
cg02288341	3.02E-05	15.4685467	4.27195184	56.0109167
cg27265272	3.15E-05	37353.3773	262.57548	5313804.6
cg16598405	3.17E-05	12819.9588	148.955293	1103360.21
cg10571951	3.17E-05	174.989136	15.3621544	1993.28797
cg26375182	3.21E-05	12330084.8	5603.21287	2.7133E+10
cg22112476	3.23E-05	98913.2577	436.288954	22425120.9
cg26145223	3.24E-05	3813.51215	78.0497812	186328.196
cg21870145	3.38E-05	33.401438	6.36095622	175.39125
cg17252669	3.54E-05	228264541	24938.9647	2.0893E+12
cg13527630	3.62E-05	553926.917	1042.46183	294336944
cg04511534	3.73E-05	67.4273291	9.111069	499.002336
cg08548478	3.73E-05	24210.9939	199.607684	2936621.55
cg24495844	3.88E-05	2273147.17	2130.76326	2425045597
cg16635274	3.91E-05	1400.51145	44.3401921	44235.9908
cg07644571	4.00E-05	13892174.3	5425.04842	3.5574E+10
cg02337836	4.05E-05	49.5013782	7.68221983	318.96854
cg16441026	4.09E-05	1.267E+16	256473343	6.26E+23
cg12931029	4.13E-05	485.971402	25.2570015	9350.60341
cg24888944	4.36E-05	492629531	33471.3637	7.2505E+12

cg23511432	0.00024325	13.9567163	3.41419728	57.0529212
cg24353213	0.00024382	18.2233827	3.86496647	85.9235595
cg26872028	0.00024662	18.1471312	3.8526664	85.4780396
cg06834005	0.00024972	30.7566605	4.91686615	192.393313
cg00674365	0.00027269	8.51968085	2.68799338	27.0034005
cg09802835	0.00027367	17.7690128	3.77225216	83.7000825
cg05436939	0.00027473	493.8063	17.4752583	13953.7086
cg02126424	0.00029402	3173659126	22797.6844	4.418E+14
cg20631104	0.00029619	56.0968838	6.33363398	496.849105
cg09655116	0.00029698	218106.051	279.584343	170146328
cg18442524	0.00030853	18.2901702	3.77183207	88.6917336
cg07004443	0.00030864	733.288732	20.3588932	26411.6697
cg00089453	0.0003145	307.441217	13.6297458	6934.83966
cg06055845	0.000316	125.514315	9.0504111	1740.67709
cg04734210	0.0003168	7275925109	31222.0426	1.6956E+15
cg03033176	0.00032129	13517.0594	75.9147074	2406791.78
cg26296364	0.00032454	39.4095167	5.31696291	292.104728
cg09913183	0.00033618	1299100183	13554.155	1.2451E+14
cg03576419	0.00034277	26.1811117	4.38338416	156.374752
cg22517356	0.0003464	67.4495645	6.7152568	677.478746
cg14021375	0.00034701	14.0706818	3.30510657	59.9024818
cg19670286	0.00034706	17.1828129	3.61754261	81.6159177
cg10784298	0.00035927	7019266.9	1218.57185	4.0433E+10
cg05888175	0.00036014	1432.02836	26.4352765	77574.5711
cg02154252	0.00036175	121.043472	8.67505669	1688.92522
cg27002699	0.00036663	542.37028	16.9900712	17313.9663
cg06834274	0.00036975	717.545751	19.2265467	26779.2189
cg02242344	0.00037417	98.4288877	7.85389141	1233.55996
cg21940568	0.0003798	93.5627772	7.65589066	1143.43238
cg17150246	0.00038041	54.7711056	6.01988788	498.327223

cg11356794	4.40E-05	529.235774	26.1241947	10721.4981
cg26198823	4.41E-05	0.00060428	1.73E-05	0.0211612
cg05446724	4.43E-05	4583670152	105823.954	1.9854E+14
cg21289924	4.50E-05	37697.344	238.812169	5950658.84
cg10282345	4.65E-05	3.0306E+17	1171280922	7.84E+25
cg23794671	4.75E-05	715.005161	30.1271281	16969.1708
cg08774802	4.89E-05	13121.8045	135.060042	1274853.4
cg04151290	4.95E-05	75480.1239	332.666234	17126021.6
cg01511567	4.97E-05	96.0080074	10.5835574	870.929987
cg05966809	5.26E-05	897105.602	1168.07019	688998371
cg27162464	5.27E-05	430.551215	22.7629103	8143.70157
cg24005914	5.30E-05	64606.3511	300.361816	13896508.7
cg25230157	5.38E-05	32665.5843	210.558616	5067664.38
cg22965432	5.38E-05	24.594798	5.1978616	116.375567
cg07375912	5.39E-05	20840537.4	5843.18366	7.4331E+10
cg05013422	5.46E-05	322533.474	680.438312	152883575
cg00150500	5.53E-05	27429984.2	6645.14645	1.1323E+11
cg18611777	5.55E-05	150.141117	13.1312755	1716.69196
cg20879085	5.55E-05	11410108	4226.72105	3.0802E+10
cg04900486	5.65E-05	4082274.18	2472.68959	6739609621
cg22077262	5.66E-05	56906.3301	275.727138	11744692.3
cg24372325	5.67E-05	1.2862E+10	154207.442	1.0728E+15
cg13494509	5.79E-05	56559.0963	272.894756	11722216.4
cg19824710	5.85E-05	51.689958	7.54716144	354.02075
cg02602480	5.89E-05	59302960.8	9564.53121	3.677E+11
cg10283505	5.94E-05	0.07119077	0.01959864	0.2585958
cg11145055	6.18E-05	22057.0621	165.354982	2942239.67
cg11797365	6.24E-05	595.721043	26.0935241	13600.4458
cg03553689	6.27E-05	1.4405E+14	16785270.6	1.24E+21
cg08229199	6.45E-05	196.835145	14.7508029	2626.57394

cg25300386	0.00038301	40.1223247	5.23036653	307.779758
cg11382133	0.00038419	36.8672433	5.03348354	270.030411
cg18132916	0.00038434	13.8201548	3.24295566	58.8958647
cg14543527	0.00038539	7.7547E+11	211399.944	2.8446E+18
cg10571951	0.00038719	308.090954	13.0063653	7297.96787
cg22144668	0.00038816	369365309	6834.07154	1.9963E+13
cg21295088	0.0003895	72.6952812	6.80649028	776.406591
cg18140857	0.0003938	77.8788497	7.0057611	865.732521
cg05987251	0.00039803	22.2943007	3.99984169	124.263879
cg01518065	0.000399	2.5784E+12	347611.691	1.9125E+19
cg22505746	0.00039994	146231595	4410.89102	4.8479E+12
cg01056889	0.00040329	16.0994917	3.45345013	75.0535326
cg21751146	0.00040376	1489254493	12325.8488	1.7994E+14
cg25120284	0.00040484	106.038166	7.99945809	1405.60681
cg00173504	0.00041345	44.2991867	5.40274539	363.226064
cg00476675	0.00041792	0.0303753	0.00436126	0.21155768
cg23651323	0.00042633	242.558147	11.4326179	5146.19267
cg15571373	0.0004361	0.0507595	0.00964202	0.26721862
cg04511534	0.00043711	80.9479188	6.99319193	936.992094
cg02223962	0.00044356	103953228	3497.33961	3.0899E+12
cg07311956	0.00045026	23.90742	4.05964884	140.791668
cg19724344	0.00045337	11809.1431	62.560407	2229139.27
cg03707974	0.00045357	80.981424	6.94664138	944.052049
cg19785066	0.00046052	11.1651801	2.8941982	43.072809
cg19839655	0.00046892	16.5261178	3.43241914	79.5685365
cg08724901	0.00047086	5702816.37	931.736264	3.4905E+10
cg11525280	0.00047346	13.4218424	3.12901136	57.5727705
cg19584674	0.00047963	1255.91758	22.8844827	68925.699
cg18301703	0.00048247	259.430391	11.4403398	5883.05323
cg11841529	0.00048354	15.0182924	3.2793097	68.7794466

cg16568036	6.64E-05	224.675745	15.7061235	3213.98151
cg04846715	6.69E-05	2999.10346	58.5767957	153552.639
cg17536086	6.89E-05	10168.1795	108.111512	956344.724
cg12605679	6.90E-05	6498.59887	86.1134499	490420.339
cg02242344	6.92E-05	39.7433263	6.47855166	243.80943
cg10671802	7.09E-05	44509918.6	7508.69955	2.6385E+11
cg00818480	7.10E-05	30673183.4	6211.36494	1.5147E+11
cg01850934	7.19E-05	184846.059	463.988885	73639836.3
cg26785250	7.22E-05	2165180197	53137.2678	8.8224E+13
cg14808521	7.31E-05	2599144085	57795.5232	1.1689E+14
cg07994696	7.55E-05	84.3154146	9.38231016	757.712014
cg25300386	7.69E-05	36.2785754	6.11710846	215.156399
cg22249338	7.76E-05	63775.8275	263.974307	15408151.7
cg08716348	7.79E-05	83832.6446	302.580842	23226560.7
cg07469063	7.81E-05	1024.05523	32.8652513	31908.7507
cg02851517	7.83E-05	3525409.6	1986.75219	6255693536
cg09552761	7.87E-05	144865.311	397.146565	52841847.6
cg19605920	7.92E-05	20020.0427	146.330379	2739021.88
cg24353213	8.02E-05	13.5921086	3.71602129	49.7159199
cg20184247	8.14E-05	44.6223672	6.74601545	295.160257
cg07234508	8.16E-05	269.699146	16.6543354	4367.48916
cg02019072	8.17E-05	1251.58943	36.0096807	43501.5273
cg02029665	8.27E-05	69.8184081	8.42995765	578.248469
cg26410550	8.30E-05	0.03671141	0.00708056	0.19034195
cg19104830	8.57E-05	2410566.56	1575.46795	3688320769
cg23227945	8.61E-05	7320904.62	2742.69496	1.9541E+10
cg17147466	8.63E-05	812.058607	28.6477239	23018.9031
cg26709300	8.82E-05	43.0547639	6.56479194	282.371888
cg26461589	8.84E-05	119205.456	345.503657	41128192
cg21527360	8.85E-05	23186.0687	152.335323	3529015.91

cg05952925	0.00048714	16.5054814	3.41483753	79.7785884
cg10959711	0.00048813	261938.81	236.002466	290725521
cg14720706	0.00049046	33.1714186	4.63138528	237.583993
cg05369939	0.00049432	2725163224	13400.6327	5.5419E+14
cg04524239	0.00050238	0.02150277	0.00247288	0.18697621
cg12249359	0.00050448	262632967	4734.56294	1.4569E+13
cg08864344	0.00051332	22.9814102	3.9196695	134.742283
cg15579745	0.00052181	7312.32259	47.9821205	1114374.71
cg22510727	0.00052318	119.577492	8.010407	1785.02497
cg23321951	0.00052984	234298357	4322.2514	1.2701E+13
cg09053081	0.00053617	11.9507602	2.93388709	48.6796746
cg06849504	0.00054351	87.2227508	6.93117532	1097.62167
cg17102205	0.00054964	12.7102515	3.00499901	53.7605814
cg02369725	0.00055061	96.2099215	7.21308511	1283.27184
cg16411949	0.00055102	2.4297E+11	84343.8862	6.9995E+17
cg11097541	0.00056635	127.806693	8.10682373	2014.91377
cg07392724	0.00056842	2262903790	10825.4644	4.7303E+14
cg03714619	0.00057336	11.9930463	2.91676045	49.3126406
cg25975979	0.00057381	259.143836	10.9619491	6126.23968
cg14548272	0.00057534	54.2981443	5.58773094	527.636085
cg03578689	0.00058042	71.0911529	6.26492292	806.706177
cg10763374	0.00058381	0.02646481	0.00333979	0.20970978
cg15742848	0.00058776	13.3998793	3.05073349	58.8569162
cg17861791	0.0005898	52568.4632	106.692028	25901122.9
cg17668148	0.00059142	293138.129	222.897448	385513443
cg23061046	0.00059328	27.215078	4.13093434	179.296113
cg13656831	0.00059919	45.8724003	5.16010439	407.797392
cg11314748	0.00060022	0.02700689	0.00343231	0.21250179
cg03434509	0.00060201	20.9165247	3.68208582	118.818796
cg01392273	0.00060281	3424545116	12215.3873	9.6006E+14

cg06762403	8.85E-05	707.794836	26.6097016	18826.7249
cg17432022	8.97E-05	479722.656	689.027202	333998173
cg22657536	8.98E-05	89.454012	9.44013022	847.659945
cg05588972	9.06E-05	539.840163	23.1332847	12597.7528
cg15680989	9.23E-05	15905.5044	124.596134	2030440.77
cg08491668	9.38E-05	16698.4922	127.035225	2194978.92
cg21253692	9.54E-05	99165064.1	9545.57694	1.0302E+12
cg22115596	9.58E-05	207545.328	442.163421	97418875
cg19247841	9.70E-05	30.2494504	5.44763899	167.968042
cg12114985	9.79E-05	78.8688336	8.76139817	709.965783
cg17013432	9.88E-05	3323659.81	1732.28136	6376974747
cg20747741	9.91E-05	146.490003	11.8942963	1804.16904
cg05021846	0.00010024	23.6802634	4.80737327	116.644755
cg03278488	0.00010086	200.076907	13.8453042	2891.28848
cg17102205	0.00010115	11.7455234	3.3924796	40.6656301
cg09758595	0.00010289	2601948.73	1505.60199	4496631427
cg04450332	0.00010328	32.0700396	5.5698077	184.65403
cg21751146	0.00010342	144858355	10991.6845	1.9091E+12
cg02318139	0.0001036	23.9992891	4.82345991	119.40928
cg06295308	0.00010473	2.6109E+12	1391487.71	4.8989E+18
cg11275803	0.00010485	165921.727	382.359046	72000440.3
cg23719130	0.00010841	58852.639	226.316723	15304362.3
cg02884176	0.00010889	26.5098568	5.04081609	139.416415
cg27105630	0.00011081	1734130.75	1190.45857	2526093321
cg25994146	0.00011094	261.523523	15.5482383	4398.86189
cg26329618	0.00011109	45.4434992	6.5608425	314.763176
cg05305140	0.00011131	27.4988941	5.12080686	147.669927
cg13939204	0.00011203	15.4243613	3.84892141	61.8123618
cg14780540	0.00011237	39865.985	184.465125	8615703.19
cg07226281	0.00011267	60415.9363	226.171008	16138608.5

cg10832166	0.00060385	0.03151367	0.00437002	0.22725539
cg01411912	0.00061564	54.1269011	5.51259188	531.459881
cg14280533	0.00061926	16.31038	3.29784716	80.6673208
cg04566159	0.00062136	50.6587563	5.3498262	479.699619
cg12033622	0.00062845	76.8555085	6.37836046	926.063871
cg24398450	0.00062955	24.8791147	3.9405657	157.076521
cg22995449	0.00062998	73.9302373	6.27060116	871.635723
cg12615761	0.00063683	8.18636981	2.44966894	27.3574316
cg16097357	0.00063767	9.91169379	2.65730592	36.9704042
cg27458485	0.00063807	40.5230787	4.84136437	339.185358
cg07245678	0.00063871	448.698148	13.482018	14933.2264
cg07722358	0.00064019	22.9132622	3.79545478	138.327978
cg04573510	0.00064321	2.8791E+11	75517.3739	1.0977E+18
cg20725013	0.00064359	21.9265769	3.72217558	129.164991
cg15215033	0.00064432	530.787192	14.4459396	19502.715
cg06324635	0.00064541	20627.7906	68.5376947	6208346.33
cg12940439	0.00064701	13.8150551	3.05579463	62.4569945
cg02461363	0.00064858	39.8857465	4.79551166	331.74203
cg20018469	0.00065967	6936655.81	802.11293	5.9988E+10
cg05467676	0.00066017	34.2552519	4.48202991	261.805992
cg12727795	0.00066131	41.8030238	4.87591426	358.392848
cg23560546	0.00066503	36.431877	4.59518481	288.841845
cg23195373	0.00066927	289.983824	11.0584291	7604.21012
cg15979473	0.00067038	187.483713	9.1883077	3825.52955
cg20864214	0.00067078	35.3005781	4.52768008	275.225015
cg09310612	0.00067867	1471632594	7584.04371	2.8556E+14
cg12931029	0.00067872	329.945525	11.6364714	9355.41764
cg23794671	0.00067873	694.620144	15.9456997	30258.7628
cg25800170	0.0006817	76.2182272	6.25339474	928.970328
cg16058797	0.00068494	23.2688837	3.783108	143.120669

cg18328334	0.00011279	44.8772378	6.50833046	309.444409
cg21305265	0.00011387	106.782993	9.9593195	1144.91834
cg19703969	0.00011434	1360.04553	34.8024422	53149.2543
cg07907506	0.0001153	24.4932808	4.81938836	124.480694
cg14986845	0.00011537	103233826	8714.2323	1.223E+12
cg08650580	0.00011627	16717.3559	118.954723	2349381.2
cg17328794	0.00011787	1186198.5	960.134507	1465489331
cg26329020	0.00011844	12191.0058	101.287267	1467317.93
cg25492645	0.00011919	254690.955	449.083537	144444134
cg18617679	0.00012044	525.178723	21.5639509	12790.4526
cg01431908	0.00012065	18.1998332	4.14689983	79.8750736
cg26283550	0.00012111	2187920.57	1280.24818	3739115986
cg21568286	0.00012308	1913565433	35011.58	1.0459E+14
cg24457521	0.00012461	2775.50061	48.3505535	159324
cg06161720	0.00012602	36509.7615	169.881679	7846418.11
cg19670286	0.00012672	12.7683091	3.47125125	46.9656923
cg09728506	0.00012816	203.848724	13.4131726	3098.02189
cg02369725	0.00012824	74.3380701	8.19579982	674.265939
cg09974990	0.00012945	147322661	9672.08582	2.244E+12
cg18442524	0.00012971	13.6124891	3.57438707	51.8410167
cg15132559	0.00013091	34455.5252	162.993741	7283612.27
cg15270687	0.00013184	9495.97675	86.7649933	1039285.21
cg18224445	0.00013361	357206.723	505.376003	252478634
cg10599693	0.00013387	1094189.07	870.834825	1374829854
cg22907891	0.00013414	0.00011907	1.15E-06	0.01229985
cg06961086	0.00013757	0.00179664	6.97E-05	0.0463353
cg16593556	0.00013806	4266.31669	58.0035729	313798.913
cg23431851	0.0001387	9.62323137	3.00288514	30.8392022
cg21194918	0.0001393	1.2521E+10	79861.7447	1.9631E+15
cg04524239	0.00014054	0.02982024	0.00488862	0.18190128
cg15832300	0.00069075	151.721929	8.34163165	2759.59726
cg07833852	0.00069231	0.03335412	0.0046768	0.23787571
cg26861703	0.00069719	19.8919907	3.53170599	112.039704
cg04316429	0.00069874	256.965123	10.3898557	6355.34085
cg13747794	0.00070212	1665.79047	22.820176	121596.691
cg06526620	0.00070573	0.04855853	0.00843569	0.27951833
cg26186134	0.00071276	106114744	2389.01774	4.7134E+12
cg26094150	0.0007128	468.566548	13.3099472	16495.5282
cg21437481	0.00071395	76.310195	6.19827969	939.493884
cg06611606	0.00071614	4649748356	11670.0579	1.8526E+15
cg23840481	0.00071871	16801883.5	1092.64574	2.5837E+11
cg16748413	0.00071884	29.2234427	4.13401392	206.581211
cg25087049	0.00071917	3333.65137	30.2962748	366818.413
cg05392364	0.00072126	41.5311115	4.78966105	360.115926
cg24850474	0.00072394	539.144698	14.055059	20681.3081
cg19824710	0.00072562	42.1242577	4.81318913	368.664733
cg03188198	0.00072904	6.622E+11	91860.3863	4.7736E+18
cg02884176	0.00073036	31.9361752	4.28012423	238.291982
cg01026744	0.00073104	111.298357	7.22718577	1713.99001
cg05673638	0.00073139	4260874046	11004.0844	1.6498E+15
cg19568591	0.00073264	18.2939089	3.38606247	98.8366592
cg08918020	0.00073302	240.00732	9.9711069	5777.04304
cg25347419	0.00074051	9.15666363	2.52977958	33.1430017
cg25320115	0.0007429	27.0816995	3.98329736	184.123449
cg23687909	0.00074544	52.469367	5.25174786	524.212996
cg12027420	0.00074552	55.2619337	5.36694856	569.016319
cg23389061	0.00074662	9839.33669	46.9864937	2060433.52
cg13440692	0.00075339	80.052963	6.25436356	1024.64093
cg07164388	0.00075683	3230.20628	29.319204	355883.899
cg09317036	0.00076539	3606.99765	30.5685448	425615.028

cg16489547	0.00014233	148.64678	11.2980334	1955.72665	cg04122324	0.00077669	1336352.06	357.737055	4992037593
cg03003256	0.00014271	101.070689	9.36724932	1090.53189	cg21225170	0.00077934	19.4465724	3.44380349	109.811486
cg18301703	0.00014481	152.601473	11.4094757	2041.04116	cg18817318	0.00078388	939.274882	17.2934856	51015.5862
cg07469555	0.00014517	152.089897	11.3861519	2031.53243	cg03361102	0.00078526	56.1226268	5.34810866	588.946381
cg06498232	0.00014643	54.6599825	6.92974791	431.1432	cg20009641	0.00078907	30.4650119	4.14371598	223.981797
cg07971863	0.00014728	160.441539	11.6558495	2208.46086	cg09643398	0.00079127	76.8986853	6.08770231	971.369409
cg20631104	0.00014769	33.3312937	5.44934487	203.873156	cg09484039	0.0007933	21.576983	3.58684135	129.79838
cg08427573	0.00014831	20034.139	120.067676	3342837.47	cg06517984	0.0007957	0.00322915	0.00011314	0.09216561
cg09819651	0.00014934	4261994115	44936.4444	4.0423E+14	cg02900183	0.00080011	171.606216	8.4774256	3473.77787
cg24148025	0.00015034	223148120	10759.1434	4.6282E+12	cg01105229	0.00080265	51.1560572	5.12440632	510.682024
cg18202861	0.00015037	24409.2735	131.474587	4531770.33	cg07570421	0.00080316	175550.859	150.539661	204717506
cg03576419	0.00015041	16.643708	3.88828292	71.2430194	cg10283505	0.00080591	0.08706536	0.02087985	0.36304745
cg00863716	0.00015056	36.5620026	5.68531933	235.128399	cg09306340	0.00080679	84.0014432	6.28939302	1121.92742
cg09139451	0.0001508	20093.0993	119.569908	3376540.52	cg00405699	0.00080939	25.7779165	3.85008362	172.593909
cg19858931	0.00015171	7031.802	71.8913444	687791.273	cg09477740	0.00081342	87.9625665	6.39945652	1209.07347
cg00536792	0.00015183	56.873214	7.02922778	460.159007	cg06295308	0.00082046	1.1721E+13	258927.254	5.31E+20
cg02900183	0.00015296	152.915691	11.3149262	2066.58074	cg04450332	0.00082581	33.0761153	4.25505551	257.11284
cg08042370	0.00015448	184.821646	12.3762613	2760.04524	cg07174453	0.00082904	3283.23859	28.489168	378377.341
cg15494117	0.00016114	3636.14595	51.4170548	257143.421	cg13730743	0.00083573	55.4488407	5.25724485	584.826087
cg02809401	0.0001627	121875.958	276.96267	53630870.5	cg15593337	0.00083739	43.4707986	4.75243055	397.630289
cg06997416	0.00016279	1.9222E+15	21851049.4	1.69E+23	cg09139451	0.00084302	30335.6868	70.8776671	12983693.3
cg12563239	0.00016384	21922.8121	121.22424	3964633.55	cg20899053	0.00085131	14164.6322	51.5183809	3894470.3
cg03368930	0.00016508	5885741.61	1768.84995	1.9584E+10	cg27157482	0.00085865	107.215944	6.86163076	1675.29542
cg19065831	0.00016579	21.8983922	4.39377768	109.140612	cg00481884	0.00086294	459.423218	12.4775241	16915.9917
cg09042952	0.00016657	5263.94988	60.8234108	455567.486	cg00536792	0.00086806	87.0604877	6.28246275	1206.45817
cg23511432	0.00016668	9.74951331	2.97931333	31.9043347	cg27181253	0.00087281	132.7199	7.46277958	2360.32322
cg23876285	0.00016843	48.5209217	6.42130422	366.635774	cg23338737	0.00087328	8.5521E+12	207500.962	3.52E+20
cg21388169	0.00016844	5416766.76	1681.50566	1.7449E+10	cg05894557	0.00087524	0.02591335	0.00301406	0.22279011
cg16083849	0.00016932	331484.637	440.041321	249708514	cg12416569	0.0008787	31.8112721	4.14311569	244.250248
cg13556548	0.00016934	14.9963193	3.65695812	61.4963546	cg18592365	0.00088368	104.064545	6.73341313	1608.31206

cg09937039	0.00016941	24.6084582	4.63553642	130.637786
cg20593518	0.00016961	10100653.5	2257.60191	4.5191E+10
cg10355455	0.00016998	38340.3002	156.418591	9397723.21
cg18273501	0.00017066	1461.69249	32.7067721	65324.237
cg14637144	0.00017088	57.5540816	6.95440616	476.312748
cg22502206	0.00017184	14509.5147	97.8795217	2150868.88
cg15872683	0.00017217	852975.768	686.469613	1059868706
cg26580856	0.00017226	414368.765	485.904989	353364294
cg03114558	0.00017412	115134.538	262.218005	50553210
cg25851671	0.00017458	62.7271088	7.22449508	544.631858
cg20724073	0.00017466	44616414.6	4515.888	4.408E+11
cg06957820	0.00017513	205.735658	12.735808	3323.47669
cg26887625	0.00017529	245436.105	375.401931	160465029
cg02165978	0.0001758	1772448.11	963.802078	3259561679
cg25607670	0.00017611	32.798433	5.29418575	203.192192
cg01790464	0.00017664	27.1719522	4.8375179	152.622688
cg01503881	0.00017685	2184.26668	39.2627638	121515.158
cg22076118	0.00017795	15172.0521	98.8253913	2329271.48
cg09317036	0.00018069	2063.55971	37.9929272	112080.826
cg01105229	0.00018109	39.8819465	5.79144082	274.641441
cg00516051	0.00018232	894796.597	683.351268	1171668201
cg05082085	0.00018234	412271009	12680.3532	1.3404E+13
cg17942851	0.00018237	23.1508202	4.46571312	120.016772
cg04467958	0.0001833	199353776	8939.3643	4.4457E+12
cg20984053	0.00018679	148.508757	10.7768273	2046.50686
cg17803089	0.00018759	148.151177	10.7564123	2040.529
cg04986336	0.00018975	335.015672	15.8145629	7096.97141
cg10014563	0.00019014	43.3368217	5.98635797	313.726665
cg16257101	0.00019035	2106675.44	1004.92242	4416342331
cg04876465	0.00019103	9.9962E+11	495913.057	2.015E+18

cg09355771	0.00088443	14.0344178	2.95767295	66.5945447
cg21913319	0.00089218	11.8064338	2.75211924	50.6489244
cg14277392	0.00089621	29.5222269	4.00467962	217.635857
cg09279544	0.00089677	115.850098	7.01233305	1913.94863
cg26697158	0.00089901	0.00327413	0.00011176	0.09591827
cg24720717	0.0009052	6.24921805	2.11733484	18.4442846
cg03525527	0.0009068	2.55E+20	224911976	2.89E+32
cg22983529	0.00091114	29.3171017	3.98218829	215.834207
cg11827182	0.00091477	104.598717	6.69395246	1634.44418
cg08849717	0.00091571	258280876	2746.94874	2.4285E+13
cg02797271	0.00091922	27.2545015	3.85949437	192.462478
cg06727065	0.00092239	341.741413	10.8355961	10778.1051
cg03017520	0.00092464	8.49929878	2.39596289	30.1499159
cg10959198	0.00092488	14.7368942	2.99958714	72.401981
cg10307580	0.00092976	1205.6354	18.0822661	80385.7604
cg02015582	0.00093718	2282.6837	23.3903238	222769.249
cg11722699	0.00093749	20.3833158	3.41735858	121.579153
cg12269002	0.00094246	17.8864481	3.237616	98.8150006
cg17536822	0.00094333	70.5463576	5.66115737	879.111505
cg26436829	0.00094726	3.6882E+12	130611.625	1.04E+20
cg10667810	0.00095948	262.193501	9.61760164	7147.87682
cg06101723	0.00096088	29393699	1083.87896	7.9713E+11
cg00500299	0.00096281	2859192937	6948.81869	1.1765E+15
cg12848614	0.00096636	163.820651	7.92955764	3384.4518
cg04964562	0.00096761	5.457E+10	22884.7313	1.3013E+17
cg01362243	0.00096838	122.313467	7.03876557	2125.45567
cg24234651	0.00097321	12.2447816	2.76328222	54.2596324
cg03607638	0.00097726	55009.8404	83.6352064	36181922.4
cg07972191	0.00098094	132.10156	7.239152	2410.6169
cg00644823	0.00098138	40.6980282	4.49145492	368.773489

cg13021277	0.00019234	122459.543	259.208021	57854458.4
cg25349574	0.00019412	46.2341489	6.15576467	347.251177
cg06184463	0.00019496	3953.13631	50.6516904	308524.484
cg02319549	0.00019584	4.18E-05	2.07E-07	0.00842097
cg18425877	0.00019734	5468.77502	58.8529514	508173.329
cg04316429	0.00020074	175.464022	11.5127429	2674.22137
cg23600666	0.00020338	3089313049	30401.4035	3.1393E+14
cg05951178	0.00020397	0.05224886	0.01100489	0.24806637
cg22956431	0.00020399	289.536793	14.5416749	5764.91741
cg01804934	0.00020618	11.4412666	3.15855844	41.443774
cg26844213	0.00020908	27.9379856	4.80535289	162.429494
cg01158447	0.00021008	10140.7495	77.2221482	1331674.96
cg15631337	0.00021012	2.3678E+10	77362.0649	7.2469E+15
cg12992778	0.00021052	787214002	15535.9468	3.9889E+13
cg09539395	0.00021147	0.00082077	1.91E-05	0.03520941
cg26872028	0.00021161	14.0885045	3.47589108	57.1036185
cg06632353	0.00021373	17287520.4	2547.10955	1.1733E+11
cg00357238	0.00021539	24187390.2	2969.00476	1.9705E+11
cg12058507	0.00021591	0.00304398	0.00014133	0.06556056
cg17208816	0.00021864	1454.25831	30.5996912	69114.0061
cg18301624	0.00021945	6092.30617	59.9059503	619574.42
cg16717690	0.00022041	159426.134	277.00032	91756905.7
cg15528052	0.00022069	104445.076	226.995875	48057146.1
cg00850821	0.00022073	393403.125	423.007486	365870638
cg06649737	0.00022145	25.9973526	4.61353177	146.495652
cg04437762	0.00022162	28.6234526	4.82620368	169.761182
cg05487507	0.00022403	90.6510419	8.27512795	993.049468
cg08089542	0.00023066	789.048577	22.6618477	27473.3846
cg18188377	0.00023151	1403259.52	749.652346	2626733965
cg15599946	0.0002362	11890.8837	79.9575202	1768352.94

cg15118279	0.00098149	767114.969	242.772598	2423936558
cg10006582	0.00098475	401069.402	186.253658	863642988
cg22761176	0.00098815	14.0678835	2.91740024	67.8362001
cg19912332	0.00099704	1734244908	5461.79303	5.5066E+14
cg01869698	0.00099984	22032213.3	931.773721	5.2096E+11
cg05703053	0.00100937	14.9930346	2.98486919	75.3101974
cg01022780	0.00101351	39.975091	4.43206032	360.556442
cg01481989	0.00101796	17.1422949	3.14694325	93.3789553
cg01729739	0.00102029	555027637	3364.32775	9.1565E+13
cg26090107	0.00102345	8.31875318	2.34934739	29.4556926
cg01950845	0.00102446	13.9835728	2.89621043	67.5159184
cg13932362	0.00102479	19.2285461	3.29285373	112.284667
cg20237547	0.00102658	40.1119795	4.42745393	363.407712
cg16492377	0.00102877	33.880838	4.13463053	277.633315
cg04726446	0.00102967	20.8426455	3.39908939	127.803604
cg04260516	0.00103874	142946070	1913.27419	1.068E+13
cg03237606	0.00104082	95.1259792	6.25025607	1447.77299
cg17222164	0.00104315	11.6156602	2.68138593	50.3185911
cg09108404	0.00105493	1097511394	4273.78907	2.8184E+14
cg00358155	0.00105497	176160078	2049.77514	1.5139E+13
cg05211836	0.00106223	14.2416537	2.90323564	69.8616045
cg13255216	0.00106645	34.6798432	4.14631212	290.06295
cg09879099	0.00106772	171.855082	7.875804	3749.98785
cg21238818	0.00107277	44.599431	4.5811475	434.194543
cg12114985	0.00107479	64.1674386	5.29798403	777.174894
cg01731839	0.00107688	2708493761	6002.40079	1.2222E+15
cg07086112	0.00109654	358207.508	165.772194	774029807
cg14167139	0.00109965	1056.71994	16.1425414	69174.7973
cg03478631	0.00110472	26542.7227	58.3657992	12070701.3
cg05113000	0.00110569	234603968	2194.21116	2.5084E+13

cg18530716	0.00023713	28.1786835	4.75122752	167.122749
cg04394254	0.00023726	114.597441	9.14442804	1436.12848
cg01518459	0.00023802	0.00119598	3.30E-05	0.04328332
cg18993457	0.00023939	269364.593	341.126629	212698974
cg08081156	0.00024037	10.5286732	2.99721046	36.9853771
cg09597785	0.00024186	620103.034	500.73601	767925144
cg22235012	0.00024268	2057.96299	34.9810206	121071.701
cg19283196	0.00024334	67.0748185	7.09354803	634.24273
cg08589960	0.00024397	2314.29056	36.8907299	145183.921
cg13804316	0.00024539	1388289.52	723.481484	2663990505
cg02245020	0.00025006	7.73513429	2.58803436	23.1188208
cg16347018	0.00025061	2251.64353	36.1415914	140278.786
cg13686362	0.00025087	148594.265	253.151635	87221461
cg26508844	0.00025125	4979.90639	52.2222737	474882.956
cg22243039	0.00025325	0.0514852	0.01050943	0.25222348
cg23560546	0.00025365	26.4870284	4.5780747	153.244043
cg18096620	0.00025427	30076.4835	119.875293	7546132.64
cg23831021	0.00025434	1810.75876	32.5251024	100809.745
cg07717381	0.00025591	9800.13258	71.0737439	1351309.12
cg22761308	0.00025615	53.5928	6.3408586	452.965189
cg22030047	0.00025671	149.781899	10.21026	2197.2621
cg02874942	0.00025906	26.0385172	4.53017875	149.663935
cg21644826	0.00026006	1.1747E+10	46321.9822	2.9788E+15
cg03070194	0.00026024	125.252205	9.37413598	1673.55317
cg05417285	0.00026103	1880.48912	32.8578228	107622.448
cg11764177	0.00026146	251.920207	12.9453171	4902.4516
cg03578689	0.00026338	59.3430511	6.61945978	532.006814
cg23843362	0.00026402	1521426.56	726.010933	3188297424
cg20313392	0.00026563	30146.7788	117.98315	7703034.45
cg09168548	0.00026567	86586.1784	192.189089	39009323.1

cg15152115	0.00110956	315.557741	9.93411087	10023.7142
cg12995933	0.00111831	1.1756E+11	25850.7096	5.3464E+17
cg07234508	0.00111842	348.996669	10.3163082	11806.4208
cg25098208	0.00111924	56.8148435	5.00299682	645.198579
cg04437762	0.0011296	24.8067859	3.59024692	171.402313
cg10064585	0.0011303	897496857	3660.70108	2.2004E+14
cg19708133	0.00113175	0.02956935	0.00355001	0.24629422
cg16761581	0.00113723	9.18459252	2.41555184	34.922347
cg22122013	0.00113772	1746.20688	19.4685267	156623.997
cg10666406	0.00114467	87540376.3	1432.02737	5.3514E+12
cg15545878	0.0011488	71.4992	5.45106886	937.822605
cg02382037	0.00115304	26.8561431	3.69234348	195.337304
cg07545140	0.00115306	6204971785	7718.23607	4.9884E+15
cg27426082	0.00115357	46240.017	71.0898819	30076561
cg17132124	0.0011586	29.2921326	3.81855313	224.700037
cg16157530	0.00116409	22645003.8	824.279952	6.2211E+11
cg25994146	0.00116466	156.626797	7.415774	3308.0773
cg24395801	0.00116614	18.2259085	3.16021437	105.114306
cg22619719	0.00116811	406977.029	167.12467	991058361
cg17106175	0.00117928	43.7668707	4.4621996	429.281327
cg18953784	0.00118176	16.2345126	3.01262187	87.4850575
cg26302094	0.00118533	1.8498E+16	2716055.62	1.26E+26
cg14027234	0.00118601	56.5085128	4.93104695	647.572828
cg16358679	0.00118658	31.7868583	3.92719133	257.284221
cg24790471	0.00119057	1251.59027	16.7639714	93443.1451
cg09357934	0.00119472	73.6142723	5.46554711	991.494716
cg07517893	0.00119942	6.6943947	2.11867754	21.1523083
cg16232698	0.00120063	82.0045615	5.69706793	1180.38756
cg04900486	0.00120272	4488462.85	422.539992	4.7679E+10
cg21719787	0.00120768	1.7532E+14	416413.138	7.38E+22

cg03348149	0.00026756	34832.6964	125.774996	9646724.54
cg11206381	0.00026814	25.8726072	4.49767662	148.830576
cg07868721	0.0002684	94.9415727	8.20104755	1099.11596
cg00034416	0.00026919	3003067.46	983.658374	9168238107
cg25746764	0.0002697	3450.27177	43.0873681	276284.577
cg23924887	0.00027025	640.726962	19.7858593	20748.7091
cg07004443	0.00027164	213.58022	11.8995277	3833.47235
cg04329105	0.00027179	107.327362	8.65990257	1330.17232
cg20899053	0.00027191	4855.49005	50.32135	468504.593
cg02927327	0.00027582	26.4945137	4.53132369	154.912627
cg02528979	0.00027601	3279940.58	1010.02411	1.0651E+10
cg23687994	0.00027636	310663.545	340.500435	283441160
cg07569219	0.00027668	2266695.78	850.651021	6039973636
cg24210813	0.00027869	36.5090527	5.2458232	254.089945
cg10307580	0.0002828	1533.77144	29.2364811	80462.9943
cg26126052	0.00028548	2530.73132	36.7085421	174471.68
cg05997131	0.00028724	56943.471	153.237985	21160281.5
cg06947320	0.00028774	3722753.78	1045.2161	1.3259E+10
cg18301048	0.00028884	6564.7697	56.6743654	760417.888
cg27314482	0.00028984	109.958759	8.65641549	1396.75927
cg09161136	0.00029059	653965940	11149.294	3.8359E+13
cg06726474	0.00029139	63919114.7	3826.16553	1.0678E+12
cg02873371	0.00029275	3627.17571	42.9725417	306158.377
cg11275620	0.00029302	511548.742	416.037772	628986436
cg03770912	0.0002933	20.9265369	4.03517693	108.525588
cg03225210	0.00029353	5.99868909	2.27460406	15.8200152
cg09257526	0.00029355	40.9050775	5.48708067	304.939086
cg18025886	0.00029405	20.8206882	4.02460319	107.712745
cg07174453	0.00029499	725.310779	20.4933668	25670.5368
cg15243267	0.0002977	323227.451	334.411193	312417727

cg08735211	0.00121052	13.1463569	2.76219617	62.5685824
cg26202340	0.00121077	56.3966281	4.9053674	648.387655
cg04963082	0.00121631	3880.05545	25.9726348	579642.013
cg11350728	0.0012276	1907303.16	296.672815	1.2262E+10
cg19969694	0.00123139	16.571167	3.01852679	90.9727141
cg13904877	0.00123162	0.03062442	0.00369681	0.25369296
cg20929407	0.00123244	8821923545	8183.79858	9.5098E+15
cg10472567	0.00123291	68.7293858	5.28156684	894.380136
cg13631572	0.00123358	6.86990637	2.13425441	22.1133963
cg14413354	0.00124472	81.8907537	5.64571911	1187.81955
cg19928764	0.0012451	358888.225	152.222333	846135749
cg23500931	0.00124667	349287945	2269.55024	5.3756E+13
cg10506057	0.0012511	0.04678881	0.00728493	0.30050974
cg00841693	0.00125438	196.409402	7.94401304	4856.06618
cg17956609	0.00125466	85.3310158	5.72683349	1271.44997
cg15574263	0.00125674	1877.44828	19.2533449	183075.307
cg08081156	0.00126204	11.3023491	2.5882765	49.3545008
cg19700658	0.00126276	8872.32369	35.3298262	2228092.69
cg03770912	0.00126907	34.2858223	3.99531079	294.224322
cg27542552	0.00127118	33.2778175	3.94762075	280.526729
cg02348830	0.00127489	67227877.5	1162.43618	3.888E+12
cg02776498	0.00127516	57.2624605	4.87959718	671.979522
cg21791967	0.00127578	366532.956	150.926954	890141913
cg26987660	0.00127636	26.0105006	3.58180343	188.884218
cg05628616	0.00128176	47.2723836	4.52178786	494.202364
cg04414912	0.00128431	0.07841894	0.01664792	0.36938729
cg07907506	0.00128576	23.0354796	3.41118881	155.556714
cg25149391	0.00129401	244.713791	8.58026557	6979.36899
cg10625931	0.00129631	147.969485	7.0454025	3107.69588
cg03233656	0.0013014	61.7913217	5.00437712	762.96557

cg03697629	0.00029781	2236813.95	811.168071	6168064083
cg14043411	0.00029854	733012846	11501.2546	4.6717E+13
cg18676229	0.00029909	17581.3018	87.938442	3514983.49
cg01038207	0.00029927	489.304709	17.0502544	14041.9663
cg24229963	0.00030298	508538826	9616.47589	2.6893E+13
cg07212963	0.00030369	77877.7968	172.704675	35117470
cg06463010	0.00030425	133619495	5201.6895	3.4324E+12
cg05963821	0.00030621	26.1584328	4.44581655	153.911796
cg20495019	0.00030842	390580589	8406.59915	1.8147E+13
cg10134882	0.00030894	636.309901	19.0769218	21224.0892
cg05095290	0.00031096	506.055865	17.1548179	14928.3158
cg22652406	0.00031604	737.926415	20.292852	26833.8524
cg10763374	0.00031822	0.04241278	0.0075903	0.23699253
cg13817182	0.00031991	6064.07228	52.7846865	696659.867
cg19967582	0.00032039	7230402.28	1327.27871	3.9388E+10
cg10667044	0.00032104	4904.65268	47.8664376	502557.096
cg06526620	0.00032322	0.05710959	0.0119965	0.27187131
cg17380238	0.00032426	22.5366702	4.12408688	123.1549
cg19450531	0.00032676	26476.7036	102.395726	6846143.48
cg04665974	0.00032779	2185872.85	759.80433	6288513939
cg09701102	0.00032807	0.00207723	7.14E-05	0.06041783
cg05973813	0.00032824	9.8409E+14	6485816.27	1.49E+23
cg12947447	0.00032959	40799.5698	124.184226	13404318.3
cg20511194	0.00033392	209985.689	259.700576	169787800
cg24703125	0.00033393	283525677	6832.95619	1.1765E+13
cg24790471	0.00033461	1216.99536	25.0856412	59040.8555
cg10500406	0.00033471	723613.056	454.65497	1151677404
cg24542714	0.00033598	28.2244203	4.54714862	175.190646
cg22866937	0.00034329	45.6850928	5.63829115	370.17026
cg06998282	0.00034505	6.43917175	2.32208505	17.8559062

cg16408670	0.00132947	0.02767883	0.00309611	0.24744483
cg20641531	0.0013297	0.07130197	0.01421401	0.35767317
cg22957613	0.00133104	0.02639938	0.0028682	0.24298414
cg14779329	0.00133879	68.7617781	5.18364838	912.134038
cg00905524	0.00134134	257.800838	8.66196601	7672.76993
cg12457909	0.00134387	19.8487231	3.19508907	123.305422
cg23892568	0.00135644	24.7432097	3.47522	176.169113
cg21392341	0.00135743	14.1584562	2.79778086	71.6503157
cg13695646	0.00135753	55.9196032	4.76848786	655.763863
cg06263843	0.00136052	379.631032	10.0221579	14380.1087
cg05233094	0.0013726	12098940.4	556.059792	2.6325E+11
cg05299836	0.00137391	256.210871	8.57815004	7652.46706
cg25769935	0.00137471	52494.4283	67.4550366	40851879.2
cg02889982	0.00137874	49659.8761	65.904459	37419369.4
cg07185648	0.00137928	441.153081	10.5749433	18403.5068
cg02167201	0.00138051	459.933015	10.743827	19689.295
cg16052317	0.00138257	17.3581058	3.01932295	99.7918549
cg14560703	0.00138433	77.674922	5.39192671	1118.96801
cg10581650	0.00139038	41.8730324	4.24105737	413.423043
cg07232612	0.00139224	17.1199671	2.99993058	97.7000183
cg13382703	0.00139604	38756145.7	858.967069	1.7487E+12
cg19247841	0.00139962	28.5398986	3.65202062	223.034287
cg15656257	0.00140193	153.67799	6.99725372	3375.17053
cg18664712	0.00141421	1554945.94	245.210839	9860318179
cg11063170	0.00141536	0.03726147	0.00494131	0.28098144
cg05679521	0.00141878	1.8383E+15	773005.49	4.37E+24
cg06798491	0.00143	10615.7268	35.581817	3167169.79
cg23371050	0.00143053	3428.78438	23.0166555	510784.997
cg00557236	0.00143514	0.0023696	5.76E-05	0.097492
cg11641045	0.00144194	314.366174	9.14368006	10808.131

cg03712541	0.00034566	11.3002558	2.99429957	42.6462944
cg13039082	0.00034602	28.3913909	4.54142873	177.492839
cg03100449	0.00034624	172.138978	10.2589589	2888.38547
cg21225170	0.00034692	14.1826396	3.31706773	60.6400841
cg09279544	0.00034807	79.8589094	7.2420457	880.613803
cg19544919	0.00035009	4964.59576	46.7416027	527307.787
cg05984290	0.00035013	15970.1538	79.2371243	3218766.63
cg25823214	0.00035124	3.0719E+13	1234502.8	7.64E+20
cg05406954	0.00035399	18001008.7	1881.61479	1.7221E+11
cg02709432	0.00035404	12.7481225	3.15438224	51.5202711
cg22931309	0.00035691	66643.5791	149.799309	29648779.2
cg19559248	0.00035957	2526.92757	34.1620777	186913.776
cg21817720	0.00036065	5365.98928	47.9180067	600898.136
cg06887897	0.00036155	0.0849546	0.0219155	0.32932323
cg07195301	0.00036259	26.1681451	4.35021808	157.410917
cg04849025	0.00036365	6896064.16	1199.60114	3.9643E+10
cg06361403	0.00036375	18530.4415	83.4718753	4113688.12
cg22681784	0.00036405	103.738977	8.08305326	1331.39979
cg09807229	0.00036549	100175.885	178.048363	56362259.3
cg17987601	0.00036626	35290.2166	111.233413	11196270.6
cg02733093	0.00036694	4070530393	21046.1822	7.8728E+14
cg07483245	0.00036707	1776.09194	28.9612608	108921.451
cg10625931	0.00036713	116.696087	8.5090493	1600.41107
cg24633648	0.00036919	19292.6516	84.4978942	4404919.29
cg03196220	0.00036944	224.398262	11.4032159	4415.82271
cg15109933	0.00037174	2.6129E+10	47968.5704	1.4233E+16
cg19792802	0.000372	11.8234829	3.03395475	46.0767413
cg18187587	0.00037375	15803364	1710.75355	1.4599E+11
cg08872085	0.00037395	1666546.78	622.702028	4460204169
cg06335889	0.00037588	8435750.43	1285.70827	5.5348E+10

cg12281657	0.00144255	15.0737699	2.84055339	79.9909411
cg08473725	0.00144529	81.8290404	5.44404817	1229.96558
cg19147218	0.00144644	10.2521825	2.4481036	42.9341498
cg15103181	0.00145201	143.38669	6.74650036	3047.46783
cg17556421	0.00145515	0.04300077	0.0061966	0.29840031
cg23168192	0.00146356	27.3346329	3.56215764	209.7555
cg09745243	0.00146411	271102.247	121.973373	602561249
cg06830319	0.00146422	48.3215597	4.43287273	526.740394
cg07967717	0.00146834	18.4890511	3.06391463	111.571323
cg11270005	0.00147275	7069.30523	29.9811355	1666884.05
cg01025836	0.00148	11.7728684	2.5737025	53.8525452
cg05221455	0.00149472	16.4964846	2.92448103	93.0537761
cg27323088	0.00149537	5522741.7	380.982595	8.0058E+10
cg16150571	0.00149829	0.06235267	0.01124369	0.34578113
cg17121747	0.00150213	6.51301234	2.04790452	20.713529
cg21263269	0.00150846	0.00075962	8.99E-06	0.06418359
cg16142824	0.00151506	0.05862054	0.01015738	0.33831246
cg26222247	0.00151957	24.8069506	3.40862912	180.53733
cg10150530	0.00152258	8.33366142	2.24677115	30.9109864
cg23177883	0.00152428	6050.03187	27.769512	1318096.1
cg21241889	0.0015293	37.1757082	3.97287642	347.867171
cg26422458	0.00153157	37.5381967	3.98639661	353.481189
cg23425316	0.00153291	7.55049351	2.16201483	26.3688997
cg06301550	0.0015535	12.1333516	2.58586901	56.9318167
cg00886812	0.00155623	78645.9212	72.9460447	84791176.1
cg20370281	0.00155653	30.1314275	3.65434857	248.444532
cg15742840	0.00155867	0.02114894	0.00193965	0.23059767
cg22243039	0.00155927	0.04187294	0.00586305	0.29904994
cg12493107	0.00156513	180.895573	7.21618525	4534.69628
cg04992638	0.00156672	5.20774092	1.87257451	14.4830368

cg01030534	0.00037706	4.72548306	2.0075839	11.1229175
cg13320002	0.00037737	109207.495	182.331803	65409746.4
cg10005098	0.00037764	3.724E+10	55375.6778	2.5043E+16
cg20313628	0.00038187	65.922262	6.53721377	664.770157
cg25975979	0.00038323	117.380038	8.46073677	1628.47205
cg01370129	0.00038337	16703605.6	1723.88611	1.6185E+11
cg17922215	0.000384	5433.51995	47.1438093	626235.756
cg19965941	0.00038435	176113.347	223.906457	138521736
cg02448276	0.00038449	511.186523	16.3448153	15987.4344
cg21841239	0.00038461	127.598169	8.77688793	1855.01886
cg03756034	0.00038544	23.3853144	4.10313294	133.281796
cg06664874	0.00038776	4042.97739	41.1591002	397133.709
cg10147666	0.00038871	22.8413413	4.05571045	128.640069
cg07091842	0.00038881	4059.87654	41.1978329	400084.09
cg07294234	0.00038911	16924.3637	78.0199237	3671294.1
cg25355065	0.00038955	12618.5267	68.3846771	2328404.88
cg15897774	0.00039034	240052.802	255.224265	225783186
cg10368049	0.00039199	16.7224259	3.5240368	79.3520452
cg11063170	0.0003927	0.044864	0.00806392	0.24960313
cg07361448	0.00039457	3.33E-08	2.44E-12	0.00045522
cg20385284	0.00039544	0.03587801	0.00569368	0.22608091
cg23837756	0.00039577	4519046.85	940.589821	2.1712E+10
cg23969515	0.00039746	7189830.31	1154.2593	4.4785E+10
cg00690402	0.00039803	56.515957	6.05954979	527.110678
cg18202577	0.00039866	10850.494	63.355558	1858293.48
cg10856045	0.00040239	50.0062964	5.72720459	436.623075
cg14998896	0.00040304	21.0184568	3.88982839	113.571983
cg08681432	0.00040322	20284.1591	83.3826951	4934442.47
cg21219327	0.00040358	148589677	4411.32388	5.005E+12
cg26277456	0.00040372	162638.872	210.884227	125430920

cg27262412	0.00156835	17.084063	2.94106665	99.2378767
cg05700129	0.00157421	20.6477152	3.15863689	134.972192
cg02469457	0.00157433	529109.343	149.369389	1874257497
cg06080793	0.00157782	15.0318579	2.79881756	80.7329336
cg09350274	0.00157866	14.4598992	2.75766485	75.8209197
cg19065831	0.00158311	21.2091454	3.18785174	141.106892
cg21951729	0.00158528	48.1320536	4.34966741	532.614191
cg16838838	0.00158982	239.77167	7.99289986	7192.69035
cg13787135	0.00159253	27.7818905	3.5278196	218.784839
cg26570683	0.00159459	234.21392	7.91469544	6930.92497
cg00557360	0.00159614	13.0265319	2.64620365	64.1260294
cg11589885	0.00159736	0.00817811	0.00041353	0.16173133
cg14042912	0.00159853	3275.34002	21.493369	499123.812
cg04452432	0.00159905	23.5153433	3.30922071	167.100179
cg21870145	0.00160325	22.2391808	3.2384623	152.720988
cg08863777	0.00160703	0.06815847	0.01284659	0.36161935
cg01860897	0.00160824	10.6182258	2.4463781	46.0872007
cg08158952	0.00161101	10.591043	2.44343366	45.9067887
cg06459662	0.0016138	10450477.8	453.382256	2.4088E+11
cg01258201	0.00162277	21.4255	3.18624969	144.072843
cg20588892	0.0016233	1.3524E+13	92291.3397	1.98E+21
cg01790464	0.00162336	20.5829064	3.1380757	135.005041
cg22637507	0.00162824	114.558958	6.00072724	2187.0274
cg22415969	0.0016307	53.1291639	4.48677978	629.116692
cg00496961	0.00163321	59.2954136	4.67515874	752.048491
cg05031016	0.00163348	13.8282157	2.69735432	70.8915204
cg16714002	0.00163383	215.523496	7.61160315	6102.57479
cg06263193	0.00163503	16.9811451	2.91452255	98.9387745
cg14637144	0.0016359	54.8524154	4.53787856	663.038342
cg07485357	0.0016371	10.7024783	2.44775671	46.7951085

cg25062542	0.00040459	21031.5824	84.6211884	5227147.79
cg24448952	0.00040771	950578.203	460.850189	1960721599
cg15586420	0.00041174	730.952116	18.8308634	28373.1545
cg27387222	0.00041363	0.04356359	0.00765222	0.24800466
cg24461171	0.00041441	47.966764	5.59532019	411.202642
cg00981594	0.00041568	25271.3158	90.8034832	7033203.82
cg19901191	0.00042034	45327.3497	117.161991	17536136.2
cg03949306	0.00042177	23463.3095	87.3201123	6304697.47
cg04900427	0.00042332	11.6316371	2.97264219	45.5133761
cg19708133	0.00042626	0.04046547	0.00679544	0.24096379
cg12027420	0.00042799	23.4932743	4.0557134	136.088004
cg22971415	0.00043064	200625.655	224.099681	179610491
cg03404102	0.0004324	8482.13647	55.0498305	1306936.62
cg19118262	0.00043365	45375.8288	115.588631	17812875
cg08160980	0.00043396	7.78419897	2.48182228	24.4150252
cg05125578	0.00043798	16.5931432	3.46660535	79.4242133
cg05094271	0.00043951	829102.909	415.459226	1654582664
cg09223516	0.00044012	10679.2454	60.5457211	1883639.03
cg21095811	0.00044077	3257117.68	759.708375	1.3964E+10
cg06035374	0.00044146	39614613.7	2291.15188	6.8495E+11
cg07554759	0.00044222	62.7051142	6.23308285	630.816474
cg27423729	0.00044348	241773135	5079.65647	1.1508E+13
cg05417332	0.00044374	4212.83928	40.0068267	443624.657
cg13169065	0.00044381	867.262569	19.8930009	37809.4973
cg14921691	0.00044507	90.670306	7.32808705	1121.86227
cg25544073	0.00044997	89903.1885	153.724982	52578203
cg01354296	0.00045338	264.548714	11.7109166	5976.1353
cg13581941	0.00045635	530307.132	333.857686	842351895
cg13109300	0.00045897	402110.508	294.588616	548876814
cg27510007	0.0004629	21139.618	80.1724308	5574028.95
cg05883874	0.00164426	16.6784318	2.89213885	96.1814429
cg03457528	0.00164564	2641.16453	19.5505096	356806.56
cg26532627	0.00164682	19.2951885	3.05484613	121.873339
cg07528363	0.00165057	81.1684549	5.24982749	1254.95897
cg16754665	0.00165169	255.477126	8.08841306	8069.39032
cg05021846	0.0016527	26.3760182	3.43492571	202.535483
cg02324920	0.0016539	160.218497	6.78066864	3785.75744
cg09171882	0.00167297	35.7484232	3.84249091	332.583678
cg01814495	0.00167332	2672276548	3531.54233	2.0221E+15
cg15074033	0.00167629	24.4460748	3.32914289	179.508838
cg22330492	0.00168436	67.227425	4.86551751	928.889201
cg00487737	0.00170637	74.6418781	5.04422434	1104.51272
cg17446896	0.00171034	13.8069902	2.67695877	71.2125196
cg09668344	0.00171071	17.4656436	2.92359596	104.340241
cg18425877	0.00171096	2418.32474	18.5804991	314754.439
cg16552437	0.00172512	4253.28306	22.8706098	790989.701
cg16475951	0.00173803	280733.651	109.266731	721275199
cg21400015	0.00174069	32.8140579	3.69062646	291.755995
cg16368008	0.00174297	0.06915486	0.01298776	0.36822333
cg02718824	0.00174557	35631893.6	666.213019	1.9057E+12
cg14806045	0.00175206	22775790.4	561.481039	9.2387E+11
cg10368049	0.001756	17.7048743	2.92564204	107.143174
cg20385284	0.00176195	0.0317335	0.00365167	0.27576865
cg04604505	0.00176301	2.336E+11	17538.4641	3.1114E+18
cg08247376	0.00177068	6.86637458	2.05185509	22.9777922
cg03627290	0.0017707	27.4558986	3.44105396	219.068452
cg05011526	0.00177132	500.895299	10.1648471	24682.7224
cg27387222	0.00177139	0.04319848	0.00602493	0.30973106
cg17658717	0.00177237	70.1013705	4.88041493	1006.92302
cg07375367	0.00178115	72552.0492	64.8047774	81225490.6

cg10059484	0.00046472	440.553482	14.5718904	13319.2994
cg07030951	0.00046689	3387335.17	745.127258	1.5399E+10
cg18859544	0.00046782	3.6604E+10	44217.096	3.0302E+16
cg21880079	0.00046844	218104.452	222.594519	213704957
cg02622866	0.00046889	7338.65043	50.0758018	1075485.33
cg10812348	0.00047004	17300.8229	72.9363726	4103829.97
cg02167201	0.00047247	291.662485	12.1046653	7027.6214
cg13880726	0.00047346	2.72E-05	7.50E-08	0.00987905
cg17925360	0.00047424	6.05E+20	1340025212	2.73E+32
cg24287218	0.00047594	21.8299969	3.87166045	123.086404
cg00129981	0.00047883	0.00657836	0.00039228	0.11031477
cg27542552	0.00047979	38.9749019	4.98732631	304.580628
cg06604199	0.00048003	31.521226	4.54356147	218.680367
cg10330832	0.00048117	17397045.6	1497.81437	2.0207E+11
cg20237547	0.00048138	29.6824682	4.42352248	199.173605
cg01493678	0.00048195	28.8072581	4.36508996	190.11249
cg25165701	0.00048218	24.4287851	4.06039775	146.972189
cg09954820	0.00048297	19.0770728	3.64241658	99.9157284
cg14555988	0.00048367	23514472.7	1702.96779	3.2469E+11
cg16157530	0.00048673	4402617.31	813.682199	2.3821E+10
cg12269002	0.00048728	15.3804331	3.31074066	71.4516011
cg07310282	0.0004878	296765.901	249.355057	353191152
cg11828766	0.00049016	708382.586	363.991529	1378619689
cg25415989	0.0004929	331.087042	12.6636116	8656.19014
cg13442432	0.00049617	10.6143567	2.80911576	40.1067727
cg06611606	0.00049711	270273889	4853.95029	1.5049E+13
cg06830319	0.00049796	34.2893295	4.68818828	250.791574
cg01773760	0.00049948	320.623383	12.4456923	8259.83412
cg13183282	0.00050111	34685594.1	1966.4271	6.1182E+11
cg11815708	0.00050152	52.5550561	5.64331765	489.434425

cg13817182	0.00178434	8650.17125	29.3053411	2553304.61
cg20020464	0.00178719	148686.913	84.4725664	261715714
cg12841566	0.0017954	501042264	1731.48271	1.4499E+14
cg27204544	0.00179873	32012.4146	47.4847033	21581575
cg22031999	0.00179945	2900.56999	19.4269401	433074.187
cg05356496	0.00180256	27.339597	3.42364856	218.320763
cg12985477	0.00181357	2234.94878	17.5680606	284322.566
cg13316191	0.00181664	226.04008	7.49333233	6818.61095
cg15951557	0.00181723	17.7841185	2.91348681	108.55545
cg01931792	0.00181811	61.2120183	4.61100614	812.601647
cg20308679	0.00181852	10.5260985	2.39745655	46.2151232
cg17077566	0.00181882	0.05202528	0.0081155	0.33351354
cg16773799	0.00182011	18368.6721	38.3524582	8797561.59
cg18328334	0.00182078	39.2748966	3.90896816	394.609892
cg05403387	0.00182193	3637.7171	21.0075769	629914.9
cg23876285	0.00182195	30.359622	3.55202159	259.487906
cg16259747	0.00182361	1166040.84	178.915432	7599407271
cg23283234	0.00182736	3619994.05	271.976576	4.8182E+10
cg18449964	0.00183187	40.0067904	3.93062821	407.197831
cg09667023	0.00183451	3.7421E+14	254692.523	5.50E+23
cg08438366	0.00184285	27.1822062	3.40151374	217.218683
cg09648722	0.0018433	8529.37347	28.6475143	2539494.73
cg09142117	0.00185526	135291968	1025.4376	1.785E+13
cg03157027	0.00185655	21.4124929	3.10938442	147.455184
cg22657536	0.00185742	63.9207732	4.66102622	876.602073
cg26151000	0.00186244	529.391257	10.1847812	27517.047
cg02621694	0.00186362	39.8616385	3.91052822	406.32624
cg10500147	0.00186677	11.7251295	2.48591218	55.3031049
cg11335133	0.00186953	9.42545717	2.29258614	38.7506674
cg02276831	0.00187107	100.545458	5.50090323	1837.76894

cg20308679	0.000506	9.14056104	2.62642719	31.8112212
cg22225546	0.00050629	2429.70532	30.0166079	196673.387
cg22336806	0.00050924	7.8963778	2.46255331	25.3203787
cg09205595	0.00051265	70.7861491	6.40118702	782.773396
cg10581650	0.00051412	29.3232173	4.35782133	197.312144
cg26920587	0.00051522	8297.18798	50.9480695	1351245.08
cg22517356	0.00051582	38.1781309	4.88623662	298.301084
cg18714412	0.00051995	189.147264	9.79241015	3653.51195
cg13436879	0.00052089	50903782.4	2256.6344	1.1483E+12
cg25768103	0.00052171	11815783.5	1193.90911	1.1694E+11
cg05794119	0.000529	47099.0192	107.205679	20692165.2
cg08724866	0.00053013	276696.246	231.100189	331288404
cg15007132	0.00053104	7779917.99	983.240591	6.1559E+10
cg27020690	0.00053146	2920.89623	31.9822124	266761.871
cg15253473	0.00053392	8253.12856	50.1193938	1359037.41
cg00352195	0.00053489	3244835037	13414.2498	7.8491E+14
cg09697857	0.00054211	15174.1412	64.8638522	3549813.25
cg13538257	0.00054218	832.060122	18.4295716	37565.9328
cg07972191	0.00054282	101.495571	7.40346642	1391.42267
cg21913319	0.00054438	9.47531959	2.64883285	33.8948081
cg15453599	0.00055359	1020828.57	396.812883	2626152051
cg15235373	0.00055437	903824.142	376.138645	2171800454
cg00254079	0.00055528	164296185	3562.50535	7.577E+12
cg22345522	0.00055741	834065.189	362.101737	1921185869
cg01818648	0.0005577	9363.51816	52.0178722	1685487.48
cg20991612	0.00055939	38.0611192	4.81671418	300.754568
cg24395801	0.00056059	15.0994903	3.22988141	70.5891574
cg04624228	0.0005634	19.7611711	3.6254796	107.710958
cg08693600	0.00056422	3726795563	13522.4681	1.0271E+15
cg03460682	0.00056493	36.0731757	4.69899478	276.926038

cg21201393	0.00187133	18.7997055	2.95900306	119.441893
cg02486590	0.00187389	203499027	1179.21602	3.5118E+13
cg27162464	0.00187591	224.170429	7.39321958	6797.09029
cg09700470	0.00188085	34.1526586	3.68596464	316.444731
cg14425609	0.00188201	6.91E+23	641152784	7.44E+38
cg10856045	0.00188277	51.3950558	4.28573926	616.335153
cg00929411	0.00188456	561.701389	10.3640417	30442.6073
cg12837296	0.00188779	1450601.9	188.422808	1.1168E+10
cg19904411	0.00188813	7727.67142	27.2651866	2190225.45
cg02096793	0.00189034	15.5776242	2.75569433	88.0585247
cg19094597	0.00189129	8778.7595	28.5537177	2699004.7
cg14542839	0.0018922	107.035116	5.61192768	2041.45823
cg17202086	0.00189533	10.4713911	2.37897363	46.0913183
cg16300030	0.00189839	209.988287	7.18923644	6133.48593
cg06222206	0.00190461	0.03030316	0.00333329	0.27548833
cg05951178	0.00190736	0.05913601	0.0099182	0.35259082
cg17387989	0.00191128	26.2724102	3.33496426	206.970595
cg18316621	0.00192441	29.50121	3.47564182	250.40595
cg21158815	0.00192672	16.1840327	2.78590854	94.017054
cg17246382	0.00192837	21.8724297	3.11199076	153.728985
cg17034360	0.00193011	34514.524	46.7292085	25492671.7
cg10794285	0.00193367	8.38487902	2.18606777	32.1610323
cg21152671	0.00193396	23.265532	3.1817556	170.121482
cg18978493	0.00195103	16.0910496	2.77416029	93.3334237
cg24910161	0.0019553	75.3046496	4.88692037	1160.40161
cg03269218	0.00195885	21.6756022	3.09262049	151.920267
cg15844609	0.00197964	4205.86967	21.2667489	831783.916
cg03751272	0.00198235	220.986243	7.22318697	6760.85496
cg02132284	0.00198312	14.5440369	2.66598712	79.3435971
cg25607670	0.00198564	27.6557822	3.3726663	226.776746

cg08799865	0.00056559	1.334E+14	1244460.81	1.43E+22	cg01597066	0.00198843	6.83423389	2.0210961	23.1096151
cg19677989	0.00056786	1579.76639	23.9673442	104127.592	cg17799946	0.00199257	22.1716259	3.10840518	158.145726
cg19568591	0.00056819	11.8928084	2.90914138	48.6187754	cg18309286	0.00199333	7.53822304	2.0943254	27.1327496
cg12615761	0.00056851	6.63115006	2.26113288	19.4469558	cg24174307	0.00200099	1489.31117	14.4690342	153296.187
cg06440553	0.00057323	1502.87986	23.3850918	96584.9478	cg04809274	0.00200235	38.1222599	3.7862637	383.836629
cg08863777	0.00057338	0.0741915	0.01688342	0.32602278	cg23510258	0.00200599	15.8144027	2.74372698	91.1516832
cg18805367	0.00057472	6.9615E+14	2477445.44	1.96E+23	cg16285902	0.00200826	1981432647	2500.81515	1.5699E+15
cg07797438	0.00057538	91735976.5	2689.87987	3.1286E+12	cg13997864	0.00200832	14.2723962	2.64223231	77.0943918
cg17842066	0.00057562	699882.873	329.259676	1487689114	cg15929495	0.00201298	2.0468E+10	5851.36565	7.1595E+16
cg10717074	0.00057593	963.305513	19.2786939	48133.837	cg10631694	0.00201717	1.5714E+13	66027.1944	3.74E+21
cg23963591	0.00057653	42.0753342	5.00468884	353.735029	cg04346127	0.00201729	1621888.92	185.389653	1.4189E+10
cg18357908	0.00057956	1111486.18	400.141316	3087413062	cg22970435	0.00202201	34.8279593	3.65542398	331.832028
cg05386508	0.0005807	64841.1901	117.663031	35732378.4	cg01476568	0.00202508	5.89387501	1.91069719	18.180674
cg27179932	0.00058175	15832.8613	64.0940965	3911116.81	cg24258806	0.00202856	383115795	1355.07539	1.0832E+14
cg04776910	0.00058218	354114.963	243.93905	514052207	cg11832722	0.00202928	14.9626526	2.68362906	83.4247091
cg08091398	0.00058572	1.85E-07	2.69E-11	0.00127544	cg08048963	0.00203154	2711002.28	222.229643	3.3072E+10
cg26302094	0.00058579	2.2918E+16	10807542.5	4.86E+25	cg04608177	0.00203211	13.6984465	2.59798966	72.2279381
cg06055845	0.00058589	44.4536502	5.11060499	386.671837	cg25129124	0.00203289	22.9116063	3.13394836	167.501709
cg17077566	0.00058771	0.05293502	0.00990831	0.28280462	cg14273450	0.00203569	9.13763516	2.24074005	37.2628571
cg17929951	0.00058803	14.3042091	3.13740087	65.2165302	cg17733447	0.00203653	17.3286948	2.82953436	106.124764
cg15837119	0.00058866	54016.9512	108.048403	27004851	cg07036561	0.00204021	71127.7425	58.7157924	86163458.6
cg05700129	0.00058866	20.9251451	3.69406793	118.531036	cg06275813	0.00204249	83.3529097	5.01323591	1385.87285
cg09847789	0.00059003	298862.124	225.058161	396868831	cg27614319	0.00205184	17.0859436	2.81134658	103.83973
cg06834274	0.00059026	138.447486	8.3151518	2305.1541	cg15372689	0.00205391	14.9812293	2.67947484	83.761649
cg13647118	0.00059066	378873.753	249.048748	576374393	cg18805367	0.00205649	2.5202E+19	11564009	5.49E+31
cg00898147	0.00059289	49.4619836	5.33948872	458.187656	cg22935921	0.00206255	18.8128371	2.90894582	121.667044
cg17900356	0.00059468	25.4423559	4.01179372	161.352632	cg11412713	0.00209066	0.02062274	0.00174039	0.24436926
cg21465517	0.00059471	2.4705E+11	77687.3965	7.8561E+17	cg26070636	0.0020919	2251046679	2482.62827	2.0411E+15
cg19844724	0.00060171	24321.1437	75.9219795	7791130.27	cg17892169	0.00209285	41.1699814	3.85519986	439.657457
cg26964592	0.00060182	0.01734346	0.00171072	0.17582983	cg08464498	0.00210605	4005.8392	20.2430827	792702.768

cg06809635	0.00060328	0.00026423	2.39E-06	0.02927154
cg09684870	0.00060503	3482.92557	32.9327714	368349.519
cg01411912	0.0006105	27.8142539	4.15184744	186.334573
cg03007010	0.00061217	2395960.99	537.093244	1.0688E+10
cg05280555	0.0006124	822.905011	17.6827804	38295.5984
cg23054032	0.00061521	43468.6316	96.3452778	19611982.8
cg25004679	0.00061647	99868.2998	137.365486	72606864.9
cg25145687	0.00062067	0.05218768	0.0096192	0.28313711
cg13908523	0.00062266	2.876E+14	1500954.21	5.51E+22
cg09648722	0.0006252	2760.5848	29.4615095	258670.671
cg17876200	0.0006255	649878.386	303.294137	1392515928
cg06594008	0.00062789	26.812847	4.07006613	176.638104
cg10959198	0.00062845	9.4243681	2.60471574	34.0991966
cg23336454	0.00062875	25123.4511	75.4451836	8366177.49
cg25794571	0.00063484	766445.079	322.458922	1821745406
cg04575016	0.0006358	3305011.62	600.599544	1.8187E+10
cg09413557	0.00063699	3971.00384	34.1668783	461525.087
cg12937378	0.00063819	180197.854	173.455834	187201927
cg02671213	0.00063889	54.5881502	5.49535202	542.252094
cg05679521	0.00064281	5.727E+14	1917224.16	1.71E+23
cg27337492	0.00064593	267178.919	203.73557	350378555
cg17937683	0.00064807	1024680.97	360.202878	2914943671
cg14113931	0.00064882	0.07286656	0.01617369	0.32828234
cg24502391	0.00065044	6996.94695	43.1272067	1135182.88
cg15746396	0.00065221	6686.06422	42.255386	1057935.07
cg03069638	0.00065376	212188.397	183.455476	245421487
cg07844977	0.00065445	7.49553389	2.35339736	23.8731586
cg08141342	0.00065458	121.968476	7.69850927	1932.36232
cg18316621	0.00065502	20.6225966	3.61741753	117.567709
cg07233502	0.00065573	1564052.23	427.876304	5717211623

cg27528222	0.00210737	29.0829079	3.39339183	249.253719
cg02423574	0.00211312	32780.3455	43.2861836	24824342.5
cg17272843	0.00211539	42.0311788	3.87467782	455.939842
cg05247767	0.00211987	1182069.43	158.263221	8828887318
cg22336806	0.0021255	8.15410998	2.13762352	31.1044059
cg02007844	0.00212646	19.8504542	2.94967985	133.587559
cg00821731	0.00213179	41.0429452	3.8345936	439.296449
cg01916115	0.00213297	15.9295619	2.72240881	93.2082433
cg11248254	0.00214127	253.375217	7.39765482	8678.29091
cg18933494	0.0021425	101.681527	5.3168885	1944.58336
cg05412137	0.00214614	12.3657842	2.4816736	61.6167331
cg20984053	0.00214647	93.5147703	5.15567577	1696.19128
cg06096427	0.002149	501314438	1391.9516	1.8055E+14
cg05082085	0.00215039	1182409015	1896.31736	7.3727E+14
cg03434479	0.00215402	2.2059E+10	5444.58187	8.9374E+16
cg01420215	0.00215479	176924.575	78.5800845	398349090
cg01249180	0.00215713	2349272.2	199.766472	2.7628E+10
cg04550737	0.00215852	7.55634037	2.07548726	27.510783
cg21290280	0.0021644	2.1E+10	5310.73127	8.3039E+16
cg26045220	0.00216547	29.7431404	3.40158048	260.071578
cg17942851	0.00217245	20.8062826	2.98823141	144.868766
cg09261514	0.00218408	7.656E+10	8351.24438	7.0186E+17
cg07067280	0.00218856	5.4657E+10	7372.93966	4.0519E+17
cg09205595	0.00219162	141.670524	5.95238344	3371.84886
cg03003256	0.00219263	76.4373672	4.76581751	1225.9536
cg17838765	0.00219829	24.8930962	3.1803322	194.843243
cg26796245	0.00219947	12.7849015	2.50197059	65.3299871
cg18128969	0.00220128	52.8954283	4.17004541	670.958243
cg25239996	0.00220702	13.9623763	2.58110793	75.5287874
cg05766064	0.00220756	13.0705196	2.52045344	67.7808528

cg02501882	0.00065627	406.015881	12.8236178	12855.1005
cg26534477	0.0006566	0.04908011	0.00866633	0.27795598
cg15551525	0.00065783	23655.4735	72.0115745	7770715.05
cg09254823	0.00065813	5285.36693	38.1010476	733184.658
cg04387237	0.00066134	385173.774	234.743318	632004512
cg13901143	0.00066163	9949.51939	49.7297469	1990618.14
cg13770691	0.00066473	795.22156	16.9942965	37211.1507
cg27528222	0.00066532	24.3811217	3.87517492	153.396712
cg09209002	0.00066534	24438.6409	72.6292189	8223235.49
cg26657230	0.00066593	12011.118	53.7158646	2685742.06
cg07696485	0.00066704	24.370207	3.87296601	153.346812
cg03751272	0.00066818	139.139137	8.10356365	2389.03528
cg18357291	0.00067272	9733246.16	912.753715	1.0379E+11
cg12619880	0.0006751	50.9578638	5.2835526	491.469298
cg27637948	0.00067575	101.263246	7.06525686	1451.36196
cg20787196	0.0006808	218.41232	9.76482367	4885.28447
cg00841693	0.00068096	199.285128	9.39290952	4228.14276
cg03911494	0.00068141	2.1697E+14	1160447.59	4.06E+22
cg12104022	0.00068613	0.01282938	0.00103771	0.15861156
cg21998208	0.00068678	19.5464986	3.51301318	108.757237
cg22608128	0.00069038	9.95831857	2.64026693	37.5598799
cg02015582	0.00069051	594.509866	14.8531816	23795.7086
cg04416750	0.00069349	754.965565	16.4086477	34736.1351
cg19866195	0.00069551	21.6682853	3.66276707	128.185761
cg03434479	0.00069827	1.1647E+10	17650.6989	7.6848E+15
cg17733447	0.00069836	18.7373495	3.44291889	101.974016
cg11475922	0.0007002	689502.898	290.020318	1639244624
cg17892539	0.00070034	975409.226	335.667031	2834425400
cg25634545	0.00070065	50523.9829	96.2896416	26510357.8
cg13459361	0.00070179	2619.92992	27.6258595	248464.046

cg07051728	0.00220767	1483.76079	13.8223615	159274.238
cg06892501	0.00220959	0.06192767	0.01042804	0.36776183
cg08872085	0.00221263	3357036.09	221.893891	5.0789E+10
cg03976754	0.00221416	46905.7565	47.7694286	46057699.6
cg06594008	0.0022161	28.2181281	3.32151685	239.72865
cg18875012	0.00222562	89.1610762	5.01639008	1584.74468
cg17126533	0.00222591	1.1509E+11	9384.21174	1.4114E+18
cg23621729	0.00223225	15.2516948	2.65924907	87.4736396
cg06285333	0.00223472	2369.83691	16.260725	345379.863
cg00863716	0.00223634	32.0821978	3.47143516	296.496223
cg25789861	0.00223733	10.7642806	2.34579152	49.3947295
cg08279757	0.00224049	40795.1542	45.0751464	36921557.4
cg06059849	0.00224309	20.6962152	2.96446159	144.489416
cg02029665	0.0022461	43.5888888	3.87102362	490.823982
cg00713366	0.00225304	7798851.61	295.062642	2.0613E+11
cg18701685	0.00225356	8.9726311	2.19526169	36.6735817
cg02978959	0.00225694	168003.462	74.4801358	378962295
cg09395021	0.00226093	81512.9866	57.407121	115741164
cg23656386	0.00226626	128.974612	5.69597256	2920.38812
cg16098170	0.00226976	3365.99062	18.2968087	619227.814
cg27043726	0.0022714	12.13167	2.44282616	60.2488296
cg02532824	0.002273	29.9879229	3.37656518	266.328495
cg02168442	0.002274	39.4577588	3.72458083	418.010723
cg04567307	0.00227741	19.9712588	2.91841264	136.667163
cg26416971	0.00228405	12.1891765	2.44481727	60.7718317
cg24680586	0.00228583	7.19996935	2.02517141	25.5976153
cg08067617	0.00229259	24.8417841	3.15109649	195.841111
cg21950518	0.00229313	203.837792	6.68361801	6216.66971
cg18233810	0.00229654	15797792.3	372.462739	6.7005E+11
cg00255368	0.00230225	39.016047	3.69900825	411.529746

cg19928764	0.00070237	287373.61	200.139942	412629236
cg17759086	0.00070307	61.6343313	5.68227664	668.533237
cg02524205	0.00070566	11.839784	2.83333011	49.475522
cg07381000	0.00071001	74.4512182	6.14114621	902.597611
cg18719814	0.000711	27049.1277	73.4687471	9958728.57
cg07730673	0.00071197	2361.1476	26.3023637	211958.821
cg26985469	0.00071508	209712.242	173.449259	253556717
cg03514404	0.00071521	4.86148547	1.94520334	12.1499077
cg01840244	0.00071572	66632.6762	107.01688	41487974.2
cg14042879	0.00071782	2574.05498	27.1935447	243651.907
cg24503449	0.00071911	33.1861821	4.36082495	252.549161
cg04117530	0.00071993	10.1381286	2.64822622	38.8115073
cg10305451	0.00072039	7.71153584	2.36032576	25.1947363
cg08410301	0.00072271	1.4309E+10	18538.7999	1.1044E+16
cg25444017	0.00072332	140.537105	7.99032972	2471.82265
cg10334703	0.00072337	26.0628944	3.93594693	172.582221
cg05652493	0.00072458	7308719.57	765.281567	6.9801E+10
cg01085803	0.00072628	26.3941643	3.95444425	176.169359
cg00405699	0.00072646	18.348537	3.39425423	99.1878585
cg04881464	0.00072685	3799.2447	31.8764686	452818.675
cg18940113	0.00072694	105.548525	7.07679865	1574.22751
cg22607959	0.00072702	104066.638	128.001067	84607616.2
cg10589018	0.0007291	1.20E-06	4.43E-10	0.00327169
cg17950435	0.00072926	87549276.1	2160.24605	3.5481E+12
cg16575322	0.00073046	14.2761715	3.05257502	66.7662779
cg24938064	0.000732	3928.6869	32.2391303	478753.012
cg17394304	0.00073255	40.9286509	4.74740335	352.856992
cg25120284	0.0007328	26.5206224	3.95691078	177.750636
cg12072333	0.00073508	2873049.9	511.757255	1.613E+10
cg12810402	0.00073714	7371.1184	41.8592961	1298000.48

cg24693053	0.00231049	14.0134576	2.56486852	76.5641557
cg18853219	0.00231334	89987.6421	58.5017172	138419454
cg11255208	0.00231869	169.493971	6.23472438	4607.77487
cg25542733	0.00232348	7543513.46	282.706551	2.0129E+11
cg12827530	0.00232597	10.8109512	2.33573794	50.0384324
cg26440289	0.0023315	4.94086394	1.76659243	13.8187712
cg24684576	0.00233348	141.960237	5.84113455	3450.13607
cg03756034	0.00233426	23.7667318	3.09046748	182.774142
cg03671191	0.00233699	18.6499402	2.8342094	122.72215
cg12766383	0.00233765	2.4198E+11	11299.4812	5.1821E+18
cg22638491	0.00234459	17.3566114	2.76097461	109.110732
cg24580593	0.0023455	2312195365	2147.57231	2.4894E+15
cg10230885	0.00234775	60945.5702	50.3856076	73718720.5
cg02534163	0.00235528	9.75155395	2.24736741	42.3129766
cg07444414	0.00235589	85.8907424	4.87079847	1514.58117
cg00220769	0.00236169	548101.58	109.523753	2742924097
cg03469057	0.00236204	63549467	592.980167	6.8106E+12
cg10717074	0.00236448	5113.59438	20.7849034	1258069.23
cg03025013	0.00236528	289.778274	7.49451554	11204.3865
cg19151808	0.0023742	43.6190857	3.8208824	497.954253
cg05950711	0.00237583	49.3148578	3.99038023	609.454503
cg05341097	0.00237652	673.406447	10.0924658	44932.1554
cg23666930	0.00237796	16099.2325	31.1309311	8325651.63
cg24542751	0.00237817	5.85619749	1.87265806	18.3135671
cg11540838	0.00237856	5.4094E+15	383862.039	7.62E+25
cg24888944	0.00237938	52010221.9	547.654192	4.9394E+12
cg07898632	0.00238096	11.8258089	2.4027658	58.2036574
cg18703238	0.00238224	14.5773319	2.58767688	82.1194513
cg07365404	0.00238555	1.672E+10	4232.73594	6.6049E+16
cg05435286	0.00239042	33.8050646	3.48494258	327.920005

cg13437538	0.00073841	197.09365	9.1632514	4239.31477	cg22126440	0.00239169	29.3334898	3.31356235	259.676303
cg27614319	0.00073991	18.0997197	3.36637671	97.3152684	cg22325715	0.00240309	0.01702307	0.00122679	0.23621314
cg05799276	0.00074274	1.1408E+14	775600.476	1.68E+22	cg08141342	0.00240595	272.109902	7.28415527	10165.0495
cg02922601	0.000743	786.74926	16.3395358	37882.0064	cg01574788	0.00241117	23.6048231	3.06282184	181.919713
cg02282382	0.00074334	2704.31541	27.4043939	266866.762	cg17394304	0.00241333	44.6167324	3.83639092	518.886852
cg12309516	0.00074425	549891.005	253.798091	1191419982	cg01462607	0.00242142	20.2596585	2.89912707	141.578397
cg01870903	0.00074704	11771.3172	50.6368452	2736424.61	cg17980625	0.00242434	11.4273608	2.36703011	55.1681088
cg21109744	0.00074718	13.6452917	2.98682628	62.3384051	cg13094036	0.00243684	0.01813745	0.00135685	0.24244939
cg03549127	0.00074823	24000.9858	68.1841953	8448399.48	cg01431908	0.00244626	13.2791016	2.49242674	70.7481331
cg01852715	0.00074889	2946.76559	28.3280035	306531.572	cg02404489	0.00244633	2908200088	2197.1247	3.8494E+15
cg19240052	0.00075007	986.332938	17.9072795	54327.2172	cg08296969	0.00245166	37316354.9	470.732909	2.9582E+12
cg05894557	0.00075133	0.04528189	0.00748602	0.27390375	cg09004287	0.00245871	23.6517733	3.0528102	183.243092
cg15658376	0.00075221	4.1498E+11	72558.9674	2.37E+18	cg10197666	0.00246318	18858061.4	368.082587	9.6616E+11
cg04452432	0.00075237	17.5349206	3.31440316	92.7688714	cg16368146	0.00246478	25.7381444	3.1436035	210.730163
cg22248683	0.00075394	7904194.4	767.60054	8.1392E+10	cg00516051	0.00247191	1135824.06	136.213979	9471100623
cg23543123	0.00075405	50.1590804	5.14264195	489.229734	cg03048083	0.00248041	4.56023973	1.70654142	12.1859253
cg16119852	0.00075446	279272.103	189.543316	411478014	cg20184247	0.00248482	29.7679523	3.30318242	268.265833
cg07218275	0.0007562	1612.61179	21.9352879	118554.031	cg21087137	0.00248611	8.87124395	2.15660149	36.4921241
cg06199563	0.00075828	14786.4576	55.3314151	3951450.15	cg14847134	0.00249055	106.251006	5.16659644	2185.05092
cg00173504	0.00075858	18.2417109	3.36582822	98.8642301	cg08038761	0.00249415	377466.328	91.6983214	1553799748
cg16150571	0.00075906	0.08033495	0.01851386	0.34858769	cg10129408	0.00249516	15.8535756	2.64400856	95.0586402
cg23212579	0.00076001	0.00048013	5.62E-06	0.04103895	cg16352928	0.00249635	27.1376947	3.19415282	230.563319
cg08704611	0.00076239	6700375.1	710.391645	6.3198E+10	cg04352704	0.00250273	3858857.03	207.080881	7.1908E+10
cg02783970	0.0007629	2.2588E+11	55277.7459	9.2304E+17	cg24903183	0.00250705	6.89459838	1.9713756	24.1128513
cg23281832	0.00076292	58598.4278	98.0734879	35012273.1	cg24963041	0.00250834	17916.9355	31.2643536	10267814.3
cg03075736	0.00076313	371814.048	212.158288	651615771	cg22344830	0.00251247	14.2633304	2.54444273	79.9556583
cg21100807	0.00076336	35437373.3	1423.02662	8.8249E+11	cg05990544	0.00251621	11.046842	2.32537279	52.4787763
cg20773694	0.00076667	1311012.74	358.015141	4800786931	cg04006913	0.00251907	538661784	1165.98132	2.4885E+14
cg23687909	0.00076716	20.7913527	3.54921792	121.795944	cg16993579	0.00252263	5916.03633	21.1105784	1657912.21
cg26831148	0.00076721	1847.84061	23.1002158	147813.118	cg25294502	0.00253001	197.059708	6.38678581	6080.13633

cg20368283	0.00076783	65.4165537	5.72610117	747.336692
cg22935653	0.00076809	822.20421	16.4683798	41049.5611
cg18642271	0.00077038	142666.475	141.439488	143904105
cg06709324	0.000772	2774028298	8690.85218	8.8544E+14
cg11528307	0.00077354	13927.7684	53.4769958	3627405.21
cg02317785	0.00077611	3.1634E+13	424585.938	2.36E+21
cg20607710	0.00077969	5.3582E+10	29537.0064	9.7202E+16
cg06127885	0.00078161	69.3939569	5.84775662	823.481818
cg18309286	0.00078174	7.16628425	2.27122507	22.6114227
cg02767164	0.00078378	47962.5232	88.956023	25860009.9
cg23227500	0.00078475	825931.595	290.751526	2346206087
cg02417033	0.00078768	17.3766358	3.28124044	92.0223545
cg12439423	0.00079012	52.2476894	5.18506952	526.477232
cg19355621	0.00079246	28110.2734	70.8266085	11156647
cg20725013	0.00079247	13.8180934	2.98050304	64.0629128
cg06312003	0.00079358	3018.66988	27.9866438	325597.021
cg15571373	0.00079522	0.08202127	0.019026	0.35359456
cg21532077	0.00079563	48.166943	5.00608687	463.446691
cg15068083	0.00079741	1.4809E+11	43877.7936	4.9979E+17
cg20427865	0.0008003	17.6222497	3.29322426	94.2977638
cg15767556	0.00080086	1750.23499	22.2378012	137752.942
cg15827505	0.00080211	857679.739	291.131361	2526744392
cg09537434	0.00080218	59428.8574	96.074056	36761111.5
cg18270926	0.0008047	43.5266628	4.78977233	395.544975
cg07722358	0.00080637	12.3604682	2.83947966	53.8060467
cg05212510	0.00080822	0.00755954	0.00043378	0.13174216
cg00207534	0.00080944	21515.9955	62.7299519	7379856.84
cg22923268	0.00081542	44523.0872	84.4965789	23460184.1
cg17794169	0.00081629	49855.8982	88.5039107	28084754.3
cg05031016	0.00081737	11.0727003	2.70845889	45.2673259

cg27124109	0.00253088	2772540430	2059.38754	3.7327E+15
cg23192824	0.00253114	12250.767	27.2025534	5517176.67
cg21693907	0.00253502	45.2289818	3.80846982	537.134571
cg19646112	0.00253526	14.5532623	2.55847178	82.7827945
cg08983217	0.00253762	22.700774	2.98973904	172.36459
cg27639942	0.00254098	14.9628534	2.5824786	86.694613
cg02015876	0.00254294	20.5736242	2.88713091	146.607143
cg02583525	0.00254327	22.8577184	2.99561413	174.413416
cg25479916	0.00254453	8.64265575	2.12992462	35.0695502
cg11538128	0.00254642	15.1952393	2.59548773	88.9602736
cg17604407	0.00255112	1564440204	1666.30104	1.4688E+15
cg24238564	0.00255583	76042.5467	51.2579465	112811170
cg21352006	0.00255588	33.6298692	3.42599687	330.113583
cg14638988	0.00255924	38.0542465	3.57646367	404.904345
cg25510614	0.00255954	29.2252305	3.26057484	261.951999
cg09806575	0.00256231	18547311.3	350.565194	9.8128E+11
cg08196842	0.00258545	25.3069899	3.09368976	207.016148
cg04986336	0.00258906	390.409231	8.04601704	18943.4557
cg19792802	0.00259007	12.4359681	2.41266377	64.1006442
cg10648455	0.00259192	0.00082775	8.18E-06	0.08379407
cg02451774	0.00259879	47.69454	3.85577324	589.964453
cg21759784	0.0026009	0.02430776	0.00216318	0.27314789
cg24503449	0.00260511	40.8393543	3.65024785	456.914964
cg27179622	0.00260567	10.9341514	2.30439731	51.8815342
cg13401703	0.00260794	9.35297311	2.18185428	40.0934687
cg04040095	0.00260916	127.110577	5.42272911	2979.51427
cg06466348	0.00261131	18.0149734	2.74197576	118.359641
cg16391678	0.0026143	41.2488163	3.65982997	464.902703
cg26669421	0.00261466	63864795.7	527.75194	7.7285E+12
cg23737575	0.00261534	0.0610397	0.00988042	0.37709379

cg25533361	0.00082086	157886.593	142.245583	175247454
cg01636650	0.00082233	28.944927	4.02924981	207.931709
cg01931792	0.00082257	36.4706698	4.43368257	300.001125
cg06222206	0.00082559	0.04209047	0.00657379	0.26949565
cg16053142	0.0008259	448046.242	218.259037	919757720
cg19690901	0.00082764	418325.542	211.864638	825981443
cg21551185	0.00082775	60.7087744	5.46837735	673.976035
cg09415957	0.00082795	82.3581714	6.20358198	1093.37934
cg23873523	0.00083592	617.069683	14.2306971	26757.2973
cg05257472	0.00083689	15.7511302	3.12444385	79.4055245
cg14220654	0.00083691	8909.47508	42.876325	1851342.11
cg15979473	0.00083718	44.3736809	4.79319353	410.795756
cg05997362	0.00084112	372.335291	11.5284406	12025.3531
cg18140857	0.00084231	35.8826233	4.38575933	293.578046
cg20631254	0.0008456	5.2505E+10	26580.0415	1.0372E+17
cg06726056	0.00085516	9.5249E+10	33517.1916	2.7068E+17
cg25463478	0.00085671	24245234.9	1104.43513	5.3225E+11
cg22188058	0.00085682	4385.48285	31.6826866	607033.743
cg15376007	0.00086192	1019837.38	298.040562	3489687059
cg20716058	0.00086344	29.7629376	4.04318415	219.09278
cg01861031	0.00086507	229291294	2762.22788	1.9033E+13
cg15765486	0.00086733	42.5743618	4.68136536	387.189664
cg26881035	0.00086831	19808667.9	1004.95909	3.9045E+11
cg26555401	0.00086974	138.347369	7.59779651	2519.15071
cg02324920	0.00087472	102.458254	6.70613595	1565.38636
cg17157872	0.00088027	21.4105057	3.52016825	130.223819
cg15951557	0.00088534	13.3560288	2.89774396	61.5594432
cg13652991	0.00088616	37.5466526	4.4283943	318.343631
cg15227302	0.0008872	183045967	2458.64855	1.3628E+13
cg19598514	0.00088857	556.12164	13.3759021	23121.5267

cg01528028	0.00261578	35.8669841	3.48517905	369.117493
cg20739396	0.00261678	1713561.66	149.284939	1.9669E+10
cg15826897	0.0026195	11.3487159	2.33255057	55.2156742
cg12718671	0.00262595	3720.63852	17.5549522	788561.022
cg26742995	0.00263684	7.53329153	2.02022578	28.0911578
cg00079898	0.00264956	0.06243459	0.01023169	0.38098075
cg07358855	0.00265232	16.4451759	2.64851203	102.111603
cg01627847	0.00265363	6.09820528	1.87546441	19.8287462
cg08938584	0.00265436	49.5473568	3.88625413	631.698412
cg02447095	0.00265514	257703267	841.859338	7.8886E+13
cg25123046	0.00265779	976389637	1336.10402	7.1352E+14
cg12210040	0.00266248	1420.53688	12.4678846	161849.832
cg19183867	0.0026816	0.03036753	0.00310181	0.29730588
cg07046854	0.00268302	221778476	788.495975	6.2379E+13
cg14796261	0.00269261	151.934218	5.71108675	4041.96391
cg26067203	0.00269478	7.08004248	1.9714883	25.4259696
cg18050520	0.00269752	28.8747481	3.20931508	259.790971
cg08182191	0.00269919	35132879.1	413.095376	2.988E+12
cg14412322	0.00271005	20.2625365	2.83578986	144.781668
cg14744703	0.00271357	127590.985	58.6616314	277514605
cg18154114	0.00271741	144344.937	61.1543623	340702776
cg00727673	0.00272115	63.4435231	4.20635702	956.904184
cg08870042	0.0027277	16.0171532	2.61084621	98.2628531
cg06151074	0.00272872	25.9503405	3.08497822	218.290089
cg24542714	0.00273125	27.8846436	3.16202209	245.903832
cg14176626	0.00273426	19.2536008	2.78118663	133.288842
cg17299902	0.00274353	1.8936E+10	3563.96757	1.0061E+17
cg16854917	0.00274526	9.54349173	2.18049895	41.7694466
cg27298072	0.00274588	0.06130364	0.0098619	0.38107635
cg25396971	0.00274957	29.860168	3.23288867	275.799672

cg25138715	0.00088978	1305.60281	18.9751116	89833.3945
cg11267546	0.00089351	14.4656933	2.99061268	69.9710409
cg04453677	0.00089509	2038272.56	385.796738	1.0769E+10
cg25551287	0.00089532	490451128	3651.14479	6.5881E+13
cg20557104	0.00089579	10.5998949	2.63185849	42.6914182
cg03033176	0.00089701	2402.38004	24.292307	237582.617
cg19711258	0.0008991	713.089434	14.7556127	34461.2284
cg26966013	0.00090128	1019.46271	17.0684402	60890.4045
cg22474865	0.00090161	3.3231E+18	38537068.4	2.87E+29
cg20063141	0.00090533	25.4081589	3.75965342	171.711183
cg21090033	0.00091182	0.14676657	0.04721851	0.45618605
cg11392765	0.00091216	36.4157804	4.35065595	304.806695
cg23631842	0.00091635	3895.64778	29.3560533	516965.664
cg01726823	0.00091944	3035834.98	445.276716	2.0698E+10
cg21263269	0.00092421	0.00140383	2.88E-05	0.06841364
cg09758869	0.00092633	225409.827	153.181157	331696086
cg11050527	0.0009294	72011.8399	95.9616834	54039330.1
cg17613375	0.00093264	2863.18569	25.7033569	318940.141
cg08069043	0.00093322	60836.9459	89.3782177	41409798.5
cg15160780	0.00093467	9996.16899	42.7590723	2336893.41
cg14346035	0.0009349	292.124815	10.1242792	8428.93665
cg20122491	0.00093973	2064472.27	374.375872	1.1384E+10
cg22712329	0.0009411	6823.73793	36.4836369	1276281.73
cg02688226	0.00094565	1040997.63	281.990896	3842946960
cg19786920	0.00094698	54.0974502	5.07699126	576.430798
cg27105224	0.00094735	83134498.4	1675.42661	4.1251E+12
cg11525280	0.00094831	9.33110654	2.48203868	35.0798519
cg03539511	0.00095106	1521.1267	19.7164382	117355.194
cg17062109	0.00095227	7.75097521	2.30050561	26.114962
cg27181253	0.00095251	58.776294	5.2451237	658.64085

cg07177860	0.00275909	101.62502	4.93040937	2094.68299
cg14896516	0.00276053	0.03628964	0.00413738	0.31830214
cg07994696	0.00276137	51.5014218	3.89836393	680.38708
cg01931502	0.00276567	4524945464	2147.34172	9.5351E+15
cg24641522	0.00276765	27.3898541	3.13333079	239.427037
cg18020334	0.00276795	63.0966305	4.17865368	952.743416
cg01041284	0.00276801	744.061852	9.78938384	56553.921
cg14851471	0.0027704	193.319681	6.14714472	6079.65172
cg10802132	0.00277723	13.9387518	2.48040688	78.3294079
cg14098532	0.00279258	24.4688139	3.00797893	199.044897
cg17969560	0.00279518	37.0840086	3.47033613	396.27968
cg21163128	0.00279571	12.7644154	2.40354844	67.7874008
cg25722212	0.00279921	28659.0402	34.2365838	23990144.2
cg26264580	0.00280319	869.537138	10.2711955	73613.1288
cg27370013	0.00281395	72.0665781	4.35414225	1192.7933
cg17900356	0.00282411	21.6051072	2.87508753	162.353546
cg10522845	0.0028319	19.6023425	2.77902295	138.268679
cg02727530	0.00283603	31.9540804	3.28578334	310.75185
cg26844213	0.00283868	22.9072586	2.93032204	179.07332
cg07628705	0.00284645	56.7523553	3.99822207	805.565518
cg06148736	0.00284759	8.6568376	2.09711783	35.735158
cg03110368	0.00284774	14.851658	2.52377334	87.3976056
cg11577646	0.00286043	28644.8491	33.7234192	24331085
cg02245020	0.00286549	7.30318664	1.9766053	26.9839078
cg06571407	0.00287517	8.26399092	2.06113298	33.1339834
cg02399524	0.00288094	11.2224518	2.28810938	55.0425719
cg14998896	0.00288372	19.8895499	2.78274863	142.159514
cg10109500	0.00288436	12.4737342	2.37194587	65.5976373
cg11235152	0.00288733	9.67237535	2.17385202	43.0364366
cg17536086	0.00289086	9334.94689	22.8121685	3819945.19

cg17436656	0.00095311	98.9604095	6.48246283	1510.71636
cg00290086	0.00095586	20.8896545	3.44149531	126.798855
cg20239584	0.00095913	2947.53171	25.718888	337803.998
cg02334620	0.00095991	3.8402E+12	130233.274	1.13E+20
cg26483081	0.00096154	2.5039E+10	16806.2863	3.7306E+16
cg03773198	0.00096171	13.7867149	2.90408211	65.4504593
cg11382133	0.00096243	17.2543252	3.18093859	93.5924197
cg02291466	0.00096473	7536462.28	621.416883	9.1401E+10
cg22837406	0.00096605	38195.7605	72.5799297	20100820.3
cg11830546	0.00096721	365842.201	181.547648	737219772
cg27264345	0.00096726	80.3365605	5.93594774	1087.26748
cg08067677	0.00096804	6409417.37	580.27319	7.0795E+10
cg18135855	0.00096822	4771.0079	31.1541348	730641.906
cg04247218	0.00096845	7.5226E+11	66322.535	8.5325E+18
cg07218516	0.00097052	3.6774E+11	49455.8453	2.7344E+18
cg15838173	0.00097123	12714.7594	46.3132154	3490690.65
cg03891929	0.00097197	1254853.3	298.425125	5276555713
cg14770958	0.00097295	19.6407807	3.34736916	115.242821
cg11155414	0.0009743	460019.605	198.287501	1067228323
cg09827532	0.00097476	1051233457	4568.09953	2.4191E+14
cg05217962	0.00097544	97.82423	6.41760988	1491.14393
cg21201393	0.00098008	13.3908374	2.86278936	62.6362975
cg26080673	0.00098334	2.0355E+10	15039.4933	2.755E+16
cg12729166	0.00098564	17.1139051	3.15955676	92.6983656
cg18701685	0.00098857	7.28164002	2.23437787	23.7302213
cg11540197	0.00098894	650224.158	225.874319	1871799585
cg16335926	0.00099059	12040087.8	735.425917	1.9712E+11
cg09992116	0.00099087	17500.0526	52.2036636	5866481.76
cg13098382	0.00099199	2987.85949	25.5108293	349941.754
cg04726446	0.00099203	15.2802099	3.01507829	77.4390558
cg05044743	0.00289108	13.3038192	2.42384013	73.021155
cg26796815	0.00289625	4242.64625	17.3999817	1034486.55
cg12656692	0.00290508	5048173294	2071.07146	1.2305E+16
cg18129755	0.00290572	188.09322	5.98781328	5908.51078
cg01564135	0.00290754	14.3604733	2.48550143	82.9704584
cg19539664	0.00290803	0.05429164	0.00797634	0.36954085
cg18308285	0.0029114	37.4907453	3.44890299	407.537118
cg21893185	0.00291717	12.8260456	2.38998824	68.8319063
cg21509551	0.00291768	523.785812	8.48231788	32343.9396
cg12782992	0.00291888	13.982738	2.46124914	79.4381032
cg24602417	0.002926	32.45741	3.27928509	321.254003
cg02272993	0.00293067	30.0136005	3.19166501	282.240214
cg15136410	0.00293175	75.9343851	4.38028955	1316.35837
cg27093949	0.00293446	23.8389329	2.94963738	192.665961
cg16215203	0.00293548	5.40581712	1.77806997	16.4351567
cg03225210	0.00294627	5.81319539	1.82188045	18.54855
cg02916964	0.00295696	22.7613226	2.89875231	178.724415
cg15436096	0.00297154	9.35481343	2.13979835	40.8975613
cg00714713	0.00297235	118227.486	53.1843771	262816624
cg13851466	0.00297671	10.269181	2.20816935	47.7572425
cg07844977	0.00298143	7.63248555	1.99575453	29.1893792
cg00122310	0.00298339	139559460	587.300089	3.3163E+13
cg23509869	0.00298421	648.443579	9.03357456	46546.2562
cg11007153	0.00298682	0.09450957	0.01991426	0.44852574
cg10723962	0.00298861	12.5347922	2.36145831	66.5355873
cg18881978	0.00299396	0.08218892	0.01578628	0.42790431
cg23971226	0.0029997	0.13663008	0.03669835	0.50868163
cg02249390	0.00299987	148.416151	5.46248624	4032.47766
cg08216021	0.00300019	92867.248	48.6348886	177327963
cg00303183	0.00300617	40.6344711	3.51654723	469.540186

cg19183867	0.00099385	0.03350902	0.00443767	0.25302804
cg25145905	0.00099435	345413.245	174.216445	684839536
cg12384572	0.00099438	3379.00249	26.7866378	426244.53
cg01966966	0.00099453	16176.8	50.476542	5184365.79
cg11412713	0.00099667	0.02418543	0.0026364	0.2218692
cg07759247	0.00099676	150.595439	7.60296294	2982.91424
cg18337826	0.00099756	5768400877	8875.84257	3.7489E+15
cg05282559	0.00099891	17734768	854.183015	3.6821E+11
cg26181929	0.00100187	3081.1253	25.7257519	369020.627
cg23657870	0.00100221	43928.1085	75.3052885	25624743.7
cg18582180	0.00100513	7.6370378	2.27400263	25.6483196
cg05003322	0.0010054	16.6843838	3.11831332	89.2689846
cg03072378	0.00100963	250.526127	9.30747975	6743.32281
cg15269548	0.001011	2326094.86	372.278081	1.4534E+10
cg24034966	0.00101355	29850.9205	64.0365283	13915143.1
cg23418097	0.00101443	3613384.16	443.5828	2.9434E+10
cg09132215	0.00101621	23.6419793	3.58374972	155.966021
cg05006903	0.00101702	25915.9581	60.3776994	11123923
cg14000147	0.00101916	670099.011	223.986627	2004729879
cg03465285	0.00102179	42708.0642	73.6721843	24758038.1
cg07358855	0.00102317	14.9566334	2.97634449	75.1596074
cg12796272	0.00102454	107829.017	106.859135	108807702
cg21852503	0.0010266	5362364.93	515.308128	5.5801E+10
cg20312228	0.0010278	19.7213509	3.32512671	116.967477
cg19839655	0.00102931	9.89147058	2.51753783	38.8638411
cg09638001	0.00103203	4895283.39	494.659278	4.8445E+10
cg22323778	0.00103478	5.8779E+11	54757.3889	6.3095E+18
cg15044073	0.00103542	1159.7769	17.1261708	78539.5911
cg03432464	0.00103677	0.06824668	0.01372341	0.33939152
cg01841828	0.00103798	75967.1557	92.0934751	62664686.5

cg13145017	0.00300856	2.424E+10	3344.3794	1.7569E+17
cg23817893	0.00300868	13.6823067	2.42989934	77.0424993
cg13860849	0.00301436	32.0031368	3.24067948	316.045067
cg20744437	0.00301719	2785.17642	14.7381531	526335.129
cg26402660	0.00301829	13.8106391	2.43621278	78.2910896
cg04457423	0.00302288	1530.2635	12.0175163	194857.766
cg24881993	0.00303133	704.512111	9.22674828	53793.3082
cg10282345	0.00303372	5.7047E+15	217960.948	1.49E+26
cg03611151	0.00303539	19.6529452	2.74269855	140.824172
cg05281603	0.00304019	35.9270195	3.36329936	383.775154
cg15044073	0.00304211	1401.07422	11.6267273	168835.9
cg04142824	0.00304431	5634236.74	193.036347	1.6445E+11
cg09859398	0.00304574	9.26764101	2.12499015	40.4186203
cg02928644	0.00304905	10.2084191	2.19531295	47.470143
cg12346504	0.00305027	638.148651	8.8979324	45767.2279
cg24719487	0.00305643	18.8021137	2.69812371	131.024192
cg08655760	0.00306263	2533.09636	14.1570981	453240.991
cg20352402	0.00306279	60.970954	4.01465801	925.971084
cg26830834	0.00306697	1.0642E+11	5343.35453	2.1196E+18
cg05060704	0.00306861	15.6005476	2.53110597	96.1544437
cg11142489	0.00307665	31.5970043	3.21104623	310.917566
cg21101743	0.00307733	16.302256	2.56762965	103.505406
cg12265829	0.00308379	7.9137817	2.0108065	31.1456824
cg18342279	0.00308472	7.15617374	1.94354465	26.3491877
cg15038286	0.00308533	29.766405	3.14487616	281.740463
cg26643856	0.0030906	270.102874	6.61788746	11023.9957
cg05343713	0.0030914	69377.7113	43.050812	111804321
cg15897774	0.00309171	160403.875	57.1213264	450434273
cg03356734	0.00309418	22.3151829	2.85159811	174.627478
cg02938682	0.00309521	50.9529934	3.76746376	689.11281

cg13979592	0.00103879	1.5508E+13	203270.163	1.18E+21
cg21872782	0.00104075	23.8870226	3.58474638	159.171609
cg26345046	0.00104414	23.90599	3.58398897	159.45818
cg12361311	0.00104419	3.4065E+10	17200.6382	6.7465E+16
cg18127922	0.00104831	102.745404	6.43603103	1640.23727
cg24439070	0.00104984	409204.318	179.998745	930274122
cg10130314	0.00105492	634387.057	213.96661	1880886635
cg19241089	0.00105582	26.2317323	3.7133663	185.304579
cg04858631	0.00105601	94.2777041	6.20666155	1432.05577
cg10733616	0.00105735	4.1555E+10	18339.9505	9.4154E+16
cg23510258	0.0010589	12.6016315	2.76528698	57.4266314
cg16332610	0.00106019	6.58025439	2.13016183	20.3269757
cg05526364	0.00106029	11.8342238	2.69596336	51.9476101
cg15878555	0.00106171	271087.415	151.443429	485253056
cg19248557	0.00106369	116.550581	6.74639266	2013.52615
cg15876689	0.00106422	50097998.6	1227.20096	2.0451E+12
cg19700658	0.00106631	1561.56657	19.0910143	127729.733
cg01389761	0.00107073	22456.4395	55.4934578	9087407.7
cg13424330	0.00107107	1651831.15	310.727733	8781147838
cg09311905	0.0010715	1198969.64	273.196145	5261890535
cg03524243	0.00107202	20916953.5	858.968352	5.0935E+11
cg23321951	0.00107239	4284444.81	454.854952	4.0357E+10
cg22377027	0.00107243	240989.396	143.529547	404626716
cg02423574	0.00107335	20193.1033	53.1133457	7677193.31
cg23500931	0.00107571	18773761	820.096762	4.2977E+11
cg09350274	0.00108043	10.3022062	2.54425859	41.7156703
cg20310435	0.00108178	458023190	2931.71183	7.1557E+13
cg23740975	0.00108224	0.03532297	0.00475689	0.26229561
cg11913218	0.00108683	661917.21	213.192363	2055112984
cg01272428	0.00108912	446.944738	11.4819809	17397.6599

cg12594581	0.00309621	20.2952879	2.7613677	149.164746
cg01758899	0.0030968	26.9853138	3.03985949	239.552901
cg10325497	0.00310083	90.6446101	4.5731929	1796.65401
cg03340356	0.00310215	23.3079706	2.89217523	187.838374
cg03680032	0.00310329	10.113978	2.18227371	46.874299
cg11408656	0.00310445	36468.0767	34.5448594	38498365.5
cg09731694	0.0031073	11.068831	2.24920664	54.472105
cg05819860	0.00311055	6372.97442	19.1600585	2119764.04
cg18611777	0.0031128	222.360461	6.18083129	7999.59948
cg17930034	0.00312551	16.0344461	2.54571036	100.994782
cg15800776	0.00313314	15957.3604	25.9813558	9800772.19
cg06868247	0.00313682	1278974837	1160.13627	1.41E+15
cg24394414	0.00313695	4596391109	1784.14001	1.1841E+16
cg08236022	0.0031378	13.7315965	2.41448629	78.0939378
cg05951994	0.00314557	18.7499789	2.67994531	131.182419
cg23426747	0.003148	4.2653E+10	3753.31512	4.8472E+17
cg10473588	0.00315155	24239919.3	303.763231	1.9343E+12
cg02671213	0.00315256	43.7776545	3.56221883	538.002612
cg14409958	0.00315638	7.1513824	1.9370398	26.4022816
cg23179456	0.00315916	3.9310518	1.58402739	9.75561937
cg04065086	0.00316072	11.4403755	2.26784511	57.7121393
cg20828120	0.00316165	2691.62172	14.2017462	510136.388
cg23432673	0.00316287	384613308	765.561026	1.9323E+14
cg01850179	0.00316671	30.7203693	3.15898834	298.747885
cg26329618	0.00316908	29.5078711	3.11600422	279.433016
cg15311651	0.00317404	21.0402209	2.78054677	159.210016
cg12045829	0.00317408	14.9085646	2.47688366	89.7358653
cg25337570	0.00317466	403807.101	76.1799496	2140460522
cg25637952	0.00317508	72361297.7	434.66098	1.2047E+13
cg05417332	0.00319057	78894.1693	43.8632562	141902141

cg11595749	0.00109371	2355.91496	22.2852388	249058.82
cg01920373	0.00109557	6297842.53	521.636991	7.6035E+10
cg00088042	0.00109663	4200.77689	28.0491229	629129.35
cg02016764	0.00109795	11.7862119	2.67956916	51.8422115
cg12831102	0.00109806	1034290.78	252.990802	4228443896
cg01026744	0.00110429	39.9392851	4.35867859	365.970205
cg10578938	0.00110477	14.0320101	2.87050376	68.5932941
cg22330492	0.00110491	20.6973006	3.35225089	127.788243
cg06897790	0.00110714	15439584.8	739.730824	3.2225E+11
cg17569080	0.00110744	53661.1823	77.196775	37301072.3
cg05338083	0.00110993	380.685547	10.7052756	13537.3895
cg03317642	0.00111141	63.0186327	5.22189898	760.51798
cg02469457	0.00111281	117476.65	105.22704	131152252
cg02469095	0.00111455	8.62187021	2.36093512	31.4861029
cg17530152	0.00111658	23.405483	3.514828	155.858732
cg12281657	0.00111928	11.258227	2.62455301	48.2930523
cg03180980	0.00111958	191.44065	8.11811773	4514.53451
cg08686462	0.00112148	157.160928	7.50084457	3292.90349
cg02737621	0.00112167	101.846653	6.30996593	1643.86636
cg13932362	0.00112192	11.1296741	2.61179155	47.4270797
cg27157482	0.00112336	32.8022483	4.01663678	267.882696
cg11043909	0.00112409	4547.08619	28.6378232	721981.998
cg25392584	0.00112528	370467324	2585.88787	5.3075E+13
cg19299094	0.00112636	202.260483	8.284016	4938.34186
cg23616741	0.00112696	24.3377072	3.56446209	166.174861
cg12511113	0.0011283	387.883989	10.7304555	14021.2118
cg19301273	0.00113113	72.5851033	5.50299369	957.405645
cg00891278	0.00113346	19.7564549	3.2774748	119.090927
cg01098651	0.0011352	908.08294	15.0226694	54891.3516
cg23652354	0.00113863	12392787.2	661.529467	2.3216E+11

cg21142512	0.00319151	371.87857	7.27556015	19007.9757
cg27573806	0.00320076	4207140.19	165.850149	1.0672E+11
cg10016610	0.00320638	31.1450913	3.16414268	306.565415
cg12317815	0.00320783	293.710471	6.70851552	12859.1549
cg02535060	0.00320877	8.35455373	2.03600174	34.282175
cg11392765	0.0032093	169.418593	5.5784868	5145.24112
cg02441149	0.00322375	194.197795	5.82979782	6468.96939
cg14581419	0.00323725	22.1949555	2.81882042	174.759643
cg24158160	0.00324615	13.1549157	2.36541959	73.1590315
cg04768602	0.00324647	11.7589594	2.27836277	60.6896885
cg06994747	0.00325064	57.9314845	3.88017905	864.923204
cg13946792	0.00325201	0.11097683	0.0256655	0.47986047
cg04900427	0.00325673	10.7925742	2.21284341	52.6380027
cg26807590	0.00325749	0.02646515	0.00235493	0.29742088
cg03719155	0.00325946	31.561608	3.16568018	314.667005
cg15056189	0.00326753	810.625597	9.34442445	70321.4909
cg09729613	0.00327658	12.5984598	2.32764413	68.1896289
cg14770958	0.00327832	20.3810948	2.73232181	152.027855
cg04299389	0.00328952	9.21305945	2.09569036	40.5023882
cg12614442	0.00328975	198839261	581.988551	6.7934E+13
cg21163429	0.00329235	14.8779001	2.45813202	90.0488302
cg17191919	0.00330206	49.4756989	3.66519072	667.862865
cg19812495	0.00331019	371682.654	71.3601333	1935926810
cg20427865	0.0033146	17.6581566	2.59896171	119.975024
cg07871500	0.00331462	891.287341	9.57921919	82928.7971
cg16441026	0.00332242	3.9141E+14	71097.5104	2.15E+24
cg00320059	0.00333497	8583107.25	201.140439	3.6626E+11
cg01066472	0.00334084	29.309448	3.07035087	279.786832
cg07549195	0.00334919	278208.439	64.1351532	1206825459
cg19868691	0.00335727	50.1816692	3.66568675	686.965389

cg04734210	0.00113868	50999951.6	1161.02278	2.2403E+12
cg13861524	0.00113888	442.277952	11.2722768	17353.1746
cg21834204	0.00114335	51.2410467	4.78019147	549.276086
cg16315748	0.00114375	5.8459E+11	47448.7492	7.2024E+18
cg21893185	0.00114502	11.5783305	2.64629408	50.6586698
cg17387989	0.00114902	19.2319438	3.23570043	114.308376
cg26873311	0.0011501	322865256	2393.64286	4.355E+13
cg04309212	0.00115032	881474.444	229.547652	3384905871
cg27231335	0.0011522	47.1435746	4.61721983	481.353868
cg23271831	0.00115257	14.1379945	2.86223893	69.8344522
cg26592281	0.00115429	2517.6199	22.3836942	283170.862
cg17658717	0.00115494	47.7365747	4.6379425	491.334372
cg07694975	0.00115636	15.6907992	2.98171417	82.5703489
cg14598198	0.00115638	48932.6787	72.6044034	32978813
cg02621694	0.00115704	24.7135451	3.57036828	171.063393
cg03316474	0.00115809	16528.5376	47.1610932	5792752.84
cg21086156	0.0011618	6143846051	7619.16888	4.9542E+15
cg05031240	0.00116539	115708.985	101.673247	131682321
cg09484039	0.00116837	12.101081	2.68598733	54.5185604
cg16348385	0.00117243	47.6178145	4.61915377	490.881312
cg18050520	0.0011754	20.9476137	3.33520493	131.566884
cg16446585	0.00117883	0.02526317	0.00273708	0.23317857
cg01385168	0.0011851	309344.548	148.465797	644552830
cg03677101	0.00118559	3.4763E+10	14759.7502	8.1874E+16
cg02916964	0.00118786	17.9524731	3.13246756	102.887351
cg06830702	0.00119184	21.3906413	3.35537135	136.366288
cg07921092	0.00119268	97718.6058	93.7531362	101851803
cg14720706	0.00119336	20.7007037	3.31149209	129.403641
cg02742085	0.0011942	73.3318919	5.45779751	985.299722
cg27572598	0.00119783	2.7044E+10	13190.216	5.5449E+16

cg03531211	0.0033573	21.8494051	2.78200464	171.60162
cg19946641	0.00336603	0.05540311	0.00801067	0.38317721
cg21143446	0.00336937	41.7149907	3.44444252	505.202348
cg02541231	0.00336942	7505.4913	19.260386	2924780.4
cg10398590	0.00337287	48.8049731	3.62738315	656.651172
cg23139584	0.00337659	34.3744928	3.22865688	365.974398
cg00417297	0.00337865	15888226.3	243.062057	1.0386E+12
cg01592312	0.0033795	8330931465	1934.42096	3.5879E+16
cg19598514	0.00338829	553.831975	8.09705609	37881.6514
cg11870261	0.00339886	32.2907646	3.15732651	330.245693
cg23543123	0.0033989	54.1830827	3.74709113	783.489474
cg10328157	0.0034012	8.91288066	2.06201947	38.5250687
cg06208248	0.00340789	4716862.1	161.027984	1.3817E+11
cg12046254	0.00340956	18.9492873	2.64509282	135.751564
cg22434226	0.00341073	61063.5032	38.2226132	97553545.1
cg18436984	0.00341225	47.1432908	3.57461252	621.742875
cg04974751	0.00341286	2578960816	1292.06171	5.1476E+15
cg07566050	0.0034133	64089.3763	38.815696	105819258
cg13050981	0.00341367	30.3141454	3.08876397	297.512993
cg08066035	0.00341495	11.0146715	2.21011464	54.8944318
cg00852573	0.00341892	16.5207762	2.52643965	108.031888
cg25353281	0.00341933	9.61467065	2.11257	43.7580254
cg10219093	0.00342336	36702.5431	32.2124322	41818533.3
cg23097623	0.00342712	498363.791	76.1787796	3260310412
cg26994413	0.0034301	0.07138664	0.01218428	0.41824806
cg08437570	0.00343408	26.5297198	2.95146026	238.467054
cg06383048	0.00344343	144095.234	50.361355	412289077
cg03097541	0.00344395	4.0095E+14	65786.5393	2.44E+24
cg10698419	0.00344489	18035.6393	25.3619822	12825664.9
cg04986324	0.00344694	30.1671094	3.07655832	295.802775

cg12872647	0.00119903	72.4493098	5.42674901	967.227798
cg17956079	0.0012033	36010.8706	62.8785516	20623611.2
cg09639735	0.00120595	1358.43369	17.2304495	107097.734
cg00341935	0.00120904	0.0002506	1.65E-06	0.03797762
cg00448482	0.0012119	1487316.82	271.649672	8143250441
cg22628873	0.00121231	28.9416141	3.76969713	222.197432
cg03448532	0.00121503	186.47903	7.85381257	4427.71306
cg00016066	0.00122026	7128275.14	501.032995	1.0142E+11
cg05372242	0.00122171	28.8808593	3.76134373	221.756929
cg20547295	0.00122199	4262.63113	26.8971748	675536.531
cg04788931	0.00122479	2.7869E+15	1207386.43	6.43E+24
cg15676837	0.00122869	39.086045	4.232733	360.929666
cg14226131	0.00122911	1189.08719	16.2311812	87111.8579
cg23401796	0.00123009	0.24854898	0.10684482	0.57818986
cg16285902	0.00123049	733321520	3080.99277	1.7454E+14
cg20352402	0.00123289	50.5642256	4.68082235	546.216181
cg13904877	0.0012331	0.05395143	0.00918009	0.31707289
cg13934625	0.0012333	17.8732441	3.10897014	102.751986
cg03714619	0.00123469	6.45204587	2.08202543	19.9944224
cg23621729	0.00123694	14.0667629	2.82825138	69.9633064
cg04339790	0.0012383	26.8104603	3.64405226	197.253149
cg14847688	0.00123836	45.3212803	4.47945548	458.541994
cg21834860	0.00123878	567604056	2765.40062	1.165E+14
cg23162960	0.00124179	1.7717E+10	10664.4836	2.9433E+16
cg16776006	0.00124316	906389.491	219.3107	3746018362
cg25234159	0.00124614	2249.68745	20.7424336	243997.1
cg07626637	0.0012487	5.202E+10	16165.7366	1.674E+17
cg25393792	0.00124984	170684.729	113.396057	256916136
cg01859118	0.00125084	209673.165	122.872332	357792803
cg15338778	0.00125089	1175659.82	241.784527	5716561054
cg11449372	0.00344853	7505.99645	18.979435	2968475.24
cg16473288	0.00345083	0.07726293	0.01388949	0.42978966
cg15203597	0.00346958	5297.58296	16.8558314	1664965.94
cg04292889	0.00347404	6624968.98	176.336624	2.489E+11
cg00324161	0.00347508	14.5719843	2.41625866	87.8807923
cg13850380	0.00348909	20.7830511	2.71355574	159.176835
cg08084502	0.00349032	37.5555135	3.29642143	427.86295
cg03965648	0.00349074	34.3128421	3.19983533	367.947414
cg16096311	0.00349166	13.4617636	2.35193637	77.0510128
cg17736030	0.00350492	3265.64242	14.2885956	746358.893
cg18113295	0.00350701	9.38426746	2.08723903	42.1918498
cg16403299	0.00351551	675.494551	8.50046476	53678.5812
cg16131859	0.00351649	111.499414	4.70353577	2643.14336
cg26084319	0.00352548	5296302793	1556.13435	1.8026E+16
cg15681626	0.00353462	11.3051909	2.21594624	57.6761927
cg03368930	0.00353637	2679870.09	128.4414	5.5914E+10
cg03773198	0.0035398	12.7151988	2.30244827	70.2192885
cg12250513	0.0035402	36.9268383	3.26614555	417.492537
cg03315058	0.00354772	28.7893108	3.00854153	275.490435
cg17526770	0.00355254	57.5714089	3.7744022	878.143596
cg01169778	0.00356208	294.554844	6.43737457	13477.9412
cg13312337	0.00356237	592.279315	8.09189323	43351.386
cg14273822	0.0035701	6.64468279	1.85885712	23.7521264
cg00444390	0.00357105	0.0659052	0.01057929	0.41056598
cg24905333	0.003574	1.56E-10	3.95E-17	0.00061739
cg26565223	0.00357457	0.04357839	0.00529504	0.3586519
cg16976499	0.00358023	170.098213	5.3677041	5390.27516
cg24210813	0.00358549	29.9928387	3.04130338	295.784492
cg02742085	0.0035883	137.249315	4.99980955	3767.61841
cg20874811	0.00359214	898115.228	88.3580823	9128887145

cg04722722	0.00125273	57.8306417	4.91758067	680.087088	cg08124910	0.00359541	15.5831824	2.45369923	98.9671313
cg02792140	0.00126077	3587774.05	372.304932	3.4574E+10	cg17775899	0.00359543	46.2204989	3.50068323	610.262161
cg05673638	0.0012631	112664786	1436.24131	8.8379E+12	cg09755397	0.00360005	180.122866	5.45790124	5944.45474
cg01258201	0.00126705	14.5578392	2.8567441	74.186093	cg07554759	0.00360046	51.9233847	3.63491846	741.705188
cg22690294	0.00127528	49.9468727	4.62518556	539.370813	cg18301624	0.00361086	12186.6796	21.5879216	6879548.76
cg22219278	0.00127688	1428118.24	256.899774	7938978268	cg27105224	0.00361124	50580319.5	327.801728	7.8046E+12
cg26440289	0.00127699	4.56135492	1.81155236	11.4851545	cg01804934	0.00361753	8.74930269	2.02986719	37.7119735
cg00956573	0.00127882	15344404.6	649.999736	3.6223E+11	cg25538450	0.00361809	16054.0484	23.5878993	10926469.8
cg10612237	0.00128219	24721822.7	781.517393	7.8203E+11	cg16608407	0.00361933	26.6654737	2.92004667	243.505521
cg04129227	0.00128627	254.484741	8.72830794	7419.82109	cg09798090	0.00361979	8214.13806	18.9481637	3560876.14
cg07138491	0.00128762	40.2989906	4.24413884	382.647388	cg02695343	0.00362118	27.2078344	2.93894042	251.88202
cg24548682	0.00128837	220.659885	8.25079734	5901.34297	cg08952506	0.00362383	19.3253853	2.62809228	142.107079
cg11267955	0.00129115	18.7924084	3.14798451	112.184355	cg14876761	0.00363032	22.7472602	2.77054827	186.7637
cg14379630	0.00129366	526702.255	172.227989	1610744387	cg12014818	0.00364431	21.370058	2.71236917	168.369182
cg17346177	0.00129376	5369.72551	28.6896839	1005028.57	cg04997812	0.00365241	97.0794977	4.43846192	2123.35467
cg04556868	0.00129731	64.4377283	5.09063341	815.658974	cg20187572	0.00365461	3810.7226	14.6629973	990357.323
cg13747794	0.00129766	322.864972	9.5533411	10911.5532	cg10140906	0.00365473	47.9496695	3.52675693	651.922108
cg03919114	0.0012994	12.6912215	2.69777831	59.7036095	cg09149672	0.00365669	0.14445963	0.03918345	0.5325867
cg27043726	0.00130091	9.49323115	2.40822037	37.4224215	cg00049382	0.00365784	23.5207341	2.79608888	197.856704
cg04740941	0.00130342	733.496049	13.1427099	40936.4933	cg00472687	0.00366087	136659.698	46.9764251	397558414
cg04809136	0.00130484	328740.676	142.402225	758909718	cg20354848	0.00366629	12872.6786	21.748992	7619013.08
cg10832166	0.00130804	0.05896287	0.01049302	0.33132683	cg17956079	0.003668	111906.092	43.9454667	284966216
cg26572163	0.0013091	144235.639	103.024337	201932089	cg10147666	0.00367226	19.6641058	2.63546124	146.720829
cg17963935	0.00131032	851278345	3046.7468	2.3785E+14	cg24575275	0.00368127	64.5832244	3.87742866	1075.71105
cg00713366	0.00131136	3797388.55	368.579793	3.9124E+10	cg00246980	0.0036818	194540.793	52.4345428	721778391
cg25562664	0.00131423	17.5626887	3.0573353	100.887866	cg26831415	0.00368201	40.9008774	3.34207551	500.551759
cg10938446	0.00131711	0.00428469	0.00015382	0.11934976	cg18404009	0.00368612	2.7566E+11	5231.61331	1.4525E+19
cg20508523	0.00132975	18.3558825	3.10465858	108.526723	cg07823452	0.00368785	3.00E+22	20202566.4	4.46E+37
cg03363567	0.00133308	2.6766E+10	11435.2397	6.265E+16	cg24546463	0.0036893	34.6121221	3.16389408	378.646998
cg18351191	0.00133331	0.00025736	1.65E-06	0.04009355	cg02379560	0.00369034	15.7553167	2.44972839	101.329602

cg16508199	0.00133567	24637621.7	751.456358	8.0778E+11
cg13038216	0.00133656	37.2328759	4.08464791	339.389606
cg11229862	0.00133731	10.5387433	2.49962772	44.4326603
cg00515408	0.00133758	101.430034	6.03111087	1705.83034
cg08633134	0.0013397	65653200.3	1097.05445	3.929E+12
cg04229059	0.00134179	380.365441	10.0751513	14359.8706
cg10454568	0.00134256	17.1978198	3.02236247	97.85888
cg01789728	0.00134283	805.647574	13.4853903	48131.1996
cg13031167	0.00134286	239.940564	8.42061688	6836.96633
cg08438366	0.00134763	18.0839528	3.08013785	106.173608
cg08104568	0.00135173	44.6149278	4.37213968	455.267199
cg07924703	0.00135199	230.644842	8.27550254	6428.25532
cg16478733	0.00136182	153.337422	7.04790195	3336.08002
cg04117874	0.00136399	4.0403E+17	6760419.6	2.41E+28
cg14856108	0.00136537	618957817	2570.5139	1.4904E+14
cg18511007	0.00136563	12.2950974	2.64644217	57.1217544
cg01281718	0.00136573	20.2930335	3.2141135	128.124663
cg07142961	0.00136843	318022.498	136.006148	743630425
cg01191114	0.00136904	170045.039	106.660312	271097230
cg24255928	0.00136971	57.7476581	4.81878413	692.040135
cg14070281	0.00137015	2664.92604	21.2843086	333665.093
cg08444060	0.00137405	3.5481E+11	29912.885	4.2087E+18
cg05401965	0.00137563	10.0542273	2.44549597	41.3361904
cg24678095	0.00137592	1464.66379	16.8480696	127328.536
cg05226740	0.0013818	18.4589907	3.09234788	110.186289
cg27579745	0.00138356	231.836223	8.23483626	6526.90988
cg16585234	0.00138569	16.8705957	2.98515211	95.3442194
cg27458485	0.00138777	23.3549244	3.38472524	161.151188
cg01038149	0.00138878	11.1395463	2.54135878	48.8280096
cg12594581	0.00139358	15.2436863	2.86779766	81.027325

cg20368283	0.00369329	74.1078687	4.0506572	1355.8235
cg18607468	0.00370201	446.554293	7.25267587	27494.7813
cg11376305	0.00370286	4.46140457	1.62514891	12.2475735
cg15551525	0.00371741	37617.2054	30.5061942	46385797.3
cg15198000	0.00372121	32.4457538	3.09136778	340.537592
cg10130314	0.00372504	1158121.06	92.5443409	1.4493E+10
cg16269716	0.00372951	122.140916	4.74835598	3141.80391
cg13784855	0.00373139	11914.3887	20.9545662	6774306.64
cg17436656	0.00374125	93.3570646	4.34763442	2004.66292
cg09162146	0.00374335	45.5636947	3.44557391	602.5267
cg23599104	0.0037471	7.81640772	1.94624924	31.3917809
cg06070445	0.00375112	15.9268793	2.45026597	103.525694
cg12309516	0.00376052	753676.302	79.7582917	7121867281
cg07880384	0.00377019	115.439387	4.64474011	2869.10606
cg06182274	0.00377646	62.6299567	3.80940378	1029.69171
cg18582180	0.00378737	8.06200732	1.96263647	33.1166586
cg13478617	0.00378746	58779.584	34.734455	99470094
cg12272369	0.00378806	2803.32186	12.9949792	604742.29
cg02392575	0.00378893	1431676.6	97.3981243	2.1045E+10
cg22608128	0.00379689	9.40954325	2.06227315	42.9329665
cg01601628	0.00380204	221.756679	5.71746993	8601.01153
cg18782736	0.00380322	46.6169107	3.45569846	628.855899
cg13078331	0.00380758	3.9384E+11	5514.51959	2.81E+19
cg15778054	0.00381153	12.9309359	2.28354285	73.223545
cg01518459	0.00381803	0.00113792	1.15E-05	0.11237692
cg23907982	0.00381942	18.2992877	2.5530903	131.160237
cg08799865	0.00382214	1.1402E+13	16198.9402	8.03E+21
cg13039082	0.00383496	23.9888194	2.78333246	206.753402
cg03677101	0.0038351	1.151E+11	3658.11037	3.6215E+18
cg05652493	0.00383825	26738283.5	246.692786	2.8981E+12

cg21368609	0.00139625	28.8631255	3.66960173	227.021916
cg27018309	0.00139776	0.05545322	0.00940631	0.32691442
cg20743280	0.00139846	11.1472298	2.5396805	48.9277024
cg16978004	0.00140196	0.03506557	0.00448778	0.27398698
cg27294268	0.00140374	31.320152	3.78349488	259.271376
cg16408670	0.00140724	0.04245733	0.00610646	0.29519964
cg12220843	0.0014078	561494.039	166.061558	1898546296
cg13784855	0.00140941	2544.03054	20.6500012	313418.45
cg00314660	0.00141184	99.8385184	5.91242569	1685.89515
cg21325316	0.00141602	118263.056	90.6525172	154283088
cg01597066	0.00141663	5.42747798	1.92056959	15.3379067
cg10779674	0.00141664	272808.223	125.116658	594839468
cg22219587	0.00141817	8067.91528	32.144784	2024939.94
cg05445326	0.00142234	0.10692358	0.02707338	0.42228387
cg16127594	0.0014233	15.4636077	2.87449378	83.1879213
cg20375554	0.00142389	248.078351	8.37959984	7344.36842
cg06324635	0.0014258	2273.34652	19.6742111	262684.2
cg25149391	0.0014266	91.7771744	5.70842739	1475.54645
cg12007596	0.00142679	10.5495551	2.47963425	44.8828745
cg10006582	0.00142694	27195.5152	51.1885511	14448466.1
cg24754334	0.00142724	22085276.7	676.904274	7.2057E+11
cg17892169	0.00143078	23.2922747	3.36322058	161.312661
cg05299836	0.00143326	115.79103	6.23572183	2150.12199
cg04445214	0.00143381	57717.3862	68.2119652	48837424.1
cg01963221	0.0014341	9490165.19	486.722549	1.8504E+11
cg08196842	0.00144477	19.2284087	3.1187197	118.552399
cg26342670	0.00144545	1456.33904	16.4790101	128704.539
cg19969694	0.00145156	10.114517	2.43429296	42.0259421
cg06895295	0.00145157	46.975685	4.39326156	502.295379
cg26227186	0.00145581	79.7314495	5.38036228	1181.53829

cg08881680	0.00384545	6.81286423	1.85475488	25.0249343
cg23725690	0.00384891	985.996539	9.197413	105702.46
cg13424330	0.00385014	1663429.45	100.522548	2.7526E+10
cg01511567	0.00385152	38.6198964	3.2410211	460.193362
cg16017089	0.00385215	5.35977737	1.71653133	16.7356185
cg04788444	0.00385631	49231166.7	298.455664	8.1208E+12
cg15338778	0.00386456	5421417.64	146.403821	2.0076E+11
cg12045833	0.00386925	211.983126	5.59606387	8030.08092
cg26497348	0.00387514	19.1001841	2.5804573	141.376891
cg19408398	0.00387602	0.03872614	0.00426352	0.35175482
cg23606286	0.00388174	1.6142E+10	1902.51293	1.3696E+17
cg07498606	0.00388312	33383.2792	28.3839408	39263164.3
cg06561166	0.00388446	11690.5612	20.2576187	6746559.01
cg21714061	0.0038895	45.3584465	3.40376543	604.444903
cg17952262	0.00389359	13.119719	2.28498364	75.3296537
cg10324701	0.00389388	0.04524427	0.00552992	0.37017588
cg07602200	0.00389761	35.4021673	3.1416727	398.931897
cg11934386	0.00390175	2.0249E+11	4245.21652	9.6585E+18
cg26883434	0.00390386	137.670817	4.85519913	3903.70269
cg20631254	0.00391338	4.0309E+10	2515.53213	6.4592E+17
cg16978004	0.0039158	0.03006164	0.0027796	0.32511976
cg03507534	0.00391659	25.6904557	2.83112319	233.122853
cg26818820	0.00392722	27.5045209	2.8918051	261.60085
cg17166812	0.00392879	336.005872	6.44671699	17512.7814
cg02700626	0.00393515	9.39433341	2.04903678	43.0707252
cg12411312	0.00394113	164027.515	46.7124497	575971199
cg20918957	0.00394528	9.21399997	2.03549423	41.7086888
cg13794888	0.00395115	60750208.5	309.207038	1.1936E+13
cg15780361	0.00395311	11.2817456	2.17098056	58.6268644
cg17925360	0.00395682	2.05E+20	3138004.94	1.35E+34

cg05950711	0.00145597	31.4353316	3.7623581	262.649128
cg15557486	0.00145846	2661664.96	294.159169	2.4084E+10
cg26045220	0.00145903	19.407355	3.12475626	120.535938
cg23168192	0.00145964	14.3744427	2.78420282	74.2132005
cg13475699	0.00146237	3137001.29	312.626724	3.1478E+10
cg04065086	0.0014638	10.2725692	2.44616562	43.1392208
cg24105407	0.00146496	28.3827252	3.61334851	222.945306
cg00557469	0.00146521	22791.1559	47.1143227	11025029.2
cg00099023	0.0014727	10437218.3	492.897642	2.2101E+11
cg26181939	0.00147452	18.2870213	3.04893186	109.682723
cg26399396	0.0014769	599.903142	11.6251099	30957.4518
cg03627290	0.0014794	19.7183147	3.13661182	123.959214
cg04346127	0.00148005	863545.42	188.738777	3951020045
cg22628146	0.0014803	1040.35917	14.34506	75450.8667
cg27494615	0.00148474	12462.2943	37.106219	4185518.85
cg11845111	0.00148531	108.305235	6.02060724	1948.31245
cg23637705	0.00148614	2.3812E+17	4549032.84	1.25E+28
cg10715223	0.00148854	6.0110328	1.98784998	18.1766812
cg15689069	0.00149053	33230.8006	53.9110129	20483497.7
cg10628982	0.0014925	2.6544E+14	333485.44	2.11E+23
cg13690989	0.0014939	308.218502	8.97174058	10588.6527
cg08949329	0.00150141	14.4748554	2.77984545	75.3716146
cg09321817	0.00150363	10.7495622	2.48026015	46.5891001
cg06680214	0.00151047	2563602.88	281.631585	2.3336E+10
cg20701182	0.00151179	28830.4279	50.6392326	16414023.9
cg10867327	0.00151233	3985.9967	23.7682218	668462.702
cg11622362	0.00151424	4106.53614	24.0261662	701886.391
cg10453019	0.00151801	18.947726	3.07585577	116.720791
cg19641839	0.00151939	0.02484881	0.00253189	0.24387414
cg10348922	0.00152096	14.5610195	2.78071309	76.247812

cg11300971	0.00395782	160.260487	5.07143024	5064.33543
cg10502118	0.00396447	12.3323649	2.23251095	68.1238423
cg04394254	0.00397071	116.626048	4.57600351	2972.383
cg17697381	0.00397669	527118.144	67.2837383	4129579363
cg05413277	0.00397915	21.1268402	2.64952484	168.461669
cg00150500	0.00397922	2910148.81	116.049612	7.2977E+10
cg07811634	0.00398586	1.217E+12	7222.21805	2.05E+20
cg12296552	0.00398642	0.08641798	0.01632018	0.45759716
cg05338083	0.00398753	453.656387	7.04975579	29193.0847
cg09479758	0.00398943	135.234503	4.78944505	3818.47385
cg06830702	0.0039911	48.2250015	3.4457294	674.937146
cg12804865	0.00399846	82410741.9	335.430619	2.0247E+13
cg00154846	0.00400958	43.7544937	3.33602724	573.872927
cg23481605	0.00401106	44.89839	3.36323956	599.38205
cg14113931	0.00401876	0.07941893	0.0141388	0.44610353
cg18361024	0.00402429	5027.80155	15.1059537	1673432.15
cg18732172	0.0040244	10.9667288	2.144545	56.0814252
cg27529930	0.00402537	9.05613754	2.01759579	40.6491864
cg24368491	0.00402611	0.04220718	0.00488244	0.36486808
cg07569219	0.00403521	537699.613	66.7445794	4331750634
cg26657230	0.00403716	11632.0536	19.6890486	6872077.68
cg01052428	0.00404482	768589.227	74.6043938	7918158297
cg16575322	0.00405207	14.5186829	2.34183768	90.0114273
cg11449134	0.0040529	38.3628247	3.18967535	461.3969
cg09948076	0.00405585	26.6959348	2.84176636	250.785196
cg15876689	0.00406076	17900673.5	202.137786	1.5852E+12
cg21654383	0.00406674	54.5656208	3.56422634	835.358557
cg09278980	0.00407185	9.9113531	2.07234199	47.4028519
cg11406274	0.00407509	42.6826845	3.29470885	552.950696
cg08632164	0.0040774	4.85069427	1.65121191	14.2496761

cg01130898	0.0015216	74.3519596	5.18196392	1066.81829
cg26542888	0.00152331	121.944448	6.25752308	2376.41126
cg19584674	0.00152361	153.582687	6.83318105	3451.92696
cg27200466	0.00152397	19.7310786	3.12175159	124.710583
cg07528363	0.0015258	17.675235	2.99276492	104.389733
cg26757053	0.00152744	12135.2667	36.1809444	4070228.13
cg10082589	0.00152803	9.59994335	2.37035681	38.8797636
cg14022778	0.00153228	9.28950573	2.33998817	36.878356
cg02978959	0.00153388	35527.6068	54.377175	23212144.5
cg26212303	0.00153532	4178003136	4661.95251	3.7443E+15
cg01184539	0.00153966	124.294901	6.28491326	2458.14411
cg13995101	0.00154313	1564.55508	16.4865954	148474.112
cg07450214	0.00154702	584.177589	11.3166068	30155.9877
cg04809274	0.00155253	25.2572633	3.41864613	186.60292
cg17721173	0.00155402	192282.542	102.607514	360330100
cg19151808	0.00155406	21.7140639	3.22693343	146.11413
cg17775899	0.00155455	28.7203624	3.58913419	229.821224
cg26608667	0.00155821	0.0669662	0.01254398	0.35749986
cg02535060	0.00156134	6.90845248	2.08574549	22.8823296
cg24217704	0.00156429	172.974111	7.09548294	4216.7733
cg23437162	0.00156613	102.789711	5.81968919	1815.51356
cg11841529	0.00156926	8.23934043	2.22893959	30.456963
cg10830649	0.00156989	369.532882	9.45833304	14437.486
cg09552892	0.00157029	16.9390091	2.93096337	97.89615
cg02362385	0.00157259	83834.9324	74.2374547	94673179.7
cg10282491	0.00157807	22.7720195	3.27691779	158.247752
cg22416703	0.0015825	1.7365E+12	44257.3747	6.8132E+19
cg07793638	0.00159041	25.6703534	3.42448755	192.427928
cg23195373	0.00159049	48.0376638	4.34327201	531.308457
cg08886727	0.001591	10.3268179	2.42423416	43.9904569
cg01029638	0.00408043	10.9437815	2.13780861	56.0229544
cg03744440	0.00408391	8.95785926	2.00583063	40.0049942
cg24683514	0.00409078	821782.776	75.3351699	8964298247
cg04650403	0.00410488	11640.9635	19.4650119	6961826.24
cg00431235	0.00411074	8.0263E+11	5935.24447	1.09E+20
cg12876594	0.00411419	19.9927813	2.58382214	154.697684
cg03969906	0.00412722	44089.8494	29.5624963	65756112.2
cg22112476	0.0041304	90131.4076	37.0504361	219259785
cg00813378	0.00413574	7.87965835	1.92203503	32.3037898
cg21531300	0.0041559	14.6187359	2.33500666	91.5232676
cg19967582	0.00416114	7455357.51	148.623547	3.7398E+11
cg04543413	0.00416193	8.22965294	1.94669979	34.7907716
cg13646565	0.00416435	724114.73	71.0703022	7377795315
cg20495019	0.00417148	132648443	367.880905	4.783E+13
cg21214613	0.00417832	57.2131845	3.58855968	912.162197
cg23973885	0.00418194	58.0863227	3.6048041	935.978986
cg01035068	0.00418327	416.295353	6.71162859	25821.128
cg01565438	0.00418499	16.3851675	2.41718531	111.068735
cg21116657	0.00418889	1835063.24	94.7364463	3.5546E+10
cg01819512	0.00419139	43.2388926	3.28195043	569.661813
cg16317734	0.00419543	82.1236466	4.01689931	1678.97993
cg26724572	0.00419849	0.00451042	0.00011175	0.18204828
cg16849024	0.00419885	13.9053554	2.29366265	84.3013725
cg21568286	0.00420368	2001344444	856.211557	4.678E+15
cg13652991	0.00420433	42.5813354	3.26326163	555.631246
cg11352083	0.00421034	22.8453749	2.6806971	194.692326
cg03583016	0.00421507	12.6468883	2.22440833	71.9039671
cg01407244	0.00421509	24.4296136	2.73719684	218.035479
cg13442432	0.004217	10.593067	2.10343751	53.3474692
cg24703125	0.00422245	113469106	344.248371	3.7401E+13

cg19838156	0.0015935	147.676268	6.64622256	3281.30451
cg04465834	0.00159435	273.580845	8.39531826	8915.26404
cg22995449	0.00159621	32.5189291	3.74313537	282.512024
cg19921353	0.00159817	372.246005	9.42745542	14698.2491
cg22273939	0.00160405	35.2013621	3.85345524	321.564886
cg03892045	0.00160591	9.05184726	2.30333163	35.5727928
cg12727795	0.0016074	21.2290801	3.18048967	141.699515
cg07575193	0.00160784	15.490552	2.82258048	85.0134136
cg26139475	0.00161028	2.8372E+10	9063.90649	8.8809E+16
cg27106909	0.00161965	19.1496482	3.05477803	120.04441
cg25619837	0.00162162	117610.283	82.7087816	167239541
cg20118643	0.00162284	4534.13587	24.1358406	851778.416
cg04357685	0.00162401	3.04E-09	1.54E-14	0.00060248
cg23302845	0.0016263	185247129	1334.56723	2.5714E+13
cg09118164	0.00162763	78811.6248	70.9182167	87583592.7
cg25060020	0.00162828	29767.1792	49.0716686	18056956
cg13168437	0.00163527	14336.3919	37.143446	5533469.7
cg04700925	0.00163805	244570.134	108.308845	552259150
cg15058894	0.00163992	17.6856833	2.95815099	105.736115
cg02404489	0.00164017	150824616	1223.44757	1.8593E+13
cg19056004	0.00164217	19.3495254	3.05957052	122.371467
cg15780361	0.00164324	10.292483	2.41069859	43.9437794
cg19297231	0.00164382	71609.1453	67.9599041	75454339.8
cg12940439	0.00164761	7.17793628	2.10347844	24.49408
cg09142117	0.00164993	3792577.3	303.01251	4.7469E+10
cg24688655	0.00165442	13.6018068	2.67543649	69.1510144
cg04380635	0.00165747	693023960	2149.04034	2.2349E+14
cg08021273	0.00165881	4536.44404	23.8843643	861623.288
cg09020665	0.00165908	90.4757974	5.4619	1498.72204
cg07570421	0.00166285	9526.33029	31.5488718	2876520.26

cg20252067	0.00422261	3444.65765	13.0019711	912605.197
cg07201835	0.0042338	0.06504728	0.00999973	0.42312637
cg05909880	0.00423695	2.0218E+13	15387.1679	2.66E+22
cg03381111	0.00424013	0.03737192	0.00392837	0.35553168
cg22776392	0.00424581	11.5239018	2.15726887	61.5594626
cg10481660	0.00424996	8.01640569	1.92424775	33.3963026
cg20215212	0.00425201	143.911829	4.77029638	4341.57814
cg13519902	0.0042533	33.9688548	3.02945721	380.887734
cg17649836	0.00425398	918620.092	74.9393196	1.1261E+10
cg11845111	0.0042618	99.1966745	4.24019766	2320.64187
cg16194715	0.00426252	41.0896863	3.21428411	525.268541
cg14898768	0.00427104	0.03348383	0.00325828	0.34409824
cg04807004	0.0042756	114.523283	4.43084949	2960.06043
cg10173186	0.00428031	19.1283585	2.52543177	144.883779
cg05257472	0.00428087	23.8219381	2.70543859	209.757019
cg07759247	0.00429246	217.039023	5.40710551	8711.85842
cg03135127	0.00429626	5940.08365	15.2606295	2312132.25
cg15049402	0.00430488	23.7014555	2.69748924	208.252542
cg01270593	0.00430876	28.51738	2.85787102	284.561813
cg05816193	0.00431176	16.2724346	2.39670289	110.481832
cg03887520	0.0043137	13.7121065	2.27131668	82.7809991
cg10130900	0.004315	16.9207994	2.42583209	118.026904
cg03400491	0.00431743	2202464.49	97.0379172	4.9989E+10
cg18353196	0.0043216	35.5544137	3.05991441	413.121467
cg21883802	0.00432344	33.6188105	3.00639241	375.940419
cg04021672	0.00432356	46.1642912	3.320245	641.862809
cg07707586	0.0043292	25.9824513	2.77242489	243.500835
cg02033116	0.00432925	3515032.2	111.98763	1.1033E+11
cg02469095	0.00432997	8.5528432	1.95785568	37.36288
cg02993937	0.00433379	1.8062E+10	1621.82071	2.0114E+17

cg23130076	0.0016648	274.994228	8.2947435	9116.83713
cg03177847	0.00166684	13.6841833	2.67849519	69.9112224
cg07517893	0.00167468	4.64446555	1.78230039	12.1029318
cg00530805	0.00167525	12.0981877	2.55521365	57.281373
cg16734497	0.00167706	300306.5	115.043556	783911738
cg05136476	0.00167754	293.974736	8.4845296	10185.7321
cg02322879	0.00167946	0.10752323	0.02674907	0.43221104
cg07065570	0.00168189	384040.452	125.925572	1171224130
cg04452959	0.00168235	11.4777032	2.50358494	52.6196135
cg00569934	0.00168324	6093.99844	26.4977977	1401505.8
cg13520531	0.00168497	36.535227	3.86831835	345.065399
cg04292889	0.0016872	418713.964	129.778111	1350931857
cg10666406	0.00168837	1941148.51	230.85589	1.6322E+10
cg06301550	0.00169203	8.01408862	2.18572733	29.3840936
cg02703870	0.00169511	11.9727981	2.54088648	56.4164887
cg00420348	0.00169693	6.54254054	2.02467158	21.1416198
cg26831415	0.0016978	33.6374816	3.7441646	302.198297
cg21538450	0.00170047	17.1032167	2.90358231	100.744526
cg01716223	0.00170047	3752.18161	21.9731368	640730.863
cg11299660	0.00170356	1016869.65	179.750444	5752552620
cg26942275	0.00170489	20.8105078	3.12408323	138.625383
cg22339486	0.00170605	51600.2313	58.6634649	45387429.4
cg10385303	0.00170701	116.822202	5.96688099	2287.19607
cg01420215	0.00170722	21588.867	42.286021	11022062.8
cg15656257	0.00170778	77.1188642	5.10532606	1164.92446
cg25533556	0.00170859	3.3162E+12	49789.5359	2.21E+20
cg05817661	0.00170924	2.4843E+11	18822.388	3.2788E+18
cg16552437	0.00171101	1475.43137	15.4370102	141018.094
cg04465286	0.00171553	1811745.43	221.948867	1.4789E+10
cg05635953	0.00171618	1017322692	2381.38965	4.346E+14

cg13666340	0.00433538	1323.74728	9.48260009	184791.813
cg01441105	0.00433744	19776355.4	191.851883	2.0386E+12
cg13843603	0.00434338	1.7683E+12	6776.0452	4.61E+20
cg05377387	0.00435036	56.6361787	3.53300489	907.911775
cg19301273	0.00435138	84.6632677	4.00592301	1789.31769
cg21379008	0.00436322	137487588	348.954638	5.417E+13
cg21998208	0.00436523	18.5796243	2.49149986	138.552061
cg06835221	0.00437201	3614146.04	111.689376	1.1695E+11
cg00619097	0.0043766	1095626523	664.177389	1.8073E+15
cg13362105	0.00438359	1.9387E+10	1624.09832	2.3141E+17
cg12455762	0.00438375	17.9047997	2.46056147	130.28809
cg06974871	0.00438877	8.35431684	1.93925233	35.9904737
cg11932205	0.00439194	0.082316	0.01476641	0.45887406
cg01861031	0.00439686	308574971	444.080223	2.1442E+14
cg20276962	0.00440167	77.5625292	3.88276667	1549.39672
cg25485294	0.00440305	11.2520626	2.12676628	59.5311834
cg01667978	0.00440709	8568.51261	16.8189428	4365280.82
cg05894734	0.00441075	24.6735838	2.71543156	224.194836
cg04917391	0.00441222	16.1218718	2.37802495	109.29858
cg16391745	0.00442356	16513255.3	176.833256	1.5421E+12
cg23969515	0.00442436	4936696.92	121.388886	2.0077E+11
cg00254079	0.00442448	56696278.7	259.572601	1.2384E+13
cg00716257	0.00443042	26.2211303	2.76429965	248.724003
cg20261082	0.00443603	1441740.77	82.5054058	2.5194E+10
cg04825775	0.00443775	167166833	361.89407	7.7218E+13
cg04413872	0.00444235	278157343	423.363278	1.8275E+14
cg17252669	0.00444395	51846257.6	250.93145	1.0712E+13
cg08617683	0.00444625	14.4620388	2.29523336	91.123878
cg20333292	0.00445383	20.6360592	2.56254163	166.181471
cg22149588	0.00445577	70895.2165	32.1900377	156139355

cg11705470	0.00171666	127636.237	82.0554881	198536495
cg15136410	0.0017215	56.2114168	4.52563102	698.184046
cg06172138	0.0017218	3.1778E+11	20410.8243	4.9475E+18
cg01025836	0.00172224	7.72659039	2.15137822	27.749746
cg24812936	0.00172313	33393983.8	658.850291	1.6926E+12
cg11057378	0.00172382	287744.716	110.948922	746262511
cg19865616	0.00172502	1750.74916	16.4015738	186879.787
cg13440692	0.00172577	31.1320225	3.62497719	267.367979
cg06191571	0.0017328	0.00857748	0.00043684	0.16842177
cg26247263	0.00173666	13885.8475	35.4928824	5432547.25
cg04427651	0.00173892	32.1622136	3.66379018	282.332758
cg26906273	0.00173933	12.8610931	2.60009497	63.6160286
cg05356496	0.00174043	18.3520108	2.96966028	113.4124
cg19769982	0.00174123	6.45877566	2.00923849	20.7619868
cg10851037	0.00174297	1415287.95	199.674392	1.0032E+10
cg04060356	0.00174426	10.6046579	2.4181069	46.5069467
cg00969988	0.00174656	107240.866	75.9749004	151373721
cg24839533	0.00175372	730.371237	11.7459149	45415.1207
cg09479758	0.001756	122.853258	6.03231298	2502.01258
cg16990120	0.00175643	93.3897913	5.44466277	1601.87205
cg01058070	0.00175807	17.9867664	2.94236905	109.953497
cg08826460	0.00176136	4243.36877	22.6187811	796072.009
cg01864807	0.00176154	3771368.11	285.37257	4.9841E+10
cg14374842	0.00176266	597428.388	143.359923	2489682404
cg18686576	0.00176445	40.0914252	3.96608516	405.266731
cg08272286	0.00176469	43.3893736	4.08473494	460.895937
cg12985477	0.0017653	280.553087	8.19761736	9601.57457
cg13656831	0.00176589	21.60913	3.14853339	148.308574
cg22802813	0.00176621	9.01629016	2.2720697	35.779487
cg23892568	0.00176751	14.4426217	2.70854617	77.0115441

cg23755113	0.004462	695164.216	65.3538397	7394413081
cg15058894	0.00446382	19.336085	2.5099434	148.961201
cg07575193	0.00446518	18.9933568	2.49586295	144.538226
cg14896306	0.00447711	1204637887	659.565889	2.2002E+15
cg18511007	0.00448216	12.4085326	2.18505866	70.465697
cg19967492	0.00449157	23.2620003	2.65430422	203.86535
cg22308501	0.00449542	902.347645	8.25272034	98662.1671
cg05125578	0.00449961	14.2909669	2.28115298	89.5300478
cg03919114	0.00450421	14.7572405	2.30348431	94.5420586
cg21834860	0.00451144	457367226	482.568658	4.3348E+14
cg22286382	0.00451803	12.8764242	2.20682069	75.1317494
cg26169020	0.00452172	1.2202E+10	1329.56378	1.1199E+17
cg09820792	0.00452604	32.5990317	2.94115562	361.319496
cg06926254	0.00452635	674730.934	63.7944941	7136381271
cg18948488	0.0045315	30.1448069	2.86982157	316.643163
cg15233961	0.00453269	29.9630801	2.86425736	313.444657
cg23325963	0.00453885	8.57197886	1.94399736	37.7977991
cg13634292	0.00454481	6.27E-12	1.15E-19	0.0003427
cg13211302	0.0045469	112.603082	4.30990807	2941.93144
cg27200446	0.00455388	5.33542597	1.67802943	16.9644047
cg13093774	0.00455575	8.62182748	1.9462763	38.1939138
cg14965353	0.00455861	1425907908	674.821548	3.013E+15
cg22876908	0.0045639	134.674456	4.5487352	3987.30819
cg23124755	0.00457388	196366118	363.847372	1.0598E+14
cg16734497	0.00457639	257659.095	46.8503898	1417025758
cg08949329	0.00457852	15.1128201	2.31255316	98.7641431
cg07560446	0.004585	0.08339847	0.01497136	0.46457413
cg18119621	0.00458548	57401.8576	29.4190782	112001241
cg10927534	0.0045931	6.1148E+15	74082.6325	5.05E+26
cg05861291	0.00459816	20.9028846	2.5535138	171.109546

cg03443162	0.00176788	1196.68976	14.0782419	101721.961
cg22715945	0.00176883	2172.287	17.5818586	268392.034
cg22402261	0.00176968	22.9537555	3.2189989	163.67663
cg10941484	0.00176996	711243739	2006.85856	2.5207E+14
cg16475951	0.0017744	78685.5199	66.9638244	92459041.9
cg18128969	0.00177442	35.5449309	3.78734505	333.595723
cg00188822	0.00177576	6030001.49	337.518035	1.0773E+11
cg00557360	0.00178131	9.05551884	2.27323718	36.0729721
cg00727673	0.00178186	45.4262126	4.14623278	497.69053
cg01916115	0.00178251	10.8854566	2.43441799	48.6741252
cg19868691	0.00178268	31.0530893	3.59786464	268.018521
cg00414306	0.00178409	26437.0098	44.4391476	15727472.8
cg08735211	0.00178467	8.58921323	2.22835167	33.1072446
cg27392771	0.00178486	34.3707623	3.7356175	316.239364
cg22344830	0.00178679	11.5282168	2.48616328	53.455774
cg27060381	0.00178685	130.144193	6.13289136	2761.74973
cg15405796	0.00179148	1206.37168	14.0424122	103638.365
cg27531553	0.00179339	23.7566482	3.25244701	173.524221
cg06038049	0.00179831	7.0794E+10	10907.7629	4.5947E+17
cg22871962	0.00180224	9253834054	5100.29295	1.679E+16
cg01784928	0.00180467	58.1468575	4.53194742	746.049484
cg20744437	0.00180829	446.244264	9.66377892	20606.2188
cg11697159	0.00180888	165364.377	87.1338304	313831919
cg20157398	0.00181674	1105591.85	175.962406	6946559606
cg06761203	0.00181722	46.8312106	4.1748377	525.328755
cg15331996	0.00181967	49.1467143	4.24903812	568.457957
cg14653418	0.00182046	11491.8127	32.2174436	4099076.3
cg07619542	0.00182187	52626.847	56.664604	48876809
cg11376305	0.00182582	3.98638501	1.67093453	9.51040583
cg03356734	0.00182614	16.6889379	2.84316816	97.9613704

cg24550644	0.00460334	5.71276548	1.71134406	19.0702093
cg26393255	0.00460713	10165.1686	17.1848134	6012905.09
cg25423888	0.00460798	1654944.5	82.5610341	3.3174E+10
cg19632842	0.00461146	18433.2005	20.6303755	16470028.8
cg13523649	0.00461306	19.5281254	2.49862399	152.623077
cg18202861	0.00461471	11023.5117	17.5985828	6904977.02
cg04623837	0.00461669	8.51795358	1.93470162	37.5021826
cg27359949	0.00461762	3.06E-06	4.69E-10	0.02001619
cg01822130	0.00462005	7.84418787	1.88598384	32.6255624
cg04111611	0.00462099	6642.24062	15.0413038	2933213.83
cg08042370	0.00462335	61.4804942	3.5552135	1063.18542
cg13520531	0.00462611	38.5287999	3.07815172	482.259667
cg09076334	0.00462777	9.90800415	2.02606356	48.4528463
cg11155414	0.00462839	376969.224	52.0982852	2727648232
cg08309135	0.00462911	0.03731876	0.00383274	0.36336629
cg12518535	0.0046327	39.1257517	3.09149795	495.172396
cg27470087	0.00463508	45.390611	3.23561458	636.759265
cg23814388	0.00463531	3.4536E+14	29807.312	4.00E+24
cg15447662	0.00464086	0.00788309	0.00027575	0.22536209
cg03302259	0.00464185	28.8543734	2.81342875	295.928896
cg20986726	0.0046545	39010404.6	215.684915	7.0557E+12
cg01126162	0.00465607	26.4995489	2.73854843	256.422739
cg24053811	0.00465956	2318.30418	10.8217153	496643.474
cg25794571	0.0046608	372426.144	51.5419553	2691035528
cg18431813	0.00466133	547824.73	58.0255746	5172063139
cg14618996	0.00467265	13.8391554	2.24116017	85.456731
cg06947320	0.00467699	2523788.18	92.43109	6.8911E+10
cg00541565	0.00468389	3492674805	849.619954	1.4358E+16
cg10537421	0.00468774	29.1916281	2.81617252	302.591956
cg00689612	0.00471145	116.706277	4.30062625	3167.06321

cg02938682	0.00182841	33.9374998	3.69931715	311.342296
cg14898768	0.00182938	0.03708008	0.00466991	0.29442351
cg05421149	0.0018299	0.06933661	0.01294355	0.37142562
cg01656048	0.00183377	19490.488	39.0344193	9731901.46
cg11124980	0.00183723	1577.57449	15.3475165	162159.217
cg06449742	0.00184332	336550244	1447.92191	7.8227E+13
cg02029242	0.00184342	0.09551759	0.02178808	0.41874328
cg14855334	0.00184391	8.30665806	2.1917388	31.4821127
cg12905273	0.00184454	23936.2069	41.9782796	13648534.6
cg06924355	0.00184615	2811.15554	18.9711106	416559.454
cg14605021	0.0018464	33.9395722	3.69180399	312.014009
cg25294502	0.00184655	102.820035	5.56688721	1899.07919
cg07971674	0.00184947	1930409.8	213.142411	1.7484E+10
cg25905648	0.00184984	1145574514	2270.00454	5.7812E+14
cg07485357	0.00185488	7.58222236	2.11732957	27.1521717
cg27124109	0.0018579	1788136315	2662.34301	1.201E+15
cg04527260	0.00186018	0.00017623	7.61E-07	0.04079125
cg02989600	0.00186467	73.9739222	4.91509283	1113.33424
cg04299389	0.00186857	7.84500318	2.14227128	28.7284226
cg23124755	0.00186859	42048572.6	660.262901	2.6778E+12
cg08183125	0.00187013	37.5418412	3.82183548	368.773028
cg06067302	0.00187126	111815.519	73.5878695	169901784
cg15705469	0.00187274	15502.5196	35.4245453	6784225.76
cg04918504	0.0018732	12.0044544	2.50637396	57.4961789
cg21091679	0.00187351	1078.48103	13.2201753	87980.78
cg21214613	0.00187547	47.9680527	4.18164021	550.246784
cg24951781	0.00187561	38.5409728	3.85667098	385.152529
cg24250450	0.00187777	4.4683E+16	1423407.86	1.40E+27
cg03871460	0.00187834	16.3816878	2.81037287	95.489
cg16500018	0.00187919	20082489.4	499.509129	8.0741E+11

cg10801634	0.00471158	1.46E+10	1303.73466	1.6349E+17
cg10715223	0.00471281	6.14670139	1.74457032	21.656873
cg07317641	0.00471499	18.6736097	2.45208949	142.206759
cg01437411	0.0047161	67.1614755	3.62945845	1242.79252
cg01583595	0.00471767	0.06446686	0.00962651	0.43172181
cg00372169	0.0047201	118433626	297.322846	4.7176E+13
cg07203561	0.00472382	15.4874473	2.31454046	103.632245
cg14470448	0.00472842	14.5688479	2.27113279	93.4561507
cg16496928	0.00472965	410495.878	52.3224912	3220543636
cg03473294	0.00473356	4.9147E+12	7674.84979	3.15E+21
cg14066993	0.00474166	183.667495	4.92891973	6844.04506
cg06598256	0.00474503	1601644956	654.50031	3.9194E+15
cg07737104	0.00474791	760220.101	62.9285044	9183987561
cg23600701	0.00475119	46.4260289	3.2339579	666.482442
cg07139165	0.0047532	0.1552766	0.0426151	0.56578114
cg02524205	0.00475845	13.670607	2.22440324	84.0160152
cg06796825	0.0047633	30108.3921	23.376927	38778205.4
cg08877188	0.00476423	37.1012605	3.01728417	456.206131
cg09692396	0.00476557	48.4978014	3.27439484	718.311889
cg15038123	0.00476587	8199.92403	15.7029602	4281915.84
cg01030534	0.00476867	4.33418628	1.56529663	12.0010293
cg00036011	0.00476976	5.83120741	1.71376435	19.8411059
cg27591870	0.00477259	10302923.1	138.752067	7.6504E+11
cg11525834	0.00477306	87273.595	32.3032689	235786676
cg02466815	0.00477888	5.8986479	1.71933539	20.2369167
cg18187587	0.00478316	25704484.9	182.907292	3.6123E+12
cg07071389	0.00478413	8.87020629	1.94707775	40.4095624
cg23514016	0.00479522	21.2737951	2.54166809	178.061943
cg14080475	0.00479561	7.56746005	1.85420484	30.8846414
cg15765486	0.00480019	47.0026403	3.23607984	682.692734

cg08008403	0.00188306	18.9507216	2.96450275	121.143368
cg22289434	0.00188982	11.2735063	2.4456924	51.9656288
cg07941017	0.00189225	481335.113	125.147556	1851282583
cg13075322	0.00189408	0.01836204	0.00147413	0.22872023
cg08254307	0.00189413	95.110932	5.37102877	1684.23775
cg19770550	0.00189571	43431886	658.138746	2.8662E+12
cg25204272	0.00189807	864.93809	12.1203583	61724.0747
cg04444465	0.00190018	24913.0621	41.8446797	14832486.8
cg14275576	0.00190224	5321.56956	23.6659119	1196619.96
cg07317641	0.00190235	16.2246379	2.79444198	94.2008733
cg14165186	0.00190249	0.00010358	3.16E-07	0.03392392
cg17906146	0.00190754	136568.92	78.1630569	238617457
cg18120111	0.00191169	908.414517	12.3055418	67060.5931
cg07967717	0.00191322	11.2618108	2.44037961	51.9707603
cg13300282	0.00191467	217.77274	7.26630342	6526.69774
cg01780754	0.00191606	189.753443	6.90524203	5214.35295
cg04330513	0.00191723	54.5560383	4.36222273	682.303839
cg21759784	0.00192189	0.03393027	0.004001	0.28774373
cg22402467	0.00192384	21.3192706	3.08413274	147.370862
cg07392724	0.0019282	119906467	939.275968	1.5307E+13
cg00576301	0.00192933	21185.8326	39.0582187	11491550.7
cg23643471	0.00193093	2335.17143	17.3444728	314395.582
cg26787998	0.00193833	67053.5943	59.492676	75575429
cg15982308	0.0019393	898.782509	12.1853979	66293.2805
cg20252067	0.00193968	780.316329	11.5674965	52638.319
cg21238818	0.0019405	18.4474516	2.91973611	116.55453
cg07276861	0.00194075	13404.9072	32.8928945	5462928.66
cg05783676	0.00194141	6673.65469	25.4497417	1750024.32
cg22198615	0.00194157	28672.2381	43.4873187	18904298.1
cg12404990	0.00194427	543816.424	128.118917	2308295364

cg11050527	0.00480044	71589.6578	30.2533377	169405411
cg01913455	0.00480457	54.178135	3.37842221	868.828741
cg04108127	0.00481026	231851.035	43.2086028	1244078704
cg14364729	0.00481032	3206643997	790.468431	1.3008E+16
cg04617640	0.00481171	16.7555483	2.36130643	118.895369
cg06427502	0.0048132	1971.68352	10.0986509	384955.965
cg01988009	0.00481381	58919228.8	233.501267	1.4867E+13
cg06046912	0.0048163	25.8799416	2.69524274	248.501319
cg02189843	0.00481721	41.1310343	3.10378016	545.065016
cg23265500	0.00482183	12.9408428	2.18156125	76.7640204
cg08790676	0.00482474	8.25226079	1.90193964	35.8054519
cg26881035	0.00482749	6612761.43	119.466088	3.6603E+11
cg18695750	0.00483006	6402.42022	14.4250094	2841660.86
cg03591499	0.00483365	216.227045	5.1391853	9097.57717
cg18384277	0.00483646	199.626422	5.01447947	7947.12763
cg00740389	0.00484369	12.5039836	2.15690201	72.4880424
cg09173265	0.00484378	2.9581E+10	1535.67388	5.6979E+17
cg10982433	0.00484473	14.8280383	2.27163927	96.7894512
cg19137671	0.00484701	670402285	484.576706	9.2749E+14
cg12233363	0.00485263	30.4304144	2.82586644	327.690688
cg01860010	0.0048563	542441175	452.949952	6.4961E+14
cg03169170	0.00486795	342953568	392.533709	2.9964E+14
cg02323652	0.00486927	1.0876E+13	9152.34401	1.29E+22
cg00268086	0.00486934	10.202474	2.02546596	51.3908788
cg11622362	0.00487011	5064.42984	13.3562532	1920332.69
cg02926368	0.00487447	3760.244	12.193714	1159567.54
cg04624228	0.00487535	16.7699361	2.35499533	119.418817
cg15970621	0.00490638	3847.03128	12.2260206	1210504.23
cg14658651	0.00490742	1.1958E+10	1138.02279	1.2566E+17
cg00504134	0.00491004	5409.52225	13.550601	2159530.11

cg02168442	0.0019446	25.5385381	3.28919428	198.290789
cg22303758	0.00194685	0.00955175	0.00050381	0.18109265
cg02957185	0.00194724	9.87220622	2.31913775	42.0244359
cg01555036	0.00195015	1232671974	2182.54579	6.962E+14
cg15987431	0.00195292	12.9463929	2.56084739	65.4506351
cg25151638	0.00195314	1023233.8	161.003721	6503001285
cg10321263	0.00195665	0.02420677	0.00229673	0.25513173
cg08038761	0.00195699	235981.806	93.8117154	593608299
cg18327128	0.00195842	0.11565478	0.02952407	0.45305502
cg11168687	0.00196148	14.8292977	2.68985053	81.7547547
cg05766064	0.0019618	8.49792404	2.19275682	32.9332977
cg13912673	0.00196193	6370.41398	24.8793306	1631160.22
cg00955482	0.00196325	74759.5272	61.3853093	91047629.5
cg21159993	0.0019657	44.1116291	4.01082655	485.145842
cg25522149	0.00197199	164.886894	6.49904786	4183.33397
cg03097541	0.00197934	2.8748E+12	36700.4241	2.25E+20
cg20772300	0.0019851	640.837285	10.6625415	38515.4351
cg03665761	0.00198872	19.6132792	2.97305048	129.389233
cg07086112	0.00199329	17128.6359	35.4301576	8280803.37
cg08417561	0.00199442	0.06936424	0.01277407	0.37665364
cg23792245	0.00199628	787435311	1798.23644	3.4481E+14
cg07112260	0.00200087	152.315295	6.28480315	3691.43608
cg22721796	0.00200503	766.491667	11.3391736	51812.3716
cg22492252	0.00200599	31229988.3	549.30198	1.7755E+12
cg03629151	0.00200779	20.776538	3.03107618	142.41296
cg19743881	0.00201335	398520.956	111.204143	1428174776
cg01305328	0.00201878	8471666721	4222.23624	1.6998E+16
cg02862835	0.00201952	12807.2139	31.614564	5188264.76
cg24910161	0.00202074	36.1933983	3.70762017	353.316149
cg13326926	0.00202087	1477.38867	14.3631845	151963.325

cg23227945	0.00491118	2473185.83	86.7816226	7.0483E+10
cg17633033	0.00491374	80093585.4	248.904911	2.5773E+13
cg06243556	0.00491635	30.0553661	2.80530704	322.005761
cg08417719	0.0049194	0.11008423	0.02365214	0.51236548
cg26900458	0.0049217	1763.79188	9.63309268	322945.28
cg01038149	0.00493732	13.0215374	2.17516719	77.952829
cg03731862	0.00494682	31075179.6	185.084618	5.2174E+12
cg01045986	0.0049501	230268.924	41.922525	1264804001
cg19544662	0.00495243	8.89422853	1.93702519	40.8395831
cg10858568	0.00495378	13042.8868	17.5768642	9678455.41
cg06615378	0.00495518	216901939	332.363532	1.4155E+14
cg25410636	0.00495909	713217.003	58.9056185	8635483436
cg16083849	0.00496046	144697.133	36.3525493	575950259
cg03432464	0.00496174	0.06993959	0.01093404	0.4473686
cg11514288	0.00496247	15.5569299	2.29298853	105.547004
cg14775474	0.00496624	163182.518	37.6564078	707144831
cg18642271	0.00497094	133837.189	35.4342162	505511198
cg16270051	0.00497913	0.07560559	0.01247093	0.4583625
cg16066354	0.00498414	27.2863767	2.71447795	274.287125
cg19872463	0.0049913	8.91821973	1.93595417	41.0829162
cg23133255	0.00499388	32.5137072	2.8604866	369.566897
cg16113047	0.00499505	12634.3446	17.2996911	9227139.57
cg18501647	0.00500256	127.087511	4.31382062	3744.06744
cg14424070	0.00500353	9.42243757	1.96750159	45.1244006
cg15945287	0.00500637	14.4114566	2.23635852	92.8697603
cg11199172	0.00501631	34.7988729	2.91602233	415.27856
cg14693391	0.00502144	13.02151	2.16763113	78.2235135
cg09639151	0.00502228	24.027541	2.60711416	221.441291
cg17157872	0.00502411	21.0998143	2.5067595	177.600669
cg07469555	0.00502544	67.3475834	3.55617365	1275.44306

cg03872783	0.0020209	8.24000158	2.15985197	31.4362406
cg16225168	0.00202485	115.581444	5.66182385	2359.49945
cg08568550	0.0020267	9.82198636	2.30196398	41.9083083
cg25335229	0.00202759	6994.01653	25.2936326	1933936.02
cg21844656	0.00202812	0.06822907	0.01240013	0.37541584
cg01169778	0.00202997	202.941445	6.94762408	5927.95892
cg05021798	0.00203309	232.896041	7.30178322	7428.39991
cg10256686	0.00203653	247851.226	92.6800101	662820711
cg06359167	0.00203912	2337022.14	209.812172	2.6031E+10
cg09541379	0.00203941	178.653707	6.62215286	4819.75392
cg13544006	0.00204944	8.49997352	2.18047555	33.1347673
cg18933494	0.00205534	71.9331643	4.74329609	1090.8828
cg21531300	0.0020591	13.4441461	2.57487013	70.195798
cg03467067	0.0020595	32570821.8	542.470882	1.9556E+12
cg03887520	0.00206049	9.70585355	2.2867178	41.1959855
cg21954542	0.00206156	0.00367604	0.00010392	0.13002984
cg24120357	0.00206698	21.8596262	3.0709953	155.598824
cg17169566	0.00206796	136300366	909.050469	2.0436E+13
cg22126440	0.00207119	18.4373705	2.88552128	117.807702
cg20263444	0.0020729	678994.625	131.900002	3495327483
cg25114752	0.00207635	0.09115673	0.01984506	0.41872129
cg16385703	0.00207692	879568.253	144.677591	5347340273
cg24965497	0.00207707	2.07E-05	2.15E-08	0.01982878
cg07007550	0.00208317	15615.5679	33.3688783	7307586.42
cg19285119	0.00208371	601867.457	125.705959	2881680683
cg21956537	0.00208728	901359.108	145.35782	5589298481
cg22888463	0.00208898	97.5936017	5.27631551	1805.14434
cg14543527	0.0020893	529316498	1470.19365	1.9057E+14
cg18664712	0.00209309	39878.108	46.7631989	34006730.5
cg22997194	0.0020936	762311.004	136.432385	4259385100

cg16944093	0.00502548	42.1023578	3.08673747	574.266049
cg19872095	0.00502632	65.208849	3.52154794	1207.47866
cg22030047	0.00502846	78.9657091	3.73009811	1671.69416
cg06090684	0.00502971	34.8912384	2.91613478	417.469908
cg15844687	0.00503127	1.4936E+15	37281.2046	5.98E+25
cg25401594	0.00504042	0.10792598	0.02277183	0.51151
cg04793272	0.0050419	67.7848084	3.55917237	1290.96873
cg01774894	0.00505183	5.43126887	1.66402777	17.7272773
cg07394446	0.00505495	11.1030512	2.06330145	59.7478111
cg15442444	0.00505934	41739.4985	24.544337	70981169.3
cg00994394	0.00506023	70565.2797	28.7397446	173260367
cg19378640	0.00506056	9.60477535	1.97484253	46.7134509
cg18529840	0.00506181	0.08134859	0.0140749	0.47016961
cg09415957	0.00506221	56.0526498	3.3568532	935.96573
cg13441378	0.005069	26.417614	2.6761799	260.778555
cg07145284	0.00506901	10.5047135	2.02812919	54.4092589
cg07717381	0.00506932	5937.74929	13.6326912	2586200.05
cg05963821	0.00507197	24.3071639	2.60962467	226.407354
cg06391932	0.00507322	29.2231676	2.75801451	309.640692
cg00388812	0.00507347	65.1367019	3.50935712	1208.9935
cg00188822	0.00507614	3998292.19	96.4301529	1.6578E+11
cg21490342	0.00508182	324435.886	45.2781799	2324710141
cg24687970	0.00508265	11.9473267	2.10700499	67.744792
cg27064845	0.00509239	203.097792	4.93166263	8364.05814
cg04247218	0.00510286	6.4139E+10	1752.0229	2.348E+18
cg24089133	0.00510651	52.9596657	3.29084906	852.280412
cg07244253	0.00511968	1367.77191	8.71722153	214609.664
cg22402261	0.00512003	31.3246242	2.80901765	349.315029
cg04304033	0.0051204	3332476.27	90.338034	1.2293E+11
cg00731944	0.00513339	0.10206966	0.02064313	0.50468194

cg06834005	0.00209987	12.7472292	2.51771103	64.5395163
cg03214395	0.00210215	55079.5663	52.4260929	57867341.5
cg09201719	0.00210529	29.0571096	3.39299798	248.84059
cg04040095	0.00210796	97.5467952	5.26191501	1808.34872
cg27401681	0.00211024	1.3466E+14	132215.886	1.37E+23
cg08343943	0.00211076	630754.79	126.511573	3144784270
cg07444414	0.00211208	38.1338274	3.74176826	388.636787
cg15726532	0.00211656	62378.5226	54.5912626	71276609.1
cg18020334	0.00212556	53.4530563	4.22228499	676.702126
cg00863378	0.0021256	29.927154	3.42257058	261.684756
cg19596493	0.00212663	5774.37782	22.9929465	1450159.47
cg17253939	0.00212819	4.2923E+11	16228.7789	1.1352E+19
cg10648455	0.00212902	0.00159585	2.62E-05	0.09721427
cg15193461	0.00213054	87.0225492	5.03372064	1504.43869
cg10013356	0.00213058	1185.39074	12.9516985	108491.656
cg05436939	0.00213453	76.2440231	4.79611941	1212.05303
cg20495332	0.00213658	2711.92989	17.4496866	421472.538
cg09270822	0.0021461	9.02E-10	1.51E-15	0.00053836
cg09360748	0.00214685	7599214.17	306.66623	1.8831E+11
cg07207099	0.00214709	5.86099532	1.89467477	18.1304288
cg14515252	0.00215162	85617.901	60.5224773	121119050
cg26694487	0.00215586	535674.866	117.191713	2448531163
cg23528791	0.00215665	19.704446	2.93413822	132.326824
cg16761581	0.00216363	6.08899864	1.9193312	19.3170957
cg00536924	0.00216519	14.1231876	2.59997934	76.7176972
cg22467567	0.00216521	14.5094446	2.62541325	80.1869892
cg01268763	0.00216687	65.2003179	4.51450504	941.649509
cg15826897	0.00217207	9.05772904	2.21393541	37.0572941
cg13676119	0.00217294	20617.9105	35.9663069	11819346.2
cg24745816	0.0021772	9.53902325	2.25491564	40.3531569

cg11270393	0.00513426	7.67679665	1.84174924	31.9984966
cg01395659	0.00513491	1655.5014	9.21444901	297433.4
cg17091967	0.00513722	56.8979787	3.35587378	964.690628
cg13383058	0.00514522	13.3373698	2.1722799	81.8888175
cg19195724	0.00514748	0.03199686	0.00286963	0.35677101
cg19403104	0.00515214	6.21899166	1.72823489	22.3788198
cg03232620	0.00515626	7.07006434	1.79565421	27.8371022
cg06485940	0.0051654	18.9263355	2.41006294	148.629386
cg25792651	0.00517222	20.1324353	2.45422864	165.14963
cg19664945	0.00517657	8.60143511	1.90286909	38.8805969
cg05951221	0.0051778	22.8908976	2.54960374	205.519463
cg03120789	0.00519429	18686.4922	18.8745261	18500331.5
cg01348004	0.00519712	25.4311378	2.62851855	246.048395
cg17532444	0.00519933	7925.59393	14.5984749	4302849.41
cg17519645	0.00521185	12.3853151	2.11906592	72.3885123
cg00169633	0.00521333	3.5577E+10	1407.43511	8.9931E+17
cg23813257	0.00521908	578.091808	6.66686577	50127.0238
cg05651282	0.0052201	29.080864	2.73262748	309.481134
cg19241779	0.00522501	5065921.94	99.8676798	2.5698E+11
cg25412979	0.00523151	66.1158964	3.48864783	1253.01033
cg12742785	0.00523804	4429471.58	95.6513321	2.0512E+11
cg15518400	0.00524252	2278.81009	10.0092844	518815.854
cg26257814	0.00524282	6.14078945	1.71728168	21.9587127
cg24839533	0.00524294	556.085544	6.57469696	47033.5187
cg00352195	0.00525889	4.4624E+11	2937.23859	6.7794E+19
cg25587223	0.00526099	10.9134337	2.03683256	58.4746323
cg00489699	0.00526513	413962200	366.721419	4.6729E+14
cg06086785	0.00526862	1313974.48	66.1470441	2.6101E+10
cg23302845	0.00527533	292867266	329.818454	2.6006E+14
cg02297043	0.00527537	35.4673624	2.89048624	435.197989

cg00221357	0.00217766	12512080.9	361.936636	4.3254E+11	cg03177847	0.00527592	14.480278	2.21433823	94.6912476
cg03340356	0.00218581	14.6836329	2.63256183	81.9008588	cg22866937	0.00527786	32.7571063	2.8225659	380.160482
cg12487497	0.00218717	4756157989	3062.51641	7.3864E+15	cg16399136	0.00527829	5.92127961	1.69709934	20.6596935
cg15522728	0.00219368	331125.007	97.1547793	1128547363	cg18023065	0.00527905	0.13753571	0.0341229	0.55435122
cg04693399	0.00219447	2159.027	15.8642224	293830.825	cg10552964	0.00528	0.01652888	0.00092536	0.29523903
cg20370281	0.00219518	15.7518274	2.69802475	91.9635992	cg22517351	0.00528287	3.0455E+10	1309.11061	7.0852E+17
cg14638988	0.00219877	26.1300236	3.23618521	210.982403	cg23650853	0.0052871	1480872.85	68.2652189	3.2124E+10
cg05951994	0.0022007	12.5458742	2.48481395	63.3443637	cg05423529	0.00528956	19.6432733	2.42304572	159.245112
cg05952925	0.00220314	8.84715002	2.19098879	35.7245386	cg21103829	0.00529584	6606486.68	106.250698	4.1078E+11
cg03707974	0.00220325	23.7638435	3.12623523	180.639081	cg14986464	0.00529984	26.1965683	2.63813867	260.130446
cg24903183	0.00220455	5.47188376	1.84304831	16.2456468	cg21084284	0.00530047	27.2619127	2.66947714	278.411032
cg02259384	0.00220559	11.9997416	2.44449134	58.9054241	cg00087884	0.00530138	63.7284961	3.4351416	1182.28641
cg17668148	0.00220877	18672.302	34.356159	10148249.2	cg04739149	0.00530295	1.0814E+10	955.371667	1.2241E+17
cg02847037	0.00221229	13843.7916	30.8220391	6217971.7	cg08491668	0.00530359	2173.41485	9.79770966	482126.156
cg03762702	0.00221493	93280.4429	61.145601	142303631	cg09388058	0.00530635	842801.466	57.5010807	1.2353E+10
cg01481989	0.00221578	8.28409546	2.1381408	32.09622	cg26454350	0.00531105	261362.299	40.577749	1683441126
cg15442444	0.00221674	27301.8027	39.2950908	18968996.3	cg03087607	0.00531261	17.694655	2.34656875	133.42921
cg04529955	0.00221677	1.0756E+10	4030.00007	2.8709E+16	cg15586420	0.00531349	769.838248	7.19142197	82410.8127
cg12360736	0.00221767	9.02110381	2.20439226	36.917347	cg27639662	0.00531741	37.5732543	2.93359922	481.234597
cg01294490	0.00222574	110.198301	5.41283887	2243.49288	cg10573476	0.00531798	7.73123154	1.83489531	32.5751233
cg07502066	0.00222667	304142.796	93.12036	993368584	cg19431506	0.00533365	1823.4309	9.26767125	358763.293
cg09859398	0.00222835	7.76213226	2.08718824	28.8669207	cg25225632	0.00533681	993698670	465.127982	2.1229E+15
cg20750843	0.00223098	9893773.34	324.546141	3.0161E+11	cg11105830	0.00533841	15439.5297	17.4491483	13661358.9
cg12272369	0.00223324	1588.97702	14.0919192	179169.916	cg11346237	0.00534098	272.131305	5.26896444	14055.0289
cg03980541	0.00224339	5.55919804	1.85009146	16.7044082	cg20747741	0.0053455	645.441347	6.8031869	61235.2031
cg19646112	0.0022498	9.56080569	2.24631765	40.6928225	cg07362258	0.00534745	36902614.6	174.703879	7.7949E+12
cg19785066	0.00225406	5.91185381	1.89035138	18.4886344	cg20275528	0.0053529	5.86651836	1.68898443	20.3767643
cg01553230	0.00225525	1.33E-05	9.87E-09	0.01789387	cg02070114	0.00535944	21260664.2	147.90872	3.056E+12
cg10573476	0.00225858	6.70764343	1.97738838	22.7534868	cg22799396	0.00535962	20.8453926	2.45815331	176.77107
cg15742848	0.00225957	6.85325925	1.99255344	23.5713439	cg09718046	0.00536882	128424.741	32.5197054	507166778

cg10282807	0.00226403	9.85127911	2.26848332	42.7808745
cg15728769	0.00226807	5605.4524	21.9698451	1430192
cg18731976	0.00227625	591.39483	9.80780726	35660.1466
cg17091967	0.00227945	43.9950768	3.8702789	500.110413
cg17034360	0.00228136	7183.37229	23.9310458	2156229.94
cg13606421	0.00228664	26.4892637	3.22596132	217.510695
cg06727703	0.00229666	82.8424021	4.84312164	1417.03308
cg02396009	0.00230295	5643209.47	257.165905	1.2383E+11
cg02335251	0.00230389	9.4922114	2.23297112	40.350758
cg20577613	0.00230549	1.4249E+11	9571.75703	2.1213E+18
cg09540111	0.00230565	695.160978	10.3366256	46751.1164
cg21496913	0.00230895	16749.3399	32.1528203	8725218.67
cg08864344	0.00230914	9.04424109	2.19406952	37.2815429
cg13338827	0.00231434	10.1799076	2.28789859	45.295066
cg09992998	0.0023191	34396499.1	486.520172	2.4318E+12
cg24631877	0.0023225	46.3728809	3.9259271	547.75446
cg20028470	0.00232363	28.7708173	3.31131317	249.979354
cg21883802	0.00233256	22.9810559	3.05413319	172.922691
cg12416569	0.00233407	14.5822316	2.59704219	81.8783305
cg23601397	0.00233823	41.1683969	3.75681973	451.136073
cg00263248	0.00233846	37.3434668	3.62855148	384.322648
cg18486906	0.00234256	16.4223934	2.70756583	99.6079222
cg14611892	0.0023475	46404.6834	45.7321894	47087066.5
cg00821731	0.00234797	22.7253558	3.03800496	169.993731
cg18299211	0.00234816	3143.15788	17.5466855	563037.472
cg09559971	0.00234933	173282.393	73.0351103	411128123
cg15049402	0.00235169	17.8564836	2.78746369	114.388578
cg18202751	0.00235275	106943.757	61.4478688	186124716
cg26796815	0.00235435	4601.39956	20.0662064	1055151.01
cg08655760	0.00235778	681.282283	10.1677094	45648.9785

cg23042086	0.00536949	221.048342	4.94239949	9886.36588
cg03467067	0.00538026	92116415.5	227.002955	3.738E+13
cg26541027	0.00538037	2278589.1	75.9855594	6.8328E+10
cg04981940	0.00538229	21.4428033	2.47617874	185.686843
cg17266233	0.00538783	28.769691	2.70033533	306.515681
cg00142642	0.00539127	69.5530084	3.50501243	1380.2008
cg22249338	0.00540196	34865.035	21.9907591	55276430.6
cg14138132	0.00540911	7053358884	811.059643	6.1339E+16
cg03460682	0.00541424	33.7765083	2.82753666	403.4793
cg10608717	0.00541642	9576819397	885.478668	1.0358E+17
cg04816394	0.00541919	16.7419673	2.29776035	121.98551
cg02987906	0.00543477	0.06813316	0.01025343	0.45273889
cg15066337	0.0054351	12.8780364	2.12516849	78.0379639
cg25975823	0.00543732	4217853.16	89.9716354	1.9773E+11
cg01294023	0.00544223	2571744615	595.520146	1.1106E+16
cg10832938	0.0054453	46.7679539	3.10712187	703.94455
cg17095910	0.00545553	4103.76862	11.6073244	1450887.06
cg05789714	0.00546346	55.4722885	3.26388665	942.794623
cg02852873	0.00546487	25801.502	19.9250828	33411028.3
cg09176275	0.00546735	33.4573374	2.81167226	398.123722
cg13581941	0.00547443	481669.388	47.0944973	4926380199
cg05816157	0.00548477	36.5873759	2.88398093	464.162595
cg01692674	0.00548584	21604.0594	18.8502786	24760131.8
cg26081613	0.00548697	196084206	275.221254	1.397E+14
cg10614773	0.00548772	279309.568	40.0135674	1949684569
cg02601318	0.00549544	441058.938	45.7007067	4256673489
cg06553210	0.00549664	14829.6128	16.84856	13052594.3
cg09209002	0.00549865	19324.5411	18.2072257	20510422.3
cg23740975	0.005504	0.04375873	0.00480383	0.39860411
cg12439852	0.00550625	11.4024902	2.04488435	63.5814847

cg21665738	0.00236128	15.474255	2.64717844	90.4557714
cg05682719	0.00236199	16.7336691	2.72167673	102.88352
cg15145873	0.00236318	45.5989596	3.88599213	535.066734
cg25947283	0.00236804	317.966731	7.74249826	13058.1679
cg11282402	0.00237074	1.7618E+11	9860.60227	3.1478E+18
cg25362068	0.00237313	43921.7	44.5151278	43336183.2
cg08026651	0.00237419	9.59015044	2.23149288	41.2150028
cg13522186	0.00237794	5185.98149	20.8242456	1291494.76
cg13222081	0.00237936	475.01624	8.91226455	25317.968
cg10602180	0.00238239	12.9531471	2.48142681	67.6159455
cg15893346	0.00238274	1252.45684	12.5644411	124848.221
cg06383048	0.00239224	85155.2623	55.9852322	129523777
cg14089714	0.00239278	10.9099531	2.33323813	51.0136855
cg05162137	0.00239515	8.368E+16	997297.583	7.02E+27
cg15345154	0.0023988	11.4583453	2.37323056	55.3227648
cg01090026	0.00240584	9.03086242	2.18031527	37.4058179
cg18158177	0.00241004	3.0532E+12	26345.1871	3.54E+20
cg03507534	0.00241146	17.6802809	2.7648629	113.05889
cg13397067	0.00241206	825106.042	124.350147	5474862672
cg03403116	0.00241277	5643141.91	245.551436	1.2969E+11
cg18123399	0.00241836	221404533	897.599574	5.4612E+13
cg20028233	0.00241845	28838.9516	37.8640847	21965013.4
cg05403387	0.00241863	824.645252	10.7633809	63180.8723
cg26060255	0.002423	67.6152703	4.43982939	1029.72983
cg17106175	0.0024275	19.5423487	2.86101779	133.485151
cg01610602	0.00242773	133056638	746.115064	2.3728E+13
cg02156680	0.00242903	9.69422363	2.23261927	42.0931473
cg12824106	0.0024295	604949.665	110.72558	3305145004
cg18113295	0.00243407	7.11523843	2.00081226	25.3030326
cg17792849	0.00243455	188.920008	6.3756095	5598.01683

cg26898605	0.00551223	38.3480118	2.91974275	503.664238
cg17469276	0.00551347	28.9982576	2.68939992	312.671587
cg05817661	0.00551434	1.1496E+11	1776.34063	7.4401E+18
cg10660256	0.00553314	39.2143605	2.93559324	523.834857
cg26175971	0.00553341	40.2855429	2.95886167	548.496398
cg07229186	0.00553428	6.16808595	1.70571074	22.3046518
cg14022778	0.00553819	8.53237581	1.87590235	38.8087561
cg09877947	0.00553848	20.1829779	2.41504384	168.672961
cg15030629	0.00554293	1.104E+10	883.621762	1.3794E+17
cg18021716	0.00555955	9.83732445	1.95447308	49.5135766
cg25258291	0.00556055	1720108212	509.290848	5.8096E+15
cg10014563	0.00556942	28.6567089	2.6725394	307.275907
cg23033906	0.00557371	8.09647345	1.84520966	35.5259805
cg10879207	0.00557692	14.9458346	2.20784398	101.174709
cg02636234	0.0055799	1.4962E+12	3672.88294	6.09E+20
cg06604199	0.00558285	19.168809	2.374137	154.76918
cg18343556	0.00558444	121.879746	4.07990052	3640.93985
cg27625401	0.00558568	114.285606	4.00342899	3262.50317
cg03743720	0.00559587	71.5611566	3.48847305	1467.97727
cg00373148	0.00560077	146.252399	4.29834477	4976.27929
cg12872647	0.00560708	77.87768	3.5732932	1697.2951
cg24868790	0.00560871	36.9803489	2.87377506	475.870998
cg12506775	0.00561407	6.20978975	1.70538182	22.6116453
cg23849812	0.0056156	0.10158634	0.02013523	0.5125237
cg03980541	0.00562052	5.62527153	1.6565332	19.1023517
cg18334142	0.00562367	1734916.25	66.5110983	4.5255E+10
cg10793756	0.00562454	1475169278	477.188556	4.5603E+15
cg03450370	0.00562709	111.362611	3.96168989	3130.38916
cg11343211	0.00563506	6.36335349	1.71661476	23.5884419
cg09841537	0.00563794	250595.36	37.691847	1666090666

cg04608177	0.0024383	9.49182211	2.21480278	40.6784241
cg25345578	0.00243853	0.07269311	0.01334312	0.39603093
cg17470143	0.00244131	21.1057885	2.93667837	151.686448
cg19865525	0.00244647	191.824189	6.39947781	5749.92532
cg10786226	0.00244792	0.0497852	0.00714882	0.34671004
cg06635722	0.0024497	13.1769185	2.48505734	69.8700903
cg20066737	0.00245207	0.03203317	0.00345689	0.29683429
cg17960769	0.00245506	2087.82829	14.8425622	293684.266
cg09560599	0.00245548	15951.4214	30.4163569	8365493.76
cg03856286	0.00245631	164164.634	69.2280696	389293351
cg01974375	0.00245912	45.4945251	3.84513156	538.278542
cg00154846	0.00245934	27.5639825	3.22203069	235.805677
cg06830503	0.00246284	123558.273	62.4976607	244275494
cg00260114	0.00246303	16473.3085	30.7046898	8838060.08
cg17691657	0.00246402	468.392572	8.74622905	25084.1363
cg15436096	0.0024641	7.75626449	2.0594163	29.2119854
cg25490585	0.00246434	2547375518	2075.31861	3.1268E+15
cg00152259	0.00246542	126.640139	5.51338514	2908.87078
cg26527984	0.00247121	46.3306427	3.86521435	555.345254
cg08680689	0.00247226	71284692.3	585.804846	8.6744E+12
cg20342628	0.00247284	70.3293918	4.47699851	1104.8079
cg09643398	0.0024752	28.7645624	3.26629591	253.314481
cg00362285	0.00247665	16.5167431	2.68603665	101.563322
cg14553243	0.00247951	13.6297886	2.50974994	74.0197791
cg07076277	0.00248061	206.796424	6.54029241	6538.66192
cg23748035	0.00248134	82.016002	4.72161809	1424.64394
cg01663570	0.00248273	557366.267	105.591522	2942065320
cg03525527	0.00248483	5.1796E+15	341407.577	7.86E+25
cg06888121	0.00248748	8.67540713	2.13955307	35.1768274
cg01313358	0.00248889	60.960407	4.24968809	874.457406

cg11475922	0.00564691	646830.14	49.6258643	8430870400
cg20568322	0.00564695	315.421958	5.36076463	18559.1084
cg07107420	0.00565642	14.4134113	2.17765621	95.399092
cg15852352	0.00565983	0.14147318	0.03540275	0.56534199
cg08009622	0.00566563	11.8959383	2.058384	68.7497314
cg07105272	0.0056659	16.7868744	2.27576945	123.825879
cg20240860	0.00567248	858.536551	7.16184341	102918.336
cg15528052	0.00567638	55189.4336	24.0830094	126473961
cg07382273	0.00567928	152301694	242.090283	9.5815E+13
cg19808978	0.00568315	6.79216962	1.74724114	26.4036641
cg01350077	0.00568512	5.87419227	1.67479583	20.6031889
cg15670863	0.00568753	3169.6306	10.4609479	960386.982
cg14656297	0.00570026	15289.7379	16.5122895	14157702.6
cg26433208	0.005704	549697.204	46.8017753	6456315262
cg12649455	0.00570594	11.6850756	2.04469834	66.778062
cg08548478	0.00571128	18215.6012	17.3480144	19126576.7
cg06822966	0.00571632	1127683.64	57.5404997	2.21E+10
cg17480669	0.00572891	1085.40558	7.62411113	154523.622
cg10857203	0.00573184	135495.42	30.988164	592452291
cg18506672	0.00574005	28.1957851	2.63760283	301.41092
cg17129986	0.00574299	11.2371769	2.01892778	62.5451521
cg21476824	0.00574458	69020.5442	25.4198469	187406145
cg16776006	0.00574461	645315.549	48.6491474	8559906601
cg26918756	0.0057474	26.7473155	2.59659202	275.522254
cg14855334	0.00575497	8.49647906	1.86078985	38.7954375
cg26283550	0.00575522	941348.819	54.1711	1.6358E+10
cg07087293	0.00575608	27.7444052	2.62322863	293.436878
cg07037112	0.0057567	18.4042874	2.32858166	145.460991
cg26873311	0.00576148	272470323	280.169414	2.6498E+14
cg15916004	0.00576161	17.6256512	2.29907864	135.125252

cg06787764	0.00249244	8.87335101	2.15600671	36.5195329
cg00609948	0.00249297	292.599323	7.37724561	11605.1936
cg17526770	0.00249488	30.8971869	3.34376774	285.497149
cg13258989	0.00249667	8696.034	24.3158259	3109950.19
cg26839512	0.00249812	9.98099677	2.2463673	44.3472876
cg23955930	0.00249867	44811522.5	491.394762	4.0865E+12
cg14399447	0.00249981	5198755409	2614.26919	1.0338E+16
cg16849024	0.00249988	11.7244601	2.37699059	57.8306724
cg02102829	0.0024999	55169550.4	528.384069	5.7604E+12
cg02002664	0.00250264	3534515.47	200.791944	6.2218E+10
cg24577137	0.00250646	3375.80745	17.3947882	655143.124
cg02801839	0.00251269	301379.631	84.202851	1078700793
cg16721177	0.00251888	6.62515014	1.94281639	22.5922607
cg07740599	0.00251982	0.01810113	0.00134067	0.24439329
cg09577511	0.00251985	10.8874054	2.3130082	51.2473743
cg05221455	0.00252031	9.67348388	2.21887986	42.1727612
cg09262011	0.00252124	395.075966	8.16364652	19119.5219
cg12819417	0.00252531	139.083949	5.65504741	3420.72194
cg13631572	0.00252604	4.73192409	1.72572465	12.9749005
cg05972308	0.00252618	5.80E-08	1.17E-12	0.00288147
cg16306898	0.00252691	4.92991171	1.75067317	13.8826766
cg16797831	0.00253187	9.2866216	2.18585082	39.4543579
cg24053811	0.00253378	905.754432	10.9010229	75258.175
cg01940297	0.00253492	8.31295198	2.10217645	32.8731541
cg16553500	0.00253774	16.2518869	2.65902517	99.3310742
cg14071579	0.00253789	7.4940721	2.02676106	27.7097866
cg27123975	0.00254252	64367.966	48.5336197	85368350.3
cg09357934	0.0025449	31.8570796	3.36488986	301.606757
cg03418154	0.00254496	76649.9517	51.5620761	113944502
cg21349401	0.00254534	100577.825	56.7080079	178385720

cg20557104	0.00576498	9.43641355	1.91769938	46.4337119
cg01184452	0.0057675	28.4126907	2.63994123	305.795063
cg14383422	0.00576917	6.41540483	1.7144203	24.0066098
cg02155398	0.00577265	9.67609322	1.93121365	48.4807986
cg06028808	0.00577869	132.164227	4.11991083	4239.74782
cg10210369	0.0057828	9.92816161	1.9450102	50.6775712
cg24688655	0.00578729	16.1456346	2.23898425	116.428472
cg21872782	0.00579303	24.4413628	2.52413871	236.666952
cg26419426	0.0057936	9887207.64	106.238056	9.2017E+11
cg03602233	0.00579686	20.7281977	2.4060759	178.572164
cg08067677	0.0057978	5963440.19	91.6772878	3.8791E+11
cg09503045	0.00580009	10.6581664	1.98424169	57.2493314
cg17777874	0.00580355	48606871.5	168.079558	1.4057E+13
cg10099601	0.00580959	15.2355621	2.19971858	105.523659
cg10119082	0.00581135	7.40533855	1.78508697	30.7206538
cg05446860	0.00581601	8.07522831	1.83013451	35.630885
cg10939691	0.0058242	32.9085267	2.74694264	394.245993
cg23039189	0.00582496	16.2441635	2.23952286	117.825477
cg24949907	0.00583132	0.08445832	0.0145754	0.48940056
cg09308608	0.0058325	4.261E+11	2302.46823	7.8854E+19
cg02851517	0.00583286	1057403.94	55.1607807	2.027E+10
cg15994744	0.00583399	50.3514368	3.10477597	816.570089
cg15080119	0.00583677	6.25428988	1.69876393	23.0262377
cg06890747	0.00583859	18.9526251	2.34030853	153.48489
cg25507767	0.00584604	29.5670533	2.66033358	328.609409
cg13354228	0.00584608	1.49E-17	1.61E-29	1.37E-05
cg19006008	0.00584902	7.69576382	1.80306692	32.8466903
cg15811902	0.00586063	8132.41628	13.4552462	4915271.99
cg17559110	0.00586594	10716428.1	106.919978	1.0741E+12
cg26779378	0.00586874	14.0185051	2.14250648	91.723636

cg23305408	0.00254583	12.625793	2.43240601	65.5362009
cg16391745	0.00254757	1248463.18	137.011525	1.1376E+10
cg26143525	0.00255222	1270864736	1548.34999	1.0431E+15
cg24398023	0.00255252	15405.4516	29.3247177	8093102.23
cg02323652	0.00255629	1.1734E+12	16889.0205	8.1523E+19
cg25163611	0.00255641	18.668906	2.78763055	125.02663
cg22970435	0.00255821	17.323326	2.71518094	110.525828
cg21152671	0.00255884	15.6103962	2.61784206	93.0860086
cg17969560	0.00255941	23.8571527	3.0369192	187.414842
cg03357260	0.00256781	542493.963	101.606186	2896474226
cg17221444	0.00257162	226.26675	6.66561939	7680.70291
cg07154254	0.00257305	2173.64747	14.70629	321273.642
cg14539086	0.00257324	0.00299777	6.86E-05	0.13100239
cg14849798	0.00257414	7.46697634	2.02042207	27.5960833
cg25016127	0.00258046	1.0562E+10	3198.07749	3.4885E+16
cg18410627	0.00258344	48584.1054	43.482664	54284054.3
cg04558861	0.00258431	121.823188	5.35905874	2769.30892
cg15433343	0.00258754	4531276.73	211.936291	9.688E+10
cg26067203	0.00258834	5.67140177	1.83393235	17.5387048
cg19678561	0.00258855	1407502.96	140.797213	1.407E+10
cg00264591	0.00258939	9.20184741	2.17174296	38.9889582
cg07616871	0.00259101	29.5453149	3.26418651	267.42517
cg24546463	0.00259494	23.9527256	3.03247094	189.196558
cg13166888	0.00259705	17.0775187	2.69407478	108.252988
cg19502671	0.00260686	0.0033377	8.15E-05	0.13668054
cg13451356	0.00261145	44.5347291	3.76001725	527.48218
cg09108404	0.00261342	6343824.53	235.957362	1.7056E+11
cg12291552	0.00261479	4938.74767	19.4222904	1255836.88
cg21760146	0.00261596	20.8317078	2.8835039	150.497473
cg27062369	0.00261714	32.4966642	3.36687253	313.654044

cg11206381	0.00586978	17.2029159	2.27278895	130.210205
cg27037013	0.0058707	68.4736178	3.38574927	1384.81499
cg27607583	0.00587239	2.3764E+10	985.832377	5.7286E+17
cg18315708	0.00587941	82.6814156	3.57308111	1913.25533
cg16590005	0.00589394	9.80280919	1.93077138	49.7702984
cg16332610	0.00590644	6.25972873	1.69606885	23.1029559
cg03804903	0.00591089	997583.788	53.4049977	1.8634E+10
cg11910375	0.00591493	22.6021133	2.45409063	208.164898
cg14559139	0.00591939	31896.5733	19.7898988	51409630.6
cg15961211	0.00592033	8.18559887	1.83154464	36.5833448
cg01974375	0.00592472	65.3114643	3.32906779	1281.31586
cg03071876	0.00592556	20.9328219	2.3993616	182.624841
cg05406954	0.0059265	308642823	277.27022	3.4357E+14
cg20567404	0.00593825	1003896.66	53.2135143	1.8939E+10
cg13939859	0.005944	9.42498318	1.90595577	46.6066997
cg22157173	0.00595128	2573.28767	9.55401134	693092.065
cg24005914	0.00595176	16360.558	16.2566441	16465136.1
cg21931680	0.0059527	62.370121	3.27979064	1186.06107
cg21942490	0.00595847	43.0253169	2.94693301	628.171013
cg09965384	0.00595889	9.80679379	1.92690629	49.9106807
cg05637351	0.00597008	27.4845271	2.58948498	291.717942
cg12783819	0.00597076	5.12982782	1.59914875	16.4557133
cg04481181	0.00597427	13.1246852	2.09399831	82.2624162
cg12635790	0.00598557	15.5586147	2.19783269	110.140545
cg01786994	0.00599829	19.2813166	2.336109	159.140336
cg08417561	0.00599906	0.07296824	0.01127805	0.47209953
cg10612237	0.00599968	13947701.5	111.798228	1.7401E+12
cg02631680	0.00601097	134018.919	29.4587546	609702308
cg23753247	0.00601755	85.3591181	3.57461262	2038.31291
cg21491013	0.00601779	0.07791738	0.01261157	0.48139269

cg13418419	0.00261894	1.186E+12	16221.0309	8.6719E+19
cg13997864	0.00262191	8.66466305	2.1228128	35.3664656
cg00049382	0.0026275	16.7420554	2.66970433	104.991559
cg11780327	0.00262778	135828.679	61.4707715	300133373
cg12250513	0.00263318	33.1958646	3.38718553	325.333647
cg05888175	0.00263445	120.536099	5.30700475	2737.69326
cg04848570	0.00263603	0.05355434	0.00794873	0.36082088
cg25098208	0.00263626	24.0624594	3.02730273	191.260011
cg00807892	0.00263702	2986098.35	179.825186	4.9586E+10
cg13255216	0.00263759	19.8104011	2.82880923	138.73399
cg01528028	0.00263894	20.8559432	2.8796351	151.050516
cg05467676	0.00264189	15.2050116	2.57907645	89.6415374
cg21992932	0.00264377	221.878701	6.55544339	7509.81361
cg15664069	0.00264921	31478.5046	36.7276539	26979568.4
cg10350722	0.0026503	9.89158592	2.21950363	44.0834926
cg21534578	0.00265153	30.0227044	3.26560434	276.017143
cg17139983	0.00265414	178267.177	67.0440397	474004649
cg11245431	0.00265518	197538.763	69.4590153	561792628
cg25220065	0.0026568	3.6114E+10	4691.79825	2.7798E+17
cg09730369	0.0026577	0.04414755	0.00576772	0.33791587
cg15961211	0.00266119	6.3102693	1.89724029	20.9881157
cg13893782	0.0026617	15.5922585	2.59823389	93.5706849
cg06319102	0.00266776	169.37854	5.94921326	4822.3334
cg09298971	0.00267384	19.7644685	2.81893548	138.575082
cg19643344	0.00267495	1.0863E+11	6802.83372	1.7347E+18
cg13787135	0.00267562	16.7065714	2.65875142	104.97767
cg20925462	0.00267903	13.9116417	2.49442236	77.5866095
cg16259747	0.00268036	70718.1219	48.2558847	103636122
cg23058435	0.00268173	94.6296323	4.85205277	1845.56263
cg18021716	0.00268244	10.3615306	2.25143746	47.6856753

cg22256433	0.00602286	7.82769327	1.80270505	33.9893551
cg25768103	0.0060279	1513927.54	58.810943	3.8972E+10
cg22690294	0.00604458	91.8312958	3.6439407	2314.24921
cg25415989	0.00604965	190.477482	4.48800407	8084.14402
cg16489547	0.0060545	66.7976043	3.32484306	1341.99415
cg20533957	0.00605794	0.08173565	0.01366957	0.48872919
cg25917723	0.00606121	2365453.71	66.3668548	8.431E+10
cg22232207	0.00606917	6.88255618	1.73529206	27.2977562
cg02060584	0.00607301	8.31035877	1.83111587	37.7158344
cg19906093	0.00607422	444.242668	5.70589431	34587.3122
cg16797831	0.00607672	11.6067447	2.01423261	66.8823059
cg08391942	0.00607866	3.1197E+11	1917.37972	5.0758E+19
cg04738888	0.00607929	6.88590809	1.73505683	27.328056
cg02084834	0.0060821	5.0727E+11	2200.09344	1.17E+20
cg18153578	0.00609243	142232084	212.2815	9.5298E+13
cg22273939	0.00609555	27.3357085	2.57032522	290.718448
cg16000360	0.00610765	1123.9199	7.41388897	170382.366
cg16348385	0.00611162	42.5095109	2.91300224	620.342303
cg07806866	0.00611642	2.1067E+14	12112.1079	3.66E+24
cg22905097	0.00611785	18.966684	2.31361204	155.486354
cg26163234	0.00612198	10.8646152	1.97358194	59.8099634
cg10510742	0.00612463	102.101553	3.73664995	2789.85918
cg12605679	0.00612637	2910.82165	9.70514337	873030.138
cg09270822	0.00612696	3.03E-10	4.73E-17	0.00194077
cg05778739	0.00613069	89495098.7	184.172817	4.3488E+13
cg23020159	0.00613107	124446508	202.288298	7.6559E+13
cg06852305	0.00613155	0.03830508	0.00371606	0.39484806
cg21505923	0.00613396	41.1492034	2.882761	587.373333
cg16492833	0.00613622	405.785493	5.53123924	29769.4349
cg16212145	0.0061457	0.10343724	0.02040966	0.5242255

cg25479082	0.00268783	92692.8224	52.8970028	162428094
cg00741410	0.00269055	14.2733063	2.51484684	81.0098134
cg15590133	0.00269154	0.02020222	0.00157988	0.25832946
cg19048426	0.00269193	1032568.76	121.915926	8745356664
cg04550737	0.00269378	4.83968291	1.72787629	13.5556757
cg27502766	0.0026944	1873912.39	149.782543	2.3444E+10
cg15066337	0.00269527	11.2387735	2.31408262	54.5831984
cg08599448	0.00269541	46.8757995	3.79719099	578.675286
cg20154865	0.00269779	5018.22467	19.1904074	1312248.27
cg04747728	0.00270219	1031.59782	11.0800797	96045.7051
cg03233656	0.00271736	33.5881472	3.37628511	334.143473
cg22852309	0.00272221	117009701	620.368151	2.207E+13
cg01022951	0.00272293	3223777627	1954.15344	5.3183E+15
cg27591870	0.00272375	6754681.14	231.03161	1.9749E+11
cg06820762	0.00272418	52218.1991	42.9290467	63517374.1
cg07007152	0.00272751	188335.182	66.8539717	530561457
cg12649455	0.00272892	8.73808733	2.11688762	36.0690712
cg14244577	0.00272965	48.2078447	3.82183076	608.084563
cg23177883	0.00273044	406.545841	7.99035862	20684.8689
cg23062425	0.00273205	144698.522	60.9467786	343540098
cg17146950	0.0027327	174.02835	5.95623315	5084.73489
cg08225020	0.00273436	3.55E-06	9.66E-10	0.0130378
cg19370322	0.00273508	3421193373	1981.84389	5.9059E+15
cg13873511	0.0027362	146926.617	61.1961074	352758233
cg21316772	0.00273782	76.177848	4.47343079	1297.22909
cg01407244	0.00274424	20.909852	2.85959224	152.896592
cg23078565	0.00274511	2.0491E+11	8108.78588	5.178E+18
cg12829717	0.00274765	35.9598513	3.447871	375.046197
cg09674170	0.00275155	11.4173409	2.31899352	56.2121766
cg04186360	0.00275214	12.2841774	2.37827303	63.4498279

cg26277456	0.00615214	113742.992	27.4640052	471069976
cg25004679	0.00615434	67225.2494	23.6386305	191180033
cg08229199	0.00615622	86.8681378	3.56148389	2118.80036
cg02770985	0.00615737	4440.0479	10.9061267	1807610.15
cg14097171	0.00615753	23.1381525	2.44424289	219.034739
cg04037038	0.00616162	5.27833791	1.6051124	17.3575701
cg00067742	0.00616291	0.1267843	0.02892256	0.55576878
cg02319549	0.00616907	0.00013328	2.25E-07	0.07909309
cg01868762	0.00617574	479686512	293.416451	7.8421E+14
cg00676042	0.00618214	1388334064	396.148169	4.8655E+15
cg15390960	0.00618314	49884225.4	153.916372	1.6167E+13
cg24886748	0.00618499	2.9178E+10	940.267731	9.0544E+17
cg09862200	0.006187	31.4375206	2.66318776	371.103276
cg23431851	0.0061946	6.25963727	1.68346118	23.2752968
cg18859544	0.00620065	3808066228	524.700918	2.7637E+16
cg11055795	0.00620802	396689588	275.534313	5.7112E+14
cg21028142	0.00621476	43.3589837	2.91365317	645.238592
cg09179916	0.0062183	33.671662	2.71149334	418.138892
cg25060020	0.00622448	29698.4563	18.5484816	47550970.9
cg04111314	0.00622598	30.3047973	2.63071131	349.09978
cg15658376	0.00622916	7.2368E+10	1198.60858	4.3694E+18
cg27331241	0.00624344	5.15918035	1.59175301	16.7219045
cg04195127	0.00625657	240.736531	4.72350205	12269.3029
cg15185033	0.00625946	894.556972	6.8476871	116861.674
cg10786226	0.00626691	0.04092504	0.00413766	0.4047838
cg01130898	0.00627644	70.5075449	3.332482	1491.77517
cg07694975	0.00628197	14.4364038	2.1274586	97.9618377
cg18492839	0.00628316	0.00743227	0.0002209	0.25006328
cg00783905	0.00628346	6106387951	584.699891	6.3773E+16
cg00356183	0.00628734	5.26110201	1.59899173	17.3104049

cg10310846	0.00275403	4.6372E+11	10690.6934	2.0114E+19	cg11245431	0.00629409	364447.749	37.3027387	3560654425
cg24265806	0.00275595	3424.88632	16.6167175	705906.346	cg04805577	0.00630579	20.7089867	2.35364174	182.212153
cg24345247	0.00275894	24320212.9	354.737542	1.6674E+12	cg02239891	0.00630621	2.959E+11	1737.3275	5.0396E+19
cg22354234	0.00276102	96269.7492	52.5089442	176500685	cg25547520	0.00630947	12.495224	2.04038764	76.5200788
cg07164388	0.00276527	435.876064	8.14331596	23330.538	cg12030002	0.00631905	122.832351	3.88805647	3880.54717
cg18542853	0.00276654	0.06231815	0.01011931	0.3837762	cg18025886	0.00633103	13.6270056	2.08935079	88.8770245
cg01964850	0.00276775	106643.796	54.294634	209466359	cg00503383	0.00634427	15.6069716	2.16978052	112.259078
cg21241889	0.0027699	22.1517474	2.91157611	168.534119	cg17210546	0.00635426	1425.54324	7.73976117	262562.821
cg16097357	0.00277183	5.44092587	1.79370964	16.5041619	cg26982447	0.00636486	50.4561905	3.01717299	843.778984
cg24893721	0.00277385	607.716898	9.12080951	40492.001	cg04615531	0.00636889	9430430.63	92.021342	9.6644E+11
cg09072291	0.00278265	14.7005941	2.52545319	85.5717568	cg06378498	0.00637128	935093.747	47.9820668	1.8223E+10
cg20829550	0.00278597	9.29451894	2.15593952	40.0698079	cg13395333	0.00637228	5481.8827	11.2884621	2662102.03
cg02441149	0.00278686	74.8909473	4.42445797	1267.6477	cg19858931	0.00637459	1415.98566	7.70957029	260068.37
cg21090962	0.00278829	17414465.9	312.39646	9.7077E+11	cg23176237	0.00637561	4088757.54	72.6249393	2.302E+11
cg04797936	0.00279192	24356297.5	350.15563	1.6942E+12	cg27072683	0.00637854	1185.91335	7.33140708	191830.908
cg27648858	0.00279229	8.58066277	2.09669688	35.1160792	cg25444017	0.00638109	145.406352	4.06050954	5206.98377
cg26834010	0.00279615	2109957.83	150.558536	2.9569E+10	cg23085143	0.00639264	0.09026215	0.01602441	0.50842768
cg21415068	0.00279682	82539.7847	49.3092729	138165008	cg12164596	0.00639473	36.1938959	2.74367012	477.461955
cg25755428	0.0027993	7.1717556	1.97043556	26.102898	cg25110734	0.00639577	14.7855357	2.13291964	102.494281
cg09374838	0.00280228	4.78154507	1.71357577	13.3423766	cg21880079	0.00639745	49255.2892	20.8677594	116259895
cg07453451	0.0028031	23.0148994	2.94285296	179.990506	cg09374838	0.00640322	5.63007633	1.62547015	19.5006715
cg08087125	0.00280613	9.5017E+14	142524.316	6.33E+24	cg22508569	0.00640577	9065.9275	12.9521342	6345752.78
cg18797229	0.0028148	2637036.81	161.506093	4.3057E+10	cg04519918	0.00640657	160686.281	29.0555895	888644199
cg14229668	0.00281539	63710.5388	44.8823853	90437099.9	cg25137841	0.00640892	78.6059862	3.40949296	1812.26392
cg22905097	0.00281672	16.4138198	2.61727035	102.936818	cg06838028	0.00641319	60.5747298	3.16798589	1158.24313
cg14098532	0.00282363	15.7491528	2.57916225	96.1691394	cg17785773	0.00642215	14.3656816	2.11369109	97.6362198
cg21388527	0.00282612	30.6283732	3.24093123	289.452994	cg02956255	0.00642354	77.1387471	3.38856177	1756.0212
cg14548272	0.00283173	15.7103274	2.57560955	95.827563	cg24345247	0.00643097	54034386.9	148.196844	1.9702E+13
cg15447662	0.00283407	0.01228582	0.00068387	0.22071505	cg01726823	0.00644476	1127590.22	49.8800025	2.549E+10
cg10982433	0.00283492	10.8403277	2.26699365	51.8363623	cg25061763	0.006445	54457.2359	21.3148061	139132888

cg04904815	0.00283597	0.00976574	0.00046744	0.20402462
cg12493107	0.0028432	56.9565685	4.00440144	810.121248
cg16212145	0.00284713	0.11457987	0.02760971	0.47550472
cg14106308	0.00285066	385.036493	7.70795756	19233.7723
cg23797439	0.00285232	22.7924403	2.92232073	177.768076
cg16838838	0.00285359	59.2989652	4.05610358	866.932316
cg08962452	0.00285614	0.08729587	0.01758471	0.4333633
cg26433777	0.00285765	93.8557707	4.74585067	1856.12787
cg18706864	0.0028592	198734221	699.831527	5.6435E+13
cg23584743	0.00286013	5.65162046	1.81075484	17.6395022
cg21509551	0.00286099	98.5452311	4.82410987	2013.04755
cg00651087	0.00286208	3109.40046	15.7470101	613981.396
cg14323199	0.00287368	35.0446196	3.38091078	363.252816
cg21922731	0.00287469	161.475301	5.70460374	4570.74219
cg23076086	0.00287542	3922.55137	17.0079697	904658.791
cg16411251	0.0028777	17.3625518	2.65736696	113.442445
cg03120789	0.0028782	12129.0747	25.0195837	5879972.05
cg17964980	0.00288095	21725.2904	30.5262647	15461709.7
cg12216208	0.00288285	6.67093036	1.9147275	23.2415901
cg25769935	0.00288939	7093.54967	20.7732306	2422273.55
cg04963082	0.00289113	719.508574	9.49221744	54538.6357
cg12165563	0.00289259	807.031839	9.87013041	65987.0095
cg21940568	0.00289477	30.2156427	3.2081055	284.586982
cg04001842	0.00289711	166.893968	5.75396383	4840.76669
cg15923947	0.00290446	12.965902	2.40067672	70.0280108
cg06200512	0.00290522	813764510	1109.86839	5.9666E+14
cg21158815	0.00290845	9.56906141	2.16346662	42.3241733
cg05216888	0.00290851	2.5258E+10	3583.82397	1.7801E+17
cg22876277	0.00290901	2.0936E+10	3360.3219	1.3044E+17
cg14816013	0.00291045	125.500158	5.21172624	3022.0869

cg04881464	0.00644578	4246.07724	10.4178786	1730599.15
cg07410044	0.00645166	999374087	334.215585	2.9883E+15
cg27494647	0.00645352	8.39470919	1.81601566	38.8053605
cg17980283	0.00645537	484.958954	5.66352901	41526.2615
cg04467958	0.00645726	94066794.9	172.025774	5.1437E+13
cg08661338	0.00646067	0.05312132	0.00642523	0.43918683
cg26405020	0.00647467	25.7667904	2.48485826	267.189279
cg07554231	0.00648157	466702.739	38.6924539	5629300396
cg00489408	0.00648824	875.435823	6.66364235	115010.356
cg15583193	0.00648896	0.10546524	0.02087862	0.53274205
cg26996201	0.00649251	7.76833316	1.7750351	33.9976376
cg05123019	0.00649668	6975.32778	11.9018826	4088025.34
cg24546680	0.00650045	1548590294	372.669566	6.435E+15
cg13265524	0.00650478	19.9371429	2.30976083	172.091267
cg22225546	0.00650862	2378.12684	8.79613395	642951.471
cg22005150	0.00651558	598.882122	5.97762919	60000.3422
cg09827532	0.00652299	261243638	225.175178	3.0309E+14
cg06055873	0.00652827	29.3875976	2.57181825	335.805568
cg05984290	0.00653421	10169.7318	13.1649356	7855978.01
cg07730673	0.0065343	1505.36758	7.72062188	293516.711
cg14521552	0.00653908	0.10035043	0.01913785	0.52619323
cg14849798	0.00654184	7.83825605	1.77706647	34.572853
cg24448326	0.00654436	83160.9814	23.6418814	292521087
cg27361964	0.00655296	86.5275222	3.47253235	2156.06691
cg13342722	0.00655619	74.6070373	3.33118568	1670.93958
cg09118164	0.00655764	80704.2815	23.3977311	278368062
cg00930347	0.00655914	8896805227	596.952693	1.326E+17
cg16243019	0.00655996	27.2257844	2.5139615	294.850711
cg27140083	0.00656719	2513192.38	60.9593218	1.0361E+11
cg09250423	0.0065698	11.388439	1.97066987	65.8134298

cg05883874	0.00291059	11.016496	2.26986863	53.4670515
cg07371589	0.00291518	5.77966975	1.82059255	18.3481924
cg16451324	0.00291828	25.076211	3.0046186	209.283254
cg10472567	0.00291841	28.1228158	3.12455588	253.121659
cg10173186	0.00291928	16.7533958	2.61788962	107.214708
cg19119531	0.00292225	2155149311	1535.07364	3.0257E+15
cg06490988	0.00292492	9.18196317	2.1313444	39.5564638
cg00411741	0.00292549	1567.8445	12.3176112	199562.752
cg15645309	0.00292777	7.71696378	2.00830682	29.6526055
cg15021670	0.0029279	20.2346319	2.79051784	146.725572
cg20746096	0.00293389	4926818.56	191.780985	1.2657E+11
cg07628705	0.00293882	23.1685919	2.9201548	183.820271
cg10452206	0.00294275	133779005	589.130246	3.0378E+13
cg14424070	0.00294593	8.29924026	2.05695204	33.48517
cg04326808	0.00294737	12.809949	2.3847033	68.8114085
cg13878456	0.00294831	90.7639585	4.64711283	1772.73427
cg13975336	0.00294981	17681574.9	294.73436	1.0607E+12
cg03011814	0.00295359	14207.4625	25.9726144	7771723.97
cg09668408	0.00296084	1.1606E+13	28065.92	4.80E+21
cg12610087	0.00296153	19.878249	2.7672149	142.79512
cg19912332	0.00296419	2360945.33	147.69865	3.7739E+10
cg21660960	0.002966	2298.83316	13.9349433	379236.123
cg23133255	0.00296692	22.2764979	2.87552818	172.574334
cg14615266	0.00296954	8.66160781	2.08468273	35.9879462
cg22144668	0.00297388	1438866.33	124.393498	1.6643E+10
cg26280499	0.00298441	0.02345968	0.00197072	0.27926679
cg17126533	0.00299072	2964413949	1653.69158	5.314E+15
cg10784298	0.00299924	110097.213	51.5413388	235178141
cg07119315	0.00299965	6.00524521	1.83813276	19.61935
cg15695648	0.00300391	203.125452	6.07374579	6793.16366

cg12457341	0.00657112	128780.348	26.6000502	623471681
cg24287218	0.00657113	15.8335662	2.16024271	116.052617
cg11683764	0.00657832	3886.33464	10.0137939	1508279.19
cg19961545	0.00658767	12.7339718	2.03177307	79.8091289
cg14986845	0.00659567	1424238739	354.38879	5.7238E+15
cg13344867	0.00659858	3951612.72	68.7218853	2.2722E+11
cg25154306	0.00659883	3063940.31	64.0180857	1.4664E+11
cg22934878	0.00661068	0.04900865	0.00555997	0.43198948
cg01799338	0.00661255	4587.27672	10.4472652	2014221.65
cg12073353	0.00661482	280.190774	4.79785276	16362.9177
cg20227763	0.00662328	411110.489	36.4376933	4638379065
cg05682719	0.00662402	17.1045088	2.20284462	132.812009
cg07626637	0.00662517	2.6778E+10	794.833083	9.0212E+17
cg15376007	0.0066252	804003.224	43.8961509	1.4726E+10
cg18154158	0.00663625	0.04475103	0.00474963	0.42164446
cg01038207	0.00664792	678.496551	6.11903561	75233.6804
cg15462247	0.00665211	8383.25713	12.2974679	5714916.36
cg00506343	0.00666016	11.2804722	1.95972532	64.9320862
cg07226281	0.0066635	44637.4008	19.5369522	101986099
cg25451082	0.00667036	44.2582201	2.86295048	684.185795
cg00699721	0.00667381	2.206E+11	1405.02474	3.4637E+19
cg18552861	0.00667532	6.362244	1.67098992	24.2240532
cg22810403	0.00667753	83.7454191	3.41584655	2053.1646
cg00987461	0.00667959	6.67858181	1.69345871	26.3386728
cg09449611	0.00668012	1991233.93	55.896782	7.0935E+10
cg07484354	0.00669347	21.7332696	2.34792918	201.170892
cg01176915	0.00669681	5545.06049	10.9078874	2818849.78
cg06637618	0.00669712	46.4300867	2.89733566	744.046671
cg18076651	0.00669794	2843.78661	9.06367199	892256.726
cg13451356	0.00670017	56.5192053	3.05913119	1044.22477

cg19206910	0.00301396	655505.551	94.0424769	4569079224
cg12914014	0.00301936	48.1198666	3.71994893	622.460578
cg25551691	0.00302068	3754.26513	16.2983197	864782.806
cg05652528	0.00302252	0.00164302	2.37E-05	0.11374275
cg22149516	0.00302379	16.5338673	2.58864337	105.603102
cg04077077	0.00302546	45.1032784	3.63724713	559.298185
cg19170321	0.00302824	0.00147435	1.98E-05	0.1097338
cg01016800	0.00303133	318.511565	7.05051656	14388.9623
cg12019494	0.00303475	12.4839899	2.35196316	66.2637945
cg20051715	0.00303688	55.8758271	3.90708632	799.088579
cg03699566	0.0030474	9.03538035	2.10663538	38.7528372
cg26040261	0.00305014	3226.57602	15.3991921	676060.976
cg06868247	0.00305394	207554900	651.834089	6.6089E+13
cg07182669	0.00305791	6.9264646	1.92448933	24.9291649
cg03315058	0.00305914	19.0118874	2.70778367	133.48624
cg16352928	0.00305922	13.298013	2.39940946	73.7002799
cg14542839	0.00306049	49.8368663	3.7507932	662.183466
cg11537619	0.0030643	36.6896779	3.38069122	398.182613
cg01111358	0.00306504	11.2405287	2.26608312	55.7567746
cg10510891	0.00306608	2534114861	1510.97821	4.2501E+15
cg19224164	0.00306614	11.8133284	2.30434523	60.5615535
cg20225999	0.00306788	11.4514643	2.28001848	57.5153387
cg26152485	0.0030751	28.9273466	3.11706164	268.455193
cg04077706	0.00307728	31.912511	3.22168634	316.110339
cg20215290	0.00308245	0.02734427	0.0025213	0.29655652
cg16410706	0.00308337	9.01931288	2.10162971	38.7071063
cg14226064	0.00309119	11.9869405	2.31251547	62.134392
cg26435178	0.0030921	11.095583	2.25287373	54.6466319
cg18773260	0.00309461	33.8271597	3.28125029	348.731927
cg02009255	0.00309762	3507793.9	161.48567	7.6196E+10

cg16410706	0.00671892	9.64143756	1.87283223	49.6346211
cg07797438	0.00671911	74898268.3	151.475207	3.7034E+13
cg13001868	0.00672334	4.04231467	1.47208996	11.1000743
cg02408311	0.00673245	0.09932324	0.01869087	0.52780339
cg18273501	0.00673257	311.786981	4.89864554	19844.4898
cg15628917	0.00673606	8.71279214	1.82015332	41.706787
cg06856329	0.00674168	1.71E+20	394736.296	7.41E+34
cg10771262	0.00674198	25.5706273	2.45110662	266.75991
cg24798540	0.00674329	29.5628084	2.55130954	342.553353
cg16107907	0.00675481	176.35402	4.17773998	7444.39348
cg05217962	0.0067583	85.9964461	3.42481068	2159.35695
cg10978355	0.00676047	8.49923794	1.80646013	39.988176
cg03872783	0.00676262	7.74038115	1.76026855	34.0365681
cg06218124	0.00676451	217937.893	29.8491605	1591231532
cg06500727	0.00677096	0.22599386	0.07701863	0.66312819
cg26667761	0.00677336	578921.109	39.0382651	8585157399
cg08493879	0.00677502	4349466707	458.863345	4.1228E+16
cg27131958	0.00677903	304.555554	4.85000015	19124.5531
cg15680989	0.00678139	6596.45817	11.3343336	3839066.52
cg04064094	0.00678903	24410.8403	16.2494887	36671253.9
cg09920522	0.0067895	5.0186E+10	897.365825	2.8066E+18
cg00672333	0.00679417	289.554575	4.77746508	17549.4431
cg00099023	0.00679919	4574758.28	68.7345258	3.0448E+11
cg00826842	0.00680382	257206.259	31.0476036	2130762187
cg16446585	0.00680531	0.02313068	0.00151169	0.35392692
cg00690402	0.00681304	33.6713976	2.63632453	430.05442
cg05134500	0.00681322	739.087384	6.17684387	88435.1575
cg03771878	0.00683084	2968.3869	9.04514005	974149.737
cg05775779	0.00683485	0.01548749	0.00075577	0.31737438
cg09697857	0.00683521	10211.4834	12.7048909	8207421.42

cg05113000	0.00309881	8017153.74	213.336476	3.0128E+11
cg10896623	0.00309966	906.557587	9.94532043	82636.5187
cg08542090	0.00310221	23.2365247	2.88916957	186.882794
cg21700166	0.00310222	425.133412	7.7007142	23470.3448
cg00791920	0.00310272	0.0018668	2.90E-05	0.12012833
cg11808250	0.00310705	1596876.38	123.460763	2.0654E+10
cg13342722	0.00310824	24.1282087	2.92484718	199.043034
cg02519208	0.00311434	17.0263039	2.59951183	111.519024
cg13249789	0.00312871	3793.39936	16.0326242	897537.333
cg09249682	0.00313012	2142815.93	135.315879	3.3933E+10
cg02373393	0.00313999	11066206.8	234.342694	5.2257E+11
cg03339560	0.00314036	35.6145589	3.32667143	381.281059
cg01874730	0.00314122	33.0341772	3.2433374	336.461098
cg06517984	0.00314561	0.00870674	0.00037373	0.20283822
cg20362242	0.00314697	51.1241998	3.75473538	696.10333
cg23239150	0.00314763	0.03382197	0.00357256	0.32019768
cg13813874	0.00314902	4219747.96	168.912411	1.0542E+11
cg24326661	0.00315112	1.3469E+16	264560.33	6.86E+26
cg03923535	0.00315135	0.0166048	0.00109345	0.25215597
cg17222164	0.00315898	7.33298573	1.95321112	27.5303982
cg19620294	0.0031591	10.1175037	2.17630209	47.0356948
cg09798090	0.0031599	2627.46267	14.0913813	489913.653
cg11401682	0.00315999	70575.4825	42.5718446	116999834
cg06069457	0.00316034	0.08989216	0.0181543	0.44510664
cg19724344	0.00316224	1299.15961	11.1178502	151811.337
cg22810403	0.00316475	68.1323752	4.12862559	1124.35009
cg21870662	0.00316586	5958.42032	18.532653	1915687.55
cg03781098	0.00317049	11.008426	2.23755723	54.1597063
cg03075725	0.0031775	684960.956	90.8891159	5162020854
cg24174307	0.00317801	323.807063	6.95746526	15070.2893

cg05552569	0.00683737	150.709134	3.9785663	5708.9015
cg16421621	0.00684178	127788392	170.438577	9.5811E+13
cg14729962	0.00684383	12.2442306	1.99277587	75.2323362
cg27540189	0.0068585	259.797674	4.61508434	14624.8317
cg12651953	0.00687128	0.10152516	0.01933029	0.53322305
cg27071152	0.00687492	7.4188958	1.7346569	31.7296262
cg23299109	0.00687669	19.3641387	2.25789234	166.070747
cg11432962	0.00687832	13.7755221	2.0560553	92.295674
cg20313392	0.00688272	25595.2607	16.2607709	40288211.1
cg17567560	0.00688664	6.05262402	1.63983122	22.340261
cg27367469	0.00688687	31.862861	2.58789357	392.304354
cg09686308	0.00689097	117.479167	3.70254213	3727.5348
cg12582022	0.00689854	12929650.7	89.6292592	1.8652E+12
cg21784254	0.00690355	14.2802763	2.07468533	98.292636
cg14315558	0.00690892	4.1216407	1.47496537	11.5175057
cg26603598	0.00690972	0.0743722	0.01128513	0.49013396
cg17339521	0.00691588	154.476657	3.98520165	5987.91221
cg08774802	0.00691935	4310.48795	9.92801992	1871501.72
cg11159295	0.00692405	2270.59873	8.32347379	619407.08
cg18505959	0.00692693	16.1773739	2.14514151	122.000076
cg21000493	0.00693139	28308981.7	110.337957	7.2631E+12
cg21787965	0.00693286	6.35594687	1.66017113	24.3336725
cg14611402	0.00694018	38.0951555	2.71124899	535.266542
cg17475987	0.0069468	491.867443	5.46204973	44293.5516
cg10561067	0.00694916	19.5151378	2.2564281	168.780297
cg04079538	0.00695067	304.837018	4.78978753	19400.7787
cg26145223	0.00695292	1571.24109	7.50357822	329016.171
cg00212119	0.00695632	0.14513191	0.03573012	0.58951028
cg02286091	0.00696338	17.6308591	2.19338703	141.720175
cg17253939	0.00696791	2.3546E+11	1293.96966	4.2846E+19

cg18506672	0.00318803	22.0926713	2.8238034	172.847064
cg08644993	0.00319452	978.152298	10.0577267	95129.0433
cg25754252	0.0031957	927899.841	100.088284	8602386648
cg10925417	0.00319811	0.00091369	8.71E-06	0.09580342
cg00229387	0.00320084	193.011131	5.83311753	6386.51571
cg26863085	0.00320354	0.00095604	9.39E-06	0.09734938
cg16685860	0.00320763	382250579	749.348002	1.9499E+14
cg18661237	0.00321607	582.589802	8.42867704	40268.5826
cg01729739	0.00321792	3766957.97	158.919804	8.929E+10
cg04789747	0.00321795	7464791931	2017.36289	2.7622E+16
cg10510742	0.00321918	81.1500112	4.35554967	1511.93875
cg12783491	0.00321977	3515.66749	15.3748492	803904.983
cg01950845	0.00322142	7.00139033	1.91797956	25.557867
cg10530568	0.00322701	7.61108806	1.97184394	29.3779139
cg01618834	0.00323379	536.251102	8.17827085	35162.1082
cg02704345	0.00323447	421603.192	75.9984285	2338854301
cg00685025	0.00323588	192230765	588.323079	6.281E+13
cg10099601	0.0032369	11.4030308	2.25621859	57.6314333
cg06722639	0.00324	7.04961847	1.92088146	25.8720393
cg08009622	0.00324258	9.81285777	2.14513015	44.8887344
cg11705208	0.00324272	33.215	3.22417597	342.176182
cg00149397	0.00325343	2.1227E+14	60902.1565	7.40E+23
cg17607368	0.00325952	12014182.5	230.897284	6.2513E+11
cg20331595	0.00326848	859.190703	9.52629258	77491.706
cg21392341	0.00327289	7.35392306	1.94545712	27.7981888
cg08661338	0.00327798	0.07321788	0.0128177	0.4182388
cg22496559	0.00327829	0.15035013	0.04251909	0.53164739
cg16321323	0.00327842	0.00361321	8.51E-05	0.15337017
cg01850179	0.00328106	20.4481873	2.73483295	152.88991
cg14957497	0.0032815	166817.85	55.0656669	505363810

cg26329692	0.0069692	181675320	181.978347	1.8137E+14
cg11685223	0.00697031	23.3765376	2.36877889	230.693761
cg06710082	0.00698094	6.42050229	1.6628614	24.7903101
cg06310422	0.00698233	116.370018	3.67227042	3687.63179
cg00420348	0.00698332	6.65854182	1.6794019	26.3999815
cg11691214	0.0069852	0.00021725	4.74E-07	0.09963752
cg04781638	0.00698721	28.9372603	2.50937437	333.694742
cg13466546	0.00698823	603.475611	5.75707355	63258.3222
cg12260435	0.00698934	24.9671566	2.40993494	258.662132
cg22783598	0.00699273	250664542	197.576195	3.1802E+14
cg20202112	0.00699901	14.4108714	2.07306512	100.17689
cg15834388	0.00700268	48.372745	2.88561854	810.891123
cg14780540	0.00700891	17613.2902	14.4424207	21480332
cg01852715	0.00701821	2607.68746	8.56355398	794066.8
cg07273125	0.00701847	211.363529	4.31257247	10359.1398
cg20991612	0.00702087	27.004079	2.45888483	296.56545
cg13405887	0.00702676	5.1551585	1.56447114	16.9869923
cg05799507	0.00702743	11.0028117	1.92403345	62.9208729
cg16142306	0.00702772	7.9619013	1.7614579	35.9882984
cg01969023	0.00702903	0.04682382	0.00505497	0.43372579
cg14753355	0.00703057	13.6160003	2.03902056	90.9237832
cg14610403	0.00703294	9.82301501	1.86509336	51.7355463
cg05193369	0.00703806	42.6213599	2.78280258	652.788066
cg09397246	0.00704078	11.0587086	1.92590568	63.5000127
cg10382221	0.00704256	14.0670533	2.05639918	96.2274203
cg20368841	0.00704658	24242.19	15.6826366	37473531.4
cg19690901	0.00705681	275770.861	30.3817419	2503133880
cg04656882	0.00706075	26946.5815	16.1116745	45067833
cg01716223	0.00706274	3647.41487	9.34141622	1424156.14
cg26232870	0.0070688	106.156658	3.56274305	3163.07856

cg01392273	0.00328256	4172590.14	160.998161	1.0814E+11
cg27074720	0.00328599	800001.076	92.7428698	6900818618
cg18928737	0.00328907	1628.99993	11.7544456	225756.352
cg20268054	0.00329319	0.0465349	0.00601634	0.35993587
cg03110368	0.00330342	9.08499496	2.08457412	39.5942426
cg10996039	0.00330787	17.4373122	2.58916295	117.435582
cg06799455	0.00331474	6560.92073	18.6085491	2313220.69
cg07011163	0.00331725	17.1571802	2.57378595	114.371917
cg14383549	0.00331788	20.2436995	2.7192547	150.705769
cg12415590	0.0033199	102869.296	46.4286318	227921685
cg01016119	0.00332295	9.8305665	2.13800088	45.2011216
cg05981247	0.00332316	3319138.82	147.248872	7.4817E+10
cg11393995	0.00333395	2151387.85	127.056464	3.6428E+10
cg02786012	0.0033347	10.3432772	2.17318217	49.2289074
cg05562419	0.00333742	18844.5434	26.3077329	13498571.5
cg02990672	0.00334044	900.67805	9.57694529	84705.6055
cg16856874	0.00334158	122.54891	4.93727719	3041.80518
cg10501305	0.00334363	2768.46258	13.8980008	551473.925
cg24701966	0.00335256	3428866.07	147.463072	7.9729E+10
cg04401436	0.00335542	2945493.62	140.08568	6.1933E+10
cg09674867	0.00335669	14.2983105	2.41701558	84.5843468
cg11199172	0.00335758	18.0981907	2.61343572	125.330998
cg05676635	0.0033621	2449219776	1299.72688	4.6153E+15
cg00289084	0.00336871	7.1943E+10	3973.64787	1.3025E+18
cg15227911	0.00336886	1054.53206	10.0498915	110651.728
cg02706018	0.00336904	20.6919719	2.73017806	156.824094
cg16358679	0.00337273	12.559888	2.31329335	68.193161
cg18949300	0.00337312	10.09453	2.15164417	47.3589164
cg27427051	0.00337471	445729.226	74.47415	2667698020
cg07707586	0.003375	22.8588531	2.82071415	185.246409

cg10996039	0.00707031	23.7795918	2.37025365	238.568976
cg11168687	0.00707157	14.876282	2.08592011	106.094076
cg26827247	0.00708188	6.77154641	1.68308781	27.2438792
cg08554302	0.00708215	758739.472	39.8561377	1.4444E+10
cg06722639	0.00709326	8.55900737	1.79334333	40.8491814
cg22220806	0.00709659	0.07075231	0.01028863	0.48654559
cg06993761	0.00710397	229867984	187.750656	2.8143E+14
cg01841828	0.00710653	60759.9265	19.9785862	184786282
cg17715556	0.00710968	15.6023043	2.11035295	115.351274
cg24058604	0.0071121	7.62226003	1.73683118	33.4510623
cg07234865	0.00711271	10.314368	1.88562012	56.4197347
cg00868652	0.00711329	19.0252661	2.2269646	162.535476
cg22837406	0.007115	28655.5772	16.268301	50474976.2
cg22402467	0.00711635	23.651268	2.36238599	236.787079
cg27430662	0.00711981	14.1095491	2.05274347	96.9821012
cg21184629	0.0071234	10003.1927	12.2094795	8195588.1
cg04797936	0.00712986	11593729.3	82.8939921	1.6215E+12
cg08446657	0.00714179	61406.6651	19.9376865	189128188
cg19227170	0.00714553	57.0730835	2.99687443	1086.91136
cg09024228	0.0071594	202.382442	4.22139056	9702.64477
cg23843362	0.00716003	171559.183	26.277266	1120076693
cg21316772	0.00716822	65.2036764	3.1034545	1369.93129
cg13063614	0.00716843	42.390741	2.7615027	650.723581
cg25707005	0.00717389	328851950	203.391085	5.317E+14
cg03262773	0.00717705	563.244334	5.56419024	57015.3366
cg14777244	0.00719063	337.858504	4.83972067	23585.735
cg17176470	0.00719162	29.3805947	2.49778528	345.593896
cg07119315	0.00719362	6.83067373	1.6824964	27.7314731
cg08716348	0.00720019	35426.5698	17.0404227	73650863.5
cg01480708	0.00720034	38.2927176	2.68249249	546.630505

cg11420031	0.00338009	12666.4818	22.8605712	7018186.93
cg04377289	0.00338225	8.13126949	2.00201314	33.0255292
cg15535174	0.00338521	69.4258992	4.0723325	1183.58594
cg24089133	0.00338777	33.5523192	3.20022422	351.774765
cg03381111	0.00339061	0.05279741	0.00738081	0.3776777
cg06080793	0.00339189	8.41982332	2.02440006	35.0194738
cg27028281	0.00339331	9.58678491	2.11313369	43.4929628
cg05256043	0.00339987	14.8880659	2.44365931	90.7059773
cg20533957	0.00340051	0.08989483	0.01793158	0.45066197
cg26031954	0.00340501	6.75662487	1.88118769	24.2676368
cg03099482	0.00340681	66.2547675	4.00218039	1096.82568
cg23061046	0.00340705	10.2824107	2.1612553	48.9197038
cg01969023	0.00341622	0.05284683	0.00738073	0.37838893
cg07410044	0.00342295	138192977	489.118358	3.9044E+13
cg20215212	0.00342306	102.237997	4.61233854	2266.22739
cg07664976	0.00342438	0.07343738	0.01277831	0.42204719
cg14558114	0.00342715	13.3684989	2.35467681	75.8986376
cg17597901	0.00343022	23.81293	2.84876675	199.053024
cg14693391	0.00343147	9.35833663	2.09260885	41.8513304
cg09456081	0.00343224	2.68E+17	568147.031	1.26E+29
cg03318314	0.0034428	13.2222176	2.34417072	74.5794817
cg01698298	0.00344331	6.17984647	1.82383392	20.9396821
cg19768950	0.0034447	230.306112	6.01701562	8815.15165
cg25547520	0.00345355	9.0921828	2.07067656	39.9230811
cg03135127	0.0034545	3660.49305	14.9614934	895579.673
cg08375849	0.00345639	123.150882	4.8885556	3102.37644
cg26570179	0.00346172	5.06713336	1.70714423	15.0402292
cg02595823	0.00346902	11.1622425	2.21387035	56.2795641
cg04189328	0.00347299	104.963289	4.63029644	2379.39238
cg26495758	0.00347398	3785945.69	146.664044	9.7729E+10

cg06641959	0.00720251	704889174	248.246639	2.0015E+15
cg05124756	0.00721787	67676.6541	20.2535583	226139498
cg20345923	0.00721884	0.0876547	0.01484184	0.51768146
cg15557486	0.00721957	2457686.94	53.4965596	1.1291E+11
cg10082589	0.00722173	9.17744637	1.82113943	46.2488049
cg14054928	0.00722182	11.23452	1.92350805	65.6167988
cg13325330	0.00722418	261190.002	29.1547366	2339935974
cg20183756	0.00722728	0.03994658	0.00381114	0.41870092
cg01959640	0.00722787	218.01464	4.28748409	11085.8448
cg02426376	0.00723364	768.266856	6.02393773	97981.4182
cg23902823	0.00723499	154.199431	3.9025287	6092.83527
cg23963591	0.00723511	22.5417551	2.32088282	218.938551
cg04396550	0.00724017	1365.67911	7.0331176	265185.304
cg11731890	0.00724031	6.25443289	1.64105355	23.8370837
cg07664976	0.00724087	0.06036867	0.00778087	0.46837637
cg27410337	0.0072522	19.8003032	2.23952336	175.060468
cg15132559	0.0072584	14715.3794	13.3397847	16232825
cg11764177	0.0072607	224.553203	4.31225383	11693.2219
cg04408790	0.0072714	1213287743	282.401342	5.2127E+15
cg26152485	0.00727407	42.090231	2.74253538	645.967072
cg15467112	0.00727922	54.7381151	2.94322295	1018.02048
cg26466921	0.00728068	168.153705	3.98327694	7098.59463
cg11298343	0.00728575	22.07001	2.30313258	211.488191
cg01940297	0.00728902	8.95197445	1.80559132	44.383159
cg00813746	0.00729048	7.39794762	1.71506356	31.9111375
cg18098750	0.00729396	15.4200512	2.09031834	113.752042
cg00228891	0.00730391	52.2296442	2.90267909	939.799284
cg17960769	0.00730485	1061.27548	6.53294029	172404.094
cg03394309	0.00730877	25.7686135	2.3991425	276.77449
cg27316993	0.00731193	3.2842E+12	2347.56195	4.59E+21

cg06329345	0.00347423	704.368071	8.66607193	57250.2032
cg05951221	0.00347545	19.5966148	2.66380017	144.16521
cg05022363	0.00347604	1393164.54	105.458495	1.8404E+10
cg11009335	0.00347672	38932758.2	315.655349	4.8019E+12
cg26088994	0.00348355	2774931.46	132.014898	5.8329E+10
cg18431813	0.0034841	459907.526	73.0552333	2895274200
cg05096838	0.00349204	16435.4217	24.3655931	11086251.2
cg11142489	0.00349209	18.7260654	2.62161391	133.759408
cg17202086	0.00349242	7.35755323	1.92798757	28.0777689
cg07064711	0.00349613	25.3407649	2.89512553	221.805362
cg00082497	0.00349863	25.4402289	2.89837782	223.29913
cg19659689	0.00350433	1087.22929	9.95475261	118744.039
cg11932205	0.00350654	0.10304283	0.02240832	0.47383398
cg24684576	0.00350849	50.1649381	3.62053113	695.069569
cg08449860	0.00351443	824.905177	9.07846239	74954.1631
cg25658385	0.003517	19.8627791	2.66886351	147.826965
cg00334257	0.0035217	1.0485E+11	4154.60804	2.6463E+18
cg22492271	0.00352311	0.16114568	0.04728446	0.54918523
cg16751451	0.00352681	12.9875339	2.32007733	72.7027652
cg11931463	0.00352897	24.3444979	2.85107877	207.870292
cg11802553	0.00353543	42.4650078	3.42056142	527.187401
cg12899201	0.00353582	4340145.15	150.476389	1.2518E+11
cg26231243	0.00353963	45422571.4	324.650827	6.3552E+12
cg04304033	0.00354338	657295.058	80.8452011	5344000479
cg24004745	0.00354378	1.5198E+10	2180.42349	1.0593E+17
cg05583848	0.00354543	13.1948527	2.32989326	74.7262293
cg18129755	0.00354572	60.1119588	3.83036451	943.369119
cg27200446	0.00354588	4.32723096	1.61652414	11.5834507
cg01667978	0.00355285	2150.36094	12.3622286	374046.809
cg03094905	0.00356662	21391.4196	26.173145	17483295.6
cg19801120	0.00731561	199.007059	4.158813	9522.86377
cg08343943	0.00732418	131550.985	23.8653432	725137760
cg07221635	0.00732445	127.868307	3.68988745	4431.11184
cg06020661	0.00733083	1192.70108	6.72623111	211490.782
cg14015706	0.00733729	5.32150606	1.56779264	18.0626098
cg27207079	0.00734019	21.148968	2.27210364	196.85671
cg03259273	0.00734082	2148246.47	50.4573988	9.1463E+10
cg27143605	0.0073415	3826.83972	9.1944159	1592782.23
cg17213699	0.00734296	8.06105344	1.75281933	37.0720368
cg22773661	0.00734575	3.94189802	1.44600572	10.7458496
cg06727269	0.00735246	41594744.2	111.668467	1.5493E+13
cg03198733	0.00735416	19.0503934	2.20800478	164.364448
cg26572163	0.00736448	145211.674	24.3629853	865510933
cg00289084	0.00737213	5.5635E+11	1426.20338	2.17E+20
cg13300282	0.00737877	178.502394	4.02241915	7921.37848
cg06490988	0.0073808	9.03424914	1.80550811	45.2048135
cg00520042	0.00738543	95.3482613	3.39791672	2675.54848
cg13085065	0.0073893	2.0918E+14	6961.31492	6.29E+24
cg25283841	0.00738972	15764815.1	85.3789116	2.9109E+12
cg27334900	0.00739446	39.5432202	2.68193135	583.037394
cg08948823	0.00739547	2228991.67	50.458186	9.8466E+10
cg13031167	0.00739579	162.426689	3.91758238	6734.3649
cg20355694	0.00739864	752472.293	37.686543	1.5024E+10
cg26846647	0.00740713	7.1031E+12	2790.3128	1.81E+22
cg05007549	0.0074176	20.5629068	2.24846672	188.05399
cg12810402	0.00742036	3900.92575	9.16759328	1659892.76
cg19104830	0.00742255	299779.471	29.333543	3063650765
cg22821834	0.00742873	12.3719809	1.96158364	78.031804
cg04381393	0.00743512	3.75E-05	2.15E-08	0.06527999
cg21282452	0.00743746	51.5795447	2.87407459	925.671673

cg11197945	0.00356839	17.5999257	2.5572597	121.128639
cg02067788	0.00357235	764047.256	84.2630372	6927927456
cg01239651	0.00357605	25.9399894	2.90207717	231.862563
cg08724901	0.00358106	100427.573	43.2819084	233023401
cg13686104	0.00358202	6252.26057	17.4486627	2240329.98
cg13430298	0.00358246	96731026.5	409.423985	2.2854E+13
cg07461318	0.00358274	668.939825	8.39849314	53281.0448
cg10552964	0.00358297	0.02519558	0.0021163	0.29996519
cg12085570	0.00358509	12.8449889	2.30473661	71.5889779
cg13116840	0.00358775	36642261.4	297.483286	4.5134E+12
cg15448245	0.00359348	77.6719181	4.14866722	1454.18433
cg08870042	0.00359474	13.4628852	2.33924399	77.481989
cg18105529	0.00359542	41.131597	3.36973017	502.060457
cg24641522	0.00359956	18.2396444	2.5826164	128.816897
cg19408398	0.00360614	0.0582966	0.00859947	0.39519774
cg03363417	0.00360963	0.04238805	0.00504409	0.35620843
cg00541544	0.00361225	23.4852162	2.80274157	196.791379
cg13916953	0.00361668	43549818	311.570067	6.0872E+12
cg05864627	0.00361738	0.03263421	0.00325461	0.32722504
cg06811166	0.00361992	41560818.5	306.502887	5.6355E+12
cg21535580	0.00362095	21.3449537	2.71519822	167.798817
cg25353281	0.00362271	7.48300672	1.92847887	29.0360399
cg06059849	0.00362464	10.719731	2.16827247	52.9973208
cg23717805	0.00362697	347209658	611.09218	1.9728E+14
cg04131468	0.00362937	14.0100804	2.36557215	82.974579
cg08104202	0.00362974	6.82951753	1.87131978	24.9248205
cg07881273	0.00363157	7.5172E+10	3523.55184	1.6037E+18
cg00362906	0.00363281	1720.60458	11.354413	260733.878
cg19544662	0.00363286	7.03573047	1.889335	26.2004903
cg01962969	0.00364948	493.298658	7.53939719	32276.2629
cg10530568	0.00743755	9.08139948	1.80523801	45.6847331
cg01236148	0.00743808	22.5634019	2.3032958	221.034183
cg18952098	0.0074384	5685.82733	10.1219398	3193916.67
cg22502206	0.00744456	5975.36329	10.2506662	3483184.95
cg05226740	0.00744913	15.6426715	2.08733743	117.227415
cg09974990	0.00745737	9893396562	471.736331	2.0749E+17
cg13934625	0.00746209	15.1939851	2.07024619	111.511947
cg22712329	0.00746264	59153.6606	18.8872773	185265219
cg01874730	0.00746813	38.3503909	2.65118495	554.752878
cg08278489	0.00747238	12.3923463	1.95979557	78.3603401
cg04994773	0.00747757	14.7698434	2.05357765	106.228402
cg09904140	0.00747817	42.117654	2.71718849	652.84274
cg20272205	0.00748534	31.8537143	2.52098537	402.485125
cg02102829	0.00749284	28568834.5	97.985332	8.3296E+12
cg05639912	0.00749305	10.2063561	1.85962352	56.0165555
cg17365504	0.00749549	1.1604E+18	66639.0491	2.02E+31
cg26668837	0.00749615	14.51316	2.04273213	103.112793
cg20470734	0.00751094	0.12309949	0.02650121	0.57180352
cg22289434	0.00751586	11.3512951	1.91188335	67.3952736
cg25345578	0.00751726	0.07218175	0.01050461	0.49599209
cg27109877	0.00751763	21.9370222	2.27911268	211.149254
cg23460835	0.00751916	7.15201855	1.69007888	30.2656699
cg25163015	0.00753851	17402.3301	13.4933591	22443714.1
cg12850793	0.00754487	39647133.6	105.761902	1.4863E+13
cg20215290	0.00754508	0.02270823	0.00141355	0.36480061
cg26931437	0.00754932	64210444.2	120.141372	3.4318E+13
cg09285418	0.00755366	25.2519399	2.36293378	269.859643
cg17463145	0.00756144	23.9395729	2.32888426	246.084857
cg13332774	0.00756204	140.738613	3.73193522	5307.52974
cg14013195	0.00756967	9.39062357	1.81490013	48.5887954

cg24241823	0.00364971	85.4970689	4.25955889	1716.08117
cg12889538	0.00365039	22.6119708	2.76171938	185.138732
cg07706558	0.0036509	8.51380565	2.00900481	36.0799966
cg10984505	0.00365362	1628346.88	105.461734	2.5142E+10
cg09871837	0.00366472	26.5407313	2.9069572	242.318814
cg03436149	0.0036649	1179847.64	94.659757	1.4706E+10
cg09802835	0.00367364	6.64335069	1.85146557	23.8373909
cg22991959	0.00367448	126.083593	4.82271599	3296.29042
cg22566736	0.00367833	143.060765	5.02311027	4074.44422
cg00987461	0.00368109	5.86374191	1.7773286	19.3455893
cg10260334	0.00368655	0.0589049	0.00871061	0.39834045
cg08603911	0.00369921	15409.8818	22.9161853	10362303
cg06466348	0.00371095	9.59558518	2.08322936	44.1983281
cg17008160	0.00372031	13.9562173	2.35142265	82.8332588
cg02770985	0.00372061	1191.34834	9.94862382	142664.039
cg22286382	0.00372273	8.92356357	2.03368213	39.1555718
cg19843939	0.00373462	7.74789266	1.94167774	30.9164798
cg21195414	0.00373545	595827.264	74.4004498	4771612668
cg26099580	0.00374134	273441.3	57.7202498	1295388440
cg07201835	0.00374402	0.08088755	0.01477438	0.44284731
cg03997400	0.00375155	6399.44699	17.0718472	2398857.1
cg06994747	0.00375495	33.7175234	3.1229053	364.042862
cg08700651	0.00376668	19.5168343	2.61459339	145.684916
cg25913761	0.00376728	468.133874	7.30707498	29991.3885
cg12891177	0.00376758	93.4940169	4.33957997	2014.28047
cg05892930	0.00376903	70.2989071	3.95677649	1248.98041
cg21701379	0.00377191	10.870013	2.16311844	54.6235385
cg14167139	0.00377513	510.719224	7.50864022	34737.8644
cg20256260	0.00377655	17.0401395	2.50094348	116.102725
cg20567404	0.00377968	550522.682	71.696484	4227197850

cg01563704	0.00757013	6.09887716	1.61795238	22.9897388
cg14808521	0.00757735	59701009.6	117.114625	3.0434E+13
cg23709674	0.00758982	16629174.8	83.1459439	3.3258E+12
cg07217928	0.00759326	284.858006	4.49282044	18060.8338
cg17861295	0.0075974	6.10894278	1.61769073	23.0694169
cg08366813	0.00760048	8908.91691	11.2109841	7079556.98
cg03219968	0.00760399	8.14617158	1.74599042	38.0071451
cg08729318	0.00760837	5.23682708	1.55245617	17.6651414
cg09825979	0.00760921	15.5059356	2.07129417	116.079136
cg18108237	0.00761488	6.24E-09	5.89E-15	0.00662399
cg11334004	0.00762286	970.133076	6.20757093	151614.568
cg07116997	0.00762436	23.5861294	2.31407648	240.400654
cg04450855	0.0076278	67.947597	3.06395432	1506.83576
cg25756470	0.00764286	111.186643	3.48878944	3543.48401
cg18953104	0.00764604	7.55478142	1.70961019	33.3846408
cg07023915	0.007653	5553717.98	61.3817649	5.0249E+11
cg26966013	0.00766058	402.709988	4.90189446	33084.2159
cg04220455	0.00766305	454.059296	5.05937894	40750.0301
cg08869573	0.00766315	6.98209446	1.67356741	29.129178
cg20495332	0.00766355	1809.39695	7.29760616	448628.94
cg26527984	0.00766673	42.5075961	2.70052503	669.09053
cg23792245	0.00767032	261919224	169.806792	4.04E+14
cg26912602	0.00767479	113.228875	3.49927602	3663.83731
cg23375552	0.00767529	22.7738905	2.28823637	226.659315
cg22931309	0.00769001	23740.9129	14.3886557	39171897.5
cg08430407	0.00769946	69.3767547	3.06963266	1567.98374
cg12648523	0.00769949	2978.68257	8.29925114	1069078.37
cg05003322	0.00771523	12.686974	1.95740042	82.2311607
cg03514404	0.00772044	4.36445043	1.47614606	12.9041618
cg12110327	0.00772323	25.589747	2.35556736	277.994661

cg16435858	0.00378273	2758.88382	12.9386526	588271.448
cg27438798	0.00378485	4.32906293	1.6055634	11.6724047
cg24908234	0.00378493	24532868.1	244.18359	2.4648E+12
cg14777341	0.00378587	14.0679657	2.34955951	84.2318133
cg01645998	0.00378616	7.88599002	1.94878367	31.9116172
cg13768304	0.00379003	1381.6646	10.3369243	184677.474
cg18404009	0.00379191	3.2806E+10	2491.21194	4.3202E+17
cg22804475	0.00379582	4.956E+16	245967.233	9.99E+27
cg16590005	0.00380445	8.60707227	2.00313372	36.9828994
cg10534672	0.00380453	170.316527	5.24927812	5526.03972
cg24395504	0.00380597	12.7625428	2.27452866	71.6115392
cg24987648	0.00380862	2164.77458	11.917448	393225.882
cg00058923	0.0038117	685.469272	8.22004021	57161.2926
cg00551143	0.00381266	9.72841916	2.0831403	45.4324365
cg13565401	0.0038161	2.91E-05	2.46E-08	0.03445552
cg13643339	0.00381692	80803.5278	38.2486162	170704479
cg12506775	0.00382093	5.51490099	1.73413905	17.5384627
cg21510789	0.00382491	7383060.39	163.646081	3.3309E+11
cg23097623	0.00382501	306044.221	58.6511043	1596953138
cg05045781	0.00382891	12420.6523	20.8636827	7394313.13
cg07641943	0.00382923	4491012.67	139.239507	1.4485E+11
cg22555539	0.00383393	2610191.04	116.744274	5.8359E+10
cg26094150	0.00383431	100.869358	4.42108916	2301.38481
cg23299109	0.00383827	15.4343611	2.41430467	98.6700255
cg25087049	0.00384018	272.401622	6.0848561	12194.6423
cg14684375	0.00384138	1.02E-05	4.24E-09	0.02472495
cg13234997	0.00384298	513838720	638.003224	4.1384E+14
cg20801007	0.00384406	3.90333826	1.55033595	9.82757934
cg08462879	0.0038461	11.6995049	2.2073852	62.0093021
cg23084986	0.00384648	44.9842274	3.405355	594.234877
cg16754467	0.00772483	6.66745966	1.6509121	26.9275501
cg00601350	0.00772539	9.80835853	1.8281574	52.6234213
cg04282419	0.00772579	127.40668	3.5995934	4509.52657
cg05588972	0.00772625	390.095157	4.83780937	31455.1939
cg19975218	0.00772695	37.2609387	2.60105404	533.774974
cg00752778	0.00772778	3610.17518	8.70799389	1496712.67
cg02811074	0.00772982	42.2857805	2.68911412	664.935422
cg24577131	0.00773772	9.1029162	1.79186779	46.2439717
cg22351880	0.0077381	2.7052E+10	568.788994	1.2867E+18
cg20303021	0.00774578	1.3415E+10	471.603496	3.8162E+17
cg04107003	0.00774819	60.5841718	2.95441623	1242.35774
cg02706018	0.00775433	25.455053	2.34944203	275.793025
cg04844391	0.00775858	2.9475E+17	40650.1958	2.14E+30
cg05148093	0.00775941	4401.67929	9.14701208	2118154.03
cg25556905	0.00776839	4.37723773	1.47603242	12.9808871
cg19539667	0.007789	8.71559175	1.76905331	42.9390902
cg24120357	0.00779862	25.0139176	2.33463765	268.005646
cg06304787	0.00780072	11224938.2	71.867287	1.7532E+12
cg27265272	0.00780142	10433.0663	11.4324185	9521071.46
cg02862835	0.00780266	14640.3321	12.4974489	17150646.2
cg19597031	0.00780445	65.2247809	3.00406894	1416.16991
cg10355455	0.00780641	20143.9716	13.5869388	29865416.9
cg06127885	0.0078144	41.6430652	2.66815399	649.941827
cg25996566	0.00781965	833620690	222.407661	3.1245E+15
cg00463577	0.0078241	103.230626	3.38638003	3146.88904
cg01834022	0.00783033	5.774916	1.5858762	21.0291665
cg26333513	0.00783486	44.2092556	2.70796976	721.743021
cg27221266	0.00784167	56481396.3	109.025456	2.9261E+13
cg01044129	0.0078432	34.310463	2.53248555	464.842879
cg18923472	0.0078511	0.06696952	0.00912497	0.49149923

cg12111272	0.00384854	1509460.43	97.4713092	2.3376E+10
cg16124934	0.00385001	14.7778028	2.37929447	91.7849633
cg06491405	0.00385326	11.6244202	2.20207405	61.3635797
cg15579745	0.00385546	972.580224	9.14904677	103389.164
cg10320160	0.00385978	0.07254966	0.01223971	0.43003089
cg19664945	0.00386153	7.21521738	1.88818762	27.5710746
cg10709343	0.00386324	1015.33375	9.26701568	111244.295
cg11589885	0.0038667	0.01603426	0.00097106	0.26476091
cg09053081	0.00386798	5.29117665	1.70856809	16.3859728
cg13181928	0.00386943	13.2089025	2.29268097	76.1009087
cg01507044	0.0038707	264.762006	6.00975441	11664.1905
cg21229079	0.00387808	11.5109015	2.19258862	60.4312416
cg10616306	0.00388005	2918451760	1099.01088	7.75E+15
cg01381613	0.00388829	0.00397504	9.32E-05	0.16946635
cg01040523	0.00389615	8.51286836	1.98853635	36.4433508
cg14934978	0.00389931	11736.5559	20.2295855	6809172.87
cg07363112	0.00390069	13960.0536	21.3824967	9114141.31
cg14311481	0.00390095	31.5459334	3.02684734	328.773078
cg09026481	0.00390397	363324.117	60.8001173	2171121040
cg25892296	0.00390473	11.5826	2.19424224	61.140297
cg07365623	0.00390785	1.81E-06	2.28E-10	0.01439288
cg25261228	0.00391713	42.8349306	3.33519633	550.14191
cg11491247	0.00392831	21.0158776	2.65278577	166.491812
cg06025835	0.00393812	38.7124421	3.2240576	464.834492
cg00973823	0.0039383	25.8653864	2.83350543	236.109734
cg12265829	0.00393998	5.7384073	1.74954704	18.821625
cg18570262	0.00394199	0.00014537	3.58E-07	0.05909812
cg16399136	0.00394249	4.7970149	1.65188306	13.9303759
cg10500147	0.00394414	6.73104228	1.84095869	24.6105089
cg00740389	0.00394511	8.10877368	1.95394473	33.6510085
cg13320002	0.00785293	41612.1817	16.3495889	105909309
cg02572755	0.00785607	7.4731E+12	2407.0945	2.32E+22
cg26404511	0.00785816	8.82871871	1.77188764	43.9905286
cg10336707	0.00786445	13.9064502	1.99606912	96.8850997
cg11517045	0.00787325	2798.08549	8.02944279	975071.694
cg25599242	0.00787875	32.1255994	2.48541837	415.243626
cg26942275	0.00788143	17.5803082	2.12156713	145.678744
cg11845241	0.00789122	0.0667136	0.00905279	0.49163923
cg13873920	0.00789143	2.8289E+13	3370.51986	2.37E+23
cg26162295	0.00790328	33.6408455	2.51308565	450.325474
cg06451479	0.00790793	65.9791588	2.99767613	1452.20805
cg02866325	0.00792026	484858.788	30.839975	7622835114
cg24461171	0.00792156	26.6771454	2.36318009	301.149325
cg01349853	0.00792865	118.078866	3.48750396	3997.87895
cg04339790	0.00793208	24.4174666	2.30811342	258.311688
cg21611775	0.00794459	0.12585094	0.02724016	0.58143776
cg07371589	0.00794944	5.97451737	1.59607349	22.3641694
cg27586487	0.00795375	8.50846239	1.75052474	41.3555607
cg18123399	0.00796219	28320438.9	88.7318934	9.039E+12
cg04507515	0.00796435	9.61021162	1.80665279	51.1200425
cg08579540	0.00796697	12.1730026	1.92165974	77.1114622
cg10498682	0.00797157	0.00604844	0.00013898	0.26323103
cg04751811	0.00797239	4.71325477	1.49945763	14.8152039
cg11008243	0.0079725	17.8541431	2.12358313	150.109699
cg01918706	0.00797523	3274.30555	8.28627478	1293835.54
cg16370061	0.00797739	6617896.8	60.5070719	7.2383E+11
cg00072369	0.00798272	1.0399E+14	4579.7388	2.36E+24
cg23930313	0.00798495	7.77711433	1.70857104	35.4000541
cg01085803	0.00798648	19.2465942	2.16461089	171.130705
cg02417033	0.00798865	14.9436584	2.02606206	110.22018

cg23761196	0.00394703	276522.779	55.1361818	1386836100
cg22122291	0.00394741	852936589	721.048287	1.0089E+15
cg10804090	0.00395769	9.4453E+12	14114.4935	6.32E+21
cg14315558	0.00395841	3.88023464	1.54282784	9.75884701
cg10723075	0.00396401	318388295	522.830755	1.9389E+14
cg05022341	0.00396522	46106.7854	30.9656571	68651398.3
cg20701689	0.0039655	7.59774181	1.9121702	30.1885683
cg05598363	0.00396939	78.2700785	4.028873	1520.5754
cg07297068	0.00397033	0.0794019	0.01416612	0.44505209
cg27262412	0.00397226	7.77409414	1.9257472	31.3834234
cg26266703	0.00397252	3913.172	14.0586373	1089217.6
cg15215033	0.00398109	70.7590769	3.89740341	1284.66223
cg02882448	0.00398583	37.133403	3.17106766	434.834501
cg15042692	0.00398668	398423959	556.983697	2.85E+14
cg09584855	0.0039907	203.13286	5.45298136	7567.04562
cg04923496	0.00399289	6.7385E+12	12425.1011	3.65E+21
cg03368099	0.00399382	13.1135292	2.27356026	75.6367231
cg24315815	0.00400165	11.2604925	2.16490036	58.5702207
cg14884929	0.0040018	8.72970173	1.99603492	38.1795386
cg07725729	0.00400467	122.151307	4.63004689	3222.63297
cg18023065	0.00400525	0.15619224	0.04410427	0.55314414
cg15846316	0.00400932	7.86387729	1.93006753	32.040623
cg21193251	0.00400985	2.6042E+12	9090.70831	7.46E+20
cg08775774	0.00401027	305.539272	6.19892009	15059.7597
cg22233523	0.00401104	82299.7359	36.9071016	183521496
cg06813515	0.00401152	39.8607082	3.23791796	490.709179
cg25258291	0.00401431	49598521.9	283.774562	8.6689E+12
cg23599104	0.00401843	5.90240188	1.76080224	19.7854972
cg16300030	0.00401875	57.9334623	3.64584228	920.579057
cg15118279	0.00402376	8881.7556	18.1097423	4355974.89
cg17147466	0.00800304	194.757531	3.95733823	9584.85068
cg15028904	0.00800654	150.964923	3.7021135	6156.05326
cg19488206	0.00800797	90.3157521	3.23749182	2519.52299
cg26173173	0.0080099	8742573.34	64.6753559	1.1818E+12
cg04772328	0.00801001	16.1499981	2.06610525	126.238698
cg16909733	0.00801004	11.4769249	1.88997297	69.6940155
cg26069745	0.00801493	23.7384713	2.28406269	246.716092
cg05401965	0.00801769	9.82473823	1.81450391	53.1966235
cg15439862	0.00802119	16.4080091	2.0739024	129.814577
cg13951491	0.00803461	13.7583242	1.9800061	95.6014659
cg01040499	0.00803656	93.6240476	3.263044	2686.28382
cg23536255	0.00803815	259.384945	4.25482115	15812.7797
cg21197375	0.00804117	1266036.6	38.8681889	4.1238E+10
cg07064364	0.00804872	138.554763	3.61135532	5315.84975
cg10282807	0.00805	9.55982428	1.80007597	50.7702129
cg25851671	0.00805872	159.600804	3.7445898	6802.45846
cg06416883	0.00805955	9551.63399	10.861579	8399673.01
cg09089053	0.00806364	133.489525	3.57344665	4986.62916
cg04796763	0.00806863	4.78811857	1.50298239	15.2537245
cg06917617	0.00807041	7.26361249	1.67502917	31.4979986
cg00530805	0.0080736	12.3103909	1.92125602	78.8784637
cg16400756	0.00809363	0.12734382	0.02770423	0.58534185
cg07548313	0.0080957	0.15551818	0.03922614	0.61657623
cg05899618	0.00809978	9.799087	1.80929482	53.0715642
cg23864823	0.00809989	580873438	189.186812	1.7835E+15
cg23345004	0.00810006	12.4887765	1.92695483	80.9409413
cg01147995	0.00810122	2865588.72	47.5851483	1.7257E+11
cg00515408	0.00810412	93.3549417	3.24893256	2682.46416
cg26964592	0.00810465	0.02250347	0.00135672	0.37325807
cg16327839	0.00810799	0.11063183	0.02168043	0.56453672

cg06085985	0.0040238	5.44351777	1.71567832	17.2712363
cg01871408	0.00402709	0.07352681	0.01241506	0.43545434
cg05779272	0.0040287	90.0359463	4.19235583	1933.63158
cg02889982	0.00402985	2014.09741	11.2782042	359683.891
cg05240614	0.00403794	1036582132	740.998989	1.4501E+15
cg08849717	0.00403948	1984540.73	101.034629	3.8981E+10
cg02987906	0.00404345	0.08552719	0.01599663	0.45727771
cg21748136	0.00404394	13.7478951	2.30246515	82.0879388
cg00868652	0.00404543	13.7868734	2.30437381	82.4856957
cg16593646	0.00405009	11710.3203	19.6869132	6965622.32
cg13425174	0.00406205	72.3092477	3.89926971	1340.92476
cg15794034	0.00406318	17.5389061	2.48545494	123.765361
cg02132284	0.00406421	9.19870194	2.02442618	41.7975812
cg04552500	0.0040643	244065.446	51.5591846	1155331339
cg02891574	0.00406627	1096019.56	83.0635182	1.4462E+10
cg26416971	0.00406743	8.40327721	1.96681409	35.9032753
cg21103829	0.00406765	652181.993	70.407151	6041166937
cg02774070	0.00407016	1.8262E+14	33977.7219	9.82E+23
cg09893504	0.00407239	16.8430978	2.45250358	115.673611
cg16933440	0.00407519	339.276439	6.36466693	18085.55
cg16493531	0.00407713	927529.342	78.5559644	1.0952E+10
cg17278655	0.00408017	8.1053E+10	2910.26665	2.2574E+18
cg08983217	0.00408785	12.0780983	2.20506412	66.1570145
cg11139684	0.00408844	6481763.01	145.186479	2.8937E+11
cg24405664	0.0040893	16976441.5	197.027712	1.4627E+12
cg16925659	0.00409033	31620750.3	239.946663	4.1671E+12
cg00169633	0.00409395	5705626631	1245.98327	2.6127E+16
cg09234474	0.00409475	63586.9365	33.4162629	120997926
cg04573510	0.00409656	996178805	715.469431	1.387E+15
cg09175928	0.00410714	2.3024E+15	74206.8219	7.14E+25

cg15173196	0.00812058	571.843242	5.19601051	62933.8015
cg22956431	0.0081249	72.5352054	3.03965678	1730.90464
cg17283453	0.00812727	13.1505283	1.95138158	88.622541
cg09560599	0.0081326	19159.6471	12.9099238	28434875.5
cg25852200	0.00813294	28402.9871	14.2975004	56424525.6
cg00403955	0.00813517	788.430954	5.64134498	110190.632
cg10635330	0.00813896	145.468001	3.63829865	5816.16335
cg12691382	0.00815182	27.0002884	2.3496395	310.26699
cg08513472	0.00815635	6.4718421	1.62244654	25.8157905
cg23747525	0.00815716	242.430037	4.14878638	14166.1483
cg26575389	0.0081756	14.7573567	2.00759681	108.477745
cg01516119	0.0081863	17629.4671	12.557351	24750292.5
cg26435178	0.00819118	11.0157047	1.86040224	65.2255445
cg02282382	0.00820492	1835.76622	6.98152088	482708.235
cg20743280	0.00821564	11.3298965	1.87270121	68.5462017
cg27612744	0.00822071	5269.54343	9.15561819	3032901.48
cg17195127	0.00822774	556.100288	5.11812473	60422.0386
cg11785652	0.00823037	4.91650129	1.50884051	16.0202386
cg10715905	0.00824639	37.6667088	2.55124642	556.112863
cg00994629	0.00826931	8.03567535	1.71137789	37.7310463
cg09026481	0.0082772	673455.218	31.7840508	1.4269E+10
cg06848185	0.00828326	6.96274681	1.64877937	29.4034751
cg08336736	0.00828799	69052551.5	104.59838	4.5586E+13
cg15007132	0.00829605	1453541.71	38.6357945	5.4685E+10
cg05670898	0.00829702	33.4733301	2.46964148	453.694934
cg06240854	0.00830269	35.0180759	2.49792201	490.914302
cg08024097	0.00830472	14381770.7	69.601034	2.9717E+12
cg24079361	0.00830925	6.9006112	1.64402656	28.9645168
cg05434115	0.0083133	51.8358762	2.76200014	972.830531
cg25823214	0.00831605	5.5586E+11	1051.83166	2.94E+20

cg00338116	0.00410744	14.0944586	2.31360688	85.8632319
cg07217928	0.00410934	221.865419	5.54259169	8881.09157
cg23600701	0.00411332	29.0927681	2.91014568	290.84082
cg04395689	0.00411788	7.12170318	1.86267364	27.2289548
cg23764900	0.00411804	34.8302927	3.08004032	393.874484
cg16594066	0.00412043	48879.1795	30.584783	78116434.2
cg02225096	0.00412236	18169.9458	22.3462648	14774143.8
cg11406274	0.00412505	25.6371223	2.79388316	235.250367
cg20415170	0.00412771	229.43036	5.59159335	9413.82656
cg23020159	0.00412953	87128187.2	326.668918	2.3239E+13
cg15586945	0.00413034	1445886342	794.793193	2.6304E+15
cg07824564	0.00413458	124090368	364.745151	4.2217E+13
cg09200186	0.00413677	3490.39984	13.2226703	921363.895
cg00465883	0.00413838	0.06374215	0.00970995	0.41844313
cg15106368	0.00414077	94.738134	4.22110015	2126.29734
cg02583525	0.00414241	13.5970797	2.28359365	80.9603649
cg00836482	0.00414478	4.90997415	1.65434321	14.5724576
cg18334142	0.00414883	1282068.73	85.4695424	1.9231E+10
cg18556179	0.00415286	50.0820584	3.44707734	727.634551
cg15569340	0.00415936	78.9901973	3.97925186	1567.99607
cg14593867	0.00415978	3629185.71	118.42157	1.1122E+11
cg19477674	0.00416002	72.0793766	3.86554365	1344.03773
cg08104023	0.00416175	0.09148097	0.01782084	0.46960575
cg07833852	0.00417019	0.08500914	0.01574362	0.45901474
cg12832498	0.00417328	4209900.36	123.64727	1.4334E+11
cg10418263	0.00417605	18.4537144	2.51079672	135.630086
cg26835312	0.00418044	20.5185758	2.5956924	162.196396
cg15348799	0.00418145	17609.4802	21.8943268	14163202.8
cg18346784	0.00419169	2529847.18	104.760077	6.1093E+10
cg11469308	0.00419373	9.00915681	2.00064723	40.5693243

cg07647796	0.00832257	918492.129	34.1850825	2.4678E+10
cg16047144	0.00832499	6.4854647	1.61743528	26.004906
cg16990120	0.00832751	74.4358537	3.02941186	1828.96766
cg03781098	0.00833487	12.6829022	1.92139619	83.7182931
cg04776910	0.00833529	34762599.2	86.8247892	1.3918E+13
cg11166999	0.00833975	0.01601027	0.00074184	0.34552914
cg03847535	0.00834022	36.2379553	2.51614959	521.904345
cg03905819	0.00834281	11.9546695	1.89198159	75.5367401
cg16585234	0.00834477	16.2007188	2.04555683	128.30897
cg20925462	0.00834508	14.3318894	1.98211534	103.628205
cg22125112	0.00834703	11.2275084	1.86149602	67.7180851
cg19224164	0.00835391	10.9920772	1.85103784	65.2746033
cg13319446	0.00835704	9.5756516	1.78645082	51.3269676
cg04670377	0.00836407	720215.964	31.9071208	1.6257E+10
cg26507725	0.00837096	8.53176128	1.73367734	41.9864462
cg25220065	0.00837364	3.175E+10	495.616274	2.034E+18
cg27111250	0.0083779	17.647474	2.08874421	149.100755
cg10087374	0.00838395	55812.0776	16.5056015	188723083
cg26680675	0.00838657	19.9674368	2.1553608	184.97995
cg10417218	0.00838749	83.2284229	3.10821343	2228.60191
cg04385193	0.00838957	438207627	164.517105	1.1672E+15
cg10134882	0.00839069	538.754268	5.01703146	57854.1641
cg21100807	0.00839464	11180917	64.1530122	1.9487E+12
cg24548564	0.00839915	124.287118	3.44270145	4486.967
cg00027499	0.00840161	48468.2079	15.8843737	147891710
cg00500989	0.00840989	644556.381	30.7945794	1.3491E+10
cg11819639	0.00841472	2300.44975	7.26429383	728504.268
cg15794034	0.00842244	29.786353	2.38489091	372.019877
cg03822267	0.00842937	57.6898393	2.82387863	1178.56253
cg27323009	0.00843003	31.8831622	2.42607606	419.004188

cg20730198	0.00419723	29.4770258	2.90723106	298.87375
cg00357958	0.00419998	9.80577379	2.05431513	46.8054769
cg24693447	0.00420273	5878649821	1203.06612	2.8725E+16
cg06425443	0.00420395	11588.0887	19.1121108	7026110.42
cg14806045	0.00420803	3932869.98	119.868642	1.2904E+11
cg15945016	0.00421081	46.162399	3.34590625	636.887863
cg26699183	0.00421151	5.6327E+14	44526.8671	7.13E+24
cg23591302	0.00421682	4.74708563	1.6334592	13.7957667
cg09930748	0.00421879	79229.587	34.9317196	179702790
cg11363229	0.00422233	0.06446195	0.00985412	0.42168611
cg09261514	0.00422514	48844064.7	263.760159	9.0451E+12
cg17404605	0.00422942	8289.52856	17.1255591	4012498.7
cg09700470	0.00423241	16.3097738	2.40791936	110.472438
cg02884024	0.00423423	92104.6862	36.5126531	232337902
cg02994288	0.00423512	1626982.56	90.1266479	2.9371E+10
cg06240854	0.00423824	29.1023296	2.88833044	293.230156
cg20272205	0.00423911	22.4920022	2.66326923	189.950817
cg01558040	0.00424198	9.05E-05	1.53E-07	0.05345202
cg09432234	0.00424408	1295.75303	9.5296299	176184.797
cg13314965	0.00424478	0.09285506	0.01820899	0.47350586
cg23067317	0.00424811	9.94361765	2.05928291	48.0145449
cg20579480	0.00425025	13.5013284	2.26697501	80.409298
cg09718046	0.00425084	33479.4988	26.4709097	42343721.8
cg24323726	0.00425085	41.1264057	3.21767907	525.652562
cg17930034	0.00425094	10.7737102	2.11161397	54.9687745
cg13254588	0.00425114	18873679.4	193.988868	1.8363E+12
cg07046854	0.00425424	1174489.75	80.9526113	1.704E+10
cg07336964	0.00426246	0.05679255	0.00794372	0.40603076
cg21367811	0.0042682	7.91576435	1.91532621	32.7147016
cg15834388	0.00427158	29.2704577	2.88775106	296.687519

cg00705992	0.00843175	32.6712642	2.4411219	437.262682
cg26399396	0.0084346	1271.59529	6.23070091	259514.073
cg25619837	0.00843969	78642.7453	17.8948087	345613160
cg19283196	0.00844114	30.8089662	2.40384242	394.864652
cg24880056	0.00844491	9.33504921	1.77084679	49.209872
cg02710004	0.00844714	0.09370294	0.01608914	0.54572492
cg00761126	0.00845369	112.441108	3.34561141	3778.98124
cg26053876	0.00845582	23.2952304	2.23672695	242.616902
cg13570585	0.00845791	8.88367896	1.74797277	45.1493027
cg10300057	0.00846741	2.1564E+13	2556.86683	1.82E+23
cg20691205	0.00847188	53.9470308	2.77046103	1050.46853
cg21329975	0.00847483	26.6539633	2.31346419	307.086559
cg26534477	0.00848104	0.05585942	0.00651935	0.47861725
cg21153026	0.00848404	3324950078	270.20483	4.0914E+16
cg23575732	0.00848905	42.4004393	2.60320754	690.608499
cg16335926	0.00848914	7241292.01	56.4258165	9.293E+11
cg23979631	0.00848915	8.9247027	1.74867707	45.5489009
cg22971415	0.00850259	93317.308	18.5436747	469600557
cg08160980	0.00850983	5.69320093	1.55841826	20.7983554
cg18357526	0.00851426	4393.01777	8.49279814	2272349.44
cg16570157	0.00851955	4.61750198	1.47710282	14.4345567
cg02528979	0.00852454	1350950.84	36.5465802	4.9938E+10
cg18706879	0.00852593	6116830.14	53.6959449	6.9681E+11
cg16364495	0.00853094	11.5077499	1.86378269	71.0535124
cg09161136	0.0085321	149635485	121.147485	1.8482E+14
cg05095290	0.00853412	345.756901	4.43525893	26953.9697
cg15345154	0.00853763	12.5304612	1.90430252	82.4514255
cg07488684	0.00854538	73.9420558	2.99216602	1827.24741
cg11923320	0.00854618	17.7258365	2.07967229	151.084035
cg24533564	0.00854635	26.7039297	2.3084325	308.910857

cg11896633	0.00427184	1485385152	759.531452	2.9049E+15
cg01657408	0.00427623	74339.1703	33.844967	163283133
cg06734406	0.00427689	5.26882326	1.68499809	16.475092
cg15706223	0.00427783	1599.21067	10.1365478	252302.344
cg03394309	0.00427821	21.9661782	2.63781781	182.921271
cg11565355	0.00427831	695.927969	7.80584159	62045.2942
cg16449219	0.00427894	18.9804871	2.51946747	142.990094
cg17031543	0.00428301	12.2119604	2.19333023	67.9933984
cg08389277	0.00428737	19.2302042	2.52870509	146.241155
cg14122599	0.00428794	33.5730206	3.01176777	374.247883
cg17349406	0.00429048	26.1649735	2.78474005	245.84192
cg15173196	0.0042916	208.753597	5.34192336	8157.74795
cg00993830	0.00429175	9.91056361	2.05345708	47.8311781
cg07238832	0.00429511	70.7671745	3.8036414	1316.63121
cg27298072	0.00429545	0.10158219	0.02114174	0.48808374
cg17512993	0.00430727	393164.661	56.6931109	2726582615
cg22251932	0.00431363	7278.69837	16.220488	3266205.67
cg11304315	0.00431621	7685.38218	16.4924249	3581347.18
cg07689503	0.0043166	214.978494	5.37856523	8592.58016
cg23269029	0.00431874	20.8968219	2.59113341	168.527473
cg17469276	0.00431943	18.8342	2.50805643	141.435051
cg11268628	0.00431966	134.763593	4.64529538	3909.59553
cg14860524	0.00432171	37.0942018	3.1007927	443.750981
cg01911068	0.00432646	48947.2212	29.398626	81494640.8
cg07557790	0.00432957	48.3148088	3.3665364	693.389428
cg04159381	0.00433195	2166.80214	11.0688389	424166.578
cg06835221	0.0043343	1375977.16	83.3898877	2.2704E+10
cg06872500	0.00434081	384.608482	6.43723431	22979.3848
cg16016641	0.00434269	152.068583	4.81458281	4803.08569
cg23042086	0.00434654	111.851141	4.37211353	2861.47138

cg03697629	0.00855014	686733.081	30.6373402	1.5393E+10
cg27576259	0.00855394	38.2653725	2.52912454	578.950823
cg02441296	0.00855452	119778.186	19.6283075	730924650
cg12165563	0.00855482	828.261166	5.53240634	123999.67
cg05487507	0.00856094	36.7423805	2.50239685	539.483786
cg00739123	0.00857118	6714448.2	54.5480448	8.265E+11
cg07706558	0.00857256	11.2884501	1.8525583	68.7854772
cg21368609	0.0085808	24.8978006	2.26479861	273.711081
cg23797439	0.0085817	23.5436951	2.23274922	248.261459
cg16257101	0.00858238	41485.6863	14.9349148	115237495
cg01027365	0.0085884	580.652015	5.04196581	66870.1009
cg20041105	0.0086048	28.5402952	2.3426963	347.69699
cg00054301	0.00861623	5684.65361	8.9802689	3598476.5
cg25044834	0.00861831	34015042.7	81.6685709	1.4167E+13
cg07571451	0.00862542	0.00262676	3.12E-05	0.22134894
cg18133072	0.00862759	113.527907	3.32286121	3878.76134
cg18188377	0.00862894	160062.759	20.9211603	1224601625
cg27106909	0.00863053	16.5965337	2.03965639	135.044772
cg00293996	0.00863434	71.6683939	2.9558073	1737.7177
cg22467567	0.0086431	14.9168537	1.98442124	112.129682
cg05006903	0.00864845	5436.60875	8.85159173	3339141.2
cg20600850	0.00865218	17.4041868	2.06296766	146.830086
cg01850783	0.00865811	10.0697868	1.79551221	56.4744737
cg11197945	0.00866289	21.4437245	2.17425717	211.489849
cg03442712	0.00867339	44.3627026	2.61282804	753.225757
cg26580856	0.00867421	98930.3537	18.4090804	531651483
cg15821939	0.00867512	36.4857197	2.48645619	535.383551
cg20511194	0.0086809	100167.364	18.4512187	543785267
cg08542090	0.0086849	29.8154264	2.36165188	376.414349
cg21097354	0.00869008	46.6979408	2.64510109	824.42886

cg15945129	0.00434879	6.59370236	1.80360688	24.1055362
cg01327552	0.00434979	11.0972392	2.1223673	58.024225
cg01323964	0.00435171	0.01461473	0.00080049	0.26682345
cg06900571	0.00435221	0.18455118	0.05776633	0.58960189
cg21951729	0.00435727	22.3446983	2.64049671	189.087734
cg05842457	0.0043589	3126643.04	107.116326	9.1264E+10
cg19840532	0.00436004	147342.113	41.2204977	526672394
cg15701440	0.00436232	200860.408	45.3892919	888863906
cg18894815	0.00436265	7.03085452	1.8393018	26.8759131
cg10437377	0.00436339	50323.5812	29.4466898	86001612.9
cg06364315	0.00436474	423.517962	6.61738048	27105.5087
cg22308501	0.00436666	438.133582	6.68652019	28708.6602
cg27313566	0.00436773	0.1296476	0.03181794	0.52827118
cg07214715	0.00437553	976.088619	8.57844361	111063.153
cg04716996	0.00437728	26.6416098	2.7864993	254.71938
cg09825979	0.0043778	10.3801083	2.07610026	51.8985761
cg00079898	0.00437993	0.09171235	0.01773115	0.47437178
cg00069002	0.00438106	62.406292	3.63370973	1071.78216
cg26843430	0.00438108	644.631759	7.53143946	55175.3894
cg02716556	0.00439338	385748.932	55.2669337	2692427974
cg16522557	0.00439615	7.74210072	1.89324768	31.6599481
cg19904411	0.00439718	1547.96857	9.87938393	242546.165
cg19264533	0.00439922	3911.39171	13.1869263	1160163.09
cg13211302	0.00440548	54.9455839	3.48615758	866.001354
cg09998519	0.00440982	1491.32366	9.74944988	228120.181
cg13112903	0.00441342	119.252647	4.43566961	3206.09853
cg02917792	0.00441525	1976294300	787.021657	4.9627E+15
cg03065614	0.00441715	15.6418535	2.35517603	103.88505
cg14829271	0.00441872	1.56E-06	1.56E-10	0.01552284
cg26293512	0.00441933	16.263058	2.38367029	110.957903

cg00264591	0.00869191	9.4936391	1.76740016	50.9953463
cg05734154	0.00869516	23786.2769	12.8011951	44197980
cg25163611	0.00870135	22.6425871	2.20148242	232.882509
cg03553689	0.0087025	1.4057E+11	660.101217	2.9937E+19
cg09370702	0.00870307	11127253.5	60.5591063	2.0445E+12
cg20547295	0.00870612	2716.57197	7.3861673	999132.968
cg16408081	0.00871043	82.6309074	3.05301096	2236.43706
cg04918504	0.00871463	11.2864401	1.84534782	69.0296586
cg09584855	0.00872144	246.003263	4.02003449	15054.0015
cg10005098	0.00873183	1913218030	221.095847	1.6556E+16
cg22581219	0.00874128	104.788882	3.23683767	3392.41905
cg27648858	0.00874314	8.65255691	1.72427132	43.4193507
cg04071446	0.00874505	9674.39214	10.1434504	9227024.34
cg19483962	0.00874568	51628398.3	88.5307379	3.0108E+13
cg10637512	0.00874759	14.8878725	1.97721038	112.101753
cg25361850	0.00874824	61.6362089	2.83000621	1342.40774
cg27308245	0.00875057	25.4900292	2.26442161	286.9349
cg19789653	0.00876113	20.3525218	2.13861185	193.688791
cg01086810	0.00877293	459781155	152.831579	1.3832E+15
cg24389730	0.00878445	7.16890167	1.64281358	31.2836171
cg02898915	0.00878447	86.5380421	3.07756896	2433.35984
cg11705208	0.00878986	49.1774291	2.66840497	906.316528
cg01722450	0.00880418	0.11389524	0.0224172	0.57866851
cg06924355	0.00880522	3009.19419	7.51324061	1205238.87
cg25388707	0.00881206	1008947.44	32.4518535	3.1369E+10
cg01871408	0.00881392	0.06294125	0.00794634	0.49854407
cg00168835	0.00881406	18.1182409	2.07325984	158.335509
cg08339719	0.00881925	49693.966	15.1963004	162506017
cg11331344	0.00881943	166.000148	3.61946794	7613.28723
cg19593490	0.00882597	16.4088343	2.02144349	133.196818

cg05286653	0.00441933	12.0014277	2.16841451	66.4237706
cg06830784	0.00442313	6.1160985	1.75754534	21.2834685
cg02216011	0.0044262	70.6017619	3.76373099	1324.37966
cg16542392	0.00443976	574340887	530.950588	6.2128E+14
cg25229048	0.00444041	0.08303248	0.01495255	0.46108491
cg10398590	0.00444616	25.4659416	2.73684379	236.956958
cg11934386	0.00444944	4207721435	982.896763	1.8013E+16
cg19217955	0.00445016	24790.5492	23.2438331	26440188.6
cg22581219	0.00446421	42.9235478	3.21553892	572.977347
cg09946603	0.00446682	0.00017502	4.50E-07	0.06807
cg27094166	0.0044736	264792.795	48.2985369	1451704932
cg03733196	0.00448063	30446.4385	24.6430185	37616561.4
cg16180217	0.00448317	247.659151	5.5328611	11085.5946
cg02695343	0.00449755	13.3381557	2.23307174	79.6689129
cg02455803	0.00449894	1663.37399	9.9719932	277458.374
cg03976754	0.00449933	5078.86255	14.0954407	1830013.36
cg18306747	0.00450073	474.291595	6.75646883	33294.3912
cg23283234	0.00450181	71852.7825	32.0384662	161144492
cg12783819	0.00450403	4.93676352	1.64045949	14.85659
cg10139651	0.00451022	10.8705581	2.09471705	56.412886
cg12959265	0.00451198	0.15084158	0.04088712	0.55648778
cg02155398	0.00451388	7.06394524	1.83256327	27.2292495
cg04330122	0.00451454	10.2200801	2.05468966	50.8349461
cg02534163	0.00451559	5.49261855	1.69505164	17.7981944
cg05460965	0.00452028	32.6723844	2.94421685	362.57
cg00214668	0.00452109	96.1610024	4.11286226	2248.29761
cg04294383	0.0045296	0.08965891	0.0169598	0.47398676
cg13654525	0.00453043	37.9105558	3.08103533	466.469899
cg09601584	0.00453111	17.5262521	2.42640166	126.594668
cg27011534	0.00453491	3.07E-08	2.00E-13	0.00473363

cg26488040	0.00883614	141550245	112.074027	1.7878E+14
cg18856581	0.00883621	6.71289453	1.6140671	27.9188846
cg17202331	0.00883883	844.11757	5.44179712	130937.346
cg04528624	0.00884361	3.6618E+10	452.184773	2.9653E+18
cg21109744	0.00885518	10.306944	1.79693985	59.1188926
cg19245758	0.00886382	22.6887655	2.19027114	235.030298
cg03271650	0.00886867	249.694137	3.999044	15590.5166
cg09219813	0.00889189	17.9620314	2.06362541	156.343575
cg15154229	0.00889449	28.4908361	2.31655783	350.402538
cg00750366	0.00889453	10.1963307	1.79027364	58.0722175
cg25544073	0.00889859	39176.3877	14.1818653	108221967
cg18773844	0.00889883	305.963846	4.20042039	22286.7871
cg22673001	0.008905	2064.10501	6.77577551	628788.468
cg21527360	0.008907	9585.70878	9.95525006	9229884.96
cg07932328	0.00891824	70368389.8	92.4893026	5.3538E+13
cg19769982	0.00891884	5.56939374	1.53762614	20.1727493
cg18251245	0.00891901	1278673646	191.223554	8.5502E+15
cg24364084	0.00891925	253767.579	22.5930157	2850349195
cg14993900	0.00892056	15.3317406	1.98156962	118.624281
cg08016383	0.00892077	13.1897648	1.90824647	91.1674145
cg19800926	0.0089322	1.2859E+10	339.801887	4.8663E+17
cg26792589	0.00893618	982.648053	5.61216462	172054.325
cg19048532	0.00894416	39.2832547	2.50588794	615.819277
cg12910830	0.00894991	211.364711	3.81688556	11704.5796
cg14553243	0.00895042	12.6489713	1.88678099	84.7986474
cg20607710	0.00895592	2799886037	230.672706	3.3985E+16
cg27621997	0.00895691	14.8114022	1.96239531	111.790746
cg15872683	0.0089575	275346.171	22.9389424	3305100662
cg01072639	0.00896024	10.7854388	1.81256291	64.1774638
cg03423942	0.00897647	634603.81	28.1880895	1.4287E+10

cg05346193	0.00453864	54.054043	3.43671762	850.183195
cg19590227	0.00454231	219532.405	44.9102366	1073128993
cg01362243	0.00454254	23.360924	2.65048183	205.899458
cg02738049	0.00454257	8942.03266	16.6849316	4792344.97
cg01731839	0.00454665	21295026	184.669046	2.4556E+12
cg01476568	0.00454801	4.27230519	1.56685337	11.6492021
cg19265600	0.00454838	61.9120809	3.58165686	1070.20463
cg10148273	0.00455004	456.871206	6.64404203	31416.3122
cg15991262	0.00455274	30.7001284	2.88249458	326.972995
cg13560901	0.00455343	20.8330896	2.55678455	169.751347
cg27665327	0.00455383	30.2083089	2.8679575	318.185302
cg14847134	0.00455409	47.1160251	3.29035723	674.674409
cg12552766	0.0045559	63567435.1	258.255374	1.5647E+13
cg07107420	0.00456259	9.27865048	1.99043437	43.2535511
cg11249215	0.00456373	61331781.8	254.802119	1.4763E+13
cg21351151	0.00456394	56933609.5	248.995177	1.3018E+13
cg01556457	0.00456548	124301.333	37.490028	412131498
cg20864214	0.00457426	12.3248866	2.17184774	69.9417493
cg25722212	0.00457445	1277.45789	9.10368585	179256.918
cg09743140	0.00457668	192564867	361.329745	1.0262E+14
cg09667023	0.00457688	6.1399E+10	2142.19701	1.7598E+18
cg26643856	0.00457696	136.328112	4.56093101	4074.90362
cg19019038	0.00457733	126177.513	37.5747998	423708570
cg05337753	0.0045835	25.437459	2.71508788	238.321686
cg17611742	0.00458662	15.4403378	2.32703301	102.449784
cg01973771	0.00458965	1221.72431	8.96267005	166536.343
cg02980693	0.0045903	15.3872531	2.32416408	101.872136
cg17852791	0.00459795	53.8377552	3.4186599	847.847977
cg04102760	0.00460257	12355.5977	18.2634872	8358797.68
cg07235774	0.00460338	58.5502362	3.50696707	977.519914

cg15366555	0.00897684	19.4763874	2.10013563	180.621509
cg03599590	0.00898213	33.5576029	2.40550358	468.140111
cg02378269	0.00898782	12.3491314	1.87356308	81.39627
cg09634802	0.00899205	3762862956	246.220939	5.7506E+16
cg09158314	0.008996	34.5721412	2.4221712	493.455189
cg02703870	0.0090046	10.3897594	1.79366344	60.182472
cg12749863	0.00900471	3.93433283	1.40759244	10.9967732
cg20898273	0.00900899	15.1564857	1.97068165	116.568325
cg19255465	0.00901536	47.2369026	2.61636283	852.834682
cg02029242	0.00902051	0.10335571	0.01881553	0.56774388
cg10883583	0.00902471	35576856.4	76.3990763	1.6567E+13
cg26231243	0.00902976	29846155.1	73.0557687	1.2193E+13
cg16463733	0.0090337	1503478.39	34.6594554	6.5219E+10
cg17346426	0.00903627	116.667351	3.2748362	4156.32112
cg08284713	0.00903673	139221449	107.09953	1.8098E+14
cg01758575	0.00903856	555.605188	4.8313447	63894.6596
cg17141902	0.00904705	107.321219	3.20562416	3593.01136
cg00561338	0.0090613	11.6117847	1.8413549	73.2251797
cg10428938	0.00906753	5.84652457	1.55199228	22.0244972
cg25385940	0.0090734	8.44792031	1.70066789	41.9643117
cg12441126	0.00908635	5.91591354	1.55599171	22.492429
cg18661868	0.00908642	44.5271565	2.57053446	771.305618
cg18130930	0.00909494	0.1346154	0.02983381	0.60740845
cg26181929	0.00909766	2338.32569	6.87792617	794973.205
cg22761308	0.00910275	46.1304806	2.59149191	821.156816
cg07907315	0.00910496	13.082263	1.89453106	90.3366584
cg05794119	0.00910537	31533.7615	13.1204896	75788186.3
cg16345031	0.00910837	4632058519	252.138611	8.5096E+16
cg00178359	0.0091115	10.0364947	1.77348536	56.798454
cg07483245	0.00911605	1182.42859	5.7973844	241166.926

cg13389876	0.00461391	795712.379	65.789829	9623952510
cg22037648	0.00461475	18.2169756	2.44547919	135.702729
cg02153299	0.00461592	159129.075	40.0499897	632261397
cg18722881	0.00461772	7.3519421	1.84885414	29.2348927
cg04062119	0.00461815	19.7178217	2.50543451	155.179666
cg19822127	0.00461873	49340259.2	234.328736	1.0389E+13
cg23461714	0.00462997	402.100238	6.33502832	25522.3171
cg10683958	0.00463395	13731.006	18.7724406	10043474.4
cg02083373	0.0046359	1.8422E+10	1442.70051	2.3524E+17
cg24490630	0.0046388	36466.0609	25.3340833	52489509
cg07671620	0.00463946	103009.41	34.8674181	304322463
cg14397459	0.00464045	7.9227E+12	9303.78341	6.75E+21
cg12260435	0.00464177	22.6491606	2.61147121	196.435049
cg24963001	0.00464624	9.16689979	1.97677725	42.509621
cg07268337	0.00464788	0.05207288	0.00672867	0.40298994
cg05523897	0.00465641	16.2781502	2.35751728	112.397128
cg04986324	0.00466051	17.9388915	2.42849973	132.511371
cg25853673	0.00466262	73423.3932	31.280902	172341407
cg17184255	0.00466518	12.3730905	2.16607491	70.6777817
cg21940081	0.00467188	0.00048162	2.42E-06	0.09574284
cg17934743	0.00467638	5.8169E+11	4098.20307	8.26E+19
cg19367800	0.00468263	1.2965E+18	363080.109	4.63E+30
cg05696801	0.00468548	1026.13068	8.39834077	125375.263
cg11314310	0.00468862	4.08762684	1.54042148	10.8468321
cg13694725	0.00470531	393.715933	6.24667021	24815.1785
cg03591499	0.00470841	127.136561	4.41603573	3660.22971
cg10296162	0.00470955	33748172.2	203.015273	5.6101E+12
cg03774468	0.00471313	48.7634986	3.29099512	722.540968
cg13994177	0.00471531	0.07971986	0.01379479	0.46069987
cg03415695	0.00471585	1577.39755	9.54737327	260614.407

cg13975303	0.00912279	14068.9546	10.7167846	18469670.6
cg07469063	0.00912346	168.123954	3.56972834	7918.15542
cg14519917	0.00912656	772.958192	5.21251245	114621.188
cg26279336	0.0091285	5.69751478	1.54027895	21.075192
cg13647118	0.00913332	261328.611	22.1073858	3089132464
cg18802611	0.00913389	248094913	121.193481	5.0787E+14
cg12606911	0.00913449	9.54331253	1.75044082	52.0296448
cg06794000	0.00913563	4094.5728	7.87869427	2127957.48
cg06035374	0.00913767	2003206.37	36.625368	1.0956E+11
cg25292098	0.00914302	803.51998	5.25667669	122823.677
cg06403845	0.00914931	0.12107955	0.02474934	0.59234943
cg26511108	0.00915062	0.07368694	0.01036767	0.52372079
cg22721468	0.00915507	506066886	143.987959	1.7786E+15
cg26738040	0.00916026	306.735675	4.13511315	22753.1318
cg10500406	0.00916253	275348.199	22.3076739	3398679348
cg12621171	0.00916838	19.1875614	2.07949493	177.044198
cg02801583	0.00917008	74843.4424	16.1370085	347123872
cg15923100	0.0091747	338014978	129.735533	8.8067E+14
cg23637385	0.00919169	9.05597109	1.72546892	47.5294636
cg12729166	0.00919371	13.3720278	1.90014468	94.1039537
cg09239591	0.00919583	40.9898129	2.50712558	670.155803
cg16721177	0.00920146	6.48577856	1.58829423	26.4845912
cg15243267	0.0092118	140869.398	18.7746287	1056968294
cg17346022	0.00921188	0.06389197	0.0080606	0.50643696
cg17306261	0.00922028	26.6463445	2.25166981	315.333834
cg26181939	0.00922044	16.7065897	2.00618291	139.124971
cg16412592	0.00922395	1585150.16	34.1029342	7.368E+10
cg20313628	0.00923074	31.3996614	2.3440295	420.617036
cg01305328	0.00923752	1206672099	175.324399	8.3049E+15
cg18982073	0.00923789	154.800233	3.47561092	6894.64749

cg00403955	0.0047169	300.549554	5.74403599	15725.8824
cg19519964	0.00471882	3422.2741	12.0997878	967947.56
cg21845080	0.00471953	0.11302648	0.02491255	0.51279319
cg23917399	0.00472108	9.33946359	1.98258877	43.9958007
cg19975218	0.0047333	21.7646599	2.56751451	184.497661
cg20346912	0.00473605	7.01406065	1.81522185	27.1024982
cg18340446	0.00474007	1.8739E+13	11522.937	3.05E+22
cg02233421	0.0047423	627213.246	59.4072243	6622030583
cg25818813	0.00474307	1116530.2	70.8587861	1.7593E+10
cg07185648	0.00474711	70.998613	3.6836665	1368.42004
cg02409878	0.00475354	5.01086807	1.63690061	15.3392324
cg23400797	0.00475566	1397699.57	75.6752299	2.5815E+10
cg17475987	0.00475768	103.302333	4.12804767	2585.08933
cg19005368	0.00475776	11024.5262	17.2110412	7061756.3
cg25365260	0.00475901	9.17482068	1.96906419	42.7499189
cg22799396	0.00476088	13.1712289	2.19901273	78.8905256
cg04295543	0.00476159	36.5585009	3.00419825	444.885417
cg16944093	0.00476275	26.2513494	2.71479765	253.843356
cg14482741	0.00476335	14.6564036	2.2717634	94.5565755
cg02951971	0.00477147	115405.893	35.1921484	378451467
cg09829176	0.00477275	46.0881378	3.22232975	659.186554
cg06945667	0.00478209	4280751.03	105.847499	1.7312E+11
cg04974640	0.0047834	5577.56988	13.923637	2234278.71
cg22670485	0.00478485	2.82E-08	1.60E-13	0.00495811
cg05508558	0.00478587	45.6890188	3.2111182	650.080847
cg07416582	0.00478993	43364151.8	214.14959	8.781E+12
cg25625860	0.00480804	16854187.8	159.700517	1.7787E+12
cg26402660	0.00481138	6.52260464	1.77116548	24.0205514
cg13976502	0.00481516	28.3071821	2.7700645	289.27
cg08807101	0.00481851	7073.51035	14.8946077	3359239.1

cg25032094	0.00923831	7.12485844	1.62440112	31.2506605
cg02384857	0.00924641	8.1716687	1.68005313	39.7464628
cg10059687	0.0092478	50.6816947	2.63657235	974.23239
cg11275803	0.00925293	44435.0538	14.0466483	140565491
cg07194495	0.00926382	351246467	128.530039	9.5989E+14
cg10320160	0.00926584	0.06880359	0.00916363	0.51660031
cg14574127	0.0092708	515075.581	25.6582979	1.034E+10
cg02407720	0.00927452	8.37212791	1.68905762	41.4980075
cg25853673	0.00928173	135627.18	18.4351858	997805621
cg09552892	0.00928552	15.4308111	1.96341002	121.273667
cg11235663	0.00928847	17.063138	2.01251634	144.669969
cg10449409	0.00929047	86.2343365	3.00028072	2478.555
cg06390536	0.00929118	6.23376792	1.57002579	24.7510982
cg23735712	0.00929263	10.5748199	1.78841561	62.5284278
cg01589129	0.00929407	6.29499832	1.57372394	25.1804035
cg15767556	0.00929697	1082.1389	5.59456128	209314.822
cg25551287	0.00930512	29817206.6	69.3984267	1.2811E+13
cg23281832	0.00930564	39025.6732	13.5226827	112625816
cg09132763	0.00932701	75.4048962	2.89775863	1962.17115
cg09168604	0.00932771	7.43942557	1.63868421	33.7740807
cg17056132	0.00933248	64.0866445	2.7833829	1475.5778
cg16613143	0.0093362	256153.436	21.4092108	3064782884
cg13721644	0.00934081	84.137921	2.97503705	2379.52994
cg21502048	0.00934942	17.1992436	2.01273344	146.971265
cg11247103	0.00935381	26172.1974	12.1912761	56186400.1
cg00551143	0.00935471	13.807552	1.90663179	99.9922963
cg14847688	0.00935534	33.1486042	2.36458573	464.702948
cg23611233	0.00936425	13758386.2	56.7674256	3.3345E+12
cg07029862	0.00936696	433.997337	4.44642533	42360.6998
cg01090026	0.00937744	8.97997984	1.71437722	47.0375113

cg00476814	0.00481899	2158.41236	10.3734633	449102.075
cg16161657	0.00482052	4726090.7	108.038539	2.0674E+11
cg22721468	0.00482145	202188427	339.203631	1.2052E+14
cg02779075	0.00483017	72.7414398	3.68935556	1434.21174
cg06090684	0.00483388	21.6425476	2.55004127	183.683249
cg27455017	0.00484022	11.5324804	2.10475789	63.1892656
cg03583016	0.00484397	9.05969062	1.95543789	41.9742272
cg25499012	0.00484641	2.73E-08	1.49E-13	0.00499417
cg24683514	0.00485748	293129.382	45.9605882	1869532954
cg23414387	0.00486198	16.0085821	2.32339418	110.30186
cg08000847	0.00486236	56245715.1	226.974233	1.3938E+13
cg14579118	0.00486317	16.2584362	2.33423219	113.243554
cg09925572	0.00486405	2287173372	699.692549	7.4764E+15
cg15923100	0.00486909	246301372	354.836849	1.7096E+14
cg11669397	0.0048695	173322.818	39.0811559	768677349
cg08734623	0.0048796	117.127494	4.24890472	3228.79678
cg21926750	0.00487971	292092.355	45.7020631	1866829159
cg04915044	0.00488265	4.27986945	1.55504091	11.779293
cg07309576	0.00488657	0.13646141	0.03409021	0.54624823
cg18978493	0.00489187	7.45381552	1.83983038	30.1980913
cg27427527	0.00490014	38.3272325	3.02274403	485.974576
cg01184452	0.0049004	19.3480265	2.45658588	152.384712
cg13929970	0.00490306	16.7911339	2.35290436	119.8273
cg08290108	0.00490419	5439.21992	13.5842013	2177905.99
cg20329085	0.00490436	17.7093904	2.39106919	131.164129
cg03395898	0.00490542	34.0380739	2.91496466	397.462958
cg11300971	0.00490989	75.087585	3.70432761	1522.04287
cg26931437	0.0049102	15104220.7	150.24785	1.5184E+12
cg14626797	0.00491294	2461.17766	10.6682708	567795.438
cg25410636	0.00491366	64020.0243	28.6468187	143072205
cg12691534	0.00938205	11.0289977	1.80291832	67.4677212
cg26220419	0.0093842	5105045.34	44.348165	5.8766E+11
cg11441617	0.00938815	6253460.76	46.5827238	8.3949E+11
cg20329085	0.00939251	22.740724	2.15272338	240.226186
cg26443922	0.00939268	1155273.99	30.7538201	4.3398E+10
cg19305681	0.00939428	11.6894413	1.82826379	74.7392355
cg20359237	0.00940507	172.559222	3.53747335	8417.50092
cg17727579	0.00941038	0.05026413	0.00526027	0.48029489
cg18252633	0.00941216	10.345729	1.77350322	60.3517977
cg26126052	0.00941548	3560.59934	7.42595354	1707237.68
cg15676837	0.00942128	29.8316852	2.29861655	387.158719
cg24832710	0.00942842	580.779609	4.75656288	70913.591
cg22852309	0.00944984	31397839.9	68.4445971	1.4403E+13
cg11276456	0.00946586	5.0629E+11	730.139769	3.51E+20
cg18768283	0.00946604	2631.11504	6.86608113	1008255.83
cg25257905	0.00946724	0.13662124	0.03037483	0.61450103
cg02268171	0.0094701	19.6009714	2.07056889	185.551943
cg09597785	0.00947917	181989.591	19.3240138	1713940569
cg14679255	0.00949323	18.4580585	2.03891167	167.098912
cg05975928	0.0094945	0.08250523	0.01252308	0.54356535
cg03791450	0.00949511	8.52629515	1.68817375	43.0629306
cg06787669	0.00949531	13.19506	1.87824889	92.6978368
cg09488991	0.00950169	8795357803	268.556847	2.8805E+17
cg12179176	0.00950428	19.482499	2.06528649	183.784559
cg24050414	0.0095122	1726.90081	6.1715388	483216.019
cg10802521	0.00951339	34941328.6	69.4458428	1.7581E+13
cg01421695	0.0095151	683.745815	4.92114219	94999.9658
cg26794902	0.00952428	7.0882E+10	444.454587	1.1304E+19
cg03891929	0.00952537	703322.46	26.7176423	1.8514E+10
cg15448245	0.00952838	99.6498707	3.07324058	3231.14851

cg17188384	0.0049144	12.7640414	2.16409668	75.2834915
cg01933251	0.00491741	15434222.9	150.943526	1.5782E+12
cg25168729	0.00491832	6229965637	930.129253	4.1728E+16
cg06849504	0.00492269	16.2537757	2.32773727	113.494434
cg27225220	0.00492586	8.06205263	1.88199164	34.5361217
cg10090217	0.00492756	76106.3438	30.1124274	192351666
cg00636194	0.0049294	130.347057	4.37191737	3886.24802
cg01381679	0.00493299	227986.268	41.9390975	1239362352
cg06323837	0.00493967	7828.80142	15.0927367	4060902.48
cg16215203	0.0049406	3.99721529	1.52112756	10.5038726
cg22821834	0.0049423	11.6893578	2.10482049	64.9181655
cg10029130	0.00494884	940.474136	7.93786944	111426.826
cg10129408	0.00495037	8.81222094	1.93174466	40.1995353
cg21163128	0.00495136	8.29197477	1.89643915	36.2557615
cg11683764	0.00495446	1119.74646	8.3628679	149928.488
cg20748955	0.00495595	17.4515391	2.37478063	128.246042
cg15048078	0.00495791	1243739.12	69.7124749	2.219E+10
cg22505746	0.00496239	286607.527	44.6813627	1838437093
cg22818988	0.00496803	404834285	399.769645	4.0996E+14
cg08051964	0.00497479	0.06857612	0.01056869	0.44496372
cg05319236	0.00498596	58317667.9	221.416526	1.536E+13
cg06549777	0.00499015	1728132902	615.254048	4.854E+15
cg24797508	0.00499288	19.6308806	2.45648436	156.87927
cg04914105	0.00499393	240.677884	5.2344404	11066.2916
cg13113115	0.0049941	1049.09031	8.1633197	134821.436
cg20929545	0.00499418	8.70191405	1.92146384	39.4091768
cg00089453	0.0049958	43.3006953	3.11853217	601.228435
cg21649629	0.00499587	6.53035031	1.76187386	24.2046132
cg02590886	0.00499631	120.282708	4.24482231	3408.37116
cg17719053	0.00500544	26.1650003	2.67730384	255.707713

cg22507887	0.00954252	0.22313146	0.07177373	0.69367509
cg13813874	0.00954367	3044626.72	38.0922405	2.4335E+11
cg21746532	0.00954435	0.10020992	0.01759606	0.57069751
cg02494781	0.00954452	4057753.46	40.850214	4.0307E+11
cg05858889	0.00954529	10.9209876	1.79116133	66.5869506
cg03640883	0.00954606	0.08209018	0.01239585	0.54363348
cg18306747	0.00954931	452.545275	4.43971112	46128.5026
cg22583148	0.00955818	10.2421045	1.76279696	59.5081039
cg22492271	0.00956314	0.14737967	0.03463054	0.62721424
cg27172150	0.00957903	35.8202077	2.38976595	536.909181
cg01734112	0.00958561	11.0691628	1.79522769	68.2511556
cg23818888	0.00958634	15.9344015	1.96163661	129.435366
cg22681784	0.00959183	44.8079981	2.52234111	795.989364
cg19711258	0.0095954	425.352599	4.36036218	41493.0747
cg24495257	0.0096042	1.155E+11	490.106185	2.7221E+19
cg04229059	0.00960904	253.216319	3.84041562	16695.7201
cg18686576	0.00960934	39.4972032	2.44444143	638.194492
cg08051964	0.00962029	0.05516421	0.00615342	0.49453637
cg26462404	0.00962066	2.9814E+10	351.112362	2.5315E+18
cg06769202	0.00963431	11.991566	1.82822502	78.6542434
cg22037648	0.00963749	16.6800374	1.98060455	140.474103
cg08359167	0.00964271	1243.16636	5.64033912	274001.714
cg03383158	0.00964382	727.85792	4.95255077	106970.565
cg13254588	0.00964556	11026242	51.2372744	2.3728E+12
cg07048608	0.00965191	0.14517537	0.03366551	0.62603799
cg19866195	0.009656	16.7776859	1.98233301	141.999726
cg05529506	0.00966079	11.5660097	1.81102449	73.8656944
cg13168437	0.00966561	5688.75554	8.14261773	3974390.13
cg07315894	0.00967339	19.2486222	2.04843239	180.874633
cg24676409	0.00967603	5.7287E+14	3783.58246	8.67E+25

cg02776498	0.00500789	19.5663947	2.45229106	156.116787	cg13179508	0.00967718	7266437454	245.8292	2.1479E+17
cg16320779	0.00501121	6343.87126	14.0198064	2870560.51	cg21784917	0.0096777	11.3742643	1.80292121	71.7579268
cg06250108	0.00501842	387.358259	6.02897636	24887.545	cg26699183	0.00968946	2.2021E+14	2986.98009	1.62E+25
cg19789653	0.00502292	13.9287593	2.21195015	87.7100848	cg25261228	0.00970082	53.99077	2.62743099	1109.44997
cg09915099	0.00502672	1459.7708	8.98511815	237162.246	cg14328907	0.00970832	3081376984	198.259172	4.7891E+16
cg03893150	0.00503	9.60262848	1.97688486	46.6443319	cg26542888	0.009717	128.517271	3.2388515	5099.55113
cg14611402	0.00503122	20.2460672	2.47490235	165.624005	cg16201038	0.00971732	6.3377501	1.56341444	25.6918929
cg02944245	0.00503776	12213362	136.236915	1.0949E+12	cg08695855	0.00972012	13.8815822	1.88992815	101.960662
cg06798491	0.00504084	1075.26989	8.18150235	141319.44	cg27648075	0.0097215	8.03163077	1.65550223	38.9652708
cg24654028	0.00504346	40.6990855	3.05211952	542.709927	cg11279444	0.00972567	5.42975531	1.50576927	19.5795221
cg10118908	0.00504528	0.00039175	1.63E-06	0.09426447	cg04567169	0.00972576	3.99E+10	366.929909	4.3388E+18
cg04848925	0.00505247	15.8489317	2.29675586	109.366711	cg04680150	0.00972874	319.174498	4.03362754	25255.7677
cg03791450	0.00505996	7.5159361	1.83444358	30.7936947	cg11637682	0.00972989	15.1438853	1.92961996	118.851
cg13344237	0.00506004	4552.90726	12.6021421	1644876.28	cg25818813	0.00973097	8118902.42	46.8995068	1.4055E+12
cg26419426	0.00506241	1237264.45	67.9884787	2.2516E+10	cg10962407	0.00973753	20138931.9	58.3573725	6.9499E+12
cg10357841	0.00506545	3978.74083	12.0927407	1309081.13	cg18797229	0.00973808	481200.212	23.6558441	9788432942
cg08973102	0.00507851	16654.429	18.5624091	14942565.1	cg02764409	0.00974627	2389975196	184.849798	3.0901E+16
cg09920051	0.00507865	7.6843E+15	59406.4799	9.94E+26	cg14125530	0.00974868	19.6420285	2.05371042	187.859632
cg26668837	0.00507958	12.7645156	2.14957431	75.7977331	cg11404945	0.00975216	153.863192	3.37684427	7010.65255
cg10147797	0.00508439	4.66438243	1.58825606	13.6983349	cg15014976	0.00975372	0.11642113	0.02278965	0.59473846
cg03918756	0.00508705	0.18722656	0.05798306	0.60455212	cg11391462	0.00975511	0.07132058	0.00962742	0.52834753
cg02535674	0.00509331	37.2546407	2.96343509	468.344408	cg24952959	0.00975841	0.00039976	1.06E-06	0.15102892
cg19486585	0.00510251	198505423	309.352747	1.2738E+14	cg15746396	0.00976146	4278.51208	7.53589806	2429128.61
cg09067715	0.00510313	0.00023476	6.77E-07	0.08141971	cg08021273	0.00976829	3428.50761	7.13921954	1646491.51
cg24421668	0.00510423	19.3563404	2.4332461	153.978635	cg21789849	0.00976953	36309.8805	12.62082	104462897
cg08381955	0.00510448	66.4921186	3.52387913	1254.6406	cg18226096	0.00977643	111.166565	3.11806639	3963.3554
cg15837832	0.00510497	3682.56273	11.7540953	1153748.36	cg14483317	0.00978111	756443179	138.947493	4.1181E+15
cg07604732	0.00510524	24.9444979	2.62555565	236.989063	cg24475782	0.00978474	9.40776713	1.71755178	51.5303723
cg21339084	0.00511325	29.4840405	2.75946964	315.027436	cg07138603	0.00978624	6.56997723	1.57496819	27.4066492
cg24234651	0.00511351	5.74430062	1.68943704	19.5313521	cg01410292	0.00978637	184129.544	18.6391543	1818949955

cg19651192	0.00511797	4921.74775	12.8007046	1892364.66
cg08632164	0.00512112	3.77296889	1.48906455	9.55989065
cg03867797	0.0051275	30312.2552	22.0457163	41678519.5
cg09477740	0.00512902	27.0926301	2.68812774	273.056446
cg04528624	0.00513154	3.7689E+10	1477.24718	9.6158E+17
cg10663408	0.0051379	0.00265345	4.16E-05	0.16913475
cg01176915	0.00513972	2561.1316	10.4952051	624989.696
cg18789887	0.00514103	10.39559	2.01639089	53.5949113
cg26202340	0.00514283	19.2098499	2.42334304	152.276557
cg21830368	0.00514332	9.77490252	1.97938668	48.2718814
cg15103181	0.00515065	58.5827538	3.38243334	1014.63612
cg06763124	0.00515886	0.01655592	0.00093515	0.29310656
cg08788576	0.00516037	2.2124E+11	2481.18711	1.9728E+19
cg24073738	0.00517384	61853009.6	213.657681	1.7906E+13
cg15737290	0.00517568	14.1118057	2.20651611	90.2522578
cg02153089	0.00517606	92760088.6	241.028226	3.5699E+13
cg04466743	0.00518026	18.1791654	2.3795918	138.881826
cg08296969	0.00519211	132720.114	33.9125566	519413177
cg27559724	0.00519313	1401441.01	68.5569107	2.8648E+10
cg24568647	0.00519519	1.1865E+11	2030.74113	6.9326E+18
cg24363820	0.00519794	10.255337	2.00403905	52.4799841
cg00142642	0.00520061	28.335725	2.71430691	295.807858
cg13050981	0.00520298	17.9529711	2.36827305	136.0946
cg02611419	0.00520372	0.11389084	0.02481184	0.52277956
cg23264429	0.00520707	0.16724048	0.04769913	0.58637079
cg27639662	0.00520928	19.4682842	2.42555891	156.258455
cg03232620	0.00521098	5.23680417	1.63905304	16.7316842
cg26860994	0.00521153	4.7801E+10	1538.16019	1.4855E+18
cg02801583	0.00521411	81221.166	29.172016	226137192
cg16936060	0.00521638	0.04981557	0.00607253	0.40865866
cg08958015	0.00979059	7.66688528	1.63460116	35.9605336
cg21126828	0.00979954	7529.70909	8.6080083	6586485.17
cg15087448	0.00980006	29.0714637	2.2537923	374.990189
cg17873465	0.00980034	9.9376E+10	448.697737	2.201E+19
cg03045231	0.00980242	0.07872985	0.01144055	0.54179116
cg10602180	0.00981532	12.6990063	1.84501119	87.4058449
cg06250108	0.00982597	331.541263	4.04716196	27159.6764
cg02528389	0.00982736	2230293219	178.53048	2.7862E+16
cg15728769	0.00982829	7339.71235	8.53252532	6313649.87
cg27011534	0.00983526	2.31E-09	6.39E-16	0.00832395
cg12938159	0.00984001	13.2243557	1.86189747	93.9276121
cg03107888	0.00984409	14.0889054	1.89028941	105.008923
cg00016066	0.00984619	3845510	38.4431072	3.8467E+11
cg05973813	0.00986279	2.4755E+13	1664.16458	3.68E+23
cg08867933	0.00986415	0.11382968	0.02185093	0.59298154
cg03278488	0.00986758	117.236093	3.14420112	4371.31751
cg08074851	0.00986782	8.6721252	1.68101687	44.7382516
cg02155796	0.00987231	1564700395	162.320776	1.5083E+16
cg13021277	0.00989278	14221.9247	9.94285873	20342554.1
cg18975124	0.00989661	490466699	122.202514	1.9685E+15
cg25189267	0.00989709	4292.54735	7.4540321	2471945.73
cg08427573	0.00990111	5824.16087	8.01788136	4230650.01
cg03277051	0.00990167	10289.6994	9.19144296	11519183.1
cg11399508	0.00990678	12.7121833	1.84105573	87.7755092
cg21841239	0.00991632	60.0602862	2.67161446	1350.20904
cg17903246	0.00991667	547613.904	23.8194698	1.259E+10
cg23400797	0.00991943	1205220.55	28.7715995	5.0486E+10
cg27233566	0.00992108	463.490627	4.36102856	49259.8382
cg26836793	0.0099228	1211205968	150.981294	9.7166E+15
cg26328687	0.00992284	45.3640626	2.49700091	824.147947

cg20807701	0.00521838	2367.84617	10.1537962	552177.274
cg27614280	0.00521928	2.19E-05	1.17E-08	0.04071756
cg08391942	0.00522173	9323743134	940.986682	9.2384E+16
cg10497871	0.00522178	9.30478401	1.94504967	44.5124907
cg21734015	0.00522274	4203.09996	12.0428302	1466935.01
cg10732920	0.00523036	2937924.92	84.7859524	1.018E+11
cg25121621	0.00523067	4837.31287	12.5451868	1865224.97
cg16167741	0.00523576	6.38218689	1.73747625	23.4433762
cg14039939	0.00523749	27528.4806	21.0448215	36009677.8
cg24475782	0.0052379	7.7611785	1.841666	32.7072834
cg06794000	0.00523949	2973.32076	10.8389923	815632.681
cg04022379	0.00524508	5.65200586	1.67526315	19.0687476
cg13741249	0.00525428	416379247	368.585188	4.7037E+14
cg27323009	0.00525976	23.780613	2.56842645	220.180552
cg13813247	0.00526236	0.00379325	7.56E-05	0.19029611
cg17603057	0.00526308	263732.119	41.0644147	1693793306
cg20935945	0.00526326	1552.2386	8.90625466	270533.998
cg09244244	0.0052635	19.7738484	2.4307414	160.858362
cg25308662	0.00526411	0.01951946	0.00122943	0.30990615
cg17736030	0.00526835	838.056134	7.40962377	94787.2802
cg23089445	0.00526868	30.9126958	2.77568814	344.2731
cg10962407	0.00527347	959124.954	60.1706929	1.5289E+10
cg21370255	0.00527987	5.66023311	1.67442515	19.1338734
cg18076651	0.00528032	727.580758	7.09577779	74604.0497
cg00671759	0.00528053	38252.5045	23.0499791	63481797.2
cg16368146	0.00528319	11.2228796	2.05215056	61.3761134
cg03111114	0.00528523	28.4514499	2.70568824	299.178962
cg24330818	0.00528824	148.640165	4.42215598	4996.18257
cg09565670	0.00528919	10.5077437	2.01193075	54.8789648
cg03774026	0.00529261	479.31171	6.26024649	36698.19

cg07235774	0.00992694	97.4141593	2.9988515	3164.38424
cg27124616	0.00992893	151.756875	3.33492704	6905.74302
cg14719951	0.00993682	6.57885276	1.57088298	27.5522137
cg04377289	0.00994388	8.96218394	1.69146619	47.4858685
cg11831182	0.00994897	3629189.64	37.3109264	3.5301E+11
cg01268571	0.0099606	10.5622048	1.75867696	63.4341456
cg11686214	0.00996872	1.5181E+10	273.870138	8.4152E+17
cg18301048	0.0099693	3396.40521	7.00412658	1646967.43
cg00531137	0.00997437	7.64940809	1.62742732	35.9545668
cg20666271	0.00997838	1.6942E+11	486.697225	5.8975E+19
cg08575233	0.00998331	234.700904	3.69130724	14922.766
cg26502485	0.00999259	19802.1511	10.6572125	36794348.2
cg02376018	0.01000047	1147.38668	5.38943928	244273.315
cg23025703	0.01000836	47.7808253	2.5197424	906.047884
cg10453019	0.01001224	15.2137918	1.91654118	120.769365
cg03629151	0.01001275	16.4304273	1.9520729	138.293473
cg00054210	0.01001487	14.1876132	1.88468905	106.801898
cg15535174	0.01001512	102.175107	3.02073227	3456.03368
cg09424275	0.01001706	44131842	67.0630156	2.9042E+13
cg15227302	0.01002162	7910085.6	44.4418018	1.4079E+12
cg14183329	0.01003318	9.00266874	1.68990134	47.9602225
cg12578480	0.01003619	31511.2299	11.8548167	83759845.5
cg17013432	0.01003732	670727.241	24.5965199	1.829E+10
cg13116840	0.01004147	29334824.6	60.5685434	1.4208E+13
cg08247347	0.01004426	1.0466E+10	246.117466	4.4503E+17
cg20632873	0.01005211	2746.96305	6.61274264	1141100.82
cg24700222	0.01006105	13.8102204	1.87027465	101.975497
cg17613375	0.01006427	2175.2313	6.24871721	757216.408
cg27219362	0.01006604	0.11699516	0.0228299	0.59955894
cg07703790	0.01006634	184.030272	3.46728787	9767.61727

cg11577646	0.00530149	2656.3987	10.402039	678372.197
cg22303873	0.00530645	17523198.9	141.585471	2.1687E+12
cg09797971	0.00530797	85.1553122	3.74230165	1937.69179
cg00076353	0.00531015	405314.514	46.2313527	3553429555
cg27310963	0.00531135	253.4081	5.17180883	12416.4808
cg22761176	0.00531175	5.54303479	1.66263918	18.479797
cg01350077	0.00531406	4.74880315	1.58794342	14.2014703
cg26044340	0.00531698	7.16613441	1.79407933	28.6238638
cg23600533	0.00531841	17412.9571	18.136907	16717904.3
cg27072683	0.00532086	674.72439	6.91023641	65880.9591
cg25413906	0.00532555	1095645839	480.517813	2.4982E+15
cg10328157	0.00532991	6.27259798	1.72392171	22.8232438
cg04064094	0.00533557	22865.4444	19.6121158	26658446.9
cg14266050	0.00534087	857.433957	7.40413451	99294.9262
cg08877188	0.00534113	19.4503063	2.41026551	156.959643
cg07352001	0.00534527	2.6868E+17	146314.439	4.93E+29
cg26332715	0.00534763	586372873	396.458578	8.6726E+14
cg09761352	0.00534787	33135091.9	169.197838	6.4891E+12
cg16976499	0.00534875	66.2685042	3.46466489	1267.51498
cg05691004	0.00534953	0.0923173	0.01726414	0.49365234
cg15997906	0.00535036	5.16011262	1.62609682	16.3746475
cg18191162	0.00535085	55.2050224	3.28170609	928.661621
cg05206690	0.00535252	276943104	316.978503	2.4196E+14
cg00811132	0.00535301	11.166956	2.04380404	61.0141209
cg23971226	0.00535827	0.19305593	0.0606621	0.61439672
cg19815813	0.00535844	5.51556679	1.65815983	18.3465288
cg06263843	0.00536601	80.8850976	3.67118095	1782.09658
cg21129181	0.0053687	7.67323832	1.82790216	32.2110163
cg18315708	0.005369	34.5643999	2.8538145	418.631885
cg19116747	0.00537892	6867233763	813.10144	5.7999E+16
cg13411897	0.01007482	12944.2511	9.55075336	17543499.4
cg24309428	0.01007844	9.65921874	1.71674755	54.3472491
cg23782001	0.01008223	20.1092358	2.04428818	197.810352
cg02492791	0.01008904	15.4424327	1.91927244	124.249545
cg08854306	0.01009284	228646.656	18.8919284	2767281987
cg11970289	0.0100934	9.13559979	1.69352851	49.2812392
cg24168884	0.01009537	43.4664761	2.45517082	769.532828
cg07138491	0.01009626	27.5841592	2.20313316	345.365342
cg05021798	0.01009742	173.007759	3.41104583	8774.92892
cg02281514	0.01010535	101.49904	3.00317694	3430.3857
cg14520423	0.01010995	360.699905	4.06009477	32044.6761
cg08144943	0.01011034	48.9045837	2.52357244	947.727227
cg02619315	0.0101153	3364.70856	6.9045097	1639691.18
cg20314660	0.010116	2.6171E+11	520.53914	1.32E+20
cg06117133	0.0101203	3241374.94	35.3757267	2.97E+11
cg22614265	0.01012478	1733910494	157.494601	1.9089E+16
cg03363417	0.01013115	0.04257025	0.00383845	0.47212452
cg22755785	0.01013184	0.00023964	4.17E-07	0.13779382
cg04903089	0.01013527	0.05160218	0.00538714	0.49428568
cg08263387	0.01013882	2113301.92	31.8661978	1.4015E+11
cg08230658	0.01014465	116.990906	3.10056285	4414.31854
cg26570179	0.01015023	4.98157028	1.46443771	16.9457822
cg20312228	0.01015081	14.2401917	1.87944669	107.895085
cg10056854	0.01015121	1771696.5	30.5032676	1.029E+11
cg05308135	0.01015769	0.07065841	0.00936787	0.5329502
cg10534672	0.01015952	165.420915	3.36398704	8134.41873
cg23356310	0.0101668	0.1028977	0.01816625	0.58283577
cg27018309	0.0101671	0.07038323	0.00930132	0.53259104
cg17742273	0.01018261	0.04285709	0.00387778	0.47365509
cg18353597	0.01018265	428204864	111.625294	1.6426E+15

cg00144465	0.00538063	248422790	304.397678	2.0274E+14
cg24577131	0.00538124	6.93829397	1.77357298	27.1429051
cg26900458	0.00538436	320.87371	5.51154899	18680.7625
cg11904590	0.00538718	122452.721	31.9594893	469177360
cg00725244	0.00538923	257.16446	5.16018818	12816.1139
cg23611233	0.00539192	10728358.7	119.823517	9.6056E+11
cg19896655	0.00539245	8170478733	851.971999	7.8356E+16
cg03071876	0.00539926	15.5029596	2.24805561	106.910948
cg09622420	0.00539973	2750.16621	10.3853271	728278.859
cg09375033	0.00540029	0.1379163	0.03415779	0.55685405
cg01466825	0.00540214	698.380901	6.92467284	70434.502
cg09988062	0.00540321	720.439787	6.98784664	74276.5996
cg23451876	0.00540601	63.0340562	3.40133438	1168.15691
cg18132916	0.00540678	5.937171	1.69250498	20.8271172
cg17742273	0.00540972	0.04856607	0.00576354	0.40923885
cg14273450	0.00541386	6.18258368	1.71254245	22.3202298
cg12045829	0.00541402	9.90278404	1.96813911	49.8263212
cg08718695	0.00541459	4.9478E+15	43108.3396	5.68E+26
cg07703790	0.00541551	98.3078812	3.87600047	2493.40514
cg13605674	0.00541654	0.07739506	0.01275136	0.46975332
cg00924576	0.00541669	6.3946E+13	11923.8487	3.43E+23
cg12265604	0.00541876	11545710.9	121.642892	1.0959E+12
cg25347419	0.00542128	5.50512578	1.65452541	18.3172828
cg02926368	0.00542678	645.919165	6.75034251	61805.9849
cg26918305	0.00543091	389.200769	5.81081641	26068.1508
cg27464144	0.00543423	12.7559197	2.11927508	76.777899
cg23179456	0.00543764	3.01546594	1.38479607	6.56633497
cg22510727	0.00544015	18.3465785	2.35851226	142.715791
cg16647169	0.0054411	2.1935E+10	1120.94239	4.2924E+17
cg02673418	0.00544941	514.184468	6.29742435	41983.1431

cg26605335	0.01018479	15.3077777	1.91021151	122.671263
cg11267546	0.01019353	10.0605308	1.72882272	58.5452049
cg00465883	0.0101964	0.06133733	0.00729256	0.51590493
cg09015232	0.01019823	0.08621251	0.01328915	0.55929814
cg17665193	0.01019843	0.02195046	0.00119151	0.40438069
cg09201719	0.01021147	26.8130315	2.17993554	329.798128
cg27392771	0.0102152	32.7187979	2.28492078	468.515035
cg19870284	0.01021947	0.1187422	0.02335641	0.6036764
cg17328794	0.01022519	331304.317	20.2881805	5410172191
cg10296301	0.01022648	0.06067556	0.00714835	0.51501721
cg14373380	0.01023011	27896.0772	11.2856734	68953894.1
cg14777341	0.01023141	16.1241228	1.93137378	134.612647
cg26738606	0.01023981	16.449495	1.94007094	139.472161
cg05481243	0.01024703	1963.15808	6.01283759	640960.209
cg05966809	0.01025205	114728.577	15.7329935	836626955
cg04100696	0.0102819	6.1204818	1.53415674	24.4175165
cg12619880	0.01028222	44.2762994	2.4484155	800.677291
cg27275781	0.01028229	1770307843	153.008888	2.0482E+16
cg12821724	0.01028279	63.8854827	2.66987485	1528.66899
cg14714537	0.01028637	17.0692962	1.95453073	149.069476
cg05022341	0.01028673	34445.1052	11.7931051	100606690
cg15628498	0.0102925	2609311972	167.321531	4.0691E+16
cg24812936	0.01029411	5302965.4	38.7054352	7.2655E+11
cg15431544	0.01029974	18.9350395	2.00222005	179.069089
cg17327665	0.01030311	425355581	108.796264	1.663E+15
cg26969942	0.01031261	3.7845E+10	313.10034	4.5745E+18
cg10560368	0.010316	69308234.4	70.7326958	6.7912E+13
cg09653271	0.0103242	35041.0211	11.7945137	104105450
cg25062542	0.01032495	10915.795	8.95786353	13301674
cg10315334	0.01032771	35.5003571	2.32013841	543.18973

cg24214699	0.00545061	6.97192978	1.77248176	27.4235854
cg07117311	0.00545147	28.0836309	2.67250649	295.112593
cg13722431	0.00545373	5.34976543	1.6392505	17.4591926
cg15911239	0.00545427	12127.2818	15.9764429	9205488.61
cg27201679	0.00545516	6.14267993	1.70733016	22.1003047
cg23217940	0.00545585	0.08065344	0.01365956	0.47622167
cg10879207	0.00545888	10.263365	1.98589149	53.0425063
cg18773844	0.00547076	205.974282	4.80066837	8837.39541
cg27090191	0.0054708	143945532	252.449904	8.2077E+13
cg19337852	0.00547682	979075.839	58.0025668	1.6527E+10
cg27035169	0.00547945	8.47370645	1.87563982	38.2822438
cg25345422	0.00548366	159.564753	4.44862751	5723.31807
cg17162095	0.0054854	0.00256685	3.81E-05	0.17288994
cg13711394	0.00548879	9.96016916	1.96638331	50.4504738
cg06469542	0.00549536	16.6234799	2.2855415	120.907927
cg23813257	0.00550146	345.495862	5.57532894	21409.9279
cg05987251	0.00551602	6.28052109	1.71564085	22.9913768
cg04320760	0.00551921	214.609313	4.84009981	9515.74538
cg27334900	0.00552814	23.7013659	2.53288734	221.78434
cg26507725	0.00552965	6.89340395	1.76252163	26.9608142
cg08437570	0.00553117	14.6798487	2.2002982	97.9403418
cg10448562	0.00553612	1933.1603	9.2132669	405622.544
cg26738606	0.00553929	13.8141864	2.16070074	88.3193775
cg06510830	0.00554031	15185.0344	16.8596456	13676756.6
cg03595406	0.00554437	7.52E-09	1.37E-14	0.00413997
cg16101148	0.00555304	29.7288512	2.70369445	326.887751
cg03195164	0.00555361	533.833213	6.30510531	45197.9603
cg11597832	0.00555428	18.4369467	2.35014802	144.638126
cg00350296	0.00556443	12.686897	2.10538987	76.4501425
cg13523649	0.00556992	11.6640067	2.05374639	66.2443288

cg01864807	0.01033076	1327718.22	27.766362	6.3488E+10
cg17987601	0.01033687	14696.5703	9.5976345	22504418
cg22628873	0.01034084	20.1515637	2.02935731	200.105479
cg07361448	0.01035222	4.18E-07	5.57E-12	0.03145391
cg13884871	0.01035649	118383.346	15.6574231	895078105
cg25598759	0.01035982	8029862.35	42.244956	1.5263E+12
cg07470489	0.01036161	30.777844	2.24074386	422.750542
cg22173752	0.01036595	10.2013712	1.7275706	60.2394917
cg24837744	0.01037807	74959888.2	71.2463939	7.8867E+13
cg06487247	0.01037829	91.876119	2.89661901	2914.16344
cg06313718	0.01037954	1534.97373	5.61782366	419405.181
cg00506625	0.01038668	3476683.09	34.5478186	3.4987E+11
cg09303936	0.01039216	24.9781531	2.13116347	292.754704
cg03169836	0.01039239	80.3504282	2.80496135	2301.70419
cg18270343	0.01039244	12.7078384	1.81804214	88.8258603
cg22547764	0.01039297	1.03E-05	1.58E-09	0.0672246
cg02247068	0.01040488	35.9429812	2.32051042	556.730054
cg23666491	0.01041125	2904.50169	6.51079014	1295715.25
cg18270926	0.01041621	31.6447157	2.25120553	444.823014
cg01262913	0.01041854	13.4929335	1.84260215	98.8055152
cg02818811	0.01041873	8.46564883	1.6515081	43.3950098
cg17139983	0.01042109	141651.078	16.2088427	1237906257
cg05159804	0.01042346	14.2895357	1.86735175	109.347814
cg27211696	0.01042467	66.7259119	2.68142897	1660.43828
cg03774463	0.0104276	13.6344037	1.84668657	100.665141
cg06888121	0.01042965	8.60558725	1.65749196	44.6796325
cg01656048	0.01043079	15232.1813	9.59198315	24188881.8
cg01840244	0.01043326	38342.4823	11.9099934	123438015
cg08464513	0.01043455	16.0535409	1.91850995	134.331426
cg09624525	0.01044511	473328.152	21.4507433	1.0444E+10

cg12110327	0.00557631	20.8179408	2.43292019	178.134351
cg13722539	0.00557632	10150.3172	14.9062413	6911798.75
cg05060704	0.00557752	9.67998439	1.94407516	48.1988041
cg12439852	0.00558359	7.99613682	1.83791987	34.7883524
cg00620542	0.00558956	27501.8544	19.9191255	37971144.6
cg14181387	0.00559017	12739.5707	15.900854	10206789
cg14591123	0.00559329	44.4676684	3.03558622	651.397586
cg16492833	0.00559702	183.162782	4.59213624	7305.66406
cg19971773	0.00560322	1482.22943	8.46038125	259681.45
cg01366985	0.00561109	8.00969229	1.8372689	34.9187702
cg18426551	0.00561279	0.11207412	0.02381577	0.52740722
cg10449409	0.00562268	48.629965	3.11084083	760.203954
cg00931235	0.0056232	6.0248E+12	5419.15276	6.70E+21
cg17604407	0.00562406	139867798	239.795402	8.1582E+13
cg14125530	0.00562599	15.8248173	2.24059137	111.767298
cg00202760	0.00562916	32.0822567	2.75393939	373.745044
cg00351047	0.00563239	26.2903151	2.59794772	266.048721
cg01893681	0.0056376	1449.46418	8.37414327	250884.936
cg27410337	0.0056378	14.1922314	2.16943755	92.8440792
cg03408033	0.00564045	31306.143	20.5279944	47743319.3
cg08247347	0.00564309	82151886.2	204.218174	3.3048E+13
cg20801130	0.00564501	74.4702412	3.51832157	1576.26775
cg07036112	0.00564571	20.9272992	2.42904064	180.298281
cg11706780	0.00565539	13.5444338	2.13859924	85.7812357
cg13078331	0.00565605	8395416076	784.69415	8.9822E+16
cg07475692	0.00565625	3041.62887	10.3743675	891765.809
cg00522384	0.00565855	95986.1633	28.3828031	324610065
cg13315345	0.00566162	100.55903	3.83630395	2635.90127
cg23489936	0.00566493	830731406	398.575086	1.7315E+15
cg15777760	0.00567005	316.665991	5.35640709	18721.0098

cg21483820	0.01045516	140618.27	16.1161432	1226937347
cg09001028	0.01045705	3523.4878	6.78780998	1829009.11
cg14671364	0.01047017	78.9821701	2.78422595	2240.54488
cg06644428	0.01047438	7.17057379	1.58661526	32.4068032
cg12489322	0.01047875	3.2867E+15	4316.99212	2.50E+27
cg01184591	0.01048116	0.02779635	0.00178843	0.43201921
cg04608326	0.01048266	13406.8378	9.2633429	19403718.8
cg01763719	0.01048634	0.10235508	0.01786695	0.5863654
cg22693994	0.01049275	263.798268	3.68897963	18864.1666
cg19764541	0.01049776	179108433	85.4948022	3.7523E+14
cg24413235	0.01049806	3828.36495	6.89799317	2124730.75
cg06791405	0.0105079	162.486166	3.2907829	8022.94008
cg07065570	0.01051199	203223.708	17.4491519	2366870071
cg00334257	0.01051413	7979968841	207.164133	3.0739E+17
cg22818988	0.01051839	546070571	110.530594	2.6978E+15
cg17265380	0.0105187	9.11022809	1.67653798	49.5045485
cg18365406	0.01053302	7.44685971	1.59886688	34.6843882
cg17964980	0.0105392	14949.4346	9.45167325	23645082.7
cg08724866	0.01054108	84401.5065	14.1604616	503063706
cg09577511	0.01054337	8.8030601	1.66226068	46.6195633
cg25464815	0.01055159	163634667	82.8663554	3.2313E+14
cg21305265	0.01056163	69.506276	2.69171006	1794.8153
cg06958211	0.01056939	106.35422	2.9716597	3806.36456
cg17324226	0.01058212	28488.0922	10.9410553	74176702.1
cg07235053	0.01058337	0.09538974	0.0157407	0.57806849
cg06759097	0.01058352	60.319795	2.60178339	1398.45526
cg09369294	0.01058449	1.4315E+10	233.679198	8.7691E+17
cg26465611	0.01058641	19.8689538	2.00792287	196.60881
cg01208563	0.01059213	7.0266E+10	338.029447	1.4606E+19
cg01554060	0.01059784	7.0273314	1.57537638	31.3470402

cg27217148	0.00567007	34.1160284	2.79785463	415.998524
cg27635271	0.00567079	16.9927544	2.28340553	126.457478
cg12210363	0.00567982	53705753.3	178.660568	1.6144E+13
cg13610108	0.00568022	357725794	310.385966	4.1229E+14
cg00295485	0.00568815	9.37921027	1.91914348	45.8379408
cg11154838	0.00568859	20798.519	18.0900317	23912528.1
cg00530939	0.00568999	4194356.23	84.8060373	2.0745E+11
cg23464435	0.00569257	3937134.3	83.2095989	1.8629E+11
cg02068689	0.00569838	128.425307	4.10912649	4013.76293
cg19481816	0.00569906	540.221319	6.24197705	46754.269
cg19663209	0.00569915	27096.7936	19.5077478	37638185
cg14896306	0.00570155	217073918	266.739837	1.7666E+14
cg25076459	0.00570183	8.08533368	1.83717706	35.5831901
cg10983208	0.00570433	13.1685822	2.11718231	81.9067673
cg03602233	0.00571292	14.5163321	2.17733596	96.7806081
cg25365404	0.00571824	36671.208	21.24101	63310430.8
cg24516766	0.00572629	3.942E+11	2346.04527	6.6236E+19
cg16213375	0.0057317	151.990649	4.3050707	5366.03437
cg04793272	0.00573612	31.4522048	2.72321844	363.261783
cg21291881	0.00573657	0.00039646	1.53E-06	0.10274614
cg11365324	0.00573847	2777236.91	74.4238365	1.0364E+11
cg25353054	0.00574225	2310485006	524.094797	1.0186E+16
cg23378074	0.00575175	0.11209471	0.02371691	0.52980014
cg01395659	0.00575736	877.449012	7.14697382	107726.261
cg23413007	0.00575898	69501.6572	25.4115776	190089747
cg10932427	0.00575964	7439919.49	98.605054	5.6135E+11
cg03517466	0.00576212	27.4908276	2.61548374	288.950602
cg02425595	0.00576405	255.219322	4.99173013	13048.9632
cg08446255	0.00576427	564.65777	6.28478098	50731.8231
cg22742344	0.00576659	97.8600549	3.77924976	2533.99245

cg24080793	0.01059841	5.51600116	1.48890237	20.4353686
cg05845178	0.01060184	11.8786145	1.78025542	79.2591231
cg15773138	0.0106174	220.346114	3.51333508	13819.4647
cg07294234	0.01062028	6982.20643	7.85549807	6205998.17
cg11349878	0.01062152	0.0007661	3.12E-06	0.18813212
cg00937681	0.0106304	190.944911	3.39579657	10736.7912
cg20976433	0.0106341	1.2587E+13	1119.10991	1.42E+23
cg11753867	0.01063958	3088.66398	6.48666537	1470685.57
cg09146903	0.01064146	19.3519346	1.99241276	187.961741
cg06997416	0.0106415	1.9407E+13	1235.02357	3.05E+23
cg12253571	0.01064512	17163.1103	9.66276974	30485291.7
cg09168548	0.01064777	1233104.92	26.1096676	5.8237E+10
cg10391522	0.01064814	18.0327516	1.95957828	165.943935
cg24247086	0.01065636	144541.22	15.8419364	1318788539
cg11044163	0.01065976	13.3636943	1.82710875	97.7436764
cg17719053	0.01066392	26.1906416	2.13621062	321.105841
cg12891177	0.01067839	71.0328362	2.69217666	1874.19492
cg10029130	0.01068014	992.835825	4.96751005	198434.017
cg26031954	0.01068052	6.87915411	1.56511475	30.235969
cg24462260	0.01068893	7084.06613	7.83506722	6405049.45
cg14344110	0.01068912	11649.9347	8.79427412	15432880.1
cg11399754	0.01069434	3.9228E+13	1431.32085	1.08E+24
cg18456691	0.01069617	1.9732E+10	245.373185	1.5868E+18
cg05497345	0.01070674	5.90297289	1.50976542	23.0798034
cg25562664	0.01070888	14.2954148	1.85358737	110.250473
cg04377145	0.01071056	13.6417525	1.83348958	101.499028
cg18352648	0.01071467	0.09393324	0.01527189	0.57775776
cg24372325	0.01071515	75667813.9	67.2161684	8.5182E+13
cg21370255	0.01072892	6.59440412	1.54843614	28.0839258
cg22652406	0.01073261	206.422017	3.43954377	12388.2851

cg24341129	0.00577106	16.5369771	2.25606922	121.215967
cg27626318	0.00577722	162387.646	32.4036786	813788700
cg00224508	0.00577789	28829424.5	145.41601	5.7156E+12
cg17095910	0.0057844	875.945342	7.12439393	107697.616
cg18298494	0.00578896	17.5109311	2.29211462	133.777214
cg02767841	0.00579362	1607.14589	8.48603763	304372.669
cg05896563	0.00580016	2.6187E+14	14967.5451	4.58E+24
cg01409498	0.00580033	11.2489929	2.01546887	62.7843196
cg02776641	0.00580056	351435.549	40.3570047	3060359560
cg01966160	0.00580497	11.1199935	2.00842275	61.5678426
cg11298343	0.00581783	16.3506013	2.24440415	119.114983
cg09158821	0.00581941	0.00834326	0.00027799	0.25040681
cg07273992	0.00581978	31.5883414	2.71520393	367.494796
cg19252931	0.00582231	0.149085	0.03854405	0.57664767
cg25994725	0.00582715	38.0794458	2.86493004	506.135985
cg03969696	0.00584377	8.23925065	1.83925579	36.9090867
cg15617292	0.00584614	6544194.47	93.1497444	4.5976E+11
cg11670109	0.005847	325.438965	5.31925964	19910.7633
cg01819502	0.00585097	19.7412773	2.36678678	164.661232
cg05460949	0.00585294	93366.654	27.2558836	319833039
cg15238694	0.00585324	0.06955796	0.01044804	0.46308304
cg03811891	0.00585327	7.72935842	1.80511076	33.0965738
cg06363275	0.00585563	490227.172	43.978953	5464492994
cg00256060	0.00586825	120.886109	3.98987862	3662.63056
cg09527362	0.0058706	129.798975	4.07196722	4137.50233
cg21644628	0.00587075	0.00130227	1.15E-05	0.14703702
cg00500299	0.00587281	5090267.95	86.1151423	3.0089E+11
cg07343367	0.00587446	216971.048	34.6395612	1359036717
cg19160353	0.00587616	980723.481	53.5103598	1.7974E+10
cg03611151	0.00588221	11.3150586	2.0131056	63.5985275
cg00393798	0.01073343	9.4293955	1.68210231	52.8585561
cg17867333	0.01073755	0.13765774	0.03000291	0.63159374
cg11206041	0.01074718	8.12652989	1.62466171	40.6487624
cg16907298	0.01076514	1.4457E+10	224.706718	9.3013E+17
cg06537680	0.0107753	3.324E+11	463.042556	2.39E+20
cg06508783	0.01078344	10.9651596	1.73993285	69.1030835
cg26329020	0.0107861	3618.53205	6.65147355	1968552.39
cg23295999	0.01078772	432352.814	20.100643	9299650535
cg08756712	0.01078909	36.7788058	2.3014525	587.750803
cg23652354	0.01079138	5077113.61	35.5093267	7.2592E+11
cg17489534	0.01079386	1540308.99	26.9419608	8.8062E+10
cg18235734	0.01081197	12.2161724	1.78271559	83.712101
cg05094271	0.01081344	266846.217	17.9220965	3973134713
cg06887897	0.01082514	0.12844602	0.02649797	0.62262803
cg09135551	0.01082599	8.61673604	1.6441319	45.1594791
cg11323506	0.0108282	9.70680796	1.68989161	55.7563102
cg02205746	0.01082839	5.08017174	1.45527539	17.7342001
cg13560901	0.01083332	26.2423659	2.12566607	323.974577
cg00108715	0.01083701	201.312855	3.40120942	11915.4279
cg03058232	0.01085831	14.2424615	1.84483939	109.954129
cg01876338	0.01086133	9.53319932	1.6816452	54.0434386
cg06784539	0.01086357	22.8043264	2.0559901	252.93765
cg20801007	0.01086671	3.86269752	1.36539897	10.9275256
cg13051028	0.01086864	149778.101	15.5839944	1439520499
cg06382770	0.01088961	103.605676	2.91094573	3687.50818
cg11521427	0.01089421	13.6606725	1.82552327	102.224921
cg15982308	0.01090057	367.354865	3.89339625	34661.1513
cg01910527	0.01090103	0.13243281	0.02792872	0.62797172
cg02512505	0.01090127	6.89723907	1.55957756	30.5030722
cg13183282	0.01090163	4430221.94	33.8484702	5.7985E+11

cg15821095	0.00588475	20.2985164	2.38240146	172.947244
cg00257202	0.00588604	338595.841	39.3019023	2917088916
cg26532627	0.00589486	10.3024935	1.95857789	54.1930824
cg01270593	0.00589547	13.7102698	2.12666346	88.3879853
cg11216502	0.00590092	6206.25426	12.3823769	3110678.35
cg14666655	0.00590131	20346.5432	17.4322755	23748008.1
cg24448326	0.00590228	62833.904	24.1201364	163684792
cg09942293	0.00590578	38.3559455	2.85909956	514.560101
cg22448601	0.00590597	24603119.3	134.581087	4.4978E+12
cg11276456	0.00590675	4.6886E+11	2299.99578	9.5577E+19
cg07869830	0.00590795	33.4466561	2.74820104	407.058577
cg14836450	0.00591331	12.3955721	2.06443839	74.4271215
cg24258806	0.00591421	27263386.9	138.340821	5.3729E+12
cg06377221	0.00591713	12561209.2	110.598726	1.4266E+12
cg12881470	0.00591878	201.171037	4.60386312	8790.39736
cg25412979	0.00593005	33.1503931	2.73813314	401.349571
cg04567169	0.00593015	5.8919E+10	1255.03814	2.766E+18
cg04228628	0.00593909	24210.9751	18.2281679	32157445.6
cg13062913	0.00593983	30.7347784	2.67789219	352.750051
cg03236948	0.00594224	32.5089864	2.72113388	388.380082
cg04821708	0.00594919	12.1668457	2.05076646	72.1838088
cg16856981	0.00595022	1421619393	427.247641	4.7303E+15
cg25864218	0.00596125	589161.483	45.4532023	7636673228
cg06150468	0.00596674	21.4934302	2.41332453	191.423714
cg10296301	0.005968	0.0747729	0.01177362	0.47487422
cg24371438	0.00597436	11248577.6	105.728169	1.1968E+12
cg11165313	0.00597459	55257.9293	22.9829373	132856767
cg09368716	0.00597739	23.673447	2.48002694	225.978228
cg19902519	0.00597992	179069.816	32.1805958	996438947
cg05552569	0.00598206	62.4931568	3.27598067	1192.12994

cg25463478	0.01090301	4072145.12	33.1915583	4.996E+11
cg07195301	0.01091282	16.8102778	1.9138518	147.652729
cg13955572	0.0109152	944.235682	4.83351798	184457.992
cg24550525	0.01092794	19.0835795	1.96966185	184.896208
cg19585103	0.01093985	5.76751421	1.49570062	22.2398919
cg13175159	0.01094262	29.83331	2.18166392	407.957605
cg27369431	0.01094301	25.50957	2.10455828	309.204154
cg10779981	0.01094322	268975299	86.4043233	8.3732E+14
cg10507121	0.01094427	65434170.3	62.4344942	6.8578E+13
cg19822127	0.01094897	26293606.9	50.5976403	1.3664E+13
cg00202760	0.01095325	49.2186555	2.44660901	990.136158
cg13861524	0.01096211	377.825041	3.90484646	36557.5864
cg08593883	0.01096592	0.11059003	0.02027248	0.60328844
cg16164732	0.0109693	491.89005	4.14684589	58346.9527
cg05212510	0.01097201	0.0169503	0.00073227	0.39236095
cg20556988	0.0109817	0.13691603	0.02957794	0.63378311
cg23584743	0.01098502	5.33808088	1.46827322	19.4072241
cg22093805	0.01098649	274.252922	3.62319781	20759.1938
cg10672880	0.0109921	166072.312	15.7319742	1753118366
cg00092273	0.01099816	130173229	72.414749	2.34E+14
cg27572598	0.01100846	1526491694	127.047926	1.8341E+16
cg24105407	0.01101022	19.4311641	1.97319923	191.349224
cg24005667	0.01101165	0.11405885	0.0213914	0.60816144
cg26354439	0.01101326	0.00500992	8.44E-05	0.29726348
cg07390023	0.01101781	67.5843618	2.62451948	1740.37419
cg14182841	0.0110222	305.11909	3.70560361	25123.4803
cg06945667	0.01102307	1929615.8	27.4856663	1.3547E+11
cg10059484	0.0110258	126.126687	3.02649928	5256.21837
cg21347053	0.01102616	4.90863875	1.43939597	16.7394761
cg14386850	0.01103238	6402698.54	36.1209781	1.1349E+12

cg08504583	0.00598262	0.01036217	0.00039846	0.26947552
cg21682062	0.00599018	1.0274E+14	10453.5772	1.01E+24
cg16854917	0.00599286	6.23032178	1.68995409	22.9692095
cg11819639	0.00599591	568.254287	6.16475424	52380.5042
cg14188862	0.00600154	92.7480734	3.66440639	2347.50304
cg03142071	0.00600292	0.04017534	0.00405621	0.39792261
cg08641278	0.00600358	11.5815036	2.01808034	66.4647601
cg03355690	0.00600362	9.7133436	1.91883755	49.1698966
cg07099000	0.00600397	7.01640559	1.74800297	28.1635376
cg04590284	0.0060041	3613152.7	75.8284601	1.7216E+11
cg27663476	0.00601339	16.6558349	2.23874134	123.916431
cg01126162	0.00602299	17.3427398	2.26393637	132.852949
cg04562522	0.00603268	78723879.2	182.071287	3.4039E+13
cg16002441	0.00603975	93.0846495	3.65923065	2367.91632
cg11505048	0.00604012	24.6500364	2.50183215	242.871727
cg07311956	0.00604128	8.78060619	1.86194559	41.4077864
cg15390960	0.00605238	2006060.89	63.4275753	6.3447E+10
cg04757806	0.00605554	0.06820389	0.01002447	0.46404175
cg04774040	0.00606359	91.5133946	3.63592313	2303.32191
cg23008047	0.00606479	2472.65753	9.32711825	655511.715
cg14425609	0.00607425	2.1579E+17	89467.144	5.20E+29
cg21504505	0.00608672	0.08988885	0.01607363	0.50268705
cg11247103	0.00608715	10776.634	14.1642253	8199236.94
cg03308839	0.00608933	0.00658187	0.00018174	0.23836874
cg02319613	0.00609024	2319049.06	65.5981891	8.1984E+10
cg11007153	0.00609144	0.14322912	0.035723	0.57426813
cg10522845	0.00609458	9.6265084	1.90834977	48.5601043
cg16788391	0.00609648	9253.96427	13.5452537	6322203.84
cg25983070	0.00609832	190097.69	32.0785029	1126521769
cg04832210	0.00609856	15214.9645	15.605743	14833971.4
cg18923197	0.01103276	689511641	105.397017	4.5108E+15
cg18127922	0.01104076	71.9138464	2.65959571	1944.50657
cg20690916	0.01104922	538.623982	4.21368581	68850.8368
cg24165760	0.01105107	83.9115575	2.75394966	2556.74589
cg11229862	0.0110553	8.43619081	1.6284014	43.7050197
cg01553230	0.01107016	1.52E-05	2.92E-09	0.07927181
cg16593556	0.0110786	1898.73716	5.60894748	642759.237
cg08272286	0.0110788	28.2381154	2.14488181	371.764615
cg27201457	0.01108197	0.08288648	0.01213325	0.5662266
cg27303409	0.01108584	5823.70229	7.24105339	4683781.01
cg16257334	0.01108642	837275.998	22.5155827	3.1135E+10
cg16501237	0.01108758	9.19696966	1.65972828	50.9627099
cg11537619	0.01109654	39.937585	2.32016689	687.45516
cg08701937	0.01110796	1475.43814	5.28405363	411978.732
cg03196220	0.0111096	156.441768	3.16659093	7728.82492
cg26280499	0.01111048	0.02476571	0.00142592	0.43013711
cg14123048	0.01111525	1971.56879	5.64233202	688914.351
cg14558114	0.01111898	14.2014982	1.83136745	110.126754
cg14212180	0.01112121	4614.93326	6.84682643	3110581.12
cg02862025	0.01112492	45264.7352	11.5192431	177867264
cg17937683	0.01112893	195573.661	16.0736417	2379613619
cg14334286	0.01112988	8.22152626	1.61639769	41.817366
cg08090734	0.01114734	2.9661E+11	410.183442	2.14E+20
cg18224445	0.01114768	38592.5641	11.0824848	134390981
cg25365260	0.01115035	9.72543358	1.67874265	56.3422025
cg14287235	0.01115103	5.00842943	1.44325785	17.3803768
cg26056577	0.01115132	98.0453165	2.84121865	3383.36653
cg23354735	0.01116198	137.818881	3.06876661	6189.47164
cg00630080	0.01116396	16885503.7	44.1609445	6.4564E+12
cg07141055	0.01118329	36.8558706	2.27122619	598.071299

cg22038796	0.00610186	58.0076978	3.18475141	1056.56378
cg03977294	0.00610195	35822.4837	19.914695	64437358.2
cg14967868	0.00610271	460926.241	41.2682373	5148099698
cg25598759	0.00610615	5897496.92	85.3264016	4.0762E+11
cg08575233	0.00610791	91.3683398	3.62406875	2303.53619
cg23213696	0.00611135	594744487	317.625842	1.1136E+15
cg12457909	0.00611245	7.43783521	1.77203026	31.2192144
cg20601919	0.00611375	5.93600102	1.66160714	21.2060404
cg06705004	0.00611904	0.0140902	0.00066904	0.29674337
cg22586726	0.00611941	5.43637317	1.62025468	18.2404369
cg23192824	0.00612011	725.580075	6.53650393	80542.5117
cg02382426	0.00612051	8.27959648	1.82660074	37.5296671
cg27241190	0.00612394	76521.9174	24.6435328	237612191
cg19488206	0.00612492	45.4406755	2.96680696	695.985622
cg17265380	0.00612618	8.03023466	1.81045004	35.6180327
cg11701250	0.00612993	13364.6484	14.9762607	11926463.6
cg07232612	0.00613515	8.70335614	1.85195147	40.9019401
cg21823502	0.00614265	0.15802676	0.04222645	0.59139363
cg04286878	0.00614446	3.02E-06	3.40E-10	0.02682298
cg12110584	0.00615495	133940562	205.28428	8.7391E+13
cg24602417	0.00616899	15.2405038	2.16954197	107.060826
cg20393324	0.0061704	5.81230672	1.64936233	20.4824064
cg13752005	0.00618211	13196.2114	14.8195677	11750679.9
cg22934035	0.00618247	19.1420945	2.3135242	158.381651
cg00721125	0.00618744	1.8219E+11	1580.76681	2.0997E+19
cg13099644	0.00619046	54362.1443	22.1307604	133535526
cg04377288	0.00619104	8.4396719	1.83274746	38.8640898
cg10191684	0.00619152	414.874668	5.54044853	31066.2556
cg26151000	0.00619262	133.30804	4.01303106	4428.33189
cg26794902	0.00621778	4.0507E+11	1961.3665	8.3655E+19

cg24323726	0.01118659	46.9360326	2.39931435	918.175294
cg22077262	0.01118773	15217.1245	8.93271462	25922789.1
cg01268763	0.01119551	57.7684259	2.51445929	1327.20027
cg01805469	0.01120024	1135312.72	23.7737314	5.4217E+10
cg22198615	0.01120488	19331.6127	9.41707418	39684433
cg05531055	0.01120777	2.1724E+12	635.084604	7.43E+21
cg06602723	0.01121178	10.6082021	1.70992323	65.8122825
cg04882359	0.01121328	75298.5198	12.8145146	442456640
cg15235373	0.01122936	354883.276	18.1880109	6924459187
cg19127747	0.01123257	57.4343428	2.50756107	1315.50285
cg23820828	0.01123502	34.0357078	2.22660606	520.26689
cg06635722	0.01124508	13.0576827	1.79109221	95.1950308
cg16749930	0.0112453	32.7934366	2.20714492	487.240089
cg22928599	0.01124819	78.2088143	2.6878279	2275.67346
cg26985469	0.01125366	28226.621	10.214195	78003418.8
cg04252889	0.01125678	0.05999731	0.00681225	0.52841216
cg03693099	0.01125835	800.839235	4.55261346	140873.695
cg07951810	0.0112595	143104503	70.6111476	2.9002E+14
cg19007249	0.01125964	12.6340065	1.77711774	89.8185403
cg11528307	0.01127852	6701.69113	7.35697837	6104770.43
cg12451530	0.01128555	8.25350777	1.61277578	42.2379796
cg00393376	0.01130133	0.00081384	3.31E-06	0.19991714
cg01659099	0.01131715	6.12106221	1.5064072	24.8720283
cg00891278	0.01131936	13.4515539	1.79990883	100.529704
cg27274446	0.01132563	33.3927576	2.21031777	504.486857
cg11815708	0.01133049	28.2606469	2.1281608	375.283749
cg03811891	0.01133617	9.07242825	1.64595801	50.0067156
cg04672495	0.01133704	19.3170474	1.9524327	191.119685
cg11079583	0.01133868	20.4097376	1.97676706	210.726594
cg06834434	0.01134035	21979.5308	9.57201272	50470030.7

cg24886176	0.00621803	6245.35922	11.9295488	3269571.43
cg00611674	0.00621834	0.00450785	9.41E-05	0.21604776
cg08970083	0.0062198	34.5326822	2.73078216	436.690322
cg01678292	0.00622058	28.8733958	2.59551809	321.19714
cg00122310	0.00622737	15641175.3	109.584939	2.2325E+12
cg14027234	0.00622787	17.1072188	2.23680373	130.83711
cg15684116	0.00622808	3860748.62	73.6914668	2.0227E+11
cg07270460	0.00623005	6.216E+15	30009.6986	1.29E+27
cg00213177	0.00623066	16.9665213	2.23132889	129.009598
cg01306563	0.00623192	47.2372708	2.98260138	748.125368
cg14200826	0.00623587	4.3901E+12	3827.90616	5.03E+21
cg26575389	0.00624173	10.567226	1.95035921	57.2542046
cg04807004	0.00624212	57.7014239	3.15485973	1055.34147
cg12827530	0.00624404	6.82904935	1.72333282	27.0614675
cg05676593	0.00624816	40675.5342	20.2084662	81871581.4
cg07365404	0.00624841	86186180.3	176.840449	4.2004E+13
cg23958684	0.0062509	37.5079071	2.7911947	504.029007
cg20460554	0.00625173	44.3873036	2.92740841	673.02967
cg07105947	0.00625623	8.08693069	1.80724384	36.1868423
cg19961545	0.00625676	9.52969587	1.89319418	47.9692492
cg17831137	0.0062591	7262488.43	87.5541674	6.0241E+11
cg07274204	0.0062608	40.2578114	2.84624942	569.412985
cg11012835	0.00626327	1093306639	361.593568	3.3057E+15
cg20465207	0.00626694	14553.2831	15.0669672	14057112.3
cg09395021	0.00627613	3032.68419	9.65692541	952391.468
cg19872463	0.00627842	5.8082196	1.64469911	20.5116029
cg16000360	0.00628539	464.288378	5.67497996	37984.9267
cg16079865	0.00628889	0.00298738	4.62E-05	0.19334186
cg02986643	0.00629002	13.5370944	2.08851283	87.7432599
cg26328687	0.00629355	31.0916787	2.64142671	365.973617

cg03747003	0.01134249	204.546072	3.32687207	12576.1059
cg08327378	0.01134725	1040.38474	4.80269865	225373.376
cg00204465	0.01134761	9.05616898	1.64489906	49.8597139
cg10439691	0.01135185	18282878.5	43.645832	7.6585E+12
cg02219147	0.01135968	175767713	72.6598086	4.2519E+14
cg17349406	0.0113598	35.7880347	2.24261368	571.111932
cg11669397	0.01136013	100436.778	13.4634187	749255934
cg15456476	0.01137121	157.763913	3.13300332	7944.27895
cg27105630	0.01137421	182150.592	15.3751819	2157947684
cg14323199	0.01138167	27.5566162	2.11267132	359.434566
cg02129266	0.0113841	1210904.58	23.5428822	6.2282E+10
cg24951781	0.01139376	38.2015056	2.27324274	641.970611
cg27028281	0.01140913	8.97319125	1.63939385	49.1145926
cg19592277	0.01141077	7.61186537	1.5796899	36.6783977
cg26298967	0.01142756	18.0099208	1.9170275	169.198015
cg17895870	0.01143224	10997.8345	8.12009818	14895431.2
cg09820519	0.01143485	24.4046735	2.05229156	290.206374
cg14077270	0.01144487	5750689.92	33.1574157	9.9738E+11
cg12224388	0.01145194	0.13155768	0.02731029	0.6337326
cg11875676	0.01145768	1.0007E+11	297.276523	3.3683E+19
cg08700546	0.01146864	311.26343	3.63300419	26667.991
cg20417500	0.01148402	23.4753794	2.03147659	271.277277
cg07142961	0.01148497	149120.464	14.5152746	1531966393
cg13338827	0.01149629	9.15174521	1.64370396	50.9546989
cg09368716	0.01149648	26.0261262	2.07829349	325.920882
cg12007596	0.01150116	8.57472729	1.61969163	45.3950287
cg02519208	0.01150534	14.4075033	1.81954967	114.081059
cg04923496	0.01151201	2.3922E+11	356.828919	1.60E+20
cg02595219	0.01151414	12.175862	1.75176074	84.630059
cg04529955	0.01151531	1339194025	111.43655	1.6094E+16

cg24520975	0.00629405	12.6899419	2.05042035	78.5373724
cg12033622	0.0062959	15.1934496	2.15730502	107.004299
cg00624212	0.00629942	11107078.5	97.8587565	1.2607E+12
cg11270393	0.00630173	5.45316908	1.6147405	18.4159951
cg23463742	0.00630483	3938361.28	72.9274054	2.1269E+11
cg12412075	0.00630511	429.065362	5.54068699	33226.4004
cg11188483	0.0063121	67.8908901	3.29021677	1400.87213
cg10718721	0.00631422	111655.589	26.6168527	468386350
cg15287024	0.00631445	3361.77149	9.90003294	1141562.62
cg05655806	0.00631675	9.16998709	1.86926136	44.9849684
cg11941520	0.00631991	173.050427	4.28275963	6992.32572
cg06389888	0.00632168	14.8162516	2.13999184	102.580443
cg10864791	0.00632417	3213178843	481.697185	2.1434E+16
cg07283778	0.00632968	5.3357E+11	2034.35614	1.40E+20
cg16640096	0.00633005	1089.61982	7.19197585	165082.779
cg03407228	0.0063327	79.8840073	3.44060913	1854.74559
cg09251959	0.0063339	21.7887314	2.3848389	199.069554
cg12582022	0.00633786	4438350.93	74.9104811	2.6297E+11
cg26455579	0.00634095	13298508.2	102.009192	1.7337E+12
cg20972917	0.00634346	27.8279223	2.5541037	303.195699
cg15366555	0.00634742	14.2683262	2.11537183	96.2408264
cg27347140	0.00635167	354.797662	5.2314621	24062.3708
cg27377289	0.00635443	9.74800306	1.89956442	50.02387
cg00842549	0.00635884	10.1357479	1.92029016	53.4988871
cg07138512	0.00635945	31.1298196	2.63415348	367.885043
cg17198397	0.00636833	0.06599523	0.00936345	0.46514606
cg14172427	0.00636897	0.07670235	0.0121236	0.48527246
cg10794285	0.00637129	4.79381312	1.5546904	14.7814923
cg08440349	0.00637156	446002.76	38.9524173	5106703923
cg02519806	0.0063741	9.75421766	1.89874429	50.1093079

cg06364315	0.01151764	490.202493	4.01195521	59895.6047
cg07869548	0.01151827	21.3418574	1.98651562	229.28331
cg24439070	0.01153423	200970.85	15.4341201	2616882737
cg13099839	0.01153666	54.5325567	2.4499735	1213.80894
cg18404628	0.01154906	14551431.1	40.2050554	5.2666E+12
cg18815943	0.01155308	6.37098162	1.51386936	26.8116972
cg08781140	0.01155729	3.74404719	1.34390175	10.4307397
cg10896318	0.01155951	3188.38666	6.08632149	1670271.53
cg05129081	0.01156879	11.4620631	1.72603079	76.1161913
cg12195820	0.01158198	97286.2868	13.0510007	725202750
cg25953133	0.01158527	2402.68467	5.70208404	1012418.2
cg15851312	0.01158616	4.4146E+11	401.864607	4.85E+20
cg25886284	0.0115891	7.23470187	1.55655234	33.6261813
cg14346035	0.01159024	116.608723	2.89795983	4692.12655
cg09323092	0.01159429	0.05194148	0.00522605	0.51624439
cg01046287	0.01160831	1165.64976	4.84312206	280550.305
cg09728506	0.01160989	121.820761	2.92385843	5075.58701
cg15837119	0.01161243	22700.4873	9.39827233	54830516.4
cg17211192	0.01161798	8545.25578	7.55201033	9669133.53
cg07381000	0.01161841	37.4958117	2.24650541	625.83241
cg02067983	0.01161922	16.5916916	1.8724972	147.014496
cg04186360	0.01162362	10.8864733	1.70416258	69.5445979
cg15718572	0.01162605	8.53947947	1.61415152	45.1771156
cg20091128	0.01162914	42746452.5	50.5123238	3.6175E+13
cg21634218	0.0116316	7.20430877	1.55387207	33.4017619
cg10497871	0.01163828	10.6328462	1.69467013	66.7135246
cg13686362	0.01165533	50430.484	11.1842472	227394269
cg12184886	0.01165842	6.4717875	1.51641804	27.6203741
cg00155423	0.01165897	10.2532273	1.6802254	62.5681941
cg23858195	0.01167775	51309.2906	11.2020826	235013738

cg25074426	0.00637535	6.91517537	1.72344656	27.7465235
cg12968732	0.00637855	0.16908293	0.04714652	0.60638706
cg19426955	0.00638048	22.312085	2.39612222	207.764502
cg02486590	0.00638561	2780400	65.0224454	1.1889E+11
cg10119082	0.00638631	5.62221222	1.62547701	19.4461503
cg07294541	0.00639034	14.3995288	2.11752816	97.9190899
cg13827970	0.00639683	26095.741	17.4556956	39012349.6
cg00321850	0.0063974	540.243019	5.86653389	49750.4191
cg01959640	0.00640176	70.1748385	3.30385959	1490.53185
cg25851425	0.00640334	6853.79244	11.9762985	3922286.25
cg20474425	0.00640709	7.06323576	1.73229146	28.7995989
cg06288821	0.00641182	581.436846	5.98154877	56518.6073
cg16618637	0.00641382	0.00314041	4.98E-05	0.19803028
cg16013543	0.00641527	14.072313	2.10199679	94.2104167
cg13996209	0.00641593	251.925068	4.72729939	13425.475
cg12434258	0.00641824	238389.305	32.3986052	1754071209
cg11060910	0.0064206	0.01450806	0.00069116	0.3045362
cg18044111	0.00643352	16.7444646	2.20567977	127.115957
cg24905333	0.00643501	6.44E-08	4.32E-13	0.00958394
cg20898273	0.00643636	10.7592704	1.94797476	59.4267966
cg07394955	0.00643721	54.043471	3.06405793	953.211989
cg19801120	0.00643928	55.3763093	3.08473362	994.100627
cg15718811	0.00644022	25.6862034	2.48643394	265.352332
cg17864516	0.00644127	209105766	216.10551	2.0233E+14
cg27211696	0.00644184	46.6924007	2.94016509	741.516281
cg06033194	0.00644336	413.651735	5.42181258	31559.1429
cg17861791	0.00644481	5069.01336	10.9499235	2346582.27
cg09820792	0.00644672	14.4089256	2.11359855	98.2292197
cg11159091	0.00644951	41.0546735	2.83481078	594.56745
cg10300057	0.00645146	9.2546E+11	2270.22988	3.77E+20

cg13709054	0.01167827	6398.6297	7.04482633	5811706.36
cg19389852	0.01168704	0.00211026	1.76E-05	0.25361107
cg22678932	0.01170366	83852.98	12.4634153	564156941
cg17493098	0.01170553	156.200079	3.07698078	7929.3523
cg04401436	0.01171716	2246036.78	25.8579674	1.9509E+11
cg21922731	0.01172481	136.24207	2.98217331	6224.28666
cg02289168	0.01173103	10914991	36.6771896	3.2483E+12
cg09078754	0.01173332	14.5191773	1.81237984	116.314751
cg14019186	0.01173627	5.33636786	1.45082571	19.6280102
cg14515252	0.01173649	53656.544	11.2473649	255973266
cg02683846	0.01174033	59.6566764	2.480485	1434.76741
cg09886849	0.01174422	0.05524933	0.00580836	0.52553397
cg13066963	0.0117497	23.3966214	2.01416802	271.775685
cg00679711	0.01175191	0.03909357	0.00313966	0.48677517
cg14112754	0.01175287	12.1386199	1.74087504	84.6390981
cg24137660	0.01175569	0.07913931	0.01100009	0.56936196
cg03893150	0.01179201	11.6151808	1.72238882	78.3286702
cg14523898	0.01179271	10.118104	1.67046768	61.285848
cg21082050	0.01179797	35.4435184	2.20530556	569.645774
cg20878190	0.01179878	1.7326E+11	309.795136	9.69E+19
cg18152871	0.01179998	8.99E-23	6.22E-40	1.30E-05
cg03460527	0.01180142	295.3898	3.52782225	24733.427
cg05226168	0.01180498	18.8677341	1.91730939	185.672376
cg02583309	0.01181589	6.68378801	1.52313128	29.3297253
cg04724058	0.01182478	8468780.39	34.1901394	2.0977E+12
cg16568036	0.01182537	103.816125	2.7952064	3855.81111
cg15773539	0.01182976	104.725218	2.80008051	3916.80568
cg19564884	0.0118335	88226.7447	12.4341681	626013611
cg09652501	0.01184191	16991.1548	8.62886893	33457379.3
cg26013028	0.01184619	379.462665	3.72005866	38706.8935

cg11910375	0.0064559	13.4297482	2.07161293	87.0616966
cg21396517	0.00645607	7.39669221	1.75256834	31.2176447
cg20583945	0.00645617	10.0176664	1.90813883	52.5924203
cg16354207	0.0064606	270.118908	4.80452329	15186.569
cg06301673	0.00646463	9.9431767	1.90364948	51.9353819
cg26159395	0.0064672	46.9009101	2.93996284	748.205161
cg04992638	0.00646789	3.63362746	1.43558159	9.19714259
cg07125725	0.0064701	18.189214	2.25428307	146.763958
cg17789479	0.00647979	9.1002E+17	107127.177	7.73E+30
cg27248189	0.00647995	23.1924199	2.412114	222.994577
cg08695855	0.00648192	9.86882661	1.89863498	51.2967156
cg20202112	0.00648698	9.95858124	1.9031563	52.1099294
cg24850474	0.00648799	70.6468597	3.29368624	1515.31701
cg26996201	0.00649142	6.24132056	1.66960313	23.3313424
cg18952098	0.00649282	2455.92813	8.89043853	678434.812
cg09634802	0.00649283	5001052841	518.526153	4.8234E+16
cg24991933	0.00649697	0.01431664	0.00067261	0.30473476
cg27140083	0.00649787	1109720.63	49.1639899	2.5048E+10
cg04380939	0.00650062	11.2106143	1.96644409	63.9112365
cg18018798	0.00650382	36913969.4	130.900013	1.041E+13
cg14103123	0.00650407	13.5726256	2.0742527	88.8108603
cg03912887	0.00650464	418718.963	37.383914	4689866612
cg20018469	0.00650543	37609.448	19.0480941	74257853.4
cg18478175	0.00651498	369.219303	5.22178329	26106.5781
cg14788660	0.00651509	155757.064	28.3017592	857199818
cg18353196	0.00651739	21.9417788	2.37126134	203.031883
cg13093774	0.00651955	5.94016892	1.6455402	21.4431751
cg04071446	0.00652302	6278.74214	11.5208365	3421852.48
cg17527393	0.00652553	17472.3045	15.3309864	19912706
cg10024406	0.00653389	7.78564872	1.77411564	34.167066

cg24421668	0.01184621	21.9041169	1.9794458	242.386194
cg13811713	0.01184865	107.785468	2.81569693	4126.05029
cg16676472	0.01185132	8.20E-09	4.13E-15	0.01627755
cg02891726	0.01185952	0.06643883	0.00803891	0.54909434
cg10246448	0.011872	41.6208694	2.27945368	759.961381
cg11779239	0.01188147	12.5156026	1.74749662	89.6369731
cg11277662	0.01188399	0.01641214	0.00066764	0.40344916
cg15021670	0.01189165	20.3517389	1.94506795	212.945402
cg05523897	0.01189172	18.0316049	1.89377044	171.688589
cg23719130	0.01189567	16645.9007	8.54859571	32413044.2
cg25851425	0.01190654	44410.8716	10.6050973	185979012
cg27377289	0.01191753	12.6876335	1.75130061	91.9179967
cg06731484	0.01192063	8.46650786	1.60172274	44.7529111
cg09601584	0.01192066	18.1986905	1.89616995	174.663847
cg14849065	0.01192767	30.9839221	2.13179324	450.326708
cg22923268	0.0119303	8447.91893	7.33872598	9724758.01
cg16717690	0.01193214	44133.0067	10.5638638	184375936
cg02853019	0.01193529	6029.12832	6.81011398	5337706.3
cg01353524	0.01193953	221633.705	15.0643677	3260774043
cg15457058	0.01194009	5.41662644	1.4510384	20.2198935
cg06200512	0.01194311	1140396305	98.9676808	1.3141E+16
cg19458266	0.01194743	656.491643	4.17439791	103243.937
cg10523140	0.01194765	11.5186782	1.713204	77.4455041
cg23115158	0.01194941	4005786.58	28.4678349	5.6367E+11
cg05136476	0.01195765	143.540587	2.98502313	6902.42563
cg06240896	0.01197276	20061.8253	8.8460807	45497757.3
cg11690979	0.01197665	8.26902914	1.59168184	42.9588635
cg17147211	0.01198928	12.5912763	1.74545198	90.8304785
cg23273897	0.01199078	10.3730129	1.67257613	64.3315387
cg05460965	0.01199148	33.6613932	2.16665199	522.967878

cg03834567	0.00653467	55.7909979	3.07526019	1012.15352
cg16209444	0.00653598	15.0623393	2.13309194	106.359253
cg11408656	0.00653696	12333.7153	13.8890512	10952550.4
cg12848614	0.00653893	37.6243454	2.75421874	513.97202
cg18352648	0.00654	0.10846178	0.02187603	0.53775566
cg25510614	0.00654488	14.5279339	2.11100294	99.9813213
cg19127747	0.0065483	39.0495828	2.78168791	548.181523
cg00468144	0.00654925	3.3348E+13	5954.77485	1.87E+23
cg00579105	0.00655909	1100.40216	7.05710129	171583.893
cg05887405	0.00656046	7.1689265	1.73246875	29.6648972
cg05357695	0.00656464	14.7245176	2.11743308	102.393517
cg13265524	0.00656805	13.7273109	2.07616869	90.7628873
cg07774177	0.00656861	211.349168	4.45045994	10036.8213
cg14512004	0.0065716	1.0333E+10	620.025127	1.7222E+17
cg11566061	0.00658328	10.0254247	1.90105686	52.8701385
cg08247376	0.00658376	4.53427584	1.52390022	13.4914721
cg24272980	0.00658723	0.00536506	0.00012353	0.23301277
cg06822966	0.00658791	273216.123	32.7153943	2281710233
cg14777244	0.00659419	116.239878	3.76087445	3592.70415
cg00123512	0.00659682	1760110096	375.794447	8.2438E+15
cg26057784	0.00659711	16.4941806	2.18288478	124.632319
cg17246382	0.00660282	9.37543556	1.86478596	47.1361292
cg11805463	0.00660605	0.05726534	0.00727055	0.4510416
cg05807291	0.00660744	13.8993096	2.08051176	92.857349
cg07331806	0.00661178	54.2314965	3.03847811	967.936943
cg21520826	0.00661251	51757147.1	140.211999	1.9105E+13
cg14045725	0.00661922	33.9160382	2.66546217	431.556546
cg14075221	0.00662161	22.2451095	2.3701182	208.784901
cg09168604	0.00662942	5.79170707	1.6297324	20.5824408
cg00320059	0.00663143	182339.632	29.0249438	1145488575

cg03948207	0.01200706	339032.666	16.4140074	7002747436
cg25509428	0.01200864	125814.283	13.1989968	1199275528
cg23927983	0.01201262	91496419.6	56.1091879	1.492E+14
cg14657834	0.01202034	8.45744716	1.59822933	44.7547865
cg02165978	0.01202079	433849.732	17.2998443	1.088E+10
cg06150468	0.01204037	23.6635976	2.00227083	279.665389
cg25634545	0.01204814	21831.9148	8.95047232	53252217.9
cg25644556	0.01204858	6.29954922	1.49739885	26.5021709
cg14562523	0.01205233	27.6453573	2.07103187	369.026567
cg27531553	0.0120587	17.383614	1.87035618	161.568175
cg22345522	0.0120713	198173.841	14.4834195	2711574518
cg27637948	0.01207703	147.302788	2.98579474	7267.11423
cg17603132	0.01207752	10.3806096	1.66975274	64.5347377
cg15945129	0.01207846	6.71777108	1.51787544	29.7313252
cg01440333	0.01207919	21.5771028	1.9599937	237.537175
cg09954820	0.01208622	12.0860933	1.7260001	84.6313105
cg02144933	0.0120877	11.6733696	1.71285851	79.555641
cg00846502	0.01209512	0.06426221	0.00753173	0.54829777
cg07312366	0.01209575	2.6305E+10	191.10822	3.6207E+18
cg24959938	0.01209659	0.07912336	0.01090931	0.57386801
cg20930060	0.01210063	1.4474E+13	759.78997	2.76E+23
cg02927327	0.01210856	13.2111711	1.75905532	99.2208944
cg05341353	0.01211562	4510.04917	6.30024933	3228529.93
cg17334359	0.01212607	40.5359516	2.24680279	731.334044
cg19632236	0.01212771	9.32387284	1.62927457	53.3578603
cg01957900	0.01212801	0.03660878	0.00276202	0.48522555
cg14667731	0.01213067	5138230.86	29.3146049	9.0062E+11
cg04757806	0.01213378	0.0615647	0.00697107	0.54370591
cg17759086	0.01214042	86.3604267	2.6493296	2815.09832
cg07971863	0.01218376	45.9032486	2.30411611	914.497417

cg00661320	0.00663202	17.5269798	2.21716995	138.552762
cg18599117	0.00663775	342450982	235.668724	4.9762E+14
cg04712436	0.00664399	15305.1325	14.5506669	16098717.8
cg21916655	0.00664777	4901.90017	10.5997101	2266913.43
cg23273897	0.00665054	9.79375866	1.88485987	50.8885093
cg18605031	0.00665167	8.12501857	1.78949058	36.8909048
cg09239591	0.00665753	22.1974877	2.36520868	208.323461
cg22376366	0.00666366	1.95E-05	7.69E-09	0.04922469
cg02926965	0.00666538	582.358548	5.85608858	57912.6279
cg06727065	0.00667503	45.341689	2.88155409	713.458329
cg24326232	0.00667581	14.1972782	2.08780193	96.5430228
cg16773799	0.00667597	1460.63053	7.55131335	282525.893
cg01052428	0.00667898	31843.2018	17.7505603	57124366
cg27598407	0.00668007	10.4334038	1.91652167	56.7986868
cg01440333	0.00668142	19.6908437	2.28564499	169.636723
cg27037013	0.00668219	29.8874454	2.56603521	348.108783
cg22697962	0.00668611	6.8829941	1.70739715	27.7472689
cg06101723	0.0066903	233837.746	30.8051858	1775028778
cg03693099	0.00669064	486.978323	5.56091511	42645.4787
cg17129986	0.00669065	8.61700589	1.81693472	40.8670656
cg02916459	0.00669274	388.057471	5.22074421	28844.2786
cg21576698	0.00669418	2861.65252	9.0829974	901580.7
cg22760295	0.00669444	661327.811	41.0669482	1.065E+10
cg00582337	0.00670097	21.9337115	2.35319267	204.440421
cg01081091	0.00670181	3704.05422	9.74843756	1407406.84
cg09598437	0.00670329	14.9588762	2.11621343	105.739796
cg23456144	0.00670571	103.977745	3.62094028	2985.79117
cg22583148	0.00670808	7.70322845	1.76050522	33.7060793
cg06926254	0.00671509	36284.1603	18.3133928	71889480.2
cg14400886	0.00671878	7.45913425	1.74437626	31.8960336

cg04822518	0.01218515	16.7743367	1.84978459	152.114129
cg10971510	0.01218895	10.6131683	1.67387639	67.2925083
cg19641839	0.01219508	0.04139206	0.00343076	0.49939522
cg24421410	0.01219942	8.70977721	1.60292866	47.3260108
cg27455796	0.01220914	3371039.5	26.4501345	4.2964E+11
cg05096161	0.01221137	11.9448291	1.71672393	83.1111742
cg21384402	0.01221787	6.06866207	1.48107662	24.8661404
cg19712821	0.0122287	18.9782768	1.89806804	189.758736
cg10314411	0.01223919	0.02426977	0.0013231	0.44518155
cg01593886	0.01224244	35.9700016	2.18061093	593.338775
cg22968727	0.01225205	8811.38699	7.21296239	10764029.6
cg27533013	0.01225273	3876994785	121.787047	1.2342E+17
cg05155520	0.01225853	0.15059793	0.03423168	0.66253649
cg09807229	0.01226216	21983.3815	8.79191051	54967468.5
cg18617679	0.01226854	248.353626	3.31598226	18600.6796
cg15909016	0.01227314	33266421	43.1254374	2.5661E+13
cg02204038	0.01227932	475099547	76.7740499	2.9401E+15
cg27629124	0.01227991	0.09605358	0.01534943	0.60108378
cg01557883	0.01228093	4009834.06	27.2002548	5.9113E+11
cg10321263	0.01228477	0.0370675	0.00281082	0.48882538
cg12680131	0.01229144	0.28351412	0.10568889	0.76053643
cg07113947	0.01229301	6611193205	135.660373	3.2219E+17
cg21774561	0.01229407	4770.62542	6.29158085	3617352.68
cg20583141	0.01229849	19.2968691	1.90141677	195.837736
cg07218275	0.01230539	286.378056	3.41385804	24023.3747
cg02873371	0.01230862	1554.67362	4.92730435	490533.947
cg25864727	0.01231626	0.13497376	0.02813075	0.64761572
cg11770080	0.01232084	7.52595479	1.54925924	36.5594046
cg19240052	0.01233339	552.479489	3.93090424	77649.7128
cg03709297	0.01234299	0.08847504	0.01323927	0.59125879

cg24283914	0.00672192	67779184.6	147.245203	3.12E+13
cg03753597	0.0067229	481022.122	37.4141255	6184356272
cg27531336	0.00672297	12.5581182	2.01475544	78.2756701
cg03081691	0.00672299	6.15366741	1.65371589	22.8985057
cg05851813	0.00672368	45918684.5	132.143851	1.5956E+13
cg26541003	0.00672538	16461.3722	14.6939198	18441421.9
cg22957613	0.00672675	0.06324157	0.0085876	0.46572918
cg14892066	0.00672753	28041.5499	17.0234177	46190990.1
cg05335944	0.00672926	9.05028227	1.83975396	44.5209583
cg10883621	0.00673479	10.5121529	1.91726697	57.6369174
cg10616515	0.00673809	298676.09	32.7074333	2727435253
cg13354228	0.00674105	8.73E-15	5.90E-25	0.0001292
cg10454937	0.00674108	9.78993403	1.87952463	50.993111
cg27367469	0.00675121	18.4366758	2.23829457	151.861609
cg14579819	0.0067539	14.1126779	2.07867434	95.8147567
cg24805759	0.00675744	18.7355423	2.24772887	156.166765
cg01147995	0.00675915	3600504.7	64.8519965	1.999E+11
cg12798700	0.00677489	17107.147	14.7574789	19830926.4
cg17369196	0.0067874	32.6788786	2.61772559	407.953037
cg05816157	0.00679656	20.1883855	2.29107921	177.894726
cg10150530	0.00679736	4.79079965	1.54062858	14.8976603
cg00402172	0.00679857	8.3196122	1.79393139	38.5833861
cg09954813	0.0067988	9.38632837	1.8546208	47.504676
cg02956255	0.00679948	34.6301896	2.6584751	451.104484
cg04482943	0.00680446	2706818.88	59.4109439	1.2333E+11
cg11612345	0.00680498	9.07752046	1.83725866	44.8501779
cg23967249	0.0068071	1.0098E+17	48825.2129	2.09E+29
cg14849526	0.0068083	816405.28	42.6597277	1.5624E+10
cg11538128	0.00681271	8.69934259	1.81542842	41.6863372
cg07383443	0.00682013	2773.62465	8.88747677	865599.305
cg15253243	0.01234948	115504.795	12.4957814	1067668938
cg12216208	0.01235684	6.06172706	1.47737324	24.8715314
cg01966160	0.01236198	14.5175005	1.78477024	118.086807
cg20949959	0.01236579	122300695	56.348187	2.6545E+14
cg19118262	0.01236905	14457.4826	7.95267976	26282814.2
cg26985711	0.01237931	20.0969821	1.91408871	211.008345
cg25096582	0.01238414	119405.879	12.5403616	1136949980
cg06762403	0.01238783	284.58028	3.39496871	23854.6929
cg23244913	0.01239599	6.61961363	1.50481515	29.1193802
cg18672998	0.01239753	0.01123799	0.0003333	0.37891372
cg02988775	0.01240097	16.3217912	1.82883511	145.666969
cg14935646	0.01241033	17.0496238	1.84572625	157.493383
cg21956537	0.01243128	593475.282	17.6409172	1.9966E+10
cg16385335	0.01243234	13.0017976	1.73984767	97.1618052
cg15402162	0.01243444	25.6816896	2.01515533	327.294463
cg03056526	0.01243856	29.8723527	2.08173649	428.66014
cg23401796	0.01244004	0.26589525	0.09409909	0.75133865
cg15846316	0.01244326	7.3204708	1.53662521	34.8746671
cg07375912	0.01244641	1119155.89	20.1921927	6.2029E+10
cg25114752	0.01244711	0.10920924	0.01923223	0.62013898
cg07461318	0.01245949	901.310202	4.33745572	187289.538
cg07870237	0.01245992	14.9271406	1.79130703	124.389356
cg24991933	0.01246352	0.0120159	0.00037459	0.385441
cg20371266	0.01246776	10.1705818	1.64878988	62.7373662
cg09244244	0.0124807	20.032518	1.90756803	210.373507
cg00260114	0.01248463	8163.82079	6.96215688	9572891.14
cg05691004	0.01248795	0.08857584	0.01322454	0.59326683
cg11357542	0.01249547	66.771932	2.47114421	1804.22125
cg19886978	0.01249853	2784.48394	5.51669216	1405434.74
cg18542853	0.01250683	0.08068213	0.01119058	0.58170394

cg26293590	0.00682496	67.8538666	3.19595017	1440.61921
cg10510478	0.00682749	6.04884014	1.64181927	22.2853195
cg19040032	0.00682924	20973.8471	15.5018609	28377384.4
cg00826842	0.00683107	492118.615	36.9585883	6552759250
cg00531137	0.00683199	6.51085938	1.67526408	25.3042434
cg08267698	0.00683736	1315652913	324.232519	5.3386E+15
cg12894325	0.00683751	86.8836862	3.41870586	2208.07968
cg02466815	0.00684096	4.55200732	1.51774257	13.6523618
cg25789201	0.00685063	62219.5176	20.8510324	185663150
cg18307767	0.006851	642616455	265.222275	1.557E+15
cg11097541	0.00685141	16.4864391	2.16222084	125.705326
cg17727579	0.00685812	0.06299153	0.00848877	0.46743328
cg27626899	0.00685837	438893.582	35.6467539	5403790107
cg11341981	0.00686056	9.17684084	1.83982561	45.7730381
cg04368796	0.0068619	706.76806	6.07586338	82214.0095
cg27629124	0.0068644	0.10911149	0.02189347	0.54378402
cg11171429	0.00686762	11322.7273	13.020168	9846582.19
cg26766373	0.00686815	11.1819194	1.94212788	64.380581
cg24700222	0.00687103	10.6855632	1.91786424	59.5356328
cg09481857	0.00687293	23.3272261	2.37680633	228.945654
cg05909880	0.00687507	2.5671E+11	1367.32975	4.8195E+19
cg09122913	0.00687562	808.338417	6.29692455	103766.686
cg04975205	0.00687596	486951.805	36.5708102	6483915960
cg16478719	0.00687919	46.7106306	2.87573045	758.723059
cg07235053	0.0068814	0.10874156	0.02175494	0.54354223
cg01434197	0.00688673	1591.17179	7.57713372	334140.557
cg07000334	0.00688796	32.5914296	2.60388687	407.929122
cg24589113	0.00689006	449238.167	35.6845072	5655533626
cg03760060	0.00689616	0.01380538	0.00061772	0.30853362
cg07242710	0.00689663	4.38E-06	5.68E-10	0.03378958

cg18940113	0.01251176	82.8444123	2.58696183	2652.99494
cg08389277	0.01251233	21.4136196	1.93349804	237.157264
cg22864672	0.01252565	1087.93827	4.49852049	263110.876
cg26156179	0.0125319	3193262.05	25.0309012	4.0737E+11
cg09571713	0.0125408	10.5725007	1.66007425	67.332995
cg11933779	0.0125448	9.05439617	1.60555332	51.0615804
cg01501819	0.01254619	5.64281556	1.4503865	21.9537119
cg05397514	0.01255502	6236987458	127.058958	3.0616E+17
cg06499652	0.01256249	34961.0389	9.45542316	129267006
cg15813594	0.01256853	18.9728402	1.88102811	191.368042
cg10883621	0.01256903	11.8475695	1.70014864	82.5603715
cg19197795	0.01257255	104.849343	2.71463938	4049.66673
cg16529993	0.01257292	212519.239	13.9156169	3245592862
cg02019072	0.01258097	295.955255	3.39047953	25833.96
cg06887580	0.01259139	36.9095697	2.16831549	628.283266
cg05704893	0.01260689	2978.52437	5.55433373	1597240.62
cg24716879	0.01260992	4.29245946	1.36646938	13.4838061
cg05748497	0.01261233	1360.42205	4.69389762	394288.137
cg06892005	0.01261557	1.4106E+12	401.067735	4.96E+21
cg16819803	0.01262046	21.0784081	1.92131131	231.247944
cg13612524	0.01263326	3244.12152	5.64628624	1863937.46
cg05585263	0.01264821	0.08443806	0.01209968	0.58925401
cg04505439	0.01265973	165253.06	13.0628671	2090549760
cg04085707	0.01266349	2102448.17	22.4942271	1.9651E+11
cg21692194	0.01266415	13.658261	1.74900028	106.659842
cg10985987	0.01266572	3629.84459	5.77015308	2283435.39
cg02275713	0.0126674	16.021049	1.80956101	141.843248
cg17735372	0.01267706	18.0885151	1.85667586	176.225901
cg21339084	0.01269174	32.9980281	2.11027525	515.984756
cg02083559	0.01269196	5.06918749	1.41438895	18.1680306

cg07218357	0.00689667	11.8756634	1.97273098	71.490428
cg05556202	0.00690384	0.07624611	0.01178258	0.49339546
cg06182274	0.0069043	23.2801124	2.37244935	228.44055
cg11594683	0.00690499	7.12E-06	1.31E-09	0.03864907
cg00557236	0.00690765	0.01260459	0.00052762	0.30111594
cg08307816	0.00690951	7.54377304	1.74105708	32.6861837
cg14262490	0.00691036	7.09E-12	5.76E-20	0.00087232
cg26792589	0.00691129	403.258243	5.18704494	31350.6462
cg14851471	0.00691911	65.7136495	3.15169827	1370.14503
cg03265267	0.00692268	527.202034	5.57770629	49830.875
cg19066060	0.00692562	46637.0145	19.0608439	114108858
cg05554353	0.00692739	8.4254E+13	6576.67144	1.08E+24
cg20473595	0.00692871	11.6676612	1.96118686	69.4142515
cg01894110	0.00693077	9027.09636	12.1430527	6710707.01
cg17211192	0.00693337	7005.77316	11.3245225	4334033.31
cg06846310	0.00693795	13.6209201	2.04556422	90.6984302
cg03839625	0.00693925	3582.33544	9.4169222	1362772.99
cg19592277	0.0069551	6.96064595	1.70110048	28.4819107
cg21295088	0.00695522	13.2106887	2.02732888	86.0848464
cg06618097	0.00695606	24.3533584	2.39686333	247.442588
cg14944944	0.00697043	44.6744727	2.82803906	705.721691
cg00735304	0.00698753	1061297948	293.524891	3.8373E+15
cg08327378	0.00698801	584.985375	5.70840375	59948.0878
cg27573806	0.00699007	172849.553	27.0269296	1105451803
cg03473294	0.00699155	6.2776E+10	894.411596	4.406E+18
cg20261082	0.00699165	18324.7259	14.6312032	22950647
cg24521811	0.00699819	32.3923443	2.58669535	405.638788
cg16608407	0.00699909	11.7570215	1.96091311	70.4914228
cg10336707	0.00701066	9.98341998	1.87458274	53.1684585
cg19878838	0.00701191	2219.99627	8.20048967	600986.482

cg14226064	0.01269801	10.6585608	1.65749749	68.540024
cg00263248	0.01269807	23.8756358	1.96899774	289.510735
cg10250177	0.01270657	23.2987472	1.95827832	277.198402
cg13075322	0.01270742	0.02437535	0.00131285	0.45257064
cg12058507	0.01271608	0.0008264	3.11E-06	0.2198839
cg06840042	0.01271894	4.21E-07	4.06E-12	0.04361402
cg01243246	0.01272408	160.200262	2.95306279	8690.68003
cg11844827	0.01272735	1.3069E+15	1674.86943	1.02E+27
cg04569641	0.0127371	0.14193046	0.0305438	0.6595202
cg04327181	0.01274573	66.0770848	2.44278049	1787.3817
cg12018140	0.01274754	25.9361762	2.00126863	336.129405
cg04364728	0.01274764	20.3461253	1.90037044	217.833748
cg02478603	0.01275887	0.13800202	0.02903867	0.65583433
cg14041079	0.01276201	1.1234E+15	1604.62947	7.87E+26
cg18592307	0.01277527	310.739844	3.39254242	28462.2087
cg13676119	0.01277631	11640.1837	7.33554879	18470857.4
cg05184938	0.01279072	9.53802728	1.61566735	56.3073608
cg23681866	0.01279821	5.2728057	1.42411916	19.5225798
cg02643054	0.01280364	9.87734655	1.62730341	59.9531557
cg02287056	0.01280841	8310541541	128.403707	5.3787E+17
cg26483081	0.0128111	2031817927	95.1308639	4.3396E+16
cg22995904	0.01281197	0.10182613	0.01684935	0.61536876
cg22027897	0.01281369	12.7345597	1.7172401	94.4358393
cg19519964	0.01281419	2597.35277	5.31668113	1268882.08
cg00599393	0.01281535	744298457	76.7879414	7.2144E+15
cg13623495	0.01281924	13.6221971	1.74178599	106.536771
cg10248985	0.0128206	45.1056359	2.24624166	905.743326
cg24363820	0.01282428	12.1510546	1.69981298	86.8613959
cg22160883	0.01283508	122.075688	2.77369337	5372.79058
cg08288330	0.0128375	1319313.23	19.9258313	8.7353E+10

cg03434509	0.00701985	7.90046658	1.75810391	35.5026638
cg10574494	0.00702248	14.3041719	2.06720551	98.9787092
cg09676684	0.0070232	6.38741186	1.6588459	24.5948284
cg15692032	0.00702357	94316.3173	22.7900124	390327462
cg00255368	0.00703378	14.3750697	2.06920009	99.8659475
cg19255465	0.00703561	30.32172	2.53627391	362.502923
cg08085208	0.00703777	220859.678	28.6835033	1700594134
cg03744763	0.00703807	11.5673032	1.94983268	68.622557
cg03744440	0.00705353	5.71234874	1.60794943	20.2935039
cg23098599	0.00705367	5134508.52	67.4461315	3.9088E+11
cg18233810	0.00705476	204728.175	28.0218116	1495750031
cg01655607	0.00705522	771356354	264.277275	2.2514E+15
cg17560720	0.00706135	3604997.62	61.1579609	2.125E+11
cg27535305	0.00706236	211.67492	4.3013733	10416.7364
cg21746532	0.00706277	0.12032603	0.02577875	0.56163903
cg08579540	0.00707919	9.18790814	1.82899717	46.1551595
cg00415978	0.00708229	10.2212836	1.88267041	55.4927921
cg21236153	0.00708423	13.5875957	2.03422386	90.7583288
cg22334684	0.00708726	0.00013777	2.13E-07	0.08899603
cg08464498	0.00708878	322.744887	4.8158373	21629.5228
cg12705353	0.00709009	12.9701937	2.00825657	83.7671472
cg03119288	0.00709158	7729.21781	11.4245906	5229142.11
cg05012767	0.00709298	4.8054E+12	2818.89168	8.19E+21
cg05940703	0.00709643	11.8757599	1.96026899	71.9460815
cg00263592	0.00709775	12.9580605	2.00724415	83.6526702
cg12959887	0.00709925	702861501	254.738748	1.9393E+15
cg23713813	0.00710026	5074552.81	66.6178291	3.8655E+11
cg21329975	0.00710037	17.711084	2.18508904	143.555933
cg20930060	0.00710418	9.2314E+11	1792.50899	4.75E+20
cg18130930	0.00710445	0.16393548	0.04394206	0.61159711

cg18229767	0.01284288	13.7332181	1.7438447	108.152566
cg02705837	0.01285531	7.39865974	1.52895605	35.8023148
cg22806837	0.01285579	7.71306838	1.54250229	38.5681268
cg04380635	0.01286438	58609976.4	44.4012312	7.7366E+13
cg22376864	0.01287365	17.3161637	1.83039392	163.816937
cg11110213	0.01287579	4193.42495	5.85997323	3000835
cg18935660	0.01288615	8.61354999	1.57815947	47.0125137
cg22416703	0.01289454	2.4383E+10	158.526922	3.7503E+18
cg14870223	0.01290646	17.0956712	1.82394115	160.236515
cg01370129	0.01290683	2609616.12	22.8252064	2.9836E+11
cg10482632	0.01290724	37.2423226	2.15074534	644.888339
cg10494773	0.01291045	4.81E-06	3.09E-10	0.07488264
cg19476445	0.01291449	149.893313	2.88719441	7781.95092
cg27598407	0.01293496	10.8649567	1.6560622	71.2819147
cg25420952	0.01293646	16.9511519	1.81931613	157.939319
cg09550809	0.01294241	23.6790776	1.95223182	287.209087
cg18267049	0.01295806	10.2198053	1.63399793	63.9195552
cg03065614	0.01295861	17.0048284	1.81953594	158.921945
cg05855588	0.01296233	6.87343783	1.50254484	31.4427538
cg06241689	0.01296331	36.9984712	2.14396416	638.484027
cg02602480	0.01297565	7925185.54	28.6024802	2.1959E+12
cg22725685	0.01298202	5.91935136	1.4554149	24.0747298
cg21616552	0.01298896	329.507698	3.39811437	31951.6389
cg17512993	0.01299502	570079.73	16.372919	1.9849E+10
cg08381596	0.01299787	269508.685	13.975304	5197377547
cg17838626	0.01300178	97.9562146	2.62942342	3649.24869
cg25755428	0.01300265	8.12709555	1.55552298	42.461399
cg05395366	0.01300565	38142.0213	9.24597474	157345637
cg04655372	0.01300581	1.0909E+12	345.18995	3.45E+21
cg05240381	0.01302789	17343061.3	33.4897771	8.9813E+12

cg03429645	0.00710455	11.734613	1.95340766	70.4927836
cg13208438	0.00710511	6.51664509	1.66467976	25.5104099
cg16735821	0.00710567	1327696076	302.328072	5.8307E+15
cg07709606	0.00710867	9.95356598	1.86767915	53.0463038
cg05580277	0.00710984	0.06699945	0.00935965	0.47960403
cg01963633	0.00711593	0.13826421	0.03272955	0.58408974
cg23473726	0.00711716	245754.472	29.1633075	2070933158
cg13694927	0.00712188	8.15598698	1.76861701	37.6113783
cg06083200	0.00712613	20.7363247	2.27857997	188.711903
cg25190465	0.00712976	1497107.18	47.544706	4.7142E+10
cg20180059	0.00713262	4477272801	417.555461	4.8008E+16
cg01041284	0.00713812	125.360721	3.71198023	4233.673
cg00054301	0.007141	1135.80003	6.75051993	191102.57
cg23470128	0.00714123	11.4571711	1.93854092	67.7142118
cg00092273	0.0071436	269758544	194.197543	3.7472E+14
cg05226168	0.00714623	13.9141814	2.04318082	94.7563932
cg05208572	0.00714666	26.6392171	2.4369374	291.20481
cg09937190	0.00715746	7.31907862	1.71581268	31.2207227
cg22027897	0.00715963	11.9781156	1.96094713	73.166304
cg10392164	0.00716439	3202.8568	8.92305768	1149638.62
cg25239996	0.00716856	7.08595037	1.70034695	29.5296749
cg21126828	0.00716976	2393.09343	8.24068609	694953.807
cg21903286	0.00717533	8.04169763	1.7593682	36.7568885
cg23907982	0.00717697	9.01592843	1.81466268	44.794532
cg07042077	0.00718454	0.0141471	0.00063431	0.31552249
cg01883662	0.00719068	0.14681385	0.0362398	0.59476894
cg22301984	0.00719279	2009.37404	7.84205127	514863.252
cg18153578	0.0071957	3793204.27	60.4332426	2.3809E+11
cg08352268	0.00720023	9.85617629	1.85776731	52.290839
cg01294023	0.00720376	221830995	181.489524	2.7114E+14

cg05027458	0.01303019	7.91319901	1.546033	40.5028345
cg08970083	0.01303523	40.9753212	2.18561107	768.195664
cg15239888	0.01303954	2659.53272	5.26119025	1344394.32
cg03595018	0.0130407	0.10933092	0.01904875	0.62750824
cg11356794	0.01305057	419.516162	3.56419894	49378.2229
cg11275620	0.01306186	114728.033	11.5963203	1135060197
cg18719814	0.01307153	10634.2049	7.02577304	16095924.6
cg15193461	0.01308251	72.5018302	2.46035565	2136.48599
cg26477856	0.013085	13.9710218	1.74049616	112.145868
cg20893919	0.01308903	95.2877835	2.60514156	3485.32373
cg24311704	0.01309219	23.5576892	1.9421028	285.754554
cg12768250	0.01310176	3629167487	101.773024	1.2941E+17
cg18372896	0.01310648	9.86735683	1.61714583	60.2077619
cg25306939	0.01310861	23.3759373	1.93807626	281.946824
cg03721195	0.01311082	14.4823834	1.7526461	119.670154
cg13985198	0.01311912	38.3712002	2.14988462	684.850242
cg16315376	0.01311935	8.92649196	1.58308958	50.3333858
cg23917399	0.01312522	9.17752513	1.59215091	52.9013719
cg01058070	0.01312659	13.5948971	1.72891637	106.900039
cg00225623	0.01313109	3.99868097	1.33737839	11.9558156
cg00506935	0.01313185	116.428588	2.71237775	4997.68745
cg16598405	0.01313424	983.695188	4.24308958	228054.63
cg03317402	0.01313459	44999.7863	9.45992014	214058971
cg27539986	0.01314272	7.14387013	1.51018404	33.7938152
cg09667013	0.01314864	15.8421371	1.78436366	140.651435
cg24496666	0.01314957	11.2161289	1.65974275	75.795811
cg21704448	0.01315565	17.0129999	1.81092132	159.831443
cg24668061	0.01316309	13.7953615	1.73279824	109.829288
cg01211283	0.01317539	8.71652666	1.57355012	48.2843449
cg01313358	0.01318565	47.9947382	2.24824739	1024.57359

cg16792202	0.0072079	7835.47548	11.3168684	5425058.77
cg21693907	0.00720843	19.7512024	2.24169383	174.024654
cg17519645	0.00721522	8.77146542	1.79931239	42.7600043
cg27645955	0.00721542	8.17627137	1.76542509	37.8670349
cg26960176	0.00721649	1055817442	275.923996	4.0401E+15
cg10508349	0.00721714	7.68354061	1.7359142	34.0090519
cg21290280	0.00722836	44580853	116.916837	1.6999E+13
cg13686012	0.00722937	41.113796	2.73090319	618.968927
cg23423766	0.00722947	7488.44979	11.1519943	5028417.22
cg13316191	0.00723214	31.7451664	2.54620586	395.787163
cg02811627	0.00723417	110.473972	3.56637872	3422.09825
cg06798642	0.00723807	1007.32368	6.47907851	156611.931
cg07583667	0.00724073	5.8205E+10	810.096026	4.182E+18
cg11153969	0.00724344	26.2537183	2.41766837	285.091923
cg15379858	0.00724655	12.5120393	1.97880147	79.1141147
cg04748051	0.00724715	1300507252	289.579784	5.8406E+15
cg23032421	0.00725584	11.3496545	1.92682083	66.8534691
cg02283931	0.00725867	15.4277428	2.09312462	113.71289
cg10440696	0.00726223	0.13916317	0.03297808	0.58725037
cg13277380	0.00726932	10.1354305	1.86811604	54.9895981
cg08315174	0.00726984	51.6559037	2.89891598	920.458686
cg02727530	0.00727188	14.0185801	2.03880447	96.3901099
cg25464921	0.00727224	3.96289433	1.4499011	10.8314501
cg19220754	0.00727693	16801.0864	13.7946348	20462774.8
cg21762534	0.00728068	1064.14158	6.55145574	172846.669
cg00393417	0.00728206	28444.28	15.8891643	50920051.7
cg15994744	0.00728896	21.3770959	2.28312756	200.155365
cg12688670	0.00728979	3544.96498	9.05471689	1387870.75
cg24727216	0.00729316	14.8558993	2.06948059	106.644027
cg27351691	0.00729617	10435569.7	77.8814111	1.3983E+12

cg15306012	0.01319006	22.4618562	1.91770684	263.092863
cg02221303	0.01319085	15171.5053	7.49597149	30706436.5
cg22076118	0.01319515	5050.13863	5.95295143	4284244.64
cg21128569	0.01319789	10.3385585	1.63005202	65.572013
cg08516222	0.01320867	32.3254865	2.06833712	505.206365
cg19355621	0.01321014	4960.29837	5.92412046	4153284.88
cg05421149	0.01321793	0.09085243	0.01362652	0.60574269
cg06305609	0.01322039	14.211815	1.74134107	115.988585
cg20750843	0.01323342	3150411.52	22.767599	4.3593E+11
cg16472904	0.01323545	31.4285859	2.05456976	480.760513
cg13430464	0.01324032	18.5873103	1.84088459	187.675049
cg12019109	0.01324347	10.2946022	1.62710907	65.1332089
cg15331996	0.01324838	32.3006778	2.06554871	505.112166
cg17518962	0.01325068	19.4435425	1.8577861	203.495625
cg05323898	0.01325076	2639562.62	21.8932551	3.1824E+11
cg19761115	0.01325251	8.29389064	1.5550721	44.235005
cg21779231	0.01325964	672.161705	3.88989606	116147.411
cg24034966	0.01326262	15389.1602	7.47366236	31688112.1
cg17399362	0.01327275	7.27899542	1.51275854	35.0246077
cg00528640	0.01328639	339102.149	14.2102997	8092036715
cg23359394	0.01329009	13.0868941	1.70895208	100.217437
cg14182820	0.01331657	0.06958411	0.00843254	0.57419811
cg17188046	0.01332183	8.38789272	1.55676132	45.1943041
cg01994599	0.01332349	74565588.7	43.4757535	1.2789E+14
cg11983038	0.0133339	10.4644731	1.62970343	67.1933281
cg18507365	0.01333617	141.374953	2.80059986	7136.64159
cg23615572	0.01334372	2362971.48	21.1440858	2.6408E+11
cg02208899	0.01334587	131366906	48.7383391	3.5408E+14
cg21817720	0.01336064	2067.29446	4.8850385	874856.23
cg21083314	0.01336362	61675.3069	9.88912827	384649018

cg21084516	0.00729956	15.6417322	2.09797204	116.619184
cg20001829	0.00730102	15.8372264	2.10489929	119.159022
cg12749863	0.00731064	3.43632918	1.3943651	8.46862723
cg24566687	0.00731232	75.8056733	3.20764342	1791.50216
cg13883681	0.00731268	41.4733465	2.72673411	630.805351
cg23338737	0.00731365	443896184	213.053406	9.2486E+14
cg23265500	0.00731781	8.76583905	1.79399354	42.8317787
cg16300531	0.00731976	8.01489052	1.75116593	36.683257
cg22983529	0.00732958	11.9019266	1.94725311	72.7465047
cg24201890	0.00733817	18.0046713	2.17600254	148.974177
cg22638491	0.00734047	9.67900259	1.84132113	50.8781925
cg09461851	0.0073438	0.14421195	0.03500629	0.59409567
cg18711066	0.00734502	24.534411	2.36422059	254.60286
cg20587543	0.00735234	1735.53574	7.42567797	405630.883
cg22761315	0.00736546	23589.3095	14.9500263	37221039.7
cg20275528	0.0073656	5.01636738	1.54221281	16.3167765
cg16496928	0.00736697	10676.6633	12.080439	9436009.57
cg03988279	0.00737593	2.2134E+10	599.306824	8.1746E+17
cg04468551	0.00738178	30.0431192	2.49268579	362.094981
cg18813989	0.00738258	4457152.62	60.9150313	3.2613E+11
cg06553210	0.00738452	3960.37599	9.23867837	1697707.98
cg23187101	0.00738507	112607728	144.855654	8.7539E+13
cg15639045	0.00738939	20.0380905	2.23527763	179.630962
cg15799267	0.00739598	20.8456321	2.25854221	192.398607
cg21714061	0.00740147	15.0796196	2.07026228	109.838705
cg05852040	0.00741148	68.1241982	3.10052441	1496.81337
cg13661243	0.00741575	10.3169861	1.86913084	56.9463623
cg26980034	0.0074179	42.9274723	2.73868443	672.866088
cg25148255	0.00742216	7.8811E+10	830.814409	7.476E+18
cg27494647	0.00742463	6.17592861	1.62864903	23.4194683

cg15458254	0.01336645	9.81272688	1.60702571	59.9179019
cg20474425	0.01336936	7.46724955	1.51830312	36.7250882
cg10334703	0.01338882	15.422873	1.76436396	134.816295
cg13934277	0.0133935	1814650.88	19.8920883	1.6554E+11
cg26887625	0.01339728	61886034.6	41.3503994	9.262E+13
cg25490585	0.01340567	796922466	70.1602162	9.0519E+15
cg16214500	0.0134119	609138.408	15.8270876	2.3444E+10
cg04453677	0.01341746	353867.147	14.1329014	8860314962
cg24568647	0.01342346	1.2557E+11	199.590996	7.8996E+19
cg27217148	0.01342626	35.8569266	2.09958784	612.367418
cg10638827	0.0134263	36.9630828	2.11284519	646.649125
cg16903217	0.01344492	118.311419	2.68680568	5209.75219
cg06726056	0.01344586	4281381443	98.6772793	1.8576E+17
cg08499107	0.01345335	1730963.4	19.5540868	1.5323E+11
cg05964918	0.01345531	1694.19759	4.65903709	616072.68
cg03008269	0.01346465	633204.165	15.8591041	2.5282E+10
cg03945021	0.01346596	15.754632	1.76895954	140.313232
cg01803049	0.01346875	0.00948973	0.00023601	0.38157991
cg04579966	0.01346968	3.55069993	1.29966256	9.70057183
cg20384898	0.0134885	5.61861171	1.42869042	22.0963179
cg15888699	0.01348856	164174.569	11.9654825	2252586915
cg18202751	0.01349013	45381.3807	9.17167603	224546714
cg22345692	0.01350396	14.1022918	1.72739375	115.12988
cg16036409	0.01351154	9.31465295	1.5853442	54.7280265
cg16411251	0.01351758	13.940063	1.72274428	112.799886
cg05302420	0.01352203	4.1637E+11	250.19229	6.93E+20
cg07478122	0.01353673	0.08127543	0.01108583	0.59586864
cg26341831	0.01353781	26.4823053	1.96566174	356.781882
cg10999157	0.01353931	3203.08842	5.28492086	1941330
cg22137448	0.01354865	31552.4978	8.46423796	117619580

cg17071957	0.00743011	16.4803238	2.11806968	128.23047
cg10565216	0.0074323	119.249437	3.598171	3952.12684
cg08738571	0.00744189	28.9085264	2.46090464	339.591744
cg15628498	0.00744273	282021949	182.779173	4.3515E+14
cg16601494	0.00744577	3.413	1.38895158	8.38659111
cg12317815	0.00744632	89.6970812	3.33137116	2415.09156
cg08331344	0.00745357	1.15E-11	1.12E-19	0.0011845
cg19635869	0.00745551	111.754769	3.53139885	3536.59524
cg23239444	0.00745814	20.9136473	2.25525213	193.938689
cg16898498	0.00746152	10.0951612	1.85585268	54.9139921
cg27476456	0.00746315	10.099235	1.85596697	54.9549362
cg03457528	0.0074659	226.354788	4.26232113	12020.7953
cg07830449	0.00746895	17.7796564	2.15858677	146.4459
cg20265748	0.00747902	10.5302156	1.87596274	59.1085514
cg12118857	0.00748574	1.63E-06	9.30E-11	0.02841097
cg11449134	0.00749456	27.4106192	2.42090805	310.355465
cg03317826	0.00749543	3735118616	359.801183	3.8775E+16
cg15916004	0.00749547	9.31397438	1.81461591	47.8063255
cg03086376	0.00749778	860951.741	38.4262337	1.929E+10
cg10030624	0.00749944	39.4755036	2.66798824	584.078806
cg00528600	0.00750005	102445285	137.582566	7.6282E+13
cg18625286	0.00750202	1.0352E+17	34841.2174	3.08E+29
cg04060561	0.0075029	10.9533317	1.8944867	63.3287501
cg18014543	0.00750382	80.6619962	3.22801765	2015.58924
cg01348757	0.00750484	60.3408598	2.98721024	1218.86947
cg03640883	0.00750808	0.10051951	0.018654	0.54166243
cg03847535	0.00750848	17.6919812	2.15273384	145.399395
cg12394377	0.00750975	48.7719962	2.82156872	843.044365
cg05590982	0.00753121	7.77972821	1.7279099	35.0273883
cg02006107	0.00753358	10.9344557	1.89191472	63.1964639

cg15372603	0.01355104	7.46726808	1.51358345	36.8397875
cg02334620	0.01355898	2.3476E+10	137.167519	4.0177E+18
cg22817039	0.01356197	215558108	52.1470886	8.9104E+14
cg05508558	0.01356224	69.6267042	2.39729552	2022.22792
cg00478851	0.01356287	331.639917	3.30669344	33261.3338
cg08962271	0.01356537	58.5028507	2.31257996	1479.98495
cg14925616	0.01357229	53.3253359	2.26831829	1253.61218
cg04460557	0.01357402	21.1418454	1.87467465	238.42944
cg20773694	0.01358042	600300.726	15.4809203	2.3278E+10
cg25074426	0.013591	6.77828373	1.48271407	30.9871818
cg14579819	0.01359739	18.281551	1.81833386	183.802938
cg01303055	0.01360814	17.4156059	1.79980027	168.520548
cg05542338	0.01360824	5.7495146	1.43295648	23.0690315
cg08693600	0.01360831	506377764	61.6936711	4.1563E+15
cg02605237	0.01360876	941545.123	16.9282189	5.2369E+10
cg09161542	0.0136152	102.921469	2.59294053	4085.25713
cg17934743	0.01362292	7.0373E+11	272.381733	1.82E+21
cg23709172	0.01362431	14.0986752	1.72264031	115.388362
cg20320141	0.01363919	1865.73971	4.69740134	741044.765
cg14732852	0.01364016	16632.1651	7.36173624	37576586.2
cg07068756	0.01364514	4.25136512	1.34608618	13.4271533
cg11340260	0.01364632	30.0973781	2.01192522	450.24147
cg24740531	0.01365119	5.45279791	1.41655187	20.9897044
cg23275064	0.01365288	0.11632341	0.02104482	0.64296755
cg03744763	0.01366213	18.7699564	1.82529079	193.016512
cg27332337	0.0136773	23.0705409	1.9034588	279.622472
cg08734623	0.0136779	165.492291	2.85120172	9605.66841
cg22934035	0.01368267	18.5221837	1.81939864	188.563012
cg04491089	0.01368431	14.5293784	1.73097673	121.955906
cg24925865	0.01370406	30669360.2	34.1762006	2.7522E+13

cg04528060	0.00753385	3.98827051	1.44591597	11.0008479
cg13783444	0.00753745	1099.54904	6.46430261	187028.386
cg03905819	0.00753923	8.25033492	1.75481452	38.7892998
cg11155735	0.00754402	9.30174184	1.8115767	47.7608268
cg27352765	0.0075454	135.748198	3.69992767	4980.52256
cg11779239	0.00754693	10.0230754	1.84783559	54.3674133
cg24930197	0.00754823	229397.194	26.7890813	1964347781
cg25217365	0.00755334	4.95985524	1.53184983	16.0591224
cg12901910	0.00755723	11.0861885	1.89755867	64.7693152
cg09857708	0.00755728	35594.707	16.2891763	77780677.6
cg14182820	0.00755776	0.08261142	0.01325666	0.51480915
cg25924823	0.00756223	0.00011749	1.54E-07	0.0899077
cg26379822	0.00756234	26.0004342	2.38056568	283.975604
cg13654253	0.00756336	3.4568E+12	2176.45327	5.49E+21
cg20303021	0.00757027	2593794943	320.008353	2.1024E+16
cg03739476	0.00757369	45.6564567	2.76403985	754.154119
cg14430111	0.00757554	239030.899	26.9727908	2118274348
cg02300764	0.00758083	411.823472	4.95986284	34194.2061
cg20034091	0.00759713	10.3815157	1.86254547	57.8648256
cg02764250	0.00760003	0.02576279	0.00175495	0.37820011
cg17300750	0.00760054	11.9666868	1.93403679	74.0428478
cg00679711	0.00760067	0.0374447	0.00335653	0.4177244
cg16714002	0.00760146	67.3534335	3.06093785	1482.0572
cg04282419	0.00760618	58.6988824	2.95040029	1167.82757
cg14714537	0.00760676	12.5225976	1.95714159	80.1247348
cg08409816	0.00761155	25.7637689	2.3701619	280.053353
cg07229893	0.00761192	0.01345296	0.00056837	0.31842267
cg03928367	0.00761852	5.66889573	1.5851591	20.2732829
cg24881993	0.00762293	163.772049	3.87095519	6928.85419
cg16784205	0.00762359	69310.5103	19.2777668	249196232

cg24521811	0.01371176	39.9982158	2.12857797	751.608485
cg01850934	0.01373135	28046.8239	8.13146425	96738337.4
cg23239444	0.01374261	28.9532366	1.99052663	421.139761
cg24277128	0.01374595	90162435	42.3502217	1.9195E+14
cg14653418	0.01374669	6735.41424	6.06599896	7478703.07
cg20140110	0.01375065	7.66348794	1.51647812	38.7272634
cg26861214	0.01375109	0.00544296	8.60E-05	0.34439843
cg17279839	0.01376481	12.8205033	1.68421747	97.5914977
cg05774290	0.01377419	178.435925	2.8834227	11042.217
cg05951609	0.01377502	6.8759127	1.48263708	31.8878949
cg02786012	0.01377663	8.58481468	1.55136204	47.5060245
cg27117639	0.01378481	3.05E-12	2.09E-21	0.00445501
cg18532308	0.01379103	19.7582541	1.83864541	212.324032
cg10105971	0.01379786	10.6250892	1.61973458	69.6981607
cg24516766	0.01380627	7.039E+11	261.148018	1.90E+21
cg01122672	0.01381696	693.420763	3.79583135	126673.793
cg04001842	0.01381746	140.149844	2.73969435	7169.40519
cg09857708	0.01382864	37889.5678	8.5734564	167449309
cg21289924	0.01382899	6618.92412	6.00772579	7292302.94
cg03517998	0.01382949	0.133331	0.02680435	0.66321895
cg15590133	0.01382985	0.02745942	0.00156884	0.48062104
cg05291965	0.01384631	197.459477	2.93430506	13287.7272
cg00106250	0.01384981	7060166.39	24.8114978	2.009E+12
cg13556548	0.01385069	6.9473277	1.48394121	32.5251174
cg03463411	0.01387052	20.5365677	1.84947239	228.03834
cg23585979	0.0138754	15.9459852	1.75647967	144.76367
cg18894815	0.01387637	6.82498522	1.47797147	31.5164562
cg09039561	0.01387865	10.4711075	1.61234869	68.0027171
cg17470143	0.01387911	16.9598922	1.7784878	161.73175
cg21646955	0.01387991	7.37259248	1.5012687	36.2061234

cg08539350	0.00762956	423.527319	4.97910724	36025.6129
cg20967975	0.00763512	11.6510872	1.91837068	70.7620455
cg16039972	0.00763759	6.66E-05	5.68E-08	0.07797113
cg24364084	0.00763846	209303.479	25.7937333	1698394947
cg19378640	0.00763907	6.62710952	1.65149652	26.593202
cg18229767	0.00764616	11.9846218	1.93214874	74.3375277
cg16570157	0.0076469	3.80149709	1.42492846	10.1418286
cg01243246	0.00765151	171.424261	3.91119313	7513.37925
cg13939859	0.00765353	5.89010177	1.60014855	21.6812988
cg13048591	0.00766024	45.8220625	2.75560923	761.95906
cg17679455	0.00766136	125782.983	22.4611318	704388320
cg04460557	0.00767027	15.4115169	2.06375699	115.088575
cg13982823	0.00767169	17510.9377	13.3037391	23048628.4
cg16412592	0.00767377	1053393.19	39.3659877	2.8188E+10
cg21856334	0.00767584	0.10201158	0.01904757	0.54633546
cg02532824	0.00768023	12.1902379	1.93888653	76.642907
cg26827247	0.00768482	5.10723261	1.53983067	16.9394112
cg15239874	0.00768571	1068483402	245.484319	4.6506E+15
cg20359237	0.00768604	125.655751	3.59476702	4392.32022
cg00237391	0.00768894	7.87787889	1.72685343	35.9387629
cg18632254	0.00769552	11393396.7	73.6455359	1.7626E+12
cg08315133	0.00769604	1246.69716	6.59325412	235733.946
cg26702921	0.00769699	0.07985211	0.01244519	0.51235531
cg00002719	0.0076994	4.85361149	1.51878562	15.5107766
cg08801795	0.00770179	15.8406546	2.07664278	120.832693
cg11983038	0.00770266	9.09126983	1.79293129	46.0983573
cg01758899	0.00770454	13.8763314	2.00500085	96.0361545
cg01056889	0.00770664	6.49199748	1.6399906	25.6989469
cg23574802	0.00771173	22.1454412	2.26825117	216.210874
cg09689449	0.00771912	109.895849	3.4629853	3487.48162

cg16464045	0.01388133	41.8921806	2.13743822	821.055214
cg01022087	0.0138853	0.02706057	0.00152559	0.47999435
cg19025234	0.01388752	12.7983915	1.67921022	97.5451571
cg06116236	0.01389179	0.08419048	0.01172199	0.60467861
cg20532887	0.01390374	39330.2545	8.58161507	180253822
cg08993385	0.01390946	21.112092	1.8580546	239.885539
cg25746764	0.01391102	1432.6475	4.37603603	469026.959
cg06440553	0.01391815	799.111739	3.88517009	164363.35
cg09262011	0.01391977	261.799352	3.09717285	22129.5046
cg06033764	0.01393275	0.04455438	0.00373229	0.53186998
cg18798248	0.01393352	5.17924299	1.3962037	19.212496
cg18785300	0.01393797	37319.0554	8.46505089	164524929
cg22248683	0.01395158	1482849.86	17.8399502	1.2325E+11
cg05997131	0.01395222	17790.4398	7.27516893	43504109.7
cg11580511	0.01395638	464250.897	14.0894688	1.5297E+10
cg26345046	0.01396202	14.0189813	1.7077913	115.079541
cg02176069	0.01396547	17.6794636	1.78986823	174.629299
cg07903860	0.01396719	3382412761	85.3345059	1.3407E+17
cg23980468	0.01397072	8.82836913	1.55473902	50.1306654
cg00262061	0.01397356	101839.834	10.3419505	1002842918
cg11420031	0.01397668	9765.01004	6.42987671	14830054.3
cg19427757	0.01398444	1.3708E+10	112.934618	1.6638E+18
cg15130433	0.01398753	5970745.05	23.5522376	1.5136E+12
cg14633020	0.01398837	25572.9875	7.80676133	83770677.7
cg01645998	0.0139965	7.38013484	1.49866172	36.3433518
cg03615240	0.014	34634920.7	33.5615772	3.5743E+13
cg12487497	0.01400484	1164738830	68.3049386	1.9861E+16
cg26631984	0.01401194	7.32176793	1.49587225	35.8374759
cg20256260	0.01402369	19.5335471	1.82378201	209.213306
cg20698170	0.01402635	201393.959	11.8104374	3434210370

cg06949933	0.00772005	11.3395843	1.89988927	67.6808772
cg24680586	0.00772138	3.87347707	1.43029185	10.4900441
cg13567541	0.00772639	6.96722284	1.67014787	29.0646085
cg21440584	0.00772708	6.26521573	1.62390812	24.1718899
cg01024168	0.00773577	7.21323255	1.68515583	30.8759124
cg23022999	0.00773787	23.5259355	2.30252085	240.375517
cg25262261	0.00773878	0.09088213	0.01555947	0.53083823
cg12451530	0.00773901	7.21381732	1.68506252	30.8826288
cg26616378	0.00774223	148670.802	23.2076706	952400948
cg19510057	0.00774493	4.1695E+11	1168.67423	1.49E+20
cg01918706	0.00774805	699.357766	5.63497425	86797.4302
cg10245865	0.0077506	28628617.9	92.914214	8.821E+12
cg05516012	0.00775311	1.6389E+11	911.030716	2.9483E+19
cg20439288	0.00775498	555581399	203.025886	1.5204E+15
cg11529190	0.00775651	43814.4023	16.7779253	114418309
cg12507643	0.00776518	11.6794868	1.9122283	71.3358396
cg02067430	0.0077769	9018939522	421.052507	1.9319E+17
cg19915711	0.00777775	9.78880482	1.82457601	52.5166937
cg16857771	0.00777866	10.201428	1.84449623	56.4214398
cg05399707	0.0077789	178297941	149.601417	2.125E+14
cg13801056	0.00777903	15.6854259	2.06595716	119.088909
cg00809888	0.00778773	188.770355	3.97811368	8957.57384
cg14277108	0.00778868	1773.47582	7.17767531	438194.311
cg22517351	0.00779739	1065680001	238.567754	4.7604E+15
cg23801544	0.00779751	18.5564691	2.15815483	159.554143
cg11555919	0.00779877	14.1751011	2.01029243	99.9523696
cg19848291	0.00779891	261.280998	4.3306323	15763.9244
cg00429081	0.00780214	19258.3128	13.4338154	27608136.6
cg14210494	0.00780841	3407585.47	52.4317423	2.2146E+11
cg20183756	0.00781397	0.06447682	0.00855342	0.48603517

cg04665974	0.01405869	478150.541	14.0169563	1.6311E+10
cg12419678	0.01405956	862.849288	3.91487681	190174.284
cg25390230	0.0140685	7.6715E+10	157.28028	3.7419E+19
cg27144788	0.01407666	414143.485	13.5898554	1.2621E+10
cg13002957	0.01408475	22.855396	1.8796358	277.909754
cg19844955	0.01408537	0.08302324	0.01138581	0.60539028
cg18014543	0.01410365	151.489271	2.75033806	8344.06485
cg11724135	0.01410447	6.43206348	1.45509604	28.4321031
cg05280555	0.01411559	406.404519	3.3534657	49251.9226
cg09298971	0.01411755	18.0143155	1.79013143	181.2803
cg09668408	0.01413546	7.4173E+11	244.871487	2.25E+21
cg13753515	0.01413913	25.6871012	1.9216243	343.369495
cg06734406	0.01414726	4.83037287	1.37273679	16.9970691
cg11188483	0.01415958	106.239513	2.55502103	4417.51124
cg12120360	0.01416006	0.08322004	0.01141681	0.60661206
cg09704415	0.01416985	72.6609954	2.36627573	2231.19402
cg22545356	0.01417144	1167.53762	4.13417147	329726.064
cg00213177	0.0141737	18.9090429	1.80522518	198.064988
cg17167832	0.01418259	94.7925041	2.49496077	3601.50707
cg26847756	0.01418777	3476.88159	5.14218431	2350889.21
cg13425174	0.01418818	64.7117909	2.3103543	1812.54273
cg08382220	0.01419305	31.0544889	1.99335995	483.79686
cg22409775	0.0141941	6.3077E+10	147.327291	2.70E+19
cg27614280	0.01419969	2.91E-05	6.89E-09	0.12288222
cg21961890	0.01420547	808742959	61.3166861	1.0667E+16
cg10799705	0.0142175	296523.501	12.5188483	7023504437
cg08500417	0.01422309	51.3619441	2.20310356	1197.4241
cg03079712	0.01422443	303.455268	3.14562557	29274.0181
cg11612345	0.01422494	11.3271729	1.62692249	78.8635264
cg16053142	0.01422758	74131.9495	9.47113143	580241756

cg10126234	0.00781933	5.4616821	1.56313654	19.0834074
cg14779329	0.00783424	21.8206381	2.24920672	211.692523
cg26982447	0.00784168	21.5247323	2.24053748	206.787035
cg03383158	0.00784241	474.218884	5.05052715	44526.748
cg18270343	0.00784541	8.99183883	1.78102141	45.3970767
cg04822330	0.00784931	6.78803877	1.65402402	27.8578
cg08430407	0.00785438	34.9336899	2.54329203	479.83585
cg24394414	0.00785747	6411257.57	61.3580931	6.6991E+11
cg23845770	0.00785977	0.11232632	0.02240434	0.56315896
cg00758533	0.00786579	38.6086828	2.60971036	571.18614
cg26305756	0.00787403	0.11770589	0.0242925	0.57032737
cg20344434	0.00787467	668067468	207.047767	2.1556E+15
cg03361102	0.00787551	13.4818288	1.97920527	91.8346924
cg23588217	0.00787594	10.3323402	1.84569864	57.8411081
cg26928858	0.00787961	138.83182	3.64896596	5282.11952
cg01267709	0.00788	8.8111854	1.76996242	43.8636366
cg20690916	0.00788114	357.159616	4.67521593	27284.9411
cg00727590	0.00788267	17.1555593	2.10790869	139.623323
cg01659099	0.00788449	5.67779291	1.57705279	20.4415049
cg02879798	0.00788544	58.2848118	2.90498331	1169.41095
cg09615786	0.0078946	1427.91084	6.7174398	303527.749
cg07036561	0.00789555	1299.2171	6.55258687	257602.853
cg12743398	0.00789923	10.6333579	1.85844625	60.8402316
cg26444282	0.00790135	162.89976	3.80029601	6982.7013
cg15689224	0.00790299	507.49481	5.11835293	50319.1136
cg00463577	0.0079039	55.37732	2.8637302	1070.85771
cg13866214	0.00790449	13.719189	1.98650188	94.7475306
cg25830305	0.00790502	7.94118671	1.72127593	36.6370349
cg04361056	0.0079151	36147.2526	15.6343426	83573956.2
cg20918957	0.00791739	6.40982865	1.62685097	25.254866

cg01348086	0.01424431	20.8637304	1.83800525	236.830251
cg06460983	0.01424945	15.6581515	1.73508826	141.305612
cg06804210	0.01426298	13.4229591	1.68188567	107.127277
cg27487704	0.01426508	566192.486	14.1791493	2.2609E+10
cg11073923	0.01426735	6211.27662	5.74463533	6715823.55
cg09541379	0.01427323	77.7099651	2.38954915	2527.18747
cg00969988	0.01427849	50124.3304	8.71675487	288232092
cg02372786	0.01428107	12.1647249	1.64845497	89.7692285
cg02556718	0.01428461	251655.027	12.0305512	5264118953
cg14367396	0.01430176	333922.405	12.7079872	8774337817
cg04117530	0.0143075	6.3117919	1.44510136	27.5681126
cg27298243	0.01430819	7.64197812	1.50137832	38.8974777
cg25985355	0.01431754	9.20637099	1.55804048	54.3999131
cg02304134	0.01432109	1223.39264	4.13673757	361804.328
cg10622825	0.01433033	83470.6954	9.60660433	725267405
cg22817638	0.01433549	23.3222871	1.87505302	290.087304
cg04466743	0.01436277	20.6489894	1.82879711	233.148204
cg05890484	0.01437064	13.3729421	1.67677681	106.654373
cg16642791	0.01438869	8.13054522	1.51798308	43.5484206
cg13901143	0.01440046	2388.53847	4.70425606	1212756.27
cg08633134	0.01440787	13202776.5	26.1271102	6.6717E+12
cg04220104	0.01443041	0.11251754	0.01954713	0.64767531
cg02373393	0.01443357	3660034.19	20.1755747	6.6396E+11
cg26875073	0.01443493	9.63824243	1.56894819	59.2089133
cg02359414	0.01444339	6.2558E+11	220.883311	1.77E+21
cg20065569	0.0144442	327522.389	12.4725374	8600568725
cg02641288	0.0144698	22.9190101	1.86210924	282.089262
cg24272907	0.01447458	0.12152897	0.02243981	0.65817359
cg02819828	0.01449195	7.90309791	1.50679622	41.4514953
cg13065262	0.0144972	1592.55419	4.31433838	587860.435

cg10929210	0.00791919	13.5727264	1.98002231	93.0388013
cg04220104	0.00792772	0.12711562	0.02772918	0.58272112
cg06659904	0.00793084	29537656.9	90.2866765	9.6634E+12
cg16937168	0.00793112	6.60075316	1.63890444	26.5847974
cg05123019	0.00793458	1262.16464	6.48164162	245780.262
cg02927346	0.00793498	10.9236353	1.86972115	63.8201099
cg25170017	0.0079359	17.9569929	2.12942847	151.427295
cg15894039	0.00793733	5.0022E+13	3847.40518	6.50E+23
cg13682722	0.00793825	265.995147	4.31102249	16412.2127
cg19812495	0.00793856	113927.266	21.0514521	616557087
cg07048306	0.00793867	218.057414	4.09245107	11618.7182
cg21184629	0.00793978	5402.31425	9.47839197	3079108.7
cg22752008	0.0079404	106.928258	3.39589506	3366.90388
cg26360644	0.00794296	1.64E-05	4.79E-09	0.05595486
cg15087448	0.00795119	22.546278	2.25885204	225.041145
cg25886621	0.00796204	7.47151736	1.69169642	32.9985753
cg12014818	0.00796493	10.0856679	1.82957221	55.5980777
cg12639429	0.00796558	10.8744522	1.86590468	63.3760726
cg11352083	0.00796913	12.4697627	1.93367049	80.4144154
cg23447841	0.00796922	0.11073471	0.02179342	0.56265493
cg13141289	0.007973	7415.90932	10.261716	5359309.41
cg15489301	0.00798041	41.5177272	2.64655315	651.308164
cg23845806	0.0079817	16973.8415	12.7276672	22636614.5
cg26436829	0.00798174	379536855	174.082832	8.2747E+14
cg19716887	0.00798394	44.3809218	2.69263278	731.501984
cg06804210	0.00798949	10.7812514	1.86046506	62.4765194
cg24631870	0.00799321	340371188	168.752207	6.8652E+14
cg03296761	0.00799442	21.5522455	2.22887983	208.400327
cg00398048	0.00800623	19.7791103	2.17858122	179.572467
cg13804854	0.00800729	22009.9851	13.5804265	35671887.2

cg22525895	0.01449757	12.869985	1.65960522	99.8047689
cg17208816	0.01450416	563.88805	3.51036938	90580.1353
cg26379859	0.01451052	5.51235439	1.40253097	21.6651551
cg09254001	0.01451307	1139322.49	15.853811	8.1877E+10
cg18233786	0.01451531	88.1587902	2.42896741	3199.70216
cg11176186	0.01451549	6436262.25	22.3360506	1.8546E+12
cg09470142	0.01451669	132217.146	10.342831	1690192343
cg20439288	0.01453295	706536511	56.4983144	8.8356E+15
cg04427651	0.01453668	23.8259766	1.87326666	303.041299
cg14528930	0.01454213	13020049.6	25.5909239	6.6243E+12
cg14200826	0.01454795	4.0613E+12	312.493831	5.28E+22
cg16523137	0.01455619	795.816285	3.747842	168983.527
cg07816439	0.01455692	73.4146289	2.3390582	2304.22131
cg26211724	0.01455949	11.6045837	1.62388532	82.9284934
cg14244577	0.0145792	31.334724	1.9753045	497.070162
cg24377657	0.01457926	0.00054376	1.31E-06	0.22639996
cg24828620	0.01458234	6.4757275	1.44644005	28.991901
cg27200466	0.01458607	14.3832507	1.693335	122.171867
cg07537523	0.01458659	56.0866942	2.21560496	1419.8006
cg03033975	0.01459712	2660.91994	4.74604399	1491873.01
cg27557378	0.01459769	9.30086115	1.55327077	55.6928126
cg01119072	0.01459814	113.688934	2.54636152	5075.93825
cg16425829	0.01461083	0.11670637	0.02081151	0.65446374
cg25446086	0.01461677	37.6032252	2.04552144	691.267528
cg02555579	0.01461938	148.833357	2.68314633	8255.74362
cg14761923	0.01462142	13324.4517	6.51132247	27266505.9
cg10981962	0.01463247	0.04277249	0.00340593	0.53714653
cg11930554	0.01463305	4.55329231	1.34835947	15.376071
cg26072749	0.01464598	8.14963416	1.51202365	43.9255942
cg01059295	0.01464827	0.10700418	0.01778525	0.64378588

cg20132549	0.00802131	78384.1636	18.8804501	325420055
cg13308525	0.00802159	656790.342	32.861513	1.3127E+10
cg25507767	0.00802172	16.8119686	2.08705878	135.426128
cg08810582	0.00802348	43.2077753	2.66918348	699.431818
cg15372603	0.00803275	6.39344307	1.62166534	25.2062576
cg05361096	0.00803288	6.04868171	1.59840454	22.8894185
cg04822518	0.00804295	12.9590148	1.94887905	86.1705941
cg23633635	0.00804538	1335552107	238.037362	7.4934E+15
cg09778958	0.00804551	4871.76728	9.12848716	2600005.45
cg26931202	0.00804774	11.5672002	1.89178972	70.7267401
cg21083314	0.00804795	43209.7063	16.1112816	115886418
cg11992317	0.00804913	5425222.6	56.7002961	5.191E+11
cg27109877	0.00805345	14.8937508	2.02013775	109.806281
cg17444839	0.00805419	6.65482045	1.63790583	27.0385723
cg26081613	0.00805578	31372223.8	89.4237331	1.1006E+13
cg22540912	0.00806331	1384.12716	6.56771192	291700.979
cg16201038	0.00806797	4.91488184	1.5132534	15.9629997
cg13843603	0.00806809	2.218E+10	491.702733	1.0005E+18
cg24973864	0.00806873	0.18880947	0.05500456	0.64811023
cg03512414	0.00807735	11.7712234	1.89879998	72.9732997
cg09913183	0.00807929	416882.397	28.934796	6006295424
cg26032238	0.00808027	8.69015372	1.75457779	43.0409939
cg01593886	0.0080863	29.5806061	2.41211584	362.757146
cg15600915	0.00808896	13.7877304	1.97780441	96.1174465
cg05302420	0.00809361	2.5965E+11	925.282778	7.2864E+19
cg06459662	0.00810104	47334.978	16.3885422	136717477
cg00752778	0.00810367	505.120834	5.03753145	50649.2236
cg10912268	0.00810438	557.401281	5.167796	60121.6045
cg22376864	0.00810611	12.3251597	1.92002413	79.1185687
cg24868790	0.00811235	15.9327089	2.05200197	123.709049

cg04722722	0.01464941	40.1127081	2.06973692	777.407668
cg15929437	0.01465102	1722725.43	16.9324618	1.7527E+11
cg19809499	0.01465208	4.03372435	1.31624561	12.3616231
cg13979592	0.01466025	1.0722E+11	148.754124	7.7287E+19
cg20377955	0.01466051	9.67982754	1.56375745	59.9191782
cg04896048	0.0146631	0.11921392	0.02160502	0.65780835
cg27102995	0.01466356	566.54757	3.48489335	92105.0135
cg07633851	0.01467733	19.0900006	1.78683222	203.952066
cg04337534	0.01468616	50.5305908	2.16354411	1180.16573
cg27079322	0.01468676	16.6588164	1.73918743	159.566564
cg23609985	0.01470794	8.62632688	1.52740445	48.7189332
cg16480966	0.01471153	3401861388	74.7068276	1.5491E+17
cg16542392	0.01471312	299361599	46.3233034	1.9346E+15
cg05663031	0.01471791	12.3602215	1.63896656	93.2142723
cg14621323	0.01473155	19.7479844	1.7964365	217.086931
cg00980538	0.01473281	577331527	52.5363473	6.3444E+15
cg09925572	0.01473316	1.2627E+10	96.2814338	1.6561E+18
cg26058820	0.01473787	0.13517315	0.02706514	0.67510385
cg06328288	0.01474037	411.358065	3.25978068	51910.0744
cg00599564	0.0147411	9.89598897	1.56822818	62.4466511
cg06632353	0.01474114	1602634	16.5200306	1.5547E+11
cg24371438	0.01474572	6500549.48	21.7336224	1.9443E+12
cg12439423	0.01475186	32.4290127	1.97909138	531.375597
cg00169792	0.01475488	9.31110869	1.54920078	55.9622393
cg23034373	0.0147561	18.0510256	1.76400516	184.715745
cg00034416	0.01475796	502420.816	13.1324709	1.9222E+10
cg21682062	0.01477059	7.7438E+13	528.569913	1.13E+25
cg21992932	0.0147715	198.409015	2.8212962	13953.2097
cg25815972	0.01477188	7.0099E+10	133.759412	3.6737E+19
cg21188037	0.0147835	6.78568471	1.45531736	31.6395022

cg15344234	0.00811524	745.779106	5.56909174	99870.2305
cg02649547	0.00811883	79.3948593	3.11273938	2025.07917
cg05378295	0.00811937	232557.817	24.7169843	2188096152
cg02791024	0.00812393	1.5678E+10	442.444164	5.5553E+17
cg27090024	0.00812483	124393.621	20.996047	736985062
cg16052317	0.00812596	7.28134355	1.67393621	31.672631
cg05497345	0.0081348	4.94117976	1.51346937	16.1319799
cg24684765	0.00813656	1293.48065	6.41356719	260867.649
cg19750232	0.00813832	647398.062	32.1461906	1.3038E+10
cg07846311	0.00813844	6.26001739	1.60912956	24.3534261
cg14960043	0.00814054	14.2290131	1.99088649	101.69581
cg16113047	0.00814737	743.386972	5.55070883	99559.2106
cg17536822	0.00815789	19.5616468	2.16089018	177.083514
cg17306261	0.00815917	17.5593016	2.10117293	146.741406
cg12142869	0.0081625	0.0178618	0.00090516	0.35247425
cg14453612	0.0081644	40181.5313	15.5833336	103607835
cg25059792	0.00818406	423.137644	4.78392048	37426.5138
cg18685956	0.00819337	5786.86677	9.40560545	3560411.62
cg05081953	0.00819404	57.1613811	2.84818906	1147.19333
cg20786557	0.00819687	252558.999	24.9708513	2554420241
cg24138862	0.00819886	154.352533	3.68174962	6471.02786
cg00145141	0.00820265	9.00975768	1.76560094	45.9762633
cg00088688	0.00820337	14.1743021	1.98505844	101.21155
cg06001881	0.00820881	3.1343E+13	3084.55259	3.18E+23
cg11870261	0.00820998	11.9938877	1.90077967	75.6812296
cg03079681	0.00821083	445301.013	28.8555256	6871924456
cg01721544	0.00821192	9.83777027	1.80576182	53.5960629
cg15225657	0.00821358	6.0988E+15	12026.2724	3.09E+27
cg26923779	0.00821553	9.1806E+12	2240.35887	3.76E+22
cg14528525	0.00821706	6761597526	347.096665	1.3172E+17

cg01851874	0.01478403	13.4028248	1.66294811	108.02244
cg22697962	0.0147919	7.33490036	1.47748314	36.4137917
cg15068083	0.01481816	4712239421	78.1351852	2.8419E+17
cg14923274	0.01483899	5353119.76	20.6784607	1.3858E+12
cg03949306	0.01484135	7427.47924	5.7110945	9659697.94
cg13538257	0.01484447	451.189221	3.30247606	61642.1467
cg11555919	0.01485015	19.4050624	1.78519697	210.932717
cg07569020	0.01485257	24.581079	1.86950258	323.203322
cg04953552	0.01486445	10.8271555	1.59238196	73.6175745
cg13716638	0.01486595	30938.4303	7.53301518	127065517
cg10276465	0.01489337	75.2324626	2.32293919	2436.53534
cg12946524	0.01489705	47699.2486	8.17556085	278295075
cg09042952	0.0149097	1437.36969	4.12615275	500716.225
cg02002664	0.01491111	371755.43	12.1848068	1.1342E+10
cg00415978	0.01491113	10.7087487	1.58755353	72.2352335
cg13929970	0.01491973	17.2862974	1.74253506	171.483539
cg24463605	0.01491974	17.7630415	1.75179716	180.115398
cg24441185	0.01492212	744365.333	13.9340536	3.9764E+10
cg20829550	0.01492512	8.53647625	1.51855625	47.9873083
cg21199629	0.01492785	121.910797	2.54891463	5830.81214
cg15648905	0.01493087	5472980.53	20.5316537	1.4589E+12
cg02233421	0.01493302	369214.299	12.1411433	1.1228E+10
cg13243329	0.01494117	1.1015E+10	90.1735369	1.3455E+18
cg04858631	0.01494238	50.1400231	2.14285702	1173.21029
cg00701946	0.01495202	9.82585739	1.5599788	61.890247
cg16416718	0.01495579	1427211.49	15.757912	1.2926E+11
cg25168729	0.01495752	1187897052	58.2886057	2.4209E+16
cg20844262	0.01496674	240018.957	11.1270502	5177391934
cg05799276	0.0149695	4.7756E+12	292.151108	7.81E+22
cg03834567	0.01497131	69.1565075	2.27904853	2098.5172

cg23734074	0.00822108	274834.143	25.4334673	2969858787
cg07244253	0.00823089	298.214858	4.35627376	20414.7183
cg25756470	0.00823246	45.5466574	2.68109212	773.75111
cg11892243	0.00823698	6574735.66	57.6002386	7.5047E+11
cg18394090	0.00824562	7570.75585	10.0284651	5715365.59
cg19910323	0.00824749	433369.993	28.4966294	6590588244
cg24304712	0.00826173	6271.66689	9.53734094	4124189.94
cg25835179	0.00826248	37.6210651	2.54873268	555.313057
cg03599590	0.00826599	23.4768863	2.25657951	244.247626
cg22325530	0.00827093	6.27500369	1.60560028	24.5239565
cg19281068	0.00827147	50135.9766	16.2833331	154367422
cg18881978	0.00827451	0.13869533	0.03200945	0.60095981
cg02083559	0.00827817	4.81956989	1.49979991	15.4875685
cg25552655	0.00827906	1.5759E+19	88640.0906	2.80E+33
cg16513905	0.0082896	17843.3978	12.4504323	25572352.6
cg03726881	0.00829082	12.9293408	1.93342562	86.4620033
cg14444703	0.00829209	1313939.43	37.6682463	4.5833E+10
cg02187348	0.00829372	26910560.2	81.9601968	8.8357E+12
cg11481534	0.00829526	32.7451748	2.45586664	436.606147
cg18532308	0.00829792	14.7306237	1.99904785	108.547315
cg00358155	0.00830401	95745.4275	19.1564971	478541920
cg20265062	0.00830695	25.7180915	2.30673489	286.734394
cg10891888	0.00830755	1147542.97	36.2871337	3.629E+10
cg11255208	0.00830882	32.8657885	2.45685688	439.65119
cg23814129	0.00831519	3695044282	289.6113	4.7144E+16
cg01678172	0.00831573	132.736976	3.51760999	5008.82839
cg25958098	0.00832033	12.6201807	1.91975994	82.9629567
cg23956317	0.0083259	0.02648215	0.00178442	0.3930144
cg26669421	0.00832675	268646.305	24.9027216	2898110438
cg10197666	0.00833502	78223.9573	18.1124938	337832414

cg01943874	0.01497745	4.9472449	1.36453311	17.936708
cg24334259	0.01500191	42.5050813	2.07133269	872.231653
cg02767164	0.01502132	14296.4739	6.40202969	31925682.2
cg02259384	0.01502212	10.3680206	1.57430231	68.2815808
cg09938049	0.01502847	20.8419154	1.80244933	240.997307
cg10565216	0.01503451	149.267211	2.64013646	8439.223
cg00656881	0.01503747	34.1219659	1.98283144	587.194924
cg04769618	0.01504473	19.0704007	1.77097717	205.355658
cg01040523	0.01504749	7.66821033	1.4841881	39.6185966
cg03969696	0.01505918	9.34653879	1.54191425	56.655412
cg10522115	0.01507374	20.8802731	1.80111349	242.064594
cg01466825	0.01507401	319.912938	3.05523659	33497.9911
cg05580277	0.01507425	0.06491846	0.00715638	0.58890219
cg11282402	0.01508126	1.1981E+10	89.3097586	1.6073E+18
cg22334879	0.01508199	36.0858592	2.00191361	650.472244
cg00009167	0.01508223	30.735639	1.9406717	486.779658
cg25905648	0.01508805	123937313	36.8270475	4.171E+14
cg26942121	0.01509651	8.49372052	1.51263084	47.6939159
cg20501434	0.01512627	13.3645622	1.65024282	108.23348
cg20656621	0.01512731	572955360	49.2153125	6.6702E+15
cg10976626	0.01512785	0.17248885	0.04178104	0.71210293
cg07685728	0.01512865	20.943765	1.79977579	243.719965
cg15987431	0.01513179	10.8428538	1.58473471	74.187483
cg24876960	0.01513902	4.54458979	1.33958129	15.4177253
cg00501919	0.0151397	43.4983553	2.07203611	913.163098
cg15614730	0.01514276	0.13091716	0.02537956	0.67531921
cg25753915	0.01514362	5980540819	77.2027313	4.6329E+17
cg22670485	0.01516701	9.34E-08	1.98E-13	0.04406293
cg07826859	0.01516973	6.98241586	1.45472275	33.5143802
cg20001829	0.01518633	19.4895724	1.77251233	214.296637

cg05557932	0.00833517	199.234289	3.90045966	10176.8267	cg06312003	0.01518639	1468.53717	4.07723863	528936.765
cg02251172	0.00833869	1.2148E+13	2307.86137	6.39E+22	cg14640066	0.01518989	8.66207017	1.51595207	49.494612
cg21197375	0.00834111	236517.037	24.0511712	2325887097	cg20465207	0.01519449	10725.1038	5.97772301	19242753.6
cg09171882	0.0083442	15.7567461	2.0310394	122.240389	cg22312127	0.01519779	8.5852E+13	483.332184	1.52E+25
cg23641145	0.00834761	95.1849009	3.22377225	2810.42352	cg07493874	0.0152191	14.8532158	1.6809136	131.248874
cg19280776	0.00835992	57.1703771	2.82638994	1156.4052	cg09131151	0.01522363	0.08755591	0.01224943	0.62582808
cg02378269	0.00836003	9.46841382	1.78115439	50.3330091	cg09461851	0.01522604	0.13424203	0.02652089	0.67949913
cg27523779	0.00836184	8.32555085	1.72320516	40.2243439	cg14975318	0.01522921	1874521.13	16.1015493	2.1823E+11
cg03048083	0.00836241	3.34625191	1.36360599	8.21161095	cg07270285	0.01522938	0.07553766	0.00937908	0.60836845
cg14528930	0.00836649	63587941.7	100.757934	4.013E+13	cg20693580	0.01523148	9.39929311	1.53886428	57.4103329
cg20187572	0.00836754	545.34275	5.0410817	58995.0199	cg19297231	0.01523183	22862.7546	6.89573337	75801299.2
cg00929411	0.00836864	94.679627	3.21584564	2787.51929	cg08101193	0.01523684	0.0921535	0.01343331	0.63217993
cg24293588	0.00837388	336714.181	26.2113617	4325469277	cg20066737	0.01524098	0.03795907	0.0027029	0.53309073
cg11844042	0.00837538	0.06014085	0.00744091	0.48608585	cg24507955	0.01524309	32.6475536	1.95474222	545.270238
cg26981201	0.00837805	117.685279	3.39876776	4074.95474	cg10867327	0.0152469	2164.48082	4.37758964	1070218.45
cg16908156	0.00838009	5240.76194	9.00043535	3051584.13	cg05587419	0.0152488	94.5685977	2.39781406	3729.73861
cg14560703	0.00838016	19.6414702	2.14674844	179.70776	cg11786558	0.01526703	84778.6635	8.84498544	812598487
cg25044834	0.00838866	3737808.02	48.4903808	2.8812E+11	cg14684375	0.01526799	2.61E-05	5.19E-09	0.13174945
cg00431235	0.00839078	1.996E+10	437.923025	9.0978E+17	cg16260298	0.01526922	145.950137	2.60431997	8179.27239
cg04195127	0.00839081	95.6984486	3.22097239	2843.30071	cg20311863	0.01527139	6.76842952	1.44378834	31.7301621
cg17191919	0.00839283	20.4907257	2.16924167	193.556045	cg07307426	0.01527396	5417.66792	5.21243071	5630986.26
cg07139165	0.00839548	0.21731649	0.06984692	0.67614229	cg13048591	0.01527738	70.3010928	2.26276849	2184.15789
cg26149678	0.00839669	14.0176087	1.96778822	99.8549298	cg27438798	0.01528085	4.08701975	1.31033113	12.747717
cg10060338	0.00839811	3370.92664	8.02359226	1416216.83	cg08937573	0.01528269	42.9963786	2.05859509	898.034088
cg03045231	0.0083987	0.09706343	0.01713095	0.54995839	cg20601919	0.01528316	5.3568752	1.38015194	20.7919949
cg02149965	0.00840229	22.0350295	2.20930008	219.772103	cg02335251	0.0152838	7.57137016	1.47491841	38.8669948
cg13707337	0.00840372	16.6746311	2.05686852	135.177976	cg16016641	0.01528569	130.042425	2.54561143	6643.2104
cg21534623	0.0084055	3.0633E+11	878.245337	1.07E+20	cg06785999	0.01528921	6.54670869	1.43420212	29.8837897
cg18815943	0.00840775	5.2448702	1.52908337	17.9902966	cg05635953	0.01529594	34985769	28.016587	4.3689E+13
cg04750166	0.00840836	1.0076E+14	3874.6142	2.62E+24	cg19893178	0.01531404	9.94191278	1.55323677	63.6359065

cg07352215	0.00840982	5138.17317	8.92899665	2956751.42
cg04677344	0.00841179	5612.23841	9.13142763	3449320.44
cg02258703	0.00841207	12.6725828	1.91669754	83.7870088
cg07638098	0.00841596	4.3563E+10	531.209948	3.5725E+18
cg19380361	0.00841995	32886.6832	14.3500407	75368004.5
cg14546261	0.00843401	251921951	141.352302	4.4898E+14
cg00886812	0.00843445	3041.18737	7.78873704	1187460.89
cg18098750	0.00843723	11.4509112	1.8662558	70.2601259
cg13240116	0.00843734	24.3906093	2.26468012	262.686909
cg01047631	0.00843781	6.52037165	1.61576313	26.3127966
cg10985987	0.00844003	2835.8507	7.64676247	1051693.34
cg09275980	0.00844119	0.09841381	0.017529	0.5525289
cg09932441	0.00844221	7185.29468	9.69833162	5323437.23
cg04200399	0.00844377	62389.8027	16.8568679	230914040
cg19318511	0.00845019	2.18E-06	1.33E-10	0.0356229
cg06974755	0.00845048	12731.8343	11.2170355	14451198.3
cg15811902	0.0084506	816.052608	5.55526873	119875.724
cg13099839	0.0084513	36.9575976	2.51736163	542.577596
cg20806502	0.00845625	6.1735E+13	3358.92701	1.13E+24
cg25495844	0.00847199	9183.66554	10.2949512	8192337.31
cg15821939	0.00847845	26.6367123	2.31276992	306.781249
cg07114009	0.00848767	4.24368751	1.44640062	12.4508269
cg05230647	0.00849193	1852.41778	6.8270011	502629.425
cg10519271	0.00849377	6239.22685	9.30657891	4182842.27
cg20320575	0.0084971	30.6134309	2.39472478	391.352759
cg12386787	0.0084998	731342588	182.875582	2.9247E+15
cg07139985	0.00849994	1.9311E+10	421.655864	8.844E+17
cg17928876	0.00851632	10.5147481	1.82211536	60.6766896
cg06994787	0.00851958	10.3847786	1.81618861	59.3790903
cg24243914	0.00852079	13.4323557	1.93928123	93.0386874

cg07309576	0.01531601	0.13489395	0.02671601	0.68110378
cg26149131	0.01532159	0.00205631	1.38E-05	0.30550509
cg01763057	0.01532766	24756.1479	6.94858186	88200279.3
cg22168087	0.01532897	205.279532	2.77368106	15192.6936
cg12111295	0.01533632	0.05272423	0.00488445	0.56912183
cg25274503	0.01535439	671.240736	3.47597208	129622.481
cg09663494	0.01535535	3800.21096	4.84354099	2981620.97
cg22802813	0.0153573	8.19814129	1.49578079	44.9327342
cg18346784	0.01535869	607974.356	12.7897912	2.8901E+10
cg18568145	0.0153591	167.351123	2.66396861	10513.0363
cg16656078	0.01537416	291.050892	2.95957698	28622.5437
cg12210363	0.01537559	25731184.4	26.1308603	2.5338E+13
cg13038216	0.01540323	18.0229312	1.73734729	186.966678
cg23912429	0.01540486	30.4907343	1.92081453	484.005542
cg07418892	0.01540506	11.9884592	1.60712221	89.4288888
cg19116747	0.01542312	4355457280	69.1402353	2.7437E+17
cg22586726	0.01544504	5.883651	1.40205183	24.6904916
cg13463731	0.01544631	14.6938157	1.6693576	129.336112
cg25512791	0.01545191	55.9287767	2.15358705	1452.47348
cg00237825	0.01545234	0.1062587	0.01731154	0.65221864
cg02803139	0.01546091	77.34173	2.29016198	2611.93018
cg06761203	0.01547655	33.8175809	1.95530155	584.886143
cg04321497	0.01548899	22338609.2	25.0527086	1.9919E+13
cg01409498	0.01549007	11.9765802	1.60415336	89.4169328
cg00590620	0.01549228	14.9239371	1.67267783	133.154092
cg21165793	0.01549675	4881.1881	5.0334652	4733517.82
cg02009255	0.01550609	1396715.1	14.752464	1.3224E+11
cg20508523	0.0155121	12.1460315	1.60775488	91.7590635
cg12179011	0.01551434	9.30297958	1.52819755	56.6323567
cg18676229	0.01551479	5768.10848	5.18957569	6411135.97

cg18953104	0.00852177	5.4166842	1.53839991	19.072068
cg23820816	0.00852342	44.2240718	2.62747551	744.352713
cg24678320	0.00853268	0.02997264	0.00219598	0.40909175
cg11993720	0.0085333	1600338706	221.546016	1.156E+16
cg06008480	0.00853338	106135.895	19.0858239	590219646
cg16452651	0.00854366	0.06233945	0.0078797	0.49319221
cg10311104	0.00854567	715832.542	30.983877	1.6538E+10
cg11349878	0.00854643	0.00083806	4.27E-06	0.16459946
cg03377767	0.00854658	20.4792065	2.1575405	194.387035
cg04621020	0.00854869	16.236057	2.03353059	129.631464
cg06526137	0.00855072	236925.856	23.3630788	2402673973
cg19619653	0.00855103	36888.2135	14.5493663	93525742.9
cg19977011	0.0085521	6316824.53	53.8884169	7.4046E+11
cg19782271	0.00855338	15.4126511	2.00646651	118.392115
cg08816023	0.00855619	17.081558	2.0594954	141.675298
cg27586487	0.00855951	5.84065836	1.5670657	21.7688959
cg10690887	0.00856832	3003.69495	7.6704561	1176225.15
cg12738981	0.00856891	9.82671072	1.7885132	53.9913507
cg23034373	0.00856892	14.6981741	1.98143255	109.030369
cg01535453	0.00857155	34.9158552	2.46908341	493.75284
cg07229186	0.00857619	4.6131273	1.47530482	14.4247773
cg19706516	0.00858102	8.47847893	1.72210503	41.7422885
cg17188046	0.00858315	7.27685433	1.6563831	31.9688174
cg23275064	0.00858946	0.13397238	0.02991829	0.59992063
cg10310081	0.00858999	33218755.8	81.6271597	1.3519E+13
cg22583967	0.00859196	392444.473	26.4082374	5831993326
cg07315894	0.00859833	14.2536137	1.9642832	103.429843
cg11685223	0.00859888	12.1585711	1.8864889	78.3629592
cg23284178	0.00860066	213581.09	22.5977308	2018648799
cg26622291	0.00860159	12.3774103	1.89491403	80.848145

cg07182669	0.01551949	5.99485147	1.40559426	25.5680072
cg24848890	0.01552458	10.2502289	1.55634424	67.5089671
cg04022379	0.01552465	6.47747789	1.42632965	29.4165657
cg23295623	0.01553659	0.13887066	0.02806083	0.68725903
cg03093398	0.01555991	4.1371E+13	383.968491	4.46E+24
cg26455579	0.01556887	6547019.99	19.639515	2.1825E+12
cg20375554	0.01556981	171.006053	2.65232951	11025.4288
cg02251393	0.01557155	18.27702	1.73532378	192.499789
cg02011054	0.01557418	700232.765	12.8442471	3.8175E+10
cg13255056	0.01558111	8731455.31	20.7104061	3.6812E+12
cg01920373	0.01558467	1251127.67	14.3224685	1.0929E+11
cg13875008	0.01558873	0.07320727	0.00879726	0.6092018
cg13098382	0.01559854	1214.86754	3.84128654	384221.045
cg13672975	0.01560768	2492389723	60.2366986	1.0313E+17
cg27582235	0.01561342	25.1621046	1.84187355	343.743199
cg03404102	0.01561462	3248.75574	4.6236167	2282718.17
cg06623120	0.01561943	1769.04915	4.11976757	759638.699
cg27314482	0.01562653	29.8679914	1.90199331	469.032621
cg17031739	0.01563364	112.868356	2.44552375	5209.21779
cg19431272	0.01564414	18.4037489	1.73473101	195.245242
cg04337056	0.01564417	0.10810558	0.01780019	0.65655575
cg06933384	0.01564595	16.5400938	1.69998566	160.927653
cg10667044	0.01565294	1803.37579	4.1266815	788082.204
cg13460740	0.01565746	66.4674154	2.21065632	1998.46411
cg20034091	0.01566083	12.4869726	1.61150075	96.7573144
cg12526849	0.0156778	45.1734605	2.05378085	993.602376
cg02016764	0.01569468	8.29304822	1.49071506	46.1353419
cg26508844	0.01569482	1624.18099	4.03607805	653595.856
cg20273422	0.01569518	0.11578196	0.02013738	0.66570036
cg07135540	0.01570324	20.3734308	1.76595868	235.043258

cg22426944	0.00860636	25615464.1	76.1692677	8.6144E+12
cg24820508	0.00860739	5328.81375	8.84160654	3211662.48
cg20876723	0.00860802	7.0627E+13	3291.67946	1.52E+24
cg10737521	0.00861598	43.6780201	2.60888103	731.259656
cg07916003	0.00861667	3.9647E+10	490.623804	3.2039E+18
cg04466840	0.00862138	4.65752463	1.47774902	14.6794452
cg20668838	0.00862447	1387147.29	36.2132757	5.3135E+10
cg03853794	0.00862852	11.1481255	1.84387659	67.4018542
cg01051318	0.00863627	56948.6987	16.0809255	201677091
cg00708105	0.00863802	8.13514497	1.70181398	38.8882595
cg07709681	0.00864154	549.87599	4.95397584	61034.5335
cg25723933	0.00864449	524080.394	28.2031831	9738626284
cg17381426	0.00864596	10.2844123	1.80570607	58.5749462
cg16393012	0.00864706	4.78516256	1.4872551	15.3960009
cg26162295	0.00864851	16.9028536	2.04797692	139.50668
cg20195987	0.00865874	1825375.77	38.6123528	8.6294E+10
cg22226091	0.0086664	49.3309833	2.6848404	906.402448
cg17166812	0.00867046	83.3921221	3.0661404	2268.07814
cg19195724	0.00867171	0.05338582	0.00598629	0.4760954
cg05694648	0.00867528	203.181614	3.84085482	10748.3282
cg14080475	0.00867574	5.60538211	1.54727051	20.3069266
cg22831315	0.00867613	276204.948	23.8691644	3196139237
cg17411681	0.00867705	3252435513	256.21139	4.1288E+16
cg13016775	0.00868329	19.3126898	2.11592504	176.27278
cg09879099	0.00868778	44.1324454	2.60779985	746.864348
cg03607638	0.00869397	1589.50998	6.4564168	391322.627
cg00496961	0.00869919	15.7538998	2.00861322	123.560552
cg03771878	0.00870115	609.853603	5.06409239	73442.8576
cg25934944	0.00870631	4732.5274	8.49915061	2635182.81
cg19946641	0.0087071	0.10867914	0.02070275	0.57051154

cg00374492	0.01570662	47.2088163	2.06915543	1077.09276
cg19843939	0.01570927	6.99617642	1.44331059	33.9126484
cg10626682	0.01571985	0.12433702	0.02290367	0.67498758
cg09490603	0.01572017	6.14828088	1.4083476	26.8409288
cg03746823	0.015721	11.9921148	1.59737036	90.0297279
cg02157015	0.01572614	14.1882421	1.64864998	122.10367
cg27508002	0.01573495	11.820239	1.59260176	87.7294339
cg16881309	0.01573573	4519.43315	4.88291078	4183012.33
cg27405799	0.01574472	36739.1557	7.24148022	186393599
cg08213047	0.01574717	3.3019E+14	542.35918	2.01E+26
cg20587543	0.01575351	4505.84275	4.8744659	4165096.1
cg25789201	0.01575992	32997.2845	7.08772126	153620712
cg27423729	0.01576215	13341871.4	21.9303665	8.1169E+12
cg20331595	0.0157734	5151.81375	4.99238653	5316332.12
cg22156128	0.01577714	31.7413306	1.91623091	525.778009
cg00898147	0.01577946	45.4882925	2.05027649	1009.2223
cg19502671	0.01578226	0.00493852	6.62E-05	0.36835732
cg08589960	0.01578231	872.930511	3.57326159	213252.699
cg16353345	0.0157887	4.4329E+13	367.828312	5.34E+24
cg15923947	0.015791	11.3054282	1.5776275	81.0157704
cg23105863	0.01581011	7.54051723	1.46153762	38.9038225
cg07594531	0.01581027	25.9883069	1.84394749	366.275125
cg15799267	0.01581152	19.8832071	1.75344459	225.465878
cg15642759	0.01581918	10.278206	1.54883871	68.2069206
cg15320854	0.01582151	16.1801056	1.68650169	155.230094
cg19716433	0.01582505	102227.206	8.71787445	1198732742
cg02299189	0.01584173	1581.51998	3.98222992	628091.675
cg07336964	0.01584361	0.06763274	0.00758145	0.60333959
cg12395726	0.01584611	27002.8343	6.77882141	107563397
cg18488946	0.01585442	0.08452093	0.01135319	0.62923188

cg22173752	0.00870833	9.22343132	1.75381998	48.5065094
cg01325552	0.00870924	7.10004551	1.6415091	30.7099401
cg14521552	0.00871475	0.14476374	0.03415837	0.61351118
cg18262197	0.00871952	5.56992288	1.54349154	20.09991
cg02619315	0.00871957	1647.88316	6.50185065	417653.226
cg20376123	0.00872438	127.234228	3.4025069	4757.82979
cg16752778	0.00872491	12034.4946	10.7407222	13484108.3
cg02251393	0.00872664	15.8755051	2.01082405	125.337501
cg05703053	0.00874125	6.06155871	1.57615673	23.311447
cg11314748	0.00874375	0.1186059	0.02409746	0.58376932
cg09076334	0.00874397	6.05877085	1.57588793	23.2939814
cg08970050	0.00874471	270.940436	4.11336659	17846.3841
cg09755939	0.00875047	27.7013811	2.31248044	331.836975
cg02291533	0.00875178	23115.787	12.6287085	42311500.6
cg02953545	0.00875238	11.4891244	1.85177005	71.2831377
cg09162146	0.00875359	18.6405467	2.09224044	166.075549
cg00256281	0.00875573	166.978313	3.63793845	7664.16398
cg12699865	0.00875919	38.3063231	2.50873045	584.907155
cg02675260	0.00876255	4.39E-06	4.32E-10	0.04450861
cg13119284	0.00876808	2.0328E+10	397.81544	1.0387E+18
cg14656297	0.00877012	2007.52339	6.80534346	592203.787
cg19306970	0.00877157	12.2758703	1.88197893	80.0736864
cg20091128	0.00877361	27800155	75.3212855	1.0261E+13
cg08048963	0.00877651	16648.1227	11.5937944	23905891.2
cg17035040	0.0087811	5772.56108	8.87292375	3755522.12
cg05413277	0.00878189	10.7671039	1.82026794	63.6887153
cg25826098	0.00878451	224919770	127.304695	3.9738E+14
cg21679294	0.00878482	122265937	109.169596	1.3693E+14
cg22484599	0.00879263	44537516.1	84.5108752	2.3471E+13
cg02249390	0.00879351	42.5149604	2.57191166	702.793134

cg14328457	0.01585746	5.11452218	1.35794133	19.2632308
cg19559248	0.01587145	992.225702	3.64296945	270249.821
cg14226131	0.01587515	359.587311	3.01155868	42935.5851
cg01051318	0.01587775	217484.412	9.99491506	4732353343
cg18773260	0.01588299	27.9359162	1.86564506	418.308623
cg20569452	0.01589955	8.58984318	1.49551823	49.3376842
cg08276690	0.01593396	1.2691E+10	77.2932493	2.0837E+18
cg20460554	0.01594447	48.6407157	2.06599856	1145.16983
cg00897404	0.01595506	97.7632029	2.35288296	4062.09914
cg02737621	0.01597661	69.5530844	2.20640967	2192.53551
cg14780632	0.01598044	4.95461015	1.34782733	18.2131354
cg15645309	0.01598192	6.54148939	1.41948887	30.1454167
cg03787837	0.01598278	7.8251386	1.46770708	41.7200371
cg22940789	0.0159914	211.989836	2.71463442	16554.6013
cg17941202	0.01599594	823.666699	3.4954422	194088.986
cg13976502	0.01599966	29.1335992	1.87466716	452.75589
cg23764900	0.01600357	26.3824517	1.84014707	378.248981
cg07924703	0.01600585	88.5736322	2.30586054	3402.32559
cg17814457	0.01600644	3600088226	60.3314826	2.1482E+17
cg14038647	0.01600801	0.00042402	7.64E-07	0.23531623
cg09534243	0.0160184	1290079.81	13.7396231	1.2113E+11
cg12789279	0.01601925	20.1343047	1.74913116	231.766626
cg13883681	0.0160237	45.3910214	2.03473785	1012.5849
cg13455434	0.01603608	7.65714571	1.46055757	40.1434914
cg09132215	0.01604246	11.550382	1.5764842	84.6258553
cg04400030	0.01604768	3658572.17	16.6264922	8.0505E+11
cg03752087	0.0160513	0.12517125	0.02305959	0.67945013
cg07723431	0.01605355	27657.5562	6.69874661	114191574
cg19901191	0.01605942	11736.9643	5.70933176	24128275.8
cg09883798	0.01606083	0.14903709	0.03164285	0.70196137

cg14181982	0.00879841	116813.898	18.893373	722236667
cg21352006	0.00879982	16.871137	2.03728509	139.713025
cg02379560	0.00880245	7.7643272	1.67549633	35.9802501
cg13851466	0.00880597	5.49822538	1.53593207	19.6821741
cg16131859	0.00881089	51.8344369	2.70153353	994.549511
cg18229914	0.00881786	47696.561	15.0427237	151233379
cg26266934	0.00882666	139.433773	3.46270038	5614.62872
cg25206802	0.00882775	0.00019435	3.24E-07	0.11652985
cg17791651	0.00882973	23.6953908	2.2169174	253.266786
cg17007872	0.00883347	1.0477E+12	1053.84572	1.04E+21
cg05598886	0.00883527	9.12481619	1.74362326	47.752443
cg21754388	0.00883578	14.668508	1.96465565	109.517985
cg27539234	0.0088363	71.7273535	2.92818965	1756.99455
cg09948076	0.00884175	12.459138	1.88532841	82.3358518
cg23609985	0.00885063	7.76505739	1.67370936	36.0254402
cg08216021	0.00885443	11596.0334	10.4985645	12808226.4
cg23095680	0.00885602	501.742568	4.76902884	52787.6037
cg15840079	0.00885605	461.915525	4.67095148	45679.3338
cg27410952	0.00885883	209.96386	3.83098307	11507.4439
cg18817318	0.00885957	109.304119	3.25145611	3674.47382
cg20009641	0.00886433	10.8191473	1.81853264	64.367252
cg01249180	0.00886539	223444.837	22.0442879	2264876742
cg01899600	0.00886791	0.12315247	0.02566085	0.59103773
cg18948488	0.00887157	14.7846243	1.96646325	111.156471
cg02273078	0.00887161	35609066.1	78.6771016	1.6117E+13
cg18793200	0.00887678	3838.64303	7.93628933	1856683.86
cg02461665	0.00888211	18.9487673	2.09215533	171.62004
cg19209269	0.00888303	0.02066401	0.00113029	0.37778079
cg02308209	0.00889068	1.7823E+11	664.108814	4.7831E+19
cg01780984	0.00889586	31.5861655	2.37713761	419.700504
cg03111114	0.0160647	41.5189607	1.9988867	862.392097
cg07187607	0.01606477	25.6993232	1.82837605	361.225041
cg11906444	0.01606845	17.2160105	1.69703489	174.652283
cg06439941	0.01607571	0.03639334	0.00245126	0.54032453
cg02716556	0.01608291	567578.93	11.7141934	2.75E+10
cg18934106	0.01608326	37.6706596	1.96205692	723.260667
cg04436646	0.01608328	1007466074	47.0079272	2.1592E+16
cg02994288	0.01608572	1162457.44	13.3785636	1.0101E+11
cg22566736	0.0160887	87.8343795	2.29570595	3360.56899
cg24406391	0.0160942	135.599812	2.48797493	7390.47202
cg10316490	0.01609865	5.61737813	1.37759801	22.9057656
cg09767313	0.01609953	0.01755284	0.00065246	0.47221677
cg19574915	0.01610553	70.4451278	2.2023481	2253.28413
cg05508408	0.01611157	10407252	20.0358125	5.4059E+12
cg16576694	0.01611849	0.15967476	0.03582946	0.71159406
cg18191162	0.01612209	57.6301819	2.12070959	1566.09744
cg15496079	0.0161246	525669958	41.3936188	6.6756E+15
cg13915028	0.01613201	16451.6022	6.04644606	44762694.1
cg22235012	0.01614753	287786.274	10.2617793	8070816700
cg15353612	0.01615383	66.7745282	2.17719678	2047.9718
cg27090024	0.01615857	295088.911	10.2985439	8455318217
cg05922057	0.01615893	584.678853	3.25307689	105084.931
cg18261462	0.01615956	6.7471232	1.42399075	31.969078
cg09371439	0.01617285	16.2330332	1.67485133	157.334184
cg18714412	0.01617479	101.851143	2.35250542	4409.62017
cg10864791	0.01617619	1107674162	47.1384763	2.6028E+16
cg25355924	0.01618869	105932328	30.4792217	3.6817E+14
cg02637304	0.01619605	36.056764	1.94014145	670.100745
cg18120358	0.01620408	0.15459091	0.03374506	0.70820297
cg25143652	0.01620502	57.39712	2.11367226	1558.62829

cg22864519	0.00889588	7.40204624	1.65204549	33.165121
cg18607468	0.00889656	125.544145	3.35996533	4690.92113
cg22920498	0.00889916	162971.983	20.2741003	1310039250
cg01577760	0.00890102	7.04328614	1.6314094	30.4079894
cg17808849	0.00890967	90.9183326	3.09675692	2669.2903
cg15056189	0.00891987	119.450087	3.31423719	4305.16056
cg03531211	0.0089199	10.3303805	1.79497931	59.45292
cg21361094	0.00892004	4.80614536	1.48185178	15.5879512
cg19223541	0.00892014	73293.2208	16.549994	324585991
cg05171100	0.0089336	10905901	57.8146965	2.0572E+12
cg01141252	0.00893617	1.0207E+12	1014.76701	1.03E+21
cg08846852	0.00894166	6.62774603	1.6053697	27.3625555
cg04519918	0.0089436	25245.7459	12.6387911	50427899.4
cg02249490	0.00894394	18.4262391	2.07342062	163.751766
cg11417025	0.0089459	6.82095702	1.61681291	28.7760286
cg02464093	0.0089512	666.527591	5.08700944	87332.0631
cg05585263	0.00895363	0.09689223	0.01683298	0.55772084
cg02962602	0.00895444	13.1527965	1.90509271	90.8071584
cg00071950	0.00895609	33.5234465	2.40729871	466.839226
cg14744703	0.00895656	26456.561	12.7693687	54814739.5
cg03326188	0.00896246	23.5057105	2.2022722	250.88562
cg05596265	0.00896588	17.617425	2.04884741	151.48696
cg12606911	0.00896792	5.87639955	1.55695277	22.1792674
cg01774894	0.00897146	3.64834065	1.38197565	9.63142112
cg05309179	0.0089728	8.83450261	1.72382092	45.2764178
cg19082003	0.00898336	5359646404	269.532939	1.0658E+17
cg19183317	0.00899435	13.1809576	1.90396915	91.25024
cg14058329	0.00900179	12.2422666	1.86878635	80.1980878
cg13730743	0.00900681	14.5407486	1.95051501	108.39874
cg25137841	0.00900939	38.8861443	2.49300921	606.548991

cg13994177	0.01620827	0.0923249	0.01323787	0.64390187
cg22800400	0.01620907	16.8795429	1.68567809	169.023356
cg06522557	0.01621773	688.036197	3.34276957	141617.243
cg23732024	0.01621807	5.05175301	1.34872633	18.92171
cg11354857	0.01623122	0.13832289	0.02756616	0.69408369
cg20707345	0.01624505	7007066.21	18.3206224	2.68E+12
cg02025938	0.01624769	5014950.99	17.2188586	1.4606E+12
cg13016775	0.01625138	25.0137975	1.81085676	345.521566
cg25392584	0.01625543	33537009.2	24.4228035	4.6052E+13
cg13643339	0.01627233	21219.9942	6.27111756	71803494.2
cg13851767	0.01628073	13.6889478	1.61934982	115.717611
cg15971010	0.01628778	5.74364794	1.37979898	23.9089114
cg07000955	0.01632318	92.0869544	2.29724153	3691.38684
cg11540692	0.01632522	17.6168251	1.69473095	183.127903
cg27558095	0.01632884	6.04606429	1.39211594	26.2585122
cg21159993	0.01633217	30.6478179	1.87603105	500.678677
cg21887246	0.01633426	16149167.9	21.131792	1.2341E+13
cg00865356	0.01633894	3274.67878	4.42589287	2422905.71
cg02779075	0.01633992	62.8745183	2.14043296	1846.91841
cg08183125	0.01634655	25.4262723	1.812069	356.771913
cg26834010	0.01635018	600719.764	11.520789	3.1323E+10
cg06323837	0.01635454	3053.41764	4.36519388	2135840.82
cg13386351	0.0163554	8.82503502	1.4916975	52.20981
cg00086730	0.01635895	660204.306	11.7118712	3.7216E+10
cg01421867	0.01636136	923294.786	12.4527078	6.8457E+10
cg00519002	0.01636868	5.89436819	1.38488257	25.0877419
cg14044905	0.01637432	9.42E-10	4.02E-17	0.02206339
cg00181849	0.01637531	0.10046578	0.01538738	0.6559516
cg24684765	0.01637589	1035.20883	3.57470294	299789.195
cg04712436	0.01637965	10554.7069	5.47222148	20357699.1

cg27571057	0.00901206	0.02621684	0.0017051	0.40309942
cg14400079	0.00901372	3.1884E+17	23285.5333	4.37E+30
cg12080566	0.00901684	15.2745657	1.97404109	118.190224
cg25542733	0.00901859	524907.854	26.720158	1.0312E+10
cg27361964	0.00901894	40.2879257	2.5141711	645.587309
cg03623835	0.00902014	7.03793556	1.62695532	30.4449277
cg13695646	0.00902075	11.8219873	1.851615	75.4797209
cg22706947	0.00902671	1.0968E+10	318.828756	3.773E+17
cg05651282	0.00902693	15.1238523	1.96857636	116.191026
cg10052561	0.0090275	6.41749943	1.58969566	25.9070336
cg11228744	0.00903032	0.12415926	0.02593246	0.59444883
cg04039397	0.00903289	5.89131892	1.55598256	22.3059304
cg03776080	0.00903423	16.4129273	2.00869293	134.10919
cg05351940	0.00903673	70336.4478	16.1488436	306351094
cg26220419	0.00903877	2682771.14	40.0089498	1.7989E+11
cg05292799	0.0090395	1.4329E+11	602.989707	3.4051E+19
cg02568374	0.00904734	52813031.5	83.9406906	3.3228E+13
cg03975690	0.00904843	235.24653	3.89754543	14198.9184
cg19245758	0.00905022	14.003131	1.92985203	101.607623
cg24368491	0.00905171	0.09180741	0.01527866	0.55165832
cg06352088	0.00905307	127.983117	3.34830785	4891.92724
cg01415527	0.00905514	1.56E-06	6.76E-11	0.03576884
cg15890111	0.00905537	645556.986	27.985062	1.4892E+10
cg11297817	0.0090572	3.74E-15	5.47E-26	0.00025539
cg10858568	0.00906205	2491.30462	7.00828039	885609.357
cg21087137	0.00906227	4.96767299	1.49045952	16.5571588
cg14364729	0.00906432	135590149	105.800411	1.7377E+14
cg06155802	0.00906723	1166293.28	32.3680662	4.2024E+10
cg26644853	0.00906766	11.7948095	1.84822367	75.2709394
cg08638929	0.00906901	7.0471376	1.62579711	30.5463381

cg04876124	0.01638347	0.13738961	0.0271668	0.69481518
cg26644853	0.01638901	14.3738516	1.63039341	126.72255
cg17611742	0.01639415	13.1128845	1.60300883	107.265623
cg09537434	0.01639693	16446.5673	5.92858246	45624662.9
cg21361094	0.01639921	4.78073875	1.33218008	17.1564366
cg05802477	0.01641297	5.3183E+10	92.277273	3.0652E+19
cg25365404	0.01641625	23916.3673	6.34048076	90212816.2
cg03123289	0.01642268	17.5430078	1.68983223	182.122887
cg08700651	0.01642668	16.7929541	1.67622006	168.237641
cg17369196	0.01643647	34.0343915	1.90718632	607.355343
cg02277383	0.01645294	125.236321	2.41932455	6482.85741
cg21535580	0.01645925	19.7712117	1.72581555	226.502081
cg01492656	0.0164637	63.616597	2.13669416	1894.08081
cg20999932	0.01646573	6.25332609	1.39810187	27.969412
cg10737521	0.01647622	52.7594572	2.06405202	1348.5902
cg05198244	0.01649091	0.11574042	0.01986085	0.67448494
cg20919942	0.01650005	10.7508769	1.54273877	74.9195891
cg17007872	0.01650796	4.878E+11	135.836085	1.75E+21
cg10538030	0.01651083	3403587856	54.8654921	2.1114E+17
cg10684940	0.01651145	355114.224	10.2980137	1.2246E+10
cg01698298	0.01651417	5.48646592	1.36420795	22.0650439
cg03871329	0.01651521	28582509.4	22.9230481	3.5639E+13
cg24153071	0.01651924	5163149.46	16.7680684	1.5898E+12
cg21819468	0.01656424	6.71811691	1.41453733	31.9066126
cg25913761	0.0165752	480.241087	3.0759777	74978.275
cg03075736	0.01657657	84655.0371	7.883224	909079242
cg09175928	0.01658243	5.9619E+14	487.617192	7.29E+26
cg08686462	0.01658843	91.6056454	2.27427408	3689.79022
cg17891715	0.01659366	3076368.42	15.1292012	6.2555E+11
cg14181387	0.01659604	7720.39951	5.09187018	11705830.4

cg24579247	0.00907347	1.3192E+12	1037.76896	1.68E+21
cg26072749	0.00907496	7.09941852	1.62858482	30.9481844
cg16903217	0.00908053	65.2857686	2.82795735	1507.17676
cg23500601	0.00908405	511904.281	26.3061826	9961384259
cg04054731	0.00908905	15.5770075	1.97944923	122.58115
cg00945244	0.00909446	6.9475E+13	2762.7579	1.75E+24
cg27384769	0.00909701	27926.913	12.7421808	61207141.7
cg18800235	0.00910001	6.27552204	1.5785671	24.9480537
cg06178310	0.00910175	8.40875158	1.69759051	41.6514482
cg22923514	0.0091055	35.2243918	2.42320294	512.032137
cg13753515	0.00911095	16.7545201	2.01429882	139.360625
cg14412322	0.00911389	9.04792713	1.72828394	47.367787
cg27195224	0.00911394	0.12635061	0.02668887	0.59816989
cg13717333	0.009116	1.1334E+10	314.316941	4.0871E+17
cg13458803	0.00911798	8.25735519	1.68931485	40.361875
cg10476085	0.0091183	32.7097414	2.37786193	449.953451
cg25001691	0.00912162	64370.4929	15.635691	265006538
cg00915178	0.00912184	17.7530932	2.04278649	154.285492
cg27147000	0.00912291	7.25021369	1.63543979	32.1415676
cg05001531	0.00912943	0.00401892	6.35E-05	0.25423947
cg21577260	0.0091353	1.2671E+15	5599.74448	2.87E+26
cg16270051	0.00913843	0.11447113	0.02243728	0.58401195
cg23343073	0.00914139	89.1000765	3.04639874	2605.96997
cg20501434	0.00914162	11.0929498	1.81670857	67.7343288
cg19695710	0.0091536	0.08856717	0.0143087	0.54820803
cg16489586	0.00915896	20.201698	2.10685055	193.70553
cg02583309	0.00915935	5.39265011	1.51854072	19.1504085
cg17108996	0.00916028	1739.36944	6.35794504	475846.524
cg07647796	0.00916623	43055.257	14.0770329	131686498
cg05172655	0.00917094	0.05444858	0.00609798	0.48616892

cg06090660	0.01660589	6.73489184	1.41433158	32.0708161
cg10418263	0.01660704	16.464562	1.66379783	162.929533
cg06469542	0.0166086	18.413853	1.69792081	199.697171
cg11491247	0.01661105	17.0856565	1.67488887	174.291957
cg25102206	0.0166149	27.9133945	1.83097283	425.542958
cg04329105	0.0166212	42.8387785	1.9788016	927.410276
cg22053945	0.01662946	12.3829043	1.57917075	97.0992648
cg26649219	0.01663391	1.24E-11	1.46E-20	0.01046884
cg13818424	0.01663786	5.94558529	1.3820549	25.5778439
cg04846715	0.01664441	455.497144	3.03679097	68321.3463
cg17405853	0.0166455	0.09758166	0.01452511	0.65556657
cg12409211	0.01664781	732.977382	3.31003423	162311.265
cg26923490	0.01665705	6.27918203	1.39544699	28.2548368
cg19936731	0.01666221	25221306.4	21.9861594	2.8932E+13
cg11094694	0.01666322	2.5306E+12	177.402888	3.61E+22
cg26014634	0.01666409	9.49667289	1.50399863	59.9646797
cg17695512	0.01666865	0.12944143	0.02427223	0.69029843
cg25755283	0.01667461	7.66190507	1.44634766	40.5882978
cg01972009	0.01669173	31.0813577	1.86337784	518.440638
cg01558040	0.01669881	0.00010572	5.86E-08	0.19061615
cg18299211	0.01670204	1808.80574	3.8875962	841594.15
cg09481857	0.01671554	28.7726042	1.83640692	450.805726
cg24032330	0.01671627	0.01404943	0.00042702	0.46224409
cg04718145	0.01671726	10.0135004	1.51712122	66.0924055
cg00226923	0.01672714	33.0667639	1.88263792	580.786599
cg12501949	0.01673002	44.7381196	1.98825866	1006.65944
cg05887405	0.01673567	7.45377625	1.43782057	38.6409692
cg10147797	0.01673898	4.45962456	1.31027177	15.1787223
cg02532538	0.01674626	14.3739701	1.61871378	127.639005
cg16806210	0.01674854	1612.70374	3.79786058	684810.115

cg21753200	0.00917579	462206558	140.160669	1.5242E+15
cg18492126	0.00917885	16.5483947	2.00398467	136.652427
cg04415434	0.0091856	3.6828E+14	4046.5579	3.35E+25
cg25783099	0.00918851	18.5255296	2.06017336	166.585616
cg21647780	0.00919089	1055610.01	30.9943772	3.5952E+10
cg14527250	0.00919248	11.7798263	1.84149821	75.3540285
cg05377387	0.00919561	29.3790918	2.3087577	373.850853
cg08183066	0.00919596	123936.979	18.2256484	842788927
cg11970289	0.00920339	6.65574338	1.59843158	27.713992
cg13646565	0.00920454	41551.2867	13.8924305	124276989
cg04799823	0.00920454	1.0585E+10	302.283073	3.7066E+17
cg04751811	0.00920769	3.65692612	1.37820224	9.70329911
cg09035101	0.00920837	261163.551	21.8819088	3117022433
cg25309564	0.00920885	5.2251E+10	448.213672	6.0912E+18
cg06508783	0.0092166	8.33900989	1.68963246	41.1563387
cg08493879	0.00924813	140617818	102.862561	1.9223E+14
cg24550644	0.00924941	4.21422317	1.42650404	12.4497909
cg16701456	0.00925242	6.71668087	1.60043422	28.1884762
cg13401703	0.00925368	5.47542949	1.52165907	19.7023951
cg08309135	0.00925839	0.06183727	0.00760122	0.50305743
cg01029638	0.00927019	6.69684	1.59868656	28.0528198
cg08473725	0.00927747	20.1521721	2.09752929	193.613526
cg02636234	0.00928593	2.0246E+10	347.616878	1.1791E+18
cg21502466	0.00928872	159.515969	3.49181687	7287.13594
cg09996156	0.00929436	321.472542	4.14885527	24909.1829
cg06422678	0.00930073	6.99423351	1.61489028	30.2926478
cg21789849	0.00931676	13185.5144	10.3390784	16815598.6
cg03991512	0.00931718	14.5386737	1.93303883	109.347536
cg14878065	0.00931893	3714.97177	7.56726971	1823777.37
cg14059420	0.00932734	14.4866225	1.93077228	108.693415

cg19878627	0.01675344	14.765208	1.62635176	134.049332
cg06561044	0.01675518	17199071.9	20.2743076	1.459E+13
cg10029031	0.01675537	2186029653	48.6412288	9.8244E+16
cg19558029	0.01676137	9.36315574	1.49769183	58.5358642
cg18780769	0.01677871	9.43436296	1.49930458	59.3656591
cg13997647	0.01678452	3.3182E+14	416.588497	2.64E+26
cg11141711	0.01678642	1.0275E+14	337.024512	3.13E+25
cg04154281	0.01680205	80.1925691	2.20431521	2917.39045
cg19573564	0.01680234	849873.028	11.7197946	6.1629E+10
cg04102760	0.01680697	7615.6341	5.00776291	11581595.2
cg17322163	0.01680887	128270.767	8.32957179	1975298394
cg10657141	0.01681364	28.0177267	1.82311841	430.577084
cg01505767	0.01682526	11.4695451	1.55177528	84.7741717
cg17062109	0.01682973	5.16597525	1.34405778	19.8557686
cg23600666	0.01684251	48896845.5	24.2036124	9.8783E+13
cg12074985	0.01684352	12.7390767	1.58084451	102.656571
cg06132502	0.0168522	14.5301048	1.61843534	130.449416
cg13561226	0.01685616	232.849233	2.6654667	20341.1903
cg16745967	0.01685899	32475.3683	6.47756973	162815622
cg24619626	0.0168618	0.0695462	0.00781164	0.6191628
cg20577613	0.01686659	4911022685	55.2618851	4.3643E+17
cg11636504	0.01689995	37.2184233	1.91435825	723.590287
cg11934304	0.01690177	9.42857978	1.49604504	59.4220855
cg21844656	0.01690244	0.09199832	0.01298942	0.65158364
cg00221357	0.01690751	2294064.66	13.8567662	3.798E+11
cg25295726	0.01691623	171.36836	2.51664364	11669.1589
cg02160530	0.0169182	10.5625637	1.52642051	73.0910982
cg25196508	0.01692213	18.1874151	1.6825896	196.591057
cg11009335	0.01692517	17089497.6	19.8264777	1.473E+13
cg20733436	0.01693975	7.78788888	1.44472796	41.9810613

cg20377955	0.00933316	7.91621757	1.66372945	37.6662809
cg26222247	0.00934172	9.38518039	1.73453622	50.7810734
cg01851874	0.00934415	11.0122217	1.80398934	67.2226965
cg01421695	0.00934484	226.249958	3.79367089	13493.2747
cg09663203	0.00934638	26213888.7	66.7273991	1.0298E+13
cg09467487	0.00934853	205.978591	3.70632027	11447.2514
cg13572309	0.00935613	21.7938427	2.13291186	222.686924
cg00571021	0.00935766	12.1646601	1.8480273	80.0740095
cg20201566	0.00936047	530980.244	25.5272885	1.1045E+10
cg13159805	0.00936116	4920752138	240.911827	1.0051E+17
cg11717150	0.00936254	1.6489E+10	324.164958	8.3868E+17
cg05342320	0.00936461	3.243E+19	62256.2193	1.69E+34
cg04674060	0.00936934	68.9180417	2.82890404	1678.98819
cg07677850	0.0093703	0.00505432	9.36E-05	0.27282784
cg12084452	0.00937339	123601.427	17.8124708	857675110
cg08084502	0.00937427	15.5648298	1.96249408	123.44696
cg00476675	0.00937792	0.10482914	0.01912098	0.57471676
cg07676709	0.00938552	15.565993	1.96188544	123.503714
cg14539231	0.0093869	9.66990481	1.74542809	53.5725645
cg00374492	0.00938756	49.2499224	2.60280023	931.902046
cg07059157	0.00938876	0.0101621	0.00031855	0.32418473
cg14029891	0.00939296	5.6883E+11	767.117332	4.22E+20
cg25134747	0.00939341	0.01724457	0.00080547	0.36919384
cg00054210	0.00940656	9.33563937	1.72961285	50.3894051
cg21476299	0.0094202	8.29800663	1.67983019	40.9904016
cg22630231	0.00942784	1637541295	181.092843	1.4808E+16
cg26593946	0.0094323	0.10663883	0.01967842	0.5778837
cg19716433	0.00943519	7290.02644	8.83579992	6014677.34
cg00170056	0.00944186	574.96114	4.74055091	69734.577
cg26987660	0.00944861	7.84926822	1.65606572	37.2032408

cg09829176	0.0169539	52.4386835	2.03265873	1352.81712
cg09937438	0.01695633	18.8437405	1.69205606	209.855077
cg02926965	0.01696033	528.666136	3.0740414	90918.7116
cg06897790	0.01696537	7168364.45	16.8856606	3.0431E+12
cg10477603	0.01699137	3224743.83	14.5927827	7.1261E+11
cg25387636	0.01699719	7.47758954	1.43300276	39.0190074
cg04164415	0.01699778	6.65631676	1.40348572	31.5689373
cg27249554	0.01701208	20.263326	1.7120744	239.827417
cg13296579	0.01702115	0.11975458	0.02095274	0.68445275
cg20439022	0.01702247	20.9895282	1.72247653	255.771435
cg19507527	0.01702614	5.76533407	1.36737496	24.3086774
cg26832893	0.0170393	5830.82969	4.70124401	7231825.18
cg12699865	0.01704096	40.833377	1.93891629	859.946708
cg19624799	0.01706682	5043.93845	4.57316425	5563175.46
cg24448952	0.01708402	133388.414	8.18789377	2173021480
cg07453451	0.01708841	16.6874426	1.65105101	168.662713
cg26493224	0.01711031	13.7488369	1.59437131	118.561163
cg01618834	0.01711366	384.139746	2.88353485	51174.4619
cg00986320	0.01711852	7861.28153	4.93271452	12528547.3
cg10089657	0.01712095	127.455692	2.36890391	6857.58232
cg09527192	0.01712145	559.908443	3.08237315	101706.526
cg18117600	0.01713384	6.18278938	1.38253982	27.6497529
cg22219587	0.01713817	3406.51891	4.24574838	2733174.48
cg14884929	0.01713847	8.10994761	1.45076843	45.3354572
cg08378932	0.01714578	10.6426689	1.52238797	74.4004828
cg20712415	0.01716325	13.1664803	1.58053871	109.68172
cg05367052	0.01716445	202255.264	8.75427956	4672822189
cg19038228	0.01716715	4353843.88	15.0937381	1.2559E+12
cg22731763	0.01716816	0.02055601	0.00084221	0.50171646
cg09275980	0.01717162	0.08443899	0.01105741	0.6448114

cg06106312	0.0094526	2101246.86	35.2890897	1.2512E+11
cg11479035	0.00945339	0.14807283	0.03499436	0.62654558
cg25556905	0.00945757	3.47361004	1.35626971	8.89643599
cg07830103	0.00947233	958677.338	29.0381508	3.165E+10
cg00820311	0.00947243	0.12391913	0.02559008	0.6000743
cg15813594	0.00947584	12.6186399	1.85882984	85.661457
cg15929495	0.00947901	26534747.6	65.3367898	1.0776E+13
cg24085895	0.00947902	118.873301	3.21638183	4393.403
cg15742840	0.00948106	0.07320574	0.01015523	0.52771634
cg22053945	0.0094888	9.08899665	1.71499411	48.1691801
cg24315913	0.00949717	254458.557	20.9248585	3094365361
cg25792651	0.00949924	10.5322874	1.77743305	62.4097081
cg05291965	0.00950023	94.786723	3.04005479	2955.38189
cg01205267	0.00950632	9.70562083	1.74199696	54.0753388
cg23033906	0.00953603	5.4422313	1.51167595	19.592745
cg09975039	0.00953848	9.30047497	1.72264955	50.2126707
cg16236960	0.00954071	40.9434222	2.4725103	678.000743
cg03219968	0.0095453	5.34221915	1.50460666	18.967951
cg05110225	0.00954668	247.38252	3.83251005	15968.1542
cg15685783	0.00955231	0.00260367	2.89E-05	0.23447387
cg23514672	0.00955686	0.00088546	4.35E-06	0.18033168
cg14280533	0.00955807	6.82824431	1.59697325	29.1958054
cg03169836	0.00956012	40.8768936	2.46966016	676.579093
cg05531055	0.00956161	8.6497E+10	461.990191	1.6195E+19
cg18098433	0.00956472	0.15405932	0.03743318	0.63404369
cg23005196	0.00956692	12503.6121	9.95294396	15707947
cg10007534	0.00956784	0.0054571	0.00010596	0.28105637
cg06028808	0.00957938	54.8849977	2.65134778	1136.1629
cg27639104	0.00958468	0.01661286	0.0007482	0.36886663
cg23637385	0.00958919	6.70537147	1.58893468	28.2969508

cg15664069	0.01718193	10481.1816	5.16920293	21251858.4
cg26837270	0.0171878	1279668488	41.2727118	3.9676E+16
cg00088688	0.01719434	17.382768	1.65938003	182.092479
cg16249035	0.01719495	0.04391834	0.00335756	0.5744704
cg08749351	0.0171959	280.375335	2.71706227	28932.1041
cg23666180	0.01722899	364.07882	2.84180625	46644.0621
cg17721173	0.01724423	21589.3035	5.84948248	79681925.3
cg09002922	0.01724617	12.4328009	1.56211201	98.9522757
cg17114257	0.0172505	255.691017	2.66704472	24513.2359
cg03368099	0.01725125	13.908296	1.59326844	121.41124
cg18870532	0.01726807	2576.33897	4.00971658	1655359.51
cg10625266	0.01727405	34.8003385	1.87286215	646.637852
cg19265600	0.01728546	72.2764187	2.1303875	2452.08005
cg01101663	0.01728893	29.2353406	1.81538138	470.812992
cg14546261	0.01730446	301501472	31.4039435	2.8946E+15
cg12379940	0.01730715	7.31870756	1.42100388	37.6941127
cg13099644	0.01730984	151441.017	8.21005071	2793451885
cg20748955	0.01731096	19.8310655	1.69425609	232.120257
cg16903510	0.01731275	16277280.6	18.7385212	1.4139E+13
cg00557469	0.01731584	9075.66127	4.99362159	16494567.3
cg12404990	0.01731823	132515.06	8.01300838	2191466714
cg24326232	0.01732024	14.4866683	1.60261552	130.950659
cg09578291	0.01732298	75.2490261	2.14305217	2642.22029
cg01819502	0.01732879	20.7899424	1.70775489	253.093525
cg24527881	0.01733213	73.3469955	2.13277793	2522.4294
cg19060120	0.0173421	596.28389	3.08479234	115260.425
cg12360736	0.01734648	6.83519336	1.40318079	33.2956869
cg15680518	0.01735723	232136867	29.7638459	1.8105E+15
cg19778003	0.01738232	10.5326703	1.51334967	73.3056908
cg24659411	0.01738402	36017.3213	6.33529056	204765262

cg06480736	0.00959119	16.9039931	1.98975481	143.608138
cg13250618	0.00960368	15008255.5	55.6206519	4.0497E+12
cg05135288	0.00960694	17.7773769	2.01333887	156.970659
cg00283472	0.00960833	19.8071176	2.0668794	189.813643
cg00103132	0.00961037	118456633	91.8058312	1.5284E+14
cg03809032	0.00961407	2853.45481	6.91747337	1177048.89
cg19431272	0.00961443	13.9909056	1.89903551	103.07624
cg00935430	0.00961984	6175814826	239.538411	1.5923E+17
cg09838956	0.00962008	0.07867975	0.01148299	0.53910183
cg05845178	0.0096258	7.78925513	1.64664325	36.846169
cg10230885	0.0096318	1822.9242	6.19495005	536413.147
cg05003455	0.00963252	688017481	140.145092	3.3777E+15
cg27321913	0.00963452	18.9766884	2.04381782	176.197066
cg13071386	0.009636	7.39020749	1.62539069	33.6012549
cg11776809	0.00963832	75121953	81.7484105	6.9033E+13
cg01957751	0.00963856	0.04123023	0.0036872	0.46103622
cg06342490	0.00964024	14.6571819	1.91924158	111.936393
cg22815785	0.00964147	2.5059E+10	334.846269	1.8753E+18
cg27002699	0.00964441	50.9966743	2.5973787	1001.26362
cg24959938	0.00964707	0.1011769	0.01785125	0.57344796
cg27455796	0.00965178	624129.244	25.4950973	1.5279E+10
cg27317433	0.00965737	10.9255425	1.78632053	66.8231025
cg05153345	0.00965885	8.25525605	1.66883691	40.8363765
cg00190795	0.00966074	9.10532151	1.70892052	48.5141811
cg22215453	0.00966621	477741.977	23.8467868	9570991639
cg04851348	0.00966866	7.39818145	1.62470705	33.6879739
cg00228891	0.00966922	24.9167193	2.18102435	284.656567
cg13476078	0.00967116	0.01228821	0.00043879	0.34413102
cg20878190	0.00967414	2.7383E+11	593.060084	1.26E+20
cg04154281	0.00968246	53.9657374	2.62914022	1107.70083

cg00984602	0.01738997	902894.088	11.1608459	7.3043E+10
cg18132058	0.01739756	0.05188991	0.00453036	0.59433738
cg08963608	0.01739824	5425003056	51.5055293	5.7141E+17
cg14816013	0.0174086	99.99989	2.24677785	4450.80851
cg23574802	0.01743609	23.2706593	1.73772891	311.627192
cg22122291	0.01743898	47389847.3	22.2598036	1.0089E+14
cg27645955	0.0174423	8.60537395	1.45909231	50.7524167
cg06007678	0.01744801	102.36266	2.2530022	4650.73411
cg25161465	0.01745691	0.09363437	0.01328334	0.66002945
cg27476456	0.01747141	9.77535268	1.4913839	64.0730535
cg08932256	0.01747161	0.14754976	0.03044928	0.71499002
cg15440392	0.01749109	20670861.3	19.1192068	2.2348E+13
cg02311932	0.01749194	17.0115594	1.64281348	176.157035
cg16429070	0.0175017	8.50307793	1.45467685	49.703365
cg06132694	0.0175068	293.530415	2.70388353	31865.3165
cg18997075	0.01751189	25.7868011	1.76614788	376.502512
cg08786829	0.01751843	9.58293803	1.4850391	61.8385746
cg20996682	0.01752823	10.5418885	1.50977423	73.6079683
cg13199010	0.01752832	138.695561	2.36947866	8118.43507
cg13159805	0.01753725	1.2173E+10	57.9835316	2.5555E+18
cg17282836	0.01754117	23.7600919	1.73984476	324.478355
cg03506033	0.0175418	4.28649737	1.28971641	14.246589
cg01550215	0.017547	489938.781	9.87290409	2.4313E+10
cg20705565	0.01755649	832871088	36.1740809	1.9176E+16
cg03642503	0.01755871	35.1064943	1.86187017	661.950528
cg14000147	0.01755955	144574.949	7.96777011	2623308115
cg04740941	0.01758711	391.600255	2.83388265	54113.3061
cg00353006	0.01758865	107.266615	2.26067952	5089.67616
cg26871717	0.01759845	172536.307	8.18959299	3634951982
cg01727686	0.01760374	12.3365212	1.54971401	98.2050582

cg22415969	0.00968276	16.3239992	1.9676562	135.426579
cg04623837	0.00968561	5.51262635	1.51237288	20.0936221
cg06467181	0.00968793	2.5817E+11	582.445567	1.14E+20
cg00756887	0.00968955	13.9076002	1.89236882	102.211229
cg21819468	0.00969175	5.34544073	1.5009667	19.0368891
cg01120874	0.00969315	34.4828468	2.35775058	504.322524
cg07817320	0.00969587	69782.5138	14.9040082	326730847
cg22031999	0.00969761	277.566115	3.90673113	19720.5658
cg21115558	0.00970065	0.19181548	0.05488315	0.67039109
cg03709297	0.00970215	0.11114094	0.0210283	0.5874136
cg17106536	0.00970231	13418716.9	53.2195816	3.3834E+12
cg04037038	0.00970546	3.88869449	1.38934332	10.8842391
cg22974920	0.00970564	1.12E-12	9.83E-22	0.00127767
cg13886554	0.00971113	17.4024256	1.99671711	151.671168
cg21213853	0.00971982	11.3714918	1.80089373	71.8036965
cg06815754	0.00972035	74275512.1	80.2702194	6.8728E+13
cg04983925	0.00972619	501.744394	4.50077623	55934.2264
cg03018496	0.00972892	0.18521254	0.05158003	0.66505747
cg01766718	0.00972941	0.12865995	0.02718292	0.60896267
cg11334692	0.00973591	3.5869E+16	10073.1354	1.28E+29
cg19473656	0.00973596	6.89168985	1.59484526	29.7805625
cg19442545	0.00974452	265.344008	3.85384808	18269.3871
cg13488070	0.00974985	825339.348	26.9072125	2.5316E+10
cg14021375	0.00974991	5.00060866	1.47547552	16.9478156
cg03756121	0.00975053	82070.7738	15.4018903	437323716
cg19628148	0.00975161	5.857848	1.53293848	22.3847099
cg13591673	0.00975397	1.22E-11	6.42E-20	0.00230479
cg02117197	0.00975472	513433.313	23.9751079	1.0995E+10
cg14457918	0.00975473	5828.44933	8.12509232	4180976.68
cg21657704	0.00975478	10.6620929	1.77151776	64.1710896
cg15363487	0.01761784	60576.371	6.8125872	538634826
cg04287755	0.01762255	0.08687098	0.01155083	0.65333567
cg15695155	0.01762758	9.08460683	1.46864197	56.1948269
cg15134649	0.01763646	8.36373565	1.44744545	48.3279519
cg07744449	0.017641	58.2132938	2.02887593	1670.27837
cg08247611	0.01764216	393926573	31.3486726	4.9501E+15
cg19915711	0.01764927	10.0033992	1.49295136	67.0269636
cg22059438	0.01765341	10.2051588	1.49804343	69.5208597
cg16197879	0.01765502	327336.924	9.10937489	1.1763E+10
cg04559178	0.01766758	24.2130013	1.74047415	336.844665
cg27148665	0.01766959	10.7867578	1.51213593	76.946881
cg19077271	0.01766988	111.234826	2.26871363	5453.83353
cg24502391	0.01767232	4626.10548	4.3371614	4934299.17
cg10037049	0.01767235	8.00604962	1.43569109	44.6452799
cg10454568	0.01767316	12.8769154	1.55932408	106.337708
cg21465517	0.01768046	5620378963	49.4812042	6.384E+17
cg25256099	0.01768293	7.80074152	1.42899541	42.5834595
cg10896623	0.01768447	636.759215	3.07064138	132044.823
cg19744936	0.01769493	6.94213989	1.40009328	34.4214966
cg27277974	0.01769907	20363.0089	5.60096512	74032264.3
cg14376467	0.01770083	15.1956557	1.60398149	143.959237
cg15600915	0.0177034	17.4314865	1.64258532	184.986874
cg10884788	0.01770399	11.7729239	1.53436543	90.3316349
cg14071762	0.01770534	663081096	34.003913	1.293E+16
cg14598198	0.01770776	15698.6671	5.35029649	46062521.6
cg25234159	0.01771678	1596.23205	3.59607864	708537.55
cg13437538	0.01772325	117.891979	2.28751873	6075.80546
cg14865516	0.0177277	2451287.79	12.8302877	4.6833E+11
cg21086156	0.01772962	134926213	25.7067182	7.0818E+14
cg14602697	0.01773113	1018.77799	3.32410294	312237.201

cg13576178	0.00976855	128676.316	17.1324038	966448989
cg20031656	0.00977309	4.68610287	1.45195443	15.1241387
cg08545129	0.00977826	7.60175996	1.63166323	35.4158589
cg24994173	0.00977985	5.16480415	1.48628307	17.9475918
cg04738888	0.0097806	4.72418856	1.45461912	15.3428188
cg15198000	0.00978156	18.2177196	2.01469092	164.732618
cg10920410	0.00978218	287704.03	20.7697021	3985305545
cg23994112	0.00978219	6.33426921	1.56126536	25.699005
cg02126424	0.00978229	343744.369	21.6808729	5449973898
cg17147211	0.00978497	8.87318255	1.69346646	46.4924287
cg02506053	0.00978644	3.3754E+10	347.040269	3.283E+18
cg00714713	0.00978831	3075.79151	6.94291554	1362611.04
cg15063443	0.00978869	8.28451414	1.66551054	41.2084901
cg11043450	0.00979477	1071.18167	5.38040234	213261.035
cg13958426	0.00980652	4.67951392	1.4506866	15.0948183
cg22786811	0.00980781	12.6860727	1.84492204	87.232109
cg15114202	0.00980905	2125709195	177.195465	2.5501E+16
cg05734154	0.00981337	10204.5958	9.25051493	11257078.9
cg14634738	0.00982063	531.420466	4.53583915	62261.4035
cg06148736	0.009821	5.40514184	1.50161023	19.456153
cg25996566	0.0098279	253559140	105.734895	6.0805E+14
cg19936731	0.0098281	8440131.56	46.5882388	1.5291E+12
cg20632873	0.00982819	1766.99276	6.05513479	515638.96
cg01730534	0.009831	46050.2227	13.2752734	159742324
cg12766383	0.00983928	32422901.1	64.2972897	1.635E+13
cg05256179	0.00984244	0.07181729	0.00972247	0.53049506
cg12876855	0.00984659	6983.18278	8.41671837	5793806.99
cg01480708	0.00985648	24.9038123	2.1671794	286.178368
cg13460740	0.00986643	53.9845379	2.60944705	1116.83827
cg00283344	0.00987068	0.00310857	3.87E-05	0.24955168

cg19921353	0.01773499	2017.22711	3.7413869	1087619.47
cg01814537	0.01774704	4167183.11	14.0371043	1.2371E+12
cg03105222	0.01775786	9.71309011	1.48265546	63.6318565
cg00651087	0.0177642	775.513648	3.16542078	189997.305
cg25471923	0.01776467	10.6294895	1.50581739	75.0330342
cg20664468	0.01776812	27685138.3	19.4387496	3.943E+13
cg27090985	0.01777856	1.0503E+10	54.261326	2.033E+18
cg05012767	0.01778018	8.6346E+11	116.35527	6.41E+21
cg27523779	0.01778525	8.79809292	1.45683045	53.1334578
cg14058998	0.01778776	2357.82789	3.83256587	1450556.25
cg09622420	0.01778896	1921.00122	3.69884037	997676.38
cg13792279	0.01779252	0.13087734	0.02434965	0.70345488
cg15246590	0.01780164	28.9226615	1.78923212	467.530366
cg00568384	0.01780608	7.94955367	1.43103984	44.1604782
cg06121514	0.01782555	0.09694321	0.01406382	0.66823865
cg21665738	0.01782949	10.9580478	1.51207454	79.4132881
cg06185555	0.01783542	2932.14426	3.96891427	2166201.98
cg25703213	0.01784407	0.06117311	0.00606132	0.61738196
cg05361096	0.01784501	6.10628646	1.36654505	27.2854044
cg02105002	0.01785099	60.1993137	2.02804561	1786.92104
cg10718721	0.01785441	83574.758	7.0665561	988422095
cg25580656	0.0178548	39.5522564	1.88606259	829.442777
cg01385168	0.01786316	74611.0296	6.92465675	803910711
cg16177440	0.01787157	3185.32615	4.01759011	2525469.85
cg02291466	0.01787514	1292833.75	11.3112452	1.4777E+11
cg23227500	0.01788701	184800.443	8.08024825	4226504248
cg09760422	0.01788833	14.2857403	1.58114807	129.072273
cg15949380	0.01789129	7282.21926	4.62730031	11460401.1
cg08460435	0.0178937	13.825358	1.5720894	121.583749
cg01179696	0.01789412	16.7342798	1.62464316	172.367772

cg11580511	0.00987078	70389.2272	14.6363882	338515433	cg08026651	0.01790401	7.70244282	1.42119068	41.7450145
cg17346426	0.00987165	84.9210171	2.90907608	2478.99297	cg07868721	0.01791517	29.8785105	1.79430457	497.532806
cg12566138	0.00987387	40.1461592	2.42938212	663.425519	cg17836177	0.01791646	23.3288712	1.71945035	316.517562
cg17617387	0.00988535	532.29079	4.51866165	62702.9653	cg04195863	0.01791651	0.06782001	0.00730832	0.62935889
cg13520520	0.00988594	22.8537125	2.12083711	246.266991	cg21281951	0.01791946	8.60922458	1.44833838	51.1750216
cg22370296	0.00988799	41252.1061	12.8476792	132454759	cg06306636	0.01792684	191388.242	8.10075304	4521735088
cg12561839	0.00988884	629776925	129.974699	3.0515E+15	cg06051154	0.01793854	7117878	15.0685794	3.3622E+12
cg26056577	0.00988923	47.2020655	2.52426306	882.647701	cg02436004	0.01794181	7.8521748	1.42510768	43.2645546
cg12756303	0.00990052	359740.48	21.5807636	5996692947	cg27113548	0.01794366	0.12520311	0.02240459	0.69966992
cg17980625	0.00990378	6.00949518	1.53809539	23.4797091	cg06329345	0.01794471	523.868876	2.93341891	93555.8839
cg04964562	0.00991489	8433805.11	45.9206586	1.549E+12	cg10676604	0.01795541	42.8005127	1.90670675	960.758064
cg05936800	0.0099152	14.5406855	1.90097664	111.222584	cg01158447	0.01795756	5563.40808	4.39940233	7035389.6
cg13441378	0.00991529	12.4935501	1.83299991	85.1548294	cg23078565	0.01796022	2.0689E+10	59.1414134	7.2374E+18
cg17468224	0.0099201	11399.1443	9.40357283	13818204.4	cg04562491	0.01796083	50.3609753	1.96044309	1293.70133
cg12774845	0.00992245	22250.3103	11.0375794	44853702.8	cg02219997	0.017966	141.391138	2.34034296	8542.10439
cg21787965	0.00992324	4.36127316	1.42373908	13.3596836	cg14188862	0.01796845	62.0775473	2.03169522	1896.75196
cg04755365	0.00992985	25.6000952	2.17627338	301.140876	cg23687994	0.01796875	49883.7839	6.40708292	388381410
cg08276690	0.00993313	4135736320	202.228531	8.4579E+16	cg04916289	0.01798319	18.7315865	1.65338573	212.214442
cg19044630	0.009936	25.4060166	2.17186192	297.19462	cg14155825	0.01798691	691427.198	10.0448943	4.7593E+10
cg06541501	0.00993798	56.4150931	2.62941861	1210.40549	cg22997194	0.01799695	167606.695	7.86994492	3569529947
cg23585979	0.00993811	16.0872665	1.94637661	132.965092	cg12416067	0.01800226	33300906.8	19.4904499	5.6897E+13
cg27576259	0.00994364	20.7283004	2.06795812	207.771343	cg24577137	0.01800596	1799.82815	3.61463507	896184.903
cg09578353	0.00994542	32.0929723	2.29622752	448.54391	cg27359972	0.01800864	27582122.8	18.8559984	4.0346E+13
cg23693200	0.00994583	325.313847	4.00008206	26456.732	cg18558763	0.01801654	4008405.16	13.5358431	1.187E+12
cg25339705	0.00994742	3.0393E+11	564.467363	1.64E+20	cg25355065	0.01802536	3005.76398	3.94240771	2291649.61
cg22806837	0.0099502	6.88818697	1.58786247	29.8811268	cg14673936	0.01802539	18.9138245	1.65465529	216.197755
cg16195332	0.0099566	8.36706203	1.66337897	42.0876591	cg04356968	0.01802715	8641.81393	4.72363055	15810073.9
cg27110054	0.00996125	8.80553345	1.68368092	46.052324	cg21236153	0.01803266	14.1851427	1.57486952	127.768219
cg11685394	0.00996234	8258.81914	8.67002083	7867119.92	cg02131049	0.01803553	2.8278E+12	135.511615	5.90E+22
cg22052291	0.00996623	51.1849841	2.56587752	1021.05521	cg13707337	0.018045	17.9652433	1.63946714	196.862724

cg04122324	0.00997009	10157.0539	9.10373068	11332249.2
cg00168835	0.00997139	13.2981401	1.85789172	95.1834425
cg17371020	0.00997595	28.9986485	2.23874823	375.621342
cg15852352	0.00997939	0.21211372	0.06520678	0.68999316
cg01108370	0.00998641	15.8350097	1.9363689	129.493679
cg19893178	0.00998665	8.79326398	1.68216818	45.9653751
cg01787574	0.00998764	6.89617069	1.58712827	29.9642891
cg21149764	0.00999224	668996.544	24.7335095	1.8095E+10
cg05412137	0.0099949	6.27480114	1.55146837	25.3779774
cg02819335	0.00999635	5.97601193	1.53343107	23.2894189
cg14973383	0.0099997	37901.0844	12.4377622	115494425
cg17213699	0.01000544	5.29253885	1.48930457	18.8080853
cg12631255	0.01000656	2515098.9	33.8770567	1.8673E+11
cg27529930	0.01000808	6.44093673	1.56080297	26.5796944
cg01578996	0.01000907	1.0519E+14	2245.27771	4.93E+24
cg03830181	0.01000911	11.7892276	1.80337485	77.0698829
cg08187418	0.01001002	75.3024036	2.80893485	2018.71965
cg24676409	0.01001682	2.9926E+12	956.76538	9.36E+21
cg15720535	0.0100244	11.266436	1.78325672	71.1802058
cg21684681	0.01002548	40034894.7	65.4214409	2.45E+13
cg08593883	0.01002651	0.12369363	0.02520364	0.60705957
cg27430662	0.01002798	8.14166757	1.65000794	40.1735952
cg27434072	0.01003124	5582896.67	40.8303036	7.6337E+11
cg04765929	0.0100402	16112.1082	10.0967011	25711371
cg11279444	0.01004339	4.28169571	1.41493333	12.9567364
cg04816394	0.01004525	10.3815919	1.74788769	61.6615422
cg05329798	0.01004916	149.119382	3.30057206	6737.19274
cg06445343	0.01004961	20.5647078	2.057289	205.56529
cg00299943	0.01005047	4.33928074	1.41930153	13.2666364
cg15800776	0.01005295	693.450749	4.76141015	100994.018

cg10535858	0.01804625	3541664.84	13.2090583	9.4961E+11
cg24994173	0.01804827	6.01646621	1.35947106	26.6264334
cg23305408	0.01805255	9.24879652	1.4631809	58.4618328
cg26831488	0.01806118	25152.3221	5.65808448	111811570
cg22617213	0.0180649	92.6608292	2.16951657	3957.57717
cg20660171	0.01806582	277.601483	2.61720895	29444.5666
cg05867645	0.01806651	183639616	25.890595	1.3025E+15
cg01522296	0.01806713	52.5230762	1.96867019	1401.28781
cg17621140	0.01806809	16988672.5	17.229727	1.6751E+13
cg07073662	0.01806865	1694078.66	11.6159516	2.4707E+11
cg20461826	0.01807171	111.552981	2.2389466	5558.00105
cg26797270	0.01807915	2.70E-06	6.51E-11	0.11173415
cg22188058	0.01808067	1822.04608	3.60765676	920223.883
cg04466840	0.0180812	4.96216983	1.31488177	18.7264969
cg16704739	0.018093	21.8474363	1.69349165	281.849912
cg10305299	0.01810958	0.15619033	0.03349225	0.72839006
cg18098433	0.01814728	0.14097688	0.02775226	0.71613913
cg17246140	0.01816262	6.6035636	1.37916699	31.6183991
cg13809748	0.0181661	250174905	26.9215275	2.3248E+15
cg25212453	0.01818586	60.5991865	2.01037	1826.65947
cg16353899	0.01818814	232379.522	8.1834893	6598681864
cg25217365	0.01819264	4.99301615	1.31458401	18.964334
cg22663372	0.01819614	16.0466214	1.60324533	160.608021
cg21307628	0.01819816	28.673954	1.76951516	464.644584
cg00042409	0.0182135	21.7400289	1.68757115	280.06455
cg05350938	0.01821809	2484029.51	12.2073544	5.0547E+11
cg25335229	0.01822293	1233.98246	3.35078949	454434.013
cg14262490	0.01822801	4.08E-11	9.68E-20	0.01719755
cg19865616	0.01822884	960.48666	3.21022317	287373.985
cg25198784	0.01823201	3412537.11	12.8648788	9.0521E+11

cg23096553	0.01005532	1356.45192	5.58691495	329334.138
cg18675600	0.01006043	0.06475188	0.00805369	0.52060699
cg01815671	0.01007098	5.50268086	1.50152007	20.1658953
cg02447380	0.01007291	26.0310589	2.17462202	311.60175
cg08325021	0.01007318	1.4471E+12	791.471564	2.65E+21
cg14221831	0.01008005	127.144201	3.17254828	5095.47735
cg08690336	0.0100817	144.214721	3.26895629	6362.24043
cg12821132	0.01008456	122685.822	16.3048246	923150746
cg14138132	0.01008588	483697366	117.174067	1.9967E+15
cg05782110	0.01010293	7.4114E+12	1157.78362	4.74E+22
cg17894889	0.01010391	7500329810	224.194137	2.5092E+17
cg07166266	0.01011298	52.2360704	2.56318982	1064.53569
cg10778862	0.01012445	128.974069	3.17640458	5236.83616
cg14849065	0.01012645	16.4204766	1.94548421	138.593801
cg14238081	0.0101278	1.7988E+15	4241.69032	7.63E+26
cg00442646	0.01013047	26.4165336	2.17808435	320.388532
cg07092593	0.0101549	3668.03293	7.02459994	1915335.49
cg15447669	0.01015539	2148582.33	31.9136966	1.4465E+11
cg00252032	0.01015699	291.929245	3.85033766	22133.8208
cg15353612	0.01015844	48.7083692	2.51639037	942.820821
cg08707078	0.01016574	22.8538137	2.10198657	248.477707
cg22016779	0.01016586	70.2216103	2.74389628	1797.10676
cg08496742	0.01016601	14.9638965	1.90091971	117.794663
cg15311651	0.01016922	9.43808158	1.70377593	52.2823351
cg08756712	0.01016943	24.0380383	2.12709712	271.650635
cg07951301	0.01017368	13.2533546	1.84654914	95.1241445
cg00353006	0.01017378	105.971353	3.02436901	3713.14731
cg25876406	0.01017824	1.7976E+16	7193.98335	4.49E+28
cg06970090	0.0101783	25.9010263	2.1644903	309.94048
cg04943729	0.01018098	77651328.8	74.4702154	8.0968E+13
cg00242147	0.01823304	57.8919418	1.99206522	1682.41325
cg20393324	0.01823386	5.73185092	1.34511291	24.4248008
cg01079738	0.01823612	4092.14452	4.10442547	4079900.32
cg02429905	0.01826189	7.58720501	1.41013883	40.8227039
cg23044079	0.01826309	36.1037626	1.83716215	709.508233
cg19803052	0.01826393	7.48083113	1.40672487	39.7823593
cg23011817	0.01827744	4394298.96	13.3627548	1.4451E+12
cg05496363	0.01827956	8.3114823	1.43173069	48.249813
cg00321850	0.0182851	600.335018	2.95634267	121908.105
cg25098174	0.01828744	29.0500986	1.7696313	476.883648
cg13389876	0.01828752	999763.708	10.3868591	9.623E+10
cg19039291	0.01828901	10622.1474	4.80911818	23461684.7
cg14240820	0.01829194	0.11000149	0.01758612	0.68806109
cg09797971	0.0183077	105.072859	2.19878839	5021.08605
cg23114616	0.01830874	9.53565984	1.46477277	62.0770747
cg24521848	0.01831001	9.13844686	1.45423225	57.4263232
cg27310963	0.01831687	189.044804	2.42779308	14720.3393
cg01904183	0.01831725	39.8303672	1.8653648	850.481444
cg09240001	0.01832846	8.10272091	1.4245313	46.0882019
cg24630566	0.01834155	767.646403	3.07385158	191707.695
cg13239297	0.01834352	34422.6257	5.84528018	202713492
cg06946814	0.0183576	27.3106892	1.74832063	426.622971
cg15718811	0.01836159	29.945502	1.77556081	505.042171
cg27455017	0.01836182	10.3055858	1.48284158	71.6226866
cg11775521	0.01837083	5.71945541	1.34232817	24.3697263
cg22777668	0.01837102	46.2493453	1.91031743	1119.71021
cg09170903	0.01837439	337.214594	2.67118589	42570.4863
cg21545902	0.01837609	7.66183236	1.41014047	41.6296647
cg22715945	0.01837812	596.592809	2.94071859	121032.656
cg03665761	0.01838679	11.8175234	1.51685828	92.067836

cg07554231	0.01018346	117001.092	15.9341808	859112602
cg13983182	0.01018404	15.9874552	1.93003967	132.43185
cg18490616	0.01018636	10.532252	1.74800805	63.4598519
cg19336497	0.01018647	21.0372152	2.05974313	214.86389
cg11974722	0.01018752	179.544217	3.4250431	9411.88913
cg00293996	0.01018761	38.3656926	2.37516241	619.716091
cg10631694	0.01019792	2337915545	166.419969	3.2844E+16
cg03746823	0.01019852	7.4197424	1.60823888	34.231592
cg01805784	0.01019963	303984283	102.566957	9.0094E+14
cg27456945	0.01020341	12.0265769	1.80312264	80.2155924
cg00481884	0.01020582	63.8044915	2.67768002	1520.35087
cg04260516	0.01022185	593739.354	23.3043764	1.5127E+10
cg04769618	0.01022411	17.0307926	1.95695888	148.213588
cg17621140	0.01022478	2800209.89	33.6343863	2.3313E+11
cg11330839	0.01022571	44770.2427	12.6291685	158709944
cg14011140	0.01022578	982093.681	26.2400028	3.6757E+10
cg22945387	0.01022971	249.49493	3.69435236	16849.427
cg06088623	0.01023934	122.910617	3.12275004	4837.72944
cg00813746	0.01024108	5.24281189	1.48007811	18.5713689
cg22967612	0.01024109	45843470.5	65.0186818	3.2323E+13
cg23591609	0.01024204	12.7941874	1.82794647	89.5492477
cg00786685	0.01024256	18.783084	2.0017852	176.244806
cg13982688	0.01024676	2556.27589	6.40051019	1020941.49
cg11751707	0.01024714	5.70913759	1.51006633	21.5846492
cg14423778	0.01024899	7.37579148	1.60434432	33.9093668
cg16066237	0.01025186	900764.322	25.6159741	3.1675E+10
cg24646841	0.01025239	0.18514187	0.05108234	0.67102479
cg18427465	0.0102552	97.9813131	2.9574718	3246.12992
cg10235181	0.01025581	0.11277751	0.0213106	0.59682805
cg14722070	0.01025619	31595.5354	11.5918056	86119271.4

cg00594128	0.01838977	7.92085764	1.41779031	44.2519498
cg08572782	0.01839188	1137199.58	10.5064394	1.2309E+11
cg24341452	0.01839214	14.34147	1.56705368	131.251255
cg16752778	0.01839277	10997.8906	4.80434119	25175896.7
cg13725062	0.01839838	0.00423229	4.50E-05	0.39790319
cg13164831	0.01840406	0.03454497	0.00210464	0.56701147
cg18711066	0.01840419	23.9543503	1.70822033	335.911528
cg00577560	0.01840484	19.6769709	1.65248063	234.304219
cg19217955	0.01840923	11327.3217	4.82303986	26603184
cg14860524	0.01841249	33.8715846	1.81057884	633.656054
cg05022059	0.01841601	10.6930747	1.49075073	76.7008491
cg05703009	0.01841718	0.12748905	0.02299679	0.7067707
cg19019038	0.01842429	58080.7593	6.34548102	531618421
cg27634164	0.01842489	0.08981842	0.01210665	0.66635687
cg16556677	0.01843301	40.9848406	1.86867582	898.902392
cg26929355	0.01843835	115.615817	2.22481125	6008.15789
cg11797365	0.01844817	133.009544	2.27714799	7769.16521
cg02184697	0.0184547	12.5761389	1.53099094	103.305165
cg21139076	0.01845795	11735272.8	15.4564827	8.91E+12
cg00742040	0.01845801	13.1741956	1.54291005	112.488366
cg11913218	0.0184659	132150.877	7.26257219	2404637621
cg04674143	0.01847128	56193.3386	6.28726832	502235810
cg03039008	0.01847704	89229783.8	21.6881348	3.6711E+14
cg01052103	0.01847872	83.9547357	2.10545768	3347.67957
cg17892539	0.01849639	199164.316	7.76063825	5111232272
cg14227687	0.01852271	5.383E+14	295.824688	9.80E+26
cg11209279	0.01852326	3.6705E+10	59.1728877	2.2768E+19
cg12059258	0.01852364	0.15982268	0.0347421	0.73522588
cg05564117	0.01852445	24.9021616	1.71471695	361.644324
cg22290047	0.01852748	10639561.6	15.0828436	7.5052E+12

cg15970621	0.01027032	762.062419	4.79926462	121005.858
cg01447966	0.01027306	2.2562E+14	2466.64029	2.06E+25
cg02899870	0.01028238	8.78673804	1.67095452	46.2051865
cg00252616	0.01029067	0.0060027	0.0001206	0.29878139
cg05184938	0.01029071	8.75488805	1.66921895	45.9185207
cg05250768	0.01029971	4.14736126	1.39904469	12.2945361
cg07806866	0.01030283	2.7218E+11	499.991805	1.48E+20
cg02154252	0.01030399	13.5034033	1.84843659	98.6465543
cg15949380	0.01030814	4778.86217	7.38299371	3093260.61
cg09806575	0.01030842	124097.32	15.921793	967236842
cg06117133	0.01031216	252353.616	18.8159975	3384478942
cg21101743	0.01031294	7.33187541	1.60003121	33.5970928
cg04665046	0.01031303	209.534933	3.52896345	12441.2987
cg27275781	0.01031652	240509761	94.8512964	6.0985E+14
cg23332610	0.01032078	0.06722369	0.00854226	0.52901964
cg13873920	0.01032242	4.0838E+11	547.358147	3.05E+20
cg01410292	0.01032287	13261.1536	9.38041485	18747379.3
cg15516684	0.01032622	1981613.27	30.5355334	1.286E+11
cg21724798	0.01033004	9.15785807	1.68558014	49.7551924
cg20019163	0.01033086	84.207567	2.8437979	2493.46634
cg06378498	0.0103337	119320.291	15.7283572	905201463
cg12529006	0.01033789	12752.1117	9.28102563	17521377.4
cg17924650	0.01034663	77.6182857	2.7877769	2161.07619
cg21857375	0.01035094	3.18E-07	3.43E-12	0.0294767
cg06251289	0.0103574	20.4599159	2.03561494	205.642115
cg14925616	0.01035782	28.140779	2.19425925	360.897849
cg12648523	0.01036116	224.062849	3.5758884	14039.6329
cg16704739	0.01036348	20.0632942	2.02588389	198.696369
cg13818424	0.01036478	5.18301781	1.47305659	18.2366881
cg20664468	0.0103804	19707058.6	52.0096774	7.4672E+12

cg20863668	0.01852951	487809.219	8.99275388	2.6461E+10
cg11139684	0.01853578	2657094.64	11.9414562	5.9123E+11
cg00532413	0.01854879	21.8054528	1.67605464	283.688706
cg05875017	0.01855312	16.205379	1.59460004	164.689766
cg13459498	0.01856073	5.3601462	1.32470204	21.6887771
cg14325123	0.01856856	345.104915	2.66011514	44771.5217
cg21187108	0.01857165	92.5999387	2.13404835	4018.06673
cg19245228	0.01857281	31487.9901	5.66158498	175126492
cg20070464	0.01857289	1984.11261	3.56429109	1104484.1
cg22960945	0.01857548	14.7458068	1.56891586	138.591765
cg15702823	0.01857889	613077.659	9.30068791	4.0413E+10
cg04645049	0.01858367	26485.8905	5.49569008	127645916
cg06980169	0.01859204	9.33710653	1.45301609	60.000408
cg22448889	0.01860738	5.49543752	1.32949573	22.7152543
cg20716058	0.01861817	16.066944	1.59027594	162.328237
cg14893163	0.01863273	16.0612378	1.58972725	162.268942
cg17161421	0.01863601	8.230169	1.42173867	47.6428498
cg26484001	0.0186387	19.4539091	1.64121073	230.59475
cg09268877	0.01864043	2.7356E+16	554.007149	1.35E+30
cg12085570	0.01864416	10.5637585	1.48203655	75.2970596
cg02837212	0.01864521	306.432874	2.59962753	36120.9848
cg23244095	0.01865805	56.456201	1.95957038	1626.53134
cg00295485	0.01866867	10.9581314	1.49051195	80.5633557
cg09684870	0.0186758	1239.01714	3.27713381	468446.99
cg00518698	0.01868352	1.5251E+10	49.7277993	4.6772E+18
cg02000318	0.0186851	9.06786328	1.44382837	56.9500825
cg00731062	0.01870182	0.10339214	0.01559697	0.68538523
cg18490616	0.018704	11.5792503	1.50335664	89.1864473
cg08641278	0.01870548	10.7310204	1.48440191	77.5765634
cg15884014	0.01871348	0.20286664	0.05366583	0.76687294

cg21938523	0.01038952	3881035.37	35.4382547	4.2503E+11
cg16058797	0.01039051	7.48797223	1.60549464	34.923647
cg17903246	0.01039239	365173.894	20.3203587	6562481248
cg04111611	0.01039331	684.264961	4.6412045	100882.979
cg24430419	0.01039336	7.05891362	1.58328339	31.4714737
cg12748367	0.01039747	9104.13967	8.52609269	9721376.73
cg02919799	0.01039775	53.1405603	2.54456516	1109.78457
cg27323088	0.01040086	144495.186	16.3237352	1279049103
cg17345480	0.01040305	111.633363	3.02891934	4114.34122
cg08066497	0.01040442	13275869.5	47.2082075	3.7334E+12
cg00313981	0.01040487	1147.9506	5.23733638	251614.653
cg19622675	0.0104066	94.772321	2.91409946	3082.18472
cg27187881	0.0104108	2496.53917	6.28355315	991908.191
cg11955344	0.01041287	28210832	56.2760076	1.4142E+13
cg06285333	0.01041515	159.908992	3.29385798	7763.20226
cg15387598	0.01042073	433.961988	4.16302285	45237.0824
cg26393255	0.01042129	1509.24984	5.57851317	408322.975
cg27111250	0.01042665	9.42712828	1.69346737	52.4785711
cg11353547	0.01042989	7.34396578	1.59693019	33.7734447
cg03236184	0.01043319	411.086994	4.10734905	41143.9384
cg15658676	0.01043632	41.8986633	2.40283587	730.594214
cg23666491	0.01043692	265.359644	3.70552806	19002.8896
cg19828022	0.01043997	16866241.8	49.6467204	5.7299E+12
cg14071762	0.01044395	904079035	126.272161	6.473E+15
cg26123920	0.01044687	16228.5319	9.72062869	27093437.7
cg21236315	0.010457	10.6661897	1.74204115	65.3070692
cg08464513	0.01045749	10.557063	1.73782543	64.1327823
cg09024228	0.0104607	61.745235	2.62914044	1450.08383
cg12803617	0.01046481	78.4257901	2.78026164	2212.23948
cg08468991	0.01046526	876636968	124.81676	6.157E+15

cg00207534	0.01871885	6252.46973	4.28055327	9132786.16
cg04707408	0.01873095	9.99387727	1.46631573	68.1146501
cg00700324	0.01873139	14450.9562	4.91654987	42474934.6
cg03465285	0.01873349	13249.5606	4.84542481	36230230
cg08535309	0.01873741	2315607.28	11.4281027	4.692E+11
cg14722070	0.01874663	148665.816	7.23471016	3054928854
cg24867524	0.01874843	8.94832741	1.43923125	55.6356481
cg17313042	0.0187579	401.307485	2.70639145	59506.4317
cg24577417	0.01876718	0.11992686	0.02045258	0.70320963
cg15689224	0.01876751	629.811325	2.91547269	136054.2
cg26874634	0.01877027	17.7969193	1.61268983	196.398793
cg21538450	0.0187864	10.568812	1.47865513	75.5414737
cg00023464	0.01880522	0.04215364	0.00300333	0.59165393
cg08199013	0.01880704	452.413278	2.75497972	74293.7501
cg11466815	0.01881751	515363212	27.7505803	9.5709E+15
cg15628562	0.01882192	647.283787	2.92150303	143411.216
cg17868052	0.01882322	124.238787	2.2225739	6944.77527
cg21748136	0.01882599	13.248907	1.53403918	114.425719
cg12291552	0.01883541	3458.85598	3.85322217	3104852.04
cg14301212	0.01883989	18.5722859	1.62182561	212.679959
cg26555401	0.01885408	83.5402221	2.07918309	3356.5917
cg22986077	0.01885635	21.2450352	1.65773425	272.270132
cg25148255	0.01886087	1.1495E+11	67.4443027	1.96E+20
cg13418419	0.01886302	4.6174E+10	57.9807549	3.68E+19
cg19044674	0.01886517	2.72E-11	4.14E-20	0.01792027
cg00134776	0.01887204	10.2916586	1.47008796	72.0489119
cg19160353	0.01887438	832468.719	9.5152434	7.2831E+10
cg19893185	0.01888122	14.1045322	1.54843042	128.477087
cg20410173	0.01889625	366663.129	8.29307886	1.6211E+10
cg19406914	0.01890399	593276.539	8.97240439	3.9229E+10

cg01012089	0.01047464	159.135857	3.28025917	7720.18907
cg20227763	0.01047653	70366.3908	13.6685188	362250587
cg04042828	0.01047886	18.0582906	1.96972707	165.556876
cg25755283	0.01048005	6.62894851	1.5575179	28.2134531
cg19430489	0.01048044	4.75207709	1.44067232	15.6747904
cg14123048	0.01049645	1299.37092	5.3570054	315169.512
cg02774160	0.01049819	8.34912332	1.64338512	42.4172395
cg15219933	0.01049889	288.629099	3.76620385	22119.556
cg10088527	0.01049899	5.3048E+10	323.86584	8.6892E+18
cg06020661	0.01050166	233.496993	3.58335693	15215.0195
cg20351640	0.01050232	189.010677	3.41028606	10475.6714
cg24546680	0.01050239	385199747	102.186981	1.452E+15
cg03880683	0.01050609	1213546.03	26.5271468	5.5516E+10
cg11961288	0.01050768	3746923704	173.829388	8.0766E+16
cg09250423	0.01051224	7.29149377	1.59165722	33.4028463
cg27045999	0.01051525	10.3245718	1.72646464	61.7428123
cg08090734	0.01052341	1.6652E+10	245.530858	1.1294E+18
cg24542751	0.01052367	3.86825804	1.37207675	10.9056729
cg20041105	0.01053171	14.7960064	1.87724597	116.618605
cg26361892	0.01053327	320972736	97.3317411	1.0585E+15
cg25373553	0.01053434	0.14343148	0.03238928	0.63516656
cg23149053	0.01053787	22.7363625	2.07515616	249.110015
cg10522115	0.01054936	14.8110388	1.87680853	116.882925
cg02133520	0.01055347	86222.6368	14.2114834	523122244
cg21534486	0.01055422	9581440.02	42.6928429	2.1503E+12
cg04671541	0.01055966	12.7775569	1.81270209	90.0677291
cg10667810	0.01056093	49.467323	2.48632205	984.191102
cg20546778	0.01056115	18.1881024	1.96837877	168.060677
cg10417218	0.01056261	43.1714331	2.40839445	773.865193
cg02062409	0.01056362	14.8904166	1.87843851	118.036606

cg11521979	0.01890633	13.9476992	1.54485555	125.926538
cg16071118	0.01890874	110.225244	2.17281218	5591.64962
cg21551185	0.01891009	43.143515	1.86115381	1000.11234
cg04383058	0.01891165	17.9984566	1.6110772	201.073196
cg02448276	0.01892141	295.693295	2.5557884	34210.3926
cg14347989	0.018922	85.6212189	2.08324209	3519.03083
cg13606421	0.01892369	18.7771358	1.62197432	217.377565
cg10273340	0.01892979	5.07305333	1.30701696	19.6905403
cg02947424	0.01894825	298802692	24.9047809	3.585E+15
cg01447579	0.01895678	762.661117	2.9831805	194977.132
cg08169325	0.01895881	10.2048302	1.46595888	71.0378444
cg07099627	0.01895926	31328.52	5.49971294	178459526
cg06080729	0.01896127	0.07624396	0.00888098	0.65456094
cg23967249	0.01896742	1.0815E+18	930.271969	1.26E+33
cg25464921	0.01897774	3.86646622	1.24921241	11.9671891
cg20473595	0.01898546	14.1790414	1.54678165	129.976469
cg07666840	0.01900709	79.9237606	2.05435074	3109.40454
cg06709324	0.01901765	114667438	21.0788208	6.2378E+14
cg07562079	0.0190378	975385295	29.8744613	3.1846E+16
cg07619542	0.01904104	18072.43	4.99532211	65383716.6
cg05927190	0.01904587	5.53992146	1.32428552	23.1753117
cg14611892	0.01904921	17696.5729	4.97534887	62944066.7
cg06002947	0.01905391	9176520.02	13.8652896	6.0733E+12
cg22605924	0.01905878	24.1013639	1.68506912	344.719238
cg07447902	0.01906409	1.8726E+10	48.3088289	7.2591E+18
cg10743378	0.01906564	14.0668953	1.54247711	128.285562
cg04946916	0.01907782	1082958.82	9.74345051	1.2037E+11
cg21325316	0.01907835	36590.9674	5.59303742	239386722
cg06840154	0.01908214	26.5851399	1.71147444	412.959521
cg06726474	0.0190825	881082.703	9.4154165	8.2451E+10

cg14581165	0.01056386	8.07414978	1.628342	40.035751
cg14287235	0.01057429	3.88147445	1.37224031	10.9790128
cg08189801	0.01057687	6.72969722	1.56017915	29.0279643
cg20377627	0.01058026	498.415527	4.25861278	58333.0889
cg12906748	0.01058733	7.53469072	1.60151942	35.4485643
cg02408311	0.01058778	0.15831871	0.03852392	0.65062982
cg24311704	0.0105913	16.32146	1.9176471	138.915057
cg09963640	0.01059379	14.5564487	1.86702942	113.490551
cg20813965	0.01059582	166.736648	3.29604466	8434.68847
cg11691181	0.01059782	38215825.9	58.532851	2.4951E+13
cg26552774	0.01059814	0.0763574	0.01061947	0.54903401
cg26570683	0.01059982	51.9848215	2.51149452	1076.02133
cg19751990	0.01060214	0.14292471	0.03214554	0.63546821
cg04704227	0.0106034	3.3465E+10	283.578165	3.9491E+18
cg22776392	0.01061071	7.33924953	1.59100322	33.8557353
cg23987137	0.01061812	7.52185616	1.59990334	35.3635864
cg19439399	0.01062731	9.85568162	1.70348033	57.0211811
cg16500874	0.0106286	0.00228502	2.15E-05	0.24275313
cg02155796	0.01062953	40671101.3	59.0646472	2.8006E+13
cg02432159	0.01063703	2649895075	155.916477	4.5037E+16
cg03262773	0.01063849	121.348735	3.05455984	4820.83054
cg18459239	0.01064081	1571.12494	5.5421172	445395.416
cg16905506	0.01064144	9.50958169	1.68883325	53.5471126
cg12578480	0.01065213	8178.62437	8.12725949	8230313.89
cg13374726	0.01065917	3674245.18	33.6016376	4.0177E+11
cg26861703	0.01065973	5.47609263	1.48486086	20.1955559
cg20666271	0.01066358	1.565E+10	234.273073	1.0455E+18
cg16429975	0.01066445	7.94972073	1.61912168	39.0323102
cg02756939	0.01066493	179.558437	3.34165265	9648.28951
cg13066963	0.0106656	15.2972871	1.88512746	124.133248

cg00727590	0.01908815	12.6522599	1.51530565	105.641843
cg00842549	0.01908815	10.4278897	1.46807523	74.0703751
cg21193251	0.01909889	1.4631E+11	67.2481124	3.18E+20
cg02216011	0.0191053	62.6735914	1.96829742	1995.62272
cg16961545	0.01910891	146.552495	2.26155791	9496.83126
cg21762534	0.01911135	773.481398	2.96872208	201525.591
cg20770175	0.01911716	13.0337958	1.52190597	111.623081
cg03316474	0.01913249	5177.85692	4.04680833	6625023.99
cg26060255	0.01915305	36.4467769	1.79905449	738.369822
cg08830818	0.0191542	118.066967	2.17969495	6395.30259
cg25591377	0.01915435	29.7688412	1.74050775	509.152519
cg09575314	0.01915612	725285.982	9.05714654	5.808E+10
cg04302492	0.01917156	261587.672	7.65750758	8936081296
cg12508343	0.01919147	7.01604632	1.3738983	35.8286389
cg21701379	0.01919192	9.77807453	1.45029652	65.9249608
cg02746725	0.01921353	12.2875997	1.50476802	100.337796
cg09911554	0.01923155	213508664	22.687359	2.0093E+15
cg26085197	0.01923346	329576.725	7.90839016	1.3735E+10
cg23414387	0.01923922	14.563752	1.54626817	137.17082
cg01748892	0.01924559	27.7784041	1.71732501	449.326557
cg21174792	0.01924843	2.513E+11	71.4958764	8.83E+20
cg12570246	0.01925915	0.12178002	0.02088422	0.71012332
cg17758623	0.01925955	1.7924E+10	46.4477137	6.9166E+18
cg26607758	0.01926521	4.46977786	1.27554432	15.6630498
cg27337492	0.01927202	47857.7657	5.76029481	397612590
cg16593646	0.01927379	4995.01502	3.98959929	6253804.76
cg05040724	0.01929983	0.03859299	0.00252594	0.58965027
cg06351970	0.01930489	8.3775E+10	59.2115702	1.19E+20
cg13654525	0.01930885	36.1535517	1.7897233	730.324797
cg03142071	0.0193096	0.05167937	0.00431889	0.61838975

cg07273125	0.01067785	54.4655228	2.53115754	1171.99073
cg06946814	0.01067934	22.8600971	2.06875075	252.608507
cg10098888	0.01068658	49.5119889	2.47487822	990.528354
cg13997645	0.01068711	6.43990724	1.5411247	26.9104798
cg25217313	0.01069668	374626.97	19.6687269	7135457611
cg08561530	0.01069855	0.00010562	9.34E-08	0.11942001
cg00716257	0.01070374	9.14623092	1.67133195	50.0520198
cg15402162	0.01070761	14.1933464	1.85056937	108.858974
cg14489649	0.01071013	135344306	76.9922142	2.3792E+14
cg14581419	0.01071143	10.5870277	1.72873526	64.8365071
cg10622825	0.01071431	16264.1117	9.48000627	27903075.5
cg01394339	0.01071604	68.0715564	2.66152971	1741.00509
cg13879483	0.01071694	4.2230624	1.39669063	12.768938
cg20691205	0.01072059	20.4366307	2.01313619	207.465286
cg23526147	0.01072235	21.4900472	2.03662898	226.758104
cg11189425	0.0107234	199121289	83.9980456	4.7203E+14
cg01052103	0.01073296	99.7883036	2.90621825	3426.34471
cg09745087	0.01073306	36.2834562	2.29875853	572.69573
cg15878616	0.0107338	0.05461063	0.00585068	0.50973922
cg24634593	0.01073685	25.26321	2.11350168	301.977419
cg24716343	0.01074455	122068.868	15.0782242	988233648
cg04311355	0.01075333	1.0895E+14	1780.8291	6.67E+24
cg25185881	0.01075701	67.5215194	2.65204761	1719.10774
cg12456825	0.01076084	11.2746208	1.75210419	72.5510936
cg19427757	0.01076194	1.1664E+10	213.967274	6.3579E+17
cg01692674	0.01076227	8182.9394	8.04966047	8318425.04
cg25972499	0.01076402	163949354	79.6961688	3.3727E+14
cg04165910	0.01076652	72632.0796	13.337495	395532968
cg11277662	0.01076921	0.02905369	0.00191445	0.44091931
cg00558500	0.01077111	0.03039384	0.00207325	0.44557347

cg00402172	0.01931619	7.73371759	1.39342012	42.923442
cg05414908	0.01932229	16.9081876	1.58170474	180.746002
cg17432022	0.01932561	134021.428	6.77968469	2649347871
cg18160072	0.01933565	16.6433176	1.57725031	175.622105
cg24296478	0.01933878	22.6530181	1.65793448	309.517195
cg25804860	0.01934634	46.4019528	1.86181754	1156.47274
cg11625005	0.01935771	0.11531478	0.01886459	0.70489206
cg00973653	0.01935982	6.976933	1.36953721	35.543097
cg09091185	0.01938325	0.0748474	0.00851964	0.65755525
cg24217704	0.01938974	1934326.71	10.3842127	3.6032E+11
cg26168907	0.01939493	7.87710338	1.39600747	44.4472963
cg01243251	0.01940294	1334.18422	3.19877522	556477.843
cg00338116	0.01940584	11.2318757	1.47813743	85.3472949
cg05030680	0.01940657	13.9631599	1.53102354	127.346072
cg24963001	0.01941179	8.58984604	1.4153362	52.1328114
cg00658161	0.01942785	0.00726822	0.00011696	0.45165805
cg23095680	0.01943244	250.785422	2.43892935	25787.2693
cg09769410	0.01943391	7.2062E+10	56.5073522	9.1898E+19
cg04164924	0.01944733	22.3694257	1.65070108	303.138595
cg01963221	0.01945422	1756304.65	10.1581384	3.0366E+11
cg13263104	0.01948293	38733.6137	5.48058804	273746688
cg13460057	0.01949192	26.7290125	1.69707453	420.983344
cg01036779	0.01949581	1128.32839	3.09932827	410774.48
cg16376036	0.01950344	197.761895	2.34113779	16705.4529
cg11992375	0.01950399	12.3887382	1.49917838	102.376632
cg03844154	0.01951587	17.1633768	1.57953712	186.498626
cg07190921	0.01953773	52.7808125	1.89113698	1473.08957
cg12019494	0.0195428	9.83754087	1.44369922	67.0341919
cg22555539	0.01954315	810965.597	8.89376326	7.3947E+10
cg00725244	0.01954351	118.249576	2.15239221	6496.47505

cg25980157	0.01078096	8.6168E+17	14076.1084	5.27E+31
cg25420952	0.01078807	10.3843833	1.71798969	62.768373
cg20361909	0.01078884	6.92042859	1.56408389	30.6200532
cg13002957	0.01078899	18.1688387	1.95517863	168.837104
cg23814388	0.01079073	9.7067E+11	590.871939	1.59E+21
cg18634758	0.01079123	8.51238123	1.64070206	44.1644076
cg09039561	0.01079321	7.73163204	1.60454883	37.2554159
cg09904774	0.01079366	4305.84091	6.92014995	2679171.13
cg10363578	0.0107969	6.0952E+10	311.111739	1.1942E+19
cg01298991	0.01080254	15.5730342	1.88602652	128.587479
cg02595120	0.01080723	9.61781281	1.68708126	54.8297973
cg13195796	0.01080797	2823514435	152.581209	5.2249E+16
cg17878425	0.01081206	809.774264	4.69709722	139604.17
cg20036915	0.01081615	3853.9638	6.73284875	2206055.34
cg26875073	0.01082186	7.38628435	1.58680242	34.381846
cg25123046	0.01082404	4372166.35	34.1367071	5.5998E+11
cg17373751	0.01083354	25.5509706	2.11259316	309.028784
cg04248364	0.01083695	0.01233309	0.00041941	0.36266141
cg12841566	0.01084043	422316.495	19.8593663	8980710661
cg21719787	0.01084683	2.3114E+10	245.777443	2.1737E+18
cg12505694	0.01085343	1007626580	119.160992	8.5205E+15
cg25707005	0.01085699	8627260.5	39.7330627	1.8732E+12
cg07221635	0.0108571	98.4722023	2.88125162	3365.47304
cg18661868	0.0108575	28.9181272	2.17211919	384.996405
cg05488632	0.01085867	17.4173964	1.93243026	156.986622
cg01759112	0.01085903	0.15336386	0.03623862	0.64904446
cg20621021	0.01085911	263.428236	3.61463078	19198.2086
cg11359655	0.01085991	1.654E+10	226.839244	1.206E+18
cg27090985	0.01086334	6702554677	184.058676	2.4408E+17
cg06381959	0.01086603	47.8574222	2.43885423	939.101989

cg13992236	0.01955051	1.0318E+10	40.5363239	2.6263E+18
cg11043450	0.01955188	664.065241	2.83873692	155344.668
cg16601489	0.01955297	0.11796326	0.01961209	0.70952814
cg07881273	0.01955939	5538253893	36.6320574	8.3731E+17
cg10361095	0.01956157	17.7205758	1.58623816	197.964475
cg08095637	0.01956956	11.9335367	1.48850966	95.672404
cg24616138	0.01957685	1715.61329	3.30181343	891428.01
cg09249682	0.0195775	414869.124	7.96166841	2.1618E+10
cg00508024	0.0195806	0.02812832	0.00140275	0.56403468
cg22935653	0.01958191	191.215467	2.3218969	15747.1913
cg01558492	0.01958779	3715.3362	3.73513128	3695646.03
cg03169018	0.0195891	9.89287188	1.44394626	67.7787789
cg04941721	0.0196078	23.9481152	1.66311071	344.843081
cg24292487	0.01961077	148.7906	2.22813917	9935.93356
cg18158177	0.01961821	6.5773E+11	77.9972833	5.55E+21
cg26582085	0.01962971	0.05365867	0.00459796	0.62620213
cg15597770	0.0196478	2760923.89	10.7129857	7.1154E+11
cg23378074	0.0196687	0.13362823	0.02462851	0.72503402
cg09987240	0.01968439	17.714378	1.58230913	198.317245
cg25133212	0.01968572	793842.462	8.74621648	7.2052E+10
cg23048494	0.01970001	15.6709851	1.55119165	158.31685
cg18327128	0.01970275	0.16114859	0.03474698	0.74737045
cg11805463	0.01971678	0.05884276	0.00543911	0.63658787
cg24237081	0.01971974	8.60016455	1.40917442	52.48664
cg11032474	0.01972469	80.871977	2.01394355	3247.49751
cg00437134	0.01972663	8.6839E+11	79.8958275	9.44E+21
cg01445100	0.01973406	79.4769441	2.00780306	3146.01804
cg17030981	0.01973446	0.06400898	0.00634823	0.64540074
cg20265748	0.01974737	10.6293113	1.45691969	77.5487218
cg20945738	0.01975569	20.2211339	1.61372789	253.384887

cg19593490	0.01086723	9.84426374	1.69391682	57.2103232
cg18853219	0.01087429	3789.92195	6.67570286	2151609.9
cg25464815	0.01087662	8327843.56	39.2913239	1.7651E+12
cg05442902	0.01087706	44.1815935	2.39337321	815.590814
cg20569452	0.01088083	7.48042082	1.58961672	35.201376
cg15844609	0.01089195	416.528396	4.00962217	43269.8886
cg22913585	0.01089286	0.00073525	2.85E-06	0.18993612
cg01554060	0.01089724	6.23552672	1.52390419	25.5145918
cg21174792	0.01090578	1.1065E+11	347.612566	3.52E+19
cg23635374	0.01091075	1316.16307	5.21879211	331932.217
cg10043155	0.01092208	61.9503486	2.58254397	1486.07177
cg23644624	0.01092272	9.64465641	1.68388584	55.2409167
cg03890222	0.01093261	9.33206074	1.67082061	52.1225061
cg23818888	0.01093302	10.1782334	1.7044696	60.7792799
cg01557883	0.01093751	3486630.27	31.8662106	3.8149E+11
cg15832300	0.01094069	26.6871818	2.12664775	334.895928
cg11354594	0.01094178	6.99290474	1.56333746	31.2796935
cg07184698	0.01094482	8.6788E+13	1591.58431	4.73E+24
cg02916102	0.0109469	5.35593741	1.47030524	19.510279
cg11418036	0.01094694	8.8326E+11	554.514964	1.41E+21
cg08750890	0.01094961	1.1688E+12	590.940098	2.31E+21
cg05392364	0.01094967	10.3209645	1.70929122	62.3195779
cg11935317	0.01096275	1304374991	123.69097	1.3755E+16
cg21649442	0.01096278	7.91054098	1.60758108	38.9259735
cg07687684	0.01097335	122294229	71.7044368	2.0858E+14
cg25955899	0.01097463	291625.978	17.9375482	4741211562
cg02254152	0.01097791	694.964941	4.48667818	107646.738
cg09940756	0.01098424	22729.1266	9.97933913	51768277.3
cg15431544	0.01098519	10.3955517	1.71074714	63.1697653
cg02399048	0.01098537	26.1437018	2.11364216	323.372212

cg08568550	0.0197727	8.04170598	1.39312062	46.4202698
cg03436149	0.01978143	441473.347	7.89663401	2.4681E+10
cg20265062	0.01978601	17.2670546	1.57274214	189.5741
cg10476085	0.0197898	39.0026123	1.79005065	849.810463
cg17850838	0.01979458	6582.39408	4.04309269	10716526.9
cg23253991	0.01980432	410.216573	2.60024998	64715.9458
cg01493678	0.01981191	12.4184617	1.49179769	103.377417
cg13554714	0.01981744	1554.21607	3.21077447	752337.982
cg22038796	0.01983431	73.7103258	1.97801744	2746.79687
cg00679681	0.01984956	9509195.84	12.7690037	7.0816E+12
cg15838173	0.01985603	2743.29785	3.50650409	2146206.85
cg09996156	0.01986781	290.719084	2.45582141	34415.2003
cg20994254	0.01987301	9.00164601	1.41619809	57.2163114
cg16765160	0.01987618	1.2017E+10	39.4448991	3.6607E+18
cg26104206	0.01988207	18.9265987	1.59277743	224.900311
cg14240963	0.01988297	3206751.73	10.711301	9.6004E+11
cg07036112	0.01988446	18.8216591	1.59130019	222.619751
cg25119415	0.01989375	0.13507571	0.02504443	0.72852308
cg20278503	0.01989538	73.8164792	1.97492788	2759.02358
cg01024247	0.0199097	29.8388286	1.71070029	520.462701
cg17428185	0.01991093	65.731391	1.93815354	2229.24328
cg04465286	0.01991651	478796.557	7.90334052	2.9006E+10
cg24385580	0.01992278	12.1669203	1.48415233	99.7430965
cg14145801	0.01992505	5.14078582	1.29522358	20.4039514
cg06395298	0.01993292	10.1058563	1.44102546	70.8719821
cg07268337	0.01993541	0.06386555	0.00629826	0.64760907
cg11931731	0.01993922	5.79283479	1.31967034	25.4282709
cg09940756	0.01993971	19765.6244	4.76801441	81937652.2
cg19536127	0.0199432	106.087645	2.08838593	5389.13249
cg21734015	0.01994331	2649.28779	3.47087403	2022178.2

cg09370702	0.01098637	618738.275	21.2841458	1.7987E+10
cg01627847	0.01099336	4.00027206	1.37412167	11.6453855
cg10449522	0.01099346	7051830.78	37.1469488	1.3387E+12
cg09250087	0.01100362	7.77229561	1.59979644	37.7601661
cg05103231	0.01101711	2255.43523	5.86057103	868002.119
cg04586579	0.01101719	7.27151338	1.57515869	33.5679873
cg22531889	0.01101883	1925.07202	5.65110313	655783.872
cg00090338	0.01102095	0.0300392	0.0020135	0.4481507
cg19998294	0.0110324	7.93429502	1.60645505	39.18755
cg00905524	0.01103606	31.4171276	2.20090176	448.468864
cg19489174	0.01104156	21.3206948	2.01373825	225.735408
cg09656239	0.01104548	9.4846E+11	549.098189	1.64E+21
cg16018130	0.01105359	3909675.28	32.165308	4.7522E+11
cg19757176	0.01105654	8.93440759	1.64986304	48.3819789
cg25644556	0.01105764	4.73845056	1.42714352	15.732765
cg14382259	0.01105801	45816928.8	56.4258927	3.7203E+13
cg22492279	0.01106023	201926.46	16.3218814	2498136950
cg24276070	0.01106615	19722165.5	46.47471	8.3694E+12
cg06624156	0.01108402	3180.06398	6.30753578	1603289.66
cg02384857	0.01109411	5.33392574	1.46543701	19.4145252
cg02952078	0.01110304	420.595731	3.96954	44564.5512
cg16400756	0.01110391	0.18099801	0.04838747	0.67704052
cg08302771	0.01110835	25.434018	2.09233124	309.171539
cg11357813	0.01111318	9.34E+11	537.468532	1.62E+21
cg08973950	0.01111419	3.93368213	1.36667061	11.3223003
cg25850845	0.01111499	10.3150201	1.70273845	62.4873657
cg12792157	0.0111162	114.711374	2.94932382	4461.59872
cg00674365	0.01111756	3.35933897	1.31829563	8.56041543
cg20994254	0.01112549	7.39071004	1.57775288	34.6205007
cg26742995	0.01112823	4.76370939	1.42737146	15.8984033

cg00090338	0.01995154	0.02249883	0.00092127	0.54945686
cg01706819	0.01995685	37.7059369	1.77311224	801.831742
cg13544006	0.01995966	6.65325573	1.34849066	32.8261908
cg26576398	0.019967	12.6826754	1.49277944	107.752192
cg26702921	0.01998142	0.08852484	0.011484	0.68239689
cg02483701	0.01998431	21.529211	1.62212752	285.740131
cg09022720	0.01998706	9.88243613	1.43473516	68.0700845
cg00667974	0.01998778	3.756E+11	66.6654457	2.12E+21
cg20199347	0.01999242	16.6040926	1.5568201	177.089114
cg09555914	0.02001379	6.08644346	1.32879633	27.8784589
cg08104202	0.02003049	5.65220265	1.3131472	24.3288755
cg14823287	0.02004758	6.7535E+12	103.852843	4.39E+23
cg07091842	0.02005165	1199.87509	3.0469351	472507.682
cg11649846	0.02005911	85315.1106	5.95147019	1223003370
cg19856002	0.02008305	263746.965	7.09160314	9809130597
cg09893504	0.02009128	14.8417554	1.52677598	144.276375
cg08826460	0.02009274	986.922021	2.94913634	330271.293
cg02225096	0.02009903	104925.9	6.12888224	1796321738
cg10235181	0.02009905	0.09394218	0.01278788	0.69011719
cg12111618	0.0200992	0.07890458	0.00927173	0.67149608
cg17355919	0.02010001	15318.2934	4.53212734	51774827.7
cg08425070	0.02010325	21.0106712	1.61193992	273.861513
cg18492126	0.02010661	19.7155976	1.59583224	243.574969
cg15831653	0.020113	79848.3025	5.86564039	1086965956
cg09013267	0.02011399	243.609954	2.36615185	25081.1501
cg20849121	0.02011471	21613194.5	14.1078535	3.3111E+13
cg03584535	0.02012455	10.3945829	1.44304948	74.8743242
cg23158347	0.02013238	1.3361E+11	55.239844	3.23E+20
cg19281068	0.02013752	26021.5529	4.91210086	137847580
cg26423145	0.02014716	8.48553839	1.39744263	51.5258089

cg11803389	0.01113111	11.9162715	1.75903986	80.72445
cg18456691	0.01114141	4342748361	156.930038	1.2018E+17
cg07719695	0.01114519	0.0730937	0.00969512	0.55106982
cg26779378	0.01114852	6.63376397	1.53872203	28.5995934
cg25560333	0.01115605	420.441205	3.95707124	44672.1316
cg15474579	0.01115619	12.1336752	1.76524703	83.4025337
cg11527279	0.01115707	13.207341	1.79961683	96.9283312
cg16586093	0.01116436	1.066E+12	546.469576	2.08E+21
cg26053876	0.01116509	11.7335873	1.75145637	78.6071944
cg11397548	0.01116646	102.088183	2.86556738	3636.97509
cg07757959	0.01116719	304.616816	3.67489837	25250.0601
cg13519902	0.01117309	14.0339739	1.82392674	107.98264
cg14160449	0.01117785	8699881545	182.386889	4.1499E+17
cg01236148	0.01118013	12.7393646	1.7838973	90.9757579
cg01093696	0.01119403	8.52346794	1.62760385	44.6358649
cg16232698	0.01119439	19.5289287	1.96514206	194.072003
cg12023170	0.01119593	125.406944	2.99885018	5244.31052
cg05245099	0.01119838	86687002	63.6994022	1.1797E+14
cg00541565	0.01119947	5993211.66	34.7019196	1.0351E+12
cg22619719	0.01120593	25688.4465	10.0444631	65697516.7
cg14784847	0.01121385	42.6886746	2.34583098	776.834716
cg08074851	0.01123478	6.15111937	1.51021903	25.053498
cg16054642	0.01123551	101.136227	2.8507953	3587.95891
cg03098361	0.01123822	6908428.48	35.6335392	1.3394E+12
cg14516246	0.01124045	22930319.7	46.7654495	1.1243E+13
cg01999333	0.01124264	0.09260343	0.0147124	0.58286836
cg03110633	0.01124394	1.2889E+12	558.676121	2.97E+21
cg10143146	0.01124593	9.04658699	1.64799865	49.6606816
cg15622309	0.0112461	14518.0961	8.79078913	23976813.7
cg04985324	0.01124975	1.0043E+13	888.605134	1.14E+23

cg00215224	0.02015554	44.5428602	1.81105923	1095.52816
cg13315345	0.02015699	85.8122441	2.00660976	3669.74256
cg23959605	0.02015981	9.66812978	1.42599232	65.5492546
cg15997906	0.02016007	4.80896684	1.27843414	18.0894434
cg19252931	0.02016245	0.14913994	0.02995236	0.74260316
cg22977667	0.02018325	2.011E+10	40.7305107	9.9286E+18
cg03994465	0.02019154	19.287445	1.58764646	234.312579
cg21852503	0.0201928	680653.691	8.14752242	5.6863E+10
cg21532077	0.02019487	30.443026	1.70482685	543.619919
cg19241089	0.02019949	17.0698473	1.55740706	187.092825
cg12656077	0.02020818	77.4330157	1.9716453	3041.04999
cg15692032	0.02020958	69549.071	5.69839635	848848163
cg18202577	0.02021835	2892.20082	3.46737149	2412439.97
cg08816023	0.02022138	17.394625	1.56133773	193.790858
cg20941453	0.02022331	34.1893734	1.73483144	673.79068
cg03296761	0.02023055	23.5530341	1.63661145	338.959754
cg20088847	0.02023216	0.06046747	0.00566278	0.64567489
cg02780181	0.02024717	16630317.8	13.3400497	2.0732E+13
cg24738356	0.02024987	0.11749284	0.01927153	0.71631921
cg18682936	0.02025565	10170531.4	12.3446824	8.3793E+12
cg07065111	0.02026551	6.62833956	1.34242262	32.7280579
cg06952384	0.02027083	175.938283	2.23621016	13842.2943
cg02580197	0.02031323	8.22189332	1.38727626	48.7282395
cg12955299	0.0203255	148088721	18.5700223	1.181E+15
cg25404454	0.02033341	2987.82991	3.46344223	2577530.39
cg11582116	0.02033931	609.784789	2.70554819	137435.175
cg26987699	0.02034362	9.4277394	1.4164404	62.7504482
cg18514705	0.02034954	70.4608295	1.93495735	2565.80771
cg02628508	0.02035079	3.51E-16	3.07E-29	0.00400519
cg11892243	0.0203598	2363779.33	9.73408935	5.7401E+11

cg27297326	0.01125075	7.41696596	1.57525633	34.9221794
cg07895149	0.01125093	11.9362713	1.75473723	81.1942497
cg11744300	0.01125722	1.1267E+13	910.141953	1.39E+23
cg19110758	0.01125865	34.6609313	2.23404438	537.760202
cg23589617	0.01126567	9.57561298	1.66867386	54.9492421
cg27126666	0.01126897	796.310802	4.54370416	139558.138
cg19809499	0.01127074	3.46583771	1.32530582	9.06359184
cg08266576	0.01128666	0.00853021	0.00021401	0.3400048
cg00131758	0.0112869	10.1733018	1.69093985	61.2062396
cg19060120	0.01128693	141.327757	3.06826959	6509.70664
cg26577320	0.01128993	9.22544246	1.65379824	51.4626191
cg24521848	0.01129382	6.80434586	1.54354842	29.9952512
cg05927159	0.01131238	5881356.4	33.9810047	1.0179E+12
cg09576143	0.01131698	7.6003826	1.58199126	36.5146237
cg26896499	0.01131792	104.35473	2.86072212	3806.69959
cg09307564	0.01131834	34.674239	2.22976059	539.20715
cg25283841	0.01131852	67917.8423	12.3794122	372621351
cg16155498	0.01132059	8.5991E+11	499.586812	1.48E+21
cg01211283	0.01132228	6.54065822	1.5290257	27.9787384
cg16699850	0.0113302	198908.672	15.7632777	2509925949
cg26404511	0.01133146	5.93740457	1.49570408	23.56935
cg08993385	0.0113323	15.5822471	1.8601357	130.531566
cg03259273	0.01133459	198310.967	15.7445895	2497825659
cg21097354	0.01134148	18.1320235	1.92446708	170.837049
cg24886748	0.01134201	218101805	76.5321243	6.2155E+14
cg14129855	0.01134321	8.9553021	1.6408926	48.8742747
cg14658900	0.01134911	2750897.3	28.4677825	2.6582E+11
cg16483033	0.01135225	17.8173031	1.91630737	165.660423
cg16317734	0.01135287	35.4518226	2.23836269	561.496015
cg26186134	0.01136047	26123.1366	9.933679	68697434.9

cg10272885	0.02036037	313168650	20.7648093	4.7231E+15
cg13908523	0.02037567	4.7997E+11	64.572623	3.57E+21
cg21504505	0.02039092	0.08591923	0.01079539	0.68382115
cg20942223	0.02039978	11.0625705	1.45070908	84.3590679
cg13078926	0.02040938	557.726971	2.66039604	116922.206
cg15190747	0.02041101	0.11031112	0.01711437	0.71101314
cg14505694	0.02041855	20226.3325	4.6342268	88278917.8
cg06032048	0.02042242	8374.49598	4.0424519	17348922
cg04914105	0.02043115	259.492677	2.36144549	28514.9285
cg27485194	0.02043211	756147.324	8.10369456	7.0555E+10
cg25250853	0.02044294	8374.89663	4.03745361	17372061.8
cg20615910	0.0204505	20.0861367	1.58940208	253.83941
cg26413730	0.02045802	465.972075	2.58223942	84085.9192
cg16584020	0.02046086	19695.8383	4.60227397	84290081.2
cg23665824	0.02047509	10.7041957	1.44160385	79.4807849
cg24616828	0.02048118	27495.7698	4.83878011	156241312
cg02622866	0.02048434	2138.67243	3.26278548	1401845.08
cg18764192	0.0204918	2996.98294	3.43569452	2614291.43
cg14398731	0.02050297	0.10147675	0.01465046	0.70288116
cg24028120	0.02050565	2.6445E+12	82.0447005	8.52E+22
cg16195332	0.02051199	9.39640203	1.41212704	62.5243826
cg02417264	0.02051391	0.01666581	0.00052184	0.53224944
cg20451727	0.02051644	227711.976	6.68475059	7756870430
cg24304712	0.02052394	12872.3747	4.29257386	38601090.2
cg12072333	0.02052536	393948.524	7.26797288	2.1353E+10
cg06994787	0.02052596	11.2858066	1.45221972	87.7067209
cg23363971	0.02052787	3.79191285	1.22773616	11.7114764
cg00467202	0.02053626	1910.78393	3.19816152	1141623.15
cg26080673	0.02054255	691856189	22.9031809	2.0899E+16
cg15718222	0.02054291	18.4680254	1.56608579	217.783703

cg18856581	0.01136371	4.72785076	1.41997149	15.7415645
cg06395298	0.01136529	8.20574302	1.60810219	41.8718528
cg00000363	0.01136727	11.3422817	1.72990768	74.3666014
cg04538470	0.01136741	9.2936E+16	6750.36832	1.28E+30
cg19414706	0.01136955	27.6240519	2.114639	360.859818
cg11472173	0.01137069	2678631231	134.082077	5.3512E+16
cg09091185	0.01137174	0.09305031	0.01479552	0.58520163
cg20097219	0.01138187	5.48605073	1.46802365	20.5015448
cg11474317	0.01138223	668914047	97.8246158	4.574E+15
cg11251498	0.01138465	15.0262936	1.84245499	122.548177
cg24550525	0.01139203	12.0881032	1.75392877	83.3113868
cg03525424	0.01139555	5.8849E+10	267.684349	1.2938E+19
cg04413872	0.0113991	3810342	30.4232216	4.7722E+11
cg11540838	0.01140335	2.7116E+12	633.39759	1.16E+22
cg04615531	0.01140354	77037.8304	12.6226788	470171775
cg14159304	0.01140417	20.1932828	1.96835173	207.162501
cg27042278	0.01140525	67.0220275	2.57919138	1741.61258
cg04772328	0.01140626	10.7908361	1.70906366	68.1321275
cg20633478	0.01141197	0.17937468	0.04738247	0.67905448
cg06093712	0.01141399	6.71944879	1.53584309	29.3981803
cg24921484	0.01141763	5.7173E+12	746.387681	4.38E+22
cg25048751	0.01142058	11.0407304	1.71734616	70.9802889
cg03896661	0.01142301	2246977.49	26.9203701	1.8755E+11
cg17237881	0.0114268	2827.28562	5.98360197	1335908.38
cg08122234	0.01143109	6.87934973	1.54352109	30.6607101
cg20433275	0.01145217	3.96895181	1.36341935	11.5537296
cg13613439	0.01145562	10.4229849	1.69391561	64.1346086
cg21131149	0.01145708	40397469.1	51.3046234	3.1809E+13
cg05299486	0.01145817	16.6573709	1.8821076	147.424094
cg05863534	0.01145947	25999.9629	9.82948122	68772507.6

cg19695710	0.020564	0.09232973	0.0122942	0.69339839
cg06080402	0.02057808	34988245.7	14.411806	8.4943E+13
cg27401681	0.0205801	7.5668E+11	66.7415505	8.58E+21
cg04618532	0.02058115	1.0268E+17	409.780524	2.57E+31
cg19056004	0.02059506	12.7151825	1.47738768	109.433608
cg01633093	0.02059577	12.603407	1.47536924	107.665163
cg03221702	0.02059889	177.778822	2.21442059	14272.4963
cg26816421	0.02060509	55.7704151	1.85321477	1678.34795
cg00065570	0.0206077	312.95059	2.41435762	40564.857
cg21534486	0.02060951	6357966.12	11.0543962	3.6568E+12
cg27494615	0.02061207	4272.60918	3.60442444	5064661.37
cg01682784	0.02064053	0.09067353	0.01187553	0.69232173
cg04671541	0.02064119	12.8758173	1.47907169	112.088328
cg07212963	0.02064457	12434.7669	4.23742154	36489980.3
cg12796272	0.02064615	12489.0912	4.23983103	36788588.3
cg08367083	0.02065085	9.00984577	1.40015719	57.977291
cg13116174	0.02065784	5.9495E+10	44.5838728	7.9393E+19
cg02792140	0.02066127	106310.215	5.87853948	1922562877
cg06361403	0.02066716	2633.08347	3.33683797	2077754.04
cg23008404	0.02066959	9.06250516	1.40102085	58.6208262
cg08611810	0.02067644	9.59974879	1.41327311	65.2069133
cg06318386	0.02068735	802.187667	2.77949856	231518.398
cg24664672	0.0206986	3634.17944	3.49944886	3774097.22
cg17008160	0.02072767	11.4805001	1.4512678	90.8184439
cg17217059	0.02073904	21.1986802	1.59327689	282.050185
cg11587658	0.02078344	18975.6596	4.47975756	80378380.7
cg10531073	0.02078797	8.2819065	1.37954542	49.7192585
cg08104568	0.02078824	19.1883671	1.56773025	234.857643
cg14482741	0.02080303	12.7913522	1.47352562	111.038918
cg17730428	0.02081628	238.69656	2.2985385	24787.9459

cg00171166	0.01146493	0.00208141	1.74E-05	0.2496188
cg18895088	0.01146605	5.63885876	1.47512548	21.5552702
cg05637351	0.01146821	12.6276493	1.76806452	90.1876175
cg07135540	0.01147168	14.0784921	1.81165468	109.404922
cg05028274	0.01147396	7.36E-05	4.59E-08	0.11785829
cg23973885	0.01147789	20.7037637	1.97530291	217.002582
cg04784315	0.01147922	0.01667499	0.00069743	0.39868687
cg17319109	0.01148274	3.186E+13	1078.24257	9.41E+23
cg11105830	0.01148726	828.276923	4.52141547	151731.834
cg17228821	0.01149481	1597702.63	24.6917218	1.0338E+11
cg04478991	0.01149858	18.2772797	1.91967381	174.018603
cg07239293	0.01150167	569.324211	4.15280826	78050.812
cg23105863	0.01151019	5.64129423	1.4742126	21.5872531
cg20516737	0.01151434	1.1989E+11	305.439612	4.7063E+19
cg13460057	0.01151529	20.9733262	1.97893859	222.280981
cg12249359	0.01151731	75880.4642	12.4295573	463238127
cg13145017	0.01152375	90761850	60.8444275	1.3539E+14
cg01027365	0.01152709	390.020534	3.80943063	39931.4313
cg24237081	0.01152756	8.09637018	1.59813359	41.0173533
cg17325073	0.01152926	4.6596E+16	5448.88264	3.98E+29
cg09299055	0.01153444	7.65721546	1.57807201	37.1547993
cg16656078	0.01153516	169.725507	3.16000503	9116.0449
cg26830834	0.01153703	105256275	62.7553707	1.7654E+14
cg00694371	0.01153732	2663.23049	5.85554478	1211295.77
cg27662481	0.01154188	0.15907298	0.03820018	0.66241086
cg19657320	0.01154365	24264272.8	45.1221184	1.3048E+13
cg09652501	0.0115593	5550.88144	6.89079188	4471515.79
cg04981056	0.0115618	9.30052128	1.64741091	52.5064484
cg13702222	0.01156862	5.55904628	1.46798654	21.0512799
cg25161465	0.01156878	0.11235225	0.02058877	0.61310252

cg13813391	0.02082612	439.411243	2.52095348	76590.9575
cg11609154	0.02082702	2942.5177	3.36531353	2572839.16
cg25165701	0.02084527	11.4541276	1.44799387	90.6060734
cg17569080	0.02085603	11808.3157	4.14887129	33608253.8
cg06969845	0.02085699	0.07265365	0.00785787	0.67175353
cg03019033	0.02086326	410.077146	2.49092731	67510.3063
cg02083373	0.02087379	4.9692E+10	41.8634864	5.8984E+19
cg16131766	0.02087706	0.16999544	0.03780451	0.76441807
cg22907891	0.02089774	0.00213538	1.16E-05	0.39400629
cg19109608	0.02089926	10.3742841	1.42519426	75.5165622
cg05783676	0.0209098	2437.28701	3.25637375	1824227.94
cg17884201	0.02091517	0.06998857	0.0073259	0.66864101
cg02461665	0.02091614	15.2896654	1.5109597	154.718797
cg25826098	0.02091936	192779084	17.9360477	2.072E+15
cg03807235	0.02092019	0.00012205	5.82E-08	0.25575467
cg09755939	0.02095443	36.9260844	1.72508547	790.416324
cg15701440	0.02095463	81569.9474	5.52183481	1204971998
cg01098651	0.02095682	401.169732	2.47342877	65066.4194
cg02605634	0.02096625	43.336157	1.76679423	1062.95486
cg24727216	0.02096982	14.3536536	1.49516532	137.795713
cg07983905	0.02098431	5.71572128	1.30087976	25.1133662
cg18237323	0.02099089	6.33838586	1.32123036	30.4073661
cg10560079	0.02100548	1718.94742	3.07416637	961164.713
cg23527902	0.02101125	17.8219984	1.5436129	205.766372
cg04465834	0.02101534	116.023596	2.04693009	6576.42145
cg13719287	0.0210238	12.6172015	1.46501027	108.66393
cg23588217	0.02105163	11.9595298	1.45258134	98.4663293
cg15945016	0.02106149	34.6722812	1.70446346	705.305284
cg26464221	0.02106567	9.92840509	1.41216861	69.8027323
cg11228744	0.02107003	0.12133367	0.02021459	0.72827892

cg25860425	0.01156992	2.9267E+10	219.848816	3.8962E+18
cg10415607	0.01157957	8241.57407	7.5154608	9037841.46
cg20277282	0.01158051	5.82026521	1.48285963	22.8447025
cg02045669	0.01159021	7.45091083	1.56680236	35.4327218
cg12691534	0.01159523	7.4148425	1.56495641	35.1318981
cg09101894	0.01159574	11.0090079	1.70949181	70.8972423
cg05414235	0.01160069	1413.6785	5.05903654	395033.102
cg15081886	0.01160688	7.64423658	1.57530346	37.0940295
cg05108179	0.01161201	2486.31956	5.7346957	1077962.16
cg13466546	0.01161769	172.499342	3.15894808	9419.59858
cg14205126	0.0116198	8440.26306	7.53003722	9460516.38
cg23466060	0.01162528	11.4986262	1.72504094	76.6465315
cg19305681	0.0116305	7.11339794	1.54950505	32.6558666
cg03019000	0.01163146	8.3001764	1.60377047	42.9568504
cg07037112	0.01163576	8.55651209	1.61455659	45.3461339
cg00794055	0.01163655	4.82556459	1.42081316	16.3892582
cg00731944	0.01163997	0.15672681	0.03714228	0.66132976
cg27299526	0.0116413	0.00420873	6.00E-05	0.29506873
cg09796739	0.01164144	12.9140007	1.76965236	94.2396465
cg14470448	0.0116416	8.95140278	1.63070088	49.1369156
cg12392916	0.01164317	8056.14771	7.43755381	8726191.11
cg19573564	0.01164837	236799.598	15.802567	3548413963
cg22168087	0.01164995	102.136557	2.80613048	3717.53075
cg09874107	0.01165396	307.837699	3.58820962	26409.842
cg16642791	0.01165434	7.2178441	1.55387341	33.5273602
cg07493874	0.01165938	11.7832971	1.73312866	80.1129732
cg27343303	0.01167401	393.360489	3.78561379	40873.814
cg11804775	0.01168559	780.234189	4.40651833	138151.108
cg24963041	0.01168918	641.348785	4.21738653	97531.5543
cg19886978	0.01170115	873.128377	4.5138863	168890.644

cg23169269	0.02108716	0.07536359	0.00837508	0.67816303
cg09540111	0.02108991	983.688813	2.8152331	343717.073
cg20201566	0.0210925	383706.208	6.89599743	2.135E+10
cg14457918	0.0210992	3014.51987	3.32928087	2729517.39
cg12230071	0.0211022	121685.22	5.79920551	2553331260
cg00190795	0.02111268	9.89112394	1.41036693	69.3679998
cg25826457	0.02111322	3940.48645	3.4632446	4483493.15
cg02944245	0.02111757	4513269.88	9.96005302	2.0451E+12
cg01120874	0.02113992	34.9049351	1.70304491	715.397749
cg25754252	0.02114873	193317.705	6.19333927	6034181806
cg16963127	0.02115562	52.5278851	1.80987993	1524.50927
cg09578614	0.02115828	0.00230271	1.32E-05	0.40272524
cg03921416	0.02116268	94.8599154	1.97697301	4551.60667
cg21145140	0.02116295	15.1343953	1.50195681	152.501005
cg20776123	0.02117107	1771949.48	8.61285556	3.6455E+11
cg16003274	0.02118035	23740.0271	4.51408656	124851148
cg24425705	0.0211866	268.067848	2.30762007	31140.4689
cg01656221	0.02119965	34.4828667	1.69757642	700.450407
cg04558861	0.0212009	81.0859897	1.92894189	3408.57221
cg13188519	0.0212018	12157.7524	4.07864003	36240252.1
cg01522826	0.02120502	26.3903588	1.63086015	427.045224
cg07117311	0.02121216	25.671804	1.62389854	405.839098
cg15227217	0.021215	44485.5938	4.9466516	400062145
cg02068689	0.02123781	278.128786	2.31597555	33400.8801
cg14908680	0.02125645	2.34E-05	2.67E-09	0.20390404
cg01911068	0.0212613	15806.4257	4.22578861	59123424.2
cg11698816	0.02126859	11390542.7	11.2605653	1.1522E+13
cg17016559	0.02129334	0.00388725	3.45E-05	0.43772606
cg17081801	0.02130672	55641491	14.1995198	2.1803E+14
cg06973760	0.0213161	780444096	21.0065778	2.8995E+16

cg21818602	0.01170263	5.2896E+11	406.387695	6.88E+20
cg27508071	0.01170737	21.9325638	1.9878713	241.986165
cg11260715	0.0117075	83.4311375	2.67600764	2601.17147
cg01822130	0.01171016	4.61240037	1.40509129	15.140822
cg03080561	0.01171057	18659018.8	41.4510161	8.3993E+12
cg13668129	0.01171108	30.3222861	2.13617354	430.414953
cg15588266	0.01171468	742729.751	20.2229462	2.7278E+10
cg04768602	0.01171664	5.996236	1.48939126	24.1406319
cg23732368	0.01171784	1441.48972	5.04103159	412195.911
cg16629179	0.01172511	17.4082456	1.88738379	160.564595
cg08641866	0.01173082	638.993887	4.20344009	97137.863
cg13672975	0.01173827	1721943306	112.808748	2.6284E+16
cg15203597	0.01174507	759.743425	4.36449308	132251.343
cg19347782	0.01174617	6.24919061	1.50238906	25.9935221
cg19655952	0.01174827	14.1401955	1.80109696	111.012972
cg06795971	0.01175035	21244.5531	9.14275414	49364888.4
cg21445527	0.0117543	306.547264	3.56580543	26353.4359
cg05628616	0.01176402	11.0703974	1.70520084	71.8705364
cg20088847	0.01176642	0.07015254	0.00887581	0.55447116
cg12544974	0.01176662	6.70571743	1.52555656	29.475568
cg21691089	0.01176865	2.4677E+10	202.46991	3.0077E+18
cg08767936	0.01176997	8.11481488	1.59142034	41.37827
cg16667508	0.01177629	7.85509113	1.57978325	39.0575458
cg01748892	0.01178215	17.1230597	1.8776498	156.152214
cg16017089	0.01178428	3.53086177	1.32285395	9.42430935
cg21926138	0.01178518	31.4657311	2.14875115	460.775662
cg02764271	0.01178528	1.4169E+13	825.38312	2.43E+23
cg05502525	0.01178684	5721397814	145.827931	2.2447E+17
cg23041619	0.01178836	442111.979	17.8600847	1.0944E+10
cg04771029	0.01179025	3527760.96	28.2994768	4.3976E+11

cg06116248	0.0213271	1396047.46	8.19119795	2.3793E+11
cg22354234	0.02132982	25742.0997	4.52373924	146484061
cg00088227	0.0213379	1.2572E+13	88.3283762	1.79E+24
cg13388731	0.02133844	23.9642531	1.60305684	358.243956
cg27662875	0.02133871	53224.0994	5.0362282	562485386
cg21926750	0.02134045	89688.0103	5.44147108	1478265543
cg25349574	0.02134105	20.0167872	1.56067958	256.729041
cg23027583	0.02135332	4.20750434	1.23778338	14.3022544
cg17538898	0.02136353	1080923.74	7.85945431	1.4866E+11
cg07562483	0.02137165	10.4408761	1.41619312	76.9753022
cg10440696	0.02140941	0.14909787	0.02946783	0.75438789
cg01287088	0.02140985	13.3127566	1.46722267	120.792496
cg02962602	0.0214183	12.2728323	1.44945472	103.916605
cg16101148	0.02142181	24.229324	1.60288598	366.251964
cg17426063	0.02143404	110.945739	2.00697693	6133.08346
cg13277380	0.02143409	10.0675839	1.40723603	72.0250502
cg09527362	0.02143588	135.492879	2.06708523	8881.25949
cg27280575	0.02144995	7.60805165	1.34982993	42.8812908
cg09758595	0.02145461	95077.2493	5.44201351	1661091675
cg16635274	0.02146799	483717.386	6.91329397	3.3845E+10
cg13694927	0.02147897	9.43135107	1.39279379	63.8647183
cg26824174	0.02148906	19.1575987	1.54611291	237.378259
cg23609905	0.02149151	927305.624	7.59479655	1.1322E+11
cg11550381	0.02149546	10940598	10.9267412	1.0954E+13
cg23067317	0.02149785	8.33885613	1.36734849	50.8550102
cg22610434	0.02150269	0.15636424	0.03214609	0.76058325
cg09701102	0.02150274	0.00231859	1.32E-05	0.40870395
cg00938535	0.02150468	1870.88234	3.03764987	1152272.61
cg10920316	0.02150609	8.64341413	1.37444365	54.3555261
cg18706864	0.02152301	14966712.9	11.409779	1.9633E+13

cg03671191	0.0117923	8.44856278	1.60501199	44.4720746
cg02223962	0.0117939	12454.6728	8.08949017	19175358.5
cg05789714	0.01179427	22.9231479	2.00245809	262.412837
cg21692194	0.01179478	8.47016445	1.60584231	44.6766691
cg08193141	0.01179767	0.01234206	0.0004035	0.37751447
cg20011278	0.01179785	253.6525	3.41133092	18860.5539
cg09259081	0.01180807	6.00746065	1.48775458	24.2577533
cg18592307	0.01181084	214.070409	3.2833117	13957.2919
cg05099952	0.01181241	9.21339784	1.63547474	51.9034001
cg14083397	0.01181512	326.279711	3.6038131	29540.5026
cg13225596	0.01182251	9.58748924	1.64961108	55.7221948
cg08469255	0.0118262	34.4805634	2.1898629	542.914928
cg13773631	0.01182783	7.87428597	1.57908884	39.2659223
cg08512882	0.01183274	3.0336E+13	964.000284	9.55E+23
cg01706263	0.01184244	0.10419208	0.01790493	0.60631282
cg01074928	0.01184312	522.789019	3.99402055	68429.3821
cg24203851	0.01184712	926.994293	4.53247548	189591.411
cg16400971	0.01185062	412202.348	17.4548319	9734311733
cg05011526	0.01185262	46.8287061	2.34122319	936.658978
cg26504026	0.01185357	13.4228815	1.77590351	101.454694
cg05327187	0.0118536	0.01926146	0.00088861	0.41750826
cg10272885	0.01186193	303088985	74.9927179	1.225E+15
cg21973275	0.01186323	107898346	59.6706775	1.9511E+14
cg03712341	0.01186919	2345474267	117.71965	4.6732E+16
cg04021672	0.01187009	19.3334684	1.9242776	194.245883
cg25767504	0.01187263	47.5352636	2.3473216	962.629613
cg26296364	0.01187449	8.00302106	1.58335343	40.4510736
cg08892236	0.01187712	49.9885963	2.37318626	1052.9556
cg01036779	0.01188933	1019.68136	4.61660895	225219.439
cg04578193	0.01189016	30.140184	2.12134228	428.233906

cg25492645	0.02152372	7588.10342	3.73019534	15436004.9
cg08741214	0.0215242	470.043897	2.47586576	89237.9824
cg02409878	0.02152581	4.2293031	1.23671946	14.4632678
cg10474377	0.02152589	558.744063	2.53956074	122932.648
cg06726167	0.02157419	6.63E-06	2.54E-10	0.1732352
cg12783491	0.02158648	1145.97984	2.81522267	466488.785
cg15616111	0.02158792	341276686	17.937724	6.493E+15
cg02606566	0.02159468	34454910.7	12.7971869	9.2766E+13
cg10683958	0.02160111	6945.679	3.66549044	13161255.7
cg03236948	0.02160732	22.0945165	1.57523532	309.901416
cg07273992	0.02161222	28.4782872	1.63486195	496.074203
cg00741410	0.02161238	9.06594461	1.38203775	59.4711335
cg00013441	0.02161531	238.434829	2.23296629	25459.931
cg23713813	0.02161727	2153017.99	8.49771299	5.455E+11
cg08971171	0.02162229	711.192385	2.62061453	193006.107
cg14022550	0.02162303	261.693488	2.26304279	30261.6823
cg12821132	0.02163551	78371.5448	5.2189188	1176891089
cg13997645	0.02164398	6.66864138	1.32061048	33.6744093
cg11699666	0.02164498	1077.68999	2.78251019	417398.55
cg11849105	0.0216464	562175.815	6.96045313	4.5405E+10
cg14967868	0.02164793	155533.281	5.76506623	4196066558
cg20772300	0.0216527	343.411149	2.35243156	50131.6251
cg00569934	0.02166265	2218.33217	3.09031636	1592392.83
cg16385583	0.02166297	0.05037439	0.0039307	0.64558007
cg00149397	0.02166544	5.9284E+12	74.1783753	4.74E+23
cg20929143	0.02167216	25999745.8	12.1734324	5.553E+13
cg23130076	0.02167463	1473658850	21.9753123	9.8823E+16
cg00358323	0.02167948	206128.986	5.99291362	7089900129
cg02372889	0.02168258	10.7135277	1.41479366	81.1282099
cg21581821	0.02168464	9.80014264	1.39642473	68.7776388

cg09565397	0.01189464	9.6121231	1.64807204	56.0612088
cg21218636	0.01189845	28.2643408	2.0909234	382.067061
cg11700584	0.01189864	52.7715353	2.39987988	1160.40597
cg19721115	0.01190826	108.924722	2.81501159	4214.75886
cg06304787	0.01191226	258608.881	15.6339825	4277768209
cg10956904	0.01191384	188601.545	14.579691	2439732284
cg03731862	0.01191743	262530.472	15.6767045	4396475592
cg17430967	0.01191943	1463.24577	4.98981668	429091.551
cg15462862	0.0119197	49.5596908	2.36506159	1038.51966
cg04107003	0.01192317	19.7350673	1.93023285	201.775077
cg11521427	0.01192401	9.53190486	1.64402665	55.265047
cg02086523	0.011925	4.1595E+12	605.870937	2.86E+22
cg00852573	0.01192706	7.86617051	1.57576177	39.2677623
cg25697727	0.01192763	14.9180067	1.81452941	122.647186
cg13063614	0.01193741	20.5177568	1.94609107	216.319961
cg08233142	0.01194707	52719.758	10.9697403	253367245
cg06974871	0.01195056	5.02369177	1.42693708	17.6864694
cg01869698	0.0119538	33350.9852	9.91082543	112229624
cg24330746	0.01195463	0.0023889	2.16E-05	0.26462385
cg04458368	0.01195545	10.1395693	1.66547171	61.7307785
cg04520793	0.01195651	234.909846	3.32718637	16585.3755
cg04997812	0.01195669	30.9535259	2.12938856	449.951119
cg23747525	0.01195743	78.376652	2.61268921	2351.17883
cg04364728	0.01195886	11.0532564	1.6972905	71.9820663
cg20356664	0.01196088	49086.2588	10.7837831	223433722
cg27468419	0.01196431	6.25360287	1.4971065	26.1220889
cg10029031	0.01197069	233625985	69.4512	7.8589E+14
cg00028034	0.01197576	14.0969657	1.7899354	111.023249
cg04614943	0.01198082	9362863.36	34.1639572	2.566E+12
cg24144070	0.01198316	227.851222	3.30053892	15729.6068

cg19276111	0.02168863	11.2032687	1.4239415	88.1449335
cg11948456	0.02168957	12.2510645	1.44266406	104.035711
cg19285119	0.02169055	153407.165	5.73443923	4103933658
cg11670109	0.02169239	295.004554	2.29727023	37883.0865
cg01917164	0.02170132	7524.04459	3.6872744	15353141.9
cg00747184	0.02170579	30528.9134	4.52374016	206027429
cg02396009	0.02170834	981444.639	7.51083845	1.2825E+11
cg04808029	0.02170915	7.81132293	1.3504036	45.1840959
cg11826475	0.02171943	46.2645039	1.75085292	1222.49236
cg19450531	0.0217219	5441.4643	3.51262234	8429466.92
cg16522557	0.02172225	6.28688827	1.30802533	30.2172773
cg10439144	0.02172959	891929692	20.2684815	3.925E+16
cg02249490	0.02173431	19.7545609	1.5457465	252.462273
cg02264779	0.02173646	34.5679748	1.67722097	712.455248
cg08468187	0.02173764	81.1657746	1.89969616	3467.86139
cg20256097	0.02173896	99777.0075	5.36531278	1855521128
cg06659904	0.02174038	148581803	15.5812033	1.4169E+15
cg23644624	0.02174047	10.91123	1.4173095	84.0006645
cg01733284	0.02174206	0.19144097	0.04664879	0.78565047
cg12691330	0.02174301	7.33940506	1.33757486	40.2720387
cg08690336	0.02176897	308.475768	2.30573958	41269.7515
cg24296484	0.02178255	13962.8585	4.01583027	48548221.8
cg06404175	0.02178369	3.18402312	1.18375554	8.56427103
cg12505694	0.02178567	277661080	16.972627	4.5424E+15
cg15063443	0.02179424	9.27023043	1.38292108	62.141776
cg09122913	0.02179897	533.92982	2.49463564	114277.632
cg11571563	0.02180125	9.88926468	1.39585191	70.0629882
cg27579745	0.02181017	125.446856	2.01973877	7791.55897
cg22628146	0.02182009	257.512224	2.24171785	29581.1293
cg26518580	0.02183612	10.8811177	1.41464195	83.6951876

cg15914589	0.01199207	515.698521	3.94814469	67359.4779
cg11753867	0.0120016	1935.91883	5.27745096	710149.986
cg24385580	0.01200434	9.88278586	1.65439568	59.0363344
cg11856711	0.01200654	47.2246133	2.33286525	955.976387
cg01611862	0.01201079	89.823131	2.68640095	3003.34723
cg08134704	0.01201801	68646.7201	11.5429051	408248370
cg23414431	0.01201888	0.00137812	8.07E-06	0.23533988
cg20320141	0.01201937	1429.93002	4.93167164	414605.837
cg19430553	0.01202802	5.89490932	1.47624079	23.5394904
cg23069367	0.01202866	5.29380883	1.44177704	19.4374103
cg12621171	0.01203362	11.5651348	1.71142968	78.1524028
cg10504436	0.01203575	16.4961338	1.8500726	147.087434
cg19973659	0.01204524	689878.133	19.0975597	2.4921E+10
cg04808029	0.01204786	6.9421663	1.52966697	31.5059904
cg16532497	0.01205196	8.01113943	1.57836579	40.6612684
cg05044743	0.01205414	7.02399511	1.53343319	32.1738877
cg00470934	0.01205621	6.5989E+11	390.762527	1.11E+21
cg08940505	0.01205654	56.262555	2.41993333	1308.0836
cg24880056	0.01205792	6.2175697	1.49287035	25.8951978
cg06096427	0.01206709	611523.631	18.549817	2.016E+10
cg16492377	0.01208546	8.82282484	1.61106189	48.3173482
cg19948397	0.01208822	1268146.61	21.7068463	7.4087E+10
cg09355771	0.01209273	5.12353043	1.43010161	18.3557336
cg01086810	0.01209607	118122971	58.5152291	2.3845E+14
cg16044810	0.01210494	44.9266282	2.29957704	877.727462
cg15485645	0.0121069	204.845776	3.20487654	13093.1072
cg09505788	0.0121112	6.42322922	1.50220662	27.464846
cg19147218	0.01211161	4.49736973	1.3895028	14.5565266
cg03595018	0.01211369	0.14861222	0.0335149	0.65897835
cg03005685	0.01211862	46.456855	2.31539019	932.127719

cg18843682	0.02184247	11.0131318	1.41698358	85.5966676
cg07223632	0.02184727	1786.04998	2.96702658	1075141.88
cg24514884	0.02185362	28.1208435	1.6233219	487.138034
cg00744739	0.02186312	0.16195356	0.03416111	0.76780158
cg05311410	0.02186363	4.98402379	1.26254305	19.6749672
cg26198823	0.02186894	0.00577516	7.05E-05	0.47336071
cg07627556	0.02188548	9.20825091	1.37975998	61.4540834
cg13340335	0.02188612	58.8906602	1.80569918	1920.64652
cg10552592	0.02189941	1471677.93	7.83003064	2.7661E+11
cg26342670	0.02191269	662.715886	2.56206301	171421.368
cg03699566	0.02191878	7.10132699	1.32818637	37.9681995
cg07950803	0.02192582	0.17422262	0.03908853	0.77653277
cg03189237	0.02193427	9816.08199	3.7806193	25486688.3
cg05782110	0.02193793	3.0872E+12	64.0802076	1.49E+23
cg10985065	0.02194445	0.10692239	0.01579611	0.72374778
cg07657064	0.02195296	9.98210919	1.39459849	71.4488828
cg19043574	0.02195535	7.05484599	1.32631184	37.5257542
cg02577849	0.02196369	1.8134E+10	30.3579932	1.0832E+19
cg24801889	0.02196561	26816481.6	11.8373595	6.075E+13
cg06164260	0.02196834	94.5809621	1.92943059	4636.37222
cg11267955	0.02197483	12.8022083	1.44515538	113.411015
cg06483978	0.02197684	6096.35297	3.52040318	10557177
cg04077706	0.02197874	22.4448622	1.56709313	321.468985
cg09232851	0.02198529	0.13193685	0.023319	0.74648706
cg24960947	0.0219935	4.83944514	1.25549779	18.6541382
cg09619624	0.02199595	4.14544144	1.2277379	13.9970305
cg26710686	0.02199688	33352384.3	12.1737013	9.1376E+13
cg04362989	0.02200847	7.97588497	1.3490858	47.1539625
cg27227156	0.02201216	18.0280554	1.51733735	214.198103
cg23801544	0.02201227	18.7109782	1.52549021	229.500462

cg04796763	0.01213797	3.68359201	1.32971299	10.204345
cg07594531	0.01213945	16.8592695	1.85399069	153.309815
cg16194715	0.01214825	10.046213	1.65536152	60.9693982
cg05899618	0.01214941	6.35865757	1.49792871	26.99229
cg14097171	0.01214957	11.9987909	1.72080088	83.6651026
cg22662233	0.01215326	267.139272	3.38862828	21059.6692
cg24999111	0.01215689	16345694.7	37.6047199	7.105E+12
cg19594360	0.01215924	2889.26933	5.69748915	1465185.28
cg12111618	0.01216123	0.09215657	0.0142934	0.5941785
cg01876338	0.01216414	7.00319058	1.5294589	32.0666859
cg08586500	0.01216431	22.1792364	1.96713188	250.068911
cg25709824	0.01217046	8.83018714	1.60865147	48.4705397
cg02029441	0.01217532	68.7335481	2.51703334	1876.93208
cg18348142	0.01218161	59.4943636	2.4384162	1451.58948
cg17088155	0.01218412	375470.666	16.4431764	8573661057
cg03894789	0.01218657	0.05633589	0.00594339	0.53399326
cg16830870	0.01219498	5602338.57	29.604855	1.0602E+12
cg04436474	0.01219712	20959.6264	8.75220928	50193719.7
cg00510149	0.0122071	7.15198189	1.53532016	33.3160771
cg24659411	0.01220771	75299.2997	11.5541216	490732634
cg22537328	0.01220932	0.00299457	3.18E-05	0.28189438
cg02446378	0.01221248	78781.2903	11.6628334	532159851
cg17346246	0.01221409	6.55082095	1.5060446	28.4940136
cg23816347	0.01221542	7.65202618	1.55786053	37.5858451
cg04796973	0.01221596	900934713	89.2718001	9.0923E+15
cg13549667	0.01221681	24.2992148	2.00369	294.682232
cg13052102	0.01222763	10.8222349	1.6796135	69.7307855
cg24101049	0.01223459	8.71309317	1.60195939	47.3907099
cg18514705	0.01223837	37.444398	2.2000491	637.296207
cg23231163	0.01224025	351.144819	3.58061135	34436.2099

cg00448482	0.02202562	76039.1486	5.05035897	1144859635
cg13577076	0.02202668	8.89429893	1.3701044	57.7390697
cg20814026	0.02202697	15.1138686	1.47886742	154.462138
cg06286401	0.0220486	1354571.16	7.63203522	2.4042E+11
cg08274892	0.02204948	349216.21	6.27866909	1.9423E+10
cg07469744	0.02204969	123.446571	2.00005779	7619.30778
cg21741562	0.02206798	5.85543161	1.28940753	26.5905685
cg13975336	0.02207812	5752123.88	9.37035209	3.531E+12
cg08881995	0.02207949	1.1985E+11	39.1298437	3.67E+20
cg00858015	0.02208727	65079.0864	4.91666451	861414783
cg24789467	0.02209104	19.0179075	1.52678906	236.889833
cg25717844	0.02211568	11.0698952	1.41204328	86.7838691
cg16449219	0.02212985	15.7971644	1.48560121	167.979402
cg13736939	0.0221395	0.07393334	0.00794042	0.68839442
cg17349352	0.02214961	14.5696	1.46796553	144.603699
cg18720905	0.02216164	10.988244	1.40954524	85.6599015
cg07394955	0.02217137	38.7925874	1.68825147	891.374814
cg11299660	0.02217255	200492.175	5.74090906	7001872348
cg03081691	0.02218813	5.60061866	1.27947881	24.5153957
cg04586579	0.02220242	7.1562875	1.32489751	38.6538963
cg00670698	0.02220712	2152327.86	8.03771882	5.7635E+11
cg27495887	0.02222105	3363.8501	3.18976629	3547434.67
cg14844619	0.02222389	14845812.1	10.5738172	2.0844E+13
cg03476370	0.02224444	2.10E-06	2.84E-11	0.15481713
cg01697163	0.02225179	50.1282055	1.74782351	1437.69493
cg06184463	0.02225827	221.785582	2.16034712	22768.9539
cg19824362	0.02225984	4272955155	23.6166531	7.731E+17
cg07748159	0.02226584	0.09002461	0.01142291	0.70948889
cg05216888	0.02226595	733928035	18.3561214	2.9344E+16
cg19750232	0.02226898	272636.43	5.95286324	1.2487E+10

cg15395441	0.01224081	10.9907883	1.68479437	71.6986175
cg04100696	0.01224181	4.69727915	1.40023212	15.7576955
cg02695062	0.0122455	37.9625502	2.20612317	653.25238
cg02045285	0.01224982	208.154045	3.19399781	13565.4778
cg14986464	0.01225422	12.6699645	1.7372037	92.4059743
cg08729318	0.01225592	4.06899314	1.35691437	12.2017318
cg26232708	0.01226595	18418601.8	37.9669397	8.9353E+12
cg25456368	0.01226897	19.9990161	1.91775421	208.55678
cg12210040	0.01227442	367.934626	3.61061505	37493.8584
cg05376156	0.01227518	319580.549	15.7105548	6500835170
cg15440363	0.01227992	71894.6173	11.3551759	455196473
cg14781281	0.01228005	6.5080607	1.50222224	28.194799
cg16836311	0.01228059	4.74418208	1.40249918	16.0479692
cg20945607	0.01228419	79305128.1	51.9928999	1.2096E+14
cg03721195	0.01230002	10.5679066	1.66837038	66.9399619
cg00350199	0.01230534	218.584591	3.21946536	14840.7323
cg01815035	0.01230604	44134.4779	10.1872979	191204004
cg15372689	0.01231236	6.91966233	1.52151558	31.4697578
cg27539986	0.01231519	5.56296454	1.45108549	21.3264999
cg09919392	0.01232343	0.07741235	0.0104378	0.57413163
cg11479568	0.01233058	17.2200783	1.85346687	159.987265
cg04142824	0.01233251	106071.366	12.2888934	915553124
cg10538030	0.01234167	246165412	65.836437	9.2042E+14
cg02261765	0.01234296	7.5123255	1.54802963	36.4560428
cg13395333	0.01234593	803.256604	4.2599173	151463.309
cg19388996	0.01234748	410.404513	3.68276432	45735.1733
cg17471836	0.0123508	6.86926847	1.51809382	31.08296
cg09578614	0.0123647	0.00417427	5.71E-05	0.30538441
cg06710082	0.01237241	4.39380158	1.37763108	14.0135429
cg25346720	0.0123777	9698401.74	32.496207	2.8945E+12

cg09600247	0.02227249	496.218599	2.42186404	101670.818
cg00357958	0.02227919	8.73374445	1.36171557	56.0163177
cg06457011	0.02227945	7.25987586	1.32632277	39.7382889
cg13374726	0.02227997	2965067.22	8.35591587	1.0521E+12
cg13356253	0.02228239	9.07669328	1.3691459	60.1735438
cg06038049	0.02228838	3294777626	22.6662044	4.7893E+17
cg01870903	0.02228973	3073.34622	3.13776636	3010248.67
cg24542360	0.02229236	13493.3569	3.87294672	47010892.4
cg04200399	0.02230335	39136.5795	4.50363535	340096774
cg13240116	0.02230964	33.1842572	1.64581182	669.089205
cg07328579	0.0223134	11.9500193	1.42314537	100.3432
cg25121621	0.02231736	3267.79878	3.16076855	3378453.27
cg01424889	0.02232778	598.631353	2.48186871	144390.997
cg03513643	0.02234632	23827.2141	4.18495178	135661332
cg10420952	0.022364	11.3991437	1.41251037	91.9925836
cg03756121	0.0223836	45057.7454	4.56963173	444280970
cg09231767	0.02238903	0.14127265	0.0263393	0.75772565
cg12796015	0.02240107	2.161E+11	40.3583049	1.16E+21
cg03555914	0.02241332	875.007627	2.60978116	293372.624
cg23062425	0.02241462	34293.1885	4.38703548	268067761
cg07948472	0.02242179	526.995255	2.42814472	114377.037
cg12106855	0.02242566	8.02132049	1.34268651	47.9200334
cg27539234	0.02243876	206.013999	2.12463231	19976.0531
cg08932052	0.0224574	928237.975	6.97223818	1.2358E+11
cg25625860	0.02247672	3538827.01	8.40819004	1.4894E+12
cg26087598	0.02247894	871816849	18.2922117	4.1551E+16
cg06826870	0.02248185	0.12846018	0.02204704	0.74849131
cg18413449	0.02248471	8.84562356	1.36028631	57.5210201
cg09858237	0.02248595	12.3736116	1.42625377	107.348543
cg22607959	0.02248756	18511.6826	4.00194063	85629054.3

cg18308285	0.01238218	16.5045379	1.83410803	148.518936
cg11790979	0.0123845	16499.7853	8.17243309	33312345.7
cg18235734	0.01238528	6.59496198	1.50387378	28.9209932
cg12518535	0.01239006	13.8292183	1.76493055	108.359663
cg23736297	0.01239133	30.2276833	2.0900655	437.169475
cg05104048	0.0123919	3.4788E+10	190.409407	6.3557E+18
cg13325330	0.01239847	87881.5634	11.7186101	659051639
cg10729325	0.01241405	27817.1043	9.12526216	84796609.7
cg12046254	0.01241683	10.1355498	1.64930132	62.2865987
cg09363539	0.01241974	59.309341	2.41553651	1456.23878
cg04917391	0.01243314	7.77769538	1.55713628	38.8485878
cg19025034	0.01243533	58.511748	2.40711697	1422.2926
cg17777561	0.01243858	268.869704	3.34501336	21611.5483
cg03319638	0.01243997	15.6701901	1.81105963	135.586291
cg19901005	0.01247299	9.78708575	1.63501038	58.5849783
cg15287092	0.01248059	33.9080423	2.13663401	538.115244
cg00917251	0.01248438	270.8277	3.34259308	21943.3359
cg20138604	0.01249772	499868.245	16.8673306	1.4814E+10
cg05022059	0.01249924	7.18352345	1.52886909	33.7524054
cg06522557	0.0125059	268.938683	3.33397689	21694.2161
cg22276332	0.01250677	1223.48645	4.61908425	324072.697
cg05662500	0.01250895	1011.04802	4.43272137	230607.342
cg07023915	0.01251091	1318801.72	20.751245	8.3814E+10
cg27009812	0.01251124	12.0630328	1.70892501	85.1510507
cg13875008	0.01251137	0.10049473	0.01655839	0.60991361
cg26274889	0.01251676	1.663E+12	425.857733	6.49E+21
cg26615259	0.01251887	304.817402	3.42280235	27145.4904
cg18664900	0.01251981	4425093.61	26.8940308	7.281E+11
cg10883583	0.01252375	7159742.41	29.8105473	1.7196E+12
cg05113709	0.01252644	847.354165	4.26290749	168431.776

cg10008501	0.02248904	11.244599	1.40705571	89.862118
cg20162652	0.02249627	4.89234808	1.2510489	19.1320017
cg13144594	0.02252652	406.218665	2.33093581	70792.8563
cg21686379	0.02254062	21.0187162	1.53537411	287.738621
cg01620672	0.02254454	0.09798793	0.01331546	0.7210892
cg10043155	0.02256335	156.678612	2.0357537	12058.5253
cg11806528	0.02257411	8.11724659	1.34228544	49.0876905
cg05096838	0.02257571	6356.22684	3.42464802	11797305.7
cg07363112	0.02257872	7950.62911	3.53349801	17889497.3
cg18536827	0.02257904	0.16178872	0.03381255	0.77413834
cg03314195	0.02258072	10.6459372	1.39430743	81.2847848
cg08887028	0.02258124	0.02604417	0.00113258	0.59889689
cg07952391	0.02258548	12.8920698	1.432218	116.047601
cg02124724	0.02258963	25.6319352	1.577276	416.538452
cg15837832	0.02259076	1853.16771	2.87778332	1193359.68
cg07352798	0.02259743	122.472452	1.96438776	7635.71321
cg07205462	0.02260529	793093806	17.7543363	3.5428E+16
cg08964780	0.02263037	6.59146533	1.30268596	33.3521789
cg01610602	0.02263485	13796098.4	10.0219309	1.8992E+13
cg23280754	0.02264385	10.6780556	1.393573	81.8190882
cg01657408	0.02264552	27520.0382	4.1890101	180795101
cg25193742	0.02264792	835724.121	6.75688383	1.0337E+11
cg04054731	0.02265168	16.8391989	1.48522385	190.919787
cg17217295	0.02265625	33088.5874	4.29554007	254881714
cg02272258	0.02268662	403083.335	6.08136089	2.6717E+10
cg05166976	0.02268922	0.08643639	0.01052192	0.71006522
cg19230459	0.02270088	26510.9183	4.15225157	169264501
cg05596265	0.02270106	23.6252663	1.55582969	358.749552
cg01319727	0.02270348	2148904.87	7.67327263	6.018E+11
cg15004702	0.02270724	10289.8015	3.63640174	29116699.1

cg19431506	0.01252654	508.001339	3.81871734	67579.0685
cg09629193	0.01253153	1725.82495	4.96589719	599785.222
cg04408790	0.01253388	27704752	39.8192935	1.9276E+13
cg11556929	0.0125424	7.3684E+10	216.561263	2.5071E+19
cg20986726	0.01254677	157202.112	13.0798941	1889350450
cg13484341	0.01255219	18.8928305	1.88012422	189.84865
cg24398450	0.01255304	8.01596319	1.5638308	41.0886304
cg13255056	0.01255499	388725.12	15.8738435	9519258420
cg23123694	0.01256009	10766.3499	7.34387227	15783810.7
cg11059483	0.01256114	9.93150612	1.63722805	60.2450061
cg17715556	0.0125774	8.33258859	1.57617602	44.0509383
cg14383422	0.01257753	4.16104489	1.35794971	12.7503209
cg12353788	0.01258014	37.7602215	2.17972824	654.133986
cg08062526	0.01258253	1.5193E+11	250.689917	9.2078E+19
cg06641593	0.01258668	0.08490196	0.0122351	0.5891526
cg02272258	0.01259158	85791.9284	11.4320886	643824172
cg14265075	0.01259309	46.5478427	2.27881273	950.802859
cg02281514	0.01259694	50.4762828	2.3184469	1098.9491
cg21496187	0.01260724	10.2169912	1.64565723	63.4317447
cg25367558	0.01261635	0.0037676	4.69E-05	0.30244538
cg09490603	0.01262522	5.20379093	1.42371366	19.0202854
cg25035631	0.01262868	5.25611568	1.42669511	19.3641597
cg09125320	0.01263139	9.79731301	1.63013245	58.8831555
cg16914989	0.01263297	182.885089	3.05043717	10964.6434
cg21644713	0.01263482	14516.6918	7.78112331	27082766
cg00927495	0.01263592	7.0915268	1.52101087	33.0633747
cg21621104	0.01263682	5.37256078	1.43323211	20.1393823
cg10539808	0.01263866	15.2611457	1.79209874	129.96079
cg13696236	0.01264451	12.0085983	1.70224957	84.7152119
cg06310422	0.01264754	22.3907728	1.94488286	257.777328

cg01957751	0.02270966	0.04120924	0.00265156	0.64045433
cg19245296	0.02271591	154278.037	5.30517843	4486505572
cg00082497	0.02272602	20.9371221	1.52904583	286.69061
cg17468224	0.02272855	6745.04873	3.42392492	13287581.8
cg18795461	0.02273362	6.1966339	1.28990902	29.7682015
cg24698371	0.02275391	154902626	13.8679246	1.7302E+15
cg25543401	0.02275807	98593.6408	4.96826037	1956561307
cg26233374	0.0227598	22.5173912	1.54362903	328.468108
cg05654163	0.02277913	10.2518622	1.38287724	76.0014522
cg25397562	0.02278141	7.07E-13	2.46E-23	0.02031872
cg05045781	0.02278434	4820.96261	3.25732959	7135194.6
cg17791651	0.02278451	26.6207669	1.57926398	448.731331
cg17198397	0.02278519	0.07570927	0.00821043	0.69812307
cg12099952	0.02279301	4.77279065	1.24302281	18.3259152
cg26779912	0.0228064	1920526.67	7.48279733	4.9292E+11
cg03072378	0.02280925	103.371107	1.90624854	5605.55749
cg15204874	0.02281185	11.9134415	1.41138841	100.560616
cg16184803	0.02282023	24.0277162	1.55576922	371.09048
cg07331806	0.02283609	39.1476392	1.66439527	920.777466
cg07916003	0.02284003	2392051565	20.0748392	2.8503E+17
cg08817644	0.02284448	41.9543476	1.68014926	1047.62554
cg05390144	0.02285643	75.5509428	1.8225563	3131.83464
cg03476195	0.02286374	6.08767746	1.28480679	28.8446614
cg01907051	0.02287138	12.6248719	1.4214621	112.129188
cg19217130	0.02287149	0.15197629	0.02999405	0.77004575
cg16328610	0.02287925	5315248.66	8.55984334	3.3005E+12
cg00935430	0.02287943	8598869000	23.8428214	3.1012E+18
cg20655350	0.02288767	12.4738097	1.41872234	109.673277
cg07565021	0.02289251	116.127563	1.93251026	6978.2868
cg01711077	0.02289272	69.0604703	1.79822911	2652.24744

cg16412956	0.01265197	17.9912979	1.85572805	174.425774
cg17566394	0.01265287	0.01398124	0.00048734	0.40111017
cg19815376	0.01265749	9.76965786	1.62828852	58.6175078
cg25061763	0.01265834	19248.5081	8.24866625	44916966.2
cg06236061	0.01266101	35.0023951	2.13908741	572.752498
cg19844955	0.01266483	0.10541807	0.01797883	0.61811422
cg00454770	0.01267313	24.0896411	1.97413615	293.956831
cg07405126	0.0126902	26748.5149	8.82475451	81076821.9
cg02647835	0.01269817	4.7576646	1.39523845	16.2233003
cg11079583	0.01269984	14.1386547	1.76050583	113.547796
cg12744185	0.01270107	22.9244698	1.95181653	269.252417
cg09597767	0.01270188	0.0093401	0.00023661	0.3686905
cg06840154	0.01270237	19.3519924	1.88241571	198.946284
cg23432673	0.0127036	977994.98	19.005087	5.0327E+10
cg01240599	0.01270524	39.6839019	2.19413367	717.737524
cg04655372	0.01270815	4.7728E+10	190.269703	1.20E+19
cg08944390	0.01270916	50633539.3	44.0994945	5.8136E+13
cg13913149	0.01270921	107754.101	11.86094	978922930
cg27214030	0.01270922	464.480911	3.70881764	58170.1603
cg00108715	0.01271567	63.0189286	2.42089172	1640.46386
cg22220806	0.01271729	0.13267794	0.0270872	0.64988016
cg10059687	0.01272026	25.4417229	1.99462817	324.512243
cg18469624	0.01272571	13887.4258	7.64883665	25214369.6
cg02556718	0.01272814	31417.8683	9.10172588	108450030
cg18795461	0.01273065	5.82576719	1.45616899	23.3074345
cg07575518	0.01273698	17.1320583	1.83248898	160.168724
cg04739149	0.01274784	13920894.2	33.2894948	5.8214E+12
cg02605237	0.01275878	45008.1232	9.7985645	206737544
cg01664011	0.01276005	4674401.89	26.3386801	8.2958E+11
cg20759486	0.01276153	10.8456655	1.66144937	70.7987024
cg23032421	0.02290362	13.1838126	1.42927792	121.6089
cg18378494	0.02291262	370.294842	2.26765623	60466.9563
cg22377027	0.02291435	49553.3839	4.46592911	549838072
cg09447457	0.02291884	5.491E+13	79.7095561	3.78E+25
cg11142466	0.02293369	1075469474	17.7455676	6.5179E+16
cg25479082	0.02293638	30784.4054	4.17512764	226982190
cg00993830	0.02297012	8.12718241	1.3354808	49.4586622
cg23923856	0.022972	5.41243337	1.26256178	23.2023774
cg12889538	0.02298607	14.6743405	1.44861286	148.649976
cg27264345	0.02299398	39.6006105	1.66095691	944.15956
cg19035395	0.02299465	14.1892226	1.44170263	139.650185
cg23616741	0.02299833	12.9722985	1.42389886	118.182923
cg27611263	0.02299837	11.4194201	1.39908224	93.2062117
cg01398529	0.02299851	57623954.3	11.7526929	2.8253E+14
cg02081427	0.02300949	63827.3778	4.59459367	886679964
cg23269029	0.02302329	16.8466615	1.47549246	192.349342
cg24413842	0.02302624	0.14423846	0.02716268	0.76593076
cg25278941	0.02302695	7.04737792	1.30853596	37.9550408
cg03341758	0.02302711	73237908.8	12.1082141	4.4299E+14
cg06580065	0.02302976	48685.3901	4.42017859	536237884
cg19183317	0.02303162	12.4275752	1.41475327	109.16718
cg01879488	0.02303379	90.1469674	1.85840713	4372.81777
cg23752923	0.02304047	166.495605	2.02176289	13711.1957
cg19663209	0.0230406	7808.65679	3.4334417	17759183.4
cg14512004	0.02304545	3726723006	20.7489365	6.6936E+17
cg13760347	0.02305156	685280482	16.4232368	2.8594E+16
cg19489174	0.02305449	19.9774787	1.50965545	264.36473
cg23528791	0.02305565	12.1612944	1.41000773	104.890972
cg17895873	0.02305744	2.6867E+10	27.1802329	2.6557E+19
cg19786920	0.02306471	34.5082803	1.62715473	731.842758

cg23696808	0.01276746	5714.19042	6.30890332	5175538.53
cg20136444	0.01276945	4.8206E+14	1337.08313	1.74E+26
cg18774755	0.01277012	26.7793605	2.01364324	356.137637
cg16751098	0.01277402	14563809.1	33.4837965	6.3345E+12
cg09703123	0.01277472	85.5198627	2.57788877	2837.06845
cg25716814	0.01277525	8.44228567	1.57472251	45.2601565
cg06727269	0.01277574	798302.218	18.0424617	3.5321E+10
cg12636538	0.01277576	69934.8894	10.7450396	455176429
cg07220448	0.01278294	8.20334787	1.56490553	43.0025424
cg15085390	0.01278451	36453.1668	9.34605961	142181136
cg22896904	0.0127858	3.2719E+18	8695.6044	1.23E+33
cg16403299	0.01279261	71.4359402	2.47934674	2058.24118
cg06404175	0.01279576	2.84758551	1.24927164	6.4907767
cg03289906	0.01280072	19.5516524	1.88169477	203.150435
cg10334121	0.01280216	6.11969433	1.4698577	25.4791051
cg04171065	0.01280857	6.59551938	1.49328772	29.1309405
cg25624927	0.01281031	9765.2146	7.0470094	13531898.5
cg16613143	0.01281391	96545.8369	11.4642395	813058612
cg20785136	0.01281758	79.8234686	2.53615346	2512.38193
cg01046287	0.0128188	177.207901	3.00432166	10452.4894
cg26529833	0.01283348	8.5202729	1.57607982	46.0605164
cg23859635	0.01284227	9.91969165	1.62751772	60.4603448
cg08615333	0.01284464	7.66605779	1.54080954	38.1412761
cg03476370	0.01284631	3.41E-06	1.68E-10	0.06913392
cg12946524	0.01285076	43584.9552	9.64811367	196893236
cg13749822	0.01285126	6.95390269	1.50908154	32.0438369
cg03107888	0.01286014	8.50468489	1.57467929	45.9329501
cg06731484	0.01286213	7.04160371	1.51281901	32.7760179
cg18464137	0.01286351	12.7550707	1.71591807	94.8132851
cg23316360	0.01286711	8.06188799	1.55672272	41.7505553

cg19678561	0.02306886	8923345.93	9.02309335	8.8247E+12
cg02067430	0.02307094	4.552E+10	29.1634252	7.105E+19
cg00534700	0.02307189	6177.47535	3.31877484	11498581.1
cg07891064	0.02310485	5.59744365	1.2666089	24.7364245
cg11424962	0.02312081	247.131296	2.12876845	28689.7702
cg09375033	0.02312733	0.14227432	0.0264451	0.76543409
cg01047631	0.02313643	6.49011399	1.29211097	32.5990418
cg22710329	0.02314306	3031.50072	2.99870198	3064658.2
cg11124980	0.02315209	708.302658	2.45625957	204250.667
cg19248557	0.02315838	37.7187252	1.64366112	865.569071
cg01937803	0.02316246	27.3440669	1.57272607	475.415275
cg27317433	0.02316342	12.9770176	1.42018679	118.578053
cg20607798	0.02316538	11.1961647	1.39174382	90.0698126
cg01909364	0.02317451	9.84563092	1.36729727	70.8963958
cg00648423	0.02318098	55.4176133	1.73156127	1773.60854
cg17404605	0.02318679	3382.43268	3.03725642	3766837.33
cg01766718	0.02320104	0.13153101	0.02282524	0.75795066
cg02688226	0.02320666	145134.45	5.07017742	4154491438
cg19873719	0.02320683	64731.689	4.54071186	922804989
cg03519879	0.0232089	58.298845	1.74236348	1950.65805
cg13264159	0.02321202	890094.362	6.4924251	1.2203E+11
cg22767408	0.02322687	1597469.27	7.02284909	3.6337E+11
cg21834204	0.02322691	26.2481598	1.56188201	441.112636
cg22645427	0.02323181	5.19941031	1.25220697	21.5889771
cg03431903	0.02324038	626935.599	6.17435816	6.3658E+10
cg12527260	0.0232407	35.6749577	1.62816807	781.677659
cg24717875	0.02325401	33.7018442	1.61511018	703.242611
cg11121935	0.02326845	424.367018	2.27978684	78993.0717
cg25353054	0.02327779	910998421	16.5862565	5.0036E+16
cg02791024	0.02327919	830486116	16.3756771	4.2118E+16

cg03613618	0.01287224	9.66749294	1.61768142	57.7743048
cg09671955	0.01287891	6.04533622	1.46426808	24.958606
cg04152196	0.01287963	34.0752973	2.1124578	549.656371
cg01583595	0.01288297	0.16596348	0.04030086	0.68345638
cg06243556	0.01289009	14.2272196	1.75504726	115.332379
cg03921416	0.01290351	96.4722416	2.63125741	3537.05166
cg26333513	0.01290923	21.9484702	1.92288105	250.527897
cg24498180	0.01291033	243.428803	3.19986212	18518.7923
cg15971010	0.01291285	4.54840865	1.37796073	15.0135057
cg10498682	0.01291406	0.01353526	0.0004554	0.40229271
cg21544415	0.01291623	6.62831369	1.49219913	29.4428148
cg16754665	0.01292047	50.4108692	2.29207158	1108.7157
cg03844154	0.01293142	11.9826235	1.69083793	84.9184087
cg03875678	0.01293206	0.08446028	0.01203096	0.59293177
cg15185033	0.01293697	241.203806	3.18957491	18240.4482
cg19047868	0.01293813	7.45030416	1.5289885	36.3031063
cg08406071	0.01294515	182644.13	12.9461902	2576733203
cg12634124	0.01294804	277386795	60.8689584	1.2641E+15
cg12059147	0.01294979	12.3694811	1.70155251	89.9202714
cg03301200	0.0129533	3.45938572	1.29983877	9.20679537
cg14133339	0.01295759	23566499.8	36.097472	1.5386E+13
cg04439516	0.01296135	1089634.2	18.8470078	6.2997E+10
cg09653271	0.01296158	17690.2889	7.89281839	39649502.5
cg06912515	0.01296175	6.01183391	1.46064733	24.7439244
cg11072113	0.01296599	14.2376248	1.7522319	115.68672
cg21474786	0.01296745	10.0300642	1.6272345	61.8240259
cg17853833	0.01296829	1147437875	81.8691185	1.6082E+16
cg06561166	0.01296897	804.106215	4.10667726	157447.679
cg01885635	0.01297485	126.464694	2.77809726	5756.93269
cg12691382	0.01297574	11.3218543	1.66910911	76.7980855

cg14441271	0.02328018	12.7878305	1.41469485	115.59285
cg14400886	0.02329404	7.67152635	1.31940408	44.6052255
cg06856563	0.0233063	4.42E-08	1.96E-14	0.10002382
cg14311481	0.02331013	28.0836815	1.57361841	501.197218
cg04274259	0.02332677	13438.034	3.63719481	49648359.9
cg09655862	0.02332765	7.43387312	1.31321392	42.0818489
cg08413402	0.02333999	3792480.64	7.81798796	1.8397E+12
cg01859118	0.023346	38835.4308	4.19584629	359448508
cg18105529	0.02335475	34.5812604	1.61717535	739.476749
cg07468655	0.02335765	0.08865894	0.01091884	0.71989389
cg19326232	0.02337176	6.70E+23	1696.83605	2.64E+44
cg01157467	0.02338449	780.566425	2.46514986	247158.988
cg16478733	0.02340292	85.7604249	1.82677757	4026.13355
cg19426955	0.02340775	19.6832555	1.49667406	258.861001
cg23890469	0.0234272	47.2607337	1.68423714	1326.16536
cg01783195	0.02343404	217.732054	2.07015237	22900.366
cg26088994	0.0234611	699400.277	6.15189602	7.9514E+10
cg03004714	0.02348346	8.56632717	1.33595756	54.9283626
cg13397067	0.02348942	195737.3	5.16911237	7411928383
cg11802732	0.02350298	74053908.2	11.4893818	4.7731E+14
cg23793040	0.0235045	6.11013182	1.27614604	29.2550457
cg16124934	0.0235073	11.7703484	1.39393676	99.3883692
cg27167413	0.02351299	2431.83958	2.85752465	2069568.77
cg22974920	0.02351488	7.06E-13	2.16E-23	0.02310476
cg26755793	0.02351977	15.8361802	1.45046914	172.898959
cg24101049	0.02352765	10.0623795	1.36440727	74.2091339
cg26915924	0.0235297	0.09161545	0.01157772	0.72496062
cg09551172	0.02353588	2227808.39	7.14456794	6.9467E+11
cg05947740	0.02353722	18.0926079	1.47625134	221.738977
cg25203092	0.02354277	17431712	9.41591179	3.2271E+13

cg05586209	0.01299187	249.589131	3.20422915	19441.4105
cg00577560	0.01300158	13.6619684	1.73561847	107.540559
cg08339719	0.01300259	22227.5529	8.25322353	59863168.2
cg03087607	0.01301191	8.16325322	1.55671655	42.807217
cg08847885	0.01302223	876861.084	17.8715792	4.3023E+10
cg08432727	0.01302315	6.30685202	1.47405071	26.9844058
cg05485462	0.01303072	12.1874304	1.6932429	87.7212943
cg00846502	0.01303117	0.11053686	0.01942992	0.62884457
cg01122672	0.01303907	259.464303	3.22320417	20886.5839
cg17833106	0.01304732	9.50495234	1.60634486	56.2420444
cg18154158	0.01304999	0.07046837	0.00867832	0.5722063
cg00370179	0.01305319	15.0049716	1.76812864	127.337552
cg10634317	0.01305822	1.55E-08	1.06E-14	0.022754
cg08048656	0.01306081	106017640	48.7798777	2.3042E+14
cg26565223	0.01306133	0.11053037	0.01941667	0.62919969
cg01462607	0.01306576	7.81330855	1.54092283	39.617682
cg07571451	0.01306765	0.01055321	0.00029004	0.38398221
cg08474990	0.01307235	6110208.94	26.7211003	1.3972E+12
cg19744122	0.01307278	4.9879E+13	758.768311	3.28E+24
cg00504134	0.01308674	728.313943	3.99467589	132787.043
cg17633033	0.01308965	883297.709	17.7562965	4.394E+10
cg20248204	0.01309338	17.512558	1.82474956	168.072208
cg08947153	0.01309491	131915824	50.7933175	3.426E+14
cg04111314	0.01309569	11.7654501	1.67838802	82.4754548
cg19896881	0.01310014	17460.3352	7.77899071	39190598.9
cg07174177	0.01310664	13.4890034	1.72685832	105.366613
cg02865149	0.01310946	47378694.3	40.8714153	5.4922E+13
cg01323381	0.01312852	5.57221255	1.43383349	21.6549223
cg13340335	0.01313198	34.0810994	2.09622905	554.100391
cg03922997	0.0131349	0.00773994	0.00016605	0.36076488

cg13538006	0.02355055	81.5386259	1.80700761	3679.31352
cg10205925	0.02355624	132.646824	1.92883989	9122.15679
cg26392523	0.02355832	2.7261E+10	25.2597349	2.942E+19
cg13348059	0.02356432	5.29582058	1.25101411	22.4183846
cg21396517	0.0235769	7.40788727	1.3085115	41.9383352
cg16914989	0.02357769	346.79072	2.19307277	54838.036
cg27147545	0.0235778	98.7735317	1.85275333	5265.79034
cg03366850	0.02361327	9.52244124	1.35269362	67.0343127
cg04544093	0.02362528	258288603	13.3967708	4.9798E+15
cg01730534	0.02362567	25831.4404	3.90032493	171078904
cg14886640	0.02364362	481.111666	2.28583405	101262.134
cg16400495	0.02364383	8.51026972	1.33192797	54.3758313
cg26229990	0.02364859	27.2077659	1.55597772	475.753936
cg10938446	0.02365845	0.01212663	0.00026535	0.55419976
cg04152610	0.02366797	32.7675351	1.59451234	673.379146
cg01024168	0.02367298	7.8007943	1.31600929	46.2400928
cg18340446	0.02367649	1.0678E+13	55.1232323	2.07E+24
cg12130607	0.02368983	0.11956826	0.01898618	0.7529989
cg10052561	0.02371005	6.45275594	1.28250366	32.4662304
cg16385703	0.02371186	182056.934	5.03388865	6584318704
cg05020775	0.02372988	377.881219	2.20610103	64726.9613
cg22323778	0.02374696	730962404	15.1648906	3.5233E+16
cg15699226	0.02375962	11.5599388	1.38523204	96.4691703
cg23425533	0.0237654	8.27870452	1.32490658	51.7296461
cg20809087	0.02377443	8.45373234	1.3284427	53.796517
cg13768304	0.02377485	756.542414	2.41556191	236945.458
cg09687332	0.02377631	193360947	12.658899	2.9535E+15
cg00931235	0.02378167	2.6535E+11	33.0684759	2.13E+21
cg26139475	0.02378243	438074282	14.1033092	1.3607E+16
cg05967404	0.02378511	10.79093	1.3720789	84.8669636

cg26874634	0.01313629	15.3384548	1.77283708	132.707173
cg08463485	0.01313668	28.1243085	2.01316922	392.901262
cg26763524	0.01313699	682.532558	3.92937378	118555.963
cg07221526	0.0131452	60.3968438	2.36242022	1544.08547
cg27405799	0.01314553	11702.8512	7.12576227	19219940.5
cg22455694	0.01315036	15.9014969	1.78568943	141.602229
cg07859876	0.01315284	327842.632	14.3183972	7506482037
cg11396509	0.01316392	215.331151	3.08117089	15048.6638
cg15585294	0.01317251	19.5205127	1.86304521	204.530956
cg08717423	0.01317468	2.17E-05	4.46E-09	0.1055631
cg12794469	0.01317485	384.799126	3.47764218	42577.8041
cg16357921	0.01318313	0.01812991	0.00076088	0.4319913
cg26229990	0.01318327	18.1548579	1.83447071	179.669735
cg10428938	0.01318747	4.26833811	1.35485286	13.4470028
cg24844211	0.01318754	9.798247	1.61218052	59.5501824
cg10056728	0.01319222	26.6674096	1.98770448	357.77488
cg00242147	0.0131927	40.5408702	2.16973089	757.495854
cg02239891	0.0131971	123572872	49.2830201	3.0985E+14
cg14067419	0.01320695	18.1774987	1.83386289	180.177842
cg18682936	0.01321215	10523380.4	29.3770302	3.7697E+12
cg12752647	0.01321458	9.61190181	1.60486825	57.5677511
cg13670911	0.01321558	2861.00967	5.2783274	1550751.92
cg26969942	0.01323987	506450249	65.7109541	3.9033E+15
cg08405378	0.01324422	35945.2191	8.9354289	144599525
cg02186769	0.01324424	575.492354	3.76906575	87870.9661
cg25446604	0.01324523	0.13863059	0.02903162	0.66198312
cg06151074	0.01324886	10.3649153	1.62924653	65.9393573
cg07105272	0.013251	8.80471616	1.57463608	49.2323451
cg21480605	0.01325204	27.3438097	1.99474433	374.826949
cg11235152	0.01326379	5.28882955	1.41546598	19.7614908

cg12996903	0.02378569	6.40624185	1.28015189	32.0586447
cg26866325	0.02378714	6.81187651	1.29062388	35.9528925
cg27427051	0.02379138	98762.2956	4.61318916	2114370491
cg04828587	0.02379359	209.037091	2.03436179	21479.2204
cg06911354	0.02380777	5.08973867	1.24130729	20.8694816
cg15597540	0.02380888	341692.24	5.43331509	2.1488E+10
cg00904122	0.0238124	489147.601	5.6968849	4.1999E+10
cg00609948	0.02381497	117.469082	1.88313718	7327.65802
cg08187418	0.02382391	167.641835	1.97365324	14239.4745
cg07059148	0.02383819	7815106.81	8.21068756	7.4386E+12
cg20780850	0.02384138	93.2952925	1.82503857	4769.22063
cg06957820	0.0238416	40.8016294	1.63542188	1017.9471
cg09313482	0.02385358	8.68985993	1.33191046	56.6957523
cg25001691	0.02386004	40358.9194	4.07734114	399486410
cg24987648	0.0238685	582.942582	2.32463626	146182.893
cg21357361	0.02389049	1208.39119	2.55783922	570876.099
cg24398023	0.02389293	5036.87332	3.08927641	8212309.13
cg01554410	0.02391983	12.2746378	1.39287767	108.169394
cg22287064	0.02392771	3.39212459	1.17510404	9.79190678
cg11908659	0.02393456	4034.51885	2.99350084	5437560.63
cg27094166	0.02395297	92736.5075	4.52281909	1901482165
cg11675148	0.02398324	3.7478E+13	61.4433309	2.29E+25
cg21167563	0.02398725	98.8155893	1.83142012	5331.66617
cg02590886	0.02400002	165.727539	1.95973348	14014.9757
cg02024449	0.02401296	3.6668E+12	44.9950878	2.99E+23
cg23250439	0.0240172	32143.9276	3.91661209	263807612
cg02893231	0.02401732	9.11714727	1.33742389	62.1511061
cg20689586	0.02402084	29418.4501	3.87033442	223609930
cg19540689	0.02402285	5.47460384	1.25056106	23.9662727
cg04988673	0.02403074	2.3683E+12	42.3476825	1.32E+23

cg02366931	0.01327533	7.04517653	1.50242884	33.0361819
cg04608326	0.0132767	2258.70617	5.00349632	1019637.72
cg17956609	0.01327828	18.8714697	1.84495547	193.030333
cg01802294	0.01328746	8.67046692	1.56853224	47.9282444
cg16511225	0.01328994	0.0830171	0.01157614	0.59534863
cg05372434	0.01330149	175.999938	2.93566926	10551.5899
cg26220061	0.0133048	6267980114	109.728364	3.5804E+17
cg16540391	0.01331938	438.408919	3.54698078	54187.6012
cg00742040	0.0133411	8.80239725	1.57190199	49.292003
cg23425316	0.01334225	4.26558067	1.35205665	13.4574084
cg04091405	0.013343	1.98E-06	5.99E-11	0.06514809
cg15457058	0.01335026	4.14515031	1.34389776	12.7854005
cg03895540	0.01336371	0.13893419	0.02908716	0.66361626
cg26175971	0.01336872	18.3212982	1.82947828	183.47852
cg26389053	0.01337174	59.1290572	2.3333137	1498.4035
cg02993937	0.01337568	60267742.3	41.2600355	8.8032E+13
cg17649836	0.01338654	132898.172	11.5722975	1526224523
cg13567636	0.01338761	70082001.8	42.494644	1.1558E+14
cg19044256	0.01339321	11.3838282	1.65645342	78.2343431
cg13478617	0.01339981	1939.3408	4.80840256	782181.339
cg16197272	0.01340297	24795739.1	34.1752495	1.799E+13
cg26832893	0.01340854	1708.69682	4.68114847	623702.677
cg00473633	0.01340957	7.55853743	1.52108155	37.5597799
cg13298859	0.01341585	6.39690015	1.46920469	27.852029
cg00031256	0.0134169	1860334.51	19.9373464	1.7359E+11
cg00810836	0.01342031	960.137708	4.15089471	222088.124
cg07498606	0.01342056	1701.37816	4.67341467	619394.563
cg06530441	0.01342609	5.88449878	1.44377006	23.9839617
cg21732915	0.0134302	700.938082	3.88667029	126410.052
cg03086857	0.01343266	11.6089662	1.66182446	81.0964687

cg24981593	0.02403479	6.48513886	1.27855788	32.8941118
cg15980914	0.02407419	0.01158709	0.00024097	0.55717788
cg14103123	0.02407514	13.9225107	1.41269633	137.210171
cg14676702	0.02409011	32.6206791	1.57914427	673.851482
cg08941457	0.02409165	9.57974874	1.34477029	68.2433173
cg02986643	0.02409187	13.3868657	1.40506873	127.544062
cg05863534	0.02409275	16081.9389	3.55951392	72658448.3
cg03011535	0.02410613	4.71323462	1.22520033	18.1313863
cg22472290	0.02413788	9.49288055	1.34230706	67.1342524
cg06424168	0.02414621	12.2044893	1.38698045	107.391246
cg26807935	0.02414645	92051.5142	4.45734114	1901016996
cg23368715	0.0241489	33.9194506	1.58522924	725.780915
cg25699052	0.02415987	9.55E-05	3.06E-08	0.2983461
cg07612928	0.0241826	15.9564198	1.43556858	177.35644
cg19115492	0.02418339	28.2548111	1.54671338	516.148862
cg18070812	0.02419285	0.06488153	0.00601487	0.69986799
cg26529833	0.02421339	10.8027509	1.36369174	85.5761047
cg12792157	0.02421689	99.0516714	1.82015385	5390.33205
cg09427374	0.02422148	24087.8725	3.72330853	155836024
cg22658758	0.02422756	13.9914341	1.41012647	138.82459
cg02149965	0.02423195	21.6571806	1.49257917	314.243615
cg10449522	0.02423356	1076368.25	6.10226872	1.8986E+11
cg24838316	0.02425195	5.16953686	1.23830154	21.5812632
cg10165801	0.02426521	7.12626128	1.29090898	39.3394115
cg06501716	0.024266	50.1016907	1.66348812	1508.98547
cg05335944	0.02427254	10.052598	1.34982968	74.8647984
cg02320501	0.02428496	13.5024822	1.40232402	130.010627
cg13811955	0.02429274	15.6804602	1.42964535	171.984492
cg24893721	0.02430796	332.165038	2.1241548	51942.3597
cg08545129	0.02430921	8.55461377	1.32117646	55.3910996

cg11399508	0.01343754	8.26268102	1.54865539	44.0846286
cg23483563	0.01343827	7.73930153	1.52778777	39.204914
cg05118482	0.01343961	3072.05807	5.27542367	1788963.57
cg23735712	0.01345608	7.30541042	1.50918396	35.3628336
cg13252294	0.01346257	1.8943E+11	215.468672	1.67E+20
cg05393563	0.01346305	869717.888	16.9385509	4.4656E+10
cg12148919	0.01346735	2.43E-06	8.58E-11	0.06896808
cg01446477	0.01347614	121431920	46.9648116	3.1397E+14
cg02033116	0.01347726	133119.066	11.4709964	1544825319
cg26169020	0.01347824	106374204	45.6813617	2.477E+14
cg03513643	0.0134826	4276.3681	5.63217517	3246938.1
cg07757611	0.01349069	7.63683165	1.52219277	38.3139368
cg17279839	0.01349274	8.69024271	1.56333189	48.3072845
cg11778840	0.01349658	10.1581441	1.61445349	63.9150597
cg16323609	0.01349956	46.0141919	2.20566987	959.937784
cg05670898	0.01350035	15.7559594	1.76756228	140.447813
cg25349900	0.01350244	35.4232433	2.089455	600.542324
cg10500737	0.01350976	12.899557	1.69566368	98.1318252
cg03281983	0.01351264	327.034793	3.30533748	32357.2877
cg00105606	0.01351626	1.2359E+10	121.197224	1.2603E+18
cg13398482	0.01351999	4.05569833	1.33510668	12.3201308
cg00700324	0.01352075	6036.57433	6.03115623	6041997.29
cg02797930	0.01352612	891.743069	4.06284522	195726.309
cg20023120	0.01352964	7.85175194	1.52990417	40.2966472
cg12031217	0.01353179	92.878616	2.54692848	3386.99629
cg12179011	0.01353227	7.34281435	1.50882775	35.7343127
cg27611263	0.01353965	11.0555413	1.64149591	74.4595171
cg13776095	0.01354287	6.8871766	1.48875837	31.8609135
cg24226687	0.01354532	1.5327E+16	2175.17669	1.08E+29
cg09767313	0.01354691	0.02366316	0.0012117	0.46211551

cg27536453	0.02430928	8.04264017	1.31063778	49.3531178
cg13520520	0.02432537	20.7289166	1.4815175	290.032339
cg24041453	0.02433703	32.2503538	1.56851386	663.102413
ch.7.1039544	0.02433858	2.1144E+10	21.773366	2.0533E+19
cg04848570	0.02434573	0.07852205	0.00857276	0.71922142
cg24718465	0.02434842	20.028639	1.47430764	272.091367
cg14019833	0.02435283	5205627.65	7.40845512	3.6578E+12
cg00402417	0.02435777	23.3517371	1.50364284	362.655022
cg11260715	0.02435879	93.2931495	1.79890194	4838.29137
cg21844669	0.02435899	0.17022444	0.03644107	0.79515661
cg04876465	0.02435941	2.6553E+11	30.1208881	2.34E+21
cg10396171	0.02436028	5.46529604	1.24589453	23.9743093
cg01348757	0.02436356	50.7521053	1.66238005	1549.45085
cg19706516	0.0243692	7.80235684	1.30450744	46.6664813
cg24127861	0.02437423	6.73283226	1.27978334	35.420863
cg01910481	0.02437602	0.10325049	0.01430004	0.74549883
cg07535628	0.02438239	25.3589552	1.51904182	423.343585
cg23591609	0.02438385	16.5197596	1.43711179	189.89647
cg25424488	0.02440045	260047.211	5.00759328	1.3504E+10
cg21788755	0.02440931	21.6531345	1.48757246	315.183461
cg12905273	0.02441703	7117.78989	3.14294049	16119596.6
cg05135288	0.02441897	22.3701285	1.49357052	335.051236
cg00951770	0.02442855	22.2954652	1.49265505	333.022534
cg20643029	0.02442858	11.1077803	1.36432046	90.4353388
cg07859876	0.02443791	131572.499	4.57330471	3785298317
cg25048751	0.02445306	11.4013665	1.36842303	94.9934013
cg03613618	0.0244686	12.8721438	1.38965324	119.232683
cg05873146	0.02447143	14.6007425	1.41232289	150.94401
cg07701282	0.02447257	421.469359	2.17754494	81576.4659
cg02927346	0.02448047	11.3684523	1.36735782	94.5193023

cg10140906	0.01354963	16.2874246	1.77763886	149.231775
cg05898524	0.01355073	19.6280568	1.8472941	208.554021
cg14935646	0.01355635	10.1238392	1.61142923	63.6032405
cg25660583	0.01355661	742169.223	16.2163808	3.3967E+10
cg20917083	0.01355778	9.29520622	1.58327266	54.5710545
cg19884658	0.01356197	5.57314347	1.42476358	21.8000577
cg04710571	0.01356351	37.1354057	2.10597345	654.822288
cg23448101	0.01356508	4.351E+10	155.57054	1.2169E+19
cg17908846	0.01357273	3644316.8	22.464654	5.912E+11
cg12742785	0.01357539	33577.3349	8.55323139	131814208
cg07952391	0.01358222	11.2407898	1.64569233	76.7794514
cg20402382	0.01358438	520.192013	3.62394292	74669.9759
cg25612145	0.01358523	0.03010715	0.00186435	0.48619676
cg02392688	0.01359299	3.4504E+11	236.852548	5.03E+20
cg04579966	0.013593	3.08685923	1.26107787	7.5559964
cg24107163	0.01359783	9.26116919	1.58088017	54.2541153
cg19025234	0.01359913	9.24462701	1.58026006	54.0816863
cg00644823	0.01359975	8.59606745	1.55676933	47.465205
cg18486150	0.0136058	12.407557	1.67865175	91.7089979
cg19385386	0.01362547	244849715	52.9806958	1.1316E+15
cg07906351	0.01362556	192.564365	2.94803527	12578.2195
cg20586124	0.01363303	321.358235	3.27404959	31542.3185
cg09192572	0.01363758	20.4256689	1.85839488	224.499085
cg24333469	0.01365254	7.13785248	1.4970383	34.033156
cg21430539	0.0136795	8.15645259	1.53786714	43.259731
cg03804903	0.01368314	18126.9639	7.46639417	44008769.5
cg22680075	0.01369051	0.00326585	3.45E-05	0.30933834
cg13053408	0.01370166	45.9852639	2.19097379	965.161929
cg24361162	0.01370396	5.00082883	1.39061238	17.9836519
cg23325963	0.01370449	4.69046241	1.37246924	16.0298221

cg23994468	0.02448075	69.3919897	1.7258251	2790.11368
cg18509435	0.02448365	0.17840294	0.03973229	0.8010515
cg01280841	0.02449763	5.14069218	1.23436377	21.40918
cg10674754	0.02451614	23363744.6	8.84755961	6.1697E+13
cg03379706	0.02453395	28.30047	1.5360003	521.429978
cg12914014	0.02453985	25.5240849	1.51559609	429.849954
cg00658979	0.02454347	18.1411402	1.45051809	226.885119
cg07973470	0.02456536	20.8149946	1.47574142	293.59073
cg24494316	0.02457391	1202.76931	2.48145413	582986.404
cg26103512	0.02457949	4725.02218	2.95615562	7552320.46
cg13995374	0.02458438	12238.7628	3.3385491	44865991
cg24000528	0.02458695	22.4068386	1.48914083	337.151734
cg09524686	0.02458699	12.9486367	1.38814914	120.784711
cg04984052	0.02459384	6282.64638	3.06371231	12883600.5
cg25925006	0.02459886	5.48372548	1.24335691	24.1855294
cg15239874	0.02460302	240607117	11.817591	4.8988E+15
cg14511745	0.02460583	12041588.3	8.05325307	1.8005E+13
cg26427777	0.02461057	1871.00314	2.62160119	1335310.94
cg04320760	0.02462793	270.029446	2.04536937	35649.2587
cg01577760	0.02463687	7.14915305	1.28570246	39.7528905
cg19091677	0.02464033	756235089	13.6194719	4.1991E+16
cg03753597	0.02465147	116859.444	4.43609365	3078413307
cg01298991	0.0246732	16.811001	1.43321979	197.185217
cg17255450	0.02467827	6.17387266	1.26125754	30.2211899
cg22338443	0.02468476	15.0326617	1.41265819	159.968576
cg21464220	0.0246913	21.9571434	1.48237405	325.232451
cg24754334	0.02469253	1867512.92	6.29657144	5.5389E+11
cg04915044	0.0247007	3.65599902	1.17953845	11.3318297
cg14037250	0.02470838	964.057809	2.39859498	387479.948
cg06425443	0.02471501	2283.77645	2.67616066	1948924.42

cg00658979	0.01370458	15.0243951	1.74210018	129.574895
cg13821072	0.01370704	284.739331	3.18240642	25476.4715
cg02977954	0.013714	0.00148857	8.40E-06	0.26366421
cg00073716	0.01372345	39914.4017	8.74648923	182148451
cg25631022	0.01372929	40.2560248	2.13021725	760.742845
cg19264170	0.01374498	8.49021567	1.54873715	46.5435739
cg00901161	0.0137541	11.5109283	1.64790199	80.4061596
cg18655657	0.01376995	730.103852	3.84594495	138600.953
cg11141711	0.01377208	1.7433E+13	507.035668	5.99E+23
cg27582235	0.01377921	14.9818511	1.7381292	129.136466
cg05204123	0.01378107	25.714572	1.94077189	340.709393
cg16416806	0.01378406	36669.5482	8.550031	157269110
cg25110734	0.01379435	8.4735938	1.54674334	46.421271
cg21093033	0.01380678	7.60133015	1.51250647	38.201635
cg02436004	0.01381561	7.15492594	1.49372969	34.2719071
cg22977667	0.0138169	1936010626	78.2880607	4.7876E+16
cg02228815	0.01381885	9.02066252	1.56591184	51.964836
cg12434681	0.01382	7.14611001	1.49324594	34.1985785
cg16062483	0.01382023	16.3379658	1.76746003	151.024137
cg14960373	0.01382417	9.88325233	1.59517977	61.233648
cg03059073	0.01383802	0.13494304	0.02738534	0.66494069
cg19516235	0.01384183	230.033542	3.02760716	17477.6408
cg25826457	0.01384368	3065.33495	5.13033206	1831514.65
cg13932603	0.0138569	3195.57584	5.16937453	1975423.7
cg14182841	0.01385736	171.953405	2.85144249	10369.4792
cg17355241	0.01385888	1847.57123	4.6232084	738344.272
cg00403315	0.01386648	1644.23011	4.51264032	599093.312
cg26340700	0.01387013	103.645861	2.5709421	4178.41559
cg27278382	0.01387835	3.6276E+15	1461.34034	9.01E+27
cg13794888	0.01388372	16961.1201	7.24537022	39705299.4

cg16860686	0.02472607	17.8102518	1.44248024	219.902541
cg18796438	0.02473021	0.01169622	0.00024089	0.56789202
cg08196359	0.02473458	6.51E-07	2.59E-12	0.16340938
cg10196720	0.02475191	4.76649536	1.21947837	18.6304886
cg23044884	0.02476659	5.38861608	1.23845064	23.4463791
cg13702222	0.02476755	5.97755737	1.25485619	28.4743324
cg20666917	0.02479761	26.7555041	1.51696818	471.899813
cg17460200	0.02480593	3059237508	15.9282581	5.8757E+17
cg09565397	0.02482222	11.1899166	1.35775274	92.2216767
cg14042879	0.0248272	659.223995	2.27462595	191053.951
cg01081091	0.02483452	2900.113	2.74292182	3066312.48
cg02446170	0.02483537	679270.152	5.47130121	8.4332E+10
cg01899600	0.02484976	0.13848963	0.02462749	0.77877923
cg01164291	0.02485659	6349.76818	3.02538562	13327079.9
cg14838256	0.02486081	311657450	11.8477135	8.1982E+15
cg21997510	0.0248704	0.13359731	0.02301693	0.7754397
cg11828766	0.02487364	31017.1555	3.69324382	260492938
cg15128200	0.02490782	9.59853836	1.33007331	69.2683165
cg16763443	0.0249286	11.2134391	1.35600467	92.7291916
cg25505476	0.02492964	1.3159E+10	18.8322832	9.1951E+18
cg01555036	0.02493709	20945371.7	8.35681479	5.2497E+13
cg10386659	0.02497054	3595.70521	2.79968335	4618056.53
cg09495769	0.02497422	8.02452321	1.29928014	49.5604996
cg26149678	0.02497849	14.5920702	1.40062472	152.023958
cg02797930	0.02499238	11174.6245	3.22475594	38723002.5
cg18746774	0.0249924	0.02634733	0.00109608	0.63332929
cg08008403	0.02500478	11.6653692	1.3612345	99.9686966
cg09526712	0.02500504	12.2379507	1.36944204	109.363839
cg13598790	0.02500951	139973.653	4.42447353	4428238370
cg20651988	0.02501478	415.96503	2.13120444	81187.3808

cg05281603	0.0138981	14.6442948	1.72543533	124.290587
cg17850838	0.01390131	4560.72073	5.54021469	3754398.47
cg08021945	0.01390203	12.3782361	1.66734302	91.8951446
cg15533222	0.01390372	96838.7308	10.3057558	909951676
cg07313709	0.01390476	864.987893	3.95116017	189363.129
cg16749785	0.01390609	0.04728267	0.00415581	0.537958
cg24716879	0.013907	3.51548335	1.29098101	9.573048
cg18881778	0.01390761	5.26756147	1.40150694	19.7981208
cg16900618	0.01391986	0.01456267	0.00050053	0.42369155
cg01723761	0.01391997	5.22732499	1.3990864	19.5305497
cg03093398	0.01392431	1.3365E+12	289.533986	6.17E+21
cg04327181	0.01392672	39.6043424	2.11021556	743.290859
cg03001484	0.01392825	38.2819596	2.09562095	699.319419
cg17856005	0.01394159	137.487563	2.71507531	6962.17523
cg10802132	0.0139418	6.40749795	1.45763431	28.1662072
cg03017520	0.01394364	3.23579784	1.26896428	8.25112873
cg09503045	0.01394865	5.96554792	1.43650681	24.7738206
cg15648896	0.01394964	7.90885566	1.52102656	41.1235408
cg22063269	0.01395725	1161455.31	16.9665111	7.9508E+10
cg07232945	0.01396101	17.3348718	1.78293155	168.5414
cg13495235	0.01396512	8064.01897	6.19072132	10504172.1
cg08124910	0.01397587	7.94775003	1.52184995	41.5065431
cg25431026	0.01397815	1811765.92	18.5203878	1.7724E+11
cg12687767	0.01398055	13.6552129	1.69802829	109.812563
cg11802732	0.01398712	23473403.9	31.077304	1.773E+13
cg20955894	0.01398924	8.67720079	1.54879086	48.6145775
cg12739419	0.01399178	18.8781199	1.81265454	196.608566
cg04748923	0.01399486	575.687055	3.6201193	91548.2496
cg05889842	0.01399503	183097.858	11.6218807	2884629987
cg15769920	0.01399956	33238.5074	8.22438638	134332013

cg01770333	0.02501642	679.037929	2.26622383	203462.916
cg11056224	0.02501781	94.4762995	1.7693622	5044.62634
cg26174326	0.02503183	14.1618096	1.39417694	143.853227
cg04821708	0.02503191	9.71698556	1.32986689	70.9994429
cg03284670	0.02504349	0.13765067	0.02429233	0.77998726
cg17963935	0.02504627	53670542.8	9.29862565	3.0978E+14
cg05421550	0.02505187	955.40746	2.36195748	386460.561
cg08223837	0.02506134	78.5613738	1.72690751	3573.95485
cg00541350	0.0250686	238324.118	4.70928253	1.2061E+10
cg02918577	0.02507119	6.32918117	1.25972715	31.7993736
cg14563485	0.02507633	0.02015317	0.00066194	0.61357371
cg12595281	0.02508354	6.81829085	1.27133459	36.5671557
cg17184255	0.02508372	10.7216766	1.34537715	85.4439574
cg02631838	0.02508669	25.3352389	1.49804185	428.475564
cg06352088	0.02508785	109.65948	1.79918701	6683.68634
cg16209444	0.02511498	13.4674774	1.38361589	131.086199
cg10933774	0.02511522	26.8789745	1.50831001	478.99919
cg01791407	0.02511967	3.60405955	1.17357723	11.0680788
cg26979537	0.02515945	8.68453567	1.30909646	57.6131418
cg08141873	0.02516248	28.8944651	1.52053644	549.076033
cg04626931	0.02516451	14.1744902	1.39138545	144.400082
cg14547335	0.02516722	0.10576674	0.01479867	0.75591937
cg10583651	0.02518248	11.5552472	1.35606201	98.4643301
cg04042828	0.02519902	16.4319853	1.41641656	190.629048
cg07352001	0.02520354	1.3532E+15	76.106683	2.41E+28
cg02367316	0.02521016	5.47703873	1.23538094	24.2823506
cg12358524	0.02522807	6.2017519	1.25436512	30.6623055
cg19596493	0.02523502	1998.93014	2.56918359	1555249.58
cg24203851	0.02524313	696.496001	2.25325869	215291.161
cg02132636	0.02524451	25901.7273	3.5291093	190104478

cg02287056	0.01399992	389130211	54.760028	2.7652E+15
cg26971042	0.01400072	36.2024953	2.06750794	633.913246
cg23046727	0.01400653	10.4570962	1.60787526	68.0095431
cg16007541	0.01401031	60.6569028	2.29437854	1603.59757
cg15578015	0.01401864	3152.75626	5.09940856	1949220.57
cg06952862	0.0140196	141401493	44.4788888	4.4953E+14
cg22024145	0.0140213	34680951.2	33.4673877	3.5939E+13
cg12091786	0.01402192	7.65284549	1.50905919	38.8096402
cg03041742	0.01402508	4.8183E+10	144.466278	1.607E+19
cg03740162	0.01402856	9.05898733	1.56122548	52.5646376
cg18807466	0.01403381	28.6899198	1.97062549	417.690477
cg04622777	0.01403657	11.4141149	1.63562108	79.6529346
cg13105327	0.0140482	12.3942153	1.66267198	92.391388
cg27494100	0.01404887	0.00373448	4.31E-05	0.32333798
cg23114616	0.01405133	7.28594639	1.49341682	35.5460138
cg08513472	0.01405214	4.27157046	1.3407394	13.609143
cg08188069	0.01407036	6.32092035	1.45075083	27.5402455
cg00526088	0.01407325	3311390.7	20.679679	5.3025E+11
cg17838765	0.01408228	10.5250095	1.60759797	68.9076671
cg26915924	0.01408891	0.09051707	0.01329922	0.61607678
cg19999035	0.01409356	8.79764355	1.5501933	49.9283103
cg26861214	0.01409438	0.01095458	0.00029813	0.40252469
cg16167624	0.01409616	11.0118818	1.62187931	74.7660699
cg17453374	0.01409943	19.9539713	1.82820468	217.787963
cg05894734	0.01410124	10.1823488	1.59631873	64.9495783
cg18546689	0.01411027	6.18017379	1.44330282	26.4632948
cg10524033	0.01411185	6.16192319	1.44240878	26.3235347
cg00884529	0.0141148	34.8163477	2.04434632	592.941645
cg03375056	0.01411701	15.7139809	1.74156916	141.785467
cg15623249	0.01411904	158.341709	2.77317549	9040.93408
cg27547291	0.02524738	13.4888531	1.38102981	131.7489
cg00863378	0.02525763	16.8555086	1.41949569	200.147257
cg10397527	0.02525831	5.09983272	1.22389589	21.2504135
cg25204272	0.02526882	4141.0253	2.80758022	6107782.95
cg23520574	0.02527988	0.16116362	0.03256409	0.79761841
cg12421755	0.02528104	4.42411793	1.20226827	16.2799102
cg26300461	0.02528181	8.2280408	1.29830752	52.1453158
cg10902825	0.02528551	6.68518914	1.26528509	35.3214893
cg01012089	0.02529993	140.004348	1.84335574	10633.4426
cg26715571	0.02530033	8.12950919	1.29607006	50.9917802
cg10363578	0.02530742	1.7139E+10	18.4548737	1.5917E+19
cg17581200	0.02532339	8075.61289	3.04094005	21445843.2
cg20241658	0.02535215	1259593.21	5.66323329	2.8015E+11
cg26835312	0.02535488	16.5191501	1.41364037	193.035177
cg24436578	0.02536907	194.446949	1.9156556	19737.1677
cg08278892	0.02537651	7.58788394	1.28387081	44.8456201
cg23526147	0.02537969	18.3149818	1.43114462	234.38481
cg25923056	0.02538521	13.5778561	1.37918432	133.671891
cg17385737	0.02539519	276532.152	4.68138589	1.6335E+10
cg06898463	0.02539743	428.395004	2.10952456	86996.9864
cg05104080	0.02540831	51.4375039	1.62435204	1628.84445
cg02357321	0.02541469	17.756389	1.42483216	221.281747
cg15287024	0.02542582	2183.40727	2.57478099	1851523.42
cg07450214	0.02543498	319.047188	2.03168888	50101.7204
cg18722881	0.02543506	5.87481234	1.24322568	27.7611865
cg03318314	0.02543807	10.6130452	1.3369386	84.2497384
cg18988094	0.02544839	221385.394	4.53706817	1.0802E+10
cg21271767	0.02545355	3215.98058	2.69678635	3835131.81
cg27274426	0.02545394	0.16636297	0.03449838	0.802259
cg01663570	0.02545779	97720.7475	4.10067576	2328724598

cg09980339	0.01412302	159262312	44.8287536	5.6581E+14
cg20941453	0.01412376	22.6893793	1.87497981	274.567187
cg05369939	0.01413103	127890.128	10.6649929	1533604861
cg02277383	0.01413395	59.3233931	2.27453032	1547.24909
cg01697163	0.01415651	52.6208461	2.2186596	1248.02987
cg06347499	0.01417603	2978279253	80.0748914	1.1077E+17
cg12651953	0.01418051	0.18432077	0.04771789	0.71197924
cg02478603	0.01418607	0.18565152	0.04833575	0.71306408
cg16328610	0.0141918	4908944.14	22.0532552	1.0927E+12
cg14838256	0.01419223	80329685.1	38.6526177	1.6694E+14
cg02392575	0.01419226	45502.0808	8.61510594	240326627
cg15687395	0.01419912	6.21029468	1.44277875	26.7315831
cg08869573	0.0141999	4.45648222	1.34979133	14.7135586
cg16557944	0.01420246	7.42130771	1.49522209	36.8345335
cg14409958	0.01420544	4.11564452	1.32832015	12.7518428
cg17574799	0.0142062	693911.352	14.8652146	3.2392E+10
cg00467420	0.01420774	5.97649711	1.43152589	24.9513598
cg19778003	0.01420786	7.77294367	1.5090412	40.0377758
cg18231048	0.01421136	943.525679	3.95198529	225264.176
cg07505018	0.01421455	5.73547296	1.41961097	23.1722991
cg25996388	0.01422248	0.12782398	0.02468199	0.66197944
cg10968375	0.01422442	1.676E+11	178.107087	1.58E+20
cg05140069	0.01423817	10.9248319	1.61474574	73.9137743
cg06917617	0.01424603	4.67732098	1.36215888	16.0607782
cg26977936	0.01424912	6.0619315	1.43472614	25.6125629
cg10439144	0.01425323	3662905790	82.310809	1.63E+17
cg24425727	0.01425717	9.02687758	1.55362225	52.4480895
cg01727686	0.01425964	10.6809842	1.60678387	71.0011001
cg26279336	0.01426063	4.03700816	1.32234742	12.3246241
cg07127957	0.01426507	0.01718014	0.00066584	0.44328266

cg10282491	0.02546818	12.9186542	1.36901261	121.906566
cg24775616	0.02547171	1.20E-05	5.76E-10	0.24884327
cg14099685	0.025503	66.5701434	1.67277583	2649.23961
cg08944390	0.0255133	56203267.6	8.89682665	3.5505E+14
cg11297817	0.02552237	1.00E-13	3.92E-25	0.02561654
cg16936060	0.0255417	0.06101073	0.00524055	0.71028935
cg06199563	0.02555125	3520.02	2.71393023	4565534.01
cg07793638	0.02555845	16.7361975	1.41107813	198.50092
cg23017840	0.02557225	79.7782579	1.70720389	3728.06697
cg07048210	0.02557314	42.0145599	1.57857143	1118.24096
cg11055926	0.02557975	219926.299	4.48990034	1.0773E+10
cg18298494	0.02559614	15.3939676	1.39587739	169.76723
cg15253473	0.02560427	1935.75987	2.51657881	1488992.21
cg10616306	0.02560666	62293352.6	8.92013339	4.3502E+14
cg01973771	0.02561217	615.000465	2.18753179	172900.606
cg01875529	0.02561242	832602.317	5.26831976	1.3158E+11
cg24539573	0.02561408	28.2264668	1.50249686	530.272938
cg08576623	0.02561514	11.1302841	1.34136507	92.3560829
cg11639692	0.02562391	489610.02	4.93373202	4.8588E+10
cg15190401	0.02564255	19829.9893	3.33466224	117921531
cg04665046	0.0256465	247.905499	1.95598797	31419.9971
cg14067419	0.0256744	24.629734	1.47604301	410.979758
cg06997549	0.02569477	9.58173613	1.3156867	69.7807975
cg19311812	0.02570061	133.183164	1.81069595	9796.09814
cg16265348	0.02570357	8.69675359	1.3001509	58.1728807
cg03301200	0.02570362	3.88140317	1.17889559	12.779156
cg26863085	0.02572153	0.00214014	9.65E-06	0.47459818
cg08321129	0.02572354	19.6446121	1.43478058	268.968502
cg11861043	0.02573114	2.45E-09	6.64E-17	0.09046877
cg27602828	0.02573596	18.4166836	1.42329106	238.302793

cg21330423	0.01427716	6.2610305	1.44343208	27.1578437
cg23649435	0.01427723	6.33227532	1.44670211	27.7166325
cg10671380	0.01427944	29.5784938	1.9692082	444.283796
cg23902823	0.01428005	72.1583749	2.35380161	2212.09427
cg26522239	0.01428333	8.34039779	1.52850997	45.5098343
cg23139584	0.01428448	11.9720733	1.64308421	87.2326191
cg26296574	0.0142893	8.09906194	1.51940244	43.1714486
cg20092936	0.01429246	4.48162045	1.34977048	14.8802497
cg13239297	0.01429403	8846.23556	6.15370456	12716873.7
cg15775835	0.01430188	5.2173E+10	138.743517	1.9619E+19
cg17164093	0.01430721	20.5490614	1.82955122	230.801915
cg25815972	0.01430781	3604629348	81.2396827	1.5994E+17
cg15905666	0.01431243	0.23019187	0.0710609	0.74567445
cg11009736	0.01431294	0.13883755	0.02859778	0.67403349
cg00520042	0.01431326	40.8851591	2.09879712	796.454419
cg25591377	0.01431702	19.8416275	1.81634955	216.748028
cg16983817	0.01432073	58.3126273	2.25252015	1509.58139
cg15190760	0.01432112	11.0114282	1.61465541	75.0943823
cg13891250	0.01433027	64206.1971	9.11635626	452202133
cg09834465	0.01433164	7.04569443	1.4766459	33.6179513
cg07382273	0.0143324	15113077.3	27.115329	8.4235E+12
cg18552861	0.01433352	4.15912175	1.32912453	13.0148028
cg13835029	0.01434457	17.4106438	1.76839791	171.415334
cg10859966	0.01434466	99656.7599	9.93914472	999227808
cg24507955	0.01434556	22.8695928	1.86724793	280.101141
cg04377145	0.0143496	7.85354416	1.5085357	40.8861096
cg02897882	0.01435342	969.401919	3.94167338	238411.454
cg00169792	0.0143543	6.71046031	1.46182695	30.8041095
cg25075644	0.01436724	717930.641	14.701973	3.5058E+10
cg15841063	0.01437098	3.98913888	1.31753842	12.078

cg22597665	0.0257396	3693.96879	2.70496889	5044570.18
cg14007549	0.02575232	44.786595	1.58450503	1265.90895
cg23316360	0.02575784	9.00223541	1.30468501	62.1147953
cg02748089	0.0257657	4.59717206	1.20268197	17.5723852
cg13668129	0.02577888	50.2022768	1.60556282	1569.71036
cg08805821	0.02579273	34.751578	1.53527482	786.616282
cg09080746	0.02579391	1.64E-11	5.43E-21	0.04978414
cg25303383	0.02581192	24.2764767	1.46962172	401.019741
cg19264170	0.02583086	10.5035077	1.32791559	83.0803361
cg13037201	0.02584018	5.87458231	1.23792481	27.8778784
cg01678172	0.02584468	109.340558	1.76078016	6789.80706
cg06900571	0.02584645	0.18885006	0.04359793	0.81802834
cg09478396	0.025853	6.57232398	1.25460681	34.4294661
cg27179932	0.02585508	2754.76772	2.59640346	2922791.22
cg25924823	0.02585699	3.50E-07	7.36E-13	0.16691366
cg13866214	0.02586972	13.4068399	1.36676312	131.510246
cg22016779	0.02587247	39.1049389	1.55462894	983.640671
cg20011278	0.0258766	261.48639	1.95381522	34995.7004
cg14970695	0.02588043	8.48813961	1.29342068	55.7038518
cg09254823	0.02589362	1152.77742	2.33400056	569363.952
cg08680048	0.02590392	4.82368093	1.20814228	19.2592364
cg02133520	0.02592032	52329.2722	3.68612038	742882069
cg18884037	0.02592981	8.3330311	1.28975465	53.8392376
cg11725331	0.02593314	20909.7397	3.2991888	132522641
cg07800892	0.0259403	0.13071515	0.02180963	0.7834362
cg22484599	0.02595068	11718597.7	7.03842182	1.9511E+13
cg07287384	0.02595287	1021.37149	2.29470521	454611.652
cg27109600	0.0259552	9.65872419	1.31236299	71.0862417
cg22628500	0.02596065	8.58777491	1.29391667	56.9973938
cg01016800	0.02596631	145.78749	1.81637197	11701.3433

cg00917031	0.01437537	34.546024	2.02566686	589.153031
cg05233094	0.01438082	15368.0264	6.825136	34603887
cg10672880	0.01438898	27914.3801	7.68169493	101437589
cg16347136	0.01438904	17996.9903	7.03863766	46016243.1
cg06479512	0.01439448	245.679065	2.99207222	20172.7093
cg01501474	0.0143954	3896663593	81.2145855	1.8696E+17
cg02947424	0.0143997	307420563	48.9464569	1.9308E+15
cg13895322	0.01441165	10.4068105	1.59377139	67.9530984
cg18394533	0.01441406	13.7723098	1.68506734	112.563167
cg16379337	0.01441843	14779172.9	26.6821027	8.1862E+12
cg22956956	0.01442247	48874.647	8.5628694	278963863
cg15458254	0.01442834	7.65505331	1.49888185	39.0957041
cg07328579	0.01443053	9.26300766	1.5567363	55.1174345
cg05628567	0.01443896	369192998	50.44272	2.7021E+15
cg20969846	0.01444484	2283.78092	4.64936392	1121799.76
cg06007678	0.01444708	51.7942185	2.19084212	1224.47941
cg06439941	0.01445498	0.07666224	0.00978845	0.6004118
cg17661462	0.01445636	14.1833348	1.6934008	118.794667
cg15028904	0.01445716	52.3912826	2.19512099	1250.43061
cg16480966	0.01445752	1628810719	67.5341074	3.9284E+16
cg00661523	0.01445947	6.50952803	1.45064774	29.2103687
cg06447369	0.01446236	176281.061	11.0081676	2822905111
cg12747410	0.01446733	7.2343232	1.48119356	35.3332836
cg19251352	0.01446822	2575667.31	18.7358602	3.5408E+11
cg05703009	0.01448205	0.15034876	0.03292053	0.68664593
cg08500417	0.01448224	19.1217293	1.79580373	203.608292
cg14013195	0.01449383	6.21648142	1.43670157	26.8981687
cg02000318	0.01449385	7.20352449	1.4793069	35.077755
cg20707970	0.01449449	15.9016699	1.73081146	146.095119
cg12206225	0.01449858	2756956.52	18.9209093	4.0171E+11

cg07365623	0.02596977	1.53E-05	8.88E-10	0.26508195
cg26092468	0.02597584	1597517963	12.6478388	2.0178E+17
cg03929531	0.02597843	0.20854467	0.05246945	0.82887994
cg04458206	0.02598071	1359.44781	2.37190447	779162.212
cg06545845	0.02598183	546476.366	4.86195693	6.1423E+10
cg07207099	0.02598369	4.56048187	1.1991654	17.3437249
cg05126887	0.02598675	17.5923469	1.4093998	219.590402
cg00152259	0.02600267	55.3629736	1.6160544	1896.63098
cg13440296	0.02600845	145277611	9.45726051	2.2317E+15
cg09251737	0.0260188	79.3647204	1.68646939	3734.87884
cg20170831	0.02601925	8.801827	1.29676266	59.7427431
cg07956775	0.02602378	28.2449925	1.49047315	535.25258
cg02670545	0.02602575	0.04665855	0.00313944	0.69344309
cg22412649	0.02603897	10.613653	1.32572694	84.9719701
cg11596902	0.02604076	0.16612413	0.03419098	0.80714929
cg15687395	0.026046	6.8424658	1.25794578	37.2188841
cg10501305	0.02604622	1552.95132	2.40315234	1003539.29
cg16007619	0.02604757	13.9199006	1.3691593	141.520152
cg14089714	0.02605413	7.40058279	1.26964887	43.1368283
cg08406071	0.02606357	119103.316	4.02864902	3521180367
cg11756870	0.02607003	8.24975652	1.28595155	52.9246085
cg18909638	0.02609327	0.00014716	6.19E-08	0.34977403
cg26995224	0.02610781	7.62759796	1.2734134	45.6884234
cg11931463	0.02615468	17.1519101	1.40117956	209.957401
cg10020934	0.02620005	0.10389898	0.01411474	0.76480306
cg27180443	0.02625598	0.05543844	0.00432478	0.71065447
cg23637705	0.0262648	5.583E+13	41.9406478	7.43E+25
cg18223453	0.02627891	12.7377396	1.35005012	120.180731
cg10030624	0.02629039	43.4662668	1.55996846	1211.12471
cg03214395	0.02629469	5523.97927	2.76099762	11051928.1

cg03841667	0.01450274	151.858427	2.70669329	8519.98342
cg03469057	0.01450772	133042.257	10.3645288	1707771033
cg08932256	0.01452256	0.16697482	0.03974429	0.70149934
cg09358240	0.01452374	2.4222E+10	113.957698	5.1485E+18
cg15778054	0.01453671	7.01951933	1.47073359	33.5027717
cg10396171	0.01453763	5.20483421	1.38616103	19.5433997
cg03723902	0.01453996	6.50E-11	4.39E-19	0.00963034
cg07402729	0.01454341	5.24764971	1.38830244	19.8356112
cg21278181	0.0145532	9.25650035	1.55305528	55.1704758
cg18344652	0.01455843	8.31813104	1.52042308	45.5079279
cg05724197	0.0145587	8.09938641	1.51242364	43.3741303
cg03258142	0.01456284	467.06777	3.37178134	64699.4215
cg03743720	0.01456294	14.6856184	1.70116862	126.776022
cg26795312	0.0145633	0.1046962	0.01712628	0.64002781
cg17941202	0.01456346	608.904189	3.55319878	104346.628
cg04491089	0.01456827	10.764756	1.59966539	72.4401325
cg16697493	0.01456935	61.3894307	2.25680371	1669.91138
cg02572755	0.01457046	2.0414E+10	109.171796	3.8174E+18
cg04904385	0.01457402	6.69978372	1.45638151	30.8209777
cg05129081	0.01457657	7.39478362	1.48501061	36.8231878
cg06116248	0.01458041	1034042.27	15.4344583	6.9276E+10
cg05040724	0.01458289	0.04881287	0.00432705	0.55065144
cg13623495	0.01458576	9.13976344	1.54825316	53.9545327
cg08817644	0.014588	32.877478	1.99365649	542.183955
cg02806452	0.01459281	5.90500598	1.42009631	24.5540358
cg06927775	0.0145933	35.0511422	2.01872015	608.594793
cg05435286	0.01459489	11.7307311	1.62620806	84.6202004
cg02275713	0.01460206	10.3745211	1.58701009	67.819788
cg21365903	0.01461231	5296233.57	21.2291954	1.3213E+12
cg10020934	0.01461908	0.10836693	0.01820529	0.64505362

cg07502066	0.0263191	50775.9866	3.58046788	720073716
cg23418097	0.0263293	285619.865	4.38446893	1.8606E+10
cg09230763	0.02632951	22.9362311	1.44568786	363.889545
cg16983817	0.02633334	102.057762	1.72309459	6044.8143
cg08655788	0.02635643	47.9046377	1.57558659	1456.50789
cg01639898	0.02635699	17.7430935	1.4020235	224.544999
cg23437162	0.02635845	61.9747672	1.62390953	2365.20059
cg13722431	0.02635974	4.54569977	1.19468806	17.2960517
cg23733260	0.02637071	9.87018522	1.30842472	74.4563706
cg02447380	0.02637272	30.9193647	1.49609053	639.003521
cg24965497	0.02638446	8.65E-05	2.24E-08	0.33361932
cg23732368	0.02638791	2346.33886	2.48539036	2215066.96
cg15516684	0.02639363	3945464.88	5.93737684	2.6218E+12
cg26009944	0.02639584	43.0015026	1.55437156	1189.6314
cg21943938	0.02639858	74.6815888	1.65820188	3363.48655
cg26149645	0.02639885	497.827582	2.07125054	119653.464
cg14832904	0.02640101	8.29962576	1.28158682	53.7488266
cg25014411	0.02640359	41.3207963	1.54685997	1103.78977
cg25362068	0.02640428	12881.8865	3.03224734	54726076.1
cg12829717	0.02642999	37.8198286	1.53002972	934.844217
cg07684809	0.02643812	4.15951045	1.18151793	14.6434741
cg27115340	0.02644396	1136.77095	2.27756216	567382.179
cg19872537	0.02646477	556875.331	4.69323343	6.6076E+10
cg04726784	0.02648125	6.85527544	1.25204679	37.5343812
cg21080336	0.02648987	13.5040849	1.35501642	134.581624
cg14889011	0.02649436	64721.6597	3.64254821	1149989786
cg16793187	0.0265082	15.6936622	1.37858407	178.65507
cg13344237	0.02652091	2096.04235	2.43815388	1801934.47
cg23975712	0.02652405	789677.34	4.86580023	1.2816E+11
cg16098981	0.02653307	8.51258419	1.28327299	56.4681795

cg02692405	0.01463066	1673.10991	4.32178021	647718.449
cg00376785	0.01463119	167926071	41.8712469	6.7347E+14
cg27316993	0.01463958	5481418729	83.1295775	3.6144E+17
cg21384402	0.01464266	4.12995223	1.32251701	12.8970027
cg14398359	0.01464675	0.01356109	0.00042919	0.42849108
cg11204680	0.01464948	0.02481803	0.00127595	0.48272676
cg00689612	0.0146526	22.1759555	1.84145717	267.056444
cg04782146	0.01465301	0.01406652	0.00045836	0.43168563
cg14632140	0.01465399	8.91589861	1.53882021	51.6585677
cg12156831	0.01465779	1989.08287	4.46428111	886245.863
cg23460835	0.01465889	4.49053521	1.34425137	15.0008451
cg02746725	0.01465944	7.15113844	1.47325857	34.7113413
cg22450798	0.01466043	213.529359	2.87603806	15853.3323
cg19629053	0.01466611	18.4036577	1.77447138	190.870713
cg04481181	0.01466785	6.23960748	1.43403957	27.1489729
cg05928186	0.01466947	29.2139509	1.94332612	439.172263
cg08553950	0.01468531	0.08454352	0.01162143	0.61503672
cg22698604	0.01469427	776905.58	14.4050162	4.1901E+10
cg11514288	0.01470145	7.85482835	1.49969477	41.1405904
cg09230763	0.01470794	16.0096847	1.72482066	148.600959
cg21661347	0.01470795	490.908822	3.38038631	71291.1037
cg03562744	0.01470864	15638.3259	6.67456004	36640203.1
cg06295928	0.01471543	0.12854926	0.02472944	0.66822843
ch.7.1039544	0.01472282	6262067027	84.0522669	4.6654E+17
cg10608717	0.01472292	10582693.9	23.9863852	4.669E+12
cg08336736	0.01472785	2085821.01	17.4242717	2.4969E+11
cg24818238	0.01474529	13.6788603	1.67097051	111.97757
cg17765480	0.0147491	4060.19868	5.10654194	3228253.78
cg13677279	0.01475705	1.5064E+15	949.478202	2.39E+27
cg23433889	0.01475871	8.5866841	1.52467644	48.3585512

cg06787764	0.0265387	6.36190509	1.24040322	32.6295802
cg27621129	0.02655576	13.3502741	1.35185567	131.840863
cg11597832	0.02655877	15.8265074	1.37881361	181.662216
cg00547414	0.02657868	57.1221518	1.60006298	2039.25737
cg22878324	0.02658307	2.1252E+10	15.8401197	2.8514E+19
cg23352722	0.02658356	6.85560985	1.25061319	37.5810736
cg18480675	0.02658742	23.1823429	1.44065109	373.040374
cg19274820	0.02661233	236413.951	4.20101172	1.3304E+10
cg17610800	0.02661491	8.53764027	1.28239711	56.8398829
cg23500601	0.02661891	14535811.5	6.76983907	3.121E+13
cg24869272	0.02662145	11.4703129	1.32695401	99.150444
cg05740582	0.02662175	0.18368655	0.041066	0.82162258
cg24096828	0.02662542	110.81108	1.72591148	7114.55685
cg01256539	0.02665474	0.06774584	0.00626751	0.73226786
cg12636538	0.02666559	122766.595	3.87935496	3885088394
cg22492252	0.02667816	8975788.33	6.36644426	1.2655E+13
cg10334121	0.02668711	6.46312972	1.24067807	33.6687227
cg27559724	0.02675677	231413.518	4.14725446	1.2913E+10
cg02953545	0.02677275	9.86890364	1.30138902	74.8394659
cg13212159	0.02677433	6378.16648	2.74004908	14846817.1
cg06236987	0.02677582	62.9613362	1.61057584	2461.31214
cg26640895	0.02677904	1.0753E+17	91.0196517	1.27E+32
cg01404317	0.02679409	22.0563487	1.42701673	340.908771
cg20263444	0.02679596	112471.291	3.80648463	3323221403
cg27616378	0.02681148	60895.0632	3.54378703	1046397169
cg04614943	0.02682339	3610704.95	5.65755138	2.3044E+12
cg14516246	0.02682476	71131764.5	7.96407055	6.3532E+14
cg04604946	0.02682711	24.8976549	1.4461382	428.654207
cg25369015	0.02682854	125.702513	1.74133849	9074.12425
cg27505984	0.026834	7.3514E+13	38.9598417	1.39E+26

cg08101193	0.01476835	0.12039306	0.02195225	0.66027345
cg22708290	0.01477747	1559.53953	4.22522152	575629.829
cg12623536	0.0147794	12.2298967	1.63352378	91.5630219
cg06104975	0.01478841	14.5157046	1.68898522	124.752827
cg21745320	0.01479225	2473896.99	17.8807282	3.4228E+11
cg07117012	0.0147927	14.2087861	1.68177383	120.045632
cg22673001	0.01479981	1424.39763	4.14539005	489437.322
cg09339476	0.01480298	385.506227	3.20884029	46314.256
cg10810752	0.01480396	5.35394065	1.38889506	20.6384783
cg06998765	0.01480849	13.3107221	1.65985494	106.74145
cg09952946	0.01480961	14.0694511	1.6779258	117.972711
cg09524686	0.01481455	10.6279868	1.58812486	71.1241964
cg07098902	0.01482516	6.39905766	1.43778677	28.4798411
cg22588983	0.01482687	6.82517894	1.45599547	31.9939647
cg24129356	0.01482976	6.72864437	1.4518797	31.1834755
cg18782736	0.01483189	14.3392598	1.68337169	122.144368
cg23180925	0.01483235	11.9067549	1.62325653	87.3372816
cg11875676	0.01483684	2005356013	65.8935823	6.103E+16
cg03596993	0.01484151	40205.942	7.94505579	203462104
cg06649280	0.01485048	9.38015405	1.548774	56.8109291
cg06892501	0.01485461	0.1417727	0.02944094	0.6827056
cg23725690	0.01485988	137.144765	2.61506989	7192.42216
cg11897145	0.01486356	826.182201	3.71316596	183826.157
cg02965178	0.01486595	14.5552064	1.6870615	125.575761
cg09285418	0.01487114	16.3136564	1.72485987	154.293917
cg04762213	0.01487537	157.274601	2.68428474	9214.85709
cg20912226	0.0148774	677468.77	13.7468369	3.3387E+10
cg17610800	0.01487905	6.59646904	1.44517563	30.1094226
cg09508736	0.01488174	0.05649216	0.00559175	0.57072747
cg18294691	0.01488268	5.44798237	1.3921424	21.3200258

cg03895540	0.02684162	0.1401426	0.02460367	0.79825296
cg25518276	0.02686374	16.1059733	1.37481868	188.681154
cg17530152	0.02692688	10.8503638	1.31285816	89.6748769
cg13250618	0.02692843	5527314.42	5.88490649	5.1915E+12
cg07117012	0.02694573	16.2206741	1.37411329	191.476402
cg16553500	0.02695842	9.90653822	1.29874318	75.564978
cg09676684	0.02696035	5.79159489	1.22163478	27.4571188
cg03912887	0.02696201	131950.258	3.83318291	4542144494
cg06649280	0.02697367	9.69971676	1.29536321	72.6317566
cg14172427	0.02699086	0.09152346	0.0109962	0.76176726
cg26523099	0.02700841	9.73864692	1.29536075	73.2160861
cg20704635	0.02700965	937.984077	2.17727161	404090.204
cg11481534	0.02701669	44.972628	1.54119748	1312.31545
cg00102996	0.02702701	8085705.1	6.09049474	1.0735E+13
cg02595823	0.02704854	8.01686061	1.26640837	50.7498652
cg00364457	0.02705581	29.3741814	1.46724447	588.070053
cg07830449	0.02706527	16.4686435	1.37382538	197.416806
cg13062913	0.02706888	22.9645823	1.42650643	369.694821
cg16155498	0.02708179	3.4238E+11	20.2560776	5.79E+21
cg08089542	0.02710551	168.444429	1.78604908	15886.1959
cg24548682	0.02711203	1145.89881	2.21812293	591979.851
cg23264429	0.02712152	0.19352923	0.04509434	0.83056009
cg25706502	0.02712258	0.12101902	0.01859428	0.78764009
cg06963233	0.02712478	7.68934437	1.25929555	46.9516604
cg07447260	0.02712701	7.94E-13	1.47E-23	0.04290499
cg27313566	0.02712748	0.16939013	0.03506866	0.8181955
cg27372920	0.02713432	4.45308252	1.1838022	16.751062
cg20118643	0.02713898	869.872678	2.14778533	352306.38
cg18469624	0.02714661	15638.4058	2.97522455	82198749.3
cg01458759	0.02715301	7794307337	13.0738908	4.6468E+18

cg07472647	0.01488664	5996.37141	5.4597406	6585746.97	cg22416074	0.02715968	6596.56074	2.69717057	16133430.4
cg04308089	0.01489795	0.10979937	0.01854891	0.64995205	cg10554436	0.02716951	12.6968294	1.33186443	121.040455
cg04184179	0.01490406	0.02638919	0.00141467	0.49226256	cg13983182	0.02717151	18.7285847	1.39149422	252.074267
cg15851312	0.01491073	702328013	53.0269648	9.3021E+15	cg25418309	0.0271723	7.9322E+11	21.9615419	2.87E+22
cg01850783	0.01491904	6.51510793	1.4408116	29.4602232	cg12661343	0.02717342	507.603009	2.01851366	127648.784
cg26631984	0.01492114	5.31124814	1.38453947	20.3745415	cg13763617	0.02717703	305.534558	1.90600446	48977.5172
cg07535628	0.01492413	18.6067239	1.76752688	195.872651	cg27035169	0.02718245	7.5440093	1.2557369	45.3216564
cg08815403	0.01492643	24.3762623	1.86291539	318.963581	cg23332610	0.0271839	0.07968377	0.00844379	0.75197341
cg01932843	0.01492813	8.85E-05	4.82E-08	0.16236314	cg06813515	0.02719204	28.6298225	1.45911995	561.754182
cg13337238	0.01493008	12.1697156	1.62700992	91.0270888	cg04574507	0.02719298	0.18990172	0.04348284	0.82935398
cg07395004	0.01493849	14285.7172	6.44182685	31680720.7	cg14638883	0.02721368	5.47179296	1.21073246	24.7292605
cg10391522	0.01493991	10.7259094	1.58717949	72.4840092	cg08718097	0.02724052	0.23687354	0.06596452	0.85059472
cg27565794	0.01494048	2.06E-13	1.25E-23	0.00338807	cg08267698	0.02725243	45565316.5	7.24392244	2.8661E+14
cg03327649	0.01494197	34.4278139	1.99164387	595.123651	cg02086523	0.02725383	3.2349E+13	32.8766402	3.18E+25
cg13811955	0.01494323	12.5551185	1.63648406	96.3229668	cg13169065	0.02725961	14488.7969	2.93131389	71614724
cg00648423	0.01494528	33.8248239	1.98461868	576.49297	cg26577320	0.02726333	10.6049573	1.30343085	86.2839168
cg02247068	0.01496102	19.1350132	1.77564407	206.206152	cg01476047	0.02726595	144.205195	1.74686604	11904.2547
cg20729316	0.01496538	136469860	38.2151272	4.8735E+14	cg13878456	0.02727042	162.69072	1.77043663	14950.1372
cg02064106	0.01496688	7.41335902	1.47640106	37.2242295	cg04795713	0.0272752	4051.79049	2.53876354	6466536.13
cg07338077	0.01496711	559522.722	13.1174829	2.3866E+10	cg15689069	0.027278	7911.82712	2.73624777	22876951.8
cg03429689	0.0149764	41.5102057	2.06339563	835.078429	cg26433777	0.02728035	17746.8556	2.99531115	105147969
cg18497587	0.01498602	14.3110113	1.67719186	122.111876	cg27020690	0.02728083	1135.50427	2.20070201	585890.295
cg23008404	0.01498716	7.61463028	1.48362385	39.081735	cg05471775	0.02728239	1.25E-06	7.14E-12	0.21779395
cg10811426	0.01499275	12.0790945	1.62261244	89.9195151	cg04129227	0.02728569	119.528228	1.70953089	8357.26184
cg23655939	0.01499446	78.5093734	2.33407239	2640.75859	cg03317826	0.02729067	1767118429	10.8722517	2.8722E+17
cg03122532	0.0150003	9.84264341	1.55911842	62.1361586	cg23487303	0.02730934	19389374.5	6.54510611	5.744E+13
cg11596902	0.01500066	0.170721	0.04108418	0.70941327	cg11802553	0.02731717	22.9045252	1.41969693	369.527653
cg22120345	0.01500845	37.8510783	2.02482148	707.57059	cg00058923	0.02731872	318.705635	1.9060953	53288.6694
cg20809087	0.01502114	6.63191492	1.44357486	30.4676236	cg26593267	0.02731907	6.27019418	1.22805832	32.0142248
cg09686443	0.01503829	6.35547446	1.43133428	28.2198619	cg08872550	0.02732157	1214.86593	2.21374168	666698.94

cg01063425	0.01505227	0.00676308	0.00012045	0.37973304	cg04377288	0.02733474	7.32477624	1.24938415	42.9430348
cg01181415	0.0150535	5.00408728	1.36622893	18.3284726	cg02519806	0.02734101	7.54658713	1.25346721	45.4347564
cg16974909	0.0150579	16.0791507	1.71285909	150.940079	cg04351402	0.02734111	107.708595	1.68717169	6876.08828
cg20791007	0.01505827	12.5698	1.63304845	96.7514902	cg23187101	0.02734415	20681314.6	6.57032173	6.5098E+13
cg16860848	0.01505889	212217382	41.0491458	1.0971E+15	cg02077702	0.02734918	27.0520185	1.44550864	506.265878
cg15831653	0.01506064	4820.83935	5.17069952	4494651.45	cg01316923	0.02736576	154.537142	1.75538892	13604.8074
cg02576345	0.01506303	14801.9645	6.42476063	34102150.4	cg24201890	0.02736632	15.4763831	1.35770454	176.414256
cg08680048	0.01506743	4.42177602	1.33364317	14.6606705	cg15084269	0.02736875	14.0544373	1.3431261	147.065274
cg25250853	0.01508193	11606.462	6.12045909	22009780.5	cg05368724	0.02737224	15.6506094	1.35927507	180.200152
cg09004287	0.01508595	9.39806032	1.54281937	57.2481391	cg00251738	0.02738083	4.4539E+10	15.4117517	1.29E+20
cg02712555	0.01509125	409.551094	3.20226733	52379.1679	cg15177964	0.02738184	866.807439	2.12669	353297.913
cg05875017	0.0150997	10.9694411	1.58926407	75.7134329	cg07233502	0.02742378	142878.25	3.74762662	5447232674
cg19048532	0.01510247	14.9006152	1.68616206	131.676745	cg00735304	0.0274364	106452094	7.81363959	1.4503E+15
cg10824810	0.01511858	5.46969326	1.38876375	21.5425729	cg13443371	0.02743991	2014.89784	2.33057137	1741981.97
cg18975124	0.01511907	35250828.8	28.7498423	4.3222E+13	cg23123694	0.02745761	6951.62297	2.67229008	18083763.5
cg13886725	0.01512794	52.5403887	2.14979442	1284.07275	cg06139836	0.02747659	9.26105207	1.28025826	66.992019
cg18882819	0.01512932	9.64057404	1.54923207	59.9914433	cg20941855	0.02752122	10.7126934	1.30032862	88.2559983
cg09096430	0.0151381	1.5626E+11	145.121895	1.68E+20	cg03991512	0.02752612	15.8447921	1.35782623	184.896589
cg06160809	0.01514505	6.14102288	1.41964483	26.5645049	cg07195622	0.02755591	97786323.9	7.64310992	1.2511E+15
cg15372508	0.01515088	8.52673433	1.51236308	48.0739045	cg08104023	0.02756313	0.11387745	0.01648697	0.78656524
cg18318639	0.01515778	479021.968	12.4763721	1.8392E+10	cg05598363	0.0275648	44.2303822	1.51998462	1287.07007
cg19217130	0.01515832	0.17271632	0.04186283	0.71258746	cg11939825	0.02757322	747.78899	2.07678388	269256.893
cg07454803	0.01516007	29.570349	1.92216865	454.905735	cg16044810	0.02757432	54.8885553	1.55633859	1935.79567
cg09731694	0.01516304	4.7341392	1.34979279	16.6040848	cg14826425	0.02757968	5.03849084	1.19547029	21.2354837
cg21028142	0.01516314	20.3232066	1.78787072	231.019347	cg07557790	0.02760317	29.5975659	1.45290977	602.938961
cg19291355	0.01516567	1382.10505	4.03464831	473452.512	cg02117713	0.02760711	5352.5934	2.5767541	11118738.9
cg09844094	0.01516596	12.986765	1.6397972	102.851782	cg17558126	0.02760824	9.73836819	1.28520937	73.790168
cg26796245	0.01516949	5.84641997	1.4057518	24.3148375	cg13052543	0.02761035	630.595344	2.03525891	195380.786
cg24129382	0.01517151	5.4747778	1.38801951	21.5942152	cg23655939	0.0276227	66.8396768	1.58871658	2812.04492
cg05169244	0.01517244	942102.427	14.1929715	6.2535E+10	cg01729401	0.02763794	2146.46709	2.32666133	1980228.45

cg13301722	0.01518724	103718.515	9.26172366	1161504149
cg07488684	0.01518866	41.7295499	2.05250971	848.40297
cg18727742	0.0151916	13.9896446	1.66260241	117.713144
cg06485940	0.01519536	9.30046229	1.53671986	56.2878121
cg06361057	0.01520278	70.6540869	2.27065402	2198.48553
cg13050390	0.01520521	7.25206221	1.46457487	35.9096742
cg13303069	0.01520735	5.76381281	1.40116108	23.7100063
cg20976433	0.01520779	1.9274E+11	148.963911	2.49E+20
cg10626541	0.01520885	1.0054E+12	204.699649	4.94E+21
cg13440296	0.01521064	109175245	35.291641	3.3774E+14
cg20116935	0.015212	22.3497469	1.81876401	274.643209
cg12898849	0.01521416	2.66E+12	246.579387	2.87E+22
cg09308608	0.01521506	1726045687	60.005616	4.9649E+16
cg16674492	0.01523164	3.49168864	1.27193932	9.58527608
cg00501272	0.01523182	5.16753844	1.37156657	19.4693091
cg01913455	0.01523696	16.3726876	1.71204438	156.575906
cg04014328	0.01524561	7.06425293	1.45628061	34.2678939
cg14870223	0.0152465	10.7674299	1.57917745	73.4164153
cg04202511	0.01524767	7.43570012	1.47065149	37.5953356
cg00034101	0.01525382	17.7290713	1.73778662	180.873743
cg26405020	0.01525819	12.5433067	1.62583262	96.7716731
cg02595219	0.01525884	7.52996168	1.47395408	38.4681744
cg05471775	0.01526372	1.54E-06	3.12E-11	0.0764691
cg17799946	0.01526446	6.71541244	1.44176011	31.2789652
cg24189904	0.01527295	6.2491878	1.4217921	27.4669892
cg01786994	0.01528475	8.91355045	1.52183088	52.2077601
cg12602851	0.01528809	30.5576566	1.92768477	484.399933
cg00984602	0.01529672	677499.334	13.1436599	3.4922E+10
cg12586496	0.01529922	1975.16451	4.28773697	909868.043
cg12904795	0.01530644	6796018301	76.852469	6.0097E+17

cg16818414	0.0276417	10.1265973	1.29018752	79.4829987
cg12019705	0.02766087	0.08137151	0.00872428	0.75895374
cg05590982	0.0276613	6.46832197	1.22782911	34.0757431
cg12910906	0.02767137	17.0982344	1.36608866	214.004866
cg03656968	0.02767546	6.95202234	1.23740808	39.0579433
cg22665276	0.02767655	91.885305	1.64311026	5138.3705
cg13216025	0.0277132	17343807.8	6.21896058	4.8369E+13
cg00661320	0.0277139	14.6483498	1.34219417	159.868191
cg03403116	0.02771553	620690.905	4.31586933	8.9265E+10
cg00479347	0.02772628	2079.50494	2.30962432	1872313.51
cg19605920	0.02772881	11961.4629	2.7972723	51148611.9
cg07286413	0.02773538	1.9543E+10	13.3975442	2.85E+19
cg10553515	0.02773865	37317791	6.74687323	2.0641E+14
cg21084516	0.0277508	14.514938	1.34009205	157.215636
cg20650138	0.02779262	23.0082767	1.40832361	375.894284
cg22871962	0.0277974	60684075.8	7.07313769	5.2064E+14
cg16451324	0.02781397	16.9603785	1.36172913	211.24204
cg01306563	0.02781894	43.7994593	1.51002212	1270.44008
cg03616164	0.02781947	1981206.14	4.8594692	8.0774E+11
cg05666074	0.02782043	1.3846E+11	16.3960173	1.17E+21
cg05928362	0.02782429	8.38720056	1.26090299	55.789489
cg11214576	0.02782718	36.9957181	1.48222869	923.395401
cg13415434	0.02784308	12705.3086	2.79851363	57682358.9
cg05208572	0.02784338	21.2796883	1.39512853	324.575925
cg04692420	0.02785354	1255.6516	2.17412465	725193.446
cg22450798	0.0278592	175.344523	1.75450806	17523.8305
cg00415665	0.02787336	70599.9042	3.36667741	1480494228
cg10418044	0.02787381	0.00358278	2.37E-05	0.54210217
cg04444465	0.02788394	2205.79772	2.30854348	2107624.85
cg25983070	0.02789058	62638.114	3.31956031	1181943678

cg07570677	0.01531056	84665039.5	33.1237218	2.1641E+14
cg21902772	0.01531267	12.5894437	1.62520508	97.5225189
cg00798281	0.01531271	14.1611428	1.66227873	120.640397
cg12977686	0.01532719	270844561	41.292196	1.7765E+15
cg22685442	0.01533094	0.0005082	1.10E-06	0.23383578
cg18529840	0.0153326	0.17497734	0.04275533	0.71609948
cg16559243	0.01533565	8.54687274	1.50830657	48.4311578
cg14893163	0.0153358	12.1358672	1.61307543	91.3034012
cg19007249	0.01533966	9.23979432	1.53089007	55.7674263
cg06630413	0.01535133	0.16574426	0.0387524	0.70888933
cg20971407	0.01535283	29.8883838	1.91617635	466.196906
cg01447951	0.01536187	20.8961345	1.78890866	244.086489
cg17463145	0.01536207	9.79843418	1.54757415	62.0385862
cg25401594	0.01536313	0.19795992	0.05342494	0.73351751
cg08252387	0.01536501	18.9940344	1.75640837	205.404021
cg22332276	0.01537439	23.3210857	1.82632069	297.797119
cg25357589	0.01537566	41308.5441	7.63404502	223524464
cg14483317	0.01538033	10308469	21.9239985	4.847E+12
cg23127201	0.01538803	252871.014	10.7821217	5930534967
cg06283270	0.01538848	412.720406	3.1618719	53872.5599
cg09439599	0.01539069	176674791	37.686227	8.2826E+14
cg18679410	0.01539169	8.51867352	1.50592527	48.1881804
cg02705837	0.01540297	5.43231334	1.3816489	21.3585581
cg03723506	0.01540721	0.0001092	6.81E-08	0.17512592
cg20454158	0.01541068	80.4649759	2.3115092	2801.03249
cg27485194	0.0154166	651051.069	12.8795229	3.291E+10
cg19878627	0.01541783	11.1281765	1.58404205	78.1774148
cg06123544	0.01541815	13.7328828	1.64892375	114.372827
cg24238564	0.01542057	4166.67591	4.90857082	3536913.03
cg10911160	0.0154347	17.4377438	1.72520587	176.254275

cg01731920	0.02794434	32.3934788	1.4575331	719.940747
cg11251349	0.02795123	4012594461	10.9623233	1.4688E+18
cg20138604	0.02795124	652071.21	4.26184841	9.9768E+10
cg23574449	0.02796248	1007.0691	2.11345276	479872.65
cg26389053	0.02796313	65.8475082	1.57324568	2756.01858
cg14921691	0.02796371	52.7449381	1.53590094	1811.33328
cg11078326	0.0279717	0.12056034	0.0182723	0.79545516
cg26572233	0.02797503	0.05695091	0.00442174	0.73351419
cg22424532	0.02797557	8.32300518	1.25754812	55.0853
cg13522186	0.02797755	1723.84721	2.23860733	1327454.42
cg12223253	0.02800041	0.00404327	2.96E-05	0.55146239
cg17152135	0.02800363	1451.35044	2.19496882	959657.408
cg19351701	0.02801556	14.8934887	1.33840671	165.73139
cg03871460	0.02802049	10.3457258	1.2867171	83.1838194
cg09758869	0.02802053	22488.9021	2.94805136	171554242
cg01181415	0.02802101	4.86171075	1.1860309	19.9288496
cg14196353	0.0280326	596843966	8.83543918	4.0317E+16
cg15717808	0.02804436	61.2910701	1.5581194	2410.98035
cg23173307	0.02805484	10.9051664	1.29344543	91.9425369
cg06468809	0.02806621	8.496537	1.25896873	57.3414885
cg07196758	0.02806729	317588906	8.22260167	1.2267E+16
cg00811132	0.02808994	9.09255878	1.26781194	65.2104801
cg17297071	0.02809667	3.93590238	1.15862284	13.3704662
cg18169610	0.02809767	168.483231	1.7347941	16363.0941
cg18832407	0.02809775	9.03012744	1.26674972	64.3719912
cg09155362	0.02809893	29.2339988	1.43715015	594.667638
cg23112423	0.02812602	0.17346958	0.03631376	0.82865822
cg09919392	0.02813903	0.08073512	0.00853703	0.76351615
cg23254528	0.02815315	48.0101462	1.51402588	1522.41396
cg19966212	0.02815594	5.24840041	1.19434624	23.0634182

cg04354805	0.01544283	37768.8325	7.46265915	191149653	cg21095811	0.02815935	111902.18	3.47328656	3605259101
cg05975928	0.01544334	0.17423645	0.04236386	0.71660946	cg11733478	0.02816131	4846733.33	5.19936936	4.518E+12
cg13662121	0.01544551	12.9664469	1.630046	103.143559	cg03760060	0.02817823	0.02133334	0.00068691	0.66254866
cg13386351	0.01544659	6.77684473	1.44031875	31.8857368	cg24294159	0.02818016	22.125802	1.39278049	351.491938
cg04759982	0.01544659	54.4944289	2.14330495	1385.54375	cg20001810	0.02818961	7.47630075	1.24000572	45.0764638
cg01233487	0.01544787	0.14655485	0.03097543	0.6933987	cg08949408	0.0282011	5.86743743	1.208149	28.49551
cg09303936	0.0154522	12.4638077	1.61758849	96.03586	cg11830546	0.02820442	46014.0481	3.14929628	672306585
cg00225623	0.01545352	3.31933287	1.25696875	8.76550888	cg15860013	0.02823814	607159.965	4.13829009	8.9081E+10
cg22165175	0.01545406	12.1640856	1.61004106	91.9013693	cg22831315	0.028239	44496.2962	3.13145779	632267943
cg01860897	0.0154556	5.3671099	1.37750672	20.9115994	cg22960067	0.02824395	460681593	8.38906996	2.5298E+16
cg13811713	0.01545899	65.5017388	2.21892314	1933.58558	cg01718071	0.02824994	526704.712	4.07243327	6.8121E+10
cg04640314	0.01545968	109897193	34.0730709	3.5446E+14	cg14188840	0.02825094	22.863823	1.39592523	374.485961
cg11615303	0.01546133	79072427.5	31.9936358	1.9543E+14	cg03418154	0.02825485	13577.468	2.75674532	66871478.9
cg01960885	0.01546306	9.34348613	1.53083702	57.028104	cg19595886	0.02826327	0.16503489	0.03299818	0.82539439
cg03071500	0.01546589	0.07181704	0.00851871	0.60545428	cg13152070	0.02826594	36916.9751	3.0647903	444683949
cg05547853	0.01546821	19.2993648	1.75754396	211.923849	cg19477674	0.02827678	37.5253162	1.47084142	957.376737
cg08193467	0.01547517	18.0293408	1.73462476	187.393341	cg14159026	0.02831158	3.7828494	1.15182736	12.4236931
cg17200441	0.01547579	16.1614146	1.69884207	153.746676	cg16857771	0.02831706	9.5942151	1.27144636	72.3970483
cg04086834	0.01547646	7.69649254	1.47498794	40.1603268	cg08916340	0.0283436	1123.58824	2.10641322	599336.599
cg13692196	0.01548916	4995012625	70.1541574	3.5565E+17	cg06445533	0.02834615	228310.171	3.70033653	1.4087E+10
cg12244052	0.01548969	11.0002803	1.57841075	76.6632937	cg08358115	0.02835338	680711639	8.6362466	5.3654E+16
cg00994394	0.01549182	2373.80134	4.38958361	1283705.54	cg09159285	0.02836569	5.05685619	1.18731195	21.5375533
cg19856705	0.01549963	9.68895677	1.54045973	60.9401734	cg11579905	0.02837224	30.1652328	1.43441625	634.363473
cg01287707	0.01550536	7.58195274	1.47010626	39.1033009	cg11331678	0.0283848	6.1256058	1.21143694	30.9739989
cg08741214	0.01551389	154.973113	2.60901173	9205.27313	cg09598437	0.02839839	14.5369979	1.32718278	159.227734
cg21553700	0.01551915	4.33662309	1.32168426	14.2290412	cg20633478	0.02840038	0.17860541	0.03827299	0.8334832
cg02441296	0.01552059	8023.82368	5.52345401	11656066.3	cg01250407	0.02840401	11.6333589	1.29617491	104.411093
cg10771262	0.01552657	11.3528485	1.58680186	81.2244883	cg04575016	0.02840928	211777.003	3.65493646	1.2271E+10
cg10575841	0.01552853	89212881.4	32.4232568	2.4547E+14	cg07007550	0.02841437	2916.10263	2.32320478	3660312.1
cg03374922	0.01553562	510.492147	3.26934818	79710.7611	cg03355690	0.02841922	7.58810016	1.23871061	46.4832251

cg12678667	0.01553568	12.578274	1.61772422	97.7997204	cg15269548	0.02841982	186267.754	3.60308937	9629424316
cg20997028	0.01554089	152.256139	2.59748693	8924.75404	cg02642822	0.02842302	6.92952668	1.22683683	39.1399563
cg04517429	0.01554864	13.6322835	1.64220948	113.164097	cg04273871	0.02842972	18.7799597	1.3629091	258.775061
cg14563485	0.01555514	0.02849008	0.00159488	0.50893285	cg18307767	0.02844177	62715910.7	6.64748019	5.917E+14
cg08790676	0.01555581	5.33926738	1.37433064	20.7430259	cg09838956	0.02844819	0.09570023	0.01173025	0.7807623
cg04397817	0.0155573	3605.02751	4.7329745	2745889.15	cg03911494	0.02846674	5.8135E+11	17.3596708	1.95E+22
cg20762044	0.01556158	57.7640253	2.1593339	1545.23699	cg17046776	0.02847038	9.85168155	1.27250425	76.2713594
cg25682080	0.01556725	11.3899485	1.58655543	81.7689212	cg23046727	0.02848879	12.4421748	1.30384482	118.731702
cg07666840	0.01556817	45.6563046	2.06465409	1009.61132	cg24944820	0.02848999	11.4606201	1.29259638	101.613942
cg09024340	0.01557287	914.641855	3.64511978	229504.042	cg05851813	0.02851124	7037273.39	5.24502945	9.4419E+12
cg18010762	0.01558131	18880.7831	6.4688124	55108101.5	cg13166888	0.02855146	9.27965818	1.26322436	68.1684575
cg21464220	0.01558168	18.7025045	1.74251132	200.735382	cg11955344	0.02855833	6806424.98	5.20490205	8.9007E+12
cg23044884	0.01558199	4.8133039	1.34710715	17.1982566	cg07154254	0.02858763	614.868135	1.95855607	193031.402
cg08872550	0.01558741	629.079157	3.39281856	116640.657	cg14186963	0.02858793	13.1440243	1.30951096	131.931217
cg13375538	0.01558897	295.360591	2.93956698	29677.1189	cg15433343	0.02862187	262258.465	3.68299332	1.8675E+10
cg04381393	0.01558975	0.00032357	4.80E-07	0.21799501	cg09677945	0.02862409	5.87113226	1.20313358	28.6503465
cg08606356	0.0155959	367.407505	3.06276686	44073.9634	cg14059420	0.02862819	12.662052	1.30365007	122.983585
cg10268345	0.01559918	9.03127034	1.51738844	53.752778	cg00700007	0.02863972	3.83841965	1.15074533	12.8034109
cg17272843	0.01560014	15.1851575	1.67435796	137.717868	cg05779272	0.02864659	44.5891706	1.48627109	1337.70626
cg10857203	0.01560278	3815.64194	4.77023806	3052074.8	cg19770550	0.02865504	919808.31	4.18824366	2.0201E+11
cg06086785	0.0156037	28844.3979	6.99748066	118899834	cg16713947	0.02866384	19.032264	1.35953848	266.43385
cg15844687	0.01560662	1.661E+11	133.475169	2.07E+20	cg15631337	0.02866427	133824936	7.03398031	2.5461E+15
cg23494957	0.01561814	1659.09027	4.07032165	676256.267	cg27665327	0.02866823	25.3600849	1.40072578	459.143336
cg08885449	0.01562021	16527.5363	6.28896632	43434714.6	cg01993865	0.02867205	2.5638E+13	24.9635218	2.63E+25
cg09912793	0.01562722	9.80821109	1.54053196	62.4466142	cg22168205	0.02867244	11.1618784	1.2858188	96.8935355
cg09179916	0.01564036	15.29476	1.67520492	139.642429	cg04745805	0.02868306	7.572177	1.23471571	46.4381105
cg14054928	0.01564608	6.21257693	1.41260167	27.3227145	cg15140562	0.02868513	1179.02044	2.08857304	665568.866
cg14109663	0.0156592	1458.68822	3.96305025	536902.433	cg00174845	0.02869309	0.17812054	0.03796825	0.83561726
cg19824362	0.01566059	1269522440	52.5540682	3.0667E+16	cg23433889	0.028695	8.53664484	1.25004885	58.297166
cg02909446	0.01567108	6.16441377	1.41002395	26.9498948	cg04401646	0.02871228	45942.0324	3.05339637	691253307

cg00521598	0.01567129	255521.202	10.5090454	6212846401
cg15954782	0.01567324	151766227	35.1116426	6.5599E+14
cg26382173	0.01567359	8.62768867	1.50241895	49.5447771
cg23275158	0.01567539	35.106158	1.95840318	629.309809
cg00957901	0.01567674	6.36143405	1.41831373	28.5323637
cg19675664	0.015679	18.6546596	1.73779643	200.251491
cg08325845	0.01568045	5.80743917	1.39404352	24.1931828
cg04006061	0.01568302	7.34645283	1.45726627	37.0353519
cg14886269	0.01568522	391.839757	3.08748339	49729.3024
cg24494316	0.01569625	786.856754	3.51985686	175900.207
cg20818283	0.0156968	414129.978	11.4832432	1.4935E+10
cg20278503	0.01569682	42.7824843	2.03166361	900.907491
cg21404224	0.01569836	4.65E-12	2.97E-21	0.0072678
cg05247640	0.01570086	0.00019653	1.93E-07	0.19980837
cg00319168	0.01570741	8.23507535	1.48843142	45.5623719
cg05178734	0.01570757	12.6400718	1.61372452	99.0078617
cg25143652	0.01571774	29.6171974	1.89439026	463.039956
cg17655624	0.0157199	19.9216048	1.75782016	225.774142
cg12938320	0.01572274	6.76519473	1.43391217	31.9181752
cg00777652	0.01572409	281555.228	10.6483475	7444661887
cg04828587	0.01572478	197.957244	2.70961705	14462.2172
cg01904183	0.01572707	25.2236359	1.83746215	346.255734
cg19403104	0.01573348	3.97745692	1.29713791	12.1962078
cg05725263	0.0157336	15.9950637	1.68605185	151.740329
cg21328770	0.01573641	434487.326	11.5414186	1.6357E+10
cg04152610	0.01574019	21.2255983	1.77810122	253.374789
cg25119415	0.01574191	0.17076864	0.04068239	0.71681944
cg05789837	0.01574537	335.407175	2.98999626	37624.7871
cg24913115	0.01574844	1.4556E+12	195.218364	1.09E+22
cg18429434	0.01575091	12589416.1	21.7230669	7.2961E+12

cg27111463	0.02871241	10.2433599	1.27369807	82.3793529
cg13932603	0.02872987	5631.13412	2.45257314	12929144.1
cg16884841	0.02873142	28.2951119	1.41512559	565.754279
cg26227186	0.02873487	30.2815654	1.42504029	643.471776
cg10859966	0.02873765	51815.4465	3.08705627	869708959
cg05709162	0.02874291	14.2092477	1.31718811	153.283132
cg12628956	0.02876657	21988.4989	2.81957905	171477400
cg27625456	0.02876895	10.501629	1.27604755	86.4264124
cg26960176	0.02878231	176613164	7.14982995	4.3627E+15
cg00897115	0.0287902	31.7880915	1.43070685	706.282182
cg04329454	0.02879623	12.9216667	1.30326553	128.116233
cg17504394	0.0288014	3.71488733	1.14545677	12.0479342
cg26127778	0.02880355	6.7249026	1.21797971	37.1305981
cg27093637	0.02880664	0.15100281	0.02772718	0.82236461
cg23084986	0.02880893	27.4473187	1.40863064	534.813941
cg09778958	0.02882141	3335.98821	2.31309033	4811233.36
cg07112260	0.02885167	44.7779156	1.48043379	1354.37447
cg06618097	0.02885927	16.9781049	1.3392808	215.231971
cg26841114	0.02886276	32.4075994	1.43154239	733.650995
cg03562744	0.02886985	9442.02223	2.5693312	34698439.8
cg02882968	0.028877	0.07416991	0.00719262	0.76483603
cg25850845	0.02888139	10.6732434	1.2762875	89.2574168
cg24591969	0.0288928	0.17818495	0.03792095	0.83726472
cg17071957	0.02889692	13.0634446	1.30284822	130.985008
cg01507044	0.02891781	2881.24925	2.26853717	3659449.53
cg17471836	0.02891887	8.20866085	1.24167649	54.2670443
cg27398547	0.02895916	13.7658228	1.30869559	144.799049
cg08410301	0.02895946	46517853.5	6.11883243	3.5365E+14
cg05662500	0.02896182	1004.95814	2.032213	496966.048
cg13686012	0.02896287	54.0158714	1.50562567	1937.87502

cg23656386	0.01575206	28.3453175	1.87709934	428.031167
cg01396817	0.01575239	118657.368	9.02452713	1560145013
cg01217876	0.0157532	53.7101484	2.11707551	1362.62501
cg16663533	0.01575521	23823296.3	24.4812039	2.3183E+13
cg02803139	0.01575566	34.3610793	1.9461776	606.668049
cg23302730	0.01575787	4.84781627	1.3460162	17.4599106
cg14240353	0.01576687	8.26278144	1.48792091	45.8852056
cg00761126	0.0157675	48.1533437	2.07309529	1118.49393
cg22381196	0.01577159	0.12191977	0.02208474	0.67306332
cg18160072	0.01577277	11.415699	1.58105368	82.4248945
cg20705565	0.01577856	251805790	38.0280224	1.6674E+15
cg10196720	0.01578275	4.07070518	1.30211433	12.7259491
cg05228295	0.01579075	32381220.1	25.8138842	4.0619E+13
cg00564831	0.01579189	251.706925	2.82696807	22411.4226
cg21942490	0.0158117	13.7007419	1.63497333	114.809413
cg03881924	0.01581398	118254.326	8.96849986	1559244682
cg03509901	0.0158171	510.757751	3.22523319	80885.153
cg01614478	0.01582126	8503.15933	5.46749119	13224295.4
cg21282452	0.01582345	18.4543115	1.72858743	197.017291
cg22213149	0.01583307	2457.62269	4.3277977	1395608.05
cg20650138	0.01584184	19.1078284	1.73918627	209.930997
cg03131236	0.01584645	0.00134127	6.22E-06	0.28923125
cg26150582	0.01584804	0.12868098	0.02432398	0.68076005
cg26097051	0.01584895	6.62131306	1.42546978	30.7560268
cg14780632	0.01585621	3.78975318	1.28374522	11.1877567
cg05421550	0.0158581	433.424165	3.12128844	60185.5644
cg21318213	0.01586842	0.03553297	0.00235969	0.53506586
cg03840075	0.01587623	0.0626596	0.00659677	0.59517384
cg06305609	0.01588135	9.06052609	1.51099251	54.3306021
cg20706778	0.01588278	6.28010518	1.41071704	27.957216
cg17188384	0.02896617	10.6049068	1.27401513	88.2752851
cg20585530	0.02897435	6.28098166	1.20728035	32.6773568
cg20922251	0.02897501	12192.755	2.62339193	56668343.2
cg00283344	0.0289778	0.00629346	6.66E-05	0.59484009
cg17106536	0.02898013	3908267.68	4.73751403	3.2242E+12
cg02311432	0.02900192	21.0937985	1.3662775	325.664687
cg16013543	0.02900265	11.5095878	1.28411678	103.160875
cg23511157	0.02902288	13.3500943	1.30338118	136.740518
cg25246158	0.02902519	901.232478	2.00479263	405139.148
cg00334820	0.02903289	2.2323E+12	18.2753928	2.73E+23
cg06422678	0.02903934	6.29946495	1.20683726	32.8820296
cg09963640	0.0290535	14.4297797	1.31318665	158.559747
cg13873511	0.02906118	24981.1688	2.80982274	222098990
cg12849291	0.0290645	1422101.22	4.24283576	4.7666E+11
cg09337254	0.02906608	35.1327683	1.43768403	858.541504
cg09728459	0.02907028	95984917.5	6.51630339	1.4139E+15
cg16629179	0.02907163	15.574892	1.32309486	183.340793
cg15042692	0.02907556	22515037.7	5.61780461	9.0236E+13
cg11204680	0.02909806	0.02092421	0.00064907	0.67453599
cg04556868	0.029113	50.0037551	1.48886133	1679.38778
cg02425595	0.02912975	171.203362	1.68665392	17377.9522
cg23343073	0.02914587	115.352111	1.61960786	8215.63655
cg12006072	0.02916552	10.6408618	1.27110842	89.0781128
cg06277849	0.02916996	30.777594	1.41559329	669.161333
cg20277282	0.02917587	5.43271375	1.18719495	24.8605999
cg08826738	0.02919427	37.3378829	1.44290033	966.191126
cg10481400	0.02919477	1362189181	8.41671284	2.2046E+17
cg21649520	0.02921732	35.9244058	1.43661063	898.338703
cg07254054	0.02921968	12.4415426	1.29044702	119.952217
cg16119852	0.02924445	29509.6174	2.82809898	307916209

cg01993576	0.01588293	12.6237647	1.60777343	99.1180933
cg27180671	0.01588442	5.64274162	1.38269515	23.0278764
cg03051983	0.01588701	60.2483115	2.15418205	1685.02891
cg01633093	0.01588933	9.75076046	1.53169713	62.0731917
cg21204356	0.01589319	458916617	41.8250265	5.0354E+15
cg26781150	0.01589444	54.6318589	2.11458379	1411.45507
cg25785679	0.01589695	333010234	39.3655465	2.8171E+15
cg02786912	0.01589715	6.41107514	1.41588828	29.029045
cg24335302	0.01590029	271470449	37.8695622	1.9461E+15
cg27087622	0.01590209	7.16842142	1.44568088	35.5446809
cg09904140	0.01590502	18.6958277	1.72961035	202.088276
cg10884788	0.01590577	8.19711349	1.48232746	45.3291673
cg13897296	0.01590591	37378581	26.1104626	5.351E+13
cg01834022	0.01590848	4.10281435	1.3022476	12.926179
cg13524209	0.01591197	23.2857573	1.80183234	300.930604
cg13074532	0.01591917	5.74309099	1.38661555	23.786762
cg21400015	0.01592701	13.3357915	1.62294639	109.580535
cg18254850	0.01592933	59.0486447	2.14322332	1626.86847
cg27625401	0.01593448	30.6346404	1.89556097	495.094174
cg01083650	0.01594067	35837863.5	25.7849016	4.981E+13
cg17297071	0.01594451	3.90936844	1.29004471	11.8470014
cg19697239	0.01596458	8.84556819	1.50210598	52.0895846
cg18257541	0.01596545	42.2046217	2.01072475	885.864709
cg07530530	0.01596629	122380.861	8.90273795	1682299886
cg02774334	0.01596762	8.05425733	1.47598826	43.950933
cg25298754	0.01596947	19.8417502	1.74637079	225.436119
cg14019186	0.01597068	4.09274383	1.30076846	12.8774279
cg16098170	0.01597387	159.32937	2.57565235	9856.08489
cg16007619	0.01599642	10.8598742	1.55982571	75.609004
cg21741562	0.01599691	4.93967669	1.3467955	18.11738
cg19601328	0.02924826	13.2074719	1.29773536	134.416707
cg26438284	0.02925034	95.3273829	1.58434098	5735.70338
cg16509443	0.02925854	114164.152	3.23926175	4023587645
cg03520966	0.02925918	1589.42536	2.1041819	1200596.28
cg17802464	0.02926574	11.8301569	1.2830742	109.076008
cg11904590	0.02927771	16251.1551	2.65795085	99362275
cg14340070	0.02927863	124.038795	1.62579036	9463.47269
cg08539350	0.0292793	245.672562	1.74171779	34652.5759
cg20053110	0.02929089	4.80113296	1.17123495	19.6808315
cg19637634	0.02929234	306.799834	1.78043341	52866.9806
cg07408835	0.02929631	0.02797694	0.00112211	0.69753346
cg13430298	0.02929723	10504772.4	5.09499099	2.1659E+13
cg02491878	0.02929739	19.1388811	1.34618348	272.100183
cg13982823	0.02931195	8469.96155	2.48465601	28873312.2
cg12091786	0.02933881	8.15970257	1.23482819	53.9190366
cg01024069	0.02934546	14061.0574	2.61003536	75751209.6
cg20207890	0.02934581	50.13584	1.48172516	1696.40263
cg16802592	0.02935472	5.91089911	1.19527771	29.2306364
cg25512381	0.02936262	33.7882147	1.42367438	801.899276
cg18032014	0.02936763	6.65190104	1.20937011	36.5874656
cg25984973	0.02937033	150.782229	1.65387585	13746.6671
cg13567541	0.02937072	6.34837935	1.20367882	33.4822874
cg12397426	0.02938578	7.8913638	1.2300232	50.6280066
cg10929210	0.02938743	11.9562831	1.28229425	111.481982
cg17878425	0.02940393	678.043817	1.92072732	239359.024
cg17055959	0.0294097	47738.0406	2.93966807	775230561
cg03100449	0.0294111	33.6694074	1.42182493	797.305608
cg00484396	0.02942783	27.2793291	1.39176308	534.689997
cg10911160	0.02943711	20.9255235	1.35513654	323.124292
cg21212076	0.02943807	17.6068586	1.33193112	232.745873

cg02060584	0.01601243	5.10089208	1.35461795	19.2077036
cg11235663	0.01602744	9.56542359	1.52253536	60.0953719
cg24324441	0.01603783	18.803241	1.72626964	204.812657
cg12467096	0.01604984	43399320.1	26.3313983	7.1531E+13
cg11517045	0.01605356	523.906982	3.20382401	85672.1608
cg06623120	0.01605671	1762.54796	4.01392425	773949.664
cg15227217	0.01606418	30672.5568	6.82312457	137884884
cg17365504	0.0160682	1.1233E+14	408.531919	3.09E+25
cg09486260	0.01607123	3073950.21	16.0531985	5.8862E+11
cg22544144	0.01607342	600211498	42.7622739	8.4246E+15
cg03519879	0.01607528	50.5330004	2.07257335	1232.08384
cg19359627	0.01607699	37.3893832	1.95966912	713.368374
cg18372896	0.01607704	5.44348867	1.3699662	21.6294159
cg04559178	0.01607853	17.2578617	1.69742622	175.461994
cg20470734	0.01609077	0.17993379	0.04451748	0.7272686
cg07665387	0.01609168	6.64268387	1.42128421	31.0460419
cg22940789	0.01609194	145.728905	2.52168997	8421.69895
cg01917164	0.01609844	2545.54007	4.28706036	1511472.6
cg02105002	0.01610091	44.7138461	2.02447985	987.576161
cg26188299	0.01610297	4218.83507	4.70716593	3781164.64
cg04385193	0.01610355	5817727.56	18.0038571	1.8799E+12
cg08141873	0.01610514	27.9580013	1.85531208	421.303696
cg08623154	0.01610649	7.5689E+11	160.021129	3.58E+21
cg22838882	0.01610846	9.96305482	1.53192881	64.7957404
cg09295382	0.01611655	19596.3938	6.25302505	61413259.8
cg24527881	0.01611957	50.8851607	2.07247848	1249.37344
cg03109066	0.0161213	2.7444E+12	202.565456	3.72E+22
cg23864823	0.0161243	32075953	24.6468031	4.1744E+13
cg03945021	0.01612556	10.1763014	1.53749189	67.3545726
cg00311035	0.0161276	12.0092946	1.58537397	90.9710641

cg21938523	0.02944315	1344155.13	4.09493579	4.4122E+11
cg22837289	0.0294445	2253670.05	4.3114354	1.178E+12
cg22381196	0.02945279	0.1190258	0.01752179	0.80854409
cg23772500	0.02945456	6.2062E+10	11.9549234	3.22E+20
cg05342320	0.0294649	8.5013E+17	61.5229273	1.17E+34
cg03719380	0.02950055	102.002749	1.58498839	6564.43974
cg00145141	0.02950282	9.28003711	1.24836197	68.9856717
cg17507671	0.02950752	9.64453313	1.25308613	74.2303478
cg13415207	0.02951141	6.95E-08	2.49E-14	0.19391789
cg16823292	0.02951313	0.17982292	0.03835693	0.84303624
cg08360738	0.02951321	0.01101661	0.00019008	0.63849298
cg13959647	0.02951757	14.3355769	1.30332203	157.680727
cg19280776	0.02951798	38.1010986	1.436428	1010.62755
cg02851730	0.0295223	3375.27849	2.24356709	5077853.45
cg08432727	0.02952772	6.66212143	1.20752151	36.7561669
cg13327503	0.02952978	15.3909505	1.31232289	180.505392
cg14397459	0.02954895	1.0943E+11	12.4843278	9.59E+20
cg26164735	0.02956142	209491047	6.69656437	6.5536E+15
cg14266050	0.02956713	447.261223	1.83221672	109180.644
cg14159304	0.029568	16.7311741	1.32247352	211.673188
cg15107861	0.02957306	23.2230327	1.36607826	394.786494
cg00003202	0.02957514	4.951E+14	28.6609779	8.55E+27
cg14798020	0.02959112	0.09141579	0.01059223	0.78896018
cg03784679	0.02961281	2130850434	8.37876259	5.4191E+17
cg04520793	0.0296138	332.379672	1.77637373	62192.0065
cg26121931	0.02961827	3.32223689	1.12612472	9.80109731
cg12619165	0.02962241	0.22232188	0.05735275	0.86180734
cg04522003	0.02962654	21.3832132	1.35371358	337.768501
cg20239584	0.02964599	706.379542	1.91172849	261005.713
cg19048426	0.02964748	149942.286	3.24512916	6928133807

cg16101252	0.0161281	228412.796	9.84991896	5296734483
cg20601684	0.01613575	3566.0036	4.55320315	2792843.03
cg23008153	0.01613862	15.9942573	1.67147984	153.047773
cg21187108	0.01614552	70.9228566	2.20223099	2284.07085
cg02091066	0.01614583	7.69064646	1.45923353	40.532267
cg02839622	0.01615053	74.2027073	2.22038464	2479.76934
cg09519400	0.01615792	474.802105	3.13023182	72019.2789
cg21163429	0.01615867	6.55420465	1.41638118	30.3291226
cg05843387	0.01616037	2858163.72	15.67733	5.2108E+11
cg17516247	0.01616571	11.7717398	1.57837564	87.7952337
cg16745793	0.01616917	0.03406455	0.00216887	0.5350215
cg09755397	0.0161735	32.2483732	1.90165441	546.86991
cg07509646	0.01617666	3.6856E+10	90.1473261	1.5068E+19
cg14413354	0.01618083	17.4050117	1.69629956	178.585458
cg02740658	0.01618719	622209.217	11.7894245	3.2838E+10
cg22637507	0.01618964	24.2723467	1.80354703	326.660079
cg07569020	0.01619262	17.3584499	1.6950226	177.765053
cg16364495	0.01619802	6.74904616	1.42326558	32.0036013
cg16579893	0.01620439	20.2584252	1.74366522	235.368457
cg06892005	0.0162154	9994916415	70.329262	1.4204E+18
cg23953820	0.01621598	65.228794	2.16347023	1966.65316
cg04604143	0.01622106	167389756	33.0150866	8.4868E+14
cg18568748	0.01622376	0.00010155	5.63E-08	0.18307006
cg11856688	0.01622418	7.6149E+15	856.165901	6.77E+28
cg10424417	0.01622439	6.67169484	1.41969257	31.3529231
cg02490920	0.01622642	24.8237922	1.8094196	340.562609
cg17465827	0.01622731	444.515333	3.08226848	64106.6417
cg01865606	0.01622897	106626.81	8.47645731	1341276929
cg03451607	0.01624009	16.3500011	1.67466996	159.626996
cg24533564	0.01624652	13.1289672	1.60801858	107.193898

cg08790487	0.02966702	312.855852	1.76278167	55525.1883
cg08404201	0.02967471	1.9241E+10	10.3332832	3.5829E+19
cg07237214	0.02970543	27.5684005	1.38614905	548.29364
cg26822986	0.02974051	6.40976429	1.20026781	34.2299259
cg05375405	0.02974162	2152360.29	4.19002627	1.1056E+12
cg27195224	0.02975565	0.14093037	0.02407435	0.82500116
cg23149053	0.02976856	20.4633305	1.3446529	311.417091
cg26906273	0.02978405	8.65832013	1.23561904	60.671214
cg24265806	0.02978784	3489195.16	4.3768611	2.7816E+12
cg09456081	0.02979991	1.2553E+16	37.7178556	4.18E+30
cg14663177	0.02980835	7.69461994	1.22107629	48.4876962
cg12738347	0.02981281	5.9163598	1.19001426	29.4141964
cg21211321	0.02981565	0.2049157	0.0490352	0.85633262
cg03330710	0.02983074	21.856134	1.35193674	353.337979
cg23176538	0.02983487	6468.48029	2.35752632	17747940.6
cg09651522	0.02986007	10.3303919	1.25596044	84.9684383
cg06909469	0.02987254	8.42899886	1.23109203	57.7113814
cg20885078	0.02987461	24.1187293	1.36396947	426.485426
cg04789663	0.02990642	12.9796421	1.28341862	131.267465
cg00229387	0.02991289	102.755704	1.56950818	6727.4162
cg06677890	0.02991953	7.62854474	1.21852897	47.7581546
cg20346912	0.02992034	6.27517149	1.19558977	32.93586
cg06244417	0.02994012	0.12733463	0.01980862	0.81853779
cg19818312	0.02994041	12407.1424	2.49876191	61605382
cg11832404	0.02994183	5.2565287	1.17493713	23.517083
cg10983208	0.02994615	9.34141987	1.24236951	70.238463
cg16117513	0.02995452	15.7229863	1.30664526	189.196185
cg01527459	0.02998849	4.29919787	1.15178112	16.0474087
cg06824394	0.02999172	4.45553839	1.15574357	17.1766669
cg22324029	0.03000108	22.8375664	1.3537793	385.258097

cg11643442	0.01624771	8.43331709	1.48190267	47.9929207
cg02605634	0.0162649	24.8560214	1.80814491	341.688211
cg08535309	0.01626796	588264.483	11.5724564	2.9903E+10
cg06241689	0.01627043	16.6277248	1.67878962	164.690816
cg02588777	0.01627523	2.4749E+11	125.714164	4.87E+20
cg00133403	0.01627759	5.92378086	1.38786032	25.2843742
cg17826834	0.01628001	0.18324572	0.04590233	0.73153141
cg18634560	0.01628197	9.26336886	1.50691173	56.9442793
cg04903089	0.01628971	0.09601057	0.01419202	0.64952184
cg23509869	0.01629627	67.1858209	2.16971295	2080.42936
cg17556421	0.01629935	0.13046094	0.0247617	0.68735402
cg02268171	0.01631741	11.119661	1.55746119	79.3900114
cg14473050	0.01632083	57.2697533	2.10525892	1557.91984
cg19788741	0.01633209	8.92307735	1.49530786	53.247436
cg21774561	0.01633416	1225.0895	3.69522731	406157.501
cg25703213	0.01633773	0.07637357	0.00935802	0.62330747
cg15593337	0.01633844	11.6513538	1.57028167	86.4520347
cg16635010	0.01634474	155.762595	2.52827094	9596.27609
cg04134731	0.01635058	3430.24996	4.46058806	2637906.62
cg21143446	0.01635194	14.93	1.6430353	135.666531
cg06784539	0.0163525	13.112617	1.60430996	107.174255
cg14470398	0.01635356	11.5409793	1.56709634	84.9942658
cg05533953	0.01635714	5078.97689	4.79199568	5383144.73
cg13382703	0.01635959	87728.8295	8.08444445	951994608
cg23345004	0.01636461	6.74083783	1.41949942	32.0105061
cg06459921	0.01636523	7.56E-07	7.60E-12	0.07520797
cg19712821	0.01636676	12.337555	1.58602429	95.9728453
cg26251270	0.01636686	22.2433605	1.76724144	279.965756
cg09487931	0.01637797	248977.831	9.77414066	6342241464
cg19136717	0.01638246	13555.3868	5.7281183	32078337.3

cg19402405	0.03000598	267447949	6.54213261	1.0933E+16
cg19169234	0.03000828	152247.08	3.17381882	7303243994
cg12623536	0.03001583	12.3266882	1.2750583	119.168858
cg09612202	0.03004111	1560.91379	2.03464502	1197482.54
cg02721176	0.03004198	0.21080478	0.05165027	0.86037611
cg08302771	0.0300427	20.1780721	1.33672899	304.590234
cg00351047	0.03007815	21.7043752	1.34537138	350.148598
cg04517429	0.03008382	16.1579695	1.30752498	199.674946
cg21942576	0.03009702	374.867923	1.76951167	79415.1075
cg11848563	0.03009846	15.3809071	1.30104366	181.832717
cg01784928	0.03010183	26.4954584	1.37090635	512.076785
cg02152119	0.03011604	26261.2413	2.66133839	259137582
cg13576178	0.03011996	81961.9898	2.96853041	2262994424
cg14593867	0.03013162	561954.867	3.56928014	8.8475E+10
cg21195414	0.03013318	586186571	6.95961635	4.9373E+16
cg02290575	0.03013907	414.69597	1.78431899	96380.0469
cg20955894	0.03015595	8.17716503	1.22344601	54.6538445
cg09871837	0.03015878	16.9770761	1.31224221	219.640179
cg23378144	0.03017409	454.857697	1.79812086	115062.079
cg27090191	0.03018164	8872065.93	4.63278167	1.6991E+13
cg17146950	0.03018314	75.9284926	1.51421343	3807.34702
cg01506678	0.03019081	0.0641675	0.00535633	0.7687104
cg04750166	0.03019418	2.8969E+12	15.6096291	5.38E+23
cg13677149	0.03021123	6.75947814	1.20059782	38.0564947
cg15593510	0.03021299	0.17163513	0.03486824	0.84485537
cg03723506	0.03022521	6.76E-05	1.14E-08	0.39934431
cg15447669	0.03022638	2180996.95	4.03544687	1.1787E+12
cg23239150	0.03022958	0.05720321	0.00430124	0.76075983
cg03918756	0.03023559	0.20480318	0.04880516	0.8594243
cg07641943	0.03023696	793551.646	3.66082984	1.7202E+11

cg19079546	0.01638254	1.1477E+12	163.042399	8.08E+21
cg20852788	0.01639164	21.1424643	1.7498454	255.453308
cg15907372	0.01639334	110.556955	2.36981772	5157.71327
cg15253243	0.01639509	52474.7748	7.33490657	375410644
cg25355924	0.01639544	93623462	28.9413025	3.0287E+14
cg24158160	0.01640026	6.08825438	1.39252983	26.6183465
cg02283238	0.0164106	6.6392226	1.41461047	31.1600102
cg12855166	0.01641328	5.57154097	1.369841	22.6610745
cg17559110	0.01641373	52142.8018	7.3151201	371678351
cg03018679	0.01641382	9.55437981	1.51209445	60.3706825
cg02993070	0.01641514	8.88621743	1.49211089	52.9215761
cg09388058	0.01641528	63731.6275	7.58810111	535274936
cg20964023	0.01641583	275418.002	9.92100791	7645904201
cg01421867	0.01642562	880513.564	12.2624848	6.3226E+10
cg01907051	0.01643648	9.27875614	1.50343092	57.2658937
cg05414908	0.01643766	12.8171546	1.59497391	102.998206
cg25520440	0.01644036	0.09090591	0.01281642	0.64478904
cg26156179	0.01645757	92120.5787	8.08820198	1049207357
cg25295726	0.01646047	67.63167	2.16095972	2116.67193
cg02491878	0.01646839	15.7886777	1.65595318	150.537073
cg17264609	0.01646964	7.16407943	1.43320543	35.8106612
cg23709674	0.01647525	4877195.55	16.6800166	1.4261E+12
cg01620672	0.01647847	0.11830023	0.02067051	0.67704875
cg07565021	0.01647917	59.863824	2.11206087	1696.76806
cg01175550	0.01647948	4.54353657	1.31859424	15.6558583
cg13463731	0.01648606	11.6792695	1.56665089	87.0681132
cg10960266	0.01650025	0.06699672	0.00735211	0.61051339
cg13752649	0.01650749	17.250724	1.68155999	176.971075
cg25429672	0.01650853	11.2061463	1.55422275	80.7977592
cg01558492	0.01651718	3049.66235	4.32121999	2152271.92

cg23589617	0.03023804	12.3643037	1.27153185	120.229788
cg08165784	0.03024674	13.4612462	1.28173962	141.374385
cg04006061	0.03026506	8.90263902	1.23185743	64.3394111
cg18770763	0.03026963	61.8760993	1.48191026	2583.59211
cg11992317	0.03027368	1183274.54	3.79258006	3.6918E+11
cg03184776	0.03027787	10.1036155	1.24661305	81.8883192
cg23504474	0.0302814	8.80018189	1.23025895	62.9486996
cg00051483	0.03029211	0.08835732	0.00983629	0.7936951
cg08122234	0.03030273	6.88520004	1.20155522	39.45385
cg17707347	0.03030764	40255244.7	5.29102051	3.0627E+14
cg04482943	0.0303205	703549.831	3.59677686	1.3762E+11
cg18639233	0.03032541	4.78235622	1.16037034	19.7100273
cg18116600	0.03034139	130.554656	1.58822049	10731.8338
cg22505962	0.03035116	48.5996086	1.44566868	1633.79202
cg06155802	0.03035634	891655.975	3.66885969	2.167E+11
cg04397817	0.03036074	4162.52176	2.20446574	7859767.14
cg04510815	0.03036123	17.4972065	1.31188196	233.368735
cg00319168	0.03036391	9.59827268	1.239213	74.3430214
cg00436603	0.03036543	0.09553517	0.01140339	0.80037322
cg18044111	0.03037019	13.3640478	1.27861378	139.680784
cg06288697	0.03040276	6.71699858	1.19748327	37.6774114
cg09992116	0.03040959	3133.92351	2.14130453	4586679.03
cg24333845	0.03041459	13.3139129	1.27735644	138.771194
cg00973823	0.03041503	16.5000543	1.30352693	208.857819
cg18862975	0.03041888	15.6095088	1.29663136	187.915217
cg27313941	0.03042537	13.0234595	1.27450254	133.079764
cg15405796	0.03043318	249.72994	1.68444461	37024.0982
cg26188299	0.03044324	19903.393	2.54580923	155606731
cg18226566	0.03045513	43988559.1	5.26075534	3.6782E+14
cg24902075	0.03045763	1.6116E+10	9.17868831	2.8296E+19

cg02043083	0.01652213	232.788957	2.7020841	20055.1486	cg22165175	0.03050945	14.0856351	1.28242412	154.710999
cg11974977	0.01652748	619887567	40.1080796	9.5806E+15	cg00157359	0.03051352	7.87472852	1.21412298	51.075015
cg15130433	0.01654375	3246509.71	15.3646184	6.8598E+11	cg15190760	0.03051967	12.5822673	1.26870541	124.783459
cg02928644	0.01654591	5.13773943	1.34743088	19.5901451	cg14572436	0.03053027	116.946228	1.56401252	8744.44415
cg16763443	0.01655348	9.00589911	1.49233182	54.3486494	cg11800672	0.0305322	6.97795052	1.20016271	40.5709935
cg02320501	0.01655599	10.3825344	1.53143508	70.3895464	cg13661211	0.03053492	2130813.07	3.92882055	1.1557E+12
cg21510910	0.01656382	4.119E+12	198.050833	8.57E+22	cg24490630	0.03053854	7732.8993	2.31764058	25801123.9
cg23011817	0.01656944	3509443.79	15.538682	7.9262E+11	cg21644628	0.03054474	0.00321003	1.77E-05	0.58343152
cg04657044	0.01657529	15.0148665	1.63722157	137.700493	cg02476533	0.03057806	58.6092888	1.46419895	2346.02596
cg15545878	0.01657656	11.7760856	1.56636957	88.5335067	cg00836482	0.0305844	4.01787356	1.1390785	14.1722523
cg25756241	0.01657855	54.2387282	2.06810705	1422.47938	cg13421439	0.03058893	5.11637155	1.16510316	22.4677598
cg15726260	0.01658118	5.96972532	1.38412836	25.7473377	cg16296724	0.03058909	5430466.81	4.26999717	6.9063E+12
cg07087293	0.01658356	11.1580448	1.55088056	80.2782419	cg15977246	0.0305913	218735776	6.03356367	7.9299E+15
cg01015652	0.01658867	15.6052761	1.64829593	147.743278	cg05355225	0.03059845	5.01684591	1.16286552	21.6437261
cg04653284	0.01659391	4015847270	55.7672357	2.8918E+17	cg13109300	0.03060642	25127.6634	2.57910949	244812975
cg11346237	0.01659978	47.5567496	2.01797128	1120.75155	cg26515676	0.03061583	506803.098	3.41336367	7.5248E+10
cg14645027	0.01660791	8.68071089	1.48105284	50.8791715	cg16532497	0.03062988	8.92487839	1.22679494	64.9280919
cg20660171	0.01660831	115.593229	2.37085822	5635.84719	cg19815376	0.0306345	10.7528313	1.2482536	92.6281173
cg11090352	0.01660847	8.95286514	1.48937147	53.8171946	cg03623835	0.03063756	6.86309973	1.19697071	39.3511198
cg16278496	0.0166091	10.2831007	1.52732561	69.2335408	cg10348922	0.03063865	8.53546625	1.22156974	59.6398075
cg26807935	0.01661183	93364.613	8.00056879	1089541405	cg23466060	0.03064011	11.7202304	1.258239	109.17147
cg26912671	0.01662145	22.2748315	1.7571572	282.369796	cg00144465	0.03065207	33990985.4	5.03973452	2.2926E+14
cg02204038	0.01663754	11348771.7	19.0797187	6.7503E+12	cg24144070	0.03066501	289.297981	1.69583097	49352.3961
cg27304533	0.01664227	8.86940577	1.48601222	52.9378949	cg26505878	0.03068627	171.426554	1.6141523	18205.8802
cg05411944	0.01664324	0.08087193	0.01032283	0.6335734	cg02704345	0.03068698	47873.5825	2.72645308	840608597
cg27607583	0.01664789	183827016	31.5836191	1.0699E+15	cg11974977	0.03070245	288814204	6.12008382	1.3629E+16
cg21061582	0.01665261	11.6257867	1.56051558	86.6117055	cg02690256	0.03070872	23.3909818	1.3404848	408.164293
cg03461399	0.01665514	826.885677	3.3819759	202171.732	cg20701689	0.03071849	5.68854482	1.17528223	27.5334224
cg10187703	0.0166558	10.6880999	1.53680349	74.3331736	cg02486145	0.03072391	6.25646001	1.18565466	33.0140751
cg13531989	0.0166588	615411919	39.2599006	9.6468E+15	cg07667560	0.03072875	14.4760115	1.28163256	163.50623

cg22132343	0.01666607	0.10159031	0.01562289	0.66060699
cg06839900	0.01667946	7.3104337	1.43398663	37.2684372
cg01035068	0.01667974	90.6636284	2.26297481	3632.33982
cg13992236	0.01668129	546696727	38.2953015	7.8045E+15
cg19081101	0.01668415	5.14137665	1.3453069	19.6488652
cg10226922	0.01668524	2.2352E+16	915.713084	5.46E+29
cg26140483	0.01668527	0.09480611	0.01377284	0.65260298
cg08169325	0.0166996	8.13122572	1.46144003	45.2408792
cg26960317	0.0167028	0.00734812	0.00013141	0.41089322
cg20371266	0.01671154	5.89702173	1.37864216	25.2239967
cg08447739	0.01671562	8.30334298	1.46661892	47.0098289
cg02852873	0.016716	1312.68915	3.66563592	470082.911
cg24719487	0.01671947	9.66994102	1.50751219	62.027863
cg02154874	0.01671956	544801537	38.050375	7.8004E+15
cg18938674	0.01672326	60206.367	7.31964256	495216344
cg25917723	0.01672581	85402.289	7.79572067	935583927
cg15827486	0.01672862	1.9973E+12	167.599976	2.38E+22
cg21145140	0.01673304	13.0104367	1.59022731	106.444822
cg23115158	0.01673371	1076096.42	12.3169272	9.4016E+10
cg05842391	0.01673733	21084.7018	6.04805114	73505438
cg00762037	0.01674098	5610.94389	4.75977365	6614325.32
cg04885941	0.01674176	4413883.96	15.8825442	1.2267E+12
cg03616995	0.01674619	6.5067E+12	206.697875	2.05E+23
cg20607798	0.01674933	8.18206331	1.46194163	45.7926354
cg18907495	0.01675021	77.3546336	2.19374884	2727.63191
cg18642003	0.01675471	2385.16348	4.07466113	1396190.91
cg06857614	0.01675533	5.43886358	1.3578559	21.785255
cg14775474	0.01675668	31286.8841	6.48551656	150931558
cg17518962	0.01675754	10.9973775	1.54194585	78.4348628
cg04844391	0.01676006	6.1806E+13	309.430002	1.23E+25

cg10705379	0.0307331	6.09901445	1.18274468	31.4505555
cg12516270	0.03074916	24.2419468	1.34400368	437.254741
cg17783086	0.03076638	6.94338748	1.19664691	40.2880996
cg20890243	0.03077388	30.3219176	1.37155947	670.345479
cg01528492	0.03077668	12.9890702	1.26795369	133.061598
cg27173965	0.03077989	13.0273131	1.26824215	133.815839
cg16674492	0.03078744	3.50259782	1.12297981	10.9246768
cg07690778	0.03078814	759.222409	1.84717097	312054.854
cg08085208	0.0307928	71406.2063	2.81171094	1813431894
cg10056728	0.0307973	26.9354955	1.35603289	535.031949
cg09619475	0.03081821	71551984.8	5.31459681	9.6333E+14
cg06646636	0.03083222	42.6266787	1.41382999	1285.18546
cg15622885	0.03083453	5335341.43	4.17580847	6.8169E+12
cg23175074	0.03083497	11.3045902	1.25079252	102.17023
cg07297068	0.0308601	0.11520457	0.01619612	0.81946117
cg02970696	0.03086282	3.855042	1.13236192	13.124204
cg10115182	0.03086885	822991.318	3.50538253	1.9322E+11
cg09423126	0.03087443	9.89758557	1.23494028	79.3254556
cg10713002	0.03087551	571.171193	1.79376219	181872.789
cg19527084	0.03088316	4.09993063	1.13862759	14.7628876
cg20402382	0.03088838	367.020238	1.72149259	78248.2919
cg26857670	0.03089303	6.54652517	1.18860538	36.0565352
cg13050390	0.03090389	7.30215295	1.20046458	44.4173352
cg16079865	0.03091506	0.00538204	4.68E-05	0.61889355
cg07115761	0.03093365	13.391741	1.2687395	141.351891
cg14331899	0.03094031	9.87257391	1.23364446	79.0079469
cg03997400	0.03096208	2007.69517	2.00664633	2008744.55
cg13694291	0.03096936	2014294.97	3.77661007	1.0743E+12
cg25104555	0.03096953	3.58246274	1.12390927	11.4191061
cg08069043	0.03097417	7421.93351	2.26058254	24367655.7

cg18003795	0.01676213	0.00625627	9.79E-05	0.40000396
cg18832407	0.01676232	7.83924372	1.45038882	42.3705294
cg23352722	0.01677921	6.06798338	1.38452188	26.5943233
cg26300461	0.01678236	6.69559825	1.40926807	31.8115743
cg04984052	0.01678238	3286.54086	4.3103195	2505928.12
cg03269218	0.01678299	5.95164268	1.379624	25.6751481
cg12520032	0.01679402	1009499580	42.0518355	2.4234E+16
cg24938727	0.01679512	18.0030289	1.68413345	192.448556
cg01214054	0.01680163	4.53820414	1.31348137	15.6799307
cg07418892	0.01680295	8.58934345	1.47355469	50.0672432
cg08420572	0.0168184	5.73319328	1.36971269	23.9973722
cg09176275	0.01683547	12.1305587	1.56721581	93.8929114
cg14697743	0.01683614	4.87465869	1.32998191	17.866632
cg00088227	0.0168369	1.9018E+11	107.260935	3.37E+20
cg24981593	0.01683721	5.02717185	1.33736123	18.8972556
cg01262913	0.01685076	7.92453561	1.45123439	43.2723101
cg27148665	0.01685402	9.1177696	1.48824209	55.8603488
cg10902825	0.01686195	5.57786564	1.36218868	22.8401436
cg09908601	0.0168628	1531.84186	3.73918003	627554.562
cg09080746	0.01686391	2.67E-10	3.74E-18	0.01898542
cg24287110	0.01686428	88.3056353	2.23827262	3483.88537
cg07898632	0.01686438	5.90169729	1.37603872	25.3118102
cg02577849	0.0168653	2818381766	50.0213627	1.588E+17
cg02483701	0.01686613	13.3760369	1.5940873	112.238749
cg08958015	0.01686772	5.02193571	1.33661799	18.8683966
cg02371464	0.01686934	15.8719724	1.6437771	153.256489
cg19585103	0.01687046	4.14928497	1.29148902	13.3307875
cg02951062	0.01687635	6.36058587	1.39446075	29.0126866
cg15080119	0.01688561	4.17925934	1.29295188	13.5087848
cg19050940	0.01688684	16.6456176	1.65729185	167.186356

cg03324464	0.0309924	15.4082873	1.28405394	184.895128
cg21915765	0.03101616	48910.1044	2.67977058	892687730
cg03137131	0.03103937	7.09289057	1.19554345	42.0805257
cg26981201	0.03105111	70.0090809	1.47264007	3328.22086
cg07702750	0.03105208	7.98000566	1.2082846	52.703221
cg24827036	0.03107195	3494125.39	3.93887594	3.0996E+12
cg06214007	0.03107619	6.37059123	1.18345886	34.2930658
cg05147525	0.03108262	343.582464	1.70061067	69415.5999
cg10256686	0.03108687	41100.1132	2.62686408	643055466
cg22927247	0.03109277	11.6490371	1.24997359	108.562345
cg23601586	0.03112061	1107.72824	1.88890582	649615.154
cg15106368	0.03112545	52.484528	1.43221177	1923.33686
cg05286653	0.03113081	10.6973641	1.23972647	92.3055209
cg20546782	0.03114457	81.1204136	1.48922925	4418.74311
cg10510478	0.03115244	4.95713976	1.15599617	21.2571938
cg02847589	0.03116134	79698188.5	5.18959184	1.224E+15
cg10997640	0.03116238	4740631.09	4.01948095	5.5912E+12
cg01200642	0.03117241	7.7035E+10	9.65311495	6.15E+20
cg11920449	0.03117455	9.87129608	1.23006087	79.2176136
cg12008118	0.03118293	46.4810651	1.4148338	1527.027
cg20746096	0.03118344	292742.836	3.1196207	2.7471E+10
cg13661243	0.03118547	9.49669582	1.2256024	73.5860437
cg20818283	0.03118566	779740.859	3.40790657	1.7841E+11
cg27111970	0.03120328	4.23916931	1.13928198	15.7735809
cg08871855	0.031226	8.43480012	1.21197348	58.7024833
cg10297105	0.03122766	20.67886	1.31399454	325.431527
cg25697727	0.03124737	14.2703624	1.27042412	160.295479
cg06160809	0.03125391	14.2790664	1.27037497	160.497287
cg05250768	0.03126963	4.19386214	1.13759421	15.4611192
cg25782440	0.03127069	78.2274083	1.47994605	4134.96656

cg08611810	0.01688733	8.42756528	1.466538	48.4296053
cg04383058	0.01688799	10.7018233	1.53082986	74.8149914
cg16733558	0.01689489	13.891988	1.60406479	120.311432
cg13572511	0.01689911	29.3558583	1.83454264	469.744554
cg21785710	0.01690178	7.13041634	1.42285831	35.7328885
cg18507365	0.01691619	82.7777165	2.20862007	3102.4577
cg11613427	0.01691922	22.9853359	1.75487899	301.061023
cg06427502	0.01692441	221.192255	2.63369689	18576.9341
cg01404317	0.01692534	15.1742944	1.62869368	141.376622
cg16523704	0.01692705	5.76954934	1.36932321	24.3096
cg15306012	0.01692839	12.564501	1.57439538	100.271309
cg27555036	0.01692926	16.7356066	1.6574186	168.985992
cg11316887	0.01693004	12.9762061	1.5834749	106.336972
cg02096916	0.01693796	5.0359E+14	432.045012	5.87E+26
cg27123665	0.01693911	352.783316	2.86187365	43487.6179
cg02696067	0.01694113	12.4139285	1.57061893	98.1177667
cg12800244	0.01694538	28.8967531	1.82723485	456.986873
cg01793704	0.01694723	153.692827	2.46509405	9582.38697
cg10037049	0.01695317	6.48771304	1.39792984	30.109108
cg24310786	0.0169536	17.3810217	1.66783908	181.132532
cg03812679	0.01696205	36.7355377	1.90668972	707.771022
cg00871610	0.01696535	10.4006937	1.52094423	71.123206
cg16473288	0.01697059	0.16709125	0.03846038	0.72592849
cg26833120	0.01697193	11.922733	1.55840788	91.2158902
cg17246140	0.01698236	5.40294415	1.35235482	21.585907
cg04926361	0.016985	7.03202007	1.41759656	34.8824959
cg26511108	0.01698695	0.1271625	0.02338546	0.69146828
cg24876960	0.01699067	3.78680194	1.2689234	11.3008153
cg20941170	0.01700329	26277400.2	21.2051429	3.2563E+13
cg06349174	0.0170108	170199601	29.5825421	9.7922E+14
cg15011837	0.03127302	44.9023767	1.4078196	1432.16037
cg03265267	0.03131048	275.579085	1.65538074	45876.9577
cg21328770	0.03131765	143044.608	2.89910198	7057964805
cg18146843	0.03131791	4.69793923	1.14880251	19.21186
cg26764215	0.03133241	1273884.66	3.52313738	4.6061E+11
cg26522057	0.03135927	8991399.79	4.18750678	1.9306E+13
cg01550161	0.03138615	50.8992277	1.42037494	1823.97711
cg15283028	0.0313868	3.9092E+12	13.3162627	1.15E+24
cg24921484	0.03138874	6.5611E+12	13.9421434	3.09E+24
cg05735639	0.03139629	14.0224816	1.26574052	155.347788
cg25153340	0.03140005	11640436.6	4.27008831	3.1732E+13
cg20929545	0.0314132	7.03285117	1.18993311	41.5661985
cg04014328	0.03141646	7.44165423	1.1959001	46.3067256
cg09216143	0.03142094	0.12858494	0.01984996	0.83295301
cg21041329	0.03142355	181447.928	2.94123884	1.1194E+10
cg00456593	0.03142535	0.06781768	0.00584516	0.78684537
cg02344701	0.03144249	0.00641416	6.45E-05	0.63804222
cg18581950	0.03144808	13.1366817	1.25748992	137.235618
cg11843238	0.03146192	5.902676	1.17094903	29.7549963
cg05651995	0.03146361	57.5666578	1.43365408	2311.52001
cg04918402	0.03147569	6.98628101	1.18845306	41.0686161
cg10512875	0.0314771	3.93787781	1.12944203	13.7296836
cg03586793	0.03147788	9.72107002	1.22380604	77.217467
cg19705910	0.03148655	6.40808888	1.17924191	34.822035
cg24812891	0.03148845	440.627131	1.71653691	113106.958
cg17684754	0.03150963	80.9184717	1.4761027	4435.86958
cg26450387	0.03152028	61.7147506	1.44074056	2643.57828
cg06527213	0.03152481	11.2133966	1.23867164	101.512185
cg07138512	0.03153119	31.0769783	1.35552384	712.476282
cg22622425	0.03155155	32143.1614	2.50302291	412774020

cg03157027	0.01701291	7.0722138	1.41846645	35.2607622
cg21653581	0.01702132	5123.71031	4.59924018	5707987.92
cg11930955	0.01702214	9569.50616	5.14195352	17809466.3
cg02631838	0.01702963	14.6210406	1.61451315	132.408229
cg04795713	0.01703137	4143.91259	4.42537113	3880355.13
cg14807549	0.01703561	6034880862	55.7436344	6.5334E+17
cg07486252	0.01703934	7.34901928	1.42767222	37.8294706
cg13313046	0.0170443	11426297.7	18.1815063	7.1809E+12
cg18802611	0.01705641	4247521.09	15.2171104	1.1856E+12
cg06470558	0.01705711	68.6403737	2.12618447	2215.94174
cg21688288	0.017064	0.0584464	0.00566804	0.60267444
cg08374291	0.01707299	23747761.2	20.6424503	2.732E+13
cg17783086	0.01707416	6.68883318	1.40319453	31.8847375
cg25766046	0.01707872	10.8466877	1.52934553	76.9287458
cg20699549	0.01708708	6.32561902	1.3890535	28.8062742
cg13383058	0.01709748	7.02725098	1.41511619	34.8962556
cg25570328	0.01709839	0.04865817	0.00405591	0.58374461
cg08698605	0.01711516	23.8309325	1.75814704	323.018115
cg00928397	0.01711615	11.6043469	1.54680648	87.0573458
cg22488745	0.01712536	6.69596545	1.40244802	31.9697791
cg02120774	0.01712715	6.21776316	1.38405261	27.9328823
cg05176146	0.01713204	1.7038E+11	99.3497111	2.92E+20
cg18070812	0.01713518	0.08704728	0.01169548	0.64787647
cg04617640	0.01714216	6.99554739	1.41306659	34.6322557
cg19558029	0.01714237	6.97369371	1.4122768	34.4354618
cg01943874	0.0171428	4.04361027	1.28187952	12.7553204
cg04199779	0.01715414	384.502414	2.87887688	51354.0914
cg22312127	0.01715477	9.2061E+11	133.485665	6.35E+21
cg06527213	0.01715577	8.59433157	1.46540338	50.4042343
cg03662064	0.01715835	10.987581	1.53070024	78.8703976

cg19486585	0.03156822	13520949	4.26387266	4.2876E+13
cg17390562	0.0315722	5507.08932	2.13951439	14175194.6
cg17373751	0.03157763	23.0540747	1.31913109	402.909434
cg04920683	0.03157801	2742170.3	3.70057396	2.032E+12
cg08000847	0.03158495	6377830.79	3.98449097	1.0209E+13
cg01439366	0.03158648	704431.455	3.2802278	1.5128E+11
cg10007534	0.03158683	0.00470813	3.56E-05	0.62330413
cg22624697	0.03159324	52.4527341	1.41794023	1940.34224
cg23272399	0.03160236	7.06904144	1.18811761	42.0592595
cg06178310	0.0316055	7.45946124	1.19372	46.6135795
cg07167398	0.03160564	1.8245E+13	14.7419192	2.26E+25
cg13549667	0.03160565	26.5985615	1.3352312	529.858403
cg00341935	0.03160728	0.00117287	2.49E-06	0.55178752
cg10090217	0.03160791	18698.2367	2.37888398	146969780
cg09638001	0.03160992	281234.41	3.02022777	2.6188E+10
cg07701375	0.03161052	2239158840	6.66304038	7.5248E+17
cg20034372	0.03161736	5.60097175	1.16382854	26.9549024
cg21496913	0.03163989	1996.73998	1.95080871	2043752.69
cg04755365	0.03165126	21.2679677	1.30819682	345.763301
cg07099000	0.0316571	5.89360511	1.16861834	29.7227761
cg09467487	0.03165981	211.479636	1.60038748	27945.5051
cg05488632	0.03166866	16.4444971	1.27862683	211.493673
cg24830559	0.03167324	6782.2839	2.16884851	21209123
cg19099736	0.03168135	2679.08109	1.99838952	3591629.87
cg16674484	0.03169384	7.63781251	1.19505854	48.8144955
cg09661370	0.03172521	8.846495	1.21010321	64.6725611
cg02965250	0.03174255	64.2831509	1.43880701	2872.04847
cg04962480	0.03175419	245.188037	1.61681537	37182.4606
cg15726260	0.03176053	8.20057181	1.20162648	55.9652929
cg03080561	0.03176367	4350868.73	3.79638176	4.9863E+12

cg25699052	0.01716026	4.78E-05	1.34E-08	0.17087961
cg02313495	0.01716473	15.2023462	1.6213655	142.541167
cg27071285	0.01717016	77.0790157	2.16272825	2747.07405
cg10625266	0.01717556	21.5574388	1.7246867	269.453676
cg00188032	0.01717622	5.72850245	1.36315966	24.0732918
cg06443918	0.01719331	849904.462	11.2634166	6.4131E+10
cg18067644	0.01720148	16.0256211	1.63538767	157.039543
cg05797937	0.01721567	123.054526	2.34621296	6453.98209
cg02818811	0.01721628	5.681201	1.36045501	23.7244484
cg00778544	0.01721651	2177390920	45.1475491	1.0501E+17
cg09180877	0.01721724	0.13886287	0.02735879	0.70481531
cg09938049	0.01722488	12.8610139	1.57213285	105.211006
cg16003064	0.01723285	82.26151	2.18339216	3099.28566
cg02096793	0.01723337	6.51077838	1.39338196	30.4225519
cg01510388	0.01723453	9.6149149	1.49293887	61.9225545
cg08114317	0.01723748	10.9202794	1.52689369	78.1013794
cg24591969	0.01723752	0.19213846	0.04943759	0.74674335
cg14871010	0.01723841	0.04536532	0.00355838	0.57835665
cg14019947	0.01724423	1.6741E+11	96.9390447	2.89E+20
cg01542023	0.01725262	8.32457195	1.45491604	47.6305824
cg07394446	0.01725587	6.20486042	1.3811397	27.8757412
cg12796015	0.01725598	1.6126E+11	96.0835661	2.71E+20
cg13933139	0.01726746	67997586.2	24.2622775	1.9057E+14
cg07721872	0.0172735	180.513079	2.50548805	13005.4389
cg06315439	0.01728	9.89414019	1.49937197	65.2900093
cg23273694	0.01730035	5.32643322	1.34360449	21.1155077
cg12943155	0.01730418	13.1805514	1.57660184	110.190747
cg26807590	0.0173043	0.10255217	0.01572188	0.66893684
cg19539667	0.01731027	5.5469184	1.35309246	22.739247
cg06993307	0.0173113	9.33797487	1.48335577	58.7841274

cg01093696	0.03176696	8.61182884	1.20668074	61.4608266
cg23371754	0.0317678	7.15074264	1.18725263	43.0684413
cg04762213	0.03177729	149.361316	1.54737132	14417.2265
cg27083176	0.03177826	19908019.3	4.32952959	9.1541E+13
cg27380724	0.03178622	0.12804462	0.01961185	0.83599577
cg10539808	0.03181282	17.5622592	1.28319257	240.363728
cg23865240	0.0318155	7.02455112	1.1848211	41.6470624
cg17476026	0.03181878	10.2249818	1.22410691	85.4094134
cg09671955	0.03182655	5.46939771	1.15918978	25.8062241
cg26782361	0.03184392	17.6348915	1.2830421	242.384407
cg06970090	0.03185269	23.6104887	1.31577804	423.669616
cg18832152	0.03185332	20.3225561	1.29874957	318.00302
cg19628148	0.03185733	5.38645407	1.15733063	25.0696617
cg09927483	0.03186991	51680.145	2.56255679	1042254906
cg19844724	0.03187039	1452.98197	1.88009114	1122901.2
cg14431006	0.03187168	7.74597829	1.19420768	50.2426675
cg10937302	0.03189464	58.1814997	1.42162388	2381.141
cg19620294	0.03190371	7.21541685	1.18648357	43.8794447
cg18789887	0.03191144	7.81227339	1.19456966	51.0908803
cg11216502	0.03192899	2483.5354	1.96473312	3139331.26
cg18426477	0.03193317	13.6791763	1.2534877	149.279379
cg07705594	0.03194212	98068.8943	2.6969582	3566057502
cg23447841	0.03194668	0.12212483	0.0178819	0.83405403
cg26286839	0.03194966	6.42053972	1.17402665	35.1127721
cg05003455	0.03196348	50702641	4.61561323	5.5697E+14
cg22295389	0.03196564	1582.44691	1.88698499	1327057.83
cg02308209	0.03196731	6.9944E+10	8.60349447	5.69E+20
cg24189904	0.03196978	6.7216921	1.17843834	38.3398462
cg04886060	0.03197311	7.10241438	1.18400542	42.6047796
cg16762077	0.031974	14.5222712	1.25924627	167.478248

cg23458341	0.01731764	1793168.14	12.6895046	2.5339E+11
cg13990355	0.0173178	7.50988515	1.42726128	39.5151021
cg07513446	0.0173179	10.8178912	1.52219869	76.8800889
cg17532444	0.01732423	690.048568	3.16795785	150307.248
cg18132058	0.01732482	0.06823257	0.00747589	0.6227601
cg16036409	0.01732483	7.03885791	1.41089899	35.1162777
cg16849609	0.01732564	7.49021159	1.42643497	39.3311092
cg07034561	0.01732656	761.181509	3.22289556	179775.385
cg08962271	0.01732708	22.6697185	1.73409446	296.359945
cg18151074	0.01732915	3624.14038	4.24342059	3095237.25
cg18508125	0.01733326	167.268597	2.46639891	11343.9815
cg14525897	0.01733899	9.78266684	1.49489053	64.0184471
cg09149672	0.01734236	0.24749555	0.07834816	0.78181858
cg15177964	0.0173446	856.835003	3.28794468	223290.321
cg06275813	0.01734575	22.9622188	1.73727204	303.500822
cg02304134	0.017354	353.550731	2.81183328	44454.314
cg04457423	0.01735538	190.612804	2.52172119	14408.1119
cg06032048	0.01735918	6687.09895	4.71834496	9477325.77
cg09617296	0.01736241	4549017088	50.228189	4.1199E+17
cg25423888	0.01736316	14862.9664	5.42957663	40686002.9
cg15262352	0.01736437	1231.79706	3.50154379	433330.01
cg23666930	0.01737659	496.932452	2.98256174	82795.2221
cg25580656	0.01737785	24.9397597	1.76141349	353.120726
cg26023912	0.01738313	9.82134858	1.49482217	64.5286712
cg00986320	0.01738432	2674.49515	4.0091473	1784151.04
cg06848185	0.01738778	5.27260661	1.33976327	20.7502184
cg09397246	0.01738998	6.26594352	1.38103062	28.4295277
cg11580525	0.01739614	16.62599	1.63946384	168.606063
cg11343211	0.01740133	4.06213793	1.27949749	12.8964415
cg27625456	0.01740371	7.80535844	1.43514877	42.4510835
cg03716942	0.03198665	5560550984	6.90076369	4.4806E+18
cg05605149	0.03200656	9.5013E+10	8.78711174	1.03E+21
cg06152533	0.03201063	15.7585034	1.26745867	195.927832
cg23873398	0.03201474	419055.028	3.04190513	5.7729E+10
cg22017954	0.03203693	121414.133	2.73094308	5397912433
cg09865015	0.0320393	24.9752834	1.31798775	473.270547
cg03377767	0.03204414	18.6061289	1.28501541	269.403799
cg09295382	0.03205927	9286.43724	2.18793279	39415249.4
cg12975295	0.03206452	26007.5789	2.38912582	283113661
cg07869830	0.03206474	22.3700757	1.30503942	383.452239
cg02916102	0.03207431	5.01055187	1.14794832	21.8700003
cg07048306	0.03208001	103.880884	1.48795333	7252.40362
cg20122491	0.03208757	119580.319	2.71880181	5259468621
cg23837756	0.03209361	212594.691	2.85485607	1.5831E+10
cg04983381	0.03212235	234469380	5.18187377	1.0609E+16
cg00901161	0.03212846	12.7653054	1.24273087	131.12495
cg10296162	0.03213373	3089839.58	3.5776769	2.6685E+12
cg06380459	0.03213557	198355165	5.10156021	7.7123E+15
cg05586209	0.03213833	163.972669	1.54478895	17404.99
cg11330839	0.03214375	11661.0238	2.22168311	61205612.7
cg11251498	0.03218227	13.8967476	1.25082783	154.393425
cg11416076	0.03218417	9.23392027	1.20806475	70.5800609
cg22276811	0.03218632	25.2255992	1.31579296	483.610169
cg21014483	0.03220135	6.17505128	1.16723655	32.6679782
cg01272428	0.03220556	126.002115	1.50787068	10529.1078
cg21037008	0.03220601	5.43942135	1.15467936	25.6238274
cg24403578	0.03220653	37.8820346	1.36154646	1053.98426
cg24214699	0.0322143	5.67504867	1.15875635	27.7937439
cg14122599	0.03222256	24.2013887	1.31036076	446.981651
cg24986868	0.03223415	26.3895694	1.3197522	527.681918

cg24738356	0.01740618	0.14171463	0.0283145	0.70928462
cg16598292	0.0174074	8.4029218	1.45379974	48.5686528
cg19803043	0.0174194	152562368	27.4055282	8.4929E+14
cg06468809	0.01742891	6.31771997	1.38230293	28.8747024
cg09038058	0.01743038	2247936.18	13.0467341	3.8732E+11
cg10706013	0.01743612	0.18105514	0.0442521	0.74077757
cg06046912	0.01744587	11.6229714	1.53804921	87.8342926
cg26358945	0.01744641	3157042781	46.4674655	2.1449E+17
cg19792268	0.01745021	17.6302517	1.65457678	187.858174
cg12268958	0.01746093	28360.0897	6.039321	133176344
cg19007537	0.01746224	8752219605	55.4260727	1.382E+18
cg19907326	0.01746525	680.669789	3.139039	147596.561
cg24053664	0.01747133	5.64898865	1.35467616	23.5562371
cg08856118	0.01747573	56.1415769	2.02594757	1555.75431
cg05995267	0.01748494	2545756.6	13.2565144	4.8888E+11
cg20835282	0.01748601	6.18601212	1.37614773	27.8071497
cg06910925	0.01749003	43.0225674	1.93283985	957.627867
cg26259537	0.017491	4.37248417	1.29491171	14.764418
cg19484886	0.01750284	270.004922	2.66506011	27354.9769
cg06372962	0.01750549	5.69159288	1.35587202	23.8918047
cg18945601	0.01750778	12935750.9	17.5768648	9.5201E+12
cg23031484	0.01751003	7.79573306	1.43254283	42.4234813
cg23368715	0.01751338	29.8698072	1.81210593	492.358294
cg00599393	0.01751632	16568.8732	5.4744002	50147513.7
cg26994413	0.01752421	0.16097768	0.03566926	0.72650269
cg26979537	0.01753165	7.30313076	1.41582362	37.6711606
cg03557733	0.01753479	16.2337699	1.62797806	161.878892
cg07587786	0.01754091	0.09486819	0.01358475	0.66250577
cg26360961	0.01754189	94.5205042	2.21477243	4033.87977
cg03432814	0.01754818	9.16828723	1.47287944	57.0701774

cg04110544	0.03225285	6.31898834	1.1689343	34.1589887
cg13003311	0.03225307	7.00597667	1.17919088	41.6249056
cg15684116	0.0322532	2042853.52	3.421925	1.2196E+12
cg14346208	0.03226937	5.82603746	1.16074398	29.2422043
cg02576345	0.03229313	4745.65763	2.04401315	11018161.1
cg24886176	0.0322986	2385.05314	1.92819291	2950160.46
cg00401745	0.03229914	4.80040102	1.14160529	20.1854793
cg17711535	0.03230585	198274.104	2.79915162	1.4044E+10
cg19856705	0.0323086	10.0444486	1.21488753	83.0455044
cg02999463	0.03233956	17805.7648	2.2800151	139054018
cg01818648	0.03236233	1621.53034	1.86174044	1412313.22
cg04971769	0.03236348	0.05739222	0.00418856	0.78639523
cg17679455	0.03236752	34956.46	2.40954786	507130038
cg05862007	0.03236786	0.18694585	0.04023935	0.86852177
cg19484886	0.03237949	209.759981	1.56682391	28081.8088
cg00620542	0.03238063	7915.28381	2.1253974	29477648.6
cg03721058	0.03238446	18.1749553	1.27573424	258.932454
cg26032238	0.03238449	8.37127458	1.19533125	58.626626
cg10096454	0.03238563	13.42864	1.24370064	144.993391
cg01501474	0.03239376	1.2312E+10	7.02756805	2.1569E+19
cg11594683	0.03242302	2.29E-05	1.28E-09	0.408603
cg02882448	0.03242523	18.9905039	1.27963217	281.830393
cg07490151	0.03244604	9480.00495	2.15101779	41780451.2
cg05468346	0.0324474	30.6021723	1.33127108	703.457742
cg26980034	0.03245308	40.0075095	1.36129743	1175.79067
cg00501272	0.03246185	5.68227036	1.15624431	27.925064
cg10542419	0.03246738	1.35E-11	1.47E-21	0.12356702
cg16489586	0.03247316	27.2848879	1.31796287	564.860458
cg22455694	0.03248335	16.5824711	1.26409032	217.530617
cg14539231	0.03249176	8.09047715	1.19049857	54.9818554

cg27111463	0.01754911	8.38965681	1.45019052	48.5359272	cg11479035	0.03250186	0.16279843	0.03083259	0.85958803
cg01719529	0.01755031	1075.84315	3.38667961	341762.024	cg10671946	0.03250797	9.61869963	1.20756878	76.6162427
cg14908680	0.01755323	0.00017036	1.32E-07	0.21955333	cg03840075	0.03251894	0.05686703	0.00410575	0.78764209
cg03184011	0.01756055	728.906024	3.1623867	168007.281	cg14252395	0.03252929	235.014709	1.57503128	35067.1852
cg12525224	0.01756197	199332288	28.1564813	1.4112E+15	cg25229048	0.03254115	0.11296221	0.01529708	0.8341761
cg09336406	0.01756575	5.653744	1.35325575	23.6206802	cg14415214	0.03255223	9.76E-07	3.01E-12	0.31666992
cg02681400	0.01756793	13.3337488	1.57192454	113.102666	cg09946603	0.03256388	0.00059629	6.59E-07	0.53986869
cg19536127	0.01757861	55.2060678	2.01390162	1513.33605	cg09730369	0.03256659	0.0784976	0.00761114	0.80958627
cg12504804	0.01757982	243964.598	8.71427535	6830025741	cg24516901	0.03259752	11.1218484	1.22087528	101.317074
cg27093949	0.01758039	8.03256755	1.43852474	44.8529939	cg00692933	0.03261741	465786804	5.21449807	4.1607E+16
cg08781740	0.01758215	2387838175	43.3062946	1.3166E+17	cg17029237	0.03262501	292.623488	1.59943079	53536.8619
cg05884501	0.01758262	130.403147	2.33954029	7268.51371	cg05022363	0.03262533	101365.013	2.59407159	3960902985
cg14798020	0.01758626	0.09932393	0.01476051	0.66835383	cg06733311	0.03264323	259.375129	1.58271116	42506.4658
cg18412690	0.0175864	4874676.88	14.6859742	1.618E+12	cg14849526	0.03266621	187369.166	2.72213945	1.2897E+10
cg25197388	0.0175936	35.7586594	1.86616458	685.192363	cg14165186	0.03267062	0.00010371	2.29E-08	0.46931293
cg03478249	0.01759481	10.3688498	1.50369858	71.4990675	cg04888113	0.03270662	47.4439638	1.37372648	1638.55742
cg23927983	0.01759529	8691616.76	16.2283182	4.6551E+12	cg27639104	0.03270708	0.00664216	6.66E-05	0.66198107
cg00682096	0.01760856	7.11579391	1.40785143	35.9658141	cg18464137	0.03271428	12.8280137	1.23345729	133.411944
cg03982801	0.01762289	7.07267768	1.40607315	35.5762212	cg16663533	0.03272844	9456511.02	3.74192566	2.3898E+13
cg15747848	0.01762658	3368.31177	4.11550946	2756772.71	cg05019913	0.03273577	729465871	5.34321904	9.9588E+16
cg17703996	0.01763637	19631.0017	5.5906365	68932442.4	cg02774070	0.03274381	8.8381E+11	9.56078126	8.17E+22
cg14732540	0.01763793	8.95515117	1.46473281	54.7504171	cg26616609	0.03274758	52.6388369	1.3843358	2001.57155
cg17029237	0.01764064	239.773086	2.59586778	22147.173	cg06467181	0.03275083	6278089771	6.36489036	6.1925E+18
cg16907298	0.01764139	2262190441	42.5022581	1.2041E+17	cg18294691	0.03275584	5.17081365	1.14424731	23.3667263
cg25733934	0.01764817	161.510201	2.42263831	10767.4121	cg23273694	0.03276517	5.95927862	1.15754147	30.6796798
cg14685356	0.01764978	0.10152098	0.01534571	0.67162142	cg04117301	0.03276675	3525.39091	1.9530226	6363664.78
cg00473834	0.0176528	1.6368E+11	89.3655087	3.00E+20	cg05676593	0.0327763	12630.6613	2.16731252	73608952.7
cg20587127	0.01765544	1847571.88	12.3092674	2.7731E+11	cg04380955	0.03278745	8.7663458	1.19444159	64.3386998
cg10790723	0.01765939	195.259637	2.5029007	15232.856	cg11703722	0.03279423	4024.55278	1.97196493	8213647.66
cg22099236	0.01766672	1253546.78	11.492738	1.3673E+11	cg15322516	0.0327988	8.38388445	1.18993748	59.0699256

cg23135546	0.01766803	5.56811705	1.3479267	23.001197	cg03459809	0.03280847	7.55370937	1.17971158	48.3665045
cg05969021	0.01767073	5.5037E+11	109.969949	2.75E+21	cg11784697	0.03281505	1.3216E+12	9.778911	1.79E+23
cg02304201	0.01767408	11220.4832	5.05884486	24886954.6	cg10514594	0.03282276	8216613.01	3.66984898	1.8397E+13
cg10793756	0.01767755	5625140.95	14.9028073	2.1232E+12	cg06209740	0.03283007	3315.68168	1.93799069	5672754.3
cg16580499	0.01768139	3.49883503	1.2431581	9.84737708	cg14892066	0.03284342	11413.2671	2.14231842	60804530.9
cg09089417	0.01768237	3036442638	44.446364	2.0744E+17	cg20502259	0.03284689	1436.14405	1.8088963	1140203.41
cg10925082	0.01768292	4.79073029	1.31291565	17.4810139	cg19937878	0.03285124	21661.8986	2.2563169	207966288
cg23980468	0.01768911	6.23093403	1.37416118	28.2532642	cg02284939	0.03285223	202.804255	1.54183014	26675.8088
cg15084269	0.01768991	11.0057081	1.51689039	79.8512608	cg09358240	0.03286756	2.2479E+10	6.96434085	7.2559E+19
cg04941721	0.01769037	22.7444234	1.72074859	300.630087	cg03119288	0.03286958	2975.69806	1.91776493	4617238.97
cg05951609	0.01771113	4.93495622	1.31926803	18.4600796	cg26552774	0.03287424	0.10555506	0.01337937	0.83276487
cg07500988	0.01771122	54874182.2	22.045263	1.3659E+14	cg09718640	0.03289315	535479.44	2.92203086	9.813E+10
cg12811135	0.01771309	0.03036308	0.00169079	0.54525877	cg10709343	0.03289471	273.233792	1.57777049	47317.8485
cg23606286	0.01771419	212388044	27.8710318	1.6185E+15	cg11780327	0.03289478	22741.5215	2.26008522	228830662
cg05368724	0.01772275	11.9133703	1.53700833	92.340678	cg04848925	0.03291036	16.1729476	1.25358033	208.653747
cg05722148	0.01772308	751502.013	10.4561918	5.4012E+10	cg01298678	0.03294954	8.67816768	1.19125886	63.2193361
cg24848890	0.01772435	6.59316017	1.38704709	31.3397874	cg14444703	0.03295335	300329.431	2.77675566	3.2483E+10
cg01741932	0.01772494	603511230	33.3564877	1.0919E+16	cg01932843	0.03296262	8.29E-05	1.47E-08	0.46743217
cg06791405	0.01772552	64.5595567	2.06046454	2022.81393	cg02375289	0.03297655	5.04437827	1.13978487	22.3250482
cg15980914	0.01772553	0.01225317	0.0003222	0.46599123	cg08378567	0.03298273	85.4898759	1.43263353	5101.45738
cg19556079	0.01772576	122397.407	7.6303194	1963368024	cg01826863	0.03299887	4.88704473	1.13665734	21.0117907
cg11323985	0.01773666	11.5179397	1.52765645	86.8408173	cg09834465	0.03300773	6.69472724	1.16580646	38.4449516
cg18436984	0.01773816	13.6780881	1.57382524	118.87603	cg06189038	0.03301259	24.2795837	1.29341117	455.770137
cg22280792	0.01774131	57785.3713	6.69057354	499082645	cg15623892	0.033033	191.784591	1.52718775	24084.3534
cg20666917	0.01774785	17.0881007	1.63542076	178.549271	cg05411944	0.03303346	0.0021426	7.53E-06	0.60953691
cg03822267	0.01775408	20.8995315	1.693261	257.958117	cg07405126	0.0330347	13737.663	2.15423638	87605699.6
cg27508002	0.01775426	6.38052762	1.37862644	29.5302131	cg25035631	0.03303568	4.6984449	1.13271315	19.4889451
cg01726854	0.01775453	54363431.7	21.8865076	1.3503E+14	cg02673986	0.03303824	17.7009352	1.2603706	248.596013
cg16096311	0.01775456	6.45255881	1.38130484	30.1421626	cg08940505	0.03306886	61.5944645	1.39258092	2724.35016
cg06896857	0.01776258	3.6427E+12	149.826342	8.86E+22	cg26247263	0.03306897	2968.61252	1.90141718	4634785.23

cg15462247	0.01776353	847.362138	3.21447304	223371.789
cg10383424	0.0177747	13.2782336	1.56467682	112.682367
cg02158880	0.0177794	10.9470362	1.51312645	79.1986689
cg15019790	0.01778162	14.9002495	1.59599067	139.109482
cg25077778	0.01778165	10.2956345	1.49708809	70.8041763
cg04948483	0.01778471	1656.43664	3.60604722	760883.64
cg13003311	0.01778535	6.42526013	1.37971697	29.9220554
cg03852670	0.0177884	8.87050437	1.45882544	53.937809
cg25338254	0.01780706	10544.3451	4.95990054	22416420.2
cg25451082	0.01780761	18.9453934	1.66280508	215.856889
cg13989528	0.0178291	5840.01197	4.47209609	7626343.26
cg25153340	0.01783172	8740187.61	15.8038817	4.8337E+12
cg03877492	0.01783676	219.216156	2.53612667	18948.4712
cg19274820	0.01783903	335602.829	8.99473716	1.2522E+10
cg23363971	0.0178475	3.41307721	1.23597756	9.42500611
cg02060096	0.01785829	188655.697	8.12953423	4377984136
cg16645133	0.01786597	7.86507021	1.42712189	43.3455123
cg02718824	0.01786702	736182.428	10.2730239	5.2756E+10
cg09188212	0.01786846	8.748137	1.45349432	52.6523565
cg07701375	0.01787119	101485767	24.0091442	4.2898E+14
cg27298243	0.0178731	5.73748709	1.35145005	24.3581019
cg09001028	0.01787955	322.517475	2.70611117	38438.0076
cg05128038	0.01787957	1201475.67	11.1642472	1.293E+11
cg16458866	0.01788099	6.53227446	1.38186836	30.8789252
cg16464045	0.0178877	23.8883637	1.72761586	330.312967
cg18236745	0.01788957	67232.0381	6.7869696	666003712
cg25087487	0.01789231	7.85647048	1.4262924	43.2759289
cg13356253	0.01789673	8.04626952	1.43207385	45.2088788
cg11991516	0.01790037	3.32735613	1.22999815	9.00106944
cg03852056	0.01790564	196816.536	8.15502562	4750046266

cg00311035	0.03307081	11.8103281	1.21944924	114.382662
cg01389283	0.03307394	0.14104392	0.0232836	0.85439487
cg23076086	0.03309214	994.749493	1.74001194	568689.519
cg01706263	0.03309453	0.0870746	0.00922228	0.82213785
cg20693334	0.03309481	10.7732032	1.21011406	95.9098918
cg13284574	0.03310018	8.4350901	1.18652055	59.9658768
cg23696808	0.03310376	3419.2196	1.92028495	6088191.59
cg20170271	0.03310773	11.2819316	1.21440284	104.810345
cg18189994	0.03310961	6.29952612	1.15895744	34.2411446
cg17689707	0.03312246	4.74374975	1.13278677	19.8653112
cg21229079	0.03316153	7.45767493	1.17410035	47.3698141
cg20802616	0.03316233	7.49319884	1.17453617	47.8044272
cg04904385	0.03316607	6.76627196	1.16495771	39.2996551
cg19803043	0.03318044	51102987.9	4.12099728	6.3371E+14
cg10729312	0.03319393	0.10094793	0.01223434	0.83294108
cg26099580	0.03319935	23070.0611	2.22671785	239018931
cg03324851	0.03319972	349.778704	1.59475769	76717.0731
cg26615259	0.0332007	200.639773	1.52563112	26386.6657
cg21474786	0.03320538	9.6099028	1.19750132	77.1191067
cg23963351	0.0332067	1047050828	5.2287794	2.0967E+17
cg24274165	0.0332089	311179397	4.74605727	2.0403E+16
cg05085816	0.03323847	49215.8435	2.36007529	1026322871
cg24767968	0.03324618	6.85524151	1.16523339	40.3304065
cg26272575	0.03324658	49.254485	1.36283386	1780.11742
cg23069367	0.03325021	5.21657438	1.14017988	23.8669781
cg01074928	0.03325277	242.055208	1.54630093	37890.8934
cg14379630	0.03326402	18151.773	2.17734709	151324915
cg24313303	0.03326474	8.65704564	1.18678734	63.1490047
cg05172655	0.03327886	0.06957618	0.00597967	0.80955016
cg05892930	0.03328767	51.6466543	1.36681975	1951.5206

cg22593342	0.01790677	11.2004025	1.51574965	82.7636784
cg21155063	0.01791018	53474487.3	21.390655	1.3368E+14
cg18365406	0.01791405	4.48954551	1.29492163	15.5654353
cg04567307	0.01793256	8.04722823	1.43133053	45.2431363
cg05191488	0.01793892	22406.2495	5.59646274	89706666.2
cg02492791	0.01794471	8.22555532	1.43647005	47.1014067
cg07067280	0.01796049	4746005.37	14.0201488	1.6066E+12
cg16003274	0.01796296	20789.0583	5.51532825	78360693.3
cg08450280	0.01796977	13.1547102	1.55647787	111.178195
cg24868015	0.01797146	28.2778506	1.77497123	450.506928
cg06220725	0.01797291	971.4465	3.25734216	289717.277
cg14952889	0.01797583	7.0663E+10	72.8281873	6.8562E+19
cg12468273	0.01797726	6.64419647	1.38409686	31.8946947
cg00147339	0.01797916	4385292.87	13.8026194	1.3933E+12
cg05711542	0.01799731	6107518361	47.6717773	7.8247E+17
cg27662454	0.01800117	11.6678729	1.5239048	89.3358026
cg09365002	0.0180037	0.17544413	0.04148317	0.74200315
cg09962502	0.01804224	16064.8245	5.2473611	49182547.3
cg27610178	0.01804444	11354.9273	4.94404209	26078737.1
cg10560079	0.01804646	649.791895	3.02964111	139366.179
cg07245678	0.01805216	33.8034819	1.82646307	625.62195
cg11872076	0.01805369	43253.1205	6.21138563	301194056
cg14865516	0.01805715	276360.751	8.52827506	8955534837
cg27549166	0.01807181	1119330558	35.2396158	3.5554E+16
cg03970032	0.01808215	8.60997381	1.44470989	51.3124812
cg02013781	0.01808867	5.30674864	1.32993543	21.1751491
cg00994896	0.01809186	16063.6893	5.22920099	49346375.4
cg00982136	0.01809639	4.38341166	1.28710024	14.9283616
cg07408835	0.01809886	0.03521774	0.00219629	0.5647203
cg08199758	0.018101	6.11267457	1.36223183	27.4290981

cg14862722	0.03328816	0.14736104	0.02527237	0.85924984
cg21823502	0.03329221	0.18582936	0.03945607	0.87521525
cg15658824	0.0332926	44.1484904	1.34980718	1443.97603
cg09667582	0.03329536	23.7354053	1.2850111	438.416029
cg07697561	0.0332992	60120.3202	2.38955757	1512603400
cg18486906	0.03330006	9.53206415	1.19539129	76.0087913
cg17667988	0.03330735	354634.992	2.74850329	4.5758E+10
cg20735720	0.03331762	14.1800684	1.23327088	163.041505
cg02919799	0.03333374	41.8174923	1.34294331	1302.14184
cg24701966	0.03333446	562097.156	2.84517149	1.1105E+11
cg12778476	0.03333918	5.65347261	1.1465627	27.8761489
cg04134731	0.03334703	3893.14012	1.92007897	7893706.58
cg13414916	0.03335263	219644971	4.55010342	1.0603E+16
cg23796713	0.03336955	0.02697372	0.0009672	0.75225698
cg03786924	0.03337102	606.191037	1.65666206	221812.029
cg03289681	0.03337684	59.9761721	1.38047315	2605.73067
cg02006107	0.03337858	8.67599107	1.18546984	63.4961923
cg00930615	0.0333795	222.074344	1.53027727	32227.5024
cg10663408	0.03339283	0.00719373	7.63E-05	0.67826477
cg16749785	0.03340346	0.05546115	0.0038612	0.7966269
cg05896563	0.03344053	3.2622E+12	9.58004122	1.11E+24
cg01289902	0.03344096	140446.289	2.53326375	7786461318
cg16018130	0.03344803	687615.755	2.86789796	1.6486E+11
cg03496709	0.03345849	11.1246216	1.20769332	102.474034
cg07491796	0.0334682	113.5787	1.44840355	8906.44119
cg12563239	0.03348479	2081.59021	1.81751785	2384030.4
cg21287489	0.03348597	38.5368295	1.33044306	1116.23509
cg09904774	0.0334899	2217.72065	1.82616876	2693225.84
cg27248189	0.03350174	19.4149825	1.26069758	298.994424
cg09942293	0.03351164	24.0565253	1.28177273	451.496894

cg26737948	0.0181039	0.05433813	0.00485475	0.60819409
cg05155781	0.01811123	15613533.1	16.8958666	1.4429E+13
cg04401646	0.01811353	14451.6833	5.12793549	40728116
cg20739396	0.01812347	7496.28736	4.58157708	12265279.7
cg20919942	0.01813611	7.6686737	1.41528958	41.5523134
cg26617787	0.01813877	11.1797583	1.50917061	82.8183334
cg01119072	0.01813877	39.6310298	1.87255867	838.755309
cg23793040	0.01813903	5.95276424	1.35541965	26.1434916
cg24174608	0.01813929	123.787256	2.27381274	6739.02669
cg14965353	0.01814023	12136853.1	16.1300149	9.1322E+12
cg01826858	0.01814781	180.451187	2.42393685	13433.7785
cg18413449	0.01816583	6.62511513	1.3798725	31.8088449
cg17244462	0.01817119	650905.387	9.76665977	4.338E+10
cg13842258	0.01819107	7141827269	47.4459045	1.075E+18
cg13891121	0.01819122	35.2463289	1.83304311	677.727487
cg07177860	0.01820532	25.7555841	1.73721938	381.845909
cg12938159	0.0182063	6.4635586	1.37334057	30.4204147
cg14761923	0.01820886	2000.29331	3.64013448	1099182.83
cg24720717	0.01821364	3.18917646	1.21786092	8.35140227
cg22534509	0.01821379	9.0438366	1.45387261	56.2573226
cg19808978	0.01822464	4.12881794	1.27235425	13.3981063
cg01574381	0.01822512	4249103.23	13.3629302	1.3511E+12
cg00503383	0.01822604	5.99880505	1.35568638	26.5442382
cg13303952	0.01822892	238330.028	8.18911803	6936180648
cg25285053	0.01822977	0.15635933	0.03350497	0.72968992
cg17217295	0.01823111	8922.62644	4.68676102	16986840.6
cg02348830	0.01823275	50442.7553	6.28889493	404597563
cg15583193	0.01823484	0.1744826	0.04094961	0.74345475
cg25923056	0.01823627	10.9439045	1.50118411	79.7830487
cg05232889	0.01824766	6.68028511	1.38028602	32.3311316

cg15377585	0.03351328	7.00247866	1.16404084	42.1245592
cg25522149	0.03352753	76.8173246	1.40285251	4206.35905
cg11057378	0.03352772	15093.14	2.11749068	107581524
cg19813688	0.03353809	5.08E-08	9.57E-15	0.27020446
cg02418535	0.03355071	194.640442	1.50736565	25133.1863
cg03553278	0.03355997	5.28061913	1.13822144	24.4986937
cg05797937	0.03356273	141.338924	1.46982331	13591.2195
cg02699635	0.03356517	493.798742	1.61991568	150524.624
cg04528060	0.033571	3.43863296	1.10078227	10.7416306
cg14323910	0.03360924	0.33975224	0.12551085	0.91969403
cg16621987	0.03361129	1309.47379	1.74456114	982895.679
cg23710492	0.03361151	26.9014374	1.29079397	560.652864
cg03059073	0.03361175	0.1458818	0.024707	0.86135515
cg12564291	0.03364156	3.5837E+12	9.3633726	1.37E+24
cg21644826	0.03364874	4449168.52	3.26726617	6.0586E+12
cg25262261	0.03365867	0.11411684	0.01540126	0.84555746
cg19257562	0.03369128	45.0669935	1.34136042	1514.15971
cg05864627	0.03370947	0.05704611	0.00405746	0.80204302
cg05485462	0.03371641	13.932146	1.22483432	158.474243
cg06949933	0.03371803	8.52352958	1.17934279	61.6025784
cg22770592	0.03372571	10041.2175	2.0318995	49621572.5
cg24745816	0.03372629	5.41217419	1.13873917	25.722861
cg08315174	0.03372673	26.150724	1.28545045	532.000564
cg00123512	0.03373289	118417896	4.17712307	3.357E+15
cg24354901	0.03375631	28649.1597	2.19901124	373247003
cg24129382	0.03375648	5.64381836	1.14210719	27.8894012
cg14927519	0.03375819	7.90165817	1.17197988	53.2741243
cg11884274	0.03376412	11.9252965	1.20951216	117.578558
cg19659689	0.03376587	427.679671	1.59181647	114906.4
cg19691267	0.03376775	1373948.69	2.95749215	6.3829E+11

cg26672088	0.01825158	57637895	20.7390132	1.6019E+14
cg04683119	0.01825317	106405.97	7.12667186	1588712180
cg02817981	0.0182579	9.29496577	1.45962878	59.1906586
cg07187103	0.01826434	10210977.7	15.4388512	6.7534E+12
cg06206902	0.0182644	5.32502657	1.32791771	21.3536635
cg13596049	0.01827135	32.4830428	1.80415902	584.842054
cg10561989	0.01827462	12.2759999	1.52969558	98.516447
cg05281894	0.01827552	8.83434786	1.44670179	53.9473325
cg11449372	0.01827686	232.844542	2.51884354	21524.3939
cg14961031	0.01827696	48.5454635	1.93103109	1220.41641
cg06646636	0.01827839	27.2676969	1.75112318	424.600224
cg14441271	0.01827947	9.30234182	1.45932366	59.2970329
cg21436938	0.01828513	11.1190684	1.50397122	82.2048188
cg14157855	0.01828611	15.8639009	1.59728293	157.557154
cg02515030	0.01829069	342.090493	2.68700829	43552.4914
cg01347529	0.01830428	13.7528488	1.55857171	121.355244
cg14520423	0.01831382	103.009829	2.19097692	4843.05642
cg05861291	0.01831927	10.3168507	1.48416542	71.7153273
cg07378350	0.01832122	11.571752	1.51321981	88.4904123
cg04108127	0.01832254	8644.18175	4.63408023	16124424.8
cg08219218	0.01832262	0.00516557	6.50E-05	0.41033305
cg06164260	0.01832889	64.259443	2.02190769	2042.26733
cg09232851	0.01832992	0.15966393	0.03476665	0.73324793
cg14373380	0.01833152	1264.69821	3.34622518	477989.818
cg03304714	0.01833787	8.4851E+11	103.903821	6.93E+21
cg17163363	0.01834168	226.169241	2.5001782	20459.5519
cg04897244	0.01834382	36769.7981	5.91068156	228741481
cg15195412	0.0183446	15.5694507	1.59039756	152.419622
cg15641348	0.01835366	215.250032	2.47821696	18695.9323
cg20053110	0.01835512	4.41970926	1.28537367	15.1970049

cg02913948	0.03378517	129131005	4.18378534	3.9856E+15
cg18101225	0.03378669	18.7857091	1.25200688	281.869751
cg26896499	0.03379291	71.0486722	1.38616611	3641.63701
cg00658394	0.03381019	63.5732064	1.37390156	2941.66095
cg03541791	0.03381178	741.086689	1.65779026	331290.088
cg01815671	0.03381749	4.81920072	1.12777818	20.5933189
cg22760295	0.03382269	132751.755	2.46374244	7152950773
cg24174608	0.03382854	166.373417	1.47811414	18726.6416
cg18927077	0.03382947	11.8756645	1.20811145	116.737084
cg23445859	0.03383805	4.34478885	1.11870163	16.8741956
cg03512414	0.03384071	10.7650825	1.19891665	96.6597651
cg10561989	0.03384246	14.3061426	1.22520138	167.046594
cg07343367	0.03384389	55047.4722	2.30065593	1317113160
cg08290108	0.03384659	1065.21963	1.70230176	666563.871
cg16581360	0.03385808	1006011.81	2.86897203	3.5276E+11
cg06485521	0.03386246	191.784573	1.49287429	24637.9234
cg23739036	0.03386825	6.62E-09	1.84E-16	0.23808245
cg20902195	0.03389807	27047516.5	3.67446657	1.991E+14
cg14042889	0.03390065	6.35478411	1.15096743	35.0863804
cg22573528	0.03390476	0.28833688	0.09138079	0.90979905
cg03939371	0.03390809	6884.69835	1.95732194	24216287.7
cg13075709	0.03391611	8036302.62	3.34554468	1.9304E+13
cg23736297	0.03393985	43.3793752	1.33093563	1413.87017
cg09180877	0.03393991	0.12879966	0.01937873	0.85605979
cg01191114	0.03394056	45888.5187	2.2567595	933088418
cg18444267	0.03395527	21.7025139	1.26253213	373.059107
cg19971773	0.03397199	416.34398	1.57833969	109825.73
cg24810594	0.03397971	2.9741E+10	6.19507266	1.43E+20
cg21810188	0.03398807	4.38752958	1.11825295	17.214724
cg16421340	0.03398894	14.9228821	1.22663812	181.546952

cg09497409	0.01835825	7.0109256	1.38951076	35.3743771
cg00013451	0.01835892	23.6265687	1.70599537	327.207658
cg24208206	0.01836141	929.948277	3.17206832	272630.887
cg09600247	0.01838224	240.257549	2.52184864	22889.4348
cg24809270	0.01838474	6.1317E+10	66.0876491	5.689E+19
cg22254127	0.01838531	2.736E+12	125.42564	5.97E+22
cg14781919	0.01838705	2.0536E+13	176.164003	2.39E+24
cg21988950	0.01838787	0.18579316	0.04585378	0.75280808
cg09877947	0.01838943	9.36034063	1.45830218	60.0808101
cg26795340	0.01839406	8.40612184	1.43198964	49.3459468
cg19946982	0.01839566	21.7629917	1.68112836	281.732091
cg22412649	0.01839801	8.54522634	1.43587242	50.8547224
cg21211321	0.01839815	0.21892324	0.06191975	0.7740242
cg21606036	0.0184053	10.6554326	1.49013367	76.1933289
cg04206724	0.01841198	1650.25255	3.48540517	781353.484
cg20311863	0.01841466	4.82752675	1.30381204	17.87452
cg06580065	0.01841509	15242.0317	5.06844781	45836425.5
cg11073923	0.01841958	5233.50201	4.23181476	6472292.59
cg04981940	0.01842128	8.51303678	1.4344503	50.5223465
cg02025938	0.01842302	632951.552	9.48961585	4.2217E+10
cg16774354	0.01842745	114.296881	2.22133391	5881.05055
cg02999463	0.01842765	78832.8896	6.67875588	930506309
cg00364457	0.0184305	22.0968366	1.68415763	289.919531
cg13731133	0.01843346	141.984134	2.30349814	8751.68678
cg25100532	0.01844408	46366471.9	19.5089716	1.102E+14
cg04667246	0.01844697	5.9168E+10	64.9737713	5.3881E+19
cg14923274	0.01845251	133303.051	7.28135192	2440440131
cg14140118	0.01845328	6.92776443	1.38489946	34.6551655
cg20696432	0.01845768	12.1519759	1.52209953	97.0176488
cg14443362	0.01846091	29.9914407	1.77175839	507.680124
cg08376992	0.03399663	246.41942	1.51586606	40057.9789
cg23694882	0.03400804	61.9984912	1.36548196	2814.98623
cg06349174	0.03402622	23614276.4	3.59602006	1.5507E+14
cg19292008	0.03402792	7.15344783	1.15987077	44.1185495
cg15342134	0.03403775	8.27690315	1.17256841	58.4248432
cg02783970	0.03404954	831093472	4.69166461	1.4722E+17
cg06874326	0.03405517	298.857111	1.53546836	58168.2926
cg11258089	0.0340701	6.14953868	1.14627154	32.9911584
cg20579480	0.03407442	10.2607777	1.19117436	88.3863542
cg12446939	0.03408168	959.12209	1.67468127	549307.622
cg15145873	0.0340886	26.067932	1.2773086	532.00697
cg23127201	0.03409498	357380.309	2.6100353	4.8934E+10
cg05852040	0.0341127	65.5623649	1.36815614	3141.764
cg23734074	0.03411413	60804.8681	2.28276565	1619628358
cg11005899	0.03411578	26987929.3	3.6037569	2.0211E+14
cg03216729	0.03412803	176557.698	2.47043671	1.2618E+10
cg25413906	0.03412999	40410215.5	3.70965162	4.402E+14
cg08450280	0.03413108	11.9784398	1.20423263	119.148923
cg01117697	0.03414328	15.1838989	1.2255852	188.11486
cg20984085	0.03415283	4.76128019	1.12369014	20.1744131
cg23778358	0.03415981	0.21731456	0.05292897	0.89224525
cg04340651	0.03416279	6.9491076	1.15577805	41.781462
cg22669260	0.03417689	202353755	4.16570262	9.8296E+15
cg05085336	0.03419109	0.15269553	0.02682159	0.86929681
cg10104451	0.03419748	26.0238284	1.27480933	531.247795
cg02917792	0.03420931	59155038.4	3.78913286	9.2351E+14
cg11803389	0.03423377	10.8643927	1.19395685	98.8603797
cg03003689	0.03423412	5980.36615	1.9083283	18741418.5
cg16198723	0.03423619	11.5927042	1.19969119	112.021153
cg25446197	0.03425996	1674788.66	2.89521398	9.6881E+11

cg02400092	0.01846237	20.554385	1.66262155	254.106379
cg04478075	0.01846661	6.59590219	1.37326413	31.6806687
cg15734651	0.0184742	45.8698149	1.90231403	1106.04237
cg02480298	0.01848102	15.354774	1.58246998	148.988029
cg21016004	0.01848471	72.9101419	2.0557616	2585.84887
cg26572207	0.01849563	7.80258527	1.4120126	43.116001
cg01724067	0.01849611	41923.59	5.97392285	294209926
cg21491013	0.01849856	0.13446594	0.02532465	0.71397193
cg11177450	0.0185097	1381.33293	3.36553636	566946.973
cg06408864	0.01851485	24214784.7	17.3397694	3.3816E+13
cg26465611	0.01851801	10.3068163	1.47903613	71.8241163
cg24121733	0.01851826	10.8552316	1.49195055	78.9812062
cg01910481	0.01852038	0.11806432	0.01994797	0.69877706
cg08236022	0.01852208	6.35787945	1.3638104	29.6394801
cg22324029	0.01852217	15.9398695	1.59115907	159.681986
cg20890243	0.01852277	21.6708288	1.67526646	280.328432
cg12149795	0.01852981	9.90305187	1.46887093	66.7658639
cg22731763	0.01853278	0.04361027	0.00321574	0.59142128
cg20940164	0.01853351	2347280.1	11.6985858	4.7097E+11
cg21267231	0.01854246	23.4768913	1.69716534	324.755881
cg00014272	0.01854758	19718.6624	5.24421931	74143666.6
cg21045608	0.01854799	10.8516333	1.49111812	78.9729161
cg03656968	0.01855186	6.53004532	1.36939448	31.1389396
cg18580265	0.01855895	23.1494287	1.69255345	316.619868
cg09488991	0.0185612	39797280.1	18.7392932	8.4519E+13
cg09386303	0.01856469	21.7827191	1.67518051	283.245207
cg24168884	0.01857072	19.8819393	1.6495534	239.635474
cg15371881	0.01858416	9.77011805	1.46426073	65.190034
cg03133987	0.01858539	30.8915375	1.7752087	537.563323
cg05986853	0.0185877	7.99376089	1.41584303	45.1322722

cg19630883	0.03426255	494839.714	2.64440434	9.2598E+10
cg26833120	0.03428448	11.617445	1.19914911	112.550663
cg12552766	0.0343082	1969658.57	2.91971895	1.3287E+12
cg05768582	0.03431814	57433.3413	2.24697855	1468010759
cg27022535	0.03432609	0.01290769	0.00022971	0.72528723
cg12525224	0.0343568	58761383	3.73612438	9.2419E+14
cg22896904	0.03437891	8.7093E+17	20.8816218	3.63E+34
cg04228628	0.0343875	7741.57207	1.93157704	31027464.6
cg15578015	0.03439089	1780.30091	1.73350084	1828364.45
cg10685559	0.03439533	3.95E-05	3.28E-09	0.47468551
cg16039972	0.03439662	0.00023029	9.81E-08	0.54040552
cg13736376	0.03442268	9.16088939	1.17638359	71.3388858
cg07207726	0.03447788	145.662004	1.43895446	14745.025
cg08402572	0.03448109	1600.47099	1.71411144	1494364.56
cg26247841	0.0344926	5.20565394	1.12794822	24.024891
cg04422150	0.03450549	0.02079823	0.00057371	0.75397863
cg24476497	0.03452311	0.07416366	0.00664753	0.82741294
cg23061725	0.03452488	7.93516227	1.16279056	54.1514547
cg22270364	0.03452608	5.15402883	1.12681037	23.5745197
cg21534578	0.03453632	14.7863915	1.21650869	179.725288
cg19430489	0.03455636	4.29870702	1.11176859	16.6211587
cg26594902	0.03456629	2864880.82	2.94309291	2.7887E+12
cg10330832	0.03457169	306054.799	2.50110806	3.7451E+10
cg06550200	0.03457889	48.8232757	1.32583633	1797.8933
cg10626541	0.03458616	5.0469E+12	8.33644084	3.06E+24
cg02067788	0.03458742	100014.038	2.30396783	4341557070
cg01239651	0.03461599	14.8128052	1.21532696	180.543348
cg20313969	0.03461777	8.07537351	1.163116	56.0663402
cg14980255	0.03461931	54.633527	1.33558617	2234.84065
cg04117874	0.03462891	4.8568E+12	8.26017934	2.86E+24

cg16884841	0.01858939	25.5314954	1.71934656	379.130812
cg09409956	0.01859679	5.87324882	1.34453156	25.655814
cg02317397	0.01859969	0.05712785	0.00526682	0.6196508
cg19459508	0.01860188	3.5225E+12	125.204345	9.91E+22
cg21997510	0.01860333	0.17066291	0.03914205	0.74410578
cg24032330	0.01860759	0.02419652	0.00109056	0.53685427
cg00326908	0.01862128	65.9007559	2.01297261	2157.46086
cg01059295	0.01862527	0.14997106	0.03087707	0.72841496
cg08190044	0.01862699	12.2729975	1.52005958	99.0924751
cg02466544	0.01863108	31.9525189	1.78326912	572.523493
cg02722618	0.01863484	7.94E-05	3.05E-08	0.20676306
cg10370375	0.01863765	0.04753679	0.00375754	0.60139047
cg16829809	0.01863822	19.7088469	1.64479808	236.161904
cg08938402	0.0186384	7.0372E+13	204.867617	2.42E+25
cg23167594	0.01864302	7.73942541	1.40707891	42.5695427
cg13850380	0.01864885	7.51341516	1.40002007	40.3218558
cg25424488	0.01865538	165494.331	7.42194755	3690186910
cg13997647	0.01865887	1.9965E+11	76.6763506	5.20E+20
cg12768250	0.01866037	647382192	29.4735976	1.422E+16
cg26397391	0.01866128	65.3041693	2.00754476	2124.30358
cg05019913	0.01866147	111342810	21.9722984	5.6422E+14
cg09257092	0.01866305	2981649.27	12.0121172	7.4011E+11
cg03924800	0.01867368	2215066.4	11.4188134	4.2969E+11
cg00172803	0.01867494	11.6674107	1.50601445	90.389885
cg06390536	0.01868818	4.0164845	1.26062129	12.7969818
cg05434115	0.01868925	20.0697615	1.64800654	244.413671
cg26724572	0.01869012	0.0194162	0.00072686	0.51865361
cg19528963	0.01869136	6.89816893	1.37940504	34.4965642
cg24313303	0.01869674	8.07026077	1.41582149	46.0009326
cg08172787	0.01869696	4.4864E+11	87.131155	2.31E+21

cg19430553	0.03463024	6.7056837	1.14744271	39.1881819
cg24938064	0.03464476	737.679218	1.61087214	337811.185
cg24908234	0.03464535	1871552.4	2.83684968	1.2347E+12
cg15734651	0.03467225	61.5117693	1.34558599	2811.93309
cg11601666	0.03469385	1291321.02	2.75221172	6.0588E+11
cg09299055	0.03469448	7.15278909	1.15206179	44.4094165
cg06530441	0.03469464	5.50625203	1.13057855	26.8170763
cg15787985	0.03469955	21.3561417	1.24628915	365.954232
cg16908156	0.03470168	1768.41631	1.71210246	1826582.42
cg24129356	0.03470897	7.25162658	1.15303029	45.6068574
cg00028034	0.03471377	15.6616202	1.2185552	201.292766
ch.12.262555	0.03471543	4.89E-05	4.87E-09	0.49012405
cg22942704	0.03471624	88.2392749	1.37963756	5643.63415
cg09455126	0.03472421	0.13662751	0.02153471	0.86683687
cg19046253	0.03473105	50.4698928	1.32497229	1922.46291
cg15395193	0.03473848	1903080051	4.6294088	7.8233E+17
cg17176108	0.03475182	5606.47537	1.85609892	16934747.2
cg09788352	0.03476056	161.54489	1.43922883	18132.4547
cg22361075	0.03478664	34707.6482	2.11116163	570596217
cg17513770	0.03479136	0.08758523	0.00912903	0.84030515
cg08039823	0.03479218	1.2974E+11	6.2226975	2.70E+21
cg17453374	0.03480247	21.1442417	1.24339529	359.563012
cg00131758	0.03481365	11.0157024	1.18667738	102.256689
cg10548700	0.03482024	0.00075866	9.61E-07	0.59915765
cg03221676	0.03483241	5.7905E+10	5.84430768	5.74E+20
cg13586425	0.03483395	18.1193247	1.2291922	267.094055
cg17162095	0.03488736	0.00562239	4.57E-05	0.69236774
cg16580499	0.03490521	3.66837461	1.09648568	12.2728208
cg24255928	0.03491133	26.5191804	1.26136266	557.545385
cg11530564	0.03491719	37.2046383	1.29183947	1071.48384

cg24775108	0.01869894	1.3716E+13	153.927496	1.22E+24
cg10012711	0.01869966	5.55160061	1.3302704	23.1684245
cg23280754	0.01869968	7.45910725	1.39731889	39.8178837
cg02668708	0.01870349	469604.173	8.79319577	2.5079E+10
cg10718991	0.01871237	14.3453754	1.55768627	132.11248
cg17399362	0.01871247	5.31701528	1.32054314	21.4083514
cg27176129	0.01871716	9.6121846	1.4571729	63.4064035
cg21801378	0.01872152	16.9858338	1.6018078	180.120579
cg25751895	0.01872309	54.798524	1.94627579	1542.88423
cg18098743	0.01872953	5401.39372	4.17491517	6988178.9
cg21490342	0.0187321	11705.5897	4.74707313	28864276.2
cg20147819	0.0187323	24.5871587	1.70302984	354.972273
cg02606566	0.01873906	11785465.5	14.9748104	9.2754E+12
cg07190921	0.01874488	18.1778383	1.61920819	204.071229
cg14950751	0.01875029	13.6799172	1.54434308	121.177824
cg08826738	0.01875625	33.1036771	1.78830536	612.788767
cg12738347	0.01875785	4.96441752	1.30488244	18.8870971
cg00731608	0.01876603	22.4553264	1.67630255	300.805894
cg16353899	0.01876635	20016.6993	5.17759317	77385039.7
cg17368254	0.01876747	8.90503168	1.43765046	55.1591584
cg00856825	0.01876986	5.78021706	1.33807669	24.9693531
cg16368008	0.01877143	0.17870201	0.04250064	0.75138652
cg08388507	0.01877442	0.19394364	0.04938243	0.76169061
cg17538898	0.01878054	741623.591	9.41881027	5.8394E+10
cg12233363	0.01878055	8.20604013	1.41799714	47.488879
cg23044079	0.01878129	17.5698168	1.60889784	191.869525
cg07545140	0.01878417	694343.224	9.31322745	5.1766E+10
cg16264966	0.01878532	5.58261815	1.33012418	23.4306134
cg09862200	0.01878571	14.2120511	1.55313751	130.04798
cg01472538	0.0187923	31.3825497	1.77096745	556.116617
cg14019947	0.03493242	9.536E+11	7.03535392	1.29E+23
cg20941820	0.03493674	4.05678909	1.10408514	14.9060404
cg23271831	0.03495046	7.21853838	1.14984159	45.3169349
cg00910297	0.03495233	810723264	4.25802527	1.5436E+17
cg04468551	0.03495403	19.0752315	1.23147263	295.471005
cg19720565	0.03496516	12.4070673	1.19445853	128.874562
cg23130254	0.0350026	13.0977059	1.19844384	143.143879
cg11897145	0.03500616	961.64716	1.62129625	570386.356
cg08769189	0.03500745	25.7888003	1.25686418	529.144067
cg19522294	0.03500959	2973616.89	2.85297611	3.0994E+12
cg25495844	0.03501193	3719.24635	1.78272816	7759339.69
cg21653581	0.03503507	3253.95555	1.76436697	6001147.66
cg00290086	0.03503587	8.88956012	1.1657684	67.7872889
cg00263592	0.03504021	10.5966133	1.18017121	95.1456983
cg11566061	0.0350486	7.2220535	1.14874342	45.4044443
cg00129981	0.03505232	0.0235193	0.00071951	0.76879234
cg24654028	0.0350683	27.6789071	1.26183279	607.150098
cg24517380	0.03508389	102.281739	1.38228564	7568.30128
cg04131468	0.0350909	9.64578976	1.17172403	79.4054382
cg02494572	0.03509916	13.2116597	1.19765207	145.741787
cg11658047	0.03510393	90.422883	1.36979003	5969.0154
cg09980339	0.03510938	39921189.4	3.3944074	4.6951E+14
cg09930748	0.03510974	11653.1549	1.92281422	70623577.2
cg17951138	0.03511556	6.14943918	1.13515887	33.3130483
cg20583945	0.03513075	8.07972543	1.1568083	56.4328274
cg00282706	0.03513757	0.09075872	0.0097363	0.84602423
cg27566947	0.0351454	61.7268058	1.33258125	2859.26172
cg20700235	0.03514998	1431.87602	1.65848922	1236226.87
cg27147000	0.03515459	6.90254745	1.14390973	41.651155
cg07602841	0.03516905	6.1061418	1.13404024	32.8779935

cg23873398	0.01879305	758915.493	9.44364334	6.0988E+10
cg13955572	0.01879536	218.803386	2.44356556	19592.2395
cg18353597	0.01881233	6853381.67	13.5738546	3.4602E+12
cg00303183	0.0188138	9.68339498	1.45671185	64.3697229
cg07962315	0.01881475	8.70504716	1.43120952	52.9467174
cg06460983	0.01881505	10.1730293	1.4686364	70.4670847
cg13736376	0.01881534	7.92742226	1.40917954	44.5961795
cg20026798	0.0188189	11.071466	1.48927751	82.306594
cg26168907	0.01881892	5.66692665	1.33288939	24.0935655
cg26846647	0.01882582	2.7788E+10	53.6473522	1.4394E+19
cg01003803	0.01882788	9.56547202	1.45343285	62.9532042
cg19306380	0.01883366	7.03712616	1.38129245	35.8513107
cg12457341	0.01883744	3389.92156	3.83995734	2992629.14
cg19237879	0.01884547	14.092234	1.54923545	128.186493
cg18536827	0.01885161	0.19482536	0.0497508	0.76294094
cg02446170	0.01885179	147950.276	7.16556434	3054788603
cg07478122	0.01885531	0.13575212	0.02564097	0.71871837
cg17935677	0.01885586	23523.7899	5.28467084	104712045
cg01770333	0.01887115	262.054286	2.51030346	27356.2338
cg10015871	0.01887334	7.14308981	1.38395603	36.8680296
cg11466815	0.01887428	705090865	28.9932258	1.7147E+16
cg04566778	0.01887732	7255632.11	13.6043092	3.8697E+12
cg10759590	0.01887894	92.1381303	2.1114804	4020.60803
cg06927327	0.01890114	0.00013617	8.06E-08	0.2300551
cg07333223	0.01890409	700842.045	9.22250074	5.3259E+10
cg12217560	0.01890674	7.94524386	1.40783092	44.8398307
cg13558810	0.01890714	28.9422024	1.74261668	480.685795
cg23244913	0.01891471	4.44791053	1.27918438	15.4660331
cg06148175	0.01892027	32.6102474	1.77670413	598.539858
cg20950465	0.01892377	4.7685602	1.29383133	17.5750624
cg10290107	0.03517525	38275428.2	3.36468396	4.3541E+14
cg00541638	0.03519069	9.99059433	1.17323225	85.0743537
cg03019000	0.03522682	8.96748374	1.16399745	69.0858595
cg16572908	0.03523475	11563.2598	1.91036899	69991179
cg08599266	0.0352487	1175793227	4.23561846	3.264E+17
cg10424417	0.03525283	6.60885408	1.13937765	38.3340435
cg20028470	0.03527729	17.1573021	1.21658468	241.966731
cg21445527	0.03530435	159.121882	1.41760389	17860.9648
cg12950012	0.03530707	1127933.98	2.60928711	4.8758E+11
cg00846114	0.0353203	8.49399567	1.15845623	62.2794029
cg16996661	0.03532044	30991813.3	3.27373659	2.9339E+14
cg05329798	0.03532474	368.500266	1.50104422	90465.3204
cg23642078	0.03532671	62.5339507	1.32871124	2943.07362
cg11472173	0.03533845	289520948	3.81056419	2.1997E+16
cg04439516	0.03534336	336987.668	2.39549405	4.7406E+10
cg19655952	0.03534468	13.2694683	1.19415584	147.450427
cg05708074	0.03536265	0.00010958	2.24E-08	0.53526561
cg19306970	0.03537241	9.96810773	1.1705604	84.8851301
cg16856833	0.03538272	8.67409885	1.15933459	64.899289
cg03100639	0.03538942	12.42135	1.18807498	129.865487
cg11154838	0.03539188	6891.2328	1.83021062	25947335.7
cg23657870	0.03539394	6226.33418	1.81738936	21331277.8
cg27660248	0.03540098	32347.9666	2.03338736	514604822
cg10812348	0.03540395	2045.24761	1.68352356	2484692.16
cg01193217	0.0354043	8.0993145	1.15364327	56.8623743
cg16681199	0.03541144	16.8719622	1.21284159	234.707575
cg00357238	0.03541274	191310.898	2.29434765	1.5952E+10
cg07194846	0.03541856	31.7747631	1.26626359	797.334436
cg16721449	0.03542044	8.99E-12	4.58E-22	0.17625641
cg18235937	0.03542651	18.4199309	1.2198621	278.141156

cg17603321	0.01892518	31681.4957	5.52351092	181717242
cg07356681	0.01892565	15352.7113	4.90137989	48089670.4
cg12830839	0.01893593	2.9254E+14	242.375737	3.53E+26
cg17347389	0.01894398	6.65567696	1.36660169	32.4147381
cg07138603	0.01894551	4.23701042	1.26857859	14.1514743
cg18603154	0.01894617	53.6023159	1.92708393	1490.96167
cg17952262	0.01895432	6.53425762	1.36227705	31.3420259
cg14421548	0.01895444	20.0003872	1.63788751	244.226472
cg21127268	0.01895568	5.51311018	1.32465665	22.9451034
cg13707690	0.01895686	0.0050005	5.98E-05	0.41788917
cg11432962	0.01896786	6.55724706	1.36282234	31.5503258
cg07261225	0.01896826	75961212	19.8239325	2.9107E+14
cg16677112	0.01897045	4.38100468	1.27525431	15.0504899
cg27173965	0.01897113	10.1739377	1.46493432	70.6577811
cg05947740	0.01897309	13.0393967	1.52594876	111.423051
cg27587033	0.01897614	0.00182426	9.39E-06	0.35425053
cg05654163	0.01897965	6.72759618	1.36837279	33.076184
cg04402803	0.01898034	16.6081652	1.58770102	173.729906
cg02822958	0.01898185	109.78581	2.16616414	5564.17861
cg18845797	0.01898368	0.17248317	0.03972345	0.74893914
cg02329038	0.01898368	5.16014297	1.30987924	20.3278857
cg03716942	0.01898762	1580299080	32.5775832	7.6658E+16
cg19422030	0.01899472	5.63514086	1.32881407	23.8971075
cg04594090	0.01899549	7.153E+10	60.903645	8.4011E+19
cg11323506	0.01900435	6.71048039	1.36734982	32.9327189
cg02918146	0.01901244	5.26877495	1.31393692	21.127338
cg10474377	0.01901334	105.724155	2.15056431	5197.51816
cg06746893	0.01902294	0.19525686	0.04985534	0.76471738
cg21091841	0.01902468	11.612428	1.49579587	90.1516622
cg24603803	0.01903453	54.6182557	1.9282834	1547.05156

cg00724111	0.03549427	12.3489691	1.18601402	128.579456
cg27477990	0.03550667	307933.318	2.35592215	4.0249E+10
cg17856005	0.03552079	116.633791	1.38038178	9854.83977
cg02951971	0.03554443	11659.3152	1.88353925	72172444.2
cg02786912	0.03555487	6.56774808	1.13560613	37.9843978
cg25731359	0.03555808	8.81277474	1.15835059	67.047921
cg10322499	0.03556014	136.696171	1.39393587	13405.0954
cg07218357	0.03556871	10.4410368	1.17154442	93.0525953
cg23684878	0.03557532	3.98295246	1.09771598	14.4517439
cg05146399	0.03557638	0.11192206	0.01452067	0.86267004
cg17237881	0.03557886	2412.21908	1.69090011	3441244.63
cg19800427	0.03557987	8.66438157	1.15674703	64.8988117
cg09597767	0.03558438	0.00635774	5.68E-05	0.7110678
cg21988950	0.03558711	0.1943761	0.04219214	0.89547637
cg22001110	0.03559616	16645.5764	1.92452949	143970365
cg23398241	0.03560627	104.09838	1.36704002	7926.96083
cg06664023	0.03560944	1.3471E+10	4.80377355	3.7777E+19
cg16697493	0.03562809	174.061542	1.41435988	21421.2951
cg16774354	0.03563849	488.97	1.5155137	157762.784
cg00956573	0.03563939	376849.719	2.36789085	5.9976E+10
cg18793200	0.035651	1568.34231	1.63810405	1501551.51
cg21870662	0.03565413	744.741458	1.55811992	355967.364
cg05031240	0.03565431	13242.8346	1.88982789	92798221.7
cg22999786	0.03566161	31.1174302	1.25911838	769.025757
cg15372508	0.03566361	8.38399893	1.15314449	60.9563143
cg02811627	0.03566557	60.4784161	1.31635694	2778.60716
cg24633648	0.03566943	663.672823	1.54534687	285024.433
cg26928531	0.03567144	47.8051724	1.29563026	1763.87862
cg10537847	0.03567224	632183.003	2.44614242	1.6338E+11
cg08093097	0.03569291	6169.02138	1.79239763	21232356.1

cg12167564	0.0190421	11.5612617	1.49426662	89.4504159
cg02170312	0.0190427	34505.8217	5.55394663	214379397
cg17884201	0.01904568	0.08719469	0.0113451	0.67014992
cg07535060	0.01904998	37.7891446	1.81444065	787.030121
cg06121514	0.01905176	0.13314341	0.02467575	0.71840449
cg02257405	0.01905595	15.9445851	1.57478043	161.438249
cg18480675	0.01905713	15.5270398	1.56790691	153.764846
cg15363487	0.01906219	10371.1072	4.55398902	23618823.7
cg12160586	0.01907016	0.08094536	0.00989292	0.66230721
cg07706687	0.01908202	887.529337	3.04049986	259071.981
cg14609668	0.01909456	24.5275165	1.68857017	356.277209
cg04080724	0.01910098	284.06834	2.52100338	32009.0098
cg16572908	0.01910718	6629.45497	4.22017028	10414194.3
cg09871079	0.01910869	218.574367	2.4144447	19787.0565
cg04707408	0.01913156	6.71762278	1.36527936	33.0529101
cg09265173	0.0191318	997753.816	9.56368862	1.0409E+11
cg17349515	0.01913498	0.14571648	0.02908943	0.72993159
cg10676604	0.01913767	21.6809368	1.65327947	284.321573
cg22708944	0.01914227	0.19793926	0.05105102	0.76746654
cg08360738	0.01914251	0.01100809	0.00025316	0.47866987
cg19972696	0.01914929	9.25200565	1.43822653	59.5174729
cg19160754	0.01915201	0.08736721	0.01136579	0.67157935
cg20470857	0.01915572	9.93064071	1.45480273	67.7876271
cg20556988	0.01915585	0.19771642	0.05093771	0.76744282
cg18792146	0.01915718	4.23152176	1.26560756	14.1479689
cg11090139	0.01915929	19201.0144	5.00438257	73671217.3
cg21433558	0.01916664	5.07051658	1.3034064	19.725343
cg09337254	0.01917321	20.5939975	1.63821513	258.88708
cg04079538	0.0191737	53.4972012	1.91432765	1495.01604
cg10506057	0.01917588	0.13207305	0.02427024	0.718711

cg01268541	0.03572835	5.98564045	1.12673715	31.7979145
cg06838879	0.03572962	10.4778099	1.16958429	93.8662574
cg24498692	0.03573526	0.09538859	0.01064169	0.85503133
cg02707152	0.03576809	26.9840191	1.24493855	584.878096
cg19540943	0.03577392	11675738.1	2.94893017	4.6228E+13
cg08798701	0.03578904	0.00016832	5.04E-08	0.56168347
cg02626657	0.03579693	7.85461558	1.14652241	53.8105363
cg07895149	0.03580915	8.65138826	1.15374129	64.872879
cg09200186	0.03582154	1134.13561	1.59318787	807352.103
cg19209269	0.03582894	0.03346223	0.00140201	0.79865157
cg25363886	0.03584446	334886592	3.66013315	3.0641E+16
cg05912499	0.03585374	66.0744031	1.31892242	3310.14673
cg05191488	0.03585745	23339.0795	1.94287382	280364389
cg03660115	0.03585837	7.02190223	1.13734277	43.352903
cg09125320	0.03586676	9.00448186	1.15606554	70.1350317
cg13325231	0.03587241	9.31293934	1.15856431	74.860617
cg00103132	0.03588079	11869909	2.92634488	4.8147E+13
cg13071386	0.03588331	7.40819956	1.14108426	48.0958528
cg19435284	0.03589716	11.0474691	1.17133879	104.194084
cg10891888	0.03591159	198818.543	2.2306685	1.7721E+10
cg17740434	0.03592054	0.12663182	0.01836807	0.87301612
cg02882624	0.03593164	1788544258	4.05064577	7.8972E+17
cg10729325	0.03594698	12126.1084	1.85277953	79363195.8
cg22211300	0.03595853	2290.97754	1.66021982	3161375.39
cg22132343	0.03597594	0.10378748	0.01249312	0.86222196
cg17300750	0.0359761	10.0586595	1.16306192	86.9916114
cg21225608	0.03598268	388114.536	2.32026353	6.4921E+10
cg19422030	0.03598586	5.69134134	1.12042186	28.9099734
cg23955930	0.03598688	663391.348	2.40237555	1.8319E+11
cg21349401	0.0360007	8853.83536	1.81049753	43297711.9

cg10584478	0.01918914	0.0358884	0.00221587	0.58125035
cg15909016	0.01919589	1788207.43	10.4534292	3.059E+11
cg25594549	0.01919657	23.4358595	1.67228087	328.437359
cg26087598	0.01919797	449085210	25.7254803	7.8396E+15
cg14415214	0.01920172	5.97E-07	3.69E-12	0.09674894
cg20698170	0.01920389	93687.6659	6.45950094	1358832336
cg11947981	0.01920652	4.39918727	1.27301633	15.2023569
cg00106446	0.01920662	1.1064E+10	43.3107153	2.8265E+18
cg03189237	0.01920779	2563.31811	3.59265533	1828897.88
cg02891726	0.01920894	0.106989	0.01647501	0.69478831
cg11399814	0.01920925	89757714.9	19.7586904	4.0774E+14
cg04407431	0.01921141	414861.476	8.22650765	2.0921E+10
cg15547624	0.01921173	651793.663	8.85455742	4.7979E+10
cg17667988	0.01922175	149924.426	6.96357707	3227843006
cg14328907	0.01922216	51841458.3	18.0395625	1.4898E+14
cg07562079	0.01922296	90190494.1	19.739712	4.1208E+14
cg02081427	0.01923431	7218.7088	4.24590309	12272950.1
cg12107580	0.01924022	0.00908592	0.00017739	0.46537731
cg07578070	0.01925051	442.093282	2.69317029	72571.1519
cg27113548	0.01925113	0.16005819	0.03451182	0.74231455
cg17916473	0.01925236	3899.56755	3.83701247	3963142.48
cg02132636	0.01925706	5720.55731	4.08258497	8015699.88
cg26706909	0.01925914	2475.52406	3.56237459	1720262.49
cg17217059	0.01926066	18.5566372	1.60774861	214.180738
cg27495887	0.01926708	747.538987	2.93112724	190648.338
cg25947878	0.01926895	4.23262444	1.26424437	14.1706066
cg02898915	0.01926932	33.2118817	1.76694565	624.257508
cg09106999	0.01927076	52.8884422	1.90565225	1467.83723
cg03807235	0.0192727	0.00054573	1.01E-06	0.29499053
cg18500436	0.01927315	12.2823142	1.50310186	100.362621

cg06148175	0.03600455	40.3153234	1.27299439	1276.77334
cg24276070	0.03601524	7308662.23	2.80410034	1.9049E+13
cg24269840	0.03603964	8.03863901	1.14536133	56.4186299
cg18634560	0.03603969	9.72115019	1.15962263	81.4926841
cg17789479	0.03604918	1.3727E+15	9.66012185	1.95E+29
cg22099236	0.03604922	1546122.12	2.52769663	9.4572E+11
cg19910382	0.03605133	0.1111594	0.0142548	0.86682467
cg25982743	0.03606945	13.5516755	1.18451874	155.040104
cg07665387	0.03609768	7.243001	1.13695696	46.141644
cg06484562	0.03611156	1024973.39	2.45032591	4.2875E+11
cg02317397	0.03611207	0.06972848	0.00577713	0.84160438
cg13804854	0.03612852	6656.16829	1.76704273	25072724.9
cg15822350	0.03613763	14925.7009	1.86095615	119710798
cg10968375	0.03614196	1.3523E+10	4.51326243	4.0522E+19
cg04582136	0.03614698	0.0005643	5.16E-07	0.61691106
cg10409799	0.03614754	733473.502	2.39195064	2.2491E+11
cg02819335	0.03615635	4.85791668	1.1073797	21.310987
cg07285708	0.03615928	10.3695489	1.162872	92.4672228
cg23275158	0.0361616	47.6133155	1.28297845	1767.00381
cg24692861	0.03616826	1904.63837	1.62723311	2229334.76
cg14826401	0.03616928	5.48E-14	2.15E-26	0.13967273
cg03853794	0.03618087	9.45009189	1.15564544	77.2765017
cg10684802	0.03618839	7.46480202	1.13813891	48.9599897
cg16751098	0.03622634	4313969.94	2.66572664	6.9813E+12
cg10268345	0.03623661	8.93033716	1.15073722	69.3041995
cg01447966	0.03625305	3.4341E+13	7.36025293	1.60E+26
cg11529190	0.03625642	13446.8143	1.83799698	98377100.8
cg01553935	0.03626409	33.2529784	1.25135447	883.650957
cg16579893	0.03626936	56.1594552	1.29389039	2437.52054
cg26504026	0.03630921	12.446977	1.17442182	131.917879

cg09686308	0.01928071	28.3902469	1.72201761	468.059159
cg00009001	0.01928438	4.92313026	1.29545648	18.7093986
cg12296552	0.01929473	0.1707046	0.03882593	0.75053085
cg03009158	0.01929829	0.05318417	0.00455386	0.62113308
cg12718671	0.01929994	226.580761	2.4112597	21291.2949
cg17107246	0.01930368	0.04049592	0.0027593	0.59432516
cg02797271	0.01932674	7.31984178	1.38084489	38.8023913
cg23575732	0.01932845	13.4518534	1.52396231	118.738081
cg06224510	0.01933992	8.62919923	1.41789923	52.5164818
cg10583651	0.01934105	8.54759503	1.41569437	51.6081596
cg00571292	0.01935043	7.70157256	1.39180554	42.6167437
cg06263193	0.01935476	5.1068627	1.30215288	20.0284062
cg17026874	0.01935493	8248675294	40.3289209	1.6871E+18
cg03680032	0.01936125	4.89219615	1.29303791	18.5095758
cg01656216	0.01936196	0.07068253	0.00767154	0.65124097
cg03675258	0.01936575	6.17236812	1.34254014	28.3776455
cg26103512	0.01936638	1619.50812	3.30670084	793179.262
cg20054157	0.01937815	9.86673161	1.44814634	67.2255214
cg00850756	0.0193819	2.2334E+13	144.182895	3.46E+24
cg11859316	0.01938463	585523863	26.1698345	1.3101E+16
cg24879595	0.01938598	1247908.27	9.67737727	1.6092E+11
cg20887061	0.01938816	171.583875	2.29764503	12813.566
cg14163776	0.01939139	83.7187006	2.04573801	3426.05984
cg09159285	0.01939426	4.74504177	1.28620399	17.505327
cg12019705	0.01939696	0.0838393	0.01049284	0.66988821
cg20020464	0.01940076	1830.71532	3.36693844	995420.217
cg17339521	0.01940184	53.2962043	1.901161	1494.07935
cg25789861	0.01940739	4.12605038	1.2573071	13.5402812
cg27612744	0.01940747	1072.46832	3.08720566	372566.14
cg17476026	0.01941274	9.04041159	1.42705131	57.2712706

cg20023120	0.03636463	7.59630812	1.1373782	50.7341334
cg18191200	0.03637285	3227.56106	1.66961258	6239262.04
cg11786946	0.03637353	0.00022265	8.45E-08	0.58653237
cg22471129	0.03637484	17.8304703	1.20051004	264.8255
cg21702506	0.03638443	24.2489153	1.22396656	480.413364
cg03591990	0.03638519	23374.0899	1.89189987	288782768
cg02634501	0.03638697	7789067620	4.23498391	1.4326E+19
cg00612202	0.03638704	392879.628	2.26215545	6.8233E+10
cg00362906	0.03639148	574.035832	1.49547634	220342.594
cg06093712	0.03639179	6.00685835	1.12027929	32.2083497
cg21292033	0.03640927	5.61139405	1.11528817	28.2328317
cg01166827	0.03641028	1784.22909	1.60573572	1982563.76
cg02273078	0.03642235	4668776.31	2.63915494	8.2593E+12
cg26359174	0.0364397	4.91E-07	6.03E-13	0.39980038
cg06069457	0.03645871	0.13666457	0.02117275	0.88213398
cg16937168	0.03648439	5.80610483	1.11695839	30.1809393
cg11378840	0.03649254	500.931482	1.47797061	169781.691
cg16479401	0.03650021	1.80E-05	6.44E-10	0.50351136
cg04171065	0.03652882	6.34448789	1.12274334	35.8519399
cg03462943	0.03653285	29.1715845	1.23529056	688.891642
cg15616946	0.03653587	7.86E-09	1.99E-16	0.31077201
cg06223477	0.03653768	536211178	3.52061465	8.1668E+16
cg05426966	0.03654424	6.72678093	1.12670172	40.161101
cg09741298	0.03654795	355.98135	1.44422793	87744.2675
cg10143146	0.03655824	7.66024801	1.1357406	51.666199
cg01814945	0.03656956	31.0679805	1.23939196	778.78463
cg16354207	0.03658292	89.49798	1.32366608	6051.29086
cg27640078	0.03660436	90.2164262	1.32368798	6148.73269
cg23679724	0.03661021	10.863508	1.16010009	101.728987
cg20116935	0.03662103	29.4906398	1.23429048	704.613581

cg18284650	0.0194139	1147107.58	9.5208084	1.3821E+11
cg18212197	0.01941881	84.1031769	2.04553591	3457.94191
cg04771433	0.0194223	0.13076664	0.02374846	0.72004312
cg10273135	0.01942584	6.603965	1.35624255	32.1567508
cg23890469	0.01943056	28.3963678	1.71611465	469.871699
cg26875384	0.01943288	16.2818437	1.56870357	168.99205
cg25369015	0.0194382	64.5585429	1.95892685	2127.59628
cg01818776	0.01944048	0.22003249	0.06180826	0.78329812
cg00739123	0.01944132	100538.466	6.41164379	1576504160
cg08938584	0.01944512	10.864912	1.46927503	80.343238
cg04680150	0.01944679	80.278675	2.02850229	3177.05613
cg06168026	0.01944913	5.23655218	1.30604057	20.9958858
cg02196805	0.01945298	8.54334019	1.41323358	51.6465663
cg21788755	0.01945472	14.6129162	1.54094107	138.575917
cg02641288	0.01946256	11.6980274	1.4864487	92.0609266
cg22505962	0.01946278	31.1754879	1.74083923	558.30029
cg25665152	0.01946614	10053.3673	4.41531752	22890809.9
cg03337035	0.01947106	20.8837274	1.63169453	267.286591
cg11846581	0.0194711	1.9085E+11	65.6850288	5.54E+20
cg20303303	0.01947178	2.7413E+11	69.6229164	1.08E+21
cg12644264	0.019474	10.8044419	1.46725738	79.560659
cg03096732	0.01947482	78.6480397	2.02009455	3061.9924
cg03722802	0.01948126	12.8515204	1.50865353	109.476147
cg27426082	0.01948631	1415.04002	3.21588063	622640.731
cg19163814	0.01949321	1.7359E+11	64.4347641	4.68E+20
cg01488300	0.01950518	15.2190349	1.54960283	149.469929
cg04003582	0.01951092	6.41125653	1.34830361	30.4858712
cg04909508	0.01951886	2.44E+11	67.7504104	8.79E+20
cg26824174	0.0195397	12.2302474	1.49515521	100.042425
cg15440392	0.01953976	3435325.6	11.2186071	1.052E+12

cg23540518	0.03664432	7.42729185	1.13257293	48.7073835
cg21576886	0.03665089	0.15967372	0.02856989	0.89239744
cg07704437	0.03665344	1907786.77	2.45270512	1.4839E+12
cg01027532	0.03665884	12.2490693	1.16809134	128.448602
cg19554255	0.03666357	7.27445574	1.13089607	46.792723
cg13119284	0.03667944	316896211	3.35966094	2.9891E+16
cg16751451	0.03667994	8.03441728	1.13768973	56.7394247
cg27231335	0.03668267	15.6455286	1.18556656	206.46885
cg08753297	0.0367255	11.596393	1.163183	115.610639
cg01374870	0.0367312	0.03347851	0.00138192	0.81105189
cg21041775	0.03675162	3.97436936	1.08864219	14.5094614
cg08911275	0.03676402	3.53536414	1.08074289	11.5650075
cg25589890	0.03676505	21.1738231	1.20646455	371.607093
cg18146964	0.03676852	230002.747	2.13581923	2.4769E+10
cg07614064	0.03680312	1429477344	3.64043812	5.6131E+17
cg27062369	0.0368072	24.2497214	1.21575341	483.691004
cg19794507	0.0368108	6.57346123	1.12226193	38.502948
cg03554335	0.03682467	9.6504844	1.14879045	81.0694843
cg04578193	0.03683307	24.8830828	1.21717117	508.694114
cg24404083	0.03686183	503212171	3.39482038	7.4591E+16
cg20801130	0.03686291	54.558349	1.2762658	2332.28333
cg00494665	0.03686474	5.06584131	1.10401333	23.2449623
cg20180059	0.0368819	157887884	3.15710542	7.896E+15
cg23336454	0.03688746	3134.07146	1.63237714	6017239.32
cg08545493	0.03690395	0.12658497	0.01816903	0.88192677
cg03547631	0.03690801	4.2457E+12	5.85350845	3.08E+24
cg01283289	0.03690888	12.2839922	1.16465094	129.563683
cg20254361	0.03694849	10.4728735	1.15288295	95.1363537
cg23842796	0.0369505	4.95935858	1.10183065	22.3221576
cg19635869	0.03695298	56.3307982	1.27644825	2485.92829

cg25985355	0.01954089	5.80010265	1.32627166	25.3652339
cg11008243	0.01954532	8.27676841	1.40413944	48.7878151
cg11399754	0.0195458	6414629575	37.5853441	1.0948E+18
cg01522826	0.01954592	20.7150605	1.6270192	263.742268
cg27099511	0.0195598	179225152	21.1197991	1.5209E+15
cg05155520	0.01956172	0.20591581	0.05464176	0.77598741
cg22230604	0.01956277	5.65313235	1.32047074	24.2019036
cg01643580	0.01956976	9.38514817	1.43222891	61.4992516
cg02382037	0.01957532	8.85031074	1.41869151	55.2114394
cg11203156	0.01957736	10721718.9	13.4129711	8.5705E+12
cg02614657	0.01957867	0.14355213	0.02813273	0.73249973
cg19653161	0.0195868	56.8952557	1.91147218	1693.49581
cg01290345	0.01958882	80.6828238	2.02144689	3220.32604
cg02665570	0.0195938	117855.969	6.49797905	2137592211
cg17346857	0.01959744	32.4902439	1.746822	604.306534
cg08876479	0.019603	12978144.2	13.7903327	1.2214E+13
cg04650403	0.01961407	679.401325	2.84123039	162459.955
cg18189263	0.0196147	0.11485988	0.01865637	0.70714663
cg05730283	0.01962217	10637077	13.3293719	8.4886E+12
cg01348086	0.01962994	12.1345861	1.49096035	98.7606285
cg05147525	0.01963361	187.233403	2.30977821	15177.3651
cg00202389	0.01963675	0.03807167	0.00244495	0.59283486
cg17951138	0.0196388	6.2707569	1.34134012	29.3157502
cg18609783	0.01964421	8.18646858	1.39966671	47.88159
cg02969706	0.01964457	0.20178069	0.05259238	0.77417009
cg19967492	0.01964877	7.72338915	1.38660458	43.0192867
cg20970697	0.01964897	3.7338E+11	70.8383348	1.97E+21
cg17807863	0.01965153	9.87071859	1.44201156	67.5660917
cg00341387	0.01965526	54.2938854	1.89362546	1556.71016
cg13860849	0.01965574	8.93805593	1.41922048	56.2906502

cg06321304	0.03696045	38470.4637	1.89429519	781280864
cg04700925	0.03696594	22670.0165	1.83413332	280202996
cg09848727	0.03697781	1593680274	3.59787359	7.0592E+17
cg17371020	0.03699695	20.6688772	1.20047064	355.862503
cg26269802	0.0369985	9.61073128	1.14625431	80.580858
cg04981611	0.03703132	6.00175075	1.11383185	32.3397218
cg10227731	0.037032	67770038.4	2.95851182	1.5524E+15
cg11926393	0.03703305	27947.605	1.8511643	421933710
cg17379405	0.03703645	10.8437209	1.15411172	101.884662
cg04693399	0.03703746	615.397712	1.47131243	257398.997
cg03363567	0.03703766	212537159	3.16737488	1.4262E+16
cg17635080	0.03704509	5.85225752	1.11200858	30.799149
cg12214534	0.03705259	2116147.34	2.39808094	1.8674E+12
cg20964023	0.03708461	84905.6141	1.97337698	3653110064
cg13085976	0.03708999	13.3767933	1.16796772	153.205091
cg13995497	0.03709124	8.25491748	1.13468353	60.055214
cg10984505	0.03709174	169779.969	2.05612715	1.4019E+10
cg02989600	0.03710074	45.8087412	1.25703283	1669.36035
cg09639735	0.03710257	286.005218	1.40249467	58323.9187
cg01761478	0.03710733	4540.13387	1.65432684	12459941.4
cg23652987	0.03711897	22.1491217	1.20322902	407.722539
cg10714284	0.03712492	118019.104	2.00798697	6936553452
cg05598886	0.03714224	7.45431391	1.12720208	49.296215
cg06839900	0.0371451	7.60357299	1.12850245	51.2310116
cg07535060	0.03714567	42.302016	1.25001468	1431.55163
cg27045999	0.037146	8.93384901	1.13938637	70.0496866
cg02397514	0.03715914	2.43E-08	1.67E-15	0.35216026
cg19196335	0.03716096	17.267716	1.18476769	251.672979
cg00708105	0.0371881	7.13967685	1.12380553	45.3592584
cg10270204	0.03720789	0.12620418	0.01800683	0.88452529

cg23542968	0.01965688	17.8764392	1.58547339	201.559408
cg02119927	0.0196593	12644.6074	4.52441173	35338537.9
cg23272399	0.01966654	6.65435908	1.35365209	32.7118727
cg10743378	0.0196686	7.85371088	1.38992949	44.376909
cg11636504	0.01966939	15.6149644	1.5512	157.186122
cg06713675	0.01967	11.7152907	1.4815917	92.6355324
cg25652029	0.019672	4.20455094	1.25784715	14.0543695
cg19178040	0.01969096	5306932.84	11.8385165	2.379E+12
cg13075709	0.01969151	4325640.08	11.4577922	1.6331E+12
cg07713066	0.01969604	1.7193E+10	42.9759136	6.8783E+18
cg25396971	0.01969622	9.15458544	1.42378571	58.8616912
cg07433773	0.01969746	0.00440178	4.61E-05	0.42074958
cg18100564	0.01969748	14.8123182	1.53738027	142.713404
cg10380348	0.01969774	6.32044177	1.34203409	29.7667432
cg17960926	0.0197022	1.37E-06	1.61E-11	0.11599867
cg03969906	0.01970371	1009.39686	3.01433384	338012.333
cg04582136	0.01970385	0.00047411	7.62E-07	0.2949568
cg07714565	0.01970391	12.9453559	1.50451576	111.386164
cg09861871	0.01970478	18509.7619	4.79381248	71469479.7
cg08363925	0.0197059	5.25E-05	1.33E-08	0.20769315
cg06677890	0.01970787	6.32457685	1.34200249	29.8064071
cg15976636	0.01970867	12.4485119	1.49502993	103.653743
cg25212090	0.01971012	6.42363877	1.34529435	30.6721982
cg04101194	0.01971123	9.20197455	1.42463417	59.4372488
cg02357321	0.01972732	17.2424294	1.57418368	188.860662
cg24079361	0.01973478	4.345475	1.26368973	14.9428712
cg07669793	0.01974231	79.6445195	2.00798028	3159.01981
cg25794884	0.01974242	306.848236	2.48909361	37827.3599
cg01994599	0.01974636	19635.9275	4.82558994	79901038.8
cg22149588	0.01975258	1040.61872	3.02198497	358336.437

cg24380059	0.03721235	8.69607962	1.13674684	66.5247513
cg01787574	0.0372166	5.44552611	1.10560959	26.8211807
cg16261871	0.03721973	9.63909943	1.14360865	81.244784
cg14995433	0.037244	115214.593	1.9913514	6666027063
cg13010199	0.03724953	65804.3915	1.9259236	2248385112
cg06359167	0.0372524	439653.807	2.15420604	8.9729E+10
cg12977686	0.03725787	195369495	3.08631924	1.2367E+16
cg05807768	0.0372612	173102916	3.06339079	9.7815E+15
cg10415607	0.03729367	16604.1155	1.77168644	155612555
cg22967612	0.037295	3593430.8	2.43103219	5.3116E+12
cg26066361	0.03729572	5.87419198	1.10981286	31.0918469
cg06683487	0.03729585	0.08948522	0.0092297	0.86759119
cg08738562	0.03729954	1037101.85	2.25888886	4.7615E+11
cg15755476	0.03730786	6.92268965	1.12046049	42.7713716
cg17186803	0.03731351	13.1559634	1.16347652	148.760522
cg03009158	0.03733329	0.04631917	0.00256914	0.83508982
cg14732337	0.03734588	20.1451681	1.19239934	340.34554
cg27468419	0.03735347	6.23060869	1.11308414	34.8765052
cg01579086	0.03736945	1537.73301	1.53593477	1539533.36
cg02296128	0.03739713	4423308749	3.65404995	5.3545E+18
cg15673540	0.03740874	411.94237	1.42039992	119470.942
cg18669588	0.03741356	20.2119973	1.19143059	342.885971
cg11809091	0.03741689	9.76277386	1.14192944	83.4655365
cg22804475	0.03741972	1.7496E+14	6.75154087	4.53E+27
cg12434312	0.03742129	12.1263159	1.15637822	127.162148
cg03592345	0.03742208	17.1870231	1.18008824	250.314978
cg07500501	0.03742927	16764355.2	2.63240948	1.0676E+14
cg19621160	0.03743914	91.4435731	1.30021347	6431.19554
cg09912793	0.03745053	9.62905492	1.14058264	81.2906452
cg24938727	0.03745228	20.1694465	1.19059938	341.682165

cg21803052	0.01975528	4.0601E+11	70.4096174	2.34E+21
cg15233961	0.01976558	8.97772946	1.4178824	56.8450716
cg10130900	0.01976643	7.73421216	1.38463045	43.2014461
cg08301518	0.01976981	0.03037499	0.00160845	0.57362196
cg10200728	0.01977046	5679223.76	11.8664653	2.718E+12
cg25731359	0.01977856	7.80112659	1.386293	43.8995045
cg05867645	0.01977949	71047287.9	17.7159385	2.8493E+14
cg23681866	0.01978329	3.6852607	1.23041441	11.0378636
cg27540189	0.01978552	43.648407	1.82255529	1045.33643
cg15482168	0.01978709	514.237278	2.69726226	98040.1433
cg26264580	0.01979004	74.7820497	1.98513946	2817.10936
cg05166976	0.01979156	0.10216292	0.01499765	0.69592658
cg17740434	0.01980035	0.15261122	0.03139518	0.74183946
cg00969405	0.01980242	7.16101207	1.36711001	37.5098517
cg24999883	0.01980307	0.0007182	1.63E-06	0.31671033
cg21193440	0.0198041	4.4849E+11	70.8959379	2.84E+21
cg13985198	0.01980601	22.0657086	1.63456489	297.87468
cg01722450	0.0198073	0.18716101	0.04570958	0.76634358
cg19788272	0.01981968	7.52667289	1.37763962	41.1216431
cg16429070	0.01982001	6.43738054	1.34387098	30.8361953
cg23395902	0.01982162	6607656.37	12.089183	3.6116E+12
cg23043245	0.01982575	10.9271588	1.46147115	81.7004155
cg13915702	0.01983114	2019973.12	10.0068997	4.0775E+11
cg02275294	0.01983936	287.325012	2.45395206	33641.9214
cg00286450	0.01984196	1.3791E+17	522.169642	3.64E+31
cg09449611	0.01984653	47581.1215	5.51507785	410504291
cg27064845	0.01985231	38.5632124	1.78404703	833.566226
cg11270005	0.01985571	129.988059	2.16272762	7812.77097
cg26839807	0.01985995	340659416	22.4862219	5.1609E+15
cg19838043	0.01986728	8.43100484	1.40170334	50.711046

cg18792146	0.03745257	4.24338479	1.08755529	16.5566888
cg02879798	0.03745375	47.8119987	1.25176026	1826.21808
cg02328440	0.03746018	11.3792538	1.15156628	112.444606
cg27431396	0.03746145	70236.0173	1.91085505	2581618174
cg06830784	0.03747755	4.63156154	1.09289062	19.6280962
cg15242360	0.03751209	0.15594533	0.0270752	0.89820019
cg10045881	0.03751394	7.59377904	1.12424592	51.2925858
cg01721544	0.03753703	8.71341434	1.13293374	67.0150307
cg21365903	0.0375372	2126772.42	2.31635808	1.9527E+12
cg04330122	0.03755485	7.66191065	1.12436347	52.2116527
cg09874482	0.0375554	10.7372701	1.14641122	100.565109
cg11699265	0.03756139	5.91026274	1.10762438	31.5370502
cg00014272	0.03758314	30808.0947	1.81034919	524284873
cg12665414	0.03758493	8.17902902	1.12824652	59.2924637
cg21276549	0.03758546	0.02255595	0.00063251	0.80436752
cg09740133	0.03759996	0.0069023	6.34E-05	0.75176676
cg12434258	0.03760607	30350.869	1.80668453	509870557
cg07757959	0.03761147	112.644816	1.31078995	9680.31116
cg05516012	0.0376295	2.2149E+10	3.9058677	1.26E+20
cg08386886	0.03764339	7781.50624	1.66835448	36294348.8
cg03880683	0.03765552	338925.111	2.06819995	5.5541E+10
cg27477205	0.037659	14558850.1	2.56249639	8.2716E+13
cg09667226	0.03766234	5082263514	3.57750102	7.22E+18
cg15348799	0.03766961	4082.58721	1.60626961	10376538.4
cg10459387	0.03770234	67.2635887	1.27024096	3561.83632
cg08307816	0.03774549	6.09899133	1.10780928	33.5777069
cg26918305	0.03775715	202.901153	1.35056882	30482.6215
cg10191684	0.03777929	134.875667	1.31902259	13791.6104
cg20887061	0.03778175	127.701749	1.31488147	12402.4387
cg09251959	0.0377834	14.5358058	1.16306968	181.665514

cg11934304	0.0198697	7.52951894	1.37677332	41.1786419
cg07145284	0.01987321	5.36022039	1.30457936	22.0239285
cg13762471	0.01988236	30577.8305	5.12875103	182306319
cg02397514	0.0198846	8.61E-08	9.74E-14	0.07616942
cg18811550	0.01989175	14.823742	1.53207606	143.428471
cg21868050	0.01989221	1.508E+15	252.13196	9.02E+27
cg27308245	0.01989295	10.8896746	1.45907668	81.2740101
cg04337096	0.01989621	7.70225558	1.38121837	42.9510224
cg06399971	0.01989722	13.8661273	1.51582378	126.841582
cg07811634	0.01989825	207497425	20.6838115	2.0816E+15
cg06642093	0.01990234	589864689	24.3871604	1.4267E+16
cg02912007	0.01990362	6.09828519	1.3310051	27.940601
cg02299189	0.01990599	1180.05202	3.06032869	455023.922
cg24082871	0.01990986	9.00910085	1.41560212	57.3352475
cg23048036	0.01991128	14.2060068	1.52125501	132.660618
cg05193369	0.01991178	14.7904115	1.53096753	142.887598
cg12455762	0.01991565	8.00927615	1.38940295	46.1698348
cg17597195	0.01991893	433973050	23.1795039	8.125E+15
cg24463605	0.01992387	10.4185962	1.44818818	74.9537581
cg18509271	0.0199261	3.026E+10	45.2826566	2.0221E+19
cg25641997	0.01993399	109325.554	6.24922583	1912569196
cg18039296	0.01993602	0.04256408	0.00298263	0.60741742
cg08274661	0.01993709	502863.726	7.95020889	3.1807E+10
cg23858195	0.01993993	3951.68522	3.69775249	4223056.06
cg16117513	0.01994135	12.2598413	1.48546705	101.182796
cg18342279	0.01994173	3.94951374	1.24218965	12.5573891
cg09555914	0.01994269	5.28258942	1.3005442	21.4569801
cg05104080	0.01994732	49.0675696	1.84881812	1302.25162
cg11625005	0.01995587	0.13419263	0.02472269	0.72838586
cg22416721	0.01995922	235.887439	2.36778636	23499.9598

cg07718808	0.03779405	5705042.25	2.40411654	1.3538E+13
cg14522034	0.03779407	14.5152511	1.16281342	181.192023
cg06830503	0.03782466	14487.3134	1.71392861	122456821
cg06491405	0.03785456	8.10277985	1.12450987	58.3854738
cg10779674	0.03786506	26921.2429	1.77110888	409208787
cg07030951	0.03786614	22972.4278	1.75534305	300643479
cg26977936	0.03791745	6.73633235	1.11226488	40.797992
cg19424078	0.03792085	6.5708E+14	6.70280478	6.44E+28
cg11365324	0.03793602	195688.22	1.97093572	1.9429E+10
cg07407499	0.03795582	13.922698	1.15765592	167.443119
cg17803089	0.03796375	26.1466854	1.19877829	570.288238
cg08856118	0.0379696	65.1629962	1.26100668	3367.32242
cg15444648	0.03797195	7.95872681	1.12203515	56.4521822
cg10723075	0.03799228	42407384.1	2.64632366	6.7958E+14
cg23473726	0.0380089	45831.4445	1.8109164	1159921740
cg01510388	0.03801762	9.43541173	1.13211618	78.6376841
cg19263607	0.03802024	13.230867	1.15343764	151.768797
cg26484631	0.03802188	18.574424	1.1752418	293.564462
cg15841063	0.0380296	3.71317825	1.0751416	12.8240714
cg05633748	0.03804108	40.4874328	1.22652906	1336.48054
cg08114317	0.0380445	9.78947176	1.1340828	84.503316
cg19224203	0.03806583	85633.4429	1.86870576	3924152587
cg20244273	0.03808768	18.6996266	1.17456687	297.706365
cg10013356	0.03809262	329.077732	1.37482006	78768.2381
cg20417723	0.03811189	0.18027616	0.03570005	0.91034859
cg27422678	0.03812847	1285384.49	2.15985078	7.6497E+11
cg12894325	0.03813427	62.2010328	1.25356241	3086.37882
cg20437660	0.0381352	0.18680864	0.03825211	0.91230172
cg01189638	0.03814342	27.2394578	1.19801193	619.349476
cg22243298	0.03814878	0.18000407	0.03558428	0.91055558

cg20587394	0.01997921	0.07639627	0.00875408	0.66670494
cg11960229	0.01998191	16.3724632	1.55368833	172.529809
cg23378144	0.01998531	283.490167	2.43502147	33004.5036
cg03400491	0.01999726	6100.99498	3.94680175	9430962.61
cg15030629	0.02000136	39800516.2	15.7347621	1.0067E+14
cg08091561	0.0200069	0.06218121	0.00598759	0.64575248
cg20941855	0.02002121	8.54512485	1.4015385	52.0992884
cg24153071	0.02002742	411080.723	7.64029061	2.2118E+10
cg17980283	0.02003146	47.462274	1.83508103	1227.55748
cg25000158	0.02003393	2422723053	29.9089546	1.9625E+17
cg02005367	0.02003484	5.1126E+13	143.144441	1.83E+25
cg03473046	0.02004028	635.006601	2.75836565	146185.616
cg24406391	0.02004373	45.0807577	1.81970594	1116.8149
cg11930554	0.02006254	3.43366341	1.21381008	9.71325295
cg09677935	0.02006428	123127428	18.648561	8.1295E+14
cg11320084	0.02007701	25.7058355	1.6646901	396.944739
cg04566159	0.02008344	10.7627451	1.45189932	79.7828608
cg21483820	0.02008649	8798.66632	4.15818363	18617871.6
cg02026498	0.02009949	11.8286381	1.47317947	94.9759905
cg23837191	0.02010265	0.11411034	0.01830031	0.71152738
cg02825728	0.02010392	0.15768752	0.03321777	0.74855572
cg18806716	0.02010472	17.7703127	1.57011173	201.122001
cg03029145	0.02011056	11444605.4	12.7757809	1.0252E+13
cg22929802	0.02011119	20454.3687	4.73879464	88288527.3
cg17833578	0.02011638	0.11807412	0.01948526	0.71548937
cg09418959	0.0201174	5237389695	33.3391428	8.2276E+17
cg08314603	0.02013487	18.1789332	1.57478531	209.853123
cg11609545	0.02014285	12376006.3	12.8873257	1.1885E+13
cg25372693	0.0201438	103.840837	2.0682262	5213.60738
cg14581491	0.02014615	1.5797E+10	39.4547211	6.3248E+18

cg13053082	0.03815845	19.7113978	1.17675423	330.178719
cg00807892	0.03816556	202784.301	1.94784966	2.1111E+10
cg25980157	0.03817989	4.6975E+18	10.4097082	2.12E+36
cg17557230	0.03819176	1634.54903	1.49593441	1786007.8
cg17067942	0.03820749	65644.0352	1.8274261	2358037543
cg23717805	0.03821133	5741146.14	2.32948399	1.4149E+13
cg24404909	0.03822159	57.369049	1.24586751	2641.69966
cg07796520	0.03823301	725952490	3.02363376	1.743E+17
cg02015348	0.03823584	52.105312	1.23903368	2191.19431
cg19877419	0.0382437	18.3162369	1.17062493	286.585844
cg04295543	0.03826254	23.2174185	1.18541642	454.733469
cg12959265	0.038263	0.19556638	0.04177511	0.91552621
cg06435751	0.03827332	12920.7331	1.66780294	100098963
cg23939875	0.0382786	9.92086176	1.13193284	86.9517117
cg01158680	0.03828985	5.90874328	1.1005852	31.7224391
cg25733934	0.03829205	120.866764	1.29515625	11279.5461
cg08986653	0.03829644	0.00492517	3.23E-05	0.75088941
cg18426551	0.03830943	0.13261132	0.0196072	0.89690318
cg17808849	0.03830948	67.6517474	1.25479094	3647.42747
cg16430951	0.03831854	270236873	2.84266439	2.569E+16
cg12738981	0.0383222	7.50624246	1.11453114	50.5537028
cg19319928	0.03836293	11.0097246	1.13719112	106.590734
cg20791007	0.03837577	12.5424398	1.14498091	137.393379
cg25382652	0.03839377	0.04047657	0.00194466	0.84248815
cg10042437	0.03840056	175.816104	1.31798286	23453.4934
cg13975172	0.03841591	561.302283	1.4016182	224783.22
cg26706909	0.03841931	2330.34192	1.51198342	3591635.59
cg12631255	0.03842192	375428.787	1.98224152	7.1105E+10
cg09205065	0.03842714	18416875.3	2.43834533	1.391E+14
cg08739433	0.03843175	8.53538472	1.12097309	64.9906703

cg12843518	0.02014707	9.71227745	1.42728854	66.089183
cg20065569	0.02014745	4925.19144	3.78303073	6412189.7
cg13940125	0.02014825	9.79185997	1.42908631	67.0921841
cg19966212	0.02015241	4.72022213	1.27481758	17.4774
cg01414687	0.02015964	7.53682723	1.37151915	41.4166764
cg11068617	0.0201613	1.847E+13	118.815464	2.87E+24
cg26916966	0.02016718	4.68674051	1.27320208	17.2521997
cg17192377	0.02017226	67.5656994	1.93211529	2362.75949
cg06997549	0.02017355	7.43877452	1.36845445	40.4363961
cg07737104	0.02017515	7475.62928	4.03157121	13861849.4
cg10219093	0.02018528	1828.6662	3.23346285	1034191.58
cg22568860	0.02018853	1874.88136	3.24555772	1083074.29
cg07705594	0.02018889	31862.3753	5.05209166	200948642
cg27303409	0.02018893	771.757618	2.82528115	210814.354
cg23540518	0.0201911	6.89621109	1.35203967	35.1748018
cg13362105	0.0201985	104018531	17.8590035	6.0585E+14
cg18233786	0.02021282	38.9663004	1.77104391	857.33197
cg08378567	0.02021283	62.4975376	1.90654411	2048.70277
cg04994773	0.02021516	6.94150932	1.35299682	35.6132038
cg00374927	0.02021946	10031122.2	12.366783	8.1366E+12
cg09251771	0.02022766	7588.09287	4.02820416	14294000.8
cg01280841	0.02023164	4.39799265	1.25979254	15.3535909
cg24380059	0.02023533	8.31456181	1.3912412	49.690836
cg18576957	0.02023731	6.00452583	1.32237308	27.2648702
cg12804532	0.02024632	99.7565441	2.04873358	4857.32659
cg07106615	0.02025982	10.2487841	1.4367997	73.1052316
cg01826863	0.02026143	4.19416843	1.25014526	14.0712039
cg00087884	0.02027093	17.896401	1.56676759	204.42162
cg26632171	0.02027314	10.1342071	1.43398783	71.6199612
cg17056132	0.02027917	22.9830384	1.62869587	324.320866
cg26827725	0.03843413	11.4553554	1.13864528	115.246749
cg26397391	0.03844491	82.6070512	1.26465912	5395.86107
cg07485417	0.03846406	16.6148305	1.16093697	237.784307
cg12468273	0.03846746	6.59311713	1.10530045	39.3279434
cg07882671	0.03847996	2.83564099	1.05681938	7.60854692
cg08325021	0.03850434	5.3122E+10	3.69322374	7.64E+20
cg09508736	0.03851527	0.06935905	0.00553929	0.86846433
cg09245319	0.03853271	20.1902574	1.17183372	347.870597
cg03041742	0.03855045	4044580677	3.20683424	5.1012E+18
cg01926269	0.03855499	9.20581337	1.12399415	75.3980788
cg20928429	0.03855757	6.406925	1.10271968	37.2249527
cg16512239	0.03857518	7497029.43	2.29789376	2.446E+13
cg03086857	0.03858052	10.1877516	1.12968213	91.8756516
cg12234455	0.03858682	17.7439976	1.16299186	270.723692
cg06299833	0.03858816	4.19692886	1.07820306	16.3366369
cg21739584	0.0385941	38.3806445	1.21090902	1216.50251
cg13547250	0.03859651	141021220	2.67584992	7.432E+15
cg26608667	0.03860808	0.13335904	0.01976476	0.89981528
cg19942640	0.03861329	7566410.77	2.29224938	2.4976E+13
cg01973862	0.03862145	179439.959	1.88343129	1.7096E+10
cg24341129	0.03863358	10.4784036	1.13066537	97.1082562
cg14597908	0.03863415	54.5145741	1.23244693	2411.33205
cg04387010	0.0386492	76.9952973	1.2544921	4725.63822
cg25250998	0.03865001	105.335386	1.27515932	8701.29977
cg26945941	0.0386512	0.1552841	0.02657461	0.90737556
cg17261676	0.03870227	8.73294123	1.11913498	68.1457229
cg19792268	0.03873306	17.0026866	1.15804857	249.636638
cg12687767	0.03873343	13.0647552	1.1423503	149.418116
cg23489936	0.03875176	11099569.5	2.31326471	5.3258E+13
cg18632220	0.03877482	0.1176061	0.01544585	0.89546331

cg10715905	0.02028589	17.2524102	1.55740765	191.116089	cg06467910	0.0387765	14.2081112	1.14668609	176.046806
cg02011054	0.02028717	101673.005	6.00993017	1720053248	cg20729316	0.03877711	18123953.9	2.36776419	1.3873E+14
cg22243298	0.02029867	0.18318609	0.04369027	0.76806903	cg12217560	0.03877875	8.5635855	1.11710381	65.647432
cg09960727	0.02030951	3976708.31	10.6062149	1.491E+12	cg10124812	0.03879398	43.5567131	1.21450243	1562.11071
cg12616721	0.02032214	79735.1021	5.77158561	1101549372	cg21856334	0.0388113	0.12022992	0.01611832	0.89682024
cg02862025	0.02033148	1509.7258	3.11550003	731591.064	cg27636594	0.03881187	32.0061603	1.19502413	857.21641
cg10535858	0.02033838	1424048.05	9.01687789	2.249E+11	cg08730245	0.03881499	0.02856986	0.00097987	0.83300816
cg19643053	0.02033864	4.64484573	1.26915879	16.9991273	cg01793704	0.038823	150.072251	1.29346641	17411.879
cg17873465	0.02034157	246343196	20.0551948	3.0259E+15	cg07689503	0.03882491	811.426127	1.41047617	466801.475
cg27380724	0.02034513	0.1226347	0.02082749	0.7220876	cg00473633	0.03883803	6.78081918	1.10312923	41.6809813
cg25466368	0.02034959	33379621.9	14.6930645	7.5832E+13	cg02608453	0.03891784	0.03926323	0.00181771	0.8481018
cg23650853	0.02035438	34258.2761	5.05043374	232381918	cg05889842	0.03891787	76761.6016	1.77257311	3324175156
cg08702399	0.0203643	1.5199E+10	37.8868533	6.0971E+18	cg18679410	0.03891802	8.43470695	1.11462004	63.8282812
cg16094298	0.02037262	15.8064872	1.53387075	162.885326	cg22171758	0.03892233	77.2838896	1.24750874	4787.78174
cg09372028	0.02038887	2213.93065	3.29646945	1486890.45	cg04062119	0.03893896	12.0254155	1.13463312	127.451434
cg27256066	0.02039251	11.2577197	1.45481325	87.115136	cg01958916	0.03896492	108.385705	1.2679273	9265.09038
cg01029838	0.02039774	11947233.2	12.4623461	1.1453E+13	cg19552441	0.038968	8.01103735	1.11113937	57.7575786
cg17558126	0.0203993	7.56910057	1.36795455	41.8809847	cg20941170	0.03898269	23230148.1	2.35797017	2.2886E+14
cg11668770	0.02040112	7.18E-06	3.22E-10	0.15987228	cg17906146	0.03898522	16754.3351	1.63524776	171660681
cg10502118	0.02040592	5.91398609	1.31658478	26.5651191	cg09689449	0.03899015	90.417888	1.25564477	6510.91351
cg10959672	0.02040771	8.61445105	1.39545457	53.1789197	cg27061971	0.03899997	0.02865489	0.00098242	0.83579962
cg13946792	0.0204088	0.24544868	0.07487098	0.80465166	cg05241277	0.03900922	0.12304794	0.01682872	0.89969979
cg05178518	0.02040986	239542121	19.7920802	2.8992E+15	cg17374636	0.03901346	56591.7337	1.73644434	1844357603
cg26626663	0.02041216	5.69843638	1.30894644	24.8078732	cg22973515	0.03902966	108.944889	1.26638986	9372.30249
cg01725531	0.02042269	23420673	13.7917282	3.9772E+13	cg11195360	0.0390385	0.23117655	0.05752861	0.9289743
cg21502048	0.02042409	9.52583803	1.41698836	64.0383454	cg25879745	0.03904464	19.2543373	1.16032316	319.505391
cg22725685	0.02042683	4.53494136	1.2633176	16.2791155	cg07059157	0.03906433	0.01835098	0.00041157	0.81822412
cg01782132	0.02044599	8362805.85	11.7320603	5.9611E+12	cg15819780	0.03909781	41.0217991	1.2041414	1397.50032
cg15299978	0.0204473	8.15998735	1.38302826	48.1446368	cg04425270	0.03912303	541.248695	1.3689293	213999.473
cg17802464	0.02045289	9.64306034	1.41904864	65.5288411	cg03851496	0.03913503	18.7365583	1.15724683	303.356733

cg00009167	0.0204572	14.4418138	1.51026346	138.099075
cg20070464	0.02046827	444.503385	2.56242672	77107.8672
cg24003225	0.02047363	67.8447377	1.9168824	2401.24717
cg01993865	0.02047557	9.327E+12	100.23266	8.68E+23
cg15127250	0.02048135	12.352994	1.47366746	103.548775
cg14600813	0.02048503	10.1620422	1.42986838	72.2214044
cg02504465	0.02048529	1.2773E+11	51.6161824	3.16E+20
cg26433208	0.02048877	101686.249	5.91686729	1747562140
cg24091990	0.02049086	7.22320742	1.35643599	38.4645687
cg19708554	0.02049313	9.96766457	1.42543797	69.7009195
cg12387232	0.02050377	0.26541511	0.0864213	0.81513682
cg08486961	0.02050599	539688555	22.1533541	1.3148E+16
cg20012172	0.02050679	979005470	24.2796592	3.9476E+16
cg04352704	0.02050936	3354.99696	3.49265201	3222767.27
cg25133212	0.02051043	48262.6429	5.26631371	442298509
cg22501294	0.0205191	164.806233	2.19479645	12375.2224
cg05446860	0.02052236	4.50588914	1.26084622	16.1027068
cg08416638	0.02052406	0.03717325	0.00229396	0.6023871
cg13319446	0.02052406	5.58733256	1.30328085	23.9536129
cg04414912	0.02053026	0.2333643	0.06813004	0.79933745
cg10978355	0.02053762	5.19737311	1.28865174	20.9619764
cg00694520	0.02055877	6.68071745	1.33904863	33.3311163
cg16274678	0.02055967	8.15381025	1.38068258	48.1534443
cg14519515	0.02057335	30.5167102	1.6906995	550.819114
cg19783435	0.02058369	178.403083	2.21677977	14357.6104
cg25924411	0.02058659	198306.157	6.50653564	6043973947
cg03861105	0.02058722	641.824227	2.69796994	152684.554
cg19038228	0.02059073	307555.959	6.95761942	1.3595E+10
cg07654559	0.02059619	20627.3109	4.59367258	92624354.2
cg16674484	0.02059883	6.24089939	1.32445347	29.4074696

cg23395902	0.03913718	19519027.4	2.30808988	1.6507E+14
cg07204255	0.03914438	3.87191583	1.06972793	14.0145282
cg21367811	0.0391489	5.62718202	1.08978592	29.0563284
cg18665732	0.03915353	1.0154E+14	4.97486124	2.07E+27
cg06319102	0.03916753	61.9465659	1.22751013	3126.14692
cg22068044	0.03918792	58384.9377	1.72311045	1978283484
cg21401879	0.03919368	3.86411787	1.06927538	13.9640425
cg18357908	0.03921498	12647.4587	1.59529028	100269032
cg22052291	0.03921609	397.294865	1.34434071	117413.099
cg25429672	0.03924699	10.1800612	1.12118177	92.43251
cg17381426	0.03931182	7.40170956	1.10301186	49.6688262
cg03109066	0.03931272	2.9838E+12	4.08286111	2.18E+24
cg02490920	0.03931824	21.9471245	1.16321369	414.090962
cg06693487	0.03932149	4.66E-05	3.53E-09	0.61379288
cg05986853	0.03933333	9.2543263	1.11488834	76.817159
cg23514672	0.03933348	0.00121071	2.04E-06	0.72016474
cg10310846	0.03934601	1573859613	2.81157816	8.8101E+17
cg14112356	0.03935463	41.0731322	1.19866737	1407.39811
cg22213149	0.03935731	3829.41642	1.49525398	9807317.27
cg04747728	0.03935735	199.463595	1.2946188	30731.6142
cg13883696	0.03935779	5.18449	1.08354684	24.8064372
cg13714271	0.03937563	1751.26974	1.43833486	2132289.08
cg07476963	0.03937864	39.7381168	1.19622588	1320.08341
cg21911557	0.03938586	6.024E+14	5.23159942	6.94E+28
cg10187703	0.03939651	13.7763617	1.13587071	167.086043
cg16458866	0.03940475	7.84810088	1.10515699	55.7320706
cg04881642	0.03940986	24.32341	1.16743093	506.777966
cg04525496	0.0394118	8.31848711	1.10820348	62.4409046
cg01802294	0.03941409	8.01283723	1.10616785	58.0432351
cg24333469	0.03941522	6.47725071	1.09480482	38.3216953

cg14832904	0.02060109	7.20958656	1.35406606	38.3867078
cg13344867	0.02060562	63602.2057	5.45646214	741366927
cg27147545	0.02061207	71.1278968	1.9232935	2630.47617
cg19870284	0.02061321	0.19366379	0.0482432	0.77742897
cg22776646	0.02062563	11.1652449	1.44749018	86.1233428
cg25672318	0.0206297	9.6508E+12	97.7013261	9.53E+23
cg27539480	0.02063433	15.2755574	1.51849168	153.66739
cg10124812	0.02064214	42.8001227	1.77781046	1030.39696
cg20163085	0.02064374	4.67E-05	1.00E-08	0.21710113
cg07000955	0.02064583	39.0383033	1.75277641	869.471496
cg19245296	0.02065375	34718.7286	4.95584928	243225741
cg23709172	0.02065541	7.93060625	1.37298396	45.808631
cg07247133	0.02066233	96.876447	2.0135281	4660.99578
cg07484354	0.02066682	9.89761685	1.42010219	68.9829365
cg25804860	0.02066737	19.434368	1.57453024	239.877678
cg11587658	0.02067058	15829.3676	4.38962001	57082134.1
cg10802521	0.02067514	409080.843	7.21629882	2.319E+10
cg27167413	0.02067539	1337.03134	3.00698771	594499.541
cg21476824	0.02067621	1328.71949	3.00400283	587714.322
cg16511831	0.02067979	7.99079545	1.37410925	46.4685119
cg12104707	0.02067991	81.3873792	1.95954387	3380.33029
cg07759857	0.02068274	5.4244924	1.29503485	22.7214872
cg14095316	0.02068526	11.5896531	1.45437215	92.3560446
cg24050414	0.02069034	156.408251	2.16463628	11301.4557
cg20615910	0.02069218	10.688237	1.43632293	79.5353249
cg02359414	0.02069261	2808059130	27.7979524	2.8366E+17
cg07537750	0.02069326	31.0933411	1.69090799	571.761363
cg00609961	0.02069504	10.7178341	1.43686504	79.9462465
cg10588471	0.02069535	0.0300491	0.00154266	0.58531901
cg06633426	0.02070532	8.00600361	1.37402206	46.6485187

cg25920207	0.03942789	10991.4868	1.56915404	76992302.2
cg23413007	0.03942835	11680.7593	1.57374893	86697525.6
cg03005685	0.03943429	37.0242739	1.19095861	1151.00294
cg24176760	0.03943538	105.216196	1.25266474	8837.51859
cg07774177	0.0394354	129.197585	1.26517053	13193.491
cg01664011	0.03943852	1611647.76	1.99633545	1.3011E+12
cg18284650	0.03944426	949781.492	1.94518287	4.6375E+11
cg00534253	0.03945187	52694490.1	2.36041833	1.1764E+15
cg02325250	0.03945634	8.9152269	1.11140999	71.5139072
cg07879540	0.03948356	820.811928	1.38141014	487713.391
cg16985259	0.03948823	7.7281E+13	4.66021782	1.28E+27
cg21073212	0.03950078	20.243034	1.15555175	354.618843
cg21785847	0.03951792	30.1354848	1.17753355	771.228507
cg05376156	0.03951889	50926.2206	1.68205326	1541853639
cg06820762	0.03953319	8818.1356	1.54535266	50318297.8
cg11386080	0.03954842	11.797112	1.12530768	123.674489
cg13662121	0.03957944	11.6099562	1.12403474	119.917185
cg26394572	0.03958824	52287.5907	1.67807496	1629243150
cg06381959	0.03960016	48.6117675	1.20300946	1964.32699
cg04589156	0.03960739	2919334209	2.81907267	3.0232E+18
cg23284178	0.03961503	34656.7886	1.64329326	730906053
cg04123157	0.03962447	4923.43278	1.49718609	16190499.3
cg11050622	0.03964184	7712.50346	1.52828368	38921249
cg07028533	0.03968117	4.10062172	1.06887085	15.7316466
cg10335067	0.03972056	215421.412	1.78118339	2.6054E+10
cg17278655	0.03972809	1.0668E+10	2.95828642	3.8473E+19
cg27123665	0.03974511	172.759209	1.27325031	23440.5945
cg15077012	0.03974627	33309.1255	1.62943908	680907837
cg08767936	0.03975343	7.35556852	1.09799663	49.2755504
cg27299725	0.03976716	160794344	2.42059497	1.0681E+16

cg02140384	0.02070586	7.51979959	1.36092578	41.5506759
cg03216729	0.02070704	84241.9441	5.65329488	1255321950
cg07491796	0.02072219	39.8231907	1.75483194	903.725582
cg04425270	0.02072609	686.913529	2.70978368	174128.363
cg00501919	0.02072948	24.3451602	1.62759603	364.148605
cg16705300	0.02073483	8.90200374	1.39586804	56.7716063
cg12030002	0.02074052	38.4876073	1.74494034	848.909206
cg16537676	0.02074205	18.1117615	1.55539768	210.901629
cg13327336	0.02074482	0.02784033	0.00133817	0.57921335
cg03033975	0.0207538	521.702975	2.59537219	104868.965
cg11479221	0.02075803	9.83480545	1.41674936	68.2713549
cg27207079	0.02076647	9.48200414	1.40870593	63.8234005
cg06484562	0.02077075	261100.606	6.68403268	1.0199E+10
cg24291500	0.02077421	18.7150413	1.56220919	224.203501
cg22584335	0.0207777	137735.385	6.06031067	3130373547
cg19035395	0.02077893	8.78659556	1.39220362	55.4547197
cg05124756	0.02078232	15215.9252	4.33198903	53445282.9
cg04362989	0.02078768	6.42355146	1.32721702	31.0891232
cg13676983	0.02078818	166179944	17.8291218	1.5489E+15
cg04238038	0.02079253	10.7476989	1.43526162	80.482213
cg06664023	0.0207954	6766893166	31.3085284	1.4626E+18
cg00604539	0.02080444	8.8355E+10	46.214324	1.69E+20
cg15011837	0.0208056	31.4172947	1.68922674	584.318485
cg18929217	0.02080723	8.30692228	1.37980456	50.0106752
cg12955299	0.02080799	15498440.1	12.3987417	1.9373E+13
cg13634292	0.02080976	2.01E-08	5.95E-15	0.06753557
cg04881642	0.0208146	21.5288141	1.59457381	290.666908
cg00720475	0.0208207	122.624355	2.07691666	7239.93058
cg17635080	0.02082277	5.56800852	1.29813608	23.8824877
cg19378537	0.02082733	55.3202866	1.83994337	1663.27625

cg21916655	0.03976842	3101.8321	1.45656771	6605502.97
cg04016086	0.03977146	0.01596837	0.00030942	0.82409733
cg12080566	0.03977527	11.5704053	1.12125963	119.396324
cg07105947	0.03978288	6.07541902	1.08792653	33.9275819
cg04436474	0.03979058	6926.06123	1.51091702	31749145.4
cg16513905	0.03980764	5206.95815	1.4898861	18197641.5
cg15522728	0.03981485	26054.3554	1.60538637	422844899
cg08756266	0.03982848	20.7348764	1.151363	373.414031
cg19865525	0.03984239	96.5428204	1.23633184	7538.84669
cg11527279	0.03985673	10.8210328	1.1167135	104.856574
cg15149938	0.03986209	0.16274897	0.02881144	0.91933033
cg17929627	0.03987146	25287.3927	1.59873848	399973000
cg16167624	0.03987375	9.89294656	1.11187298	88.0229968
cg22486630	0.03987565	15.116664	1.13387295	201.533628
cg23633635	0.03988264	30172213.6	2.21703803	4.1062E+14
cg15726532	0.03988498	8422.49474	1.51853302	46715097.4
cg19119531	0.03990429	9142068.82	2.09448048	3.9904E+13
cg07676709	0.03990437	16.9618283	1.13948513	252.485629
cg26266703	0.03993667	1139.02376	1.38199492	938769.822
cg12901910	0.0399684	8.36215008	1.10219327	63.442189
cg15746415	0.0399724	36329.6353	1.61751903	815967152
cg00684824	0.03997413	15.5908145	1.13402003	214.34674
cg03861105	0.03997749	507.962948	1.33000967	194003.369
cg22864519	0.03999921	6.15353245	1.08652201	34.8506162
cg26817935	0.04000363	6.0087648	1.0853001	33.2675305
cg06084073	0.04000674	17.9628362	1.14088207	282.819313
cg19189310	0.0400125	68455.5199	1.66157565	2820309875
cg16559243	0.0400175	8.13336915	1.10024974	60.1242528
cg24718991	0.04003248	2923100.54	1.96904979	4.3394E+12
cg19675288	0.04004422	4.30031956	1.06854931	17.3064061

cg21791967	0.02082876	2569.54574	3.29642114	2002949.57	cg15694117	0.04004965	0.14965109	0.02441369	0.91733154
cg01084871	0.02082933	321793.626	6.86623291	1.5081E+10	cg14844588	0.0400537	12.3613142	1.12095473	136.314237
cg07918226	0.02083393	1033892.8	8.19489042	1.3044E+11	cg27123975	0.04008036	7912.43457	1.50146334	41697069.2
cg16792632	0.02083402	34.2209164	1.71018774	684.761732	cg26763524	0.04012787	458.08489	1.31789598	159224.832
cg13414916	0.0208393	42226459.6	14.387444	1.2393E+14	cg14688451	0.04013545	59.2393524	1.20169819	2920.28473
cg00157796	0.02084003	3.98202002	1.23346221	12.8552649	cg00976340	0.04015592	107.965173	1.23404329	9445.76142
cg26866325	0.02085055	5.42804593	1.29271987	22.7920087	cg15034464	0.04015993	5.1369E+13	4.1265724	6.39E+26
cg16723994	0.02085163	10.7865654	1.43470956	81.0965475	cg11872076	0.04016549	20063.8538	1.55975794	258090195
cg25102206	0.02085533	12.4471863	1.46614038	105.673678	cg00720475	0.04016652	112.979018	1.23626525	10324.8542
cg18706879	0.02086991	403163.091	7.08078164	2.2955E+10	cg16557944	0.04017171	6.26621481	1.08577376	36.1635634
cg25385322	0.02087078	0.00374613	3.27E-05	0.42859139	cg05945782	0.04017937	4.7918839	1.07272981	21.4053447
cg04672495	0.0208738	9.8279768	1.41411064	68.3037985	cg07824564	0.04019608	2446626.65	1.9307723	3.1003E+12
cg02375289	0.02087411	4.60894017	1.26071763	16.8493951	cg20972917	0.0402074	15.0206437	1.12866232	199.900123
cg01178040	0.02087635	22.3923148	1.6020911	312.975812	cg18229914	0.04021013	14274.705	1.53297487	132922728
cg05713859	0.0208821	110955460	16.5733601	7.4283E+14	cg05081953	0.04021524	55.1278144	1.19597931	2541.07733
cg09976989	0.02088302	804580.068	7.85373964	8.2426E+10	cg21940081	0.04021685	0.00289442	1.09E-05	0.77040637
cg25468277	0.02088653	15291849.3	12.2671526	1.9062E+13	cg05228295	0.04022666	21469696	2.12248659	2.1717E+14
cg00500989	0.02088694	17387.5106	4.3909713	68851628.6	cg20516737	0.04026577	4.0206E+10	2.95626428	5.47E+20
cg14376467	0.02089379	9.40919948	1.40438692	63.0403442	cg18501409	0.04028544	894.669304	1.35130012	592342.996
cg01123729	0.0208945	70243391.7	15.4408684	3.1955E+14	cg00955482	0.04028638	3835.45934	1.44124819	10206950.1
cg10671946	0.02089734	9.23176997	1.40026907	60.8637143	cg14399447	0.04029561	23713493.4	2.12000339	2.6525E+14
cg00749852	0.02089929	172.464055	2.18154068	13634.3322	cg24028828	0.04029829	18000.5435	1.54254458	210055237
cg17408972	0.02090708	6.20715154	1.31840254	29.2237986	cg27347140	0.04029859	203.03006	1.26495317	32587.1394
cg05587419	0.02090836	38.2993086	1.73656033	844.679573	cg25594549	0.04032457	24.7326898	1.15201456	530.988032
cg09655862	0.02091549	6.26078941	1.3199861	29.6953763	cg11268628	0.04033028	52.0803795	1.19035526	2278.61884
cg21987515	0.02092187	4.84829749	1.2697978	18.5115997	cg07962315	0.04033101	8.12563856	1.09674661	60.2016924
cg13001868	0.02092639	2.96838127	1.17891216	7.474083	cg15821095	0.04033588	12.7942759	1.11884836	146.305345
cg05030680	0.02092851	7.29458345	1.35064175	39.3967888	cg06641593	0.04038404	0.11334028	0.01413225	0.90898593
cg11331678	0.02092997	5.18419356	1.28262006	20.9538769	cg27289153	0.04039185	8.86734651	1.1002793	71.4635221
cg21461649	0.02093157	3.62430791	1.21500702	10.8111374	cg03415695	0.04039281	390.851374	1.29864262	117634.209

cg07252933	0.02093449	12.2565672	1.46078735	102.837308
cg26493224	0.02093584	9.01784818	1.39452241	58.3150081
cg12508343	0.02094051	5.79080288	1.30410821	25.7136622
cg04656330	0.02094326	5610669332	29.7655206	1.0576E+18
cg00438309	0.02094866	0.08094966	0.00958145	0.68391
cg17869426	0.02095554	1007.87877	2.84293616	357313.549
cg04971769	0.02096832	0.06071194	0.00562695	0.65505097
cg10998878	0.02096849	405903.044	7.02849358	2.3441E+10
cg14876761	0.02097589	8.63619994	1.38464132	53.8651768
cg16944926	0.02097864	11326.675	4.09157179	31355570.4
cg04170535	0.02098763	5.78778673	1.30329131	25.7029836
cg01313977	0.02100031	10.1096384	1.41743072	72.1056678
cg23504474	0.0210071	7.99265062	1.36796236	46.6989925
cg03528946	0.0210176	4406309552	28.3860836	6.8398E+17
cg15132013	0.02101833	6.07084436	1.31223322	28.085824
cg13828592	0.0210226	8124600.9	10.9873951	6.0077E+12
cg06451479	0.02102297	20.9851	1.58172106	278.414716
cg16683920	0.02102583	17.3430384	1.53686517	195.710716
cg27621997	0.02103873	7.61989821	1.35757273	42.7696045
cg15154229	0.02104517	10.2899504	1.42023183	74.5533775
cg23842796	0.02104628	4.33274773	1.24687405	15.0558133
cg18488946	0.02104772	0.1443668	0.02788773	0.74734553
cg11724135	0.02104992	4.99797119	1.27391465	19.608626
cg18915287	0.0210562	16830.3357	4.32196802	65539633.5
cg06630567	0.02105805	8.66970859	1.38382621	54.3159586
cg15213225	0.02106186	75.0837256	1.91446003	2944.72894
cg10548700	0.02106254	0.00098532	2.75E-06	0.35310572
cg17030981	0.02106504	0.133304	0.02405885	0.73860376
cg17480760	0.02106788	3.93804963	1.22883975	12.6202256
cg05735639	0.02108107	12.3197215	1.45836836	104.072154

cg10383568	0.04040664	10.5669936	1.10857598	100.725035
cg25924411	0.04042599	125943.624	1.66916666	9502823580
cg19815813	0.04043023	4.5938855	1.06874672	19.7462913
cg25748136	0.0404429	7.02408265	1.08859136	45.3225508
cg08260245	0.04045417	29.0099143	1.15773212	726.916971
cg02362385	0.04045533	4781.81379	1.44547281	15818867.6
cg19280530	0.04046689	11683.9937	1.5019583	90891810
cg15547624	0.04050874	177793.469	1.68639145	1.8744E+10
cg04202511	0.04051046	7.64227498	1.09188085	53.489689
cg14856108	0.04053477	8591572.2	1.99023702	3.7089E+13
cg03739476	0.04054143	33.4794514	1.16326772	963.556062
cg07294541	0.0405633	9.80744596	1.10308191	87.1975105
cg01915791	0.04056338	5.98596704	1.07992509	33.1798952
cg25794884	0.04061314	437.304797	1.29672696	147475.522
cg23261237	0.0406149	43261.544	1.57788542	1186119837
cg19896639	0.04061642	504.794796	1.30457747	195325.914
cg17400113	0.04062955	14.6902411	1.12144862	192.432521
cg03988279	0.04063611	316518394	2.30312097	4.3499E+16
cg07264792	0.04063994	1721359.33	1.84366038	1.6072E+12
cg13485756	0.0406499	11995038.5	2.00109124	7.1901E+13
cg21388527	0.04065204	27.6989298	1.15179011	666.120245
cg10248148	0.04065285	16367.132	1.51104812	177282912
cg10733616	0.04066409	181414461	2.24343097	1.467E+16
cg27303326	0.04067017	1.62E-06	4.63E-12	0.56774072
cg16002441	0.04068524	42.9819048	1.17282738	1575.2055
cg10139651	0.04069703	7.25225897	1.08749358	48.3637431
cg09473613	0.04070195	5.54994568	1.07520378	28.6474971
cg00414306	0.04072264	3874.03856	1.41729857	10589282.3
cg00002719	0.04073977	3.88618527	1.05885513	14.2629861
cg07687684	0.04075359	10623423	1.97493662	5.7145E+13

cg19956166	0.02108439	995.717935	2.82108661	351444.086	cg07397616	0.04078479	15.9724926	1.12315606	227.146101
cg00676042	0.02108615	13979680.2	11.8413216	1.6504E+13	cg16685860	0.04079578	6285461.75	1.92575541	2.0515E+13
cg25346193	0.02108678	53.6071599	1.81868534	1580.11258	cg09313258	0.04079937	190.117195	1.24557109	29018.4545
cg13285862	0.02109317	134271.707	5.88966955	3061104044	cg12351660	0.04080091	10.3603482	1.10276294	97.3344412
cg13332774	0.02110834	32.5060177	1.6861905	626.644016	cg06549777	0.04080871	68520770.1	2.12594378	2.2085E+15
cg26883434	0.0211099	22.3730614	1.5942077	313.982848	cg25185881	0.04082868	127.599563	1.22413238	13300.562
cg19828362	0.02111602	286.412468	2.33671894	35105.6776	cg10260334	0.04083253	0.09971878	0.01094696	0.90836511
cg15773138	0.02111981	68.4287457	1.88487675	2484.24373	cg25145905	0.04084249	25618.887	1.52608144	430073620
cg18720905	0.02112215	8.51569217	1.37885051	52.5923679	cg22397447	0.04085449	1.22E-08	3.16E-16	0.46868325
cg00336946	0.02112256	5.73751796	1.29955211	25.3311215	cg03278924	0.04087243	372218861	2.26818471	6.1083E+16
cg08039823	0.0211236	2.2139E+10	35.6039164	1.3766E+19	cg26626663	0.04087323	6.68700514	1.08203932	41.3257051
cg02806715	0.02112676	6.50610107	1.32421827	31.9655392	cg25291004	0.04087746	6.57610211	1.08124781	39.9955669
cg05146399	0.02115122	0.11607165	0.01860151	0.72427589	cg18709306	0.04090264	33.3563589	1.15608967	962.422471
cg14945182	0.02115702	96.5247967	1.98246694	4699.71841	cg00417576	0.04091446	20.2483334	1.13227721	362.097728
cg09170903	0.0211617	138.343378	2.0919407	9148.86845	cg04346128	0.04091894	14.5357685	1.11681949	189.18775
cg23788592	0.02116782	2284478.3	8.94944551	5.8315E+11	cg10232654	0.04092207	12.3213989	1.10918476	136.872482
cg24547885	0.02117745	3.82389291	1.2222321	11.9634864	cg02271139	0.04092534	11.7544273	1.10698849	124.813007
cg08642731	0.02118177	4.75421948	1.26265407	17.9008673	cg24315815	0.04093062	7.23314775	1.08498448	48.2204375
cg10933774	0.02118262	19.2734413	1.55671847	238.620886	cg19539556	0.0409545	0.17392048	0.03250348	0.93061832
cg12135835	0.02118723	22.0240714	1.58794707	305.463407	cg03408033	0.04095541	5390.77376	1.42360109	20413332.1
cg02117713	0.02118794	1927.06942	3.09923851	1198228.71	cg11505048	0.04095566	18.5078287	1.12743816	303.821296
cg23410129	0.02118927	0.08159978	0.00968556	0.68746902	cg02153299	0.04095908	25710.5886	1.51774647	435536752
cg25782440	0.02118951	52.4690956	1.80797758	1522.69919	cg23654545	0.04095937	2.00E-07	7.51E-14	0.53056339
cg03660115	0.02119416	5.83758287	1.30184242	26.1762662	cg00564831	0.04096792	171.160721	1.23501049	23721.25
cg11350728	0.02119488	1692.27113	3.03856593	942478.01	cg19884658	0.04097381	4.97521634	1.06802481	23.1762198
cg21611775	0.02119971	0.19639173	0.04919316	0.78404622	cg13801056	0.04099349	11.5011926	1.10512069	119.69501
cg10726226	0.02120185	82.8852376	1.93522642	3549.95289	cg03446876	0.04099613	2641.96613	1.38037059	5056602.29
cg05293820	0.02121283	3.81215921	1.22128959	11.8993545	cg05583848	0.0410009	7.45834366	1.08562849	51.2393426
cg18357526	0.02121531	314.146928	2.36045272	41809.0528	cg04943729	0.0410099	7528621.61	1.90935334	2.9686E+13
cg10531073	0.0212163	5.59390299	1.29324518	24.1963017	cg21440584	0.04101448	5.62149775	1.07303068	29.4504504

cg26971710	0.02122319	3003.61528	3.30577619	2729073.07
cg20703523	0.02122332	56152.568	5.11870996	615997180
cg21153962	0.02122416	821.799633	2.72400145	247927.415
cg10554624	0.02123388	14.3589398	1.48832354	138.531137
cg03067403	0.02123539	11.7613592	1.44461538	95.7552932
cg08526991	0.02123701	4.21522804	1.23948139	14.3351466
cg20693580	0.02123851	6.12016756	1.31038327	28.5843478
cg15977246	0.02124018	9256709.61	10.9505574	7.8249E+12
cg21384203	0.02124329	5.92911256	1.30412367	26.9563205
cg08224785	0.02124337	6.20280549	1.3129319	29.3044872
cg19795338	0.02124871	9.38875792	1.39657517	63.1178166
cg15888699	0.0212492	15499.9722	4.21670809	56975520.7
cg00402417	0.02125141	16.0207712	1.51236587	169.71099
cg15140822	0.02125881	539868.396	7.15466409	4.0737E+10
cg02043159	0.0212592	3.87048932	1.22355823	12.2435428
cg06459916	0.02125957	3.0248E+13	102.254021	8.95E+24
cg00528640	0.0212696	36013.7955	4.77497306	271623201
cg21805880	0.02126992	10.0256093	1.40985992	71.2927855
cg06893364	0.02126992	16933.8122	4.26705673	67201824.4
cg25599242	0.02127249	13.6946802	1.47685851	126.988648
cg07914457	0.0212733	12.9086595	1.4638891	113.829312
cg21654383	0.02127579	13.331553	1.4708744	120.833094
cg06065608	0.0212792	11.5642471	1.43996117	92.8718167
cg21573582	0.02128126	721273092	20.8588001	2.4941E+16
cg10064585	0.02128393	25886.5045	4.54137951	147556731
cg05211836	0.02128689	5.43614656	1.28672013	22.96668
cg07390023	0.02129197	24.3910677	1.60883304	369.786156
cg27456951	0.02130349	7.57687547	1.35163473	42.4737841
cg16966043	0.02131137	3830.32366	3.41164725	4300379.93
cg14688451	0.02131256	45.8919442	1.76665143	1192.12568

cg08052882	0.04104227	382.030378	1.27371331	114584.035
cg11457534	0.04104368	0.11191102	0.01369125	0.9147506
cg26512283	0.04106289	140.3009	1.2222472	16105.0421
cg07400063	0.04109408	20.2467318	1.12937375	362.971203
cg09026415	0.04109664	170482.302	1.62757318	1.7857E+10
cg10509187	0.0410996	7.00301806	1.08184948	45.3318718
cg02618319	0.04110211	7.84452072	1.08679639	56.6219267
cg05678729	0.04110837	11703567.9	1.9293494	7.0995E+13
cg18605031	0.0411223	6.57000603	1.07884348	40.0104188
cg00671759	0.04114074	8496.42436	1.43898201	50166872.4
cg11439192	0.04115224	6361597.92	1.87631581	2.1569E+13
cg21760146	0.041163	10.4653706	1.0987846	99.677391
cg14633527	0.04117572	9499567.25	1.90337856	4.7411E+13
cg12649539	0.04117783	875.931774	1.31173213	584918.563
cg01636650	0.04118395	11.7597337	1.10366716	125.301668
cg14383549	0.04120015	11.5566321	1.10269084	121.118033
cg02992632	0.04120564	6.5014714	1.07759068	39.2255902
cg25767504	0.04121126	28.1875757	1.14247005	695.457551
cg18394090	0.04121591	2285.94657	1.36120628	3838912.43
cg25756617	0.04122068	23.8057499	1.13463549	499.467655
cg13629565	0.04123635	881769758	2.26871599	3.4271E+17
cg01640991	0.04123663	0.00559056	3.84E-05	0.81360456
cg01652532	0.04128958	6834.59125	1.41758942	32951457.4
cg09372028	0.04129225	1696.9569	1.3415226	2146562.95
cg15160780	0.04132166	1515.53183	1.33418298	1721530.52
cg01381679	0.04133491	39334.6782	1.51566543	1020816918
cg22488745	0.04134014	7.27070342	1.08104558	48.8999994
cg24569831	0.04137791	9.60715159	1.09250219	84.4825418
cg04283162	0.04140435	3.00061802	1.0437601	8.62622411
cg09284397	0.04140495	296.657775	1.24840786	70494.4582

cg00670698	0.02131405	857369.84	7.62754621	9.6372E+10
cg24580593	0.0213145	1069360.19	7.88201866	1.4508E+11
cg26955540	0.02132095	30.6011412	1.66300138	563.096252
cg23295623	0.02133149	0.19054846	0.04645223	0.78163555
cg13065262	0.02134789	413.786824	2.44674111	69978.6076
cg00753089	0.02135241	20.1637641	1.56202718	260.288291
cg00390941	0.02135387	0.04737102	0.00352905	0.63586839
cg11987645	0.02136095	50.0888418	1.78757296	1403.5187
cg07823452	0.02136432	4.4749E+13	106.085147	1.89E+25
cg15623892	0.02138504	90.0880905	1.94890068	4164.32923
cg03079712	0.02138598	116.578514	2.02476374	6712.16584
cg11778001	0.0213882	44.711853	1.75650599	1138.14004
cg11832722	0.0213892	4.8910518	1.26528604	18.9067034
cg27419119	0.02139555	0.13024694	0.02294639	0.73929978
cg00494710	0.02140111	0.05884034	0.00526772	0.65724514
cg04788444	0.02140187	81487.25	5.34015122	1243442676
cg24801889	0.02140342	920398.162	7.64667686	1.1078E+11
cg10889792	0.02140585	0.14162994	0.02679416	0.74863469
cg12099952	0.02141095	3.69499174	1.2135436	11.2504932
cg20241658	0.02141151	189690.237	6.04759922	5949862872
cg12531003	0.02141654	1487.39508	2.94899854	750201.833
cg20780850	0.02141794	41.3547378	1.73504269	985.690064
cg27252587	0.02142219	41653375.9	13.4214722	1.2927E+14
cg21287322	0.02142424	6.25325053	1.31166386	29.8118622
cg25880288	0.02142623	27847.2924	4.54740685	170530529
cg13995374	0.02142961	11117.2322	3.96880389	31141083.3
cg03547606	0.0214297	68.1951306	1.86778762	2489.88471
cg16636114	0.02142989	236.53405	2.24514907	24919.6624
cg05106869	0.02144323	12.0207824	1.44442289	100.039407
cg07879540	0.02144422	1332.76913	2.89768412	612997.649

cg01651570	0.04142979	4.22318179	1.05757449	16.8643103
cg26257177	0.04143266	1.9983E+19	5.61977032	7.11E+37
cg14039939	0.04143295	5503.68484	1.3973255	21677516.7
cg05713474	0.04143478	1.33E-10	4.29E-20	0.41351872
cg21367838	0.04144253	7106.8373	1.41070341	35802803.1
cg24287110	0.04145187	74.8588088	1.18204331	4740.80872
cg10770023	0.04145727	10.8147657	1.09659268	106.656884
cg04411541	0.04147729	8.72141774	1.08727166	69.9577946
cg02931001	0.04148501	12.9608752	1.10393696	152.168369
cg03429645	0.04150031	7.98546785	1.0833302	58.8626594
cg16127594	0.04152082	7.65377533	1.08134802	54.1733798
cg07218516	0.04152166	910568141	2.20928633	3.753E+17
cg26213155	0.04153241	0.14467029	0.02254123	0.92849824
cg01951603	0.04153475	27211.9342	1.47954058	500486013
cg00131871	0.04154043	94.1287645	1.19031875	7443.57286
cg02317785	0.04156223	1293955425	2.23034093	7.507E+17
cg16278496	0.04158769	9.19579245	1.08823989	77.7058437
cg01067724	0.04160668	3.4401E+16	4.25370163	2.78E+32
cg21233517	0.04163624	75.6987908	1.17811128	4863.97766
cg00630431	0.04164593	5938.42355	1.38927181	25383711.1
cg14611399	0.04164837	5.75970237	1.06847184	31.0482412
cg15240082	0.04167204	108.329845	1.19327326	9834.59165
cg15287092	0.04167258	51.3317805	1.16011836	2271.27832
cg14591123	0.04167331	20.7722295	1.12119355	384.844807
cg06727703	0.04168136	37.5897492	1.14639453	1232.55058
cg13649519	0.04169747	67.7459009	1.1717415	3916.82558
cg15205428	0.04170471	5.165096	1.06361333	25.0826272
cg15109933	0.04170974	53112276.7	1.94976001	1.4468E+15
cg19081101	0.04171076	4.74721919	1.06020123	21.256427
cg06746893	0.04173063	0.19774054	0.04154748	0.94112381

cg20445950	0.02144859	244.82424	2.25510446	26579.2159
cg18703238	0.02144977	6.42322372	1.31647135	31.3396892
cg01599041	0.0214509	3129951.89	9.12426251	1.0737E+12
cg12529273	0.02145303	10.7787007	1.42109782	81.7539696
cg19744936	0.0214578	5.26485199	1.27822051	21.6853557
cg03892781	0.02146956	0.16841977	0.03690198	0.76866395
cg10016610	0.02147466	10.8106972	1.42124191	82.2317249
cg20928429	0.0214771	6.03910547	1.30410598	27.966128
cg02341811	0.02149188	316347460	17.957805	5.5728E+15
cg01534125	0.02149481	5.29254718	1.27869649	21.9059455
cg12785251	0.0214983	0.00030536	3.08E-07	0.30301197
cg26543539	0.02149864	15.8048679	1.50257553	166.24379
cg23796713	0.0215002	0.03245808	0.00174674	0.60313852
cg25507001	0.02150642	18.8103227	1.5414316	229.545208
cg25436985	0.0215081	21923.4802	4.36585308	110090508
cg01554410	0.02151286	8.55925999	1.37233196	53.3842638
cg13870539	0.02151688	7.0920905	1.33474485	37.6834177
cg12821724	0.02151702	18.1991118	1.53363356	215.962716
cg20459849	0.02151893	5.01877828	1.26839335	19.8582997
cg01601628	0.02152081	43.6441758	1.74450317	1091.89488
cg14079785	0.02152528	11.6373731	1.43562571	94.3340951
cg27179622	0.02152628	5.20101482	1.27497745	21.2164969
cg18086594	0.02153145	85433.4726	5.32621722	1370368111
cg23818978	0.02153232	0.01860003	0.00062218	0.55604851
cg19601328	0.02153252	8.76682686	1.3768043	55.8229324
cg18384277	0.02153965	31.231001	1.65985287	587.627637
cg10549973	0.02154062	77.3270289	1.8968563	3152.30489
cg00908526	0.02154095	6.3463286	1.31268415	30.682085
cg06637001	0.02154448	4.58642591	1.25134659	16.8101329
cg20546782	0.02155131	59.980804	1.82670012	1969.506

cg13862524	0.04173169	168109.468	1.56896436	1.8012E+10
cg15775835	0.04174488	2.0636E+10	2.42925764	1.75E+20
cg07953400	0.04174758	3287606.53	1.7517136	6.1702E+12
cg06191203	0.04175677	212.361448	1.22133884	36924.5481
cg07568203	0.04179763	0.11328965	0.01391528	0.92233452
cg04039397	0.04180251	5.23212566	1.06331799	25.7450163
cg11323985	0.04181185	10.7508335	1.09199697	105.843169
cg04467350	0.0418139	2152867740	2.21695614	2.0906E+18
cg06846310	0.04182157	10.4731442	1.09082143	100.554267
cg24237813	0.04182335	246.707285	1.226072	49641.8515
cg02456292	0.04182668	84.8985459	1.17853738	6115.85445
cg14934978	0.04183506	2627.80321	1.33765482	5162280.76
cg02929073	0.0418442	7.42544167	1.07679552	51.2048787
cg00473834	0.04185094	2.1781E+11	2.61855975	1.81E+22
cg05092885	0.04185309	17931.353	1.43482177	224092934
cg18011163	0.0418558	7.91045363	1.07919102	57.9835038
cg04867634	0.04186252	146.52258	1.20155641	17867.5477
cg13749822	0.04186394	6.17314816	1.06929997	35.6380428
cg27256066	0.04186536	10.988144	1.09222175	110.544684
cg24803730	0.04187788	18.5641155	1.11331571	309.549556
cg11609545	0.04187878	3144861.05	1.73273305	5.7078E+12
cg07616871	0.04187932	14.1639081	1.10228336	182.000654
cg02140384	0.04189309	6.64403461	1.07191921	41.1814581
cg27049761	0.04190065	4.75141339	1.05876088	21.3229725
cg04468971	0.04191351	8286494926	2.30566559	2.9781E+19
cg13308525	0.04192852	113928.782	1.52969883	8485178389
cg07846311	0.04193438	5.02996652	1.06070146	23.8526713
cg07266404	0.04195076	5.48981135	1.06395245	28.3264806
cg21287322	0.04195533	6.33312234	1.06945916	37.5034783
cg14267284	0.04195681	9053229416	2.30237551	3.5598E+19

cg10087172	0.02155412	6.7136638	1.32338795	34.0590086
cg09827065	0.02155474	2140.19408	3.09074447	1481982.97
cg10985065	0.02156318	0.1379973	0.02548346	0.74727888
cg05666423	0.02156429	63853.811	5.09055574	800955611
cg16881309	0.02157101	681.18308	2.60977496	177797.088
cg24516901	0.0215711	9.30810476	1.38822787	62.4110898
cg03289681	0.02157294	37.6913187	1.70510325	833.166851
cg24524538	0.02157516	1408843.68	8.01608166	2.4761E+11
cg16856833	0.02158314	7.62399633	1.34786486	43.1239971
cg24502126	0.02159323	31.6172536	1.6608546	601.88937
cg01458759	0.02159334	6004955732	27.3154782	1.3201E+18
cg17730428	0.02159381	185.657683	2.15404258	16001.9007
cg07800524	0.02159725	23.6859285	1.59173221	352.460799
cg06132694	0.02160029	53.013466	1.7915324	1568.7283
cg15433297	0.02160434	9.25095726	1.38630321	61.7326784
cg22547764	0.02160458	0.0001342	6.67E-08	0.27006743
cg11258089	0.0216117	5.15306959	1.27207692	20.8746231
cg20101015	0.02161294	5.12350622	1.27098638	20.653499
cg13580767	0.0216266	267185.435	6.25162602	1.1419E+10
cg12805580	0.0216297	9601875185	29.106398	3.1676E+18
cg03023152	0.02163545	359.052155	2.36939628	54409.8304
cg00472687	0.02163939	1804.83259	3.00175958	1085170.4
cg01356198	0.02165356	5.73463362	1.29158461	25.4617641
cg21075077	0.02165452	8025657344	28.2467095	2.2803E+18
cg16823292	0.02165963	0.20609234	0.05352888	0.79347921
cg19477600	0.02166016	718804.344	7.2070191	7.1691E+10
cg16663033	0.0216625	5.69831867	1.29025256	25.1662633
cg04164415	0.02166504	4.87884436	1.26121358	18.8731891
cg00599564	0.02166762	6.06833122	1.30211655	28.280605
cg27160726	0.02167058	4.54919457	1.2482918	16.5787929

cg07274204	0.041963	24.6399959	1.12351805	540.382415
cg01175550	0.04197918	5.12606874	1.06106894	24.764254
cg04614997	0.04200149	5.85547378	1.06600381	32.1636499
cg20852788	0.04201806	19.8974977	1.11396043	355.407969
cg21745091	0.04202744	1821686588	2.15662705	1.5388E+18
cg20696432	0.04202787	12.3476057	1.0948174	139.259173
cg03122532	0.04203583	8.96223945	1.08215733	74.2237137
cg02478520	0.04203837	7.1926E+13	3.15306458	1.64E+27
cg02135580	0.04204094	1851030.41	1.68073396	2.0386E+12
cg15267303	0.042049	2.99E-08	1.67E-15	0.53649686
cg02529547	0.04209012	41.4259096	1.14239303	1502.20278
cg09819651	0.04210988	415500645	2.0291065	8.5082E+16
cg05230647	0.04211089	420.557701	1.24034615	142596.307
cg10732920	0.04216041	79725.9106	1.49145582	4261756023
cg10457563	0.0421654	11.5266538	1.09038473	121.850337
cg00500522	0.04217658	13905.6018	1.40098693	138021103
cg07974719	0.04220162	9.79770854	1.08371051	88.5800146
cg13722539	0.04221454	2654.92466	1.31949618	5341906.29
cg06074332	0.04222681	19191985.9	1.80175266	2.0443E+14
cg08416638	0.04223808	0.03297424	0.00122545	0.88726729
cg11856688	0.04228454	2.0144E+15	3.41317083	1.19E+30
cg18476690	0.04229571	16.0066144	1.10126487	232.652209
cg01317270	0.04229584	21228.3532	1.41419765	318656291
cg07533422	0.04229934	5.29183997	1.05964046	26.4274265
cg02191862	0.04230066	12.3821508	1.09140968	140.476727
cg15140822	0.04232322	256625.022	1.5398096	4.2769E+10
cg05986680	0.04233901	7.9193E+12	2.79299008	2.25E+25
cg01010839	0.04234922	14.2462924	1.09608137	185.165858
cg18929217	0.0423631	9.47404908	1.08059011	83.0635084
cg05526364	0.04236985	5.58657738	1.06103665	29.4144852

cg25748136	0.02169095	7.37566073	1.33944184	40.6142093	cg12855522	0.04237186	5.88501211	1.06292222	32.5831626
cg20345923	0.0216915	0.17907012	0.04123783	0.77758963	cg16184495	0.04237919	6.72451565	1.06774367	42.3501559
cg16107740	0.02169388	1.2882E+10	30.0943801	5.5142E+18	cg20859525	0.04238608	38044080.2	1.82169618	7.9451E+14
cg19654882	0.02169859	38.3451498	1.70432967	862.714849	cg08738571	0.04238748	18.1168352	1.10464404	297.127135
cg25920207	0.02169937	19618.8593	4.24236407	90727630.3	cg14058329	0.0423909	11.7024371	1.08813897	125.85436
cg15787985	0.0217022	17.4403008	1.51879046	200.267318	cg08535411	0.04239302	29796.9634	1.42428702	623370864
cg13216025	0.02170585	3883527.85	9.18537667	1.6419E+12	cg20362242	0.04239646	25.7788963	1.11795867	594.432974
cg20234170	0.02170598	161.71717	2.10298022	12435.8958	cg22334684	0.04241824	5.95E-05	4.94E-09	0.71688117
cg21754746	0.02170689	55.3486763	1.79789735	1703.92151	cg06479512	0.04241989	149.90694	1.18692207	18933.0802
cg24948406	0.02171354	10.007415	1.40010112	71.5293733	cg05928186	0.04243047	20.0263786	1.10778591	362.033706
cg05205736	0.02171554	13.952277	1.46971106	132.451907	cg07505018	0.0424368	5.14132599	1.05746381	24.9968203
cg22043720	0.02171998	2818.9067	3.19120813	2490039.72	cg10726226	0.04244044	76.5816422	1.15948168	5058.07726
cg16142824	0.02172041	0.17712748	0.04039923	0.77660258	cg11253514	0.04246406	8.85953299	1.07698525	72.8805942
cg09146903	0.02172234	11.4509096	1.42774003	91.8397813	cg21330423	0.04247482	9.10997841	1.0778867	76.9948332
cg01836913	0.02172933	20.7573476	1.55711267	276.70925	cg15317837	0.0424863	8619952.82	1.71818655	4.3245E+13
cg19507527	0.02173267	3.74886723	1.21278165	11.5882406	cg09674867	0.04249017	8.21486829	1.07394663	62.8374435
cg24841124	0.02173978	165507081	15.8296141	1.7305E+15	cg14609668	0.04250469	26.8266939	1.11762656	643.928423
cg21926698	0.02174059	2.9203E+11	47.1223344	1.81E+21	cg00758533	0.04253946	26.3166988	1.11630864	620.409631
cg15257224	0.02174113	727.53729	2.61601154	202334.929	cg07318155	0.0425465	149268.277	1.49248547	1.4929E+10
cg06346307	0.02175493	56.4977872	1.80103692	1772.31234	cg06508879	0.04257384	15.3944687	1.09586926	216.257244
cg20162652	0.02175689	4.34357193	1.23884741	15.2291694	cg06798642	0.0425937	258.954554	1.2038789	55701.1681
cg24377657	0.02176461	0.00386613	3.36E-05	0.44490899	cg02571738	0.04261297	11.2698085	1.08400465	117.166087
cg14329685	0.02176727	1288.9551	2.84026818	584946.614	cg06191536	0.04263473	6.40042956	1.06357147	38.5169214
cg09269866	0.02177641	8.3080551	1.36136623	50.7018451	cg18575176	0.04264504	220603.4	1.5036772	3.2365E+10
cg07029862	0.02178782	103.657044	1.96576803	5465.94641	cg17794169	0.04264993	3608.99267	1.31176308	9929253.47
cg19515317	0.02179351	204.847256	2.17034579	19334.4297	cg19962750	0.04267254	6.659519	1.06461756	41.6573941
cg06439982	0.02180098	2.7173E+10	33.0093388	2.2369E+19	cg09069694	0.04267401	17.5692761	1.09925535	280.807788
cg01164291	0.02180411	4348.08292	3.38424808	5586418.2	cg12968701	0.04267572	0.13677063	0.01997589	0.93643915
cg12406822	0.02181059	16.012473	1.4970209	171.273021	cg06796435	0.042681	9.17295671	1.07584129	78.2114757
cg09371439	0.02181458	12.9785078	1.45186474	116.017464	cg16699850	0.0426811	415944.981	1.53228855	1.1291E+11

cg05928362	0.02181876	6.88361853	1.32386434	35.7923412
cg14762670	0.02182287	10.0627264	1.39893631	72.3824677
cg14037218	0.02182875	24.5508411	1.59245435	378.499891
cg19445684	0.02183615	10.3690339	1.40476645	76.5371804
cg00511731	0.02183774	1908.10638	2.99704787	1214818.75
cg22935921	0.02184244	6.45140742	1.3110633	31.7457271
cg22767408	0.0218496	1163842.55	7.60237877	1.7817E+11
cg11801857	0.02185048	427.123589	2.41001805	75698.4208
cg04692420	0.02185081	572.623248	2.5148014	130386.99
cg22401939	0.02185323	13.1455019	1.45362927	118.87778
cg10560368	0.02185534	1005736.16	7.43905292	1.3597E+11
cg01754155	0.02186628	59.1739103	1.80790269	1936.80317
cg09370982	0.02187364	6.74591708	1.31908443	34.4992301
cg15476790	0.02188364	11.788595	1.4301196	97.1743709
cg13969672	0.02188562	5804977.66	9.56598468	3.5227E+12
cg09731288	0.02188707	181.217376	2.12525358	15452.1502
cg00759508	0.0218924	11.1174132	1.41782099	87.1738225
cg27326372	0.02189588	70.9095037	1.85448607	2711.34833
cg04085707	0.02189795	36075.2717	4.57528117	284447048
cg05323898	0.02189856	105199.065	5.3426368	2071419715
cg11722699	0.02190178	5.27181244	1.27234964	21.8430575
cg26123256	0.0219175	4.61481121	1.24785275	17.0665029
cg20034372	0.02192114	5.03905292	1.26379632	20.0918882
cg14431006	0.02192238	6.13317637	1.30024355	28.9298512
cg11971423	0.02192802	8.25955363	1.3573895	50.2584013
cg00436603	0.02192869	0.12960494	0.02257684	0.7440121
cg07548313	0.02192941	0.23817218	0.06981592	0.81250783
cg15616400	0.0219301	6.82960592	1.32051948	35.322097
cg12436713	0.02193638	12556036.2	10.6409848	1.4816E+13
cg23725986	0.0219413	101.567807	1.95097194	5287.63086
cg23761196	0.04268209	63214.473	1.43989147	2775257501
cg27596707	0.04269709	169.01683	1.18391571	24128.9887
cg11052143	0.04271352	5.0857187	1.05485268	24.5195704
cg14960373	0.04272424	9.24351149	1.07563085	79.4347842
cg02965178	0.04272454	12.5820246	1.08655538	145.696525
cg09677935	0.04274162	26642951.3	1.74917507	4.0582E+14
cg24818238	0.04276714	14.0106818	1.08982164	180.120485
cg16679650	0.04277165	14.8410949	1.09180641	201.737318
cg16645133	0.04278382	7.99856608	1.06992401	59.795891
cg19223541	0.04279409	18614.5823	1.37590952	251835364
cg08293536	0.04279425	3702.81851	1.30564131	10501249.4
cg07604732	0.04280043	17.8726724	1.0980099	290.919434
cg01963633	0.04280851	0.18583443	0.03646916	0.94694898
cg24250450	0.04281111	7.4318E+12	2.61063552	2.12E+25
cg12235877	0.04284041	29.8538813	1.11572464	798.812001
cg06289844	0.04286508	6.19914907	1.060365	36.241718
cg13202410	0.04288344	8.99542698	1.07292284	75.4180109
cg11846968	0.04288577	0.17768255	0.03336753	0.94616187
cg23041619	0.04289414	83655.4486	1.43707749	4869768069
cg09109520	0.04290637	11.7677232	1.08191359	127.994796
cg02045285	0.04290936	111.924576	1.16252949	10775.7359
cg10277175	0.04291044	8.84812458	1.07206167	73.0268702
cg01184539	0.04292209	35.6509027	1.12060335	1134.1987
cg16898498	0.04293277	7.76949543	1.06739549	56.5536018
cg06476131	0.04293709	3.73680957	1.04279997	13.3906273
cg15768282	0.0429434	31597327.6	1.73065074	5.7689E+14
cg02506053	0.04294884	7939973466	2.06155534	3.058E+19
cg14930269	0.04295422	48800943.9	1.7531485	1.3584E+15
cg06146234	0.04296343	8.53085614	1.07024742	67.9987685
cg06895295	0.04297913	19.2910437	1.09802659	338.921089

cg04016086	0.02194501	0.01441507	0.00038361	0.54167787
cg26631329	0.02194907	25725.4736	4.34190243	152421664
cg00031187	0.02195184	7.22511214	1.33095139	39.2217522
cg19910382	0.02196272	0.12896519	0.02236045	0.74381432
cg18923051	0.02196283	17.7541797	1.51537913	208.007943
cg06710694	0.02196717	9.05E-07	6.11E-12	0.13394163
cg04252889	0.02197345	0.10949053	0.01650043	0.72653711
cg00419186	0.02197445	1920.0647	2.97979169	1237216.84
cg27596707	0.02197637	198.908637	2.14758171	18422.8827
cg07071389	0.02197658	4.55220829	1.24466733	16.6491076
cg23048494	0.02197891	10.3781701	1.40191543	76.8280391
cg22650315	0.02198262	10.81385	1.41018424	82.924875
cg03231960	0.02198545	162025.86	5.64954106	4646816283
cg14657834	0.0219881	5.06870207	1.26398269	20.3260225
cg26928531	0.02199363	36.0392248	1.67741781	774.300664
cg07702750	0.02200019	5.80408442	1.2887651	26.1392832
cg05799507	0.02200089	6.37732516	1.30638421	31.1319409
cg10537847	0.02200209	2702537.27	8.46735793	8.6257E+11
cg06375085	0.02200717	8.3217E+10	37.5683691	1.84E+20
cg15044767	0.02201084	0.11055395	0.01679012	0.72793868
cg11572305	0.02201222	488.30282	2.44147889	97661.9725
cg21035374	0.0220124	8.81215884	1.3685482	56.7419865
cg12804865	0.02201715	124267.403	5.42423159	2846926279
cg10614773	0.02201871	5851.99812	3.49147696	9808422.75
cg25154306	0.02202928	36328.6607	4.53932552	290741782
cg09840386	0.02203097	25.3002048	1.59269157	401.898504
cg25925006	0.0220447	4.69776014	1.24948771	17.6623989
cg08618068	0.02205353	750.7304	2.59309991	217344.55
cg04270652	0.02205938	7.67763098	1.34079476	43.9634902
cg02349739	0.0220662	830.328636	2.62953828	262192.663

cg00094858	0.04304233	4.76E-07	3.57E-13	0.63400086
cg26923779	0.04304973	2.226E+11	2.26370246	2.19E+22
cg00786685	0.04305003	14.7799821	1.08785818	200.805468
cg20154865	0.0430646	1051.35467	1.24244375	889655.283
cg16727916	0.04306927	15699511.4	1.67629731	1.4704E+14
cg20512519	0.04307243	78160831.5	1.76185192	3.4674E+15
cg16277607	0.04307843	11.5665981	1.07920282	123.967607
cg16332813	0.04311094	16.7571582	1.0912665	257.317853
cg06849960	0.04311413	7.19258329	1.06300951	48.6667842
cg23107161	0.04313894	32.2905755	1.11316948	936.677917
cg13455960	0.04314593	12.5359803	1.08105335	145.368221
cg07540542	0.04314827	1552.55278	1.25406153	1922090.81
cg22923514	0.04315781	25.3374748	1.10455913	581.216169
cg04189328	0.04315903	39.9582188	1.12012589	1425.42839
cg11354594	0.04319445	5.47608747	1.05340327	28.4672878
cg24547885	0.04319948	3.8179472	1.04180973	13.9917304
cg17636471	0.04321795	2.24E-15	1.40E-29	0.35757663
cg03478249	0.04322295	9.34094243	1.07044	81.511533
cg13752005	0.04322923	3602.3624	1.28305559	10114148.6
cg12938320	0.04323131	5.95457139	1.05578443	33.5834851
cg14807549	0.04324333	1462390103	1.89819654	1.1266E+18
cg23302730	0.04324776	4.75110755	1.0484319	21.5302709
cg01381613	0.04324821	0.01247702	0.00017783	0.87543183
cg19973659	0.04326877	163152.191	1.43777385	1.8514E+10
cg10370375	0.0432706	0.05237809	0.00299941	0.91466641
cg07513446	0.04327493	9.82561438	1.07150055	90.1004653
cg17200441	0.04328861	13.5987056	1.0818976	170.926336
cg10054857	0.04331054	0.2070886	0.04496427	0.95377259
cg05927159	0.04332062	450770.399	1.47803271	1.3748E+11
cg00398048	0.043341	13.7501281	1.08157056	174.806925

cg12995933	0.02207496	1392610.1	7.64413992	2.5371E+11
cg19169234	0.02207933	44502.7693	4.65790525	425190374
cg02918577	0.02208088	4.91958045	1.25734801	19.2486658
cg00869941	0.02208098	21.7065742	1.55638291	302.73743
cg10758227	0.02208252	13.321245	1.45086357	122.310307
cg22409775	0.0220843	603720507	18.2782904	1.9941E+16
cg27280575	0.02209849	5.84335211	1.28857776	26.4980237
cg26201826	0.02210124	2622682.14	8.3515852	8.2361E+11
cg13661211	0.02210948	1178980.1	7.44014222	1.8682E+11
cg20874811	0.02211742	34484.6384	4.47875005	265518342
cg02135580	0.02211776	2763836.17	8.40143407	9.0922E+11
cg01915791	0.02212581	5.73491361	1.28472134	25.6002862
cg15597770	0.02213002	294758.404	6.08743262	1.4272E+10
cg10824705	0.02213199	6.02800983	1.2938477	28.0843739
cg26827725	0.02213285	9.66342542	1.38442729	67.4515674
cg23130075	0.02214226	37.5008175	1.68122575	836.47976
cg23694882	0.02214824	47.8500512	1.74073651	1315.32107
cg00417297	0.02215007	49899.5091	4.71192514	528438151
cg08516222	0.02215196	20.4314864	1.54077494	270.932258
cg22546130	0.02216244	32.3325549	1.64512488	635.449696
cg23253991	0.02216493	95.5396029	1.92112152	4751.29534
cg18785300	0.02216555	10376.5436	3.75895943	28644272.4
cg07684809	0.02216933	3.66986855	1.2045917	11.1804981
cg26797270	0.02217418	3.03E-05	4.07E-09	0.22554625
cg06002947	0.02217658	984488.379	7.20703755	1.3448E+11
cg02311932	0.02217913	8.57733093	1.3600892	54.0924861
cg07728579	0.02218657	7.65366626	1.33796242	43.7819525
cg25014411	0.02218936	34.1005335	1.6567046	701.903271
cg01596834	0.02219609	4.91726357	1.25578061	19.2545424
cg04513669	0.02219849	189.656386	2.11692453	16991.4157

cg17368254	0.04334507	8.13085699	1.06466135	62.0956471
cg18236745	0.04335029	88512.2298	1.40535894	5574671780
cg00337957	0.04336137	20376.4091	1.34434329	308848232
cg23242944	0.04337529	41.4311484	1.1171948	1536.47337
cg04911307	0.04337656	5847.59083	1.29442447	26416619.2
cg12819417	0.04338014	37.4717789	1.11376973	1260.70424
cg27571057	0.04338096	0.01165335	0.00015502	0.87601528
cg08627125	0.04338499	7.48836435	1.06164906	52.819338
cg04003582	0.04339477	6.45522186	1.05688694	39.4270075
cg10759590	0.04343297	54.4863928	1.1251368	2638.58316
cg10575841	0.04344599	74865238.1	1.70509164	3.2871E+15
cg11993436	0.0434631	2276.34969	1.25470324	4129875.29
cg26495758	0.04346536	222038.731	1.43506462	3.4355E+10
cg13447915	0.04346552	645.170535	1.20903136	344279.753
cg21115558	0.04347012	0.23166811	0.05602143	0.95802824
cg04206724	0.04349307	2496.47236	1.25674349	4959145.85
cg09713758	0.043494	0.11117616	0.01317918	0.93785368
cg04667246	0.04350538	5021088754	1.91795562	1.3145E+19
cg00982136	0.04351365	4.27968744	1.04324293	17.5565288
cg09505788	0.04354244	6.23427259	1.05447608	36.8582611
cg16158863	0.04355025	8.27752313	1.06309491	64.4508675
cg15080939	0.04355405	30.519554	1.10395295	843.734485
cg23341126	0.04356825	2928254.66	1.53695858	5.579E+12
cg10245865	0.04358421	2236136.26	1.52339764	3.2823E+12
cg18599117	0.04358664	9213702.9	1.58650036	5.3509E+13
cg18675600	0.04359149	0.10710326	0.01223225	0.93777569
cg18840832	0.04360771	308.473152	1.17868729	80730.2205
cg15387598	0.04360785	546.331446	1.19816659	249112.312
cg08886727	0.0436359	5.7602993	1.05126677	31.5629192
cg13105327	0.04366056	12.2715326	1.07390836	140.226594

cg18133072	0.02220267	36.380819	1.6716014	791.794017
cg10002561	0.0222058	1.1643E+11	38.1695301	3.55E+20
cg16525330	0.02221264	44.9485591	1.72248852	1172.93842
cg17513770	0.02221468	0.09578426	0.01282737	0.71523799
cg06457011	0.02221593	5.36209862	1.27115687	22.6188461
cg18142162	0.02222171	13.3384661	1.44777387	122.888443
cg03058232	0.02222516	7.84204921	1.34194	45.8274853
cg26505822	0.02223062	4448257411	23.8514892	8.2959E+17
cg21879102	0.02223136	4.54215667	1.24118686	16.6221444
cg00656881	0.0222384	15.4706702	1.47833293	161.899685
cg21401879	0.02224139	3.64721212	1.20280406	11.0592878
cg06905367	0.02224883	0.0812646	0.00944743	0.69901906
cg19290577	0.02225065	18.0399684	1.51076553	215.414274
cg05901193	0.02226076	51.842473	1.75582822	1530.69758
cg11739758	0.02227906	5.0231748	1.25853173	20.048986
cg19564884	0.02228469	3144.15839	3.14877881	3139544.75
cg00576402	0.02228575	2.1544E+19	566.857894	8.19E+35
cg02939508	0.02228871	4964570759	24.02781	1.0258E+18
cg20276962	0.02229392	14.2022086	1.45901424	138.245894
cg11629443	0.02230233	5536772.21	9.11309928	3.3639E+12
cg01492656	0.02230327	29.2647974	1.6168659	529.684228
cg05868023	0.02230562	25.6272687	1.58653524	413.956706
cg02627110	0.02230564	5272924289	24.1761398	1.15E+18
cg04380955	0.02230582	7.216195	1.32474074	39.3084238
cg07903860	0.02230716	12887932.4	10.2722465	1.617E+13
cg19867816	0.02231593	15554.8766	3.9463918	61310229.1
cg00736406	0.02233472	9.9543434	1.38619777	71.4825506
cg00014118	0.0223463	8.86008524	1.36322619	57.584802
cg24838316	0.02235106	4.00412033	1.21774919	13.166077
cg06897702	0.02235158	0.0093966	0.00017131	0.51542137
cg05445326	0.04367329	0.20116119	0.04235006	0.95550814
cg24330818	0.04367808	55.4778811	1.12062415	2746.50096
cg05502525	0.043679	506596739	1.76526969	1.4538E+17
cg23771949	0.04368112	0.11336774	0.01367032	0.94015693
cg13879483	0.04368362	3.81398657	1.03865652	14.0051049
cg08402850	0.04369354	17.1624844	1.08373297	271.792848
cg18604823	0.04369875	8.42204057	1.062074	66.7851462
cg15141467	0.04369886	7.2401066	1.05754362	49.5668856
cg19977011	0.04370567	2334825.08	1.51279718	3.6035E+12
cg01366985	0.04371907	5.69003766	1.050197	30.8290051
cg01292626	0.04373664	151.296145	1.15140099	19880.5835
cg12415590	0.04374701	4882.32137	1.26890461	18785542.9
cg08081524	0.04375107	93.5013846	1.13559846	7698.59176
cg04204002	0.04381523	858047.75	1.46052291	5.041E+11
cg21647780	0.04382931	175104.951	1.39646979	2.1957E+10
cg19516235	0.04383366	115.006158	1.14014219	11600.6727
cg23162960	0.04386472	89941150.9	1.65466939	4.8888E+15
cg01535980	0.04387604	11.2296561	1.06862979	118.006419
cg03443162	0.04390384	961.795263	1.20639238	766790.425
cg17169566	0.04392357	5624584.02	1.5267855	2.0721E+13
cg02125271	0.04393299	13.7620396	1.07387167	176.365332
cg14400118	0.04393576	8.71563185	1.06059065	71.62258
cg13394083	0.0439435	123664898	1.65796999	9.2239E+15
cg19838156	0.04394969	65.0745232	1.11983437	3781.53561
cg16093048	0.0439592	81.4571806	1.1264538	5890.40784
cg25461508	0.0439629	6.66000663	1.05261791	42.1384511
cg04948483	0.04396504	883.072566	1.20128221	649154.003
cg16525330	0.04396565	54.6693008	1.11423048	2682.32874
cg07921092	0.04397101	3745.61299	1.24885508	11233982.9
cg10508349	0.04397373	6.33012529	1.05107599	38.1233008

cg10999157	0.02235571	691.72799	2.53043413	189093.091
cg10056854	0.02235592	22561.446	4.15007759	122652851
cg10062803	0.02235796	9.94681683	1.38557533	71.4065579
cg18725076	0.02235807	9.13306901	1.36888656	60.9348882
cg02189843	0.02235912	11.550552	1.41526588	94.2686826
cg22960067	0.02236705	415331454	16.7092515	1.0324E+16
cg09619624	0.02237481	3.53828875	1.19631893	10.4650081
cg09820519	0.0223781	13.2597648	1.44281089	121.860295
cg24371075	0.02238629	21.6137572	1.54609143	302.151924
cg10638827	0.02238877	14.9439319	1.46721746	152.207227
cg01926269	0.02239258	9.459959	1.37505542	65.0816125
cg27640078	0.02239686	60.8291748	1.78989274	2067.2683
cg00375581	0.02240209	6.9319141	1.31561696	36.523878
cg19598293	0.02241334	9.30514616	1.37143764	63.1350214
cg26387796	0.02241436	6.33890345	1.29886491	30.9360092
cg08645907	0.02242204	6.68535954	1.30856706	34.1549423
cg05365729	0.02243771	44.9851516	1.71326968	1181.17065
cg16967099	0.02243821	8.90676031	1.36249304	58.224429
cg14015706	0.02244147	3.31058112	1.18447985	9.25296225
cg07627556	0.02244252	6.58886208	1.30555477	33.2526099
cg25509822	0.02244579	408063386	16.5020767	1.0091E+16
cg19410789	0.02244654	612672.807	6.58073009	5.704E+10
cg25984973	0.02244914	95.4864945	1.90509732	4785.93432
cg23271269	0.02245486	1069.22318	2.67996084	426587.655
cg04874562	0.0224578	0.08631435	0.01053223	0.70736885
cg13959152	0.02246562	1.8559E+10	28.2262893	1.2203E+19
cg07956775	0.02246719	29.3607769	1.61190242	534.806083
cg03635685	0.02246786	23.5063124	1.5620304	353.736216
cg11942971	0.02247001	10109.9558	3.67827517	27787808.8
cg02325250	0.02247216	8.12926348	1.34439277	49.1559657

cg15720535	0.04397388	8.83773265	1.06058583	73.6437508
cg13694725	0.04397601	100.137293	1.13236145	8855.36803
cg25860425	0.04399445	969203424	1.74474186	5.3839E+17
cg24874612	0.04399904	5.29884355	1.04583917	26.8470944
cg25065723	0.04401353	0.12507692	0.01654096	0.9457878
cg05876083	0.04404367	2.57E-10	1.19E-19	0.55490277
cg26592281	0.04404423	10974.0413	1.28161866	93966783.3
cg16400971	0.04405164	75411.1604	1.34870687	4216515278
cg00201025	0.04405916	13.8870346	1.07249566	179.813995
cg07574241	0.04407123	1079.42449	1.20370512	967975.634
cg25332377	0.04407271	13.7143042	1.07196357	175.45572
cg21016004	0.04408283	39.4007881	1.1022235	1408.44584
cg09339907	0.0441369	10912.7321	1.27635959	93302642.7
cg12968732	0.04413978	0.2222304	0.05137372	0.9613154
cg06374165	0.044145	4.17378007	1.03815527	16.7801875
cg24791601	0.04415195	1783480441	1.74644092	1.8213E+18
cg02322879	0.04415975	0.18098101	0.03425082	0.95630186
cg25308662	0.04416341	0.0405293	0.0017861	0.91966864
cg20939662	0.04417124	6.99353212	1.05204722	46.4898253
cg14653977	0.04420122	29.4578842	1.09175206	794.838841
cg14277108	0.04420235	619.070332	1.18148397	324378.565
cg14230666	0.04421695	4.05055528	1.03686061	15.8237259
cg04897244	0.0442187	15281.2254	1.28303355	182002916
cg20351640	0.04422173	125.531018	1.13308473	13907.2005
cg13621317	0.04422375	5.87215477	1.04681472	32.9401191
cg09125316	0.04423153	0.15251204	0.02441669	0.9526238
cg16701456	0.04424234	5.46176857	1.04470498	28.5543924
cg18949300	0.04424724	6.04625687	1.04740216	34.9027562
cg00869941	0.04424852	19.1106049	1.07887017	338.516378
cg01741932	0.04427751	87780968.8	1.5971244	4.8246E+15

cg24773720	0.02248587	4.92822018	1.25247136	19.3915446
cg05873146	0.02248596	12.3387693	1.425686	106.787349
cg02900441	0.02249132	10.6652991	1.39654405	81.4500661
cg07566050	0.022504	817.0868	2.57458609	259315.795
cg13042536	0.02250954	222143054	15.0246017	3.2844E+15
cg03314977	0.02251116	7.08575626	1.31791777	38.0964146
cg26841114	0.02251268	25.7900636	1.58115466	420.659278
cg07254054	0.02251656	9.52215292	1.37388192	65.9964986
cg23107161	0.02252907	35.8691788	1.6558037	777.02326
cg14037393	0.02253293	1382.79461	2.76918332	690499.951
cg10660256	0.02253485	9.63374048	1.37577669	67.459317
cg06785999	0.02253563	4.04243544	1.2173925	13.4231847
cg07065111	0.0225374	4.83764622	1.24855017	18.7439973
cg10397527	0.02253907	4.2186095	1.22468909	14.5315789
cg01254948	0.02254084	8.76260802	1.35742306	56.5654892
cg17330672	0.022543	4.45160645	1.23394847	16.0596658
cg04745805	0.02254362	6.02243114	1.28757283	28.1690294
cg00976340	0.02254373	70.7061454	1.82118747	2745.10949
cg16926389	0.02254511	3742702.02	8.41924615	1.6638E+12
cg17407629	0.02255983	27.7928303	1.59631866	483.889237
cg04911307	0.02256407	6645.96462	3.44842124	12808425.3
cg02378501	0.02256415	95029.6335	5.01307413	1801415859
cg19321739	0.02257413	1.3325E+11	36.6357779	4.85E+20
cg00735923	0.02257621	3.34390458	1.18493005	9.43658899
cg16269716	0.02257682	31.9326848	1.62719581	626.65867
cg15642759	0.02258012	6.25402475	1.29387954	30.229109
cg04396550	0.0225839	226.899849	2.14307302	24023.2324
cg02838877	0.02259988	9.56856076	1.37317804	66.6755166
cg20466166	0.02260156	14.0729351	1.44957169	136.624841
cg04123157	0.02260369	2670.70543	3.02740895	2356030.4

cg02186542	0.04430206	8.69667071	1.05667191	71.5757473
cg04981056	0.04430991	8.71096078	1.05663375	71.8137552
cg09020665	0.04431595	28.7498506	1.08913446	758.908969
cg15991262	0.04433385	14.4922071	1.07009681	196.266419
cg23737062	0.04435481	10.9111437	1.06218396	112.083274
cg26912671	0.04437634	17.6702666	1.07488481	290.48538
cg03609847	0.04437779	2.536E+14	2.30195449	2.79E+28
cg12798700	0.04438844	2037.63263	1.21066598	3429473.35
cg11915525	0.04439144	16.3343631	1.07255477	248.762511
cg10094907	0.04439466	799.69622	1.18236126	540878.72
cg09250087	0.04440791	6.72887572	1.0488147	43.1704175
cg10936964	0.04441478	19.9273545	1.07757043	368.51369
cg24041078	0.04444919	5.08242378	1.04116213	24.809807
cg00076353	0.0444512	61245.6729	1.3144022	2853793488
cg19896655	0.04445765	443847987	1.63761445	1.203E+17
cg21338852	0.04446474	50.4430094	1.10186128	2309.27182
cg14161426	0.04447353	3.7369E+10	1.82446541	7.65E+20
cg03072286	0.04447881	14.8280912	1.06880107	205.718628
cg26455413	0.04448654	18852.6261	1.27451457	278868144
cg04822330	0.04449201	5.12795851	1.0410586	25.258865
cg12406391	0.04449276	3.78217725	1.03328239	13.8441001
cg27345592	0.04449894	33515.1334	1.29194592	869435897
cg02884024	0.04450167	10768.3901	1.25623939	92305834.7
cg06969125	0.04450843	6517891.77	1.46965629	2.8907E+13
cg08511084	0.04452318	122254309	1.57729211	9.4758E+15
cg16509355	0.04459076	9.70346963	1.05644475	89.1265948
cg03094905	0.04459363	2118.65245	1.20315828	3730754.51
cg19751990	0.04459844	0.18825614	0.03689749	0.96050913
cg01208515	0.04462077	84.6906486	1.11254321	6446.94594
cg05724197	0.04462326	6.81460207	1.04716277	44.3472616

cg11649846	0.02260466	18567.5027	3.97441262	86742919.9
cg25800170	0.02261425	14.5185636	1.45561631	144.810611
cg01136750	0.02261703	8207506.94	9.33500192	7.2162E+12
cg18755581	0.02261737	10190.1287	3.65063325	28444030.6
cg15847400	0.02261752	539.781952	2.41736199	120529.965
cg03234661	0.02262714	661.876449	2.48653391	176181.162
cg10382221	0.02263796	6.59958832	1.30279235	33.4317024
cg23684246	0.02264167	13.6035184	1.4417238	128.357258
cg24810594	0.022659	1.8572E+10	27.4193816	1.2579E+19
cg06890747	0.02265967	7.06788481	1.31501727	37.988091
cg01102833	0.02266196	20.4943423	1.52636184	275.175948
cg10160068	0.02266642	4.7039E+10	31.1943385	7.0932E+19
cg14633527	0.02267704	32717725.6	11.2593853	9.5072E+13
cg01718071	0.02268275	119532.704	5.13203133	2784095896
cg03290610	0.02268628	17.9916686	1.49811014	216.072324
cg04674143	0.0226869	9978.19542	3.62511422	27465171.5
cg03453744	0.02269048	23.0228293	1.55054893	341.847111
cg17603132	0.0227061	5.28298075	1.26187526	22.1177849
cg11521979	0.02270638	8.66737794	1.35226079	55.5539589
cg08602346	0.02270808	0.19868374	0.04947569	0.79787111
cg01066472	0.02271623	6.77035856	1.30622436	35.0917931
cg20384898	0.02271649	3.81321067	1.2055697	12.0611654
cg21851967	0.02271783	4353.48276	3.22275248	5880939.42
cg01516119	0.02271928	1319.23773	2.72758799	638068.579
cg18152871	0.02272067	6.25E-18	9.88E-33	0.00395801
cg26951614	0.02272274	25.0503492	1.56792444	400.223365
cg14334298	0.02274075	4.80604746	1.24487147	18.5546
cg03511487	0.02274768	9886140687	24.7792817	3.9443E+18
cg00547414	0.02274799	33.6351324	1.63286913	692.843112
cg05721376	0.02274857	8.67045111	1.35154681	55.6227295

cg15416643	0.04462859	6.4319572	1.04566319	39.5634786
cg24805759	0.04463292	11.7717399	1.06088374	130.621156
cg17601287	0.04463685	15.425272	1.06772721	222.846263
cg24371075	0.04465012	65.0834047	1.10490717	3833.67008
cg13762471	0.04469394	27129.5722	1.27359467	577902609
cg04059461	0.04469874	16.2951543	1.06828688	248.558753
cg06799455	0.0447128	3389.54661	1.21151018	9483227.15
cg22251932	0.04473478	1761.02692	1.19203358	2601617.82
cg03830181	0.04474526	9.70832506	1.05476218	89.3581294
cg12828075	0.04476505	5.18617784	1.03920898	25.8816476
cg20141824	0.04477019	66761.952	1.29603012	3439085362
cg14579118	0.04477269	10.0610105	1.05534004	95.9159402
cg00381622	0.04477898	44.0084809	1.0921889	1773.27053
cg07489950	0.04478133	10935.7217	1.24185223	96299710.2
cg15169829	0.04478393	7.52295575	1.04809785	53.9976902
cg25446604	0.04481011	0.17100716	0.03046435	0.95992372
cg16044565	0.04487028	7.8386E+10	1.77547996	3.46E+21
cg07914457	0.04487037	12.4236652	1.05935513	145.699446
cg07270460	0.04487131	2.1365E+13	2.01831641	2.26E+26
cg10815165	0.04487603	18.8528604	1.06943322	332.353939
cg11572305	0.04487654	2121.99663	1.19134597	3779649.1
cg02611419	0.04490754	0.16741335	0.0291885	0.9602147
cg22714033	0.04492696	5145.37855	1.21332247	21820184.8
cg01425188	0.04492793	13.8112737	1.06119305	179.751725
cg19657320	0.04495248	4802844.22	1.4138421	1.6315E+13
cg13679837	0.04496977	723904020	1.58027709	3.3161E+17
cg17822282	0.04497879	4.68E-08	3.19E-15	0.68530126
cg03675258	0.04499109	6.33154819	1.04207966	38.46971
cg05118960	0.04501972	8.39536506	1.04837556	67.2298716
cg26008908	0.0450348	7.35655186	1.04516197	51.7803523

cg09920522	0.02274891	138168369	13.6567197	1.3979E+15
cg25446197	0.0227557	1289664.85	7.11160692	2.3388E+11
cg05714559	0.02276216	62.1610313	1.77824721	2172.92274
cg25490477	0.02279222	11.4076244	1.40332261	92.7326998
cg21624854	0.0227934	2292841.83	7.67877918	6.8463E+11
cg21041329	0.02280398	276104.046	5.71441093	1.3341E+10
cg26430382	0.02280527	23869999.5	10.6257394	5.3622E+13
cg04990724	0.02280626	6.9133E+11	44.3594427	1.08E+22
cg22307331	0.02280709	5.91053838	1.28036831	27.2846991
cg03431903	0.02281045	28587.542	4.16647227	196148565
cg00983948	0.02281398	5.08292558	1.25369272	20.6080262
cg08639762	0.02281869	9.40666691	1.36563766	64.7941875
cg02303873	0.02282791	171388.34	5.33787006	5502937072
cg09478396	0.02282923	5.40818558	1.26434704	23.1332619
cg24668061	0.02283497	8.46260015	1.34540829	53.229642
cg20975074	0.02284166	6.92951239	1.30845763	36.6982782
cg19628553	0.02284771	12.1628016	1.4146586	104.572044
cg23514016	0.02285303	6.40965014	1.29418964	31.7446638
cg07948472	0.02285324	280.627054	2.18688166	36010.8849
cg10869957	0.02287355	0.07267222	0.00759704	0.69517233
cg01589129	0.02287839	4.19003024	1.21975105	14.3933907
cg11674713	0.02287858	1.8259E+10	26.469945	1.2594E+19
cg26061304	0.02288481	1812.48286	2.82899157	1161224.42
cg01747518	0.02289106	13.6020416	1.43577012	128.861531
cg03450370	0.02289757	15.8833903	1.46678634	171.996479
cg22351880	0.02289897	21426044.1	10.3631518	4.4299E+13
cg02208899	0.02289953	12133312.3	9.57760839	1.5371E+13
cg17117049	0.02290186	10.8427002	1.39113643	84.5094302
cg21014483	0.02290667	4.60459753	1.23547489	17.1612703
cg22548015	0.02291028	3.2959E+10	28.5899344	3.7996E+19

cg16905506	0.0450524	7.4612109	1.04532031	53.2560857
cg17371249	0.0450644	1.3413E+11	1.75712897	1.02E+22
cg06763124	0.04508382	0.03933668	0.00166106	0.93155837
cg10549973	0.04508646	59.6876771	1.09368119	3257.4564
cg06443918	0.04509229	667182.878	1.34088776	3.3197E+11
cg16191009	0.04511608	22.9800529	1.07060446	493.256706
cg07951301	0.04511763	10.7404356	1.05300967	109.549759
cg05153861	0.04512771	172910517	1.5095774	1.9806E+16
cg07125725	0.04515079	10.9052625	1.05297868	112.941272
cg19782271	0.04515203	11.0669664	1.05329931	116.280096
cg02670343	0.04517045	33.5865388	1.07854513	1045.90486
cg26694487	0.0451862	16096.3175	1.23087545	210493628
cg06104975	0.04518623	12.1186522	1.05495704	139.211101
cg20425447	0.04521051	5329504721	1.61255626	1.7614E+19
cg22043720	0.04521393	1613.25278	1.17053707	2223410.61
cg15445332	0.04522911	6701069971	1.61735474	2.78E+19
cg01185682	0.0452312	4.8210457	1.03397552	22.4787544
cg04678743	0.04525481	13.8902758	1.05718009	182.504158
cg25206802	0.04527052	0.00059896	4.19E-07	0.8553164
cg04326808	0.0452719	7.35985635	1.042923	51.9381443
cg13821072	0.04527242	197.423061	1.11769938	34871.5099
cg25930229	0.04529318	8.53000862	1.04595109	69.5644834
cg17927777	0.04529711	26.5063501	1.07104069	655.984972
cg21045608	0.04530048	10.3211775	1.05005654	101.448543
cg20446334	0.04530231	3.39701068	1.02590938	11.2482465
cg00917031	0.04530518	25.6081869	1.07014047	612.797339
cg12959887	0.04531176	29315753.1	1.43175646	6.0025E+14
cg14360865	0.04532479	9.64007387	1.04829502	88.6496862
cg04897804	0.04536709	11.6748429	1.05198514	129.566429
cg26243475	0.04536972	1.5446E+14	1.9608408	1.22E+28

cg04920683	0.02291521	264582.147	5.63098281	1.2432E+10
cg24008238	0.02292022	4.18538558	1.21910249	14.3691384
cg09245319	0.02292114	18.719618	1.49989958	233.631705
cg01803238	0.02292614	23.4923831	1.54763135	356.604343
cg03786924	0.02292966	394.302375	2.28599267	68011.7503
cg11424962	0.02294032	102.820931	1.89754101	5571.49694
cg02851730	0.02294809	826.952004	2.5306314	270228.852
cg05916700	0.02294813	11.9894445	1.40961976	101.975571
cg08753297	0.02295736	9.63070904	1.36739892	67.8299178
cg17822282	0.02296076	2.33E-07	4.49E-13	0.12131471
cg18342621	0.02296106	814.519135	2.52395473	262857.892
cg08165784	0.02296228	10.1993662	1.37818016	75.4814741
cg00036588	0.0229696	270.301413	2.16660214	33722.3215
cg17641346	0.02297179	97434.3817	4.88372475	1943897173
cg11457534	0.02297237	0.11475875	0.01775726	0.74164431
cg19024632	0.02297317	8.3865738	1.34123178	52.4403171
cg01283289	0.02298302	10.2188413	1.37812462	75.7730577
cg13470499	0.02298444	14342.0339	3.74574505	54914024.8
cg21702506	0.02298844	19.9438619	1.51118548	263.209005
cg01244303	0.02299088	4862.84985	3.22534211	7331721.05
cg05311410	0.02299744	3.46429685	1.18689714	10.1115356
cg15426734	0.02300049	5.31345904	1.2589713	22.42533
cg21552528	0.02300365	1611.90461	2.76800922	938666.121
cg01731685	0.02300722	176.692313	2.04057977	15299.6585
cg12184886	0.02301228	4.09017454	1.2142335	13.7778506
cg21771463	0.02301488	8.21600244	1.33669248	50.4997949
cg21557473	0.02301759	11.3772388	1.39795688	92.5933874
cg00506625	0.02302153	14879.6973	3.75637609	58941220.8
cg05803296	0.02302184	5.52308522	1.26541515	24.1062945
cg24096828	0.02302287	75.7114877	1.81483035	3158.54833

cg18882819	0.04540737	8.24837436	1.04407171	65.1637995
cg02045669	0.04542525	7.04727305	1.04055359	47.7284959
cg04691829	0.04543023	12.2365112	1.05224891	142.297325
cg02886027	0.04543851	772.285147	1.14450327	521120.705
cg02801839	0.04543912	26395.5612	1.22952503	566662438
cg02329038	0.04544791	5.15455836	1.03377464	25.7014159
cg11555438	0.04548573	9.12906897	1.04541605	79.7193618
cg03877492	0.04550113	128.643926	1.10209115	15016.235
cg21433558	0.04551341	4.95421341	1.03245361	23.7727199
cg19638477	0.04552915	4.55156124	1.03059684	20.1016624
cg00928397	0.04553495	9.31249692	1.04531278	82.9633012
cg22325530	0.04555842	4.8844827	1.03182741	23.12225
cg23693200	0.04555955	124.0313	1.09988002	13986.765
cg14658900	0.04559883	389569.719	1.28650962	1.1797E+11
cg19974445	0.0456012	2146955216	1.52241305	3.0277E+18
cg07640390	0.04560323	814775.945	1.3048681	5.0876E+11
cg07677850	0.04565526	0.00944467	9.76E-05	0.91388241
cg04527260	0.04565866	0.00098741	1.11E-06	0.87497253
cg10116014	0.04565883	0.09906524	0.01026172	0.95636197
cg03112433	0.0456862	4.99383037	1.03131769	24.1810472
cg24183484	0.04568927	78.3720727	1.08716189	5649.7398
cg15777760	0.04569494	109.159322	1.09395421	10892.3732
cg12436713	0.04569535	8475816.62	1.35694022	5.2942E+13
cg16493531	0.04569717	77196.3799	1.24015723	4805262521
cg11072113	0.04570343	11.5501318	1.04783321	127.315629
cg26293512	0.04571932	10.6348234	1.04600444	108.125229
cg05365729	0.04572297	40.1405302	1.07270844	1502.05043
cg14632140	0.04572547	6.89715952	1.03736678	45.8572708
cg08193467	0.0457319	14.9817177	1.05268417	213.218619
cg00014118	0.04574427	8.37324991	1.04100738	67.349488

cg19643467	0.02302832	0.10993832	0.01638089	0.73783735
cg16177440	0.02303428	388.786101	2.27259027	66512.0478
cg09828721	0.02303491	3.5651E+17	260.791601	4.87E+32
cg17639493	0.02303871	0.17301772	0.03811103	0.78547154
cg12501949	0.02306368	18.4289997	1.49274424	227.519237
cg19094597	0.02306978	207.795502	2.08231999	20735.9921
cg27289153	0.02307479	8.18131762	1.33485534	50.1432297
cg21490755	0.02307874	6.2357E+12	57.2627295	6.79E+23
cg10401088	0.02308226	6.21167459	1.28516928	30.0232053
cg26397881	0.02308365	11054749.8	9.27867732	1.3171E+13
cg26680675	0.02308387	7.18051597	1.31098727	39.3289934
cg04812556	0.02309403	6.64619706	1.29697966	34.0575391
cg17827670	0.02310335	99.8511521	1.88095789	5300.62509
cg25428429	0.02310482	6.19321318	1.28431168	29.8649386
cg02424695	0.02311081	1.6027E+11	34.4476668	7.46E+20
cg09323092	0.02311268	0.10007647	0.01373389	0.72923955
cg02861504	0.02312204	0.01732493	0.00052342	0.5734463
cg26392523	0.02313579	1.0204E+10	23.5236864	4.4265E+18
cg19811296	0.02313722	45890.9223	4.35266551	483836111
cg04110544	0.02314634	5.14889557	1.25163929	21.1811228
cg23487303	0.02314751	64277.5981	4.55507394	907034590
cg01505767	0.02314993	6.78324304	1.29974519	35.4010819
cg08036278	0.02315425	25965.3769	4.02155188	167646923
cg26286839	0.02315601	5.4274866	1.26057581	23.3683771
cg22345692	0.02315751	8.46833112	1.33970693	53.5285967
cg26607758	0.02315787	3.36298104	1.18060366	9.57954127
cg18720617	0.0231754	0.08013405	0.00906926	0.70804748
cg20009327	0.0231757	11.4581491	1.39593545	94.0510401
cg17665193	0.02318061	0.06176131	0.00558215	0.68333216
cg13205784	0.02318086	8640693123	22.8452865	3.2681E+18

cg17792849	0.04575271	45.6911516	1.07479926	1942.39186
cg11478273	0.04579561	653.187374	1.12871386	377999.918
cg25217313	0.0458164	118785.778	1.24256003	1.1356E+10
cg17639493	0.04583762	0.19107541	0.03764435	0.96986165
cg06976589	0.04584101	5.72181023	1.03274913	31.7009344
cg17214455	0.04587426	8.20233495	1.0393142	64.7333586
cg24034106	0.04588133	1.29E-08	2.32E-16	0.71727515
cg19270251	0.045889	38.5194415	1.0689319	1388.06539
cg01518659	0.04589959	1.70E-07	3.86E-14	0.75291936
cg12456825	0.04590257	8.96867956	1.04072425	77.2896501
cg17327184	0.04590789	21.9682821	1.05774948	456.256823
cg12058064	0.04591987	10.9366128	1.0442924	114.536406
cg03339560	0.04592849	17.6526582	1.05327228	295.855447
cg14677130	0.04594384	19.0436052	1.05450196	343.914863
cg00442646	0.04596084	18.998772	1.05421864	342.389446
cg03149565	0.04596207	0.25071682	0.06443736	0.97550433
cg10810752	0.04596983	5.26616773	1.03016996	26.920337
cg24646841	0.04597145	0.21588631	0.0479024	0.97295537
cg21107197	0.04598031	15.1008598	1.04963598	217.25243
cg18189263	0.04602187	0.11388857	0.01347783	0.96236641
cg16225168	0.04602781	42.1419159	1.06817807	1662.58897
cg07740599	0.04603612	0.05168196	0.00281393	0.94921485
cg14781281	0.04603679	5.91444562	1.0317572	33.9039717
cg13475699	0.04603787	84515.2875	1.22078113	5851035564
cg10500167	0.04604475	0.10769194	0.01206021	0.96163766
cg15395441	0.04604575	8.3209992	1.03788232	66.7118288
cg08462879	0.04606844	7.08187014	1.03474209	48.4689712
cg05153345	0.04607153	6.1486521	1.03216814	36.6276785
cg19116023	0.04608065	7.60099544	1.03590417	55.7726606
cg07238832	0.04608688	27.5061994	1.05923726	714.279075

cg23369753	0.02318864	38093147.9	10.871522	1.3348E+14
cg21413173	0.02319535	5.8794959	1.27389982	27.1359424
cg16440442	0.0231965	32.5893254	1.60975813	659.766277
cg17152135	0.02320021	383.658124	2.25441531	65291.2334
cg06767314	0.02320935	6.68845553	1.29632421	34.5094515
cg16897415	0.02321326	2.6721E+10	26.5292941	2.69E+19
cg02494572	0.02321768	11.1418363	1.38971852	89.3278126
cg05103992	0.02322674	198508.205	5.28363997	7458022846
cg02854695	0.02323268	34.0729495	1.61828532	717.404944
cg22083798	0.02323579	15.5205325	1.4535942	165.718141
cg08952506	0.0232414	7.7746803	1.32269724	45.6987825
cg15038664	0.02324353	11.1131765	1.38868823	88.9347864
cg04209460	0.02325118	189.931405	2.04450294	17644.3565
cg24676486	0.02325744	4.6542E+11	38.8997669	5.57E+21
cg08964780	0.02325827	4.62824479	1.23217124	17.3844748
cg07048210	0.02327173	22.3071836	1.52625953	326.03265
cg08242069	0.0232735	253473.207	5.44297252	1.1804E+10
cg00237825	0.02327741	0.15928826	0.03258248	0.77872373
cg08535411	0.02328595	23736.9969	3.93955293	143022579
cg16472904	0.02328605	13.213282	1.42087686	122.875407
cg05204389	0.02329097	724.133516	2.44962601	214060.982
cg14159026	0.02329217	3.34250744	1.17841983	9.48079429
cg23840481	0.02329439	30426.5872	4.07271685	227311951
cg25864727	0.02329705	0.19341266	0.04677585	0.79973865
cg13908395	0.02329711	2267.94741	2.86042183	1798191.23
cg04022108	0.02329716	3311.91791	3.01159675	3642187.56
cg08367083	0.0232982	6.97520085	1.30236897	37.3576369
cg02478520	0.02330915	5.7873E+12	54.322372	6.17E+23
cg19641924	0.02330979	7.06416126	1.30443093	38.2560497
cg19387936	0.02331473	9.29704964	1.35396263	63.8386393

cg26691604	0.04609062	187.294959	1.09501517	32035.5395
cg20058667	0.04609884	1030064.16	1.2708036	8.3493E+11
cg20310435	0.0461	1216169.26	1.27436891	1.1606E+12
cg12811135	0.04613928	0.02334695	0.00058131	0.93767633
cg13995101	0.04614015	493516.966	1.251659	1.9459E+11
cg00528600	0.04615154	5197738.57	1.3021148	2.0748E+13
cg00689580	0.04620576	4.18106912	1.02436428	17.0655492
cg13841742	0.04621682	59.6151456	1.07099	3318.39287
cg17345480	0.04622654	48.819441	1.06722449	2233.21132
cg13249789	0.04623384	833.996897	1.11888332	621647.327
cg01941047	0.0462396	1.227E+12	1.59065144	9.46E+23
cg01064131	0.04625099	0.03529673	0.00131707	0.94592954
cg20147819	0.04625292	20.1212089	1.05113748	385.1666
cg02969706	0.04625786	0.2133111	0.04668314	0.97469079
cg08444060	0.0462634	92125452.4	1.35502593	6.2634E+15
cg13484341	0.0462758	17.3698529	1.04826411	287.820395
cg18632254	0.04629369	9885020.73	1.30296513	7.4993E+13
cg24797508	0.04630835	12.4860167	1.04217987	149.59089
cg02831290	0.04631785	470.437361	1.10565688	200162.741
cg24405664	0.04632302	612648.353	1.2425783	3.0206E+11
cg10277546	0.04633618	2.3698E+10	1.47393759	3.81E+20
cg04114121	0.04634442	1264.90627	1.12269139	1425135.95
cg05768041	0.04636889	1732.28224	1.12749881	2661467.78
cg23816347	0.04638275	6.03829482	1.02924281	35.4250755
cg25692259	0.04638392	7584975.27	1.2889839	4.4633E+13
cg09539395	0.04646198	0.00062031	4.32E-07	0.89069684
cg20950465	0.04646867	4.13225957	1.02244293	16.7007552
cg16841327	0.04647352	4955.18338	1.1421459	21497991.1
cg14283602	0.04649507	33820095.5	1.30883709	8.739E+14
cg02205221	0.04649755	18083.3214	1.16424413	280874519

cg03224126	0.02331546	14.5995503	1.43958973	148.060842
cg04945388	0.02331637	49.4276004	1.69905771	1437.90742
cg06958211	0.02333236	30.8377499	1.59299853	596.966539
cg04468971	0.02333237	1.3975E+10	23.8630454	8.1847E+18
cg20776123	0.02333564	125753.614	4.92496902	3210978877
cg24902075	0.02333601	8165226073	22.1720433	3.007E+18
cg14658651	0.02333635	3869254.61	7.8419969	1.9091E+12
cg00701946	0.02333831	6.399198	1.28659178	31.8280714
cg02699703	0.02333996	116.910497	1.90859345	7161.32829
cg20461826	0.02335442	30.169037	1.58751737	573.329658
cg02913948	0.02336267	15449662	9.43807838	2.529E+13
cg27477990	0.02336511	445362.153	5.83353018	3.4001E+10
cg01592312	0.02336751	5180117.3	8.13435689	3.2988E+12
cg02571738	0.02336822	11.607404	1.39428323	96.631606
cg22986077	0.02336945	10.3712629	1.37313468	78.3339731
cg09750385	0.02336956	598.112389	2.37923834	150358.383
cg21589273	0.02337365	115833.278	4.85685509	2762558894
cg19937878	0.02337423	10618.8057	3.51303321	32097343.9
cg24294159	0.02337543	21.3798849	1.51445034	301.825333
cg13388731	0.02338325	17.1228115	1.46935121	199.537506
cg01027532	0.02339279	11.8746504	1.39806188	100.859142
cg00215224	0.02339787	27.6648417	1.56755164	488.241311
cg16926284	0.02339869	15065286.1	9.37098691	2.422E+13
cg24865406	0.02340307	17045743.2	9.52466205	3.0506E+13
cg21252609	0.02341164	10742971.9	8.93996669	1.291E+13
cg04015486	0.023416	88015336.1	11.8770996	6.5224E+14
cg05967404	0.02341966	7.95852114	1.32385584	47.8436223
cg06537680	0.02342751	2978256165	19.0951114	4.6452E+17
cg25212453	0.02342869	32.9594609	1.60408203	677.226003
cg03153397	0.02342947	2316.54911	2.85036552	1882705.84

cg09961689	0.04652112	26.0226871	1.05149374	644.017379
cg01535453	0.04652878	23.6848121	1.04985536	534.331053
cg24884519	0.04653896	3.99638447	1.02146098	15.6355349
cg25355076	0.04655356	1.8239E+11	1.48545376	2.24E+22
cg22076311	0.0465593	24.9809508	1.0502519	594.188788
cg18018798	0.04657311	2181450.81	1.24790473	3.8134E+12
cg08775774	0.04658836	95.8176288	1.07134624	8569.60865
cg23047335	0.04660449	212.16375	1.08386662	41530.439
cg06965373	0.04661588	6.52846663	1.02850647	41.4395805
cg10825332	0.04661896	68672736.2	1.31007991	3.5997E+15
cg03839625	0.04667273	821.851451	1.10388428	611875.555
cg00034101	0.04670531	14.7045377	1.03997111	207.912919
cg09551160	0.04671539	20720.5673	1.15541307	371591703
cg00202389	0.04671714	0.04852336	0.00246031	0.95699817
cg12361311	0.04673755	85017521.8	1.30155905	5.5533E+15
cg23483563	0.04674192	7.59541423	1.02965955	56.0285362
cg13053408	0.04674271	43.0268615	1.05571212	1753.61331
cg14312538	0.04674274	4.39779989	1.02157493	18.9321833
cg08087125	0.04674556	5.4208E+10	1.42745459	2.06E+21
cg17083504	0.04677707	9044.38313	1.13870055	71837030.7
cg01486387	0.04682181	6068.46616	1.13026349	32582032.3
cg06904356	0.04682874	0.15918082	0.02600015	0.97455323
cg25682080	0.04684622	10.6516974	1.03354701	109.776
cg09920051	0.04685358	3.1977E+12	1.4928029	6.85E+24
cg07757611	0.04685608	6.60564305	1.02659654	42.5040593
cg22584335	0.04689715	88437.6938	1.16913784	6689737867
cg08711724	0.04690979	134380469	1.29138816	1.3983E+16
cg02774160	0.04694518	6.45622247	1.02550612	40.6460846
cg25533361	0.04694976	9026.98051	1.1306707	72069062.1
cg19478371	0.04697066	475.803778	1.08605437	208451.107

cg16233311	0.02343076	17.3123837	1.47030112	203.848468	cg03131236	0.04697202	0.00034908	1.36E-07	0.8989381
cg24183484	0.02344029	74.2011696	1.78952949	3076.68222	cg00636737	0.04697659	11.1633361	1.03276717	120.666184
cg14099685	0.02344219	55.8877849	1.72220609	1813.62992	cg05569109	0.04697814	1334.13227	1.10088675	1616795.66
cg26164440	0.02344294	1.7954E+11	33.153594	9.72E+20	cg10224098	0.04700888	3.48593116	1.01664427	11.9527709
cg17386181	0.02345101	71.3734852	1.77965096	2862.45702	cg11314310	0.04701093	3.16227645	1.0153248	9.8490575
cg04953552	0.02345433	8.35782433	1.33203891	52.4408313	cg04657044	0.04701169	13.2806394	1.03474635	170.45277
cg11647690	0.02345692	7.56486451	1.31418552	43.5457354	cg20092936	0.0470213	4.14267342	1.0188861	16.8436326
cg26842775	0.02346665	6.73757991	1.2936454	35.0907468	cg03616995	0.04702331	2.5335E+11	1.41257428	4.54E+22
cg06193043	0.023467	7.33557229	1.30857151	41.1216509	cg04254119	0.04703322	0.15141433	0.02350077	0.97555509
cg07357081	0.02347714	24.7250461	1.54144244	396.594702	cg04832210	0.04703398	2053.13107	1.10513498	3814327.9
cg11306587	0.02347759	9.06453115	1.34628969	61.0312367	cg18813989	0.04703876	289822.23	1.17889315	7.1251E+10
cg26608305	0.02348191	0.14761059	0.02820281	0.77257872	cg11993754	0.04707022	0.01013482	0.00010901	0.94229293
cg14558573	0.02348828	105.695969	1.87454124	5959.66497	cg16101252	0.04707643	56238.0659	1.15174344	2746028290
cg15624376	0.02349493	4.86088759	1.23753779	19.0929346	cg01650726	0.0470885	29.5971082	1.0445387	838.63701
cg10407113	0.02349675	57.4336697	1.72618793	1910.93123	cg24889708	0.04709554	5.64088751	1.0224471	31.121035
cg00047287	0.02350002	20.368171	1.50101356	276.38817	cg24441975	0.04711102	21421.5134	1.13571951	404044510
cg20254282	0.02350088	332033293	14.0671211	7.8371E+15	cg08454455	0.04712182	4.8119634	1.02017566	22.6970635
cg16748413	0.0235028	6.74986051	1.29340709	35.2252723	cg13458803	0.04713588	6.17114484	1.02329045	37.2162455
cg11413751	0.02350324	52218240.3	10.9607773	2.4877E+14	cg18613421	0.04714039	13.705546	1.0336178	181.732542
cg11044163	0.02350395	7.58016259	1.31376411	43.7360593	cg23346960	0.04717239	5593.37653	1.11378219	28089747.9
cg01044129	0.02350484	7.9874615	1.3230456	48.2217251	cg03935183	0.04718789	14.3037355	1.03359155	197.947486
cg03845289	0.02350831	0.00020124	1.27E-07	0.31776631	cg22375689	0.04719923	0.15891303	0.02583443	0.97750744
cg11920449	0.02351827	8.58281676	1.33568148	55.1514298	cg06649737	0.04719964	7.37986926	1.02502463	53.1328408
cg15681626	0.02352908	4.26159548	1.21540931	14.9424526	cg12943155	0.04721245	9.9796876	1.02872171	96.813515
cg22484594	0.0235354	0.06090489	0.00540515	0.68627218	cg07915528	0.04722337	71.8393193	1.05380195	4897.39823
cg14214182	0.02353792	14.9902096	1.43934775	156.116814	cg01999333	0.0472245	0.13011033	0.01735712	0.97531733
cg02614549	0.02354523	0.07360844	0.00769521	0.70410018	cg08850949	0.04723845	188.644311	1.06597263	33384.2304
cg03181248	0.02354673	4.25710536	1.21504361	14.9154696	cg26875384	0.04724822	13.5289429	1.03215207	177.330746
cg15392111	0.02354805	135.944306	1.93570025	9547.37407	cg17597901	0.04725131	12.5197787	1.03114443	152.010576
cg12430467	0.02354839	552.746382	2.33741969	130711.897	cg07840972	0.04725692	2655.00586	1.1001851	6407154.71

cg14180029	0.02354877	8.15543848	1.32599935	50.159283
cg17735372	0.02355074	7.91352336	1.32060836	47.4204569
cg08067617	0.02355243	8.45655601	1.33241417	53.6720045
cg05816193	0.02355991	6.54550381	1.28720087	33.2843311
cg03677069	0.02356266	5.15994434	1.24667415	21.3568441
cg12074985	0.02356685	9.50321036	1.35320592	66.7385545
cg24165760	0.02357039	32.159504	1.59387316	648.880804
cg02400334	0.02358069	39.9795145	1.64077024	974.153205
cg00417576	0.02358169	16.2322495	1.45373944	181.247008
cg04337056	0.02358909	0.15657641	0.03144265	0.77971075
cg08700546	0.02359224	60.19724	1.73293199	2091.08478
cg05542338	0.02361227	4.17970304	1.21134346	14.4219357
cg16164732	0.0236142	231.620056	2.0748198	25856.6311
cg25076597	0.02361715	4.30351118	1.21603913	15.2299445
cg25193742	0.02362222	455622.68	5.73074983	3.6224E+10
cg13959647	0.02362331	10.9974521	1.37885427	87.7133694
cg14188840	0.02362355	13.0069994	1.41020516	119.969802
cg12544957	0.02362541	18.2981208	1.47614202	226.821824
cg17046776	0.02362775	7.91446583	1.31932258	47.4779788
cg00684824	0.02362901	14.6235203	1.43238312	149.294797
cg07447157	0.02363713	52.466651	1.6993874	1619.84811
cg01166827	0.02363914	649.582894	2.37999868	177293.349
cg20355694	0.02364325	13791.0099	3.58207605	53095454.1
cg09132763	0.02364601	25.9894408	1.54654781	436.747591
cg00051979	0.02364609	141.637953	1.94054338	10337.9856
cg02670545	0.02365043	0.07823407	0.00860746	0.71107765
cg22815056	0.02366336	2719.9553	2.87957768	2569181.2
cg20655350	0.02366413	7.58906812	1.31132328	43.9204853
cg24798540	0.02366431	11.7377396	1.39007103	99.1133021
cg27533013	0.02366655	22140589.3	9.59797996	5.1074E+13

cg06236061	0.04727027	27.6908486	1.04083254	736.701697
cg06439982	0.04727558	3.0092E+10	1.33666273	6.77E+20
cg20066782	0.0472948	21.906419	1.03754673	462.524896
cg13233166	0.04732484	43.6760192	1.0456011	1824.40001
cg13696236	0.04733804	9.92393368	1.02732755	95.8647117
cg14219044	0.04736409	267483.166	1.15645397	6.1868E+10
cg10758227	0.04738887	36.6509964	1.04236528	1288.69943
cg01045118	0.04742638	8.38582074	1.02443814	68.644447
cg00777652	0.04743006	669306.625	1.16425233	3.8477E+11
cg00579105	0.04743408	193.320718	1.06140071	35210.924
cg23089445	0.04743901	18.21958	1.03333436	321.244609
cg16007090	0.04744426	30533.8998	1.12347035	829856377
cg21583565	0.04745392	387.738784	1.06923167	140606.914
cg00662646	0.04746013	18251.7603	1.11619608	298448239
cg00013475	0.04746018	13.8767045	1.02990486	186.971568
cg09459581	0.04746897	8.79525642	1.02456953	75.5014988
cg02254152	0.04747608	312.809788	1.06605057	91787.3559
cg16792202	0.04747679	1198.29449	1.08208492	1326984.29
cg01573194	0.04750812	8.7233573	1.0240885	74.3070181
cg00445566	0.04750871	4906092.9	1.1844294	2.0322E+13
cg21267231	0.04751712	23.1275265	1.03499004	516.79964
cg20586124	0.04752734	338.156395	1.06555711	107314.518
cg05235884	0.04753882	0.18794343	0.03596938	0.98202232
cg26259537	0.04754299	4.27037566	1.01585202	17.9515401
cg20912226	0.04754586	156978.928	1.13821713	2.165E+10
cg20837456	0.0475599	0.00123328	1.63E-06	0.9304732
cg07213036	0.04758346	4.16480453	1.0153154	17.0839492
cg20778688	0.04758697	42.1100082	1.04059164	1704.08134
cg21878918	0.04761979	191.545313	1.0566846	34721.4362
cg26477573	0.04762107	4.64511423	1.01623524	21.2323736

cg01972009	0.02368054	14.3533325	1.42759278	144.311568
cg11847964	0.02368614	10.1951335	1.36369487	76.219945
cg26816421	0.02369492	25.1732117	1.53845024	411.901906
cg15755476	0.02370541	6.48439294	1.28341007	32.7622113
cg20140110	0.0237092	5.52834605	1.25632358	24.3270209
cg22770592	0.02370967	12549.1584	3.52338598	44696033.3
cg01408654	0.02371058	4.91387265	1.23670658	19.524554
cg17894755	0.02372904	6.97390359	1.29556936	37.5397357
cg10657141	0.02374745	11.8218081	1.38962818	100.570173
cg04761177	0.02374958	0.05335463	0.00420619	0.67679289
cg05819594	0.02375093	12.702901	1.40292272	115.019659
cg27588321	0.02375185	13.760403	1.41792369	133.539408
cg05126887	0.02375375	11.3552822	1.38206371	93.2970265
cg13455434	0.02375553	5.14767562	1.2438339	21.3039412
cg18877506	0.02375898	5.69195929	1.26054691	25.7018603
cg12200478	0.02376258	7.16007306	1.29959659	39.4481231
cg22092811	0.02376436	6.71459197	1.28850362	34.990779
cg12045833	0.02376592	32.0660713	1.58656218	648.088642
cg14519917	0.02376964	98.1744391	1.84116329	5234.85373
cg10316490	0.02377086	3.81056101	1.19484837	12.1524836
cg23771949	0.02377515	0.12407977	0.02032264	0.7575685
cg23176237	0.02377791	54487.3423	4.26635507	695879837
cg15702823	0.02377907	171798.571	4.97008011	5938485574
cg06838879	0.02378607	8.79707498	1.33528875	57.9563995
cg14621323	0.02379104	10.0826191	1.35963148	74.7696788
cg20588892	0.02379151	2035034174	17.2787156	2.3968E+17
cg24717875	0.02379884	21.1401999	1.50004486	297.929791
cg21225608	0.02380891	707889.457	5.98526928	8.3723E+10
cg13553473	0.02381346	242162.762	5.18859227	1.1302E+10
cg17621438	0.02382128	15.5321662	1.43928546	167.616635
cg09975039	0.04762405	6.86173291	1.02037528	46.1431978
cg21767373	0.04762479	11804.9847	1.10314606	126327482
cg05868023	0.04763109	21.848039	1.03272714	462.209998
cg26622291	0.04764994	8.78179579	1.02275903	75.4038196
cg20373747	0.04765043	25.5782282	1.03413964	632.647402
cg16621855	0.04766707	0.11719382	0.01404049	0.97819851
cg03319638	0.04773044	14.7084645	1.02724746	210.600596
cg08526991	0.04774288	4.43274023	1.01491718	19.3603836
cg18640660	0.04776898	15.2592438	1.02714522	226.690946
cg26475097	0.04777227	2308484.95	1.15463892	4.6154E+12
cg17778955	0.04778378	43.9305119	1.03761719	1859.92474
cg05819594	0.04787851	11.3919855	1.02298662	126.861224
cg11090139	0.04788949	6712.03136	1.0853281	41509443
cg26922546	0.04791517	12.514562	1.02346396	153.02372
cg17516247	0.0479175	11.2911165	1.0224723	124.6873
cg10316593	0.04795882	6046.43677	1.08137259	33808326.5
cg12160586	0.04796858	0.07736564	0.00612398	0.97737724
cg09339476	0.04799233	294.76217	1.05152571	82627.3061
cg12302189	0.04799532	3946.08216	1.07579016	14474536.9
cg09952946	0.04801833	9.84550518	1.0201441	95.019882
cg17125688	0.04802529	44.086573	1.03344745	1880.7206
cg10824915	0.04802595	35.0678617	1.0313834	1192.33539
cg26147668	0.04808787	6.98E-05	5.29E-09	0.92265845
cg11751707	0.04814474	4.61072264	1.01254952	20.9952826
cg11928161	0.04815069	2658.60713	1.06623886	6629088.62
cg06796713	0.0481586	6.80402423	1.01565005	45.5813944
cg22315048	0.04817306	23.8156359	1.02579891	552.919782
cg15195412	0.04823369	14.5991124	1.02103796	208.742566
cg00177496	0.04823375	346.089935	1.04644908	114461.607
cg01294490	0.04824063	32.7704908	1.02735915	1045.30637

cg01772385	0.02382323	409986.431	5.56004791	3.0232E+10
cg21198712	0.02382604	9.47909203	1.34786355	66.6634141
cg08918020	0.02382889	16.2369687	1.44760011	182.121534
cg11571563	0.02383485	7.13460838	1.29783973	39.2210497
cg06644105	0.02384036	261.261828	2.0922027	32624.823
cg03416888	0.02384462	3.4937E+13	62.5231722	1.95E+25
cg06941635	0.02385146	15.1135731	1.43334834	159.361186
cg22671547	0.02385573	0.00400456	3.33E-05	0.48109152
cg13347514	0.02386088	2.6348E+15	110.527854	6.28E+28
cg02683846	0.02386211	22.0511321	1.50665467	322.736481
cg05775779	0.02386272	0.0366821	0.00208508	0.64533596
cg12491555	0.02386533	105.719317	1.85427415	6027.4658
cg01010839	0.02387382	12.0866998	1.39102469	105.022084
cg14400118	0.0238783	7.88435645	1.31443142	47.2927501
cg23341126	0.02388059	841673.428	6.08758882	1.1637E+11
cg25323497	0.02388351	6.7333904	1.28717371	35.2233316
cg01151699	0.02389109	7.61382114	1.30815912	44.3143892
cg01706819	0.02389686	23.9859864	1.52250119	377.883147
cg01072639	0.02390245	5.93129473	1.26547906	27.7999521
cg06217494	0.02391184	316.479162	2.14064671	46789.1593
cg03628403	0.02391539	631774160	14.5626131	2.7408E+16
cg18923197	0.02391545	22112.9952	3.75201548	130325836
cg24936695	0.02392793	0.17918062	0.04029236	0.79681848
cg14205663	0.02394203	0.10752223	0.01551858	0.74498009
cg12886494	0.02394408	0.16783918	0.03565345	0.79010575
cg18746774	0.02394408	0.03574985	0.00198389	0.64421511
cg21165793	0.02394745	467.187876	2.2507117	96975.7752
cg22747507	0.02395764	26.9061916	1.54391096	468.902136
cg19091677	0.02396435	62460192.6	10.6672494	3.6572E+14
cg02882785	0.02396747	6.91032639	1.29030843	37.0086793

cg00885546	0.04825877	38.6889949	1.02837736	1455.53411
cg03890222	0.04828492	6.98497538	1.01476178	48.0801329
cg19839026	0.04831007	14.0388655	1.01981618	193.260069
cg19929126	0.04831811	5.93842449	1.01325549	34.8035473
cg00402533	0.04831981	12.519057	1.01883769	153.829005
cg08757348	0.04832085	8.84349652	1.01621586	76.9594664
cg23548719	0.04833208	461713799	1.15747439	1.8418E+17
cg25835179	0.04833984	25.888701	1.02402329	654.501556
cg06000635	0.04834652	43.2236173	1.02774545	1817.84418
cg02573089	0.04835276	4141987.09	1.11660514	1.5364E+13
cg10932427	0.04835649	205237.013	1.09236025	3.8561E+10
cg09636214	0.04835841	3.6129561	1.0093092	12.9330554
cg06939307	0.04836156	6.58465508	1.01366211	42.7733087
cg19370322	0.04837035	23460594.7	1.12921826	4.8742E+14
cg09360748	0.04837548	295601.364	1.09408441	7.9866E+10
cg08527546	0.04837994	395511996	1.15132596	1.3587E+17
cg19910323	0.04838077	61899.3213	1.08166213	3542257682
cg19631443	0.04840264	3.6088E+11	1.20534009	1.08E+23
cg14601444	0.04841153	11.3848416	1.01711906	127.433084
cg20268054	0.04842523	0.10280675	0.01073688	0.98438512
cg05725263	0.04843481	13.0139328	1.0177999	166.400533
cg07114009	0.04845228	3.47157701	1.00849737	11.9503008
cg26631329	0.04846662	20141.2697	1.06902662	379476747
cg09424348	0.04847725	14.9311865	1.01824546	218.945567
cg01966966	0.04847792	872.255624	1.04630591	727158.154
cg10830649	0.04850284	17623.995	1.06639561	291266389
cg07800524	0.04850582	22.7682879	1.02072004	507.871807
cg19484164	0.04852461	44446.4366	1.0717974	1843152200
cg12610087	0.04853277	9.14854616	1.01436394	82.5107178
cg25994725	0.04853422	19.6492366	1.01935274	378.762411

cg15587603	0.02396766	394265.007	5.46809031	2.8428E+10
cg25041148	0.02397012	8.2721E+10	27.5031583	2.49E+20
cg23510527	0.02397044	6.2188832	1.27245303	30.3936628
cg21661138	0.02397215	49728.2813	4.16047821	594379260
cg07337598	0.02397918	6.57624191	1.28173088	33.7410595
cg16142306	0.0239814	4.29735379	1.21181811	15.2392916
cg13529912	0.02398225	11484687.4	8.5168935	1.5487E+13
cg27110548	0.02398489	107.368247	1.85168307	6225.65524
cg07116997	0.0239856	7.85679874	1.31203109	47.0486462
cg02819828	0.0239899	4.34374563	1.2134407	15.5492775
cg17010747	0.02399131	12.061942	1.38813989	104.809642
cg18884037	0.02399252	6.0644873	1.26793165	29.0063002
cg18358754	0.02399506	1.0344E+16	128.505107	8.33E+29
cg14852540	0.02400125	287687.092	5.23189634	1.5819E+10
cg17927777	0.02401586	23.565206	1.51542393	366.444612
cg14574127	0.02401931	26296.0085	3.81400147	181300419
cg03221676	0.02402298	3.1223E+10	23.998255	4.0624E+19
cg21871261	0.02402907	14.6079475	1.4227123	149.989656
cg21915765	0.02406486	18096.4764	3.62109137	90437502.5
cg19590188	0.02407616	851716728	14.8437717	4.887E+16
cg15107861	0.02407784	15.8825598	1.43725399	175.512268
cg18768283	0.02408036	322.783486	2.13340546	48837.0266
cg24512935	0.02408058	163564626	11.9484399	2.2391E+15
cg21826946	0.02408371	173368713	12.0356318	2.4973E+15
cg13736128	0.02409371	8.09392467	1.3153499	49.8054673
cg11505841	0.02409866	7.07965526	1.29238975	38.7820459
cg11173279	0.02409895	43780.457	4.05721855	472424246
cg02886027	0.02410371	542.223032	2.28153601	128863.106
cg13365753	0.02410943	8.07384431	1.3146564	49.5847902
cg00897404	0.02411188	49.1612273	1.6655152	1451.0983

cg12992778	0.04853817	4167396.86	1.10278958	1.5748E+13
cg01356198	0.04857119	5.52504173	1.01078025	30.2005171
cg18773129	0.04858554	209.869596	1.0337575	42606.9436
cg02605461	0.04859991	7.54524274	1.01249857	56.2279193
cg14957497	0.04861865	15938.9329	1.06042929	239572392
cg21724798	0.04862616	7.64429531	1.01234112	57.722886
cg08375849	0.04862777	36.1118939	1.02183821	1276.19898
cg24361162	0.04867041	4.69115617	1.00905997	21.8093541
cg06025835	0.04870689	18.0538668	1.01655276	320.634719
cg14029891	0.04871407	4623675863	1.13379426	1.8856E+19
cg23562261	0.04872555	5.55712417	1.00963656	30.5868768
cg09490277	0.04873289	9.27456332	1.01245933	84.9589927
cg17366544	0.04874653	9.42374124	1.01241254	87.7180947
cg02026377	0.0487504	2571.03414	1.04398674	6331705.44
cg02882785	0.0487542	7.02985837	1.01071553	48.8949733
cg21153962	0.04876824	330.329664	1.0318373	105750.865
cg25864218	0.0487984	56030.438	1.05931976	2963609395
cg22867063	0.04882963	6.98898304	1.01003013	48.3608186
cg14971941	0.04884552	53.6579417	1.02036838	2821.70121
cg10860308	0.04885032	0.11328906	0.01297611	0.98908001
cg25528940	0.04887185	997130.994	1.07071839	9.286E+11
cg08639762	0.04890007	10.4945172	1.01140174	108.893318
cg05721376	0.04890487	8.28140814	1.01020207	67.8891115
cg02806452	0.0489072	5.27363381	1.00799825	27.5905375
cg15857427	0.04890911	1152416.99	1.06903355	1.2423E+12
cg04936446	0.04891041	7014.85107	1.04321219	47169824
cg03856286	0.04896792	16478.9887	1.04490488	259886879
cg25346720	0.04901267	753382.95	1.06030139	5.3531E+11
cg19112186	0.04902455	9.05154786	1.00946126	81.1626182
cg04264633	0.04903422	8.37413129	1.00903435	69.4982036

cg02205746	0.02411239	3.69250111	1.18658576	11.4905849
cg17129764	0.02411597	23.9294716	1.51551978	377.837108
cg05569109	0.02411813	159.965185	1.94344602	13166.7461
cg00027499	0.02412024	3494.92239	2.9100161	4197393.44
cg04888113	0.02412058	27.9746558	1.54668806	505.972336
cg03271650	0.0241213	98.2931451	1.8232311	5299.13205
cg21844316	0.02412137	20714.8806	3.67319268	116821065
cg00506343	0.02412475	5.50590775	1.25016225	24.2488686
cg06070445	0.02412757	5.89478872	1.26134098	27.5488822
cg04769770	0.02412919	1.4292E+12	38.9616017	5.24E+22
cg13415434	0.02413534	15261.0644	3.52625216	66047485.1
cg00370303	0.02413653	759.097859	2.38117423	241993.866
cg16875032	0.02415136	0.04542474	0.00309109	0.66753437
cg07362258	0.02415391	146338.731	4.73330174	4524331065
cg27369431	0.02415551	12.4787392	1.39081612	111.962271
cg19280530	0.02415641	12725.7025	3.43922058	47087268.8
cg25751690	0.02415862	3.7641E+12	43.9948139	3.22E+23
cg10517814	0.0241668	370.81754	2.16576688	63490.512
cg09023136	0.02417157	9.27897504	1.337695	64.3639826
cg02603726	0.02417363	1.2742E+11	28.198103	5.76E+20
cg03345120	0.02417499	19723327	8.96568043	4.3389E+13
cg03216222	0.02418311	700020069	14.2744694	3.4329E+16
cg12690148	0.02418574	4.92353924	1.23126746	19.6880365
cg22122013	0.0241931	70.8402479	1.74343319	2878.42445
cg01405107	0.02419343	10.154626	1.35312208	76.2063016
cg26871717	0.02419398	5576.30829	3.08151299	10090891.8
cg22471129	0.02419596	16.1315501	1.43728805	181.054109
cg07352798	0.02419896	114.451781	1.85572528	7058.80898
cg07287384	0.02420283	325.97023	2.12688176	49958.8612
cg01515239	0.02421086	463.306359	2.22599645	96429.9748

cg10088527	0.04903824	1143380664	1.09188042	1.1973E+18
cg26360644	0.0490472	3.30E-05	1.14E-09	0.95783887
cg08301503	0.04907163	5.60054677	1.00703243	31.1470844
cg20431441	0.04909807	28.4485839	1.01331719	798.685682
cg20967975	0.04911259	7.72811383	1.00798099	59.250863
cg21793358	0.0491306	11.5533488	1.0093622	132.241794
cg05641885	0.04915269	0.01262595	0.00016202	0.98390584
cg12385383	0.04916573	261.245488	1.02054347	66875.3531
cg15104158	0.04918048	6.74047664	1.0068718	45.1239428
cg02007493	0.04918859	657027169	1.07481119	4.0164E+17
cg27025247	0.04920928	3794916.28	1.05385402	1.3665E+13
cg03114558	0.04922214	5849.21439	1.02998466	33217299.5
cg04677344	0.04922437	1697.47323	1.02557904	2809549.78
cg24008238	0.04922994	4.21505018	1.00486258	17.6806744
cg01534125	0.04924268	5.61090683	1.00573517	31.302749
cg10297971	0.04925318	20682631.1	1.05662215	4.0485E+14
cg09418959	0.04925698	856435251	1.06919738	6.8601E+17
cg20376123	0.04925886	70.423233	1.01390053	4891.43816
cg04562522	0.04926068	2729802.14	1.04913681	7.1028E+12
cg22364205	0.04926245	163.648346	1.01659648	26343.5706
cg00856204	0.0492626	92649.8402	1.03761065	8272845798
cg25261331	0.04926884	0.03393737	0.00116429	0.98922904
cg00243313	0.0492746	5.18058534	1.0052373	26.6986357
cg08931196	0.0492936	44386.5465	1.03364193	1906042568
cg19848291	0.0492955	108.479032	1.01455757	11598.8494
cg09265173	0.04930667	376427.828	1.03973156	1.3628E+11
cg03075725	0.04930749	48422.9646	1.03324058	2269349023
cg14033806	0.04933205	17.2613684	1.00836202	295.483997
cg08847885	0.0493645	141916.353	1.03354192	1.9487E+10
cg14283447	0.04936881	8.4059664	1.00589774	70.2459784

cg14367396	0.0242123	13860.102	3.46646517	55417382.7
cg00161556	0.02421236	20.047616	1.4781749	271.894014
cg09284397	0.02422213	199.699641	1.99397553	20000.2186
cg17942371	0.02422222	2.3314E+10	22.4289524	2.4235E+19
cg10963518	0.02422344	3.90682057	1.19427646	12.7803297
cg16993579	0.02422359	360.835505	2.15363951	60456.8504
cg00110785	0.02422399	9.00139677	1.3314586	60.854422
cg06856329	0.02422589	6.2771E+13	62.721228	6.28E+25
cg02290575	0.02423142	343.958564	2.13964169	55293.1337
cg27317046	0.02423602	3.73640184	1.18723628	11.7589892
cg20672465	0.02423673	259.771997	2.06245814	32718.9624
cg15438211	0.02423756	280.081738	2.0827123	37665.2021
cg19856002	0.02423956	108438.095	4.52380874	2599318653
cg20999932	0.02424331	4.39619053	1.21256217	15.9385568
cg07953400	0.0242443	4340696.9	7.30998474	2.5775E+12
cg26426223	0.02424461	987860178	14.8147474	6.5871E+16
cg04565008	0.02424787	50.2081206	1.66467064	1514.32681
cg12421755	0.02425224	3.50681854	1.177327	10.445506
cg07800892	0.0242645	0.17289078	0.03755413	0.79595027
cg16909733	0.02426536	4.93137957	1.23056468	19.7620693
cg10194844	0.02426563	51.4770924	1.66937089	1587.35908
cg19037922	0.02427039	4.75018665	1.22453063	18.4268754
cg15317837	0.02427845	3040748.87	6.95731575	1.329E+12
cg17953385	0.02427956	7.21289108	1.29271442	40.2453913
cg02456292	0.02428091	47.8089707	1.65280397	1382.92121
cg09945896	0.02428759	0.03889716	0.00230671	0.65590875
cg01256539	0.02429445	0.08952568	0.01096436	0.73099056
cg02125271	0.0242957	12.2688522	1.38475022	108.701722
cg00356183	0.02429917	3.11175143	1.15878216	8.35618401
cg14883008	0.02430952	118791825	11.1623567	1.2642E+15

cg04238038	0.04938792	10.8045341	1.00639464	115.996204
cg00326648	0.04939909	11.8996281	1.00653273	140.682111
cg19136717	0.04940542	2952.09221	1.02100435	8535564.47
cg02283238	0.04942015	6.54823076	1.00477876	42.6753907
cg06761377	0.0494234	8.23001692	1.00533147	67.3739764
cg23861541	0.049444	0.20696886	0.04300055	0.99617592
cg12507643	0.04945602	8.28361287	1.00504393	68.2738734
cg06168026	0.0494907	4.60734207	1.00340883	21.1554855
cg06023487	0.04952354	6.6181E+14	1.07369845	4.08E+29
cg25247520	0.04952968	7.45146756	1.00413941	55.2954779
cg26894854	0.04953459	23.3955482	1.00643698	543.850915
cg24425171	0.049547	5.75014358	1.00347105	32.9497807
cg20601684	0.04956477	3751.60046	1.01578533	13855787.8
cg19299094	0.04956803	34.2843973	1.00669864	1167.59857
cg19386159	0.04957023	60784.3748	1.02091494	3619048038
cg27223183	0.04959678	355135.081	1.02278568	1.2331E+11
cg02258703	0.04960874	9.39087138	1.00383847	87.8512507
cg10143811	0.04961457	3.26017217	1.00199329	10.6075787
cg23737055	0.04963951	550332571	1.03222229	2.9341E+17
cg04380939	0.04965074	8.1649102	1.00321082	66.4523921
cg23054456	0.04965913	0.16615479	0.02768134	0.99732941
cg16788391	0.04966793	1052.88874	1.01015188	1097433.69
cg27201679	0.04966915	4.33142899	1.00212199	18.7215502
cg18396533	0.04967127	12.7038371	1.00365882	160.79914
cg07357279	0.04968645	4.32239903	1.00200791	18.6456945
cg22469141	0.04970499	4166849.7	1.01984586	1.7025E+13
cg01893681	0.04971066	754.993519	1.00841398	565259.133
cg02679582	0.0497229	12053.1395	1.01144337	143634508
cg12206225	0.04974115	427005.705	1.01477123	1.7968E+11
cg05132828	0.04977504	13.1008652	1.00253169	171.199246

cg07825681	0.02430965	0.18783436	0.04383106	0.80494862
cg07098722	0.02431387	0.07754535	0.00837856	0.71769871
cg27585376	0.0243183	20.3166725	1.47785609	279.301337
cg21346043	0.02431864	5.59609615	1.25026266	25.0477704
cg07635623	0.02432253	604858522	13.7650516	2.6578E+16
cg11248715	0.02432519	14.2626426	1.41141387	144.12709
cg10835172	0.02433336	40185.246	3.9512669	408692716
cg26594902	0.02433999	267707.462	5.04967082	1.4192E+10
cg24673765	0.02434015	17.0704414	1.44431181	201.756965
cg16243019	0.02434329	9.3941124	1.33669846	66.0203856
cg12975295	0.02434695	15531.1733	3.49041206	69108558.1
cg11786946	0.02434909	0.00019205	1.12E-07	0.3301027
cg06961160	0.02435027	35.0212078	1.58490127	773.855772
cg21338852	0.02435431	88.6434903	1.78725497	4396.50106
cg17167832	0.02436837	24.5858369	1.51338726	399.410906
cg06733311	0.02437664	128.536857	1.87410882	8815.77607
cg02698708	0.02438576	25.5688122	1.52056035	429.949497
cg17147878	0.02438639	143.206221	1.89992768	10794.1065
cg07830160	0.02440029	3.86501518	1.19086078	12.5441551
cg26090107	0.02440422	3.47659257	1.1746407	10.2896961
cg13369332	0.02440798	954.225836	2.42569609	375375.526
cg11104474	0.02440993	16.583409	1.43718368	191.353032
cg08850949	0.02441038	122.215865	1.86008213	8030.13884
cg04604946	0.02441052	19.3502214	1.46609397	255.393635
cg00727675	0.02441273	5.78596688	1.25441854	26.6875939
cg15232240	0.02441312	313.525753	2.10052301	46797.1059
cg20484322	0.02441419	13.8581971	1.40415051	136.772821
cg14044905	0.02441705	1.31E-08	1.78E-15	0.09599398
cg08095637	0.02443375	8.71033387	1.32209357	57.3861924
cg09286468	0.02444156	0.03384442	0.00177254	0.64621561

cg25342397	0.04977896	409.968701	1.00582651	167100.721
cg04710768	0.04978132	8.34676121	1.00202921	69.5273375
cg00979026	0.04979639	1369.77097	1.00644472	1864257.89
cg04060561	0.04982128	7.70444128	1.00159524	59.2638752
cg12104022	0.04982668	0.05861758	0.00344341	0.99785473
cg09937190	0.04982991	5.88424404	1.00131753	34.5787693
cg02382426	0.04983882	5.88660541	1.00124872	34.6089066
cg06526137	0.04985913	40093.41	1.00654198	1597033769
cg14615266	0.04988668	5.04614382	1.0008013	25.4431799
cg10944735	0.04988932	10.6131958	1.00114233	112.5114
cg20051715	0.04989335	21.8813721	1.00143805	478.106904
cg03327649	0.04990659	22.7323393	1.001275	516.10122
cg00138497	0.04992049	142.614845	1.0017235	20303.9999
cg05213414	0.0499321	79.5295327	1.00129819	6316.74623
cg22476252	0.04994734	9.83300303	1.00052561	96.6371548
cg04354805	0.04995328	29635.4495	1.00210262	876417099
cg25170591	0.04995491	3.9267835	1.00026927	15.4154777
cg24241823	0.04996545	26.8574781	1.00049648	720.966183
cg09805692	0.04998717	57.7721068	1.00022727	3336.85796

cg25793521	0.02444231	6.29493812	1.26772848	31.2576759
cg15778012	0.02445225	8.38407202	1.31528317	53.4429887
cg03534647	0.02445285	3.4839E+11	30.7293873	3.95E+21
cg02276831	0.02445959	12.4301941	1.38360622	111.671748
cg18632102	0.02447878	3.50E-07	8.29E-13	0.14754835
cg24164786	0.02448031	20.8767628	1.47862729	294.759353
cg27652893	0.02448197	16.4833655	1.4342938	189.432137
cg01134183	0.02448431	66.3792368	1.71583707	2567.96123
cg04632671	0.02448883	0.0057253	6.37E-05	0.51465287
cg12763105	0.02449051	2.14E-05	1.82E-09	0.25071676
cg03351412	0.02449824	6.4049E+12	44.3581818	9.25E+23
cg05168062	0.02450165	55.7496007	1.67701931	1853.29886
cg10919204	0.02450406	0.23380652	0.06589595	0.82957289
cg13781389	0.02450995	1.3319E+11	26.9068517	6.59E+20
cg11717738	0.02451725	11.6909481	1.3715338	99.6535904
cg14865907	0.0245179	452.623949	2.19392353	93379.9364
cg10473588	0.02452499	48998.113	4.0032579	599715317
cg14153069	0.0245268	6.72089729	1.27722334	35.3661406
cg09953959	0.02452849	764559.481	5.69572044	1.0263E+11
cg22656956	0.02452896	44.8309474	1.62963817	1233.2884
cg26640895	0.02453047	2.0957E+15	92.7648142	4.73E+28
cg09089053	0.0245396	45.2567339	1.63121492	1255.61135
cg01961105	0.02454036	5.42216365	1.24230865	23.6655026
cg00455450	0.02454265	6189547172	18.0552199	2.1219E+18
cg02494781	0.0245433	219694.026	4.84747573	9956824452
cg03420907	0.02455299	4.72719377	1.22048398	18.3094258
cg27342919	0.02456095	0.28716727	0.09677189	0.85215905
cg12489322	0.02456605	8.9663E+10	25.3531891	3.17E+20
cg10603826	0.02457541	63997.0349	4.12891071	991937285
cg18274325	0.0245755	68882.4291	4.16798957	1138387936

cg16754467	0.02458022	4.29954906	1.20543806	15.3356051
cg12106855	0.02458298	5.7838791	1.25207864	26.7181759
cg02462253	0.02458409	5.70832142	1.24995673	26.0688492
cg04114121	0.02458448	1136.06148	2.46226254	524166.561
cg13352650	0.02458807	5767573.03	7.34170432	4.531E+12
cg16096796	0.02459526	8.07453919	1.30654299	49.9012918
cg10531737	0.02460119	8449401562	18.6386424	3.8303E+18
cg01968002	0.02460159	8.96641352	1.32407266	60.7191538
cg01805469	0.02460412	71256.2414	4.17799129	1215285431
cg03419278	0.02460589	6.87E-11	9.42E-20	0.05007542
cg04233770	0.02461609	14.7775548	1.41116344	154.748997
cg05426966	0.02462898	5.84177706	1.2530585	27.23445
cg16860686	0.02463865	9.14360841	1.32673138	63.0162034
cg01650726	0.02464335	27.1689445	1.52462226	484.153726
cg22156128	0.02465079	11.311832	1.36304874	93.8759844
cg13296579	0.02465231	0.17448196	0.03804562	0.80019596
cg25726357	0.02466481	12.04269	1.37369731	105.573755
cg16705353	0.0246704	103.107958	1.80638565	5885.37166
cg21712685	0.02468032	43692.5906	3.90516469	488850696
cg09219813	0.02468432	8.29438309	1.30954418	52.5349139
cg23910392	0.02469845	387.046872	2.13623061	70125.9877
cg24925865	0.0246996	2483306.91	6.52530513	9.4506E+11
cg25506900	0.02471503	8042.01923	3.1412392	20588713.3
cg06002370	0.02472117	43.9566484	1.61835544	1193.92
cg09636214	0.02472242	3.61083985	1.17747342	11.073001
cg03935183	0.02472357	12.4513041	1.37831834	112.481254
cg14007549	0.02472975	29.7584621	1.53971189	575.150501
cg14497203	0.024733	7.65174453	1.29537233	45.1987378
cg13744172	0.02473593	5.57694517	1.24426171	24.9966041
cg16792613	0.02473927	497.715963	2.20238634	112478.531

cg03609308	0.02474556	189.227906	1.94719068	18389.1598
cg19197795	0.02474856	36.562382	1.57992237	846.122444
cg19824325	0.02474961	43.7828996	1.61648683	1185.86942
cg17055959	0.02475628	15030.5334	3.39335132	66576347
cg05888917	0.02476512	4.73126684	1.21817787	18.3757122
cg23778358	0.02476711	0.22586268	0.06162146	0.82786017
cg18640660	0.02476726	13.8844658	1.39658387	138.03567
cg14823287	0.02476828	1.6646E+11	26.5889807	1.04E+21
cg14021524	0.0247692	7390219.98	7.44789321	7.333E+12
cg00699721	0.02477317	363277372	12.2064043	1.0812E+16
cg23512654	0.02478778	845347048	13.5630391	5.2688E+16
cg11386080	0.02479081	10.2788354	1.34382376	78.622257
cg24344221	0.02479671	593329.596	5.39518342	6.5251E+10
cg17214455	0.02480064	9.12495856	1.32350786	62.9122587
cg15333318	0.02480349	7.70707195	1.29542974	45.8527052
cg02637304	0.02480422	16.7497491	1.42934976	196.280928
cg00282706	0.024806	0.1185156	0.01840503	0.76315795
cg26712096	0.02480609	25.1592529	1.50493365	420.608581
cg26620147	0.02480671	7.02341742	1.28021858	38.5312265
cg10684940	0.02481025	15220.8325	3.38809792	68378703.2
cg02641983	0.02481133	114.293047	1.82287882	7166.08284
cg11848563	0.02481289	12.0467315	1.37069075	105.876355
cg00765922	0.0248238	4.34750057	1.20453351	15.6913535
cg06793967	0.0248287	0.01937842	0.00061867	0.60698459
cg06791473	0.02482936	2184.68554	2.6469592	1803144.87
cg22693994	0.02483052	46.7609569	1.62699344	1343.94339
cg26440766	0.02483166	8.7494E+11	32.4801598	2.36E+22
cg17084151	0.02484887	5.67785259	1.24562215	25.8810506
cg24942416	0.02484923	6.6381628	1.27047957	34.6839149
cg05641885	0.02486003	0.01105105	0.00021584	0.56580817

cg17581200	0.0248615	2542.50106	2.6941657	2399374.19
cg04886060	0.02486225	6.12891059	1.25753909	29.8706778
cg01819512	0.02486402	10.3637745	1.3438344	79.9263823
cg27200454	0.02486545	818485.304	5.58807206	1.1988E+11
cg06615378	0.02486753	2728.00414	2.71747041	2738578.7
cg16262756	0.02486959	17190.4203	3.42872181	86186796.7
cg15593510	0.02487143	0.21897931	0.0580951	0.82540419
cg03324851	0.0248861	143.347527	1.87174472	10978.2671
cg02272859	0.02488626	7.10268948	1.28083228	39.3870443
cg19632236	0.02489012	6.16590793	1.25811062	30.2186628
cg08747889	0.02489235	85.8160595	1.75405067	4198.50817
cg02605461	0.02489277	7.10376196	1.28075789	39.4012282
cg01937803	0.02489303	15.128538	1.40896838	162.439886
cg00067742	0.02489343	0.25777406	0.0788468	0.84274148
cg25198784	0.02489744	1414082.45	5.97149226	3.3486E+11
cg02344701	0.02490659	0.00693493	9.00E-05	0.53419076
cg27083176	0.02491549	2594191.09	6.43629503	1.0456E+12
cg11550381	0.02491566	383186.545	5.05725291	2.9034E+10
cg04544938	0.0249164	20897537.3	8.37201798	5.2163E+13
cg06598256	0.02492206	617479.747	5.36802838	7.1028E+10
cg24421410	0.02492366	5.55428236	1.24119827	24.8550561
cg14273822	0.02492946	3.56145488	1.17354327	10.80826
cg26898127	0.02493382	0.00771064	0.00010973	0.54181596
cg07880384	0.02493402	22.389565	1.47929249	338.873227
cg16265348	0.02493991	7.57739744	1.2904897	44.4923753
cg05765761	0.02495853	0.09985892	0.01332492	0.74835747
cg09775785	0.02496548	3169247.79	6.57108836	1.5285E+12
cg06023487	0.02497287	4.5989E+14	69.7422778	3.03E+27
cg08924619	0.02497933	8.03577086	1.2994255	49.6939713
cg17299902	0.02498232	1368519.55	5.9039728	3.1722E+11

cg19882886	0.02498391	22.5081026	1.47888986	342.564174
cg14335434	0.02498447	676.726357	2.26809954	201912.903
cg21616552	0.02498557	107.675493	1.80026655	6440.16399
cg17737146	0.02498687	4.44439802	1.206127	16.3769435
cg27634164	0.02498759	0.12484525	0.02024298	0.76996249
cg15048437	0.02499138	1863918283	14.6053082	2.3787E+17
cg16830078	0.02499183	3304934.31	6.59008635	1.6574E+12
cg20223677	0.02500015	0.26486329	0.08288778	0.84635591
cg02929073	0.02500054	5.92518412	1.2503188	28.0790842
cg24046271	0.02500141	167170.795	4.52683462	6173425179
cg08113093	0.02500259	2.0719E+10	19.7333193	2.1754E+19
cg26092468	0.02500411	32737.0282	3.68818854	290579779
cg01585985	0.02500686	47173266.8	9.18797623	2.422E+14
cg22487177	0.02502671	2162.41062	2.61945095	1785114.43
cg00060374	0.02503192	4.72193711	1.21482952	18.3537605
cg02205221	0.0250337	80016.2641	4.11779924	1554860287
cg19764541	0.02503779	1019145.39	5.66302045	1.8341E+11
cg24000528	0.02503997	11.6370455	1.36011124	99.5659946
cg06796825	0.02504082	3148.47355	2.74393449	3612653.92
cg21767373	0.02504104	24510.9927	3.54861449	169302348
cg06511482	0.02504496	498.46794	2.1776822	114098.507
cg21139076	0.02505681	617311.679	5.31000586	7.1765E+10
cg03155239	0.02505844	609159701	12.5878112	2.9479E+16
cg22648949	0.02507834	6.59438662	1.26611157	34.3460528
cg20784775	0.02507987	32.0343111	1.54285909	665.126903
cg14196353	0.0250828	23873617.3	8.36987991	6.8095E+13
cg05818685	0.02509272	6.50333191	1.2637066	33.4676782
cg25332377	0.02509539	10.1237482	1.33553973	76.7407178
cg25512381	0.02509561	22.7627875	1.47787157	350.601841
cg19401111	0.02509943	27.0010609	1.50963634	482.935706

cg05988291	0.02510375	5.40383225	1.23464743	23.6516129
cg22960945	0.02510487	8.35356766	1.30368146	53.5269502
cg20641531	0.02510743	0.22733841	0.0621882	0.83107004
cg13847963	0.0251105	23.845955	1.48606156	382.642003
cg20600850	0.02511569	7.94665508	1.2953985	48.7489578
cg01596674	0.02512059	17.5145822	1.42962824	214.573677
cg09353354	0.02512587	13625427	7.76978475	2.3894E+13
cg16536824	0.02512692	9.27552504	1.32045441	65.1558767
cg13247671	0.02513011	2.24E-06	2.55E-11	0.19730177
cg17836177	0.02513149	11.8918334	1.36194958	103.833287
cg25361850	0.02513161	17.2731797	1.42688382	209.100932
cg06146234	0.02513395	7.63807531	1.28872043	45.2698607
cg12876594	0.02514282	6.45010549	1.2617003	32.9744401
cg07144296	0.02514558	36.1540948	1.56416526	835.665261
cg23346960	0.0251575	7302.35754	3.03004089	17598582.9
cg02933679	0.02515963	10.354331	1.33809581	80.1229405
cg27345592	0.02516092	38150.9312	3.72249134	390999848
cg19722847	0.02516688	92.3220738	1.75710324	4850.80507
cg06326971	0.02516902	39.7891068	1.58214077	1000.65244
cg15354705	0.02517088	3.09E+10	20.2474657	4.7157E+19
cg02152119	0.02517695	28642.9835	3.58834375	228634869
cg04734688	0.02518209	389293.12	4.96376308	3.0531E+10
cg05390144	0.02519389	34.6376505	1.55423074	771.93611
cg14400871	0.02519651	12044.8198	3.21792503	45084233.9
cg26782361	0.0251988	13.8832633	1.38704022	138.961363
cg00114963	0.02520561	238.579785	1.97516606	28817.9889
cg03899598	0.02520842	16.0950857	1.41255054	183.392932
cg24368167	0.02521102	9588.99323	3.12544511	29419422.8
cg10601100	0.02522293	0.03618653	0.00197768	0.66212279
cg26513180	0.02522462	28.5030204	1.51605877	535.877756

cg23772500	0.02522835	547729737	12.1687387	2.4654E+16
cg24828620	0.02522846	4.38432044	1.20148308	15.9987819
cg01783195	0.02522917	129.289907	1.82904994	9139.10532
cg05883442	0.02522932	32.9341608	1.54336366	702.788962
cg25098174	0.02523013	14.6185588	1.39526748	153.162217
cg01250407	0.02523038	8.00007803	1.29462681	49.43606
cg01441105	0.02526114	53764.8985	3.85968757	748937386
cg07245011	0.0252645	9962364403	17.3593041	5.7173E+18
cg13323474	0.0252777	60583.6904	3.91297783	938002641
cg18862975	0.02528544	12.5427855	1.36783584	115.014875
cg24693053	0.02528763	5.46226739	1.2339925	24.1787248
cg23780961	0.0252914	387424616	11.5697383	1.2973E+16
cg00955828	0.02529856	4.4418929	1.20267739	16.4054074
cg22777668	0.02530229	20.9006274	1.45668568	299.883654
cg20314660	0.02530296	930436562	12.876942	6.723E+16
cg21678189	0.02530938	1511.19313	2.47341293	923301.021
cg24918060	0.0253176	12.9935612	1.37315151	122.952661
cg17492041	0.0253244	518459983	11.9472118	2.2499E+16
cg17382841	0.02532514	11.2353258	1.34853786	93.6069721
cg11206041	0.02532685	4.6760424	1.21003229	18.0700736
cg18426477	0.02532756	12.6912447	1.36895269	117.657602
cg07447260	0.02532938	2.49E-11	1.27E-20	0.04893655
cg25055477	0.02532949	688.657501	2.24249966	211482.375
cg12461469	0.02533356	468.151916	2.13772805	102522.964
cg23849812	0.02533444	0.21386286	0.05533954	0.82648539
cg14563754	0.02533934	1.316E+13	41.7408118	4.15E+24
cg02304930	0.02534589	99.5291496	1.76489529	5612.82681
cg08231730	0.02535063	7.60544517	1.28464038	45.0264502
cg18108237	0.025352	1.12E-06	6.86E-12	0.18433394
cg07212778	0.02535461	4.4760595	1.20321311	16.6513384

cg22090592	0.02535653	12.9632769	1.37193608	122.488613
cg09459581	0.02535758	8.47531456	1.3018151	55.1775417
cg05134500	0.02535843	132.913915	1.82840265	9662.04499
cg06750292	0.0253707	12.1341708	1.3605018	108.223379
cg08881680	0.02537242	3.60426168	1.1713067	11.0907777
cg10832938	0.02537295	13.9872703	1.38451126	141.308876
cg14227687	0.02537605	5.6093E+12	37.3259696	8.43E+23
cg01386425	0.02537812	96.0685588	1.75565309	5256.82894
cg02642822	0.025387	5.33565716	1.22918902	23.1609922
cg08947062	0.02538824	12.0120443	1.35845119	106.215969
cg16727916	0.02538874	27137966.3	8.242089	8.9355E+13
cg27536187	0.02539091	976143.024	5.47026121	1.7419E+11
cg17371249	0.02539413	8.3623E+10	22.1616653	3.16E+20
cg25899024	0.02539616	9721.48117	3.099059	30495449.1
cg12219828	0.02539655	18483139.4	7.85494119	4.3492E+13
cg09131151	0.02540111	0.13724492	0.02405552	0.78302889
cg21506220	0.0254069	1.89E-05	1.36E-09	0.26204319
cg00051483	0.02541321	0.10822632	0.01540009	0.76057569
cg21864868	0.02541445	6.39985133	1.25666669	32.5926496
cg16749930	0.02541712	13.3056573	1.37506708	128.750457
cg21649520	0.02541887	16.719483	1.41422007	197.664506
cg24462260	0.02542452	257.290172	1.97933867	33444.6214
cg19434107	0.02542835	8579.93149	3.04642445	24164467.4
cg07849384	0.02542839	5.83858927	1.24236791	27.4388323
cg02400595	0.02542839	0.08582627	0.00996316	0.73933822
cg08277369	0.02543077	9.24151808	1.3145151	64.9712253
cg19537645	0.02543592	3.1177E+10	19.5070431	4.9827E+19
cg03302259	0.02543773	11.296814	1.34724654	94.7250573
cg07218880	0.02544567	14.6524466	1.39084261	154.36268
cg13714271	0.02544608	1822.97446	2.51598146	1320850.71

cg06318386	0.02545295	287.89762	2.00496768	41339.8384
cg13736131	0.02545823	8.45114654	1.29968642	54.9531614
cg16824282	0.02546138	0.00745278	0.00010137	0.54793626
cg10513852	0.02546439	0.18096028	0.04039417	0.810677
cg14019833	0.02549261	2113525.1	5.96353352	7.4905E+11
cg06907744	0.02549263	36142.6564	3.6212168	360732783
cg11378840	0.0254933	128.874288	1.81433333	9154.09637
cg16806210	0.0255005	343.138831	2.04527874	57568.8071
cg26575637	0.02550407	11.6496243	1.35103529	100.451666
cg22927247	0.025513	7.86144394	1.2873239	48.0083535
cg11555438	0.02551412	8.1242781	1.29250198	51.0667647
cg05710032	0.02552002	12.3288593	1.36012481	111.755018
cg27018502	0.02552352	35.582614	1.54849527	817.646938
cg01988009	0.02552952	149992.452	4.30025296	5231723750
cg02679582	0.02553126	51689.8403	3.77415607	707930339
cg16376036	0.02553374	121.385672	1.79898135	8190.45807
cg07974719	0.02554091	8.29062348	1.29527668	53.0654483
cg20797766	0.02554236	24899.48	3.44912385	179751187
cg09729613	0.02554761	5.3838773	1.2285678	23.5934352
cg22946648	0.02554868	8.88324989	1.30612999	60.4167496
cg03072286	0.02554997	11.1177501	1.34243545	92.0747195
cg17355919	0.02555112	1447.91753	2.43463171	861101.561
cg22424532	0.02555571	5.4367132	1.22993534	24.032036
cg03965648	0.02555993	8.72858087	1.30313853	58.4650994
cg19800926	0.02556036	16463422	7.61896639	3.5575E+13
cg25256099	0.02556421	5.1088225	1.22051805	21.3844173
cg19038034	0.02556642	15866.336	3.25962863	77229846.8
cg06767637	0.02556754	5.81699598	1.23998782	27.2885278
cg09692396	0.02557151	14.8100992	1.38985793	157.814
cg00402533	0.02557335	13.116546	1.36935729	125.638342

cg01200642	0.02557826	2.2889E+10	18.4032518	2.8468E+19
cg23018117	0.02558135	0.18108501	0.04039827	0.81171252
cg06132502	0.02558266	8.69769082	1.30219253	58.0941943
cg01461514	0.02558521	8.82384404	1.30444041	59.6886011
cg09924165	0.02559332	4.86284767	1.21284895	19.4973063
cg13415207	0.02559703	2.50E-07	3.99E-13	0.15654449
cg01518659	0.02559712	3.37E-07	6.98E-13	0.16233661
cg22343980	0.02560155	1.6575E+10	17.6344959	1.56E+19
cg02451774	0.02560361	9.87549559	1.32215998	73.7621882
cg19675288	0.02560398	3.93928329	1.18197514	13.1288318
cg17711535	0.02561142	196458.594	4.41830034	8735481112
cg08278489	0.02561304	6.42877457	1.25459595	32.9421935
cg09106154	0.02561656	0.00206399	9.05E-06	0.47069785
cg10971510	0.02561961	5.86982006	1.24067681	27.7709611
cg12869659	0.02562135	6.13672789	1.24739432	30.1904768
cg20733436	0.02562182	6.08326246	1.24605872	29.6985058
cg14112754	0.02562633	6.38391158	1.2533417	32.5165333
cg13486406	0.02562751	29.5550438	1.51053449	578.272539
cg25006823	0.02563404	30.7859645	1.51785866	624.416251
cg24062310	0.02564025	0.06856281	0.00651418	0.72163519
cg17861295	0.02565241	3.73968129	1.17405757	11.9118657
cg23176538	0.02565597	621.346061	2.1866024	176562.015
cg08572535	0.02566063	12.2393907	1.356065	110.46866
cg07744166	0.02566992	15.3821395	1.39407266	169.725885
cg09423126	0.0256801	7.54859826	1.27836282	44.5736804
cg23264525	0.02568145	27104.5736	3.45577617	212588396
cg09731079	0.02568163	10.2445933	1.32665586	79.1099605
cg20141824	0.02568648	46312.317	3.68697195	581732310
cg01910527	0.02568694	0.20696632	0.05186622	0.82587588
cg00208412	0.02569376	254381.525	4.53196732	1.4279E+10

cg12500707	0.02569836	11.0215344	1.33817049	90.7763405
cg15233114	0.02569958	46534004.2	8.52488475	2.5401E+14
cg19053239	0.02569958	3.81632545	1.17651694	12.3792012
cg01879488	0.02569959	75.6181715	1.69052377	3382.44748
cg06761377	0.02570256	7.16061796	1.26986012	40.3780296
cg25791744	0.02570595	22403489.5	7.79611862	6.438E+13
cg02131399	0.02571167	38835176.5	8.3293248	1.8107E+14
cg09969043	0.02571543	0.18078154	0.04021632	0.81265419
cg22125968	0.02572142	6.78422494	1.26129553	36.4908199
cg13233166	0.02572384	35.2497841	1.54015945	806.76535
cg13951491	0.02574513	6.59862122	1.25672662	34.6469958
cg01240734	0.02574666	14726773.6	7.37966307	2.9389E+13
cg16907919	0.02575183	4380543.75	6.36891377	3.0129E+12
cg06435751	0.02575324	4920.05135	2.79860961	8649618.44
cg03459809	0.02575357	5.06941074	1.21713665	21.1142482
cg21686379	0.02575591	13.2049028	1.36664643	127.589298
cg00692933	0.02575869	29591354.2	8.02052071	1.0918E+14
cg07424616	0.02576227	792066946	11.9340812	5.257E+16
cg01728712	0.02576228	231.370845	1.93237576	27702.9286
cg26102728	0.0257634	14.8629474	1.38618755	159.363143
cg06119923	0.02577045	468.20127	2.10379977	104198.333
cg12130607	0.02578457	0.16149759	0.03251193	0.80221244
cg03034880	0.02579308	11.7023732	1.34607895	101.736632
cg00381622	0.02579552	36.0293139	1.54189593	841.893041
cg18245365	0.02580169	1.8783E+13	40.0939673	8.80E+24
cg19116023	0.02580288	6.3776757	1.2507595	32.5200386
cg08519905	0.02581524	6.72705028	1.2586654	35.9533245
cg04533380	0.02582001	176951360	9.89011659	3.166E+15
cg23295999	0.02582564	12352.4602	3.11588958	48969409.8
cg24014780	0.02582628	86329544.9	9.06352454	8.2228E+14

cg01734112	0.02582768	6.30116106	1.24859976	31.7993258
cg05487736	0.02583238	536329.197	4.90799313	5.8608E+10
cg17557230	0.02583418	3456.02455	2.67100136	4471770.73
cg00612202	0.02583817	218828.957	4.40323912	1.0875E+10
cg02219147	0.02584305	3905780.08	6.2297093	2.4488E+12
cg03317618	0.0258438	89243.8796	3.9504646	2016084399
cg11926393	0.02584514	38969.1242	3.57471418	424815121
cg23665824	0.02584958	6.52548659	1.253574	33.9684575
cg09183102	0.0258515	37768.8848	3.5598478	400716193
cg13094036	0.02585234	0.10579255	0.01467014	0.76291443
cg07765912	0.02585534	12390.2621	3.11187352	49333172.9
cg17493815	0.02585666	11.8678338	1.34710771	104.553985
cg18527241	0.02585868	5.70722763	1.23338412	26.4090049
cg17457701	0.02585945	0.06710954	0.00623529	0.72229033
cg16391678	0.02586409	8.9870584	1.30261539	62.003888
cg17724175	0.02586838	7.8459835	1.28142573	48.0398164
cg08499107	0.02586993	281563.144	4.52839485	1.7507E+10
cg08898253	0.02587017	35.505226	1.53674002	820.321623
cg04240980	0.02588211	6.3599443	1.24925355	32.3784483
cg26572233	0.02589041	0.07469913	0.0076227	0.73201846
cg08843298	0.02589269	3.5233E+10	18.538466	6.6961E+19
cg01445100	0.02590821	35.7813509	1.53693502	833.024854
cg22394521	0.02591192	474036162	11.0183627	2.0394E+16
cg12308275	0.02591328	49.1718679	1.5965819	1514.40561
cg23440155	0.02591554	6.01762411	1.24052619	29.1906775
cg07115761	0.02591666	10.0160025	1.31878155	76.07045
cg16996661	0.02592792	60978293.4	8.59803133	4.3247E+14
cg05990544	0.02593217	4.71539883	1.20453854	18.4593397
cg16565692	0.02594467	8.23723368	1.28772749	52.691287
cg20849121	0.02594826	5479981.2	6.42683171	4.6726E+12

cg00652657	0.02594903	10.0326951	1.31846309	76.3426532
cg03430067	0.02595149	7.8541E+12	35.1486585	1.76E+24
cg27470087	0.02595175	11.2579853	1.33675294	94.8135056
cg03352866	0.02595325	6798.56642	2.88007337	16048377.8
cg19241779	0.02595944	11773.1476	3.07497418	45075827.3
cg11475454	0.02596188	12.3150626	1.35100942	112.257373
cg14277392	0.02596351	6.54443279	1.25242466	34.1973469
cg02780181	0.02596809	1499377.19	5.49266957	4.093E+11
cg12008118	0.02598035	29.4818361	1.49942441	579.674876
cg16421621	0.02599126	385659.271	4.65994066	3.1917E+10
cg26468205	0.02599304	221.864504	1.90845093	25792.5719
cg27180443	0.02600087	0.09237221	0.01134476	0.75212044
cg17928147	0.02600283	4.560335	1.19895497	17.3456518
cg10143811	0.0260066	3.49707176	1.16145967	10.5294323
cg18312782	0.0260112	30.0263274	1.50178163	600.340503
cg12595281	0.02601228	4.88604313	1.20877962	19.7500165
cg27285056	0.02602477	10.1444642	1.31884744	78.0303706
cg25989526	0.02602531	0.03816014	0.00215101	0.67698198
cg09745243	0.02602531	3188.82924	2.62119495	3879387.89
cg21943938	0.02602832	43.5846952	1.56958547	1210.2722
cg26945941	0.026033	0.17741213	0.03869363	0.81344307
cg02431562	0.02603361	8.70333406	1.29478268	58.5024998
cg07397574	0.02603909	2.2237E+11	22.6180576	2.19E+21
cg17283453	0.02604297	5.59261229	1.22806864	25.4687005
cg21466150	0.02604618	11.3222166	1.33585544	95.9629201
cg12477903	0.02607927	29.2007286	1.49475748	570.44876
cg03471346	0.02608596	0.21010494	0.05315734	0.83044201
cg26084319	0.02608646	2252400.1	5.70839845	8.8874E+11
cg02077165	0.02609672	73815.4462	3.79696068	1435021469
cg02291472	0.02609719	4897734073	14.2342599	1.6852E+18

cg22488472	0.02610212	406.004685	2.04361953	80660.7109
cg11181536	0.02611158	1.069E+15	61.3086884	1.86E+28
cg24658737	0.02611416	0.06573505	0.0059729	0.72345015
cg00487737	0.02611446	15.9602228	1.39016753	183.235982
cg26464221	0.02612014	6.35883384	1.24598239	32.4521181
cg05855588	0.02612284	4.53850762	1.19698473	17.2082826
cg14993900	0.02612446	6.79290136	1.25574132	36.7460305
cg12358524	0.02612508	5.32885604	1.22001979	23.2756115
cg11845241	0.0261254	0.14570446	0.02669149	0.79537675
cg27392850	0.02612582	3.92977182	1.17663903	13.1247614
cg18843682	0.02613023	7.05217296	1.261261	39.4312861
cg18009321	0.02614151	5.45495615	1.22321516	24.3265025
cg11584101	0.02614696	0.17512031	0.03771479	0.81313256
cg00131871	0.02615198	70.2839727	1.65661878	2981.87904
cg17026456	0.02615353	1337020.69	5.3348114	3.3509E+11
cg17593472	0.02615699	10.9541465	1.32851889	90.3211284
cg04981611	0.0261608	5.13184933	1.21415153	21.6907666
cg00839844	0.02616088	95.2932522	1.71716409	5288.25637
cg23039189	0.02616665	7.53821177	1.27073892	44.7177906
cg26058820	0.02616701	0.19058697	0.04421564	0.82150555
cg26354439	0.02617164	0.01873555	0.00056256	0.62397152
cg26174326	0.0261799	10.1370353	1.31594175	78.0881714
cg26043149	0.02618016	1763.44784	2.42552647	1282092.08
cg07470489	0.02619116	14.3059726	1.37054673	149.32789
cg24137987	0.02619203	5878915.09	6.3375617	5.4535E+12
cg17278453	0.02619239	10.0707529	1.31469571	77.1433747
cg15141467	0.02619424	7.2440383	1.26434655	41.5045155
cg22723056	0.02620318	4.4140668	1.19219778	16.3429139
cg16556936	0.02620376	10116779.5	6.75097184	1.5161E+13
cg05912499	0.02620714	45.028905	1.56939856	1291.96136

cg04234631	0.02621319	0.01107846	0.00020911	0.58692612
cg04580334	0.02622335	2.5628E+10	17.0273682	3.8574E+19
cg17284325	0.02622822	3.3553E+13	39.7596011	2.83E+25
cg18395988	0.02623006	1.4951E+12	27.51346	8.12E+22
cg26537098	0.02623144	5.17883666	1.21463468	22.0810006
cg09061824	0.02623235	7923782.94	6.54098696	9.5989E+12
cg02628508	0.02623303	5.98E-13	9.95E-24	0.03588304
cg08991927	0.0262342	5.53105026	1.22408672	24.9921157
cg13430464	0.02623548	9.75110044	1.30892597	72.6427329
cg02618319	0.0262378	6.18580165	1.24033615	30.8498161
cg17327665	0.02623893	688376.426	4.89739787	9.6758E+10
cg20706597	0.02624287	0.00369455	2.65E-05	0.51590469
cg21758133	0.02624354	10993.8494	3.00267993	40252283.7
cg04478795	0.02624437	7.13980296	1.2614465	40.411374
cg16676472	0.02624476	6.15E-07	2.05E-12	0.18454645
cg10118828	0.02624632	23.1652391	1.44960316	370.189797
cg14223995	0.0262473	6.36636343	1.24443258	32.5695292
cg03893307	0.02624739	1.8946E+12	28.2133204	1.27E+23
cg08916340	0.02624862	753.356316	2.18708321	259498.923
cg00222341	0.02624881	10.2984833	1.31716336	80.52058
cg19196335	0.02625063	10.867204	1.32551988	89.0941919
cg19818312	0.02625272	3863.85484	2.65249611	5628424.54
cg10976626	0.0262531	0.24564106	0.07122144	0.84721022
cg00181849	0.02625518	0.15736722	0.03080829	0.80382406
cg13447539	0.02626145	56.7667681	1.61095014	2000.35115
cg08932052	0.02626215	460798.491	4.66219558	4.5544E+10
cg04220455	0.02626587	41.7227339	1.55329278	1120.70728
cg22622477	0.02627322	8.54385458	1.28802204	56.6740697
cg22439359	0.02627727	249.544046	1.91767823	32472.7214
cg27298551	0.0262773	0.00996546	0.00017104	0.58062556

cg14301212	0.02628176	11.2664227	1.33059098	95.395417
cg15983200	0.02628392	12900.1733	3.05308982	54506902.9
cg15659713	0.02628584	3.72422953	1.16770376	11.8779147
cg10978585	0.02628762	0.18463953	0.04160559	0.8194033
cg10314411	0.02628951	0.06137207	0.00523395	0.71963451
cg12568595	0.02629314	1547.04545	2.37655296	1007067.66
cg02414175	0.02629615	222.603493	1.89087413	26206.0358
cg10815165	0.02630079	21.8077764	1.43787889	330.750465
cg22209752	0.02630304	1050960514	11.557178	9.557E+16
cg06212224	0.02630465	7.77900391	1.2733658	47.5220095
cg04498679	0.02630661	6.26033315	1.24117243	31.5764114
cg16048771	0.02631127	0.06012901	0.0050344	0.71815852
cg21551132	0.02631412	8.8758E+11	25.5191512	3.09E+22
cg07633851	0.0263209	9.04329341	1.29588532	63.1083279
cg19484164	0.02632159	27352.0352	3.32849549	224766365
cg08203284	0.02632698	9.67343966	1.30609532	71.6451802
cg07701716	0.0263292	113.64428	1.7452303	7400.18229
cg05508408	0.02633345	310225.323	4.42594213	2.1744E+10
cg02560808	0.02633879	3824.11279	2.63823687	5543034.74
cg10089865	0.0263508	24.7691598	1.4582397	420.720459
cg05308135	0.02635393	0.1205561	0.01863593	0.77987928
cg00372169	0.02635423	95353.5527	3.84707067	2363434623
cg02184697	0.02635537	7.1719977	1.26049749	40.807341
cg09427374	0.02635852	2661.66775	2.5258603	2804777.12
cg00072369	0.02636409	6127840847	14.1109682	2.6611E+18
cg26713775	0.02636633	19.634045	1.41857394	271.748772
cg27071152	0.02637256	3.8126403	1.1701414	12.4226234
cg02718725	0.02637423	47.4009176	1.57299735	1428.38574
cg05569124	0.02637848	349705054	10.0639765	1.2152E+16
cg26831488	0.02637887	5238.59248	2.73220741	10044205

cg16411949	0.02638349	566416.359	4.73231383	6.7795E+10
cg26502485	0.026384	1465.08756	2.35219436	912544.304
cg02549819	0.02638516	16.935807	1.39372202	205.795386
cg27172150	0.0263877	16.0965595	1.38537836	187.024163
cg24441975	0.02639091	25277.5829	3.28417158	194556278
cg14572307	0.02639512	161.953187	1.81593085	14443.7411
cg21653132	0.02640286	26.4196837	1.46787013	475.518696
cg17367077	0.02640577	7.84765282	1.27312773	48.3735084
cg14205016	0.02640887	10.9847259	1.3242545	91.1185907
cg18934106	0.02641322	16.6654735	1.39044968	199.746897
cg09747445	0.0264154	7.64238744	1.2690332	46.0240802
cg21488617	0.02641848	13.100573	1.35167976	126.971653
cg08066035	0.02641848	5.49082602	1.22078015	24.696642
cg02384546	0.02642935	4.30983268	1.18652563	15.6546621
cg25706502	0.02643118	0.14896615	0.02773155	0.80020469
cg01319727	0.02643469	162865.291	4.07373376	6511250968
cg14247055	0.02643522	253686.967	4.29044778	1.5E+10
cg02435083	0.02643607	0.10402459	0.01410258	0.76731463
cg18273421	0.02643761	13.9068195	1.36076756	142.125395
cg02219997	0.026458	64.7070214	1.62819598	2571.55691
cg02207219	0.02646744	12.3940145	1.34196121	114.467984
cg20902958	0.02647161	61.5361579	1.61812914	2340.1709
cg08948823	0.02647269	36174.3158	3.40801447	383971703
cg22434674	0.02647911	538.995002	2.08446431	139371.834
cg12656692	0.02648358	1083107.88	5.06490644	2.3162E+11
cg06286401	0.02648382	83211.5876	3.75356216	1844692596
cg08790004	0.02648465	709.145184	2.15187271	233697.323
cg16071118	0.02648619	61.6801945	1.61800215	2351.32346
cg25643782	0.02648724	9109.74159	2.89869657	28629209.6
cg03151810	0.02650093	0.30107446	0.10427057	0.86933285

cg00079563	0.02650248	61.6595919	1.61730384	2350.76748
cg16327839	0.02650277	0.22847456	0.06201002	0.84180948
cg08132177	0.02651594	578151.804	4.69515656	7.1192E+10
cg01133446	0.02652252	9.2313479	1.29562289	65.7736019
cg08230658	0.02652944	36.9992166	1.52290199	898.903566
cg24495257	0.02653836	262610403	9.55630118	7.2166E+15
cg24844545	0.0265501	12.1719266	1.33751207	110.769691
cg24956391	0.02655075	0.27605113	0.08851689	0.86090045
cg24389730	0.02655083	3.95814582	1.17361191	13.3493178
cg16509443	0.02655515	27115.4689	3.27899052	224230186
cg15038123	0.02656112	631.010505	2.11663364	188116.758
cg11201447	0.02656257	6.07898691	1.23354025	29.9577432
cg25129124	0.02656329	5.56988236	1.22104821	25.407342
cg03502630	0.02657093	8.82045309	1.28797343	60.4052775
cg03317402	0.02659368	1908.63832	2.40395231	1515379.58
cg03939371	0.0265937	2813.76834	2.51476611	3148321.52
cg18207091	0.02660338	1552807.05	5.2300067	4.6103E+11
cg14460968	0.02660468	1958547.32	5.37222945	7.1403E+11
cg16520101	0.02660531	3.6711E+15	63.9994567	2.11E+29
cg11274211	0.02660782	29879.1718	3.30572851	270066010
cg04831490	0.02660794	5312.81134	2.70544389	10433025.2
cg15831613	0.02660976	37.9299673	1.52471265	943.576104
cg21786349	0.02661706	6677.07695	2.77684808	16055382
cg12120360	0.02661958	0.1265822	0.02036261	0.78688615
cg19847514	0.02662508	106741610	8.52513561	1.3365E+15
cg22469141	0.02662765	15287512	6.80379386	3.435E+13
cg11052143	0.0266359	4.75562243	1.19801961	18.8777751
cg21492378	0.02663633	0.02473561	0.00093928	0.65140042
cg25513776	0.02663804	9.27339491	1.29436277	66.4387565
cg01939117	0.02663876	4.85119575	1.200753	19.5994516

cg18604823	0.02663909	6.48595257	1.24183353	33.8753786
cg26392737	0.02664189	0.10578574	0.01451623	0.77090414
cg14117138	0.02664974	5.35660472	1.21448787	23.6257725
cg19459207	0.02664976	9.28551802	1.29436147	66.612648
cg17809945	0.02665106	5.06770397	1.20670161	21.2824971
cg18015985	0.02665647	18298.1615	3.11420941	107514515
cg15574263	0.02666688	93.2414079	1.68985995	5144.78146
cg26779912	0.02666721	1144570.94	5.02194449	2.6086E+11
cg00924278	0.02667105	743.108329	2.14814143	257064.075
cg08222513	0.02667532	0.27234245	0.0862086	0.86035978
cg24830559	0.02667668	1581.17646	2.34361119	1066780.62
cg22125112	0.02667862	5.50806261	1.21806491	24.9073373
cg24847572	0.02668234	240878.792	4.18888789	1.3852E+10
cg11131549	0.02668321	28.8479637	1.47493139	564.23303
cg05950943	0.02669074	22.8850295	1.435781	364.766336
cg01569295	0.02669185	4.38994272	1.18639204	16.2438692
cg14996263	0.02669721	4.72353993	1.19641615	18.648887
cg20159072	0.02670811	1921.80525	2.3937631	1542899.3
cg14042912	0.02671249	162.54919	1.79964232	14681.9393
cg06941335	0.02671376	6.33720544	1.23750522	32.452528
cg06288570	0.02671466	34.8512692	1.50653562	806.227843
cg07657064	0.02671747	7.91788008	1.2696648	49.3774616
cg07648738	0.0267175	84399197.3	8.21497814	8.671E+14
cg16576694	0.02671904	0.22120574	0.05823576	0.84023945
cg13808674	0.02672053	9.81899392	1.30153347	74.0761908
cg13678049	0.02672495	17.4303757	1.39052358	218.491798
cg09371063	0.02672703	588.995876	2.086818	166241.686
cg00938535	0.02672866	313.753107	1.94050415	50729.6067
cg04051396	0.02672963	8.44549043	1.27895755	55.7690976
cg05861454	0.02673161	7218.50133	2.78549496	18706464.1

cg00700007	0.02674373	3.19722996	1.14332021	8.94087177
cg17684754	0.02674696	55.1989241	1.58744804	1919.38328
cg00427553	0.02674832	0.00022207	1.30E-07	0.37938786
cg07525751	0.02674859	0.16629117	0.03400164	0.81327707
cg10934807	0.02675103	0.17428314	0.03714606	0.81770743
cg07794237	0.02675387	525133.331	4.55869424	6.0492E+10
cg17282836	0.02675402	10.9331057	1.31715801	90.7505392
cg25303383	0.02675668	14.882048	1.36472123	162.286148
cg13175159	0.02676218	11.3818251	1.3231214	97.9093394
cg15444076	0.02676633	55.8384024	1.58883534	1962.39793
cg26619624	0.02676713	42.2641721	1.53867993	1160.90437
cg00498024	0.02677508	5.67955595	1.22119822	26.4145128
cg25471923	0.02678947	6.64852705	1.24333511	35.5518889
cg03787837	0.02679018	4.83417356	1.1985971	19.4971555
cg06208294	0.02679171	694169.562	4.69367804	1.0266E+11
cg00444390	0.02679525	0.19375475	0.04533409	0.82809442
cg12066473	0.02680523	11.0256956	1.31749559	92.2704896
cg06096184	0.02680958	6.19173754	1.23293887	31.0944968
cg01447579	0.02681018	231.896631	1.86918147	28769.8376
cg05666074	0.02681166	4827197281	12.9442799	1.8002E+18
cg26333638	0.02681452	2915.48433	2.49937111	3400875.06
cg16855845	0.02681571	104.010317	1.70448871	6346.85696
cg17121747	0.02682626	3.15674921	1.14101271	8.7335272
cg20001850	0.02682717	3395323077	12.4070818	9.2916E+17
cg08473764	0.02682987	4.71147864	1.19463246	18.5814732
cg14353516	0.02683828	6433.91249	2.73386428	15141655.1
cg27109600	0.02684064	6.4811933	1.23900758	33.9028328
cg04913657	0.02684381	800687842	10.4908941	6.111E+16
cg05863499	0.0268477	61.9906305	1.60492876	2394.39803
cg01024069	0.02685919	6187.13462	2.71874371	14080266.1

cg09526758	0.02685977	5.25509671	1.20934499	22.8355363
cg06449094	0.02687255	0.00488518	4.39E-05	0.54376121
cg27101112	0.02687385	723.921704	2.12507548	246608.951
cg11146971	0.02687918	4.52346167	1.18854956	17.215694
cg22857553	0.02688229	1181888.49	4.95317979	2.8201E+11
cg08288330	0.02689581	32336.5334	3.27860841	318931468
cg05155784	0.02690183	9.42622125	1.29235785	68.7531301
cg14862722	0.02690669	0.17163729	0.03603281	0.81757035
cg21907914	0.02691172	6.43320346	1.23700125	33.4568026
cg05423529	0.02691673	7.89894605	1.26627861	49.2730022
cg25582283	0.02691882	15.2226193	1.36477594	169.792075
cg26384310	0.02691886	7434555697	13.4113011	4.1213E+18
cg17550268	0.02691965	6145124743	13.1213304	2.878E+18
cg10112854	0.02692545	7.75664694	1.26352088	47.6173942
cg22868409	0.02692983	22213.8169	3.13461433	157420853
cg09677945	0.02693042	4.71569545	1.19367368	18.6297009
cg26480413	0.02693085	2083.46283	2.39239476	1814423.54
cg14042889	0.0269417	5.32120259	1.21011566	23.3987526
cg07924644	0.02694512	0.02343523	0.00084271	0.65171652
cg04141094	0.02694831	4119.11755	2.58378738	6566766.87
cg04785418	0.02694843	6.2954E+10	17.0445617	2.33E+20
cg22588640	0.02694888	48.0496715	1.55519833	1484.55081
cg11099930	0.0269555	226643.732	4.07883524	1.2594E+10
cg01506678	0.02695609	0.07216857	0.00702824	0.74105394
cg03719155	0.02695663	10.0622454	1.30108519	77.8187185
cg16260298	0.02696572	44.6267951	1.54157454	1291.89396
cg21367838	0.02697954	7288.84849	2.75311519	19297162.9
cg26660414	0.02698546	6.68485892	1.24142756	35.9967349
cg08345979	0.02699993	916765.854	4.76641764	1.7633E+11
cg06033764	0.02700383	0.10320597	0.01379037	0.77238465

cg11123303	0.02700402	10.4978868	1.30654276	84.3490391
cg10729312	0.02700913	0.11710966	0.01750174	0.78361794
cg20431441	0.02701078	20.3941201	1.40886906	295.215607
cg17202331	0.02701641	114.933034	1.71464345	7703.99364
cg10534923	0.02702195	82.4094524	1.65079565	4113.96641
cg18827378	0.02703003	11930127.3	6.36381365	2.2365E+13
cg18021286	0.02703714	8.49275098	1.27490189	56.5744076
cg05529506	0.02703976	6.5379679	1.23756075	34.5397381
cg10537421	0.02704488	9.25815717	1.28732495	66.5826251
cg05740582	0.02705568	0.23310521	0.06409722	0.84774404
cg20207890	0.02705592	50.2042112	1.5592064	1616.50364
cg24237813	0.02707481	245.56089	1.86568611	32320.6302
cg22007666	0.02707623	3.50540082	1.15271503	10.6599069
cg07049592	0.02707764	9.54445393	1.29122969	70.5502682
cg24698371	0.02707795	6926019.15	5.95660244	8.0532E+12
cg21916898	0.02707864	0.02202176	0.00074721	0.64902085
cg09010837	0.02707903	6473901.65	5.91063518	7.0908E+12
cg15842430	0.02707998	0.04923133	0.00340904	0.71097073
cg10248552	0.02708179	25.3705771	1.44235978	446.259105
cg00244776	0.02709981	18.0600799	1.38746573	235.08075
cg18728839	0.02710248	8.2452E+12	28.9413816	2.35E+24
cg27522780	0.02710268	61.4572029	1.59361018	2370.08261
cg01591227	0.02710444	310.27774	1.91391787	50301.1532
cg21788473	0.02710471	13304.5599	2.92815448	60451494.5
cg00003202	0.02710797	4.5691E+14	45.535799	4.58E+27
cg07654982	0.02711189	13.1255588	1.3380078	128.758812
cg18164983	0.02711717	180863.331	3.93046154	8322570775
cg05748497	0.02712006	309.356523	1.91228347	50045.6442
cg02954903	0.0271269	8.41809018	1.27221643	55.701405
cg01587630	0.02714662	164.531914	1.7791479	15215.5708

cg24873171	0.02717912	8.99409445	1.28091889	63.1528941
cg19068479	0.02718168	0.03135285	0.00145217	0.67691785
cg07911673	0.0271859	58.3013346	1.58103856	2149.88154
cg18923472	0.02718766	0.11934416	0.01809721	0.78702904
cg18732172	0.02719043	5.09813012	1.20139792	21.6339069
cg11357542	0.02719482	20.6806315	1.40656372	304.066226
cg22973515	0.02719819	82.8091856	1.64428712	4170.41593
cg05984388	0.02719989	2363.07734	2.39786083	2328798.41
cg01317270	0.02720295	9668.23742	2.80960137	33269778.4
cg08378932	0.02720376	6.26609177	1.22946747	31.9357015
cg17822007	0.02720406	128.025358	1.7266819	9492.4793
cg26812418	0.02720483	390.895846	1.95780495	78046.3663
cg14462067	0.02722461	207.372143	1.82186118	23603.997
cg08900316	0.0272251	0.23488079	0.06492927	0.84967823
cg00930347	0.02722568	25256189.5	6.79735688	9.3842E+13
cg12802310	0.02722916	102.642626	1.68313185	6259.46727
cg24102622	0.02723051	6.26011514	1.22898735	31.8872621
cg06116236	0.02723474	0.16504847	0.0333546	0.81670886
cg21232956	0.02723813	58569.5775	3.43357102	999075130
cg16584020	0.02724085	2931.19442	2.45210746	3503884.26
cg15450139	0.02725154	4.1253695	1.17249855	14.514878
cg27640988	0.02726359	34.0678472	1.48581752	781.131057
cg26353176	0.02726654	6.241645	1.22811188	31.7219733
cg10554436	0.02726737	7.90247816	1.26104573	49.5217259
cg10012394	0.02727247	66.5612193	1.60145775	2766.47693
cg02565308	0.02727525	2428231.82	5.20178413	1.1335E+12
cg07921731	0.02728068	3.8241E+12	25.7525511	5.68E+23
cg15014976	0.02728388	0.21240746	0.05367455	0.8405647
cg01348004	0.02728558	8.83670852	1.27666331	61.1652399
cg16756022	0.02729075	29.3660683	1.46048593	590.465099

cg13721644	0.02729942	21.5567561	1.41053656	329.444658
cg05493583	0.02730284	0.17078846	0.03555329	0.82042185
cg19230459	0.02731198	21631.0269	3.05696105	153060937
cg22338443	0.0273144	9.41951611	1.28535756	69.0292621
cg01883217	0.02732016	21.8750014	1.41233356	338.812088
cg18676313	0.02732044	21148.3641	3.04776853	146747792
cg02297043	0.02732866	9.34406594	1.2839654	68.0014963
cg27223183	0.02733606	588072.697	4.41629851	7.8308E+10
cg19718306	0.02734023	3.98100204	1.16699364	13.5805173
cg02699635	0.027347	177.251159	1.78346566	17616.2479
cg23378365	0.02734893	10.8557789	1.30531903	90.2828607
cg12351660	0.02735164	8.60934155	1.27189604	58.2758022
cg22777062	0.02735736	13.5966193	1.33839924	138.126242
cg16601489	0.02735771	0.1677431	0.03434649	0.81923223
cg15807973	0.02735839	4.8167E+11	20.1698843	1.15E+22
cg08425070	0.02736563	10.3238168	1.29772038	82.1295519
cg22076311	0.02737736	21.1495932	1.40560106	318.230616
cg20908260	0.02738043	0.04303194	0.00263017	0.70404124
cg11062635	0.0273854	35112099.6	6.94203399	1.7759E+14
cg11104313	0.02738782	18.5804573	1.38519345	249.231176
cg11010575	0.02739227	5.45675221	1.20824641	24.6440994
cg03199124	0.02739292	63.5857105	1.58868168	2544.96708
cg21151061	0.02739578	9.49367101	1.28513101	70.1327635
cg13851767	0.0274003	7.83669371	1.25787985	48.8232388
cg05774699	0.0274028	7.13804387	1.24482337	40.9308433
cg13714586	0.02740523	3.7269E+12	25.1507849	5.52E+23
cg21000493	0.02741477	1046773.69	4.68091898	2.3409E+11
cg26847756	0.02741584	499.414803	1.99740589	124869.536
cg25067702	0.0274165	7.04874044	1.24288707	39.9752663
cg09760422	0.02741779	8.54952725	1.26986858	57.5606148

cg05159804	0.02743631	7.60295489	1.25311614	46.1289432
cg00062312	0.02743884	2.08E-10	5.16E-19	0.08379184
cg24683695	0.02744352	0.01999209	0.00061751	0.6472551
cg03719380	0.02745103	47.5843842	1.53617905	1473.96465
cg27102332	0.02745463	208381414	8.40287284	5.1676E+15
cg08756266	0.02746132	16.3590258	1.36405349	196.192985
cg10637512	0.02746522	6.88369376	1.2389433	38.2464959
cg07960806	0.02746533	4.4260657	1.17963766	16.6068431
cg05651995	0.02746581	39.4233349	1.50391956	1033.43249
cg05072819	0.02747083	45265.3598	3.28802973	623155192
cg15556748	0.02747631	164.407545	1.76184201	15341.8073
cg00243313	0.02747662	5.1103912	1.19849725	21.7907034
cg09850289	0.02747763	126126.006	3.6825601	4319758282
cg07469949	0.02748198	3970325.41	5.39826111	2.9201E+12
cg24425342	0.02748634	0.00529995	5.02E-05	0.55914642
cg26423145	0.0274878	5.34076521	1.20424786	23.6859652
cg14729962	0.02748942	5.97354318	1.21927936	29.2658263
cg01726393	0.02749268	14.9474114	1.34979004	165.525823
cg27131891	0.02749639	0.28287698	0.09204593	0.86934195
cg08386886	0.02750317	3902.49111	2.50083285	6089746.01
cg15324310	0.02750661	697786855	9.55242493	5.0972E+16
cg17916916	0.02750784	5.39724621	1.20542024	24.1660673
cg22140675	0.0275102	223467.654	3.91490743	1.2756E+10
cg04710768	0.02751601	7.63757561	1.2525836	46.5697949
cg00951770	0.0275162	13.1220485	1.3299706	129.467641
cg09026415	0.02754084	76547.5161	3.46982042	1688710513
cg10481660	0.02754226	3.68575266	1.15523381	11.7593275
cg14676702	0.02757063	18.2353707	1.3780944	241.296057
cg14254668	0.02757316	1741.65853	2.28005638	1330394.48
cg18032014	0.0275819	4.85677939	1.19060804	19.8119827

cg27274426	0.02758295	0.19199066	0.04422599	0.83345594
cg26233374	0.02758297	15.1221223	1.3496311	169.437842
cg09769410	0.0275839	188610026	8.19367722	4.3416E+15
cg12195820	0.02758456	11829.3086	2.81558521	49699274
cg09466397	0.02758795	59.0300823	1.56839203	2221.73446
cg06566039	0.02758894	4.54E-09	1.72E-16	0.12003945
cg09493652	0.02759429	3074.91875	2.42542935	3898330.55
cg10507121	0.02759483	1638697.86	4.84823496	5.5388E+11
cg12129344	0.0275955	19.1422955	1.38491988	264.583879
cg11809091	0.02759761	7.68657995	1.25227581	47.1809093
cg03243768	0.02760247	10.0841029	1.29026025	78.8128833
cg04492263	0.02760703	6.9112481	1.23754557	38.5968416
cg22658758	0.02760748	10.1140239	1.29059625	79.2606352
cg20986280	0.02761097	82.6452601	1.62678522	4198.61142
cg23737575	0.02761327	0.16908236	0.03477533	0.82210129
cg10674754	0.02762044	1384064.26	4.74888357	4.0339E+11
cg15370732	0.02763152	82.3246683	1.62524461	4170.04982
cg12976581	0.0276348	8.85759045	1.27142571	61.7078196
cg24949907	0.02763755	0.17826429	0.03842095	0.82710504
cg17432857	0.02763962	5.18936588	1.19869294	22.4657352
cg24837744	0.02764058	6765443	5.64584019	8.1071E+12
cg09313258	0.02764172	132.851368	1.71269286	10305.1087
cg13949137	0.02764557	9.7866629	1.28528359	74.5195622
cg25379116	0.02765685	8658.10121	2.71005437	27660964.1
cg09172260	0.02765717	11.8465545	1.31236729	106.937178
cg22361075	0.02766166	6396.01513	2.62066458	15610166.2
cg11540692	0.02766338	9.10326804	1.27480026	65.0058612
cg18962747	0.02767381	70119.2102	3.40722946	1443020992
cg20893898	0.02767583	0.07150582	0.00683189	0.74841459
cg17363387	0.02767787	0.09710882	0.0121832	0.77402654

cg24400608	0.02768276	853.510443	2.09843472	347154.033
cg24630566	0.02770826	802.114805	2.08213912	309003.445
cg07982208	0.0277111	221.559148	1.80797984	27150.9976
cg13975172	0.02771159	231.799384	1.81693175	29572.3571
cg24687970	0.0277182	4.98757693	1.1926099	20.858391
cg02735652	0.02771827	70.360738	1.59401514	3105.76313
cg19402405	0.02772051	76741047.2	7.31483676	8.051E+14
cg00601350	0.02772573	5.20826263	1.19819824	22.6389913
cg18501647	0.02773033	20.3296067	1.39090821	297.138879
cg05639912	0.02773165	5.30758447	1.2006131	23.4633896
cg23120805	0.02773523	1.3337E+16	58.3392405	3.05E+30
cg02631680	0.02774401	22502.5408	2.99518088	169059688
cg02311432	0.02774566	15.313275	1.34807467	173.949111
cg08366813	0.02774915	618.901879	2.02072304	189555.683
cg11516606	0.02775379	14779.7592	2.85894325	76406302.3
cg08596000	0.02775741	118.390388	1.68577545	8314.44304
cg05481243	0.02775772	138.974523	1.71558252	11257.9359
cg07722220	0.02776071	84.4169689	1.62440957	4386.96298
cg00966077	0.02777741	12533.4202	2.80426584	56017021.9
cg15768282	0.0277846	59851804.4	7.07172793	5.0656E+14
cg01157467	0.02778491	146.958247	1.72475965	12521.5861
cg00567190	0.02778623	23.5439883	1.41202996	392.569139
cg26929355	0.02778696	29.216205	1.44569607	590.432978
cg16490176	0.02778871	36.3752738	1.48066678	893.624796
cg14066993	0.02779038	28.9891178	1.44436952	581.824069
cg10600917	0.02779324	107.458784	1.66640903	6929.50537
cg12397426	0.02780126	6.46503657	1.22592882	34.0939026
cg15102606	0.02780294	1030097238	9.62884797	1.102E+17
cg06376277	0.0278174	0.03057482	0.0013674	0.68364872
cg13202410	0.02782112	7.86901256	1.25221434	49.4494886

cg01208515	0.02782131	119.464876	1.68450074	8472.45497
cg06424168	0.0278279	7.67598863	1.24872986	47.1845858
cg10860308	0.02783353	0.12495777	0.01958569	0.79723727
cg04066177	0.02784256	6.31302374	1.22221862	32.6081341
cg05805038	0.0278461	72.0447764	1.59317102	3257.93637
cg01375976	0.02785188	16.5376532	1.35716193	201.519044
cg02182440	0.02785219	5.73026481	1.20927879	27.1533206
cg26343133	0.02785268	4.36758817	1.17405323	16.2478379
cg11585745	0.02786535	30.068245	1.44803624	624.36238
cg00594003	0.02787095	2570.89385	2.34865369	2814163.37
cg20246548	0.02787503	12499.2263	2.78873709	56022010.1
cg10305299	0.02788776	0.21725089	0.0557134	0.84715617
cg13118072	0.02788797	376084413	8.54398795	1.6554E+16
cg17390562	0.0278898	3480.1132	2.42515246	4993990.29
cg05927190	0.02789277	3.90559559	1.15949156	13.1554877
cg13665981	0.02789834	6.62989187	1.2280157	35.793896
cg17081801	0.02790013	20398807.3	6.21791485	6.6921E+13
cg25363886	0.02790222	32022737.9	6.52859929	1.5707E+14
cg06391932	0.02790376	9.74751148	1.28041061	74.2058676
cg12441126	0.02790655	3.49039446	1.14531004	10.6371664
cg08554302	0.0279098	84423.9629	3.42469999	2081176613
cg07195622	0.02791419	2117047.74	4.85638565	9.2289E+11
cg14867604	0.027915	91.0762299	1.63146418	5084.31614
cg26497348	0.02791811	6.07472564	1.2161625	30.3432244
cg08963608	0.02791899	149480224	7.703386	2.9006E+15
cg06528626	0.02792329	5.1717301	1.19505877	22.3811522
cg24166172	0.02793507	531317.434	4.17343873	6.7642E+10
cg16621987	0.02793676	251.434889	1.82023192	34731.5651
cg00794552	0.02793778	2138758.46	4.85225167	9.4271E+11
cg19292008	0.02793849	5.94866553	1.21314815	29.16925

cg22876908	0.02794224	25.8893826	1.42262863	471.142023
cg10002977	0.02795827	13443.9994	2.79827548	64590181.5
cg11984608	0.02796482	6.29689827	1.22031311	32.4924215
cg00783905	0.02796595	1968377.97	4.79763549	8.0759E+11
cg01046376	0.02796737	0.02071938	0.00065299	0.65742209
cg23231666	0.02797176	1.57E-06	1.04E-11	0.2355607
cg25397562	0.02797387	3.41E-10	1.23E-18	0.09464497
cg07167398	0.02797993	2.2143E+10	13.1382848	3.73E+19
cg21210531	0.02798022	3.53667159	1.14634098	10.9112788
cg11078326	0.02798472	0.15497247	0.02937905	0.81746923
cg10705379	0.02798695	5.51559198	1.20268596	25.294845
cg03460527	0.02798752	68.3817638	1.57876221	2961.85555
cg09070503	0.02798969	3.0084E+12	22.3089958	4.06E+23
cg02486145	0.02799253	5.13720381	1.19342108	22.1136222
cg21431125	0.02799435	12992981.9	5.86893273	2.8765E+13
cg02644867	0.02799847	6.16934363	1.21718959	31.2694103
cg08939008	0.02800747	19589.2576	2.90660305	132023192
cg23975712	0.0280121	121580.012	3.53876004	4177084411
cg16782084	0.02801361	6.8159167	1.23016109	37.7647453
cg19109608	0.02801457	7.54998145	1.24380301	45.8289775
cg12395726	0.02801482	1532.1991	2.20682578	1063805.8
cg01652532	0.02801606	2613.12268	2.33757365	2921152.95
cg22123255	0.02801661	3.4484E+12	22.5406601	5.28E+23
cg15380914	0.02802324	9092164.46	5.63203704	1.4678E+13
cg01289902	0.02804563	30206.9823	3.03893078	300257507
cg07568203	0.02804987	0.14527091	0.02597828	0.81235706
cg07307426	0.02805269	768.195267	2.04544118	288506.936
cg17867333	0.02806529	0.23054053	0.06224233	0.85390339
cg10361095	0.02806716	9.330578	1.2717059	68.4589774
cg25401140	0.02807787	5.7319E+12	23.5689059	1.39E+24

cg24287239	0.02808235	5.6088999	1.20374246	26.1349575
cg04802826	0.02808814	1148.53466	2.13296751	618449.115
cg19698993	0.02808935	7.50856476	1.24200029	45.3933425
cg13971154	0.02808949	3.03509396	1.12676346	8.17544738
cg17327184	0.02809038	20.7341265	1.38527186	310.339086
cg07093915	0.02809232	1518.35737	2.1975649	1049074.41
cg18011163	0.02809339	7.24773345	1.23723255	42.4573698
cg00255925	0.02809612	35158794.6	6.46997956	1.9106E+14
cg20656621	0.02810372	18393197.3	6.03048824	5.61E+13
cg18613421	0.02810815	11.1897065	1.29609032	96.60556
cg14331899	0.02810942	6.67217705	1.22606704	36.3095532
cg01573194	0.02811083	8.1109187	1.25202643	52.5444196
cg10008501	0.02811824	6.09150315	1.2140277	30.5647151
cg19979540	0.02811824	121396974	7.37428962	1.9985E+15
cg19942640	0.02811846	8148233.58	5.51813194	1.2032E+13
cg04067276	0.02811909	10.901757	1.29227494	91.9682823
cg25316172	0.02811978	16.9063818	1.35457289	211.008022
cg15169829	0.02812106	7.31742192	1.23811856	43.2467983
cg16000989	0.02812627	0.01671756	0.00043349	0.64471062
cg07595113	0.0281279	1.254E+11	15.5106499	1.01E+21
cg19974445	0.02812834	1053124466	9.28779769	1.1941E+17
cg19713083	0.02812889	313513.127	3.88695673	2.5287E+10
cg09965384	0.02813243	5.11631009	1.19134893	21.9722604
cg03019033	0.02813299	85.5526862	1.61151762	4541.8443
cg14547335	0.02813366	0.16255904	0.03210994	0.82296756
cg01398529	0.02813542	1898106.83	4.71273599	7.6448E+11
cg06479604	0.02813562	5.04466818	1.18951356	21.3941882
cg15647311	0.02813579	26.0454162	1.41845928	478.239819
cg10207240	0.02813853	9.72654123	1.27623011	74.1289546
cg22397447	0.02814343	7.20E-08	3.02E-14	0.17154023

cg26299549	0.02814388	3223335.2	4.98452547	2.0844E+12
cg26394572	0.02814631	21146.6331	2.90778302	153787297
cg02580197	0.02814873	5.8106283	1.20752419	27.9608487
cg27665171	0.02815271	126767.478	3.52155597	4563321870
cg03535361	0.02816571	4150031145	10.709407	1.6082E+18
cg08781253	0.02816677	2122859.06	4.75729156	9.4729E+11
cg18648378	0.0281682	55.730684	1.5378805	2019.60369
cg09835085	0.028173	5.6616711	1.20388109	26.6259849
cg07560446	0.02817349	0.16884806	0.03448791	0.82665679
cg17778955	0.02817416	35.6551774	1.4659025	867.241631
cg23994468	0.02817947	33.978754	1.45821215	791.761144
cg14033806	0.02818668	17.8529812	1.36102701	234.182669
cg22817638	0.02819219	9.38028322	1.27041389	69.2606669
cg27365825	0.02819664	4.41826406	1.17211802	16.6545152
cg11915525	0.02819669	13.5908366	1.32170047	139.752421
cg01564135	0.02819733	4.90142418	1.18518529	20.2702136
cg10771090	0.02819942	5.8394186	1.20755133	28.2379794
cg08576623	0.02820529	7.95664694	1.24805991	50.7253138
cg02517558	0.02820599	740739.625	4.23741502	1.2949E+11
cg00667974	0.02820742	3801031366	10.5539745	1.3689E+18
cg22356722	0.0282097	7.42889487	1.23887965	44.5470865
cg03123289	0.02820973	10.9634864	1.29146776	93.0708736
cg10442148	0.02821012	2274940.39	4.77545238	1.0837E+12
cg06974034	0.02822656	5.50120055	1.19956118	25.2285652
cg23921854	0.02823235	49831.6457	3.17079829	783144395
cg26396492	0.02823244	9.94769016	1.27774655	77.4461411
cg14638883	0.02825368	4.3638984	1.17001064	16.2764411
cg14283447	0.02825543	7.13947996	1.23300441	41.3398148
cg09432138	0.02826084	808345.773	4.25910181	1.5342E+11
cg10976172	0.02826098	20.1686691	1.37715309	295.373997

cg25446086	0.02826118	22.6721471	1.3944208	368.630655
cg12387009	0.02826121	8.0832E+11	18.5546828	3.52E+22
cg25037730	0.02827333	9.4800811	1.27053824	70.7353267
cg09714181	0.02827485	2198.95543	2.26866864	2131384.41
cg05341353	0.02828314	510.638714	1.94153528	134301.91
cg02215430	0.02828428	4.73221225	1.17983954	18.9804053
cg03718284	0.02828591	1315.07501	2.14688959	805547.849
cg13188519	0.02828972	822.336324	2.04199568	331164.77
cg12749531	0.02829322	2.1296E+11	16.021147	2.83E+21
cg14323910	0.02829651	0.3646403	0.14801785	0.89828726
cg19259199	0.02830432	143.16471	1.6948224	12093.3817
cg12235877	0.0283074	25.4417767	1.41045201	458.919551
cg10132588	0.02831835	7.67570274	1.24165485	47.4499115
cg11103652	0.02832163	9750319.9	5.52223219	1.7216E+13
cg25292098	0.02832918	89.2640475	1.61081387	4946.61135
cg00042356	0.02833083	1.0993E+12	18.9642528	6.37E+22
cg12073754	0.02833226	9637847.49	5.51010386	1.6858E+13
cg07701282	0.02833462	110.956223	1.6482007	7469.5293
cg06348477	0.02833556	1.31E-06	7.25E-12	0.23763294
cg14719951	0.0283363	3.58463564	1.14505572	11.2218231
cg04374102	0.02833965	10571.3074	2.67229222	41818981.6
cg01302668	0.02834112	9.97235538	1.27627791	77.9202328
cg08094235	0.02834195	4.30504467	1.16746746	15.8748832
cg25387636	0.02834506	4.64310086	1.17683436	18.3189634
cg00086730	0.02835232	19988.1504	2.85708206	139837131
cg00138497	0.0283534	210.340948	1.76294096	25096.3108
cg09547173	0.02835403	17.9129458	1.3577989	236.318962
cg19578881	0.02835584	4512904323	10.5504704	1.9304E+18
cg04195863	0.02835839	0.11331273	0.01617262	0.79392049
cg13175480	0.02836484	47161.5054	3.12689826	711314347

cg04273871	0.02836618	11.5645259	1.29603987	103.189927
cg03207666	0.02836743	10.7061414	1.28547289	89.1667689
cg03586793	0.02836882	7.44831026	1.23698678	44.8487619
cg16758662	0.02837567	7.37156988	1.23553514	43.980977
cg16762077	0.02837867	11.4989412	1.29503597	102.101911
cg18498156	0.0283787	28.8458772	1.42746353	582.911307
cg01957900	0.02839838	0.0910316	0.01067699	0.77613155
cg00588720	0.02840649	4718.26998	2.44529668	9104037.08
cg12379940	0.0284201	5.00973618	1.18554783	21.1695015
cg27117639	0.02842265	8.29E-10	6.25E-18	0.10986931
cg08257866	0.02842742	56.038194	1.5297281	2052.83487
cg25426743	0.02843719	5.29397942	1.19228706	23.506267
cg22605924	0.02844487	11.9858916	1.29952748	110.549103
cg03615240	0.02844545	221796.13	3.66361735	1.3428E+10
cg08272268	0.02844648	7.16391475	1.23083349	41.6966836
cg12018140	0.02846412	9.3430118	1.26551747	68.9772142
cg19628619	0.02846773	4.73990501	1.17814202	19.069602
cg21944443	0.02847014	20.3152364	1.37331922	300.519228
cg13919420	0.02847316	111.227672	1.64256255	7531.88665
cg15228639	0.02848011	9.42560841	1.26643558	70.1512935
cg16047144	0.02848159	3.85793732	1.1527398	12.9115698
cg00151788	0.02848409	1.0367E+12	18.4007095	5.84E+22
cg24364518	0.02849306	8.1181E+10	14.0552315	4.69E+20
cg13679837	0.02849501	1892085133	9.4617417	3.7836E+17
cg19574915	0.02849895	26.1038063	1.40932603	483.499691
cg25039393	0.0285095	58.2159169	1.5330111	2210.74263
cg02077702	0.02852097	18.3237844	1.35734261	247.366488
cg27598661	0.02852403	4.23E-13	3.56E-24	0.05014444
cg20446334	0.02852963	3.25327774	1.13187451	9.35069744
cg09667226	0.02853337	207882275	7.46911744	5.7858E+15

cg12934382	0.02853561	10.8255169	1.28407809	91.2653344
cg02661886	0.0285433	619.78533	1.96333806	195653.445
cg07882671	0.02855509	2.65917475	1.1080039	6.38193637
cg15773539	0.02855611	24.7297715	1.39988604	436.865272
cg07318128	0.02856162	23.8016754	1.39414507	406.356386
cg23065793	0.0285619	2538.29266	2.27451263	2832663.82
cg04332613	0.02856231	1.5709E+11	14.9154057	1.65E+21
cg01960526	0.02856668	3748152.44	4.8858208	2.8754E+12
cg24041453	0.02856737	19.8573541	1.36778369	288.287187
cg21635948	0.02856798	62715.5333	3.18222446	1236002726
cg12249789	0.02856925	6.34454513	1.21361271	33.1681208
cg09556286	0.02857663	10.5701823	1.28016722	87.2766871
cg22256433	0.02858797	4.67475997	1.17519696	18.5955049
cg09841537	0.02858848	50813.6456	3.10892523	830520641
cg26149645	0.0285989	125.66122	1.65812003	9523.28056
cg08931196	0.02860384	103423.841	3.3456799	3197105306
cg26228569	0.02860882	261330.972	3.68500334	1.8533E+10
cg23769143	0.0286152	25.7605697	1.40437507	472.528293
cg08331842	0.02861636	17.4431085	1.34826509	225.669297
cg09740133	0.02861717	0.0101979	0.00016794	0.6192377
cg00922748	0.02862221	3.87217778	1.15195123	13.0159683
cg22645427	0.02862495	4.52523699	1.17083678	17.4898587
cg18361024	0.02862542	251.943684	1.78181799	35624.0764
cg11929911	0.02863066	503551843	8.10667587	3.1278E+16
cg11876919	0.02863894	7680.46722	2.5445412	23182794.8
cg00484396	0.02864338	18.5219074	1.35614644	252.967557
cg12030690	0.02864594	6.51503641	1.21600586	34.9058347
cg08736918	0.02864757	2.21E-06	1.89E-11	0.25690973
cg12691330	0.02864818	5.37116934	1.19172642	24.2081233
cg09109520	0.02865717	8.5985548	1.25156162	59.0743144

cg09831010	0.02866455	1288.78277	2.10976925	787271.421
cg16007090	0.028667	52183.5032	3.10267242	877668551
cg06381123	0.0286723	109.774894	1.63167181	7385.38678
cg25191743	0.0286761	2094771.07	4.55581148	9.6318E+11
cg05148250	0.02867958	601236.881	3.99921942	9.0389E+10
cg24656271	0.0286836	6.36950455	1.21266721	33.4556654
cg15651127	0.02868373	47.3132795	1.494293	1498.06391
cg18444267	0.02868493	14.1899581	1.31814022	152.756821
cg04852989	0.02868891	135.269558	1.66680596	10977.7945
cg19615059	0.02869041	16.7710893	1.3411646	209.720296
cg06625838	0.02869235	346.852075	1.83828235	65444.9857
cg04525491	0.02869307	37.1031936	1.45666256	945.069234
cg06927343	0.02869684	0.00103132	2.18E-06	0.48886516
cg01566404	0.02869998	19.0679982	1.35899056	267.543105
cg06939307	0.02870437	6.58875685	1.21668105	35.6804414
cg13577076	0.02870707	5.48187734	1.19359385	25.1768884
cg17138785	0.02871338	317948463	7.65657905	1.3203E+16
cg02012771	0.02871555	13131.896	2.68011861	64342933.5
cg26454350	0.02871755	6647.26693	2.4967252	17697645.6
cg18709306	0.02871996	22.4790016	1.38197884	365.639111
cg24664672	0.0287201	793.424009	2.00155468	314516.343
cg25382652	0.02872194	0.04743284	0.00308851	0.72846487
cg05741182	0.02872306	119500372	6.90887775	2.067E+15
cg25169875	0.02872404	412704.586	3.83311086	4.4435E+10
cg24403578	0.02873115	34.7804715	1.44579563	836.688926
cg12249809	0.02873141	25.6364422	1.40069544	469.214898
cg11832404	0.02873269	4.20731354	1.16094969	15.2474198
cg04660111	0.0287352	34.3293604	1.44372039	816.297252
cg24108579	0.02873814	4.79128131	1.17667229	19.5095752
cg06671340	0.02874233	548.878771	1.92483063	156516.579

cg06501716	0.02874692	24.3427875	1.3927947	425.454883
cg02748089	0.02874979	3.66363179	1.14424124	11.7302169
cg04784699	0.02875642	6.37878985	1.21193242	33.5736212
cg26605335	0.0287578	7.33623473	1.22962303	43.7697885
cg17334359	0.02876468	14.9016197	1.32327273	167.809904
cg09002922	0.02877019	7.40113726	1.23057565	44.5131779
cg22599959	0.02877727	25.0878227	1.39639308	450.731859
cg23411036	0.02877772	49.2493387	1.49746472	1619.7359
cg00909706	0.02877803	5.59294355	1.19526667	26.1707436
cg24623196	0.02878223	147371.478	3.43075564	6330486540
cg19483962	0.02879093	155488.369	3.44785064	7012088242
cg01441114	0.02879259	270316127	7.46376274	9.7901E+15
cg11698099	0.02879309	5.20585777	1.18625193	22.8458681
cg17176470	0.02879618	9.51964716	1.26269254	71.7701887
cg10553515	0.0288065	8405105.88	5.20430393	1.3574E+13
cg06291428	0.02880887	0.20026543	0.04736455	0.84675645
cg13719287	0.02881548	7.99377138	1.23978575	51.5414706
cg01791407	0.02882036	3.23048422	1.12887415	9.24463394
cg19137671	0.02882231	289491.738	3.66890977	2.2842E+10
cg18189994	0.02882598	4.91480232	1.17886119	20.4903529
cg18077580	0.02882702	5.5020275	1.19267993	25.3817524
cg14663177	0.02883522	5.77582696	1.19858386	27.8329937
cg13348059	0.02884703	4.18069516	1.15912082	15.0788526
cg22968727	0.02885082	1113.55984	2.06269003	601164.253
cg06288697	0.02885131	5.37933678	1.18963118	24.3245677
cg09713758	0.0288617	0.12005346	0.01793518	0.80360681
cg00356511	0.0288648	1.4212E+10	11.1430067	1.8126E+19
cg03699778	0.02886493	2.1264E+10	11.6155483	3.89E+19
cg12587087	0.02886542	9.41778663	1.26019003	70.3820081
cg14753355	0.0288669	7.31300363	1.22772348	43.5603156

cg14155825	0.02886696	27182.7523	2.86574329	257839571
cg22160883	0.02886756	37.6621701	1.45375904	975.704376
cg22716904	0.02886959	9.3191E+13	27.5569442	3.15E+26
cg07870237	0.02887393	6.63041657	1.21529079	36.1744073
cg26738040	0.02887503	75.551795	1.56166607	3655.11797
cg19306474	0.02887832	13338433.5	5.42305547	3.2807E+13
cg09155362	0.02887874	28.3636318	1.41157547	569.927453
cg16773162	0.02888587	8.60967928	1.24827738	59.3830976
cg08715146	0.0288891	8.83746741	1.25159041	62.4012692
cg03048947	0.02889934	5.97754134	1.20206924	29.7245778
cg15718572	0.02890259	4.96428596	1.17927112	20.8977686
cg07448499	0.02890337	1700.97846	2.1501624	1345632.18
cg18557263	0.02890751	3215364.7	4.67205986	2.2129E+12
cg02157015	0.02891027	9.07754813	1.25472217	65.6734075
cg14161426	0.02891921	5.5471E+10	12.7268013	2.42E+20
cg01501896	0.02891947	8.22854176	1.24197655	54.5170513
cg20964423	0.02892325	9.82245224	1.26473333	76.2853051
cg20786683	0.028925	2.09E+11	14.5742446	3.00E+21
cg14825683	0.02892742	13.4202386	1.30588826	137.915937
cg02286091	0.02893397	5.49696985	1.19134996	25.3633933
cg08007986	0.02893838	16.7721915	1.33592665	210.570247
cg24005667	0.02893931	0.18709078	0.04157875	0.84184728
cg05678729	0.02894374	2254953.2	4.49124048	1.1322E+12
cg06080402	0.02895787	13115747.6	5.37429721	3.2008E+13
cg25160978	0.02897169	5.37542578	1.18819473	24.3185748
cg10335067	0.02897234	87390.892	3.21084807	2378551658
cg26450387	0.0289731	35.681447	1.44263449	882.528228
cg05666120	0.02897474	4.46914295	1.16588303	17.1314258
cg14033514	0.02897812	5.39200725	1.18849807	24.4625911
cg23630954	0.02897972	0.07130886	0.00666531	0.76289863

cg07976603	0.02898567	668.541007	1.94730242	229521.143
cg15699552	0.02899574	0.07075087	0.00656513	0.76246543
cg23047804	0.02899644	116409.778	3.30144401	4104639170
cg26455413	0.02899761	13134.4707	2.64031137	65338627.2
cg09191574	0.02900808	12.7787404	1.29783866	125.821653
cg12275410	0.0290128	6.44345022	1.20996064	34.3135549
cg06986734	0.02903692	6.49479751	1.21063823	34.8431047
cg11416076	0.02903779	7.24783979	1.22427098	42.9081326
cg23039471	0.02903924	22.4284052	1.37399292	366.11059
cg00388812	0.02904197	12.7323434	1.29673117	125.016327
cg17817203	0.02904789	0.09376857	0.01119617	0.78531689
cg09078754	0.02905333	6.66489419	1.21362908	36.6016401
cg00334820	0.02905849	3.1676E+10	11.7902831	8.51E+19
cg18492804	0.02906242	104.34361	1.60666637	6776.50883
cg17405853	0.02909323	0.13909021	0.02365089	0.81798557
cg23205003	0.02909384	3248199123	9.30504704	1.1339E+18
cg16779976	0.02909786	7.55112772	1.22857574	46.4110822
cg07880636	0.02909804	0.01125692	0.0002001	0.63327357
cg10246448	0.02910097	16.2950685	1.32860032	199.856387
cg08545493	0.02910309	0.14898937	0.02694501	0.82381976
cg12628956	0.02911395	5330.89922	2.39408782	11870277.4
cg02970696	0.02911408	3.49044551	1.13560875	10.7283515
cg11176186	0.02911635	1026537.86	4.08768033	2.5779E+11
cg25337570	0.02912122	1951.91177	2.1608087	1763210.02
cg09189752	0.02912581	70.2725046	1.54084	3204.89143
cg26789332	0.02912669	20.0623831	1.35644841	296.730207
cg10936964	0.02913636	16.8186944	1.33214293	212.34094
cg27303326	0.02914876	2.99E-06	3.25E-11	0.27483277
cg21785847	0.02915397	32.0072651	1.42166788	720.607843
cg16458494	0.02915521	4.11587509	1.15443631	14.6741987

cg19833103	0.0291652	32.0808328	1.42169061	723.912659
cg04681368	0.02917109	126276.27	3.29116586	4844999335
cg03691812	0.029175	27184.2715	2.81584785	262437694
cg00486259	0.02917607	6.0934187	1.20108786	30.913435
cg01958916	0.02917902	59.4816928	1.51310966	2338.2785
cg21204804	0.02918999	5.87188744	1.19642241	28.818469
cg26015971	0.02919224	5.11797912	1.17985751	22.2007403
cg05301811	0.02920895	392.341336	1.8301601	84108.3378
cg27477205	0.02921443	2252230.01	4.3925392	1.1548E+12
cg06209740	0.02922527	4924.5306	2.36236229	10265572.6
cg04315434	0.029229	1495207232	8.46237549	2.6419E+17
cg07223632	0.0292291	170.020037	1.6806806	17199.4684
cg15467112	0.02923547	14.0965558	1.30654729	152.090083
cg15864491	0.02924476	202.339728	1.70971931	23946.2496
cg22928599	0.02924587	26.2876269	1.39121005	496.718182
cg16261871	0.02925342	7.5417601	1.22627114	46.3830091
cg19518452	0.0292593	211842102	6.9229662	6.4823E+15
cg24713667	0.02927347	34.1567325	1.42772084	817.16421
cg22188945	0.0292773	1481352941	8.4065733	2.6103E+17
cg22927302	0.02928482	12.9663594	1.29464826	129.862668
cg01536987	0.02928516	7.3229404	1.2222018	43.8761062
cg02461363	0.02928831	6.13008883	1.20045808	31.3030415
cg05890484	0.02929555	6.72938332	1.21170085	37.3727556
cg23939875	0.02929558	8.28760402	1.23738821	55.5075437
cg03105222	0.02930101	6.0057339	1.19782993	30.1118204
cg24458009	0.02930383	13.7499621	1.30196619	145.212264
cg19896639	0.02930405	236.47655	1.73372741	32254.8739
cg21378238	0.0293042	0.08092101	0.00843437	0.77637245
cg18345226	0.02930489	29.6355387	1.40659036	624.392986
cg21073212	0.02930622	17.1612031	1.33129111	221.219003

cg26818792	0.02930631	165843966	6.72068536	4.0925E+15
cg19540943	0.0293099	2670290.33	4.43388051	1.6082E+12
cg07529884	0.02932025	6.21676698	1.20176776	32.1594514
cg09639151	0.02932069	6.1659417	1.20077046	31.6620356
cg21212076	0.02932577	8.92688738	1.24622596	63.9445181
cg23672425	0.02932738	0.01590417	0.00038358	0.65943287
cg03442712	0.02932763	15.4177197	1.31658244	180.547813
cg05362170	0.02933533	585585.669	3.79881902	9.0268E+10
cg17653784	0.029337	2823479.6	4.4488791	1.7919E+12
cg26752655	0.02934087	49338.2727	2.96159114	821945044
cg18169610	0.02935497	71.5276727	1.53523243	3332.52988
cg01565438	0.0293582	6.33488598	1.20357654	33.3429401
cg10187792	0.02937645	7.96605806	1.23131742	51.5367363
cg27395200	0.02937758	6.12854074	1.19934855	31.316177
cg03035419	0.0293798	8.7205E+12	19.8308463	3.83E+24
cg06328288	0.02938141	103.230044	1.59174718	6694.8081
cg17543218	0.02938422	6.81173086	1.21203997	38.2822995
cg02601318	0.02938489	4587.2023	2.32799104	9038877.1
cg14653977	0.02938635	25.8460275	1.38530933	482.215143
cg22109796	0.02938726	24.4390465	1.37753766	433.575803
cg07266404	0.02939335	4.54083146	1.16367321	17.7190212
cg02619135	0.02939425	35246622.9	5.70194665	2.1788E+14
cg12656077	0.02939588	57.2193962	1.49983638	2182.94432
cg10122766	0.02939811	3800771618	9.10849278	1.586E+18
cg25637952	0.02939872	70660.4548	3.05942582	1631972845
cg04544093	0.0294089	19678492	5.371227	7.2096E+13
cg11439393	0.02941412	78.6721946	1.54775616	3998.89488
cg14732852	0.02941659	939.81122	1.98359107	445275.815
cg23323203	0.02942307	25.7041548	1.38362962	477.514766
cg01064131	0.02942486	0.03589247	0.00179685	0.71695956

cg13538006	0.0294251	44.1026789	1.46032922	1331.92315
cg04604505	0.02942684	1562982.31	4.16247357	5.8689E+11
cg07533511	0.02942755	9.78742186	1.25619168	76.257173
cg19760410	0.02943948	11.0421623	1.2712322	95.9143015
cg07983905	0.0294401	4.20697403	1.15437437	15.3317943
cg01683593	0.02944809	6017382.6	4.75437944	7.6159E+12
cg23753247	0.02945221	25.9947195	1.38445685	488.079813
cg02308245	0.02945349	69237.708	3.04287874	1575435838
cg18628449	0.02945532	1099.86849	2.01201945	601242.052
cg09297288	0.02945748	4.03551661	1.1494304	14.16823
cg16679650	0.02945849	11.5236868	1.27633688	104.044128
cg26694290	0.02945901	11.2274061	1.27301391	99.0206363
cg17322163	0.0294597	60910.7054	3.00305592	1235446200
cg25616762	0.02946314	1907.19745	2.1249856	1711730.24
cg16713947	0.02946919	16.0211266	1.31879678	194.629304
cg09433665	0.02946964	53653511.9	5.90297	4.8767E+14
cg27359949	0.02947048	0.00019107	8.58E-08	0.42564263
cg00957535	0.02947301	1081364.79	3.99769343	2.9251E+11
cg24889708	0.02947395	5.09826191	1.17639295	22.0948913
cg08579502	0.02948054	2.2548E+11	13.5472189	3.75E+21
cg10743104	0.02950286	7.28483924	1.21863159	43.5479297
cg08130144	0.02950429	8.21419775	1.23326766	54.7107872
cg09848727	0.02951007	551023326	7.41347521	4.0956E+16
cg24843609	0.02951368	0.16650175	0.03313717	0.83660835
cg22532997	0.02951523	2142501.43	4.2652569	1.0762E+12
cg23958863	0.02951638	34668.9198	2.82949152	424787985
cg22830895	0.02953642	7.9714448	1.22913556	51.6980666
cg24365117	0.02953772	10.323335	1.26110663	84.5061336
cg26822986	0.02953807	5.18313206	1.17764292	22.8123972
cg22174408	0.02954245	82165.9374	3.07811872	2193301128

cg01349853	0.02954264	15.543705	1.31335541	183.96145
cg12527260	0.02954787	21.5800006	1.35675324	343.243275
cg26413730	0.0295485	102.642234	1.58402595	6651.04523
cg03360992	0.02955067	20.709588	1.35115332	317.422923
cg26891370	0.02955207	14.6521751	1.3054878	164.449054
cg00694336	0.02955469	83.7003797	1.55201832	4513.9632
cg06189265	0.02955701	4988.88757	2.32866265	10688108.5
cg08204817	0.02956057	6.9193E+10	11.9095749	4.02E+20
cg02711212	0.02956158	4.50336353	1.1610772	17.4667826
cg08199013	0.02956441	75.6590196	1.53617426	3726.32676
cg07748159	0.02957145	0.11525386	0.01645837	0.8070939
cg21252725	0.02957749	1925.75488	2.11687496	1751889.91
cg02476533	0.0295786	38.1617809	1.43490051	1014.92857
cg11699666	0.02958706	735.68095	1.92355222	281368.218
cg25167574	0.02959145	6.38025072	1.20155629	33.8790614
cg07690778	0.02959751	372.850964	1.79764523	77333.3019
cg06521987	0.02960008	392517.984	3.58078244	4.3027E+10
cg09181339	0.02960284	1473714.67	4.0811622	5.3216E+11
cg10083643	0.0296069	17351706.9	5.2078978	5.7813E+13
cg09150450	0.02961079	7.9367906	1.22755645	51.3154771
cg23895155	0.02961139	930.691416	1.9670925	440338.474
cg26918756	0.02961552	8.01413987	1.2286684	52.2732069
cg02512352	0.0296211	4.77518205	1.16724938	19.535126
cg02569236	0.02963085	13.7703032	1.29598343	146.314564
cg11509669	0.02963284	8.81136039	1.23999369	62.6132797
cg11335133	0.02963291	4.06250483	1.14863044	14.3683686
cg07803108	0.02963587	10.1215106	1.25705346	81.4961178
cg10723962	0.02963909	4.63428034	1.16361881	18.4566922
cg01402045	0.02964172	712323583	7.49326459	6.7715E+16
cg01941047	0.02964387	3.4678E+10	10.9967205	1.09E+20

cg21487267	0.02964645	4.4146E+13	22.274048	8.75E+25
cg19305040	0.02965026	2262811096	8.39122763	6.102E+17
cg23425533	0.02965161	5.97963045	1.19315065	29.9676998
cg10089657	0.02965326	32.4015654	1.40978231	744.697555
cg13077699	0.02965712	41.7967911	1.44555881	1208.50963
cg22211300	0.02966065	704.488109	1.91020706	259816.596
cg12222095	0.02966132	5.6438E+11	14.4483212	2.20E+22
cg19381368	0.02966413	29.6010349	1.39697072	627.229518
cg23930313	0.02967106	3.87809531	1.14304102	13.1575534
cg02573089	0.02967283	651886.217	3.74505407	1.1347E+11
cg24377179	0.02967395	22.1486793	1.35732782	361.418948
cg17125688	0.02967583	39.3069385	1.43627565	1075.72346
cg10872815	0.02967692	6.35647398	1.20006268	33.6688758
cg17270013	0.0296814	30.3047932	1.39975781	656.09957
cg07850533	0.02969131	157113.986	3.25070616	7593674505
cg22841359	0.02969395	2.2684E+11	13.143257	3.92E+21
cg26870192	0.0296955	5.33864954	1.17938676	24.1661006
cg06935581	0.02970248	6664.54678	2.37966824	18664863.9
cg02634501	0.02970688	2467566550	8.40626582	7.2433E+17
cg23963351	0.02970748	23363421.8	5.31320156	1.0273E+14
cg15695155	0.02970927	5.95629591	1.19201241	29.7626608
cg08222053	0.02971301	0.02325965	0.00078333	0.69065129
cg21232625	0.0297205	7.00067389	1.2109733	40.4711111
cg20770516	0.02972506	49.1697508	1.46678364	1648.27609
cg01814495	0.02972771	165819.124	3.2601405	8433986783
cg21650862	0.02973169	20.1225877	1.34325967	301.444722
cg06445928	0.02973352	25.5413681	1.37507487	474.418885
cg14669362	0.02973964	4.68265306	1.16381511	18.8408274
cg11939825	0.02974756	242.042374	1.71451842	34169.6595
cg16246590	0.02975793	221850.955	3.34792645	1.4701E+10

cg06637618	0.02975987	14.9264754	1.30384071	170.879515
cg01043831	0.02976025	7.03199702	1.21098696	40.8336206
cg10567498	0.02976052	0.07988202	0.00817748	0.78033081
cg21554588	0.02976901	13.0598043	1.28668879	132.556131
cg10122252	0.0297711	0.00887073	0.00012508	0.62909798
cg03554335	0.02977678	8.67257901	1.23592535	60.8561242
cg05597001	0.02978214	11.3330641	1.26868917	101.237044
cg03616164	0.02978444	352193.075	3.49677489	3.5473E+10
cg08546494	0.02978671	7.67184181	1.22101879	48.2033176
cg01045118	0.02979198	8.23061607	1.22938545	55.1031744
cg15622885	0.0298073	767578.275	3.76780682	1.5637E+11
cg17346022	0.02981237	0.12925347	0.02040984	0.81854924
cg02160530	0.02981643	6.0198042	1.19199183	30.4012509
cg25461508	0.02981791	5.91500507	1.18992817	29.4028545
cg19263607	0.02981794	10.0589126	1.25337179	80.7276207
cg08781140	0.02982029	2.55315373	1.09602183	5.94750378
cg02896768	0.0298257	3.98944609	1.1448848	13.901556
cg20058667	0.02982659	227569.686	3.34066492	1.5502E+10
cg08434234	0.02982731	4.65576302	1.16229209	18.6494681
cg26880187	0.02983095	22253.884	2.6607027	186129533
cg02426376	0.02983193	59.2189504	1.49026598	2353.1934
cg11334004	0.02983946	205.580985	1.68269548	25116.5716
cg06473182	0.02984854	8159712.04	4.73157744	1.4072E+13
cg14614337	0.02984856	33581525.8	5.43263885	2.0758E+14
cg17832674	0.02985055	1595.31738	2.05475479	1238608.89
cg20368841	0.02985475	1804.81713	2.07930111	1566567.18
cg19036153	0.02985868	1411.88585	2.02973284	982110.355
cg12545993	0.02985981	4.97120528	1.16943046	21.1324083
cg26009944	0.02986325	23.6237498	1.36148325	409.907032
cg20170271	0.02987289	7.80475645	1.22188318	49.8527392

cg00516067	0.02988199	9.47564893	1.24508039	72.1141568
cg10968199	0.02988692	238.241192	1.70467333	33296.0367
cg18575176	0.02990355	43025.146	2.82575061	655104941
cg10247383	0.02990406	172.911712	1.65145066	18104.3618
cg05151395	0.02990408	56.3380244	1.48064465	2143.64264
cg23118464	0.02992968	5.03730278	1.17021109	21.6836256
cg18627816	0.02993297	10.41084	1.25573065	86.3127687
cg05862007	0.02994357	0.22100371	0.05655659	0.86360647
cg21199629	0.02994365	30.1597111	1.39222322	653.349378
cg18376163	0.02994418	4789.52426	2.27764543	10071603.9
cg26616609	0.02996301	23.0147833	1.35568225	390.711208
cg09704415	0.02998013	26.300625	1.37292922	503.829961
cg21978251	0.02998099	5.90502356	1.18783668	29.3553009
cg09981375	0.02998253	280855.22	3.37357902	2.3382E+10
cg18492839	0.02999376	0.03265936	0.00148577	0.71789809
cg24874612	0.03000612	4.7032941	1.16167595	19.0422944
cg25028206	0.03000889	1.26E-07	7.38E-14	0.21489298
cg07194846	0.03001407	21.331229	1.34457092	338.413783
cg00561338	0.03001819	4.88386941	1.16579742	20.4599702
cg07704437	0.03002383	357782.992	3.44366636	3.7172E+10
cg11032474	0.03002805	32.5390322	1.4002664	756.133705
cg24153171	0.03003104	1178.51389	1.98092804	701133.484
cg05956498	0.03003367	145.060875	1.6177062	13007.7127
cg12164596	0.03003481	10.7385295	1.25784643	91.6773404
cg09216143	0.03003741	0.16468283	0.03228393	0.84005992
cg13125627	0.0300392	4.36290274	1.15295056	16.5097455
cg22473973	0.03004152	773.873215	1.90128138	314987.44
cg27338695	0.03004553	169915019	6.23536866	4.6302E+15
cg13957558	0.0300594	10.6541647	1.25648663	90.3401767
cg21295575	0.03006466	0.2314671	0.06170045	0.86834079

cg15239888	0.03006791	305.814592	1.73674468	53849.3459
cg21949305	0.03007017	10.1244326	1.25014681	81.993678
cg01579086	0.03007606	1144.66466	1.97192041	664457.432
cg23679724	0.0300781	8.69159599	1.23176751	61.3296262
cg08069370	0.03007929	1617.62829	2.03851316	1283642.09
cg14876077	0.03008429	8.00156596	1.22189809	52.398034
cg01370334	0.03009001	45.8076167	1.4454565	1451.67825
cg26985711	0.03009212	8.77272027	1.23266519	62.4343267
cg11699265	0.03009559	5.07589117	1.16934681	22.0333873
cg23710492	0.03011216	13.9368507	1.28849615	150.746131
cg22880620	0.03011255	7.24184016	1.20983602	43.3482291
cg18119621	0.03011269	1344.34301	1.99983077	903705.534
cg01424889	0.03011582	124.846708	1.59094773	9797.11659
cg09135551	0.0301185	5.14555445	1.17064182	22.6172772
cg08299454	0.0301243	507.31279	1.82012705	141400.166
cg24181662	0.03013047	9.58077389	1.24258072	73.8714411
cg10453879	0.03013062	9.8772E+10	11.3948754	8.56E+20
cg19115492	0.03013875	13.7466936	1.28630256	146.910681
cg04314218	0.0301395	1424.10206	2.00880541	1009588.42
cg02853019	0.03014219	518.073112	1.82268592	147255.074
cg06416883	0.03014636	394.199329	1.77522709	87534.2156
cg24986868	0.0301484	17.4271303	1.31575496	230.821755
cg06768655	0.03014994	144.607552	1.61209348	12971.5456
cg07424295	0.03015943	232.273245	1.68665371	31986.9217
cg20254361	0.03016174	8.69713229	1.23062154	61.464965
cg02792794	0.03016288	84740.7063	2.97012255	2417741083
cg14826401	0.03016858	4.15E-13	2.65E-24	0.06494517
cg25505476	0.03016935	53659612.3	5.5114573	5.2243E+14
cg14219044	0.0301755	583856.398	3.57099787	9.546E+10
cg06605053	0.03018221	9.4680355	1.24037701	72.2713302

cg08435603	0.03019401	74378315.4	5.67285022	9.7519E+14
cg08016383	0.03019755	5.96954094	1.18656677	30.0323758
cg05447008	0.03019833	3.90898821	1.13942167	13.4104776
cg04246008	0.03020198	8801.55027	2.38547416	32474586.5
cg14309262	0.03020314	156.114218	1.62159794	15029.403
cg02887499	0.03021749	545940.17	3.53731981	8.4259E+10
cg13298466	0.0302189	0.05659855	0.00421577	0.75985999
cg02403395	0.03022847	3.05712324	1.11271503	8.3992777
cg08125503	0.03023307	73.0165275	1.50678096	3538.28023
cg12730843	0.03023332	27.2765788	1.37147532	542.490074
cg23175074	0.0302357	8.5578707	1.22764916	59.6564176
cg08718097	0.03023832	0.29432892	0.09736519	0.88973804
cg01169952	0.03024435	179.330703	1.64133733	19593.4744
cg02059453	0.03025396	24.4624248	1.3567898	441.048591
cg02285386	0.03025661	22.2095367	1.34427637	366.936093
cg08143349	0.03026769	27.1667141	1.37009528	538.670822
cg19044674	0.03026913	6.62E-09	2.64E-16	0.16599294
cg00750366	0.03027808	5.51639824	1.17674579	25.860003
cg23356310	0.03028191	0.18911632	0.04191538	0.8532664
cg05343713	0.03029961	698.903965	1.86530135	261870.154
cg13516551	0.03030307	3379.22558	2.16684377	5269953.3
cg18325192	0.03030984	8.42462047	1.22475094	57.9499291
cg21389025	0.03031101	3.9681E+14	24.4721734	6.43E+27
cg23158347	0.03032288	1711172665	7.5458225	3.8804E+17
cg10205925	0.03032675	64.2859619	1.48538594	2782.22971
cg20247708	0.03033593	63322269.4	5.50872517	7.2788E+14
cg10575842	0.03033685	6.27398457	1.19056385	33.0623866
cg06521149	0.03034351	0.18248641	0.03913852	0.85085719
cg02289168	0.03034961	155118.511	3.10928529	7738676346
cg22349506	0.03035412	6.12818295	1.18770471	31.6194977

cg26814987	0.03036524	4.74407588	1.15909148	19.4171525
cg14344110	0.03036575	685.201735	1.85739994	252773.464
cg03640148	0.03036731	65.2860298	1.48618973	2867.91491
cg22232207	0.03036826	3.51029336	1.1264308	10.939118
cg22525895	0.03037048	6.2969394	1.19057435	33.3044684
cg22714033	0.03037413	2707.31889	2.11510671	3465345.52
cg20828120	0.03038031	186.480076	1.64108586	21190.1275
cg07862554	0.03039147	5.9813066	1.18454161	30.2024246
cg25717844	0.03039478	6.73166827	1.19782997	37.8312106
cg10083258	0.03040305	5.77385402	1.18045828	28.2410575
cg12627700	0.03040778	49177570.3	5.34076938	4.5282E+14
cg21784768	0.03040964	12671930.7	4.69701872	3.4187E+13
cg07322750	0.03042023	27.172075	1.36635517	540.358522
cg15267303	0.03042485	5.22E-08	1.33E-14	0.2050189
cg19895051	0.03042661	191803627	6.0627107	6.068E+15
cg14970695	0.0304354	6.47080558	1.19286277	35.1015439
cg13325231	0.03043695	7.59706315	1.21105711	47.6570162
cg13823666	0.0304371	13.7103942	1.28049374	146.798772
cg01399540	0.03043931	73939.5418	2.88226854	1896789205
cg24517380	0.03044131	49.2757943	1.4447918	1680.59087
cg12810523	0.03044216	8.38725176	1.222353	57.5496538
cg11166999	0.03044616	0.0513382	0.00348817	0.75558622
cg13903087	0.03044702	0.05687736	0.00424023	0.7629391
cg18798248	0.03044999	3.36884627	1.12143908	10.1201442
cg27392956	0.03045123	287.501445	1.70605305	48449.303
cg22617213	0.03045679	31.111216	1.38300412	699.858916
cg10315334	0.03045774	11.5254743	1.25932656	105.482217
cg21042539	0.03045927	43.6850823	1.42792946	1336.47108
cg26232870	0.03045982	25.1423587	1.35542021	466.378025
cg13612524	0.03047	283.990446	1.70309091	47355.4129

cg20333292	0.03047336	7.45087256	1.20835492	45.943043
cg03169170	0.03047394	196104.87	3.15306944	1.2197E+10
cg06922635	0.03047708	4.7504175	1.15813636	19.485155
cg21399057	0.03047998	4.78058778	1.15879874	19.7221646
cg01656221	0.03048729	16.5908806	1.30276081	211.287688
cg24116028	0.03049846	0.05300318	0.0037038	0.7585009
cg17504394	0.03050191	3.06759144	1.1112167	8.46830077
cg17153568	0.03050261	10.423669	1.2467325	87.1501098
cg24105797	0.03050531	2.2087E+10	9.39730565	5.1914E+19
cg14212180	0.03050656	283.8774	1.70111073	47372.7999
cg27422678	0.0305074	301525.739	3.27597424	2.7753E+10
cg12230071	0.03050812	36298.2854	2.68440275	490822595
cg03198733	0.0305108	7.73126252	1.21206276	49.3146249
cg02837488	0.03051315	1147.02544	1.93937805	678396.544
cg07012770	0.03052294	4.35661999	1.14830979	16.5287607
cg25289803	0.03052454	3.21745861	1.11605379	9.2755744
cg19319928	0.03052627	9.00665884	1.22936348	65.985288
cg23861541	0.0305292	0.21727854	0.05448911	0.86641102
cg07783282	0.03054432	6.14406858	1.18576089	31.8357428
cg04035209	0.03054657	5.28972976	1.16919208	23.9321164
cg01045986	0.03054961	5374.19908	2.23864348	12901570.1
cg08576184	0.03054979	17.2877473	1.30654901	228.744735
cg26009773	0.03055016	1684278068	7.33857809	3.8656E+17
cg07537523	0.03055718	19.0978593	1.31865615	276.590854
cg03259703	0.03056089	0.1507212	0.0271271	0.83742374
cg11388673	0.03056491	0.27024559	0.0825626	0.88457338
cg08247611	0.03056551	17493294.9	4.77433229	6.4096E+13
cg27219362	0.03057013	0.22312979	0.05730096	0.86886689
cg00662646	0.03057433	10118.1147	2.37261282	43149157.9
cg06237983	0.03057823	9.34852454	1.23289374	70.8860044

cg08468187	0.03058584	22.66823	1.3393697	383.649602
cg13144594	0.03058935	71.9847709	1.492272	3472.4281
cg06916552	0.03059425	505329.015	3.41770073	7.4716E+10
cg27628552	0.03059584	16238.0986	2.47731181	106436278
cg07490151	0.03059865	5542.33265	2.23996329	13713372.6
cg12179176	0.03060009	9.18587286	1.2305438	68.5715213
cg14269191	0.03060026	0.17960649	0.03787907	0.85161774
cg18913687	0.03060165	1.9695E+10	9.18163532	4.2247E+19
cg10164640	0.03060368	47.1036893	1.43376087	1547.50879
cg02203311	0.0306068	4970596574	8.06708681	3.0627E+18
cg05749728	0.03061155	3.94226945	1.13682385	13.6709733
cg05704893	0.03061396	305.737822	1.70729913	54750.5791
cg03451178	0.03061407	7.36205766	1.20514489	44.9737565
cg07024545	0.03062144	36626860.4	5.08970779	2.6358E+14
cg05235884	0.03062351	0.18545277	0.04025576	0.8543555
cg13561226	0.03063797	81.9695402	1.5087672	4453.30832
cg15616257	0.03064237	6.06796909	1.18323587	31.1182662
cg02653313	0.03064246	6.3298E+13	19.4043224	2.06E+26
cg07813370	0.03064354	3849.0244	2.16048724	6857244.3
cg06363129	0.03064661	6.75292481	1.19505155	38.159018
cg03415545	0.03065311	6.07320278	1.18320645	31.1727441
cg23211736	0.03065642	9.01513923	1.22755871	66.2068012
cg23536255	0.03066794	38.9855488	1.40681612	1080.36366
cg21697812	0.0306719	0.14410837	0.02487436	0.83488449
cg24718991	0.03067898	2637950.74	3.96219488	1.7563E+12
cg11816600	0.03067942	771493.502	3.53348292	1.6845E+11
cg07407499	0.0306808	9.64001338	1.23488262	75.2540011
cg24476453	0.03069269	2317.97714	2.05637218	2612862.63
cg00674304	0.03069421	5.41799003	1.17023433	25.0843913
cg15128200	0.03069617	6.11604628	1.18348062	31.6067889

cg00042409	0.03069981	11.2818777	1.25278513	101.598239
cg16361168	0.03070737	114.489613	1.55378442	8436.09398
cg24803730	0.03071667	12.7217166	1.26656352	127.780464
cg17636471	0.03072021	1.01E-13	1.65E-25	0.06207503
cg27487704	0.0307205	17345.844	2.47622062	121507067
cg19091719	0.03072862	4.57255985	1.15158166	18.1561624
cg07591620	0.03073	95.4550321	1.52688473	5967.48592
cg00178359	0.03073799	5.32231291	1.16783188	24.2560725
cg01170643	0.03074298	1147099.91	3.64881448	3.6062E+11
cg07907315	0.03075527	7.11617141	1.19951692	42.2169082
cg23820828	0.03075732	10.0431639	1.23841432	81.4470086
cg14940444	0.03075855	7.66778017	1.20780378	48.6791429
cg08805821	0.03076289	17.8988984	1.30644101	245.223903
cg11524454	0.03076481	10.602925	1.24453649	90.3324398
cg05637945	0.03076875	263535.28	3.1778641	2.1855E+10
cg14184729	0.03076949	92.8279818	1.52145552	5663.67801
cg21638712	0.03078962	4.78124437	1.15576437	19.7793758
cg10927534	0.03079787	3622021.04	4.04131551	3.2462E+12
cg16793187	0.03079999	10.2252662	1.23981129	84.332245
cg13725062	0.03080047	0.01152649	0.00020073	0.66189257
cg21931680	0.03080174	11.1446401	1.24969173	99.3869134
cg21950518	0.03080227	16.7364686	1.29755596	215.874604
cg15456099	0.03080644	12389451	4.52462607	3.3925E+13
cg21210285	0.03081052	546.358761	1.790415	166725.533
cg24397995	0.0308123	138.027435	1.5765966	12083.987
cg04789663	0.03081376	10.0893248	1.23806426	82.220671
cg10160975	0.03081711	5.45167052	1.16958537	25.4113228
cg09575314	0.0308175	45536.661	2.69323713	769923847
cg08863844	0.03082238	16.6675568	1.29666317	214.24797
cg15492422	0.03082553	8.5785E+10	10.2188133	7.20E+20

cg07796220	0.03083086	13.6611137	1.27291132	146.613535
cg17697381	0.03083237	18151.9897	2.47193357	133294331
cg09612202	0.03083252	463.759668	1.76221116	122047.252
cg26029577	0.03083347	4.6276752	1.1518591	18.5920117
cg14040796	0.03084699	51.7309509	1.43885953	1859.86973
cg27087555	0.03084707	116.343157	1.55050501	8729.88489
cg22157173	0.03084777	138.98078	1.57610322	12255.3251
cg18259504	0.03085105	22.7590178	1.33385353	388.328163
cg06973760	0.03085422	21274952.8	4.73574152	9.5576E+13
cg24405617	0.03085625	4.94069452	1.15860859	21.0687739
cg14186963	0.03086035	8.4813194	1.21770719	59.0723119
cg14523898	0.03086426	5.37015324	1.16745726	24.7020142
cg07270285	0.03086446	0.12626681	0.01929123	0.82645342
cg21747310	0.03087422	5.90119535	1.17752923	29.5738787
cg10232654	0.03087605	8.61300891	1.21921367	60.8457111
cg20599535	0.03088022	1.5743E+10	8.67759821	2.8563E+19
cg01105418	0.03088435	6.30618551	1.18462684	33.5700444
cg00504595	0.03088975	7.5313173	1.20406456	47.1077232
cg27093637	0.0308945	0.20054604	0.04662177	0.86265955
cg07318204	0.03089661	5.06099825	1.16076854	22.0661591
cg10227731	0.03089689	609496.757	3.40287626	1.0917E+11
cg04186815	0.03090264	152.065565	1.58686105	14572.1241
cg21911557	0.03090353	5.864E+12	14.9071899	2.31E+24
cg10248985	0.0309063	15.1121176	1.28340043	177.946098
cg27370013	0.03090777	12.1488318	1.25789292	117.334401
cg17942763	0.03091079	10.746882	1.24375314	92.8604465
cg19893185	0.03091119	7.379352	1.20153042	45.3212293
cg02498311	0.03091776	9.67E-06	2.70E-10	0.34637589
cg26576398	0.03091924	8.01605369	1.2105897	53.0791866
cg15943584	0.03092412	49.3801859	1.43037027	1704.73535

cg04636406	0.03092489	6.9669697	1.19502821	40.6171725
cg17868910	0.03092507	20.3463147	1.31855146	313.96008
cg23880953	0.03092565	3900.11604	2.13594252	7121401.89
cg09675001	0.03093033	12.3319766	1.25923707	120.769671
cg25724712	0.03093104	0.0016448	4.87E-06	0.55536133
cg10943191	0.03093133	1060.07219	1.89484012	593059.559
cg05388307	0.03093725	2.4774E+13	16.9223577	3.63E+25
cg14089881	0.03093762	0.29821604	0.09937006	0.8949658
cg24767968	0.03093907	5.13048068	1.16178777	22.656317
cg22864672	0.03094124	113.128502	1.54277933	8295.45599
cg08284713	0.03094515	607721.208	3.39014721	1.0894E+11
cg15640707	0.03094666	1.04E-06	3.82E-12	0.28283982
cg19701264	0.03094984	9.50013575	1.22915927	73.4262689
cg20458811	0.03095021	9.19030788	1.22542437	68.9244978
cg25978167	0.03095155	1356.60954	1.93668071	950280.26
cg08163199	0.03095538	4.03284849	1.13628083	14.3132459
cg08229694	0.03095571	6587456910	7.93534122	5.4685E+18
cg15506890	0.03095982	5.75101978	1.17378611	28.1773895
cg09874482	0.03097869	7.81212977	1.20693054	50.5657694
cg25220781	0.03098625	19.863034	1.31434429	300.18019
cg12253571	0.03098768	769.463439	1.83622521	322440.831
cg15190747	0.03099253	0.14929792	0.02652248	0.84041425
cg06693487	0.03099599	6.33E-05	9.70E-09	0.41330265
cg06838028	0.03099865	15.0736055	1.28136121	177.322039
cg14826425	0.03100003	3.96096717	1.13403303	13.8349241
cg11208039	0.0310084	107.109652	1.53246019	7486.31358
cg13364230	0.03101073	9.42200184	1.22732228	72.3315469
cg12076945	0.03101118	9106929.28	4.32027895	1.9197E+13
cg00384653	0.03101134	0.02101082	0.00062817	0.70276679
cg03132197	0.03101141	4923958.31	4.08429188	5.9362E+12

cg17785773	0.03101258	5.70061063	1.17225762	27.7216894
cg17477995	0.03101304	158.362704	1.58797821	15792.878
cg05605149	0.03101725	1.9896E+10	8.71201908	4.5437E+19
cg01467604	0.03102553	214531.534	3.06509813	1.5015E+10
cg09619475	0.0310302	9604540.97	4.33414941	2.1284E+13
cg22934878	0.03103087	0.11336531	0.0156748	0.81989495
cg02697115	0.03104691	5.28919915	1.16390737	24.0359572
cg11797883	0.03105011	58091626.4	5.09759663	6.6201E+14
cg22703115	0.0310525	6.54257357	1.18661524	36.0734193
cg18187762	0.03106514	2330.29988	2.02544957	2681033.19
cg17195127	0.03106854	94.1131202	1.51222667	5857.11097
cg04599533	0.03107239	96946610.7	5.32927752	1.7636E+15
cg10634273	0.03107409	59.2583575	1.44970748	2422.24931
cg10324701	0.03107709	0.12740037	0.01957655	0.82909663
cg16998150	0.03107765	4.35828409	1.14327588	16.6142228
cg14593314	0.03107918	532978.941	3.31775435	8.562E+10
cg01117697	0.03107935	10.0414471	1.23342423	81.7485639
cg00945238	0.03108113	7.22416063	1.19700974	43.5990577
cg14966074	0.03108193	6.55358871	1.18644241	36.200261
cg02988775	0.03108776	7.25580819	1.19740072	43.9675303
cg03860859	0.03109275	233.610905	1.64150042	33246.4459
cg14340070	0.03109726	56.4167914	1.44251283	2206.46519
cg03133269	0.03110324	221.718295	1.63322483	30099.3478
cg03994465	0.0311051	10.9236777	1.24249421	96.038061
cg07447902	0.03110972	638503350	6.30053866	6.4707E+16
cg00194126	0.03111478	0.13599266	0.02216518	0.8343721
cg03284670	0.03111635	0.17355431	0.0353095	0.85305935
cg20568322	0.03111674	57.5852269	1.4445858	2295.50807
cg17254405	0.03111882	18.9857178	1.30616892	275.965439
cg01803717	0.03112019	30761202.6	4.77943051	1.9798E+14

cg03040420	0.03112069	719.293539	1.81633261	284850.468
cg13598790	0.03112372	62992.4194	2.72484192	1456247744
cg24055398	0.03112667	57.2130581	1.44342322	2267.75763
cg15673540	0.03113605	179.916489	1.60105581	20217.873
cg13666293	0.03113714	758.432007	1.82401075	315359.495
cg21845654	0.03114969	400360.907	3.21670244	4.983E+10
cg05355757	0.03115825	4.729932	1.15103909	19.4365743
cg20090147	0.03115956	167744503	5.55218777	5.068E+15
cg10714284	0.03116122	11464.3099	2.33023937	56402103.1
cg25953133	0.03116456	190.454257	1.60803912	22557.1775
cg18236571	0.03117105	7.61562608	1.2015837	48.2677657
cg07718321	0.0311714	529.434498	1.76349901	158945.872
cg21084284	0.03117763	8.73655386	1.21650751	62.7430349
cg05251593	0.03118343	7.96590382	1.20631403	52.6029063
cg15050111	0.03118675	15948.6605	2.39771993	106084022
cg18058279	0.03118749	0.01968149	0.00055243	0.7011993
cg07723431	0.03119054	1547.56664	1.94170032	1233435.71
cg19005707	0.03119214	0.13507848	0.0218633	0.83455833
cg02098923	0.03119232	9.91005843	1.23022657	79.8302205
cg19771626	0.03119942	0.12266436	0.01818556	0.8273897
cg02454483	0.03119991	4493298500	7.44070005	2.7134E+18
cg03308399	0.03120584	5.30207725	1.16250106	24.1823634
cg20082196	0.03121265	7.0478903	1.19267018	41.6483606
cg02227867	0.03121835	1743.79669	1.96056235	1550997.29
cg26383001	0.03122351	257342.807	3.07520401	2.1535E+10
cg00630080	0.03122535	98132.5385	2.81883517	3416303020
cg17460737	0.03124246	100.147277	1.51424566	6623.41477
cg02753742	0.03124367	21.40447	1.31773788	347.680174
cg04176995	0.03124862	32.9249375	1.3697213	791.439477
cg01439366	0.03126602	122381.36	2.86815045	5221900820

cg13864794	0.03126825	4.1506452	1.13654709	15.1580658
cg20922251	0.0312693	6830.49548	2.21214009	21090738.7
cg12510981	0.03127634	4.17340896	1.13703551	15.3182044
cg16985320	0.03128521	1798.07755	1.96069215	1648949.76
cg00931558	0.03128533	4.9485498	1.15449195	21.2111874
cg10864356	0.03128628	90636791.4	5.18599602	1.5841E+15
cg07509155	0.03128881	13.4290651	1.26276017	142.813967
cg12968701	0.03129138	0.15727154	0.02920424	0.84694338
cg17186803	0.03129336	9.45476603	1.22351388	73.0621877
cg13532478	0.03129777	30.5036399	1.35910386	684.621741
cg07187607	0.03129902	13.4678526	1.2629069	143.623455
cg17324226	0.03130271	1364.41388	1.91137477	973971.855
cg03506799	0.03130507	2347017.39	3.72942004	1.477E+12
cg14886640	0.03130729	159.442007	1.57622523	16128.2493
cg12482825	0.03131271	12.821045	1.25710341	130.760281
cg10585486	0.03131468	18.8615758	1.30135154	273.376586
cg11340260	0.03131634	11.4161522	1.24402638	104.763479
cg18048370	0.0313208	14.0608463	1.2674083	155.993455
cg01550215	0.03133159	9393.31683	2.26946413	38878958.2
cg25852545	0.03133404	1.1622E+14	18.1935649	7.42E+26
cg06649191	0.03133787	21.9592281	1.318711	365.665942
cg00489408	0.03134829	117.293087	1.53179088	8981.42724
cg07667560	0.03135094	9.52310112	1.22345085	74.125949
cg10224037	0.03135279	539.441968	1.75566832	165747.501
cg12635790	0.03137056	5.9971278	1.17363392	30.6446
cg17083504	0.03137057	3007.51191	2.0458898	4421121.74
cg20651988	0.03137705	54.8331343	1.43012876	2102.37895
cg03940662	0.03137883	17.4561848	1.29107159	236.019747
cg11653266	0.03137939	1608615.36	3.58458132	7.2188E+11
cg24684739	0.03138387	6469939.79	4.05762243	1.0316E+13

cg10773064	0.03139061	8.41165081	1.20938968	58.5054349
cg04467350	0.03140783	391308701	5.83820012	2.6228E+16
cg03938320	0.03141163	7.0823E+10	9.27673162	5.41E+20
cg21440776	0.03142121	0.15658662	0.02892431	0.84770797
cg14597534	0.03142249	3.83129594	1.12713698	13.0231097
cg10678266	0.03142359	9.46412813	1.2216985	73.3157333
cg06856563	0.03142518	2.20E-06	1.55E-11	0.31335297
cg27547291	0.03142832	8.64031455	1.21175946	61.6087913
cg09022720	0.03143329	6.28805412	1.17788337	33.5683698
cg21071097	0.03143563	9.31177267	1.21975718	71.0871901
cg19906926	0.03143912	18.9862419	1.29955907	277.384375
cg03924483	0.03144323	46.812829	1.4081379	1556.26871
cg23017840	0.03144638	30.9508599	1.35715418	705.856226
cg18897345	0.03145208	6.85E-05	1.10E-08	0.42619413
cg24226590	0.03145271	8.91931819	1.21484622	65.4850267
cg26603598	0.03145683	0.14703222	0.02563633	0.84327483
cg05824225	0.03146126	0.00367409	2.22E-05	0.60751817
cg24302080	0.0314658	725336304	6.12946398	8.5833E+16
cg19954234	0.03147282	9.00101594	1.21554339	66.6519095
cg00501711	0.03147668	15.843119	1.27807964	196.391846
cg17199658	0.03148631	10.9382049	1.2365599	96.7557869
cg25213362	0.0314866	7.4392503	1.19496222	46.3131335
cg09534243	0.03148852	50444.9654	2.61432016	973367596
cg16930572	0.03149659	4.70266285	1.14719987	19.2774061
cg26072123	0.03152421	4116256.37	3.85254704	4.398E+12
cg24137660	0.03152579	0.1555158	0.02851771	0.8480752
cg23112423	0.03152845	0.22671295	0.05861569	0.87687723
cg22995904	0.03152887	0.18815354	0.04104425	0.86252662
cg07079755	0.03153993	3484.19039	2.05765489	5899717.56
cg10322499	0.03154746	61.4092944	1.43924347	2620.19702

cg21369988	0.03154893	492515.451	3.18667913	7.612E+10
cg10997640	0.03155179	2507624.64	3.67909416	1.7092E+12
cg01561271	0.03155357	11445.5916	2.28441073	57345890.1
cg16529993	0.03156182	4846.75401	2.11651901	11098896
cg05085816	0.03156492	46358.9764	2.58339922	831909632
cg16098981	0.03156584	4.97224318	1.15219979	21.4573917
cg02674839	0.03157484	0.03401003	0.00155901	0.74193333
cg27285599	0.03157726	4.05616853	1.13156321	14.5396236
cg19099736	0.03157884	1049.00477	1.84765332	595572.23
cg21872393	0.03158471	12965.819	2.30608583	72899481.8
cg02429905	0.03158516	4.57884579	1.14366126	18.3322017
cg18394527	0.03158585	84661.1485	2.72109316	2634055377
cg25032094	0.03159387	3.89414234	1.12736235	13.4511717
cg06739774	0.03159736	6.5763E+12	13.4918536	3.21E+24
cg01161216	0.03159861	23879459.6	4.47115128	1.2754E+14
cg26752003	0.03159974	413.664546	1.70081934	100609.367
cg18815565	0.03160521	5.63817001	1.16463816	27.2951396
cg07832245	0.03160802	0.18872194	0.04125239	0.86336749
cg00324161	0.03160845	5.59564039	1.16382651	26.9036588
cg05768582	0.03160865	46525.3474	2.57771778	839738146
cg11861043	0.03160909	3.16E-08	4.57E-15	0.21838752
cg13136677	0.03161011	25412.1144	2.44377229	264253572
cg23306295	0.03161524	8552514.28	4.07840398	1.7935E+13
cg06055013	0.03162157	7.57730782	1.19516157	48.0400266
cg04987149	0.03162538	74770941.1	4.93162929	1.1336E+15
cg17143179	0.03162776	10.2997067	1.2278033	86.4014286
cg01868762	0.03162952	777717.451	3.29866391	1.8336E+11
cg07935287	0.03163535	100156.533	2.75332467	3643352065
cg23739036	0.03163542	9.76E-08	3.94E-14	0.24174683
cg24569831	0.03163593	7.49413883	1.1938161	47.0441947

cg02014690	0.03163768	7248.10537	2.18522147	24041055.9
cg16463733	0.03163968	42140.2641	2.55081619	696170059
cg01678634	0.03164254	503237325	5.82195877	4.3499E+16
cg06434428	0.03164432	21.859262	1.31150336	364.33558
cg13732201	0.03164551	0.16848794	0.03319909	0.85508915
cg04551092	0.03164633	226.18344	1.61049342	31766.0089
cg12262378	0.03164985	8.39777659	1.20563923	58.4939921
cg13285458	0.03165137	2270.15477	1.97201069	2613374.6
cg06571407	0.03165564	3.39924048	1.11347999	10.3772281
cg17495719	0.03165663	4.13361215	1.13277035	15.0840366
cg00393798	0.03167301	6.39075369	1.17677549	34.7064782
cg18565264	0.03167397	15.044765	1.26858308	178.423438
cg09462445	0.03168425	3.8441822	1.12534592	13.1317282
cg08454455	0.03168831	4.39208708	1.13853638	16.9431818
cg26593267	0.03169474	5.09339762	1.15335598	22.4932284
cg12886812	0.0316968	35355242.1	4.58644805	2.7254E+14
cg16855355	0.03169834	6.82924676	1.18334069	39.4126659
cg18989709	0.03170529	4.99063895	1.15119324	21.6353573
cg00445566	0.03171427	2769697.11	3.6639804	2.0937E+12
cg09886849	0.03171563	0.11418627	0.01576576	0.82701416
cg01553935	0.0317391	24.8408527	1.32417053	466.003395
cg23061725	0.03175406	5.35128892	1.15774345	24.7345758
cg11196848	0.03175432	6.33519681	1.17493075	34.1592205
cg16791424	0.03175559	5.54303508	1.16129184	26.4578092
cg18104645	0.03175707	3.08894318	1.1034803	8.64679685
cg15204874	0.03176063	7.32928394	1.18990079	45.1452789
cg10116178	0.03176639	0.175107	0.0356973	0.8589574
cg06685766	0.03176899	594017.426	3.18958338	1.1063E+11
cg20814026	0.03177415	9.09503973	1.21233963	68.231497
cg26512283	0.03177524	75.2589203	1.4576634	3885.60559

cg22005145	0.03178333	5.10378102	1.15267178	22.598437
cg22492099	0.03178507	52.9232678	1.41328956	1981.81063
cg17507671	0.03178701	6.13638707	1.17129271	32.1484511
cg24718163	0.03179302	598086.997	3.18604052	1.1227E+11
cg22471112	0.03179844	13.2032455	1.25197971	139.240031
cg03549146	0.03179847	0.03464182	0.00160836	0.74613552
cg24843003	0.03180604	9.02802274	1.21110392	67.2982665
cg08319238	0.03180879	1907.94438	1.92981213	1886324.43
cg10805447	0.03181509	417001881	5.62263371	3.0927E+16
cg18009496	0.03182061	3.3265597	1.11019045	9.96765865
cg09436823	0.03182185	0.05558726	0.00397274	0.77778564
cg11675148	0.03184226	9.1442E+10	8.95394125	9.34E+20
cg20146536	0.03184353	5.44181818	1.15850262	25.5617766
cg02451122	0.03184853	1620058.44	3.46028698	7.5849E+11
cg15822350	0.03187015	16374.8902	2.31947257	115602587
cg15755406	0.03187428	1248568.25	3.37639715	4.6171E+11
cg16443152	0.03187432	55532876.3	4.69155573	6.5733E+14
cg26034516	0.03187706	3.76978227	1.12188446	12.6673101
cg11993436	0.0318802	2134.09902	1.9430334	2343952.82
cg18632220	0.03188047	0.13512018	0.02171517	0.84076996
cg08278892	0.03188157	6.2016989	1.17129471	32.8363723
cg10542362	0.03189259	279.14779	1.62844339	47851.5182
cg17428185	0.03189285	29.6684321	1.34114803	656.315216
cg05993512	0.03189451	4.08E-06	4.87E-11	0.34152206
cg01629007	0.03189627	7.57075702	1.1915248	48.1033729
cg19618984	0.03190285	9.10002435	1.21055996	68.4067255
cg07915528	0.03190393	70.9520474	1.44595229	3481.57616
cg25518519	0.03190511	8018754785	7.19260773	8.9398E+18
cg02670133	0.0319085	2120420997	6.40822245	7.0163E+17
cg04876124	0.03191763	0.1973634	0.04481863	0.86910988

cg16512239	0.031918	754705.874	3.22204498	1.7678E+11
cg25083496	0.03192691	7.6536E+13	15.8341727	3.70E+26
cg04451175	0.03192763	6.78524425	1.17990162	39.0198123
cg06971096	0.03192936	0.2089238	0.04997139	0.87348288
cg09065658	0.03195233	31854.0144	2.44609166	414816112
cg11897736	0.0319716	0.05074507	0.00332917	0.77348446
cg23501177	0.03197531	27.8160015	1.33173446	580.994155
cg05375405	0.03197624	162821.018	2.81146665	9429485444
cg10580282	0.03198288	12.8956918	1.24627418	133.436823
cg23528933	0.03199402	3554.75686	2.02082135	6253049.7
cg15320854	0.03199538	8.19293071	1.19836712	56.0129799
cg20722436	0.03200083	3.9446E+10	8.15351415	1.91E+20
cg05177841	0.03200289	27723.675	2.41031326	318880606
cg02866325	0.03201079	9280.52504	2.19297554	39274558
cg24309428	0.03201294	5.16510888	1.15155532	23.1672324
cg19424078	0.03201448	4.6883E+12	12.2713285	1.79E+24
cg02626657	0.0320235	6.55477234	1.17525528	36.5580494
cg05819860	0.032029	250.372539	1.60671471	39015.27
cg18448361	0.03203088	4.5151E+10	8.21648431	2.48E+20
cg08739433	0.03203124	6.91085633	1.18051318	40.4569267
cg22878324	0.03203155	3839873044	6.64911717	2.2175E+18
cg25043378	0.03203714	38.2861348	1.36725272	1072.09742
cg00836119	0.03203889	347936.837	2.98877163	4.0505E+10
cg09421020	0.03204014	47241.7475	2.51798438	886336996
cg18581950	0.03204225	7.62421465	1.19036908	48.8324588
cg05240381	0.03204318	2969278.11	3.59122737	2.455E+12
cg05396397	0.03205534	7.25888012	1.18520153	44.4577056
cg09205065	0.03205869	7590718.6	3.88725587	1.4823E+13
cg01947949	0.03206583	168.094761	1.5511237	18216.3735
cg07562483	0.03207325	7.22666588	1.18452666	44.0890876

cg11844827	0.03207542	3.0422E+11	9.6181735	9.62E+21
cg14679255	0.03207692	7.36766386	1.18644174	45.7523274
cg17296166	0.03208119	6.6292071	1.17571288	37.3785024
cg17701073	0.03210296	30.1039811	1.33773662	677.450005
cg23511157	0.032107	9.09007844	1.20754523	68.4276864
cg02067983	0.03210822	7.68723772	1.19035798	49.6435733
cg16438182	0.0321126	13.950748	1.25246143	155.392706
cg02106682	0.03211343	7.91956371	1.19332161	52.5587478
cg14950855	0.03211613	187200.052	2.81986738	1.2427E+10
cg04569641	0.03211851	0.24497969	0.06767335	0.88683428
cg09673444	0.03212775	67917.7149	2.58421535	1784996746
cg10287032	0.03213547	4.87852547	1.14473685	20.7908139
cg07816439	0.03214107	30.6791042	1.33897323	702.932225
cg10713002	0.03214691	104.317252	1.48602954	7322.92914
cg14839257	0.0321476	10.6976834	1.22383846	93.5094244
cg07640390	0.03215184	34287.5835	2.43454307	482898986
cg14176626	0.03215792	6.08952177	1.16634025	31.7937029
cg01799338	0.03216163	323.242678	1.63566583	63879.6918
cg07350057	0.03216739	6.89842142	1.17868193	40.3740965
cg03773789	0.03216801	640.327183	1.73332172	236550.951
cg25512537	0.03216895	17.0794114	1.27321338	229.110295
cg03047121	0.03217493	30.2283931	1.33646509	683.710896
cg11799329	0.03217669	302.575044	1.62574589	56313.633
cg21236845	0.03218341	36.1253025	1.35666483	961.945396
cg09310612	0.03218355	116978.463	2.69758924	5072662893
cg06911354	0.03218428	3.74809966	1.11890413	12.5553661
cg15822328	0.032197	27.2205564	1.32408745	559.599511
cg22334879	0.03219966	11.7864161	1.23314834	112.654415
cg07840972	0.03221421	4341.29935	2.03582188	9257627.22
cg02328440	0.03222211	7.98250822	1.1926979	53.4254632

cg01731920	0.03223132	16.0600418	1.26539814	203.829084
cg10297426	0.03223352	0.13768551	0.02242716	0.84528296
cg08730245	0.03224427	0.03475046	0.00160518	0.75231207
cg07500501	0.03224955	10068501.8	3.91793759	2.5875E+13
cg05406678	0.03225051	7.50551337	1.18611979	47.4932897
cg22663372	0.03225104	7.47816331	1.18574649	47.1626329
cg06289967	0.03225295	1498052.46	3.33328999	6.7326E+11
cg10006979	0.03225514	4.49069115	1.13559338	17.7583873
cg08854306	0.03225969	1978.80918	1.9010081	2059794.36
cg03185843	0.03226235	65.1968618	1.42402477	2984.94161
cg16903510	0.03226435	392250.566	2.97345151	5.1745E+10
cg01946401	0.0322651	4.88960803	1.14370872	20.904157
cg09718640	0.03227188	429116.696	2.99458644	6.1491E+10
cg14904363	0.03227388	10.152961	1.21650822	84.7364742
cg03366850	0.03228146	5.57575821	1.15631366	26.8863724
cg27307781	0.03228182	14.0484467	1.25023706	157.857146
cg26379859	0.03228271	3.44190864	1.11010826	10.6716935
cg13976657	0.03228408	4.15920429	1.12799853	15.3359954
cg27331241	0.03228512	2.89914875	1.09410987	7.68210183
cg11926764	0.03229043	27442.7868	2.37090018	317645828
cg27298559	0.03229219	1190589252	5.84176055	2.4265E+17
cg23627444	0.03229803	4459.7093	2.03286903	9783712.95
cg07131742	0.03230045	191.598195	1.55837513	23556.5029
cg00911719	0.0323012	5428756.27	3.70249658	7.9599E+12
cg17426063	0.03231562	38.4698111	1.36045198	1087.81962
cg03478631	0.03231574	895.493383	1.77404338	452022.993
cg05483534	0.03232557	18.7540168	1.28026838	274.718295
cg03196669	0.03232739	0.02321996	0.00074035	0.72825694
cg04992603	0.03233031	687.122014	1.73398082	272284.823
cg07850154	0.03233296	8.9076134	1.20229801	65.9949327

cg24498692	0.03233402	0.11713839	0.01643805	0.83473434
cg21527078	0.03233784	0.16546454	0.03185734	0.85940996
cg08728129	0.03233825	19.2657468	1.28292166	289.315406
cg14517217	0.03233873	2.84991936	1.09220269	7.43638562
cg21578906	0.03233885	0.15480607	0.02804185	0.85461279
cg01468567	0.03234394	6.51512665	1.17090363	36.2513826
cg02608453	0.03235393	0.04434408	0.00255575	0.76940259
cg11832221	0.0323558	30719.6166	2.38504867	395671105
cg15614730	0.03236124	0.19384233	0.04313393	0.87112048
cg23609905	0.03236284	219961.431	2.81331063	1.7198E+10
cg07394347	0.0323762	489788.982	3.00641435	7.9794E+10
cg10356463	0.03237625	0.00018818	7.28E-08	0.48641498
cg18307957	0.03237802	6949.75768	2.10255787	22971606.3
cg00506935	0.03238452	27.084544	1.31919104	556.077552
cg23371050	0.03238827	229.446212	1.57827594	33356.3751
cg03555914	0.03239117	159.264145	1.53051492	16572.8982
cg12953420	0.03239785	7.30001807	1.18149973	45.1039153
cg16315376	0.03239958	4.98160398	1.14420414	21.688768
cg25285433	0.03241212	7.9059667	1.18924528	52.557963
cg14896516	0.0324184	0.15758983	0.02899327	0.85656268
cg26069745	0.03241942	7.53909683	1.18442547	47.9878072
cg16604243	0.03242516	3.22464036	1.10303315	9.4270108
cg10224098	0.03242697	3.14652611	1.10075788	8.99437266
cg20802616	0.03242704	5.1972314	1.14800389	23.5288524
cg10091265	0.03242778	14.4797646	1.25084964	167.616936
cg12282391	0.03242881	6.9649018	1.1764765	41.2331712
cg01184591	0.03242935	0.09103676	0.01012938	0.81818371
cg03184776	0.03243127	6.21759338	1.16532027	33.1741139
cg03340649	0.03243558	4.0097598	1.12326382	14.3137999
cg12837296	0.03244124	1628.88653	1.85670625	1429020.52

cg13390441	0.03244879	10.4786667	1.21710943	90.2157634
cg27557378	0.03245078	5.33862043	1.15034538	24.7759227
cg08599266	0.0324547	200422182	4.94360913	8.1255E+15
cg05768041	0.03246	1038.5728	1.78685808	603648.091
cg08651517	0.0324683	6.32582356	1.1665837	34.3019053
cg20170831	0.03246904	6.43555473	1.16825215	35.4515628
cg26687072	0.0324772	8.86107453	1.19976807	65.4448502
cg00568384	0.03248514	4.5921031	1.13563227	18.5688725
cg06313718	0.03248646	101.907495	1.47078484	7060.94948
cg22059438	0.0324895	5.38407797	1.15076463	25.1904645
cg03969182	0.03249239	12.7930071	1.2368657	132.31916
cg00630249	0.03249288	318365737	5.11834464	1.9803E+16
cg16093048	0.03249563	72.2923275	1.42895354	3657.34816
cg13787932	0.03250586	6.1731041	1.16378413	32.7442291
cg04035031	0.03250672	2.7956E+10	7.42086066	1.05E+20
cg06427816	0.0325079	9.28010142	1.20396945	71.5302889
cg12224388	0.03250845	0.24165675	0.06573358	0.88840416
cg05800561	0.03251442	5.80537581	1.15775117	29.1102176
cg27540841	0.03251527	21561279.1	4.08082956	1.1392E+14
cg04360557	0.03251669	125045.978	2.65733159	5884285063
cg20535715	0.03252841	1612248950	5.83654452	4.4536E+17
cg04562491	0.03252924	19.9522918	1.28282525	310.325937
cg22598028	0.03253676	3.71869983	1.11541741	12.3978058
cg09422355	0.03253677	0.14613514	0.02505957	0.8521884
cg20065905	0.03253762	57.825909	1.40133372	2386.1809
cg25246158	0.03253921	1147.11131	1.79645812	732477.054
cg21404792	0.03253923	2.33E-07	1.94E-13	0.28089327
cg00776327	0.03254944	207281.591	2.76569163	1.5535E+10
cg16184803	0.03255258	10.6810172	1.21747536	93.7054919
cg27303986	0.03256356	765450.575	3.07972537	1.9025E+11

cg22448930	0.03256628	3.895E+12	11.0954767	1.37E+24
cg18831134	0.03256711	4.95827439	1.14213326	21.5250582
cg27576694	0.03256947	5.72007208	1.15573853	28.3102309
cg03943115	0.03257403	8.76856277	1.19738693	64.2129051
cg05719612	0.03257463	6.44125207	1.16712521	35.5486523
cg04117301	0.03257705	863.047567	1.75208464	425122.787
cg19710451	0.03257783	9.1419863	1.20148395	69.5605741
cg02372889	0.03258161	6.81371254	1.1724984	39.5963685
cg01352090	0.03258618	8.48670991	1.19398167	60.3227394
cg15134649	0.03259361	5.45205197	1.15089589	25.8275931
cg08721931	0.03259435	26.3591562	1.31142013	529.811235
cg12502759	0.03259765	278467992	5.00735026	1.5486E+16
cg19097500	0.0326055	29.7081298	1.32421789	666.486221
cg13809748	0.03261531	6945254.34	3.68266874	1.3098E+13
cg10482632	0.03262874	17.3238223	1.26593765	237.069195
cg11632438	0.03263101	10.1026193	1.21069745	84.3009265
cg13199010	0.03263444	41.1758183	1.35972704	1246.90321
cg18457249	0.03263806	1686652.2	3.27002198	8.6996E+11
cg22395490	0.03264138	5.84855769	1.15709567	29.5616239
cg01905111	0.0326427	312297.733	2.84375385	3.4296E+10
cg27227156	0.03264612	9.99707611	1.20942481	82.6355882
cg06499652	0.03264842	2532.54763	1.91010132	3357831.04
cg11154879	0.03265659	6.65048097	1.16926649	37.8261907
cg13621317	0.03265784	5.06453849	1.14325732	22.4355003
cg05241277	0.0326622	0.14189858	0.02365494	0.85120526
cg19389852	0.03267361	0.01273515	0.0002324	0.69785971
cg18252633	0.03267467	4.96659023	1.14125382	21.6139636
cg27194187	0.03267988	829288797	5.43254063	1.2659E+17
cg01527459	0.03268512	3.51364657	1.10907681	11.1315214
cg00224202	0.03268802	7.96560023	1.18640245	53.4816723

cg21235989	0.03270197	17535576	3.94583145	7.7929E+13
cg09781028	0.03270282	6.15423043	1.16129227	32.6141431
cg24834740	0.03270409	5.44443658	1.14962721	25.7839144
cg14202186	0.03270704	112.471438	1.47480908	8577.26236
cg12817389	0.03271254	12.5399757	1.23118454	127.723332
cg16400495	0.03271505	5.20907513	1.14534526	23.6910779
cg09503975	0.03271596	18513.7082	2.24325617	152794583
cg17574958	0.03272361	0.14631841	0.02507232	0.85389297
cg26723570	0.03272563	1778.65053	1.84952937	1710487.96
cg19570723	0.03272782	4367.60513	1.99103207	9580947.89
cg25528940	0.03273486	404892.288	2.88725054	5.678E+10
cg26329692	0.03273595	46929.9866	2.41880609	910541630
cg22800400	0.03274084	7.21772366	1.17615971	44.292909
cg25074751	0.03274433	5.15660985	1.1441029	23.241463
cg13835114	0.03274464	29.2501717	1.31924518	648.531871
cg14037250	0.032751	197.72409	1.54298359	25337.156
cg17980610	0.03275134	16.1858639	1.25658383	208.487634
cg11846968	0.03275443	0.20918243	0.04974851	0.8795699
cg05296818	0.03275786	15.4807358	1.25188289	191.434186
cg26574895	0.03275908	16697.2082	2.21938423	125618971
cg23371754	0.03276602	5.25872281	1.14572847	24.1367535
cg10185013	0.03277051	83.9206907	1.4375746	4899.00303
cg09043226	0.03277059	325.860643	1.6065783	66093.9827
cg12385643	0.03277544	7.87063711	1.1841058	52.3153664
cg00658161	0.03277632	0.02261272	0.00069741	0.73318866
cg26924012	0.03277712	19.4006431	1.2748857	295.230351
cg18116600	0.03278165	144.994453	1.5030091	13987.5343
cg06769202	0.03278707	5.39485777	1.14791659	25.3541857
cg18123911	0.03278975	24080956.8	4.01849381	1.4431E+14
cg26879059	0.03279155	309.179675	1.59866498	59794.9365

cg18378494	0.03280078	86.6152576	1.44027817	5208.8569
cg11849105	0.03280151	7671.95357	2.07812877	28323014.6
cg05367052	0.03280933	5059.74966	2.00788028	12750295.4
cg25753841	0.03280992	366480.221	2.84925586	4.7138E+10
cg04167018	0.03282315	225770302	4.80990233	1.0597E+16
cg08474467	0.03282552	0.0504918	0.00325324	0.78365504
cg04480386	0.03282592	3.75749162	1.11413072	12.6724298
cg05048168	0.03282844	8652.18105	2.09596852	35716298.4
cg08145698	0.03283003	6.18037555	1.16028157	32.9204935
cg22001110	0.03283027	3328.29422	1.93857495	5714270.89
cg10109500	0.03283038	4.23750233	1.12508191	15.9601055
cg19276111	0.0328326	6.69178069	1.16780498	38.3453828
cg18043598	0.03283685	85698519.7	4.4382556	1.6548E+15
cg07404418	0.03283737	1746.60211	1.83867875	1659136.45
cg00401745	0.03283954	4.01535734	1.12007309	14.3946808
cg12526849	0.03284663	19.3940363	1.27346882	295.35756
cg07204255	0.03285066	3.37103098	1.10412754	10.2921533
cg23191024	0.03285156	2210.51126	1.87327738	2608455.15
cg04383128	0.03285171	0.14412769	0.02432556	0.85394925
cg16467656	0.03285481	708.154956	1.70709957	293763.44
cg06550200	0.03285577	19.2675589	1.27260864	291.714839
cg07517487	0.03285963	175.028392	1.52312508	20113.2122
cg00672333	0.03287738	58.7354527	1.39296117	2476.63287
cg21505923	0.0328781	12.5547536	1.2285931	128.294582
cg04645049	0.03288581	2971.28408	1.91622602	4607248.29
cg14611399	0.03288624	4.94904952	1.1388914	21.5060813
cg12560320	0.03289643	0.00527326	4.26E-05	0.65293352
cg15456476	0.03290028	32.2849512	1.32619955	785.943617
cg25388707	0.03290058	17214.2906	2.2088513	134156519
cg11972708	0.03290339	2910609.49	3.35045797	2.5285E+12

cg18267049	0.03290346	4.83662082	1.13660291	20.5814191
cg23986385	0.03291959	24196594.5	3.97368754	1.4734E+14
cg03308628	0.03292638	10.2800405	1.20805736	87.4786539
cg03079754	0.03292873	5.99414708	1.15631319	31.0727228
cg26898605	0.03293265	11.0893616	1.21540998	101.17898
cg16353345	0.03293549	3667526246	5.96146189	2.2563E+18
cg21940313	0.03294733	16.2918359	1.2536468	211.721448
cg02497558	0.03294953	6.05929603	1.15709969	31.7302551
cg27536453	0.03294962	5.74072585	1.15204834	28.6063806
cg10457563	0.03295015	10.2063662	1.2070028	86.3046151
cg24960947	0.03295141	3.2314139	1.09964434	9.49583011
cg15899747	0.03295529	1068101.74	3.07676009	3.7079E+11
cg22831269	0.0329558	37562.7441	2.34624389	601369598
cg10054857	0.03295785	0.23578962	0.06249469	0.88962352
cg24363811	0.03296063	1946042.93	3.22857862	1.173E+12
cg04280397	0.03296094	31.2151348	1.32112454	737.54185
cg04896048	0.03296472	0.20546125	0.04798099	0.8798136
cg26818820	0.03296795	8.59948184	1.19013287	62.1368334
cg03247626	0.03297983	652656.647	2.95137566	1.4433E+11
cg01459033	0.03298149	1000768.36	3.05477334	3.2786E+11
cg13075951	0.03298181	0.17481999	0.03518834	0.8685272
cg22290047	0.03298361	257049.157	2.73656726	2.4145E+10
cg06787669	0.03298686	6.06321607	1.15675185	31.7808778
cg25261331	0.03299025	0.04436755	0.00253175	0.7775186
cg08301503	0.03299195	4.82260237	1.13550475	20.4820752
cg01490772	0.03299961	113.590038	1.46530561	8805.46468
cg23318893	0.03300132	7.41421727	1.1755276	46.7625073
cg06191203	0.03300315	215.855803	1.54310097	30194.8664
cg26229968	0.03300879	2028.23028	1.84852448	2225406.34
cg01869405	0.03301408	2445523.46	3.27529398	1.826E+12

cg11404945	0.03301543	31.1487526	1.31959889	735.257355
cg10165801	0.03301613	4.42508831	1.12742974	17.3681835
cg07489950	0.03301802	7464.19762	2.05254668	27143960.6
cg11571761	0.03301818	156.993198	1.50335046	16394.623
cg19046253	0.03302318	31.6568155	1.32113635	758.554533
cg06984883	0.03302408	71295063.1	4.29508712	1.1834E+15
cg21037314	0.03302433	84533.0111	2.49529173	2863725262
cg00303541	0.03303342	10.5681503	1.20916659	92.3659331
cg21793948	0.03303885	452.306736	1.63616632	125037.034
cg24425972	0.03304258	0.17203425	0.03410096	0.86788709
cg07141055	0.03305062	15.9465306	1.24959665	203.499135
cg14046757	0.0330552	1.9645E+12	9.74662382	3.96E+23
cg13284574	0.03305564	5.84043117	1.15252443	29.5964539
cg04898211	0.03305591	1558.04172	1.80632242	1343887.44
cg12910906	0.0330761	10.4109316	1.20707474	89.7935253
cg22205015	0.03307735	302.676043	1.58226389	57899.8153
cg22287064	0.03307913	2.6878963	1.08264875	6.6732507
cg10113467	0.03308036	192356216	4.62669651	7.9973E+15
cg22295573	0.03308205	5.86428666	1.15262018	29.836245
cg11995437	0.03308882	8.39761751	1.18624716	59.447965
cg11502198	0.03308902	16.9723944	1.25516575	229.501301
cg09858237	0.03309675	7.82737163	1.17946978	51.94516
cg13459498	0.03310205	3.6818643	1.11018367	12.2107045
cg01898246	0.03310556	329.059865	1.59155727	68034.2434
cg23128382	0.03310973	1913.22056	1.83249808	1997498.92
cg06244417	0.03311035	0.17704305	0.03601008	0.87042957
cg21807925	0.03312645	223528.288	2.68100155	1.8637E+10
cg08829299	0.0331285	7.1495354	1.17054741	43.668335
cg15679098	0.03313839	88.547003	1.43146173	5477.31846
cg08081524	0.03313907	53.6421866	1.37517995	2092.44194

cg19557190	0.03313962	1.3876E+10	6.47660228	2.9731E+19
cg04958124	0.03314478	160065313	4.52995443	5.6559E+15
cg07017214	0.03314964	8.03842547	1.18130788	54.6989358
cg16296724	0.03315277	878586.608	2.9858869	2.5852E+11
cg20050012	0.0331601	265.781296	1.56204576	45222.553
cg26256999	0.03316206	29968911.1	3.95576019	2.2705E+14
cg09323728	0.03316928	9.20590326	1.19391205	70.9840015
cg11770080	0.03317003	4.22818252	1.12199391	15.9337117
cg25274503	0.03317143	126.162678	1.47135494	10817.9345
cg11830695	0.03317414	6661.41976	2.01920958	21976180
cg12234455	0.03318166	12.3048509	1.22169431	123.933912
cg13503148	0.03318347	6.61910667	1.16271368	37.6813088
cg15477139	0.03318628	0.11660464	0.0161385	0.84249703
cg08402850	0.03319574	12.7456723	1.22489989	132.624848
cg14789568	0.0331998	746809360	5.09368229	1.0949E+17
cg17600693	0.03321088	135543894	4.44126164	4.1367E+15
cg06467910	0.03321164	8.74435194	1.1884478	64.3391244
cg12416929	0.03321685	234.830363	1.54416703	35712.004
cg02874569	0.03322024	5.78845735	1.14995474	29.1370063
cg24242519	0.03322508	13.8113274	1.23226777	154.798145
cg06834434	0.0332281	1702.46636	1.80708904	1603900.89
cg18176922	0.03323294	28.7203333	1.30598506	631.597994
cg19631443	0.03323297	8.9618E+11	8.91894953	9.00E+22
cg08828868	0.03323583	85270.2995	2.465841	2948699436
cg10383568	0.03323689	7.71985459	1.17639723	50.6598908
cg24239961	0.03324473	10.8421563	1.2084719	97.2735519
cg13388277	0.03324934	0.1374672	0.02212303	0.85418807
cg08216000	0.03324978	0.05777697	0.0041865	0.79736684
cg23414001	0.03326298	0.06178112	0.00476057	0.8017755
cg09774842	0.03326801	4.06214817	1.11760824	14.7646082

cg17313042	0.03327087	93.340394	1.4330007	6079.84988
cg20902195	0.03327175	7853818.92	3.52223274	1.7512E+13
cg11906444	0.03327216	8.14399608	1.18095265	56.162008
cg01814945	0.0332738	11.0058775	1.2094709	100.150686
cg24716709	0.03327651	174185.482	2.60321976	1.1655E+10
cg13766687	0.03328107	0.06349263	0.00501579	0.80372532
cg14192174	0.03328685	8.57847268	1.18563479	62.06818
cg25982743	0.03328941	10.3749033	1.20359225	89.4311339
cg05643490	0.03329111	16.323311	1.24755511	213.578126
cg13037201	0.03329325	4.54567513	1.12739813	18.3281858
cg10498429	0.03329367	1809.90854	1.81124441	1808573.64
cg05750276	0.03329423	108489268	4.32819445	2.7194E+15
cg19954000	0.03330164	9.2555019	1.19258669	71.8306822
cg27204544	0.03330951	375.574358	1.59837863	88249.4895
cg19767052	0.0333157	67.1070693	1.39461213	3229.11199
cg00675313	0.03331583	7.68012335	1.17492652	50.202539
cg27049761	0.03331685	4.39062753	1.12409892	17.1493893
cg01814537	0.03331922	152770.957	2.56943509	9083306050
cg13053082	0.0333218	12.1441396	1.21818372	121.065587
cg08572782	0.03332663	20193.3999	2.18873288	186305694
cg10773698	0.03333054	8.34477553	1.1824706	58.8896493
cg27372920	0.03333631	3.51287048	1.1043075	11.1746583
cg01105948	0.03334057	12.6951247	1.22215939	131.870027
cg20884043	0.03334624	29.2990839	1.30544622	657.58076
cg10626682	0.03335377	0.21384129	0.05164445	0.88544073
cg14350197	0.03335615	5.58782972	1.14533143	27.2618388
cg27326750	0.03335639	0.03819579	0.00188738	0.77298806
cg14562523	0.03335884	10.2340036	1.20127604	87.1863136
cg21357361	0.03336746	75.1343537	1.40548699	4016.5232
cg26853048	0.03337168	91669705.7	4.23929798	1.9822E+15

cg14250028	0.03337175	8.26043262	1.18098553	57.7778012
cg07300976	0.03337705	1280.06794	1.75675997	932724.997
cg20910436	0.03338148	84012392.7	4.20633819	1.678E+15
cg08933227	0.03338229	4.60741036	1.12780209	18.8226555
cg12727940	0.03338462	11.8822882	1.2151001	116.195179
cg17141902	0.03338693	27.5597584	1.29824204	585.052908
cg16425829	0.03339815	0.19771129	0.0444045	0.88031064
cg04943986	0.03340552	0.00074245	9.71E-07	0.56755989
cg27274446	0.03340853	11.8572879	1.21452405	115.761624
cg18488855	0.03340934	9.21418197	1.19067721	71.3049251
cg05812599	0.0334138	0.17026447	0.03331609	0.87014978
cg21540749	0.03341461	4496369.56	3.33155986	6.0684E+12
cg08887028	0.03344858	0.04797	0.00291964	0.78815348
cg20512519	0.03345056	67446045.3	4.10746743	1.1075E+15
cg17210546	0.03346913	86.7799878	1.41817892	5310.16657
cg23818870	0.03347	4.46900738	1.12432722	17.763536
cg13517138	0.03347144	17931.9639	2.15230701	149400308
cg21296513	0.03347571	53498.9206	2.34396981	1221062871
cg08986653	0.03347856	0.00791101	9.14E-05	0.68483521
cg07549195	0.03348973	2223.35494	1.82654365	2706372.33
cg13671601	0.0334937	5.44149852	1.14154931	25.9383505
cg03076324	0.03349492	1.4645E+10	6.22815125	3.4435E+19
cg01711077	0.03349637	25.5512571	1.28814969	506.825212
cg24065504	0.03350191	5.74791103	1.14636105	28.8203104
cg08911275	0.03350218	2.97560365	1.08889964	8.13134361
cg13672444	0.03351431	111.823539	1.44500099	8653.63001
cg23426747	0.03351818	3098379.1	3.20951422	2.9911E+12
cg16479401	0.03352592	4.38E-05	4.20E-09	0.45721843
cg26272575	0.03352835	26.8866273	1.29257849	559.262538
cg21880328	0.03352963	14.0473689	1.22876013	160.591614

cg16257334	0.03353046	20456.2815	2.16798186	193017968
cg17004840	0.03353371	11828.6598	2.07702527	67364223
cg15147084	0.03353437	2.0894E+10	6.37234495	6.85E+19
cg03456872	0.03355579	1846245613	5.26190752	6.4779E+17
cg27261378	0.03355972	2.8269E+12	9.30594996	8.59E+23
cg27111970	0.03357915	3.55751543	1.10363552	11.467478
cg00541350	0.03358416	9622.93803	2.03896827	45415584.7
cg16872595	0.03359289	10.109467	1.19673624	85.4000402
cg06710890	0.03359762	0.03222992	0.00135609	0.76600368
cg26341831	0.03359795	11.5838516	1.20937318	110.954683
cg00134776	0.03359931	5.69648711	1.14454686	28.3518014
cg02069772	0.03362275	11.7308529	1.21017306	113.713413
cg08736088	0.03362676	7.16535793	1.16477666	44.0791408
cg19311812	0.03363136	63.6670573	1.37936587	2938.66498
cg16267491	0.03363403	8852188.15	3.45003116	2.2713E+13
cg01550161	0.03363978	27.6649008	1.29296679	591.930699
cg02007493	0.03364647	161791484	4.31504877	6.0663E+15
cg06485521	0.03364789	48.5762607	1.35032133	1747.47525
cg01425188	0.03366409	11.1580849	1.20486408	103.33353
cg03762535	0.03366665	5.379392	1.13880041	25.4108252
cg00268086	0.03366798	4.44792799	1.12218472	17.6299527
cg11055795	0.03367177	336567.011	2.67182366	4.2397E+10
cg22276811	0.03367567	15.0112652	1.23259574	182.815886
cg13443371	0.03367799	564.285304	1.63074262	195259.448
cg08738562	0.03368103	605573.081	2.79401243	1.3125E+11
cg21142512	0.0336914	26.9898955	1.28935734	564.974839
cg08979191	0.03369229	4.63743651	1.12558898	19.1062793
cg14966496	0.03369639	3.2623E+12	9.2195737	1.15E+24
cg24127861	0.03370209	4.29915414	1.11895063	16.5179105
cg09013267	0.03370859	69.9466071	1.38708323	3527.20567

cg18577280	0.03371032	81.6246822	1.40362329	4746.70718
cg15603184	0.03371045	97586.425	2.42261785	3930917269
cg07411238	0.03371306	11.7926688	1.20926491	115.001301
cg27394136	0.03371474	185.569554	1.49510204	23032.5814
cg14347989	0.03372449	40.0401991	1.32833636	1206.93643
cg26024566	0.03372477	107.279907	1.43298079	8031.49526
cg07028533	0.03373663	3.71514603	1.10617111	12.4775542
cg15658824	0.03374106	43.1659781	1.33560935	1395.09481
cg02036832	0.03374377	89266447.9	4.08309918	1.9516E+15
cg16300838	0.0337444	395.508538	1.58334736	98795.1274
cg11662322	0.03375459	1938279.08	3.03961704	1.236E+12
cg21927946	0.03375531	8.39206265	1.17744764	59.8130339
cg13623999	0.03375696	7.96215654	1.17268185	54.0606446
cg08098598	0.03375846	9.09466822	1.18469675	69.8178584
cg21379008	0.03376186	301067.433	2.63334035	3.4421E+10
cg09447457	0.033762	3.1469E+10	6.39355175	1.55E+20
cg12473573	0.03376741	6868338066	5.68499554	8.298E+18
cg26471467	0.03377089	1.2218E+10	5.93938871	2.51E+19
cg20539321	0.03377173	259451.867	2.60178155	2.5873E+10
cg08333931	0.03378685	10.1348448	1.19417957	86.013094
cg11361387	0.03379286	16.8474758	1.2414913	228.626202
cg27639942	0.03379734	4.51944955	1.12243082	18.1974906
cg16509355	0.0337983	7.69437373	1.16909397	50.6404006
cg06235653	0.03380061	5.75025711	1.14328891	28.9213484
cg03211327	0.03380757	8.4889466	1.17780288	61.1835953
cg17421417	0.0338078	5.31461188	1.13634352	24.8561276
cg17450838	0.03380922	7.0373E+11	8.06157339	6.14E+22
cg19351701	0.03381617	9.5476311	1.18832285	76.7108532
cg23894219	0.03382042	4.70677781	1.12571803	19.6796683
cg06826870	0.03382242	0.20609107	0.04792381	0.88627207

cg08231603	0.0338228	4.789E+12	9.31635704	2.46E+24
cg23905308	0.03382339	3.3434E+10	6.37398156	1.75E+20
cg07823935	0.0338247	0.00015697	4.81E-08	0.51198227
cg19691267	0.0338293	238710.645	2.57566806	2.2123E+10
cg20735720	0.03383025	9.98096931	1.19216308	83.5621824
cg03517998	0.03383199	0.21973445	0.05420865	0.8906923
cg02536026	0.0338381	5.59836461	1.14056642	27.479054
cg25532627	0.03384347	14.239549	1.22474808	165.556296
cg26495953	0.03384467	29.1880671	1.29369092	658.536945
cg19406914	0.03385085	17185.0925	2.10430419	140344445
cg18191873	0.03385262	234.254523	1.51623573	36191.7218
cg26211724	0.03385273	5.64734482	1.14117557	27.9470613
cg10420952	0.03385823	7.46286468	1.16563448	47.7802864
cg10337772	0.0338708	191594896	4.27586849	8.5851E+15
cg22817039	0.03387301	2537296.05	3.07515764	2.0935E+12
cg21784254	0.03387537	6.91004345	1.15861489	41.2118825
cg07477282	0.03387668	1249.20775	1.721212	906640.203
cg02452416	0.03388065	10.4521009	1.19563833	91.3707859
cg08804892	0.03388521	10.3206007	1.19442038	89.1769777
cg26014634	0.03388822	6.27936467	1.15005907	34.2855613
cg21627187	0.03388834	7.00330418	1.15964677	42.2941456
cg10541755	0.03389418	3.85301622	1.1080528	13.3980384
cg26266931	0.03389983	3.5075E+14	12.763716	9.64E+27
cg07602841	0.03391724	4.68385926	1.12442672	19.5108646
cg24291087	0.03391825	0.03655968	0.00171844	0.77780398
cg07059148	0.03392468	2954911.69	3.09868115	2.8178E+12
cg08749351	0.0339293	37.5243075	1.31664264	1069.44254
cg17767099	0.03392945	18.1356851	1.2459593	263.975776
cg00720190	0.03394381	19935954.8	3.57593971	1.1114E+14
cg05858889	0.03394462	4.62273885	1.12306186	19.0280831

cg15553385	0.03394709	78574.875	2.34980314	2627458817
cg10210369	0.03395192	4.62152006	1.12297444	19.0195315
cg16408081	0.03395856	19.1208195	1.25041463	292.387602
cg26257814	0.03396219	3.34799194	1.09580764	10.2290307
cg07881863	0.03396936	244510.048	2.55727794	2.3378E+10
cg00337957	0.03397293	8134.36876	1.97630996	33480555.5
cg00990022	0.03398269	15.3157052	1.22915762	190.838685
cg00026327	0.03398275	12.2343551	1.20845677	123.859992
cg24869272	0.03398658	7.75459549	1.16745789	51.508283
cg13892257	0.03400054	0.15790928	0.02866483	0.86989338
cg19658926	0.03400301	1.2693E+10	5.79248016	2.7816E+19
cg10635330	0.03401527	29.3670056	1.29043692	668.317071
cg06686306	0.03401848	2.6546E+16	17.3288685	4.07E+31
cg22397446	0.0340205	578321698	4.57920232	7.3038E+16
cg22613968	0.03402342	8.19979384	1.1719246	57.3728198
cg25934198	0.03403099	648.266422	1.62889444	257996.678
cg10325497	0.03404703	12.9657233	1.21274022	138.619943
cg20893919	0.03404756	22.0061646	1.26199828	383.733707
cg24425705	0.03405401	78.0743117	1.38801351	4391.59856
cg04059461	0.03405543	10.9771849	1.19751139	100.624168
cg12482755	0.03405829	1043104073	4.76810802	2.282E+17
cg02490260	0.0340611	902.065137	1.66819221	487786.423
cg22245862	0.03408192	0.07641247	0.0070827	0.82438386
cg14145801	0.03408448	3.80238337	1.10548418	13.0785402
cg11214576	0.03408924	23.583786	1.26772961	438.733117
cg27369401	0.03409652	6.4781744	1.15047211	36.4778452
cg14835423	0.03409736	1070.5408	1.68760642	679102.418
cg17891715	0.034104	58600.962	2.27776704	1507648802
cg19800427	0.03410705	5.48014164	1.13601696	26.4361831
cg04148483	0.03410887	32532.9756	2.17887243	485753314

cg07782527	0.0341111	3916.07646	1.85895643	8249604.24
cg05564117	0.03411512	11.073567	1.19741119	102.4075
cg03149565	0.03412223	0.28884124	0.09156063	0.91119145
cg25509428	0.03412298	7197.89984	1.9446651	26641997.2
cg19527084	0.0341243	3.69471348	1.10280602	12.3783399
cg08108362	0.03412437	226659889	4.2230679	1.2165E+16
cg23782001	0.03413491	8.79297692	1.17663829	65.7096097
cg11993754	0.03414355	0.01450925	0.00028891	0.72867005
cg04902856	0.03414358	555.285286	1.60409401	192221.745
cg08563839	0.03414848	0.11434443	0.01537557	0.85035229
cg22942704	0.03414855	45.4516844	1.33016649	1553.08048
cg10103630	0.0341515	165.138201	1.46472439	18618.2641
cg22366783	0.03415317	1.9825E+11	6.98595369	5.63E+21
cg01763057	0.03415944	2444.46178	1.79095558	3336427.48
cg12950012	0.03416101	139315.559	2.42212241	8013147800
cg03394422	0.03416106	8.92211725	1.17757484	67.6000993
cg14667731	0.03416151	277891.518	2.55022951	3.0281E+10
cg02124724	0.03417055	14.0989542	1.21835592	163.154711
cg14927519	0.03417286	5.32438575	1.13292033	25.0230159
cg21809927	0.0341733	0.28545551	0.08947613	0.91068808
cg07616094	0.03418394	0.18736789	0.03977639	0.88260206
cg08219107	0.0341845	621.236664	1.61541761	238907.259
cg20224517	0.03419771	21886.5977	2.1054183	227519234
cg17475304	0.0342003	23.0595235	1.26331784	420.908822
cg24383507	0.03420163	3.77475275	1.10399189	12.9065787
cg11525834	0.03420164	2049.51799	1.76460409	2380434.25
cg24770624	0.03420989	4.11952625	1.11113495	15.2731192
cg11601666	0.03421247	621367.855	2.69871527	1.4307E+11
cg24539573	0.03421969	14.9062417	1.2225923	181.741732
cg04507515	0.0342211	4.89727661	1.12542778	21.3104019

cg00373148	0.03422207	26.9399032	1.27756562	568.079143
cg06508879	0.03422542	10.6870602	1.19261914	95.7667471
cg23598419	0.03422782	4.87273925	1.12494568	21.1064305
cg26059292	0.03422856	0.04330355	0.00236814	0.79184363
cg27201457	0.03423566	0.15563703	0.02781342	0.87090623
cg05395366	0.03424623	1365.29581	1.70917534	1090603.53
cg26106626	0.03424856	41130.9234	2.20059863	768769387
cg02811074	0.03425334	16.665143	1.2321768	225.395409
cg27517345	0.03425695	5.23003133	1.13059833	24.1935858
cg00478851	0.03426517	77.521134	1.38071055	4352.48809
cg11837377	0.03426723	111630343	3.95063304	3.1543E+15
cg23530245	0.03426851	229395.716	2.49690136	2.1075E+10
cg00653081	0.0342817	195960.953	2.46586801	1.5573E+10
cg10079801	0.03428347	7.4568E+11	7.57258648	7.34E+22
cg23260573	0.03428613	14.4590429	1.2187101	171.545245
cg00094858	0.03428772	3.22E-06	2.65E-11	0.39210589
cg18296078	0.0342893	5018807454	5.22522198	4.8205E+18
cg26257177	0.03429032	2.6268E+17	19.4721333	3.54E+33
cg06965373	0.03429449	5.63359536	1.13647059	27.9262805
cg04368094	0.03429855	7.36034035	1.15913064	46.7372772
cg13601799	0.03430029	4.50249077	1.11772781	18.1371734
cg18303049	0.03430059	2021.68915	1.75597906	2327605.79
cg09424348	0.03431812	12.5346204	1.20538752	130.345391
cg02208653	0.03431993	312770184	4.24164786	2.3063E+16
cg14794786	0.0343439	1281545318	4.69522207	3.4979E+17
cg00846114	0.03434584	6.29579247	1.14529808	34.60846
cg08871855	0.03435222	6.16987809	1.14352502	33.2895171
cg23659592	0.03436206	1.1573E+11	6.52902886	2.05E+21
cg08245096	0.03436602	11.8141365	1.19939699	116.369994
cg03423942	0.03437121	6517.68956	1.90869671	22256169.4

cg02399524	0.03437251	4.03355368	1.10809679	14.6824316
cg03520966	0.03437339	453.882645	1.56868234	131326.432
cg16911228	0.03437457	68977.8944	2.2702323	2095798703
cg07469594	0.0343754	262.023635	1.50643416	45575.4303
cg25969992	0.03438217	11.8419405	1.19935832	116.922152
cg12834596	0.03438381	213.232853	1.48342208	30650.9188
cg08917718	0.03438391	37.0324854	1.30422731	1051.50764
cg12737497	0.03439391	572.536376	1.59465432	205560.476
cg07948599	0.03440819	668520078	4.4452419	1.0054E+17
cg08347626	0.03441527	0.13362292	0.02069681	0.86269722
cg02737625	0.03441781	2.4457E+11	6.84724407	8.74E+21
cg13041080	0.03443085	61.9954989	1.35324741	2840.16202
cg16065270	0.03443129	356189203	4.23453168	2.9961E+16
cg27475522	0.03443458	0.17115635	0.03333975	0.87866559
cg13641837	0.03444124	221.768736	1.48533886	33111.2136
cg00021325	0.03444956	10.0506946	1.18403082	85.3157365
cg13883696	0.03445328	4.41728393	1.11484505	17.5023402
cg08500346	0.03446502	2606.49973	1.77742726	3822289.1
cg02532538	0.03446763	7.57316939	1.15953401	49.4620201
cg10116014	0.03447003	0.10688519	0.01345298	0.84921312
cg00103299	0.03447042	5.52246492	1.13304268	26.9165665
cg18261462	0.03448066	4.11460547	1.10884807	15.2680774
cg15169374	0.03448102	6.48410778	1.14629937	36.6777257
cg10994379	0.03448372	10.7200278	1.18913692	96.6406763
cg11367633	0.03448544	26.3911975	1.26996574	548.436297
cg07397616	0.03448753	14.2794614	1.21423364	167.927334
cg11142466	0.0344885	29716515.7	3.51192384	2.5145E+14
cg23732024	0.03450762	3.30493358	1.09106101	10.0109763
cg17473165	0.0345198	34.7102667	1.29481933	930.479326
cg05596720	0.03452454	1317.45054	1.68721322	1028723.53

cg17895870	0.03452656	1301.18985	1.6855628	1004468.67
cg02451467	0.03453472	0.00543032	4.31E-05	0.68418707
cg13085065	0.0345362	877824508	4.47401845	1.7223E+17
cg11928161	0.03454546	1122.20623	1.66636438	755745.163
cg01585703	0.03455005	176.122415	1.45626246	21300.4908
cg06990379	0.03455111	2113.9504	1.74449748	2561646.75
cg13485756	0.03458735	3180170.02	2.96069481	3.4159E+12
cg16556677	0.03459048	17.9821366	1.23296089	262.26074
cg22742943	0.03459871	12302.6734	1.97817765	76512730.6
cg00795268	0.03459963	0.0074483	7.91E-05	0.70124431
cg10282371	0.03460703	25368.9994	2.08374402	308860457
cg11863417	0.03461079	155.156516	1.44064178	16710.2917
cg05102288	0.03461146	373316.718	2.53079791	5.5068E+10
cg22378853	0.03462075	5.48267671	1.13095806	26.5790085
cg16249035	0.03462237	0.08837302	0.00930757	0.83907915
cg08233448	0.03463246	641123087	4.3293198	9.4943E+16
cg13548946	0.03463351	40.4096818	1.3064161	1249.94049
cg06044899	0.03463882	239.855931	1.48561317	38725.3349
cg01437577	0.03464644	6.806E+11	7.14886882	6.48E+22
cg09149894	0.03464688	4.44475946	1.11369907	17.738981
cg06192381	0.03465434	33.8809276	1.28940168	890.271258
cg02657892	0.03465491	6188406.69	3.09030424	1.2392E+13
cg17193066	0.03465729	69573.0622	2.23519337	2165544624
cg01868559	0.03470771	62859210.2	3.63521439	1.0869E+15
cg18505959	0.03470773	7.21404288	1.15261412	45.1516372
cg15112775	0.03471811	132126.377	2.33245385	7484555181
cg05375507	0.03471962	2484272.55	2.87927441	2.1435E+12
cg24832710	0.03472226	105.142813	1.39691685	7913.8649
cg16332813	0.03472279	14.7588293	1.21321748	179.541628
cg16197879	0.0347257	4535.13357	1.83016772	11238006.4

cg27398547	0.03472643	7.59017472	1.15660813	49.8100875
cg16361947	0.03473051	13.6727588	1.20645261	154.953731
cg25508545	0.03473407	16019.9491	2.00287832	128134977
cg03803619	0.03473953	133041.357	2.33059163	7594639296
cg15397308	0.03474969	118.042152	1.407629	9898.87935
cg16741710	0.03475237	13.3609693	1.20410786	148.255406
cg10937302	0.03476119	21.3059099	1.24487566	364.648303
cg21245975	0.03476748	3.714187	1.09846494	12.5586029
cg03341758	0.03476771	1520222.81	2.76976715	8.3439E+11
cg15628917	0.03476803	3.80312834	1.10032286	13.1450374
cg22918700	0.0347708	0.0111549	0.00017165	0.72491435
cg26942121	0.0347711	5.27458371	1.12635418	24.7002531
cg05338167	0.03477658	5.91015746	1.13550446	30.761624
cg11884274	0.03477902	9.03869012	1.17050635	69.7970744
cg16463395	0.03478069	79886.0319	2.2415459	2847043236
cg04283162	0.034787	2.85548022	1.07787476	7.56467041
cg14346208	0.03478884	4.38173681	1.11135731	17.2758277
cg24417499	0.03478932	6.56004186	1.14386885	37.6215763
cg04590978	0.03479456	68.1925114	1.35204104	3439.4064
cg21591820	0.03480076	1635946.76	2.77762216	9.6353E+11
cg16920620	0.0348024	17.9472402	1.22892929	262.100866
cg27092787	0.03481527	416126.924	2.51654552	6.8809E+10
cg10464585	0.03482194	11863012	3.19405568	4.406E+13
cg18800391	0.0348279	1.9807E+10	5.4172776	7.2423E+19
cg23652987	0.03482843	12.6769075	1.19839736	134.099081
cg05304729	0.03482991	0.13605891	0.0213392	0.86751274
cg05862039	0.03484094	5.57248497	1.13009725	27.4778021
cg06536868	0.03484358	3.68176415	1.09721909	12.3543122
cg20223364	0.03484718	272.14211	1.49028873	49695.9591
cg18191200	0.03484753	1083.91885	1.64428767	714522.214

cg03463411	0.03486336	8.45102411	1.16382084	61.3666689
cg02930317	0.03487742	6941491.61	3.06062089	1.5743E+13
cg00776119	0.03487953	1740760.63	2.77386724	1.0924E+12
cg15696309	0.03488372	0.02172509	0.00061939	0.7620118
cg17379405	0.03488872	7.77715566	1.15665941	52.2921006
cg04574507	0.03489629	0.26655342	0.07803475	0.91050111
cg15340874	0.03489742	4.5153E+10	5.69504952	3.58E+20
cg13792279	0.03491813	0.19340228	0.04201873	0.89018497
cg08213047	0.03492616	7.4594E+10	5.87977017	9.46E+20
cg07194495	0.03492701	1720976.25	2.76198298	1.0723E+12
cg11582116	0.03493794	168.99205	1.43718081	19871.0648
cg26860970	0.03493978	54.9432913	1.32738428	2274.22104
cg02447095	0.03495257	15965.4999	1.98085882	128680138
cg19872095	0.03495864	14.0768143	1.20525399	164.410742
cg02735733	0.03496765	0.21149599	0.04991183	0.8961914
cg15718222	0.0349746	10.9535923	1.18386652	101.346885
cg10784843	0.03498185	9821262550	5.06072263	1.906E+19
cg27637930	0.03499375	6.58320095	1.14190731	37.9527606
cg12062819	0.03499617	2.47962602	1.06602189	5.76774757
cg05422369	0.03499938	75344.3787	2.20435281	2575257180
cg00249383	0.03500046	5.71268548	1.13049219	28.8677584
cg01594685	0.03500086	25.3042953	1.25532473	510.073086
cg26462404	0.03500442	15439880.5	3.20481077	7.4385E+13
cg21157923	0.03500714	9.7772652	1.17397608	81.4283329
cg03085549	0.03500716	6.17909621	1.13668332	33.5900327
cg22573528	0.03501062	0.34393856	0.12751489	0.92768563
cg22947959	0.03501373	11.0773726	1.1842362	103.617997
cg06875660	0.03502426	1063309.12	2.65114767	4.2647E+11
cg14095744	0.03502654	0.08511013	0.00861251	0.84107124
cg26401236	0.03502665	2.6774E+10	5.40163306	1.33E+20

cg23878101	0.0350322	93028798.5	3.62705058	2.3861E+15
cg02071074	0.03503302	13.6003079	1.20113733	153.994361
cg23001000	0.03503791	2.7239E+10	5.40072541	1.37E+20
cg22718077	0.03503876	0.21161645	0.04993856	0.89673246
cg22377939	0.03503945	811.638521	1.60024637	411659.792
cg03929531	0.03504684	0.25692569	0.07261296	0.90907759
cg24177983	0.03505175	15.0456809	1.20937244	187.181804
cg19472611	0.03505344	19581.8531	1.99943632	191778536
cg18935660	0.03505457	4.77120176	1.11577235	20.4023395
cg13320146	0.03505908	4.44064061	1.11013217	17.7630101
cg05460130	0.035061	6.44326789	1.13945283	36.4347695
cg12073353	0.03506961	41.5869493	1.29829636	1332.1106
cg06545845	0.03507332	117366.959	2.26422202	6083768709
cg03747003	0.03507413	31.9548646	1.27446074	801.212101
cg00155423	0.03507413	5.63911481	1.1287277	28.1729739
cg07614064	0.0350743	447486894	4.0327189	4.9655E+16
cg26392924	0.03507468	631.979038	1.57057208	254300.653
cg20299151	0.0350811	9.63E-12	5.47E-22	0.16950825
cg23312397	0.0350845	11.7874039	1.18836266	116.919603
cg24438687	0.03509361	471639.917	2.49243004	8.9248E+10
cg01733284	0.03509893	0.26744039	0.07842944	0.91195811
cg01389283	0.03510183	0.17310952	0.03387322	0.88467848
cg04940329	0.03510312	0.18334118	0.03784316	0.88824483
cg02296128	0.03510495	214703873	3.8191758	1.207E+16
cg06500727	0.03510796	0.38318158	0.15700069	0.93520686
cg13342158	0.03510856	5872156.16	2.96939883	1.1613E+13
cg00979026	0.03511516	542.91389	1.55194273	189926.784
cg07734926	0.03511719	42.6142736	1.29933441	1397.62043
cg20426415	0.0351196	0.18564095	0.03875924	0.8891443
cg01921126	0.03512012	17.1348779	1.21924177	240.808712

cg23654545	0.0351224	7.31E-07	1.43E-12	0.37320646
cg27143605	0.03512869	157.154244	1.42280469	17358.2899
cg05926928	0.03513002	27.3298677	1.259401	593.07692
cg12585028	0.03513525	15.5916787	1.21098326	200.74633
cg09161542	0.03513837	30.3469798	1.26844563	726.037573
cg26715540	0.03513918	167.638978	1.42883577	19668.3394
cg06257798	0.03514333	20.0039911	1.23204687	324.792561
cg09804380	0.03514469	7.31344454	1.14863769	46.5651365
cg17577122	0.03515438	5.90786005	1.13159064	30.8440253
cg21287489	0.03515457	40.9906857	1.2949014	1297.57858
cg25264370	0.03515825	1.2223E+11	5.90769441	2.53E+21
cg14588779	0.03515868	40.2958788	1.29326173	1255.55238
cg25181693	0.0351601	1248.3099	1.64212043	948942.342
cg00411097	0.03516515	15.8941263	1.2120953	208.418638
cg19176285	0.03517026	4.6921315	1.11345118	19.7728454
cg07234865	0.0351718	4.69129114	1.11342387	19.7662481
cg10641368	0.03517508	1.6666E+15	11.4233193	2.43E+29
cg06719671	0.0351768	5.25055274	1.12212706	24.5678988
cg04918402	0.0351828	4.89595946	1.11663426	21.4666699
cg12819144	0.03518696	0.16318523	0.03020135	0.88172955
cg03056526	0.0351895	10.7671116	1.17936073	98.2996035
cg00830029	0.03519596	8.16194603	1.15681863	57.5866962
cg07102854	0.03520677	1922.54744	1.68916828	2188170.77
cg02713960	0.03520906	14.7076089	1.20484935	179.535938
cg03427905	0.03521709	0.00689435	6.71E-05	0.70836165
cg01303055	0.03521726	8.35439825	1.15842435	60.2507797
cg17465219	0.03522058	174197907	3.7220071	8.1528E+15
cg02015876	0.03522329	6.3660491	1.13675055	35.6512528
cg01574788	0.03522361	6.43580006	1.13760528	36.4093971
cg14618996	0.03522423	5.45840514	1.12469745	26.4908456

cg00474746	0.03522588	0.04031637	0.00203006	0.80066865
cg18756771	0.03522658	8.14092649	1.156234	57.319439
cg10270204	0.03523394	0.16180374	0.02969676	0.88159285
cg27312391	0.0352349	0.02435442	0.00076698	0.77334169
cg24476497	0.03524609	0.11280249	0.01479623	0.85997581
cg06840042	0.03525336	2.13E-05	9.53E-10	0.47555558
cg13033722	0.03525934	11.3264716	1.18249461	108.4901
cg10788217	0.03525992	2140.47181	1.69826659	2697821.17
cg13623690	0.03527102	11.0575475	1.18036534	103.586027
cg06874326	0.03527216	75.0474102	1.34708337	4180.96897
cg15843331	0.03527293	13.2831279	1.19536668	147.604488
cg17379209	0.03527712	1.9961E+11	6.01735811	6.62E+21
cg02910952	0.03529369	145.647798	1.40937652	15051.5356
cg01643624	0.03530139	56.8545458	1.3207319	2447.46066
cg14408814	0.03530373	6952596.15	2.95799088	1.6342E+13
cg16121929	0.03532079	3.4825E+11	6.21606518	1.95E+22
cg13288195	0.0353228	5.17573403	1.1196414	23.9257166
cg06097557	0.03532365	10.3822286	1.17450854	91.7751266
cg21558912	0.03533496	33648508.6	3.2880945	3.4434E+14
cg16740166	0.03534179	1056812.82	2.59127243	4.3101E+11
cg09473613	0.03534386	4.42451095	1.10746034	17.6767478
cg21872093	0.03535507	6.62464068	1.13844677	38.5488943
cg21940708	0.03535838	3185.48093	1.73851276	5836764.06
cg20648939	0.03536377	1022452185	4.14433605	2.5225E+17
cg11864574	0.03536448	7.27518357	1.14567485	46.1983572
cg11731890	0.03537783	3.47980089	1.08911949	11.1181687
cg14286292	0.03538811	13.1063868	1.1924661	144.052207
cg21437596	0.0353883	80.9712696	1.35065807	4854.18676
cg07451103	0.03539449	1698.95466	1.66291267	1735777.82
cg14240300	0.03540166	6.72057744	1.13905655	39.6522555

cg26691604	0.0354063	171.696834	1.42125478	20742.0959
cg15029192	0.03540803	7418936.23	2.94645534	1.868E+13
cg21992238	0.03540807	6.00066885	1.13020486	31.8597345
cg14788768	0.0354155	0.35694796	0.13669529	0.9320866
cg08821874	0.0354161	3.44E-07	3.27E-13	0.36203987
cg15042460	0.03541819	170.697872	1.42024759	20515.9745
cg09819958	0.03542461	7377.50224	1.83604822	29643850.7
cg06971353	0.03542974	65.7186361	1.33033978	3246.49327
cg19227170	0.0354345	13.777205	1.19580651	158.730845
cg13763617	0.03543635	264.793443	1.46270263	47935.6267
cg01662334	0.03543675	8.06759946	1.15293769	56.4524534
cg27382247	0.03544238	26318.4389	2.00064071	346219199
cg01015662	0.03545513	16.3830703	1.20965869	221.884896
cg23666180	0.03545903	63.9734335	1.32708034	3083.91292
cg23202177	0.03546093	5.03660432	1.11628039	22.7249203
cg22758697	0.03547091	1396.43717	1.63616601	1191833.07
cg05096161	0.0354714	5.77161207	1.12656968	29.5689707
cg27566947	0.03548619	26.178419	1.24823105	549.024657
cg19803052	0.03549116	4.32607369	1.10454192	16.9435974
cg15699226	0.03549117	6.82850949	1.13930349	40.927235
cg23130010	0.03549138	0.00420181	2.56E-05	0.68970924
cg09254001	0.03549146	202898.226	2.29236899	1.7959E+10
cg13862711	0.03549254	4.20729742	1.10244532	16.0564441
cg14636712	0.03549552	183021.938	2.27581345	1.4719E+10
cg20229931	0.03549603	6.65973794	1.13731644	38.9971583
cg22871548	0.0355003	143307.917	2.23770308	9177785623
cg23865240	0.0355065	4.47219086	1.10690668	18.0688142
cg18568145	0.03550746	47.7458316	1.29968578	1754.01198
cg11690979	0.03551364	4.7159393	1.11083483	20.0210534
cg12078933	0.03551372	9.73828251	1.16678713	81.278019

cg14971941	0.03551949	30.9932645	1.2618939	761.222829
cg13085976	0.03552093	8.75241525	1.1582856	66.136342
cg12782992	0.03552146	4.70718789	1.11062681	19.950552
cg26579311	0.0355315	1.9956E+10	4.97887201	8.00E+19
cg23873872	0.0355317	3.8605E+11	6.08410669	2.45E+22
cg03237964	0.0355331	476.225344	1.51781324	149419.291
cg04204002	0.03553537	39388.9986	2.04613727	758254704
cg06704455	0.03553817	4.94532109	1.11419478	21.9496637
cg19552441	0.03553937	5.19824695	1.11794952	24.1708331
cg03237606	0.03554191	9.68481473	1.16597375	80.4440377
cg04984818	0.03554216	3.51172444	1.08866063	11.3278722
cg26667761	0.03554696	3361.13587	1.73142623	6524814.14
cg13787438	0.03555438	12.4797426	1.18595038	131.324192
cg15242360	0.03556719	0.19731168	0.0434394	0.89623486
cg04543413	0.03556808	3.42098616	1.08655957	10.7708281
cg26817935	0.0355779	5.04455411	1.11533007	22.8161392
cg09409749	0.03557809	48.866911	1.29992317	1837.01241
cg10111008	0.03559176	294135995	3.7203644	2.3255E+16
cg09013975	0.03559958	8.08905143	1.15115948	56.8407371
cg12625296	0.03560472	9925190.41	2.95774329	3.3306E+13
cg00532413	0.03560731	10.0376165	1.16790642	86.2686794
cg09569536	0.03560774	26.6614909	1.2472586	569.917979
cg09067228	0.03561127	50.7877464	1.30244826	1980.42045
cg18154114	0.03561239	4409.57209	1.7586038	11056683.7
cg20957975	0.0356134	9797078.55	2.95306954	3.2503E+13
cg04039784	0.0356169	5276423359	4.50635586	6.1781E+18
cg20439022	0.03561745	8.94761982	1.15877592	69.0900619
cg12659606	0.03561945	6.33137866	1.13211428	35.4084005
cg08208505	0.03562382	5.6076E+11	6.16156941	5.10E+22
cg21153026	0.03562529	2072760.55	2.65777576	1.6165E+12

cg20929407	0.03563263	185956.246	2.25916225	1.5306E+10
cg15104158	0.03563333	4.48848265	1.10611266	18.2137654
cg13840445	0.03563849	11.7223774	1.17970744	116.481534
cg00594128	0.03564133	5.40861638	1.1199775	26.1193918
cg24298175	0.03564501	15314236.7	3.0351096	7.7271E+13
cg00766289	0.03564552	0.21560424	0.05152637	0.90216315
cg11985218	0.03564615	26.5015335	1.24595995	563.686882
cg16855929	0.03565467	30.7884772	1.25837318	753.298263
cg26764215	0.03566034	592160.821	2.43740064	1.4386E+11
cg23849169	0.03566415	5.19188504	1.11669483	24.1387973
cg21576886	0.03566577	0.21317448	0.0504019	0.90161997
cg22137448	0.03567362	8052.00733	1.82620973	35502396.6
cg21300742	0.03567857	4632133.49	2.79378703	7.6801E+12
cg23620049	0.03567922	7.08109653	1.13999023	43.9845245
cg00813378	0.03568637	3.42245414	1.08579128	10.7877016
cg24441912	0.03568814	912654.305	2.5042751	3.3261E+11
cg01814969	0.03570268	2849.37181	1.70150211	4771618.95
cg26933795	0.03570489	573249.03	2.42485204	1.3552E+11
cg24303559	0.03570501	0.14891478	0.02518413	0.88053927
cg24598948	0.03570661	0.20070456	0.04484365	0.89828364
cg01651570	0.03570814	3.74186475	1.09213327	12.8203692
cg05713474	0.03570883	9.42E-10	3.56E-18	0.24957459
cg19565710	0.03571867	1.538E+11	5.57910361	4.24E+21
cg14400246	0.03572643	14704.386	1.89649624	114009701
cg13285637	0.03573256	30.0301027	1.25458866	718.806965
cg05827233	0.03573593	0.14440935	0.02372477	0.87899963
cg11733478	0.03575499	144136.873	2.20457727	9423774134
cg26505878	0.0357552	65.2072289	1.32051273	3219.94829
cg21779231	0.03576057	174.128932	1.40951831	21511.5226
cg24791380	0.03576404	193.914018	1.41951444	26489.795

cg21041775	0.03576505	3.20586726	1.08055242	9.5114172
cg14511745	0.03576786	859494.928	2.48055287	2.9781E+11
cg04936446	0.03577224	1010.6109	1.58379362	644865.832
cg07731871	0.0357796	6.9001151	1.13690214	41.8783522
cg14248370	0.03578221	2512597226	4.21019759	1.4995E+18
cg18223453	0.03578864	6.53366902	1.13269139	37.6879628
cg13897241	0.03579042	9.90736521	1.16440631	84.2969368
cg04632980	0.03580521	1048.79786	1.5858656	693612.977
cg25225632	0.03580553	503910.216	2.38806651	1.0633E+11
cg00689580	0.03580678	3.86353537	1.09373318	13.6476664
cg17677842	0.0358111	4166.28739	1.73730145	9991329.1
cg10459387	0.03581656	59.3957931	1.31067554	2691.63506
cg04337534	0.03581939	15.7635445	1.20037391	207.009942
cg03541791	0.03582126	125.106564	1.37681613	11368.0047
cg07685728	0.03582469	10.2806147	1.1667976	90.5821524
cg15816080	0.03582576	205632.392	2.24748758	1.8814E+10
cg17493098	0.03583926	23.2954516	1.23143828	440.68637
cg11658047	0.0358425	105.587182	1.36075829	8192.97092
cg04882359	0.0358454	3232.16944	1.70593013	6123884.64
cg13286281	0.03585293	7.34476304	1.14078618	47.2880415
cg01040499	0.03585411	15.2553084	1.19719994	194.390617
cg02203542	0.03585435	452959389	3.73022302	5.5003E+16
cg02707071	0.03585497	6.88196767	1.13586964	41.6962277
cg22009551	0.03585626	6666322.89	2.82262753	1.5744E+13
cg14334286	0.03585792	4.25344303	1.10031523	16.4423585
cg07913620	0.03586602	41646875.1	3.18292819	5.4493E+14
cg22508569	0.03586815	1673.69774	1.63192542	1716539.3
cg10959651	0.03588164	25.4965481	1.23795488	525.119269
cg08708747	0.03589147	13.9767398	1.18970665	164.199516
cg21803680	0.03589809	52.8503229	1.29845731	2151.13475

cg10455560	0.03589961	881175.352	2.46215799	3.1536E+11
cg09659004	0.03590278	5.84609938	1.12321897	30.427618
cg11756870	0.03590658	5.27274522	1.11557949	24.9214354
cg15320998	0.03590766	11.0044551	1.17088939	103.423973
cg23025703	0.03591031	12.50532	1.18073866	132.445081
cg02026377	0.03591268	1588.86576	1.62364485	1554831.64
cg00500522	0.03591285	17289.2635	1.89957375	157360898
cg11110213	0.0359189	543.724874	1.51280236	195423.241
cg04347874	0.03592281	8.11320539	1.14746263	57.3649196
cg04329454	0.03592928	8.12394805	1.14748405	57.5158602
cg26518580	0.03592961	6.46856596	1.13043714	37.0143054
cg18982073	0.03593796	20.0894922	1.21762124	331.455861
cg11980016	0.03594054	11.2351532	1.17201858	107.701934
cg21962450	0.03594379	13.9469821	1.18871337	163.637691
cg06755309	0.03594737	321824.537	2.29719052	4.5086E+10
cg17829017	0.03596851	0.0682009	0.00554546	0.83876949
cg16158863	0.03596961	6.3818862	1.1290143	36.0743628
cg06811800	0.03597504	0.14336426	0.0233392	0.88063483
cg00543443	0.03597955	143.944957	1.38416381	14969.4353
cg24542360	0.03598022	802.938003	1.5488605	416247.58
cg04254119	0.03598212	0.15720446	0.02789241	0.88602047
cg05445638	0.03598258	97.8423573	1.34954809	7093.57969
cg00953066	0.03598743	1009857100	3.87877158	2.6292E+17
cg20859525	0.03598866	5336949.21	2.75287058	1.0347E+13
cg09097040	0.03599032	6.0548E+11	5.89029087	6.22E+22
cg02554391	0.03599098	0.00635233	5.62E-05	0.71844919
cg10257332	0.03599233	12.8403746	1.18154692	139.541831
cg12020230	0.03599252	0.06690203	0.00534122	0.83798811
cg02191862	0.03599298	9.30028005	1.15689186	74.7651631
cg19518003	0.03599497	748.160411	1.54095989	363243.719

cg26173173	0.03599857	22238.365	1.92294855	257180503
cg08023416	0.03600433	20.7101209	1.21881963	351.905319
cg23874600	0.03600516	10.7358516	1.16762557	98.7118758
cg06639387	0.03600533	27365012.4	3.05893357	2.4481E+14
cg24354901	0.03601075	28739.351	1.95420336	422653196
cg16884042	0.03601274	2240857.91	2.59643912	1.934E+12
cg16345031	0.03602945	14225600.9	2.92517236	6.9181E+13
cg17527574	0.0360408	182451.295	2.20068529	1.5126E+10
cg07064364	0.03604442	21.5951561	1.22139689	381.81755
cg06321304	0.03605365	9128.92385	1.80971632	46049897.4
cg03684845	0.03605479	9.49153171	1.15762118	77.8226731
cg10776186	0.03606578	0.12801726	0.01873059	0.87495488
cg25003924	0.03607207	0.07686242	0.00697919	0.8464926
cg15322516	0.03607209	5.28920865	1.11426056	25.1069895
cg04203587	0.03607373	13.0393349	1.18149133	143.906476
cg05204981	0.03607601	77.1667134	1.3260439	4490.57656
cg11858733	0.0360858	9.45027967	1.15688784	77.1965807
cg21748751	0.03609185	15.5475451	1.19476967	202.320299
cg09571713	0.03609206	5.30396135	1.11427543	25.2469051
cg26994334	0.03610586	40608.9474	1.98867352	829239486
cg05804949	0.03610625	136.189457	1.37484423	13490.6688
cg09896999	0.03611025	0.01778596	0.00041066	0.77032193
cg25883149	0.03611258	3.9871671	1.09368672	14.5356995
cg19501190	0.03611584	6.18846702	1.12523163	34.0348806
cg06641959	0.03611893	1477727.62	2.50774588	8.7077E+11
cg24176760	0.03612214	70.7489995	1.31728653	3799.79664
cg09118552	0.03612259	5.03339595	1.11022273	22.8198127
cg03004714	0.03612601	5.09760956	1.11110256	23.3872409
cg25098077	0.0361266	1914686.3	2.54872068	1.4384E+12
cg09398924	0.03612685	20.7939215	1.21686294	355.329396

cg18840832	0.03613304	391.30144	1.47095076	104093.774
cg09173344	0.03613746	10304.9702	1.81694796	58445488.7
cg13884871	0.03614307	913.379495	1.55327405	537099.106
cg18558763	0.03614856	744496.963	2.39409037	2.3152E+11
cg27611781	0.03615388	22.7452128	1.22342346	422.866425
cg18235443	0.0361543	49603454.7	3.13812323	7.8407E+14
cg15496079	0.03615664	28115503.8	3.02460635	2.6135E+14
cg01268541	0.03615845	4.54620824	1.10263331	18.7442272
cg10171063	0.03616184	3.22975596	1.07855579	9.67156602
cg18796438	0.03616217	0.02140993	0.00058736	0.78041133
cg18991611	0.03616243	2.74083498	1.06719348	7.03918877
cg04411541	0.03616426	6.91043115	1.13276502	42.1570741
cg27529569	0.03617812	1.0377E+10	4.41815974	2.4374E+19
cg26843035	0.03618319	8.4777E+10	5.05507183	1.42E+21
cg22276896	0.03619043	5.70110649	1.11854266	29.0580022
cg24350628	0.03619557	1973.41582	1.6292622	2390265.97
cg11366901	0.03621126	14751402.9	2.88819677	7.5342E+13
cg23622514	0.03621187	0.00051694	4.35E-07	0.61494311
cg11094694	0.03621624	3463375032	4.09933679	2.9261E+18
cg25565132	0.03621725	43.0810587	1.27338706	1457.51255
cg08158952	0.03622923	3.58800828	1.08542932	11.8605635
cg05010623	0.03623167	1380036.96	2.47676043	7.6895E+11
cg18816996	0.03623206	8.2204346	1.14469611	59.0336108
cg20198611	0.03623788	0.02210276	0.0006238	0.78315039
cg27115340	0.03624077	331.946026	1.45082633	75948.5556
cg04346128	0.03625241	11.7456509	1.17090863	117.823297
cg08093097	0.03625988	546.684918	1.49707229	199632.577
cg23085143	0.03626189	0.22155542	0.05405724	0.90805238
cg02221303	0.03626257	1191.05047	1.57343544	901594.8
cg03249020	0.03626451	5.69898757	1.1177923	29.0558982

cg18226566	0.03626548	476804.105	2.30840152	9.8485E+10
cg15782984	0.03626805	0.03373148	0.00141329	0.80508141
cg04945511	0.03627335	162427.364	2.15368613	1.225E+10
cg00541476	0.03627614	296.619682	1.43894421	61144.2993
cg21304158	0.03627824	6.57384097	1.12790711	38.3146668
cg22295389	0.03628523	197.457783	1.40165609	27816.792
cg25278941	0.0362968	4.7978984	1.10526895	20.8273553
cg08695703	0.03630241	6.74928267	1.12954598	40.3284304
cg26008908	0.0363074	6.06697611	1.12184141	32.8105191
cg16414271	0.03630838	182841.908	2.16543789	1.5439E+10
cg05007549	0.03631316	6.83722159	1.13036209	41.3563049
cg00013475	0.03631755	11.3314811	1.16729324	110.000179
cg09687332	0.03632348	3508455.94	2.61135109	4.7138E+12
cg05636499	0.0363354	83606258.8	3.19229146	2.1897E+15
cg14839898	0.03633769	0.04463018	0.00242755	0.82052088
cg21410954	0.03634386	31.4289533	1.24512665	793.316173
cg05876083	0.03635081	3.77E-09	4.88E-17	0.29151404
cg20724257	0.03636163	9.15078337	1.1509394	72.7552089
cg19039028	0.03636578	6.0764071	1.12136028	32.9267266
cg00036011	0.03636672	3.156869	1.07569703	9.26452483
cg22021322	0.03637256	1252.45459	1.57230823	997668.566
cg09651522	0.03638076	6.38357819	1.12471965	36.2313138
cg01292626	0.03638383	63.8584233	1.30145834	3133.32982
cg21271767	0.0363902	2407.82262	1.63775328	3539977.51
cg12828075	0.03639901	4.3730719	1.09791784	17.4182049
cg12570246	0.03640147	0.20412315	0.04607533	0.90430725
cg14112356	0.03640287	32.8415106	1.24732405	864.702978
cg26121931	0.03640328	2.70085612	1.06490346	6.85003295
cg01760189	0.03640767	7.72467266	1.13808694	52.4305883
cg22486630	0.03640932	8.62150874	1.14600331	64.860557

cg22625098	0.03641355	26.5824597	1.23052666	574.247746
cg14022550	0.03641613	74.3470565	1.31314918	4209.335
cg09138133	0.0364225	5.67183399	1.115916	28.8280667
cg17457440	0.0364238	32728.4504	1.92881687	555341196
cg02402637	0.03642497	90575.9786	2.05682211	3988681303
cg19274914	0.03642658	28.8114661	1.23653748	671.310483
cg06644428	0.03643158	4.00215987	1.09152723	14.6741952
cg16198723	0.03643683	7.93800475	1.13970549	55.2878968
cg20276377	0.03643874	4.59398172	1.10101483	19.1683776
cg12146178	0.03645251	1225128.82	2.42006714	6.2021E+11
cg08881995	0.03646916	5339338297	4.0969049	6.9586E+18
cg18780769	0.03647147	4.99587844	1.10656634	22.5551786
cg03101763	0.03647173	10.4227583	1.15899205	93.7313515
cg06398287	0.0364783	356.206482	1.44724446	87672.167
cg25879745	0.03647902	11.0144143	1.16292547	104.320805
cg19632842	0.03648213	488.228469	1.4760628	161488.413
cg18639233	0.03649823	3.37238023	1.07935129	10.5368369
cg10439691	0.03650201	148067.97	2.11197828	1.0381E+10
cg01951603	0.03650468	6774.84972	1.73987355	26380416.4
cg24416238	0.03650685	13.5291824	1.1776413	155.428293
cg06185555	0.03650963	267.513041	1.42016053	50390.942
cg02273392	0.03651786	11.8811715	1.16791893	120.866468
cg05297121	0.03651966	12.1227163	1.16936778	125.674961
cg17896947	0.03652368	2075.0928	1.61413725	2667685.24
cg06888626	0.03653667	6012.63941	1.72448242	20963874.3
cg16630572	0.03653694	16.0272377	1.18973712	215.906811
cg05527530	0.03653845	5.02582911	1.10638413	22.8301886
cg14889011	0.03654294	4072.8644	1.68248756	9859344.45
cg12452106	0.03654344	20264.2754	1.86019015	220752087
cg12019109	0.03654378	5.13456644	1.10781968	23.7978915

cg00885546	0.0365448	31.6475682	1.2413544	806.835315
cg09369294	0.03655178	7831385.82	2.6989609	2.2724E+13
cg04983381	0.03655463	3115851.51	2.54723176	3.8114E+12
cg14074581	0.03655542	11.8875182	1.16741513	121.047848
cg06320150	0.03655563	0.11093501	0.01412043	0.87154383
cg16501237	0.03657687	5.32514746	1.11004241	25.546047
cg12616484	0.03658033	92.6879212	1.32663941	6475.79944
cg09470142	0.03658984	4032.96404	1.678182	9691915.97
cg23242017	0.03659048	6.80E-07	1.12E-12	0.41252232
cg17400113	0.03659384	9.96665118	1.15411068	86.069852
cg26162554	0.0365941	3.94355981	1.08929533	14.2768113
cg19906093	0.03661672	39.9518806	1.25792094	1268.88162
cg23766305	0.03661715	1259102.82	2.39637338	6.6156E+11
cg07109238	0.03661841	4.45937396	1.09747506	18.1197886
cg00630431	0.03662948	1462.33954	1.57303682	1359432.22
cg18800100	0.03663014	999.688354	1.53624794	650530.934
cg06458795	0.03663531	5.5496E+12	6.19171054	4.97E+24
cg04916289	0.03663615	9.36497372	1.1490961	76.3232359
cg15338603	0.0366431	47364505.3	2.99628411	7.4873E+14
cg14031178	0.03664538	8.26735771	1.14011994	59.9491343
cg25886284	0.03665056	4.55529953	1.09866172	18.8873002
cg09225691	0.03665185	1.9168E+12	5.78243672	6.35E+23
cg04962480	0.03665196	116.126957	1.34314876	10040.1911
cg01612730	0.0366542	7.53775397	1.13349848	50.1259915
cg05529874	0.03666295	0.14665343	0.02422525	0.88780209
cg14452947	0.03667254	7.40914528	1.13208145	48.4907105
cg01377358	0.03667545	6.56976795	1.12365026	38.4121753
cg08074477	0.03668131	21.8010093	1.2101889	392.735388
cg22017954	0.03668308	101480.79	2.04106256	5045583096
cg12416067	0.03668798	569505.395	2.27028211	1.4286E+11

cg22678932	0.03668867	5706.34557	1.70759436	19069154
cg04521765	0.0366894	12.974148	1.1718064	143.648744
cg15042811	0.03670278	3.73431389	1.08482372	12.8547154
cg04241844	0.03670684	30.0833494	1.23401843	733.382814
cg10164393	0.03670907	6.09890113	1.11814766	33.2662637
cg05708074	0.03671207	0.00088889	1.22E-06	0.64803526
cg07028390	0.03671755	49803157.8	2.98590869	8.3069E+14
cg19478371	0.03672168	196.256529	1.38505301	27808.7735
cg13570585	0.03672788	4.60553344	1.098762	19.3043975
cg13812587	0.03673097	95.7496938	1.32477969	6920.39888
cg21281951	0.03673342	5.54604357	1.11137324	27.6762102
cg09231767	0.03673558	0.19789934	0.04327619	0.90498137
cg00326648	0.0367359	8.35494521	1.13977259	61.2447693
cg01792640	0.0367408	6.68400083	1.12415391	39.7417709
cg24441185	0.03674603	20135.3129	1.84089935	220235193
cg21851937	0.03675798	15.1350026	1.18192757	193.809087
cg04837533	0.03675924	43.2820352	1.26082001	1485.80651
cg11441617	0.03675945	27154.3128	1.87383418	393501576
cg25968378	0.03676053	747108261	3.51370443	1.5886E+17
cg26674967	0.03676605	18198.0684	1.82767667	181197090
cg23406136	0.03677865	25.5909528	1.22033316	536.654156
cg05168491	0.03678615	14.5704283	1.17872311	180.107931
cg11931731	0.03678619	4.17606925	1.09169535	15.9747445
cg08223837	0.03679723	36.7941764	1.24743571	1085.2755
cg06299833	0.03680177	3.69268517	1.08337362	12.5865384
cg03221247	0.0368081	181.156558	1.37514417	23864.9147
cg08024174	0.03680843	17.870206	1.19320319	267.636113
cg00106250	0.03681203	783164.747	2.2961155	2.6712E+11
cg27124616	0.03681704	27.9726523	1.22623904	638.105013
cg18988094	0.0368173	29250.7818	1.87685163	455874202

cg08430355	0.03681845	177539.107	2.09578617	1.504E+10
cg16791210	0.03682358	232.716925	1.39582216	38799.4751
cg26738010	0.03682669	0.10904545	0.01361728	0.87322178
cg10169261	0.03682995	34515.3128	1.89471736	628751731
cg13673567	0.03683677	1.3284E+10	4.15717201	4.2449E+19
cg04793634	0.03684399	0.01497577	0.0002899	0.77363222
cg12302189	0.03684513	533.529222	1.46752831	193967.931
cg03386722	0.03684712	40449133.1	2.91465699	5.6135E+14
cg26243475	0.03684813	4.7306E+12	5.94359407	3.77E+24
cg05118960	0.03685053	6.24891861	1.11838103	34.9156351
cg21515384	0.03685229	14.5221214	1.17744421	179.109982
cg07744449	0.03685361	16.9577582	1.18862036	241.93222
cg03687929	0.03685393	0.15819293	0.02800632	0.89354828
cg08049198	0.03685655	4559.49739	1.67222335	12431961.6
cg04264633	0.03685828	6.27081137	1.11854052	35.1557001
cg13906792	0.03686144	9.45218352	1.14686006	77.9029422
cg18903398	0.036869	0.10796181	0.01334988	0.87309779
cg13151171	0.03687128	9.3664533	1.14609724	76.5471238
cg10386659	0.03687243	806.594729	1.5036814	432668.156
cg18875012	0.03688563	13.9829549	1.17421349	166.514036
cg13913015	0.03689113	2.78018487	1.06420237	7.26311846
cg08353062	0.03690138	94944713.4	3.05537106	2.9504E+15
cg00897115	0.03690164	15.2734356	1.1802848	197.645377
cg04750536	0.03690366	13.4257579	1.17103701	153.924234
cg05945782	0.03690834	3.83680435	1.08514458	13.5659966
cg06374165	0.03691289	3.17081486	1.07261678	9.37340072
cg15276815	0.03692364	0.09573416	0.0105676	0.86727621
cg12516270	0.03692611	12.6396264	1.16641877	136.966379
cg12129480	0.03692757	0.24549837	0.06563044	0.91831542
cg25194201	0.03693363	303028587	3.26850332	2.8094E+16

cg09120934	0.03693594	31.8578519	1.23351214	822.791032
cg19595886	0.03694273	0.21678261	0.05155666	0.91151561
cg01189638	0.03695526	15.1569035	1.17888	194.872865
cg01792666	0.03695803	39749.0504	1.89828761	832322248
cg16721321	0.03695951	12.0085854	1.16231924	124.067569
cg20778688	0.03696648	26.4050341	1.21894487	571.991272
cg26422458	0.03696693	5.63285331	1.11020361	28.579475
cg05465498	0.03697133	3.93E-06	3.27E-11	0.47117531
cg00402068	0.03697214	1.35E-05	3.60E-10	0.50776789
cg00437134	0.03697437	2442632226	3.6932838	1.6155E+18
cg12538674	0.03697874	4.27172105	1.09169329	16.7149518
cg24575275	0.03697895	10.5825248	1.15319825	97.1123834
cg06898463	0.03698363	133.22049	1.34373495	13207.7379
cg24220907	0.03698923	23044848.5	2.78264579	1.9085E+14
cg18013306	0.03699087	2.8676E+11	4.91547075	1.67E+22
cg25753024	0.0369973	0.0215967	0.00058784	0.7934479
cg18664866	0.03699802	164.407981	1.36041757	19868.8878
cg07566700	0.0369991	7.33977943	1.12775926	47.7693813
cg26837270	0.03700766	2081408.83	2.40349299	1.8025E+12
cg13629565	0.03700933	42977306.2	2.88426225	6.4039E+14
cg10920316	0.03701957	5.78162734	1.11144478	30.0754616
cg06241292	0.03702398	5.52256764	1.10833948	27.5175195
cg11826475	0.03703286	18.6717385	1.19251052	292.352826
cg24616828	0.03703798	2587.22604	1.60397063	4173230.14
cg18843739	0.03704306	5.72826517	1.11059809	29.5453614
cg09667582	0.03704335	14.1265532	1.17250618	170.199107
cg09036188	0.03704511	6.96573831	1.12370792	43.179824
cg00467202	0.03704896	351.352504	1.42207894	86808.5296
cg13374867	0.03704933	1503.68784	1.55184056	1457029.26
cg24328964	0.03705795	145445.984	2.04124271	1.0364E+10

cg08880849	0.03706018	3.28048553	1.0739	10.0210311
cg26269802	0.03706601	7.06068948	1.12439432	44.3379469
cg00747944	0.03706733	4.29201456	1.09130573	16.8801358
cg21443261	0.03707109	333729178	3.24389233	3.4334E+16
cg15190401	0.03707524	3769.11645	1.6381705	8672014.75
cg02114946	0.03708125	10.1913841	1.14922016	90.3780792
cg20437660	0.03708587	0.21963028	0.05282123	0.91322106
cg10799705	0.03709203	5075.81803	1.66647355	15460148.5
cg21597684	0.03709628	245.83662	1.39009717	43475.8413
cg00520612	0.03709846	4.43357459	1.09318016	17.9811017
cg10813183	0.03711041	0.02190823	0.0006031	0.79584062
cg25227227	0.03711071	2.7421E+13	6.35491266	1.18E+26
cg10542419	0.03711332	2.75E-10	2.83E-19	0.26840367
cg05236474	0.03711556	547083208	3.3266574	8.997E+16
cg04892403	0.03711603	216.402872	1.37880325	33964.3841
cg09487570	0.03712313	18391934.3	2.71464565	1.2461E+14
cg15083037	0.03713549	40.5530737	1.24710686	1318.69356
cg16459276	0.03714209	14.7718906	1.17410693	185.850832
cg11931596	0.03714571	3.00901642	1.06784768	8.4789057
cg13841734	0.03714657	239121352	3.15679164	1.8113E+16
cg16841327	0.03714957	10352.1023	1.7345169	61784363.5
cg02271343	0.03715114	1187.41569	1.52451862	924853.266
cg27025247	0.03715904	2067862.22	2.37642427	1.7994E+12
cg05096209	0.03716399	68.7136733	1.28618283	3670.99358
cg09080114	0.03716695	24.6774788	1.21009778	503.246903
cg24777101	0.03717242	9.14858372	1.14066901	73.374996
cg01931502	0.03717264	393154.098	2.1509598	7.1861E+10
cg24396358	0.03717601	16.2838364	1.18039235	224.639993
cg11616676	0.03718597	18.6229215	1.18967532	291.519207
cg16823406	0.03719057	112.864682	1.32389976	9621.90401

cg25130277	0.03721298	232222.921	2.07952258	2.5933E+10
cg18997075	0.03722439	10.0044127	1.14606982	87.3317411
cg13212159	0.03722515	996.489761	1.50486475	659854.544
cg00257542	0.03722825	1684792.2	2.33609661	1.2151E+12
cg15444648	0.03722963	6.26682935	1.11471623	35.2315226
cg16928994	0.03722978	8452.17938	1.70753885	41837605.2
cg19043574	0.03723362	4.38243514	1.09133992	17.5983096
cg24742092	0.03723861	48.4821614	1.25796107	1868.51567
cg21581821	0.03723874	5.72813383	1.10871583	29.5941631
cg04867634	0.03724514	54.4601295	1.26647561	2341.85774
cg16625170	0.03724594	1233201771	3.44520905	4.4142E+17
cg00456593	0.03724709	0.09180485	0.00970538	0.86839764
cg12516504	0.03725909	57163721.8	2.87002208	1.1386E+15
cg22827290	0.03726888	2.71E-08	2.06E-15	0.35788175
cg05054115	0.03727089	959.048153	1.4991219	613541.406
cg18825419	0.03727693	7.39495776	1.12516065	48.6023044
cg26672431	0.03727969	11293487.6	2.60370989	4.8985E+13
cg13975303	0.03728398	770.941916	1.47930738	401776.836
cg00251738	0.0372848	71452862.8	2.90137992	1.7597E+15
cg10523140	0.03728925	6.78657795	1.11935081	41.1467432
cg09805692	0.0372903	37.059105	1.23698873	1110.25851
cg07706540	0.03729488	2.36E-06	1.20E-11	0.46654493
cg01113435	0.0372977	45.9989603	1.25265136	1689.14066
cg12122509	0.03730119	5.156E+10	4.26667856	6.23E+20
cg04458206	0.03730186	256.685104	1.3858335	47543.4046
cg08845123	0.03730227	4.29583388	1.08951182	16.9380345
cg06106428	0.0373033	5.56741202	1.10624398	28.0192048
cg19794507	0.03731052	5.45878445	1.1048958	26.9693556
cg23910786	0.03732254	904520.427	2.23728818	3.6569E+11
cg01453732	0.03732345	4610913.02	2.46163775	8.6367E+12

cg21391660	0.03732368	3638.65952	1.61828545	8181401.54
cg17253307	0.03732737	173339033	3.04464974	9.8686E+15
cg02893231	0.03732904	5.50039794	1.10521626	27.3741698
cg01079738	0.03733636	190.917351	1.3606935	26787.3955
cg17929627	0.03734231	33367.4503	1.84135414	604656497
cg08574227	0.03737815	0.14756916	0.02435306	0.89420604
cg22184507	0.03739267	6.42064359	1.11464213	36.9846635
cg23817893	0.03739775	4.30652799	1.08891953	17.0317299
cg21105875	0.03740345	2.99334847	1.06602204	8.40520618
cg25096582	0.03740364	2852.45824	1.59030782	5116316.42
cg01158680	0.03740646	4.49141501	1.0915226	18.4813478
cg21844669	0.03741136	0.2300744	0.05766642	0.91793847
cg10881110	0.03742002	8.74098003	1.13456164	67.3429538
cg14522034	0.03742075	9.382681	1.13924241	77.2747765
cg05160963	0.03742396	499279681	3.20881541	7.7686E+16
cg07305215	0.03742551	257075.537	2.06484177	3.2006E+10
cg21906605	0.03742728	68.5061387	1.27888243	3669.68137
cg04164924	0.03743101	10.0672344	1.1437894	88.6082777
cg23085167	0.03743359	0.23889039	0.06202457	0.92009689
cg00310855	0.03744924	270.342174	1.38440015	52791.7389
cg01680448	0.0374518	3.0426E+11	4.643843	1.99E+22
cg02604524	0.03745824	2.63677213	1.05788864	6.57211638
cg12996903	0.03745994	4.64660103	1.09324214	19.7494227
cg10939691	0.03746498	8.927456	1.13540445	70.1947842
cg22222281	0.03747705	3.80871871	1.0805768	13.4246249
cg17300307	0.03748027	191131920	3.01831413	1.2103E+16
cg21583565	0.0374806	185.462098	1.35332983	25415.9694
cg09550809	0.03749022	7.76603887	1.12597399	53.5637238
cg18179964	0.03749099	4.77564381	1.09471875	20.8334549
cg04036714	0.03749724	312434578	3.10039171	3.1485E+16

cg07556218	0.03750936	821190.083	2.19681187	3.0697E+11
cg18378490	0.03751266	5447676.01	2.45003672	1.2113E+13
cg05247767	0.03751354	2623.18327	1.57580279	4366720.57
cg23249140	0.03751933	5.49415783	1.10337235	27.3577368
cg14589014	0.0375213	178128.166	2.00968248	1.5788E+10
cg22270364	0.03753126	4.00760999	1.08336606	14.8250332
cg05958352	0.03753148	5.34812804	1.10154658	25.9657413
cg04321497	0.03753474	231145.405	2.03846846	2.621E+10
cg20419291	0.03754088	0.13711774	0.02108239	0.89179991
cg06849960	0.0375415	5.62459027	1.10465924	28.63871
cg00006626	0.03754463	4.68268417	1.0930267	20.0612949
cg15628562	0.03754601	105.767997	1.30803687	8552.41127
cg26857670	0.03754793	4.08106388	1.08437615	15.3591375
cg00747184	0.03755001	4709.48939	1.62750339	13627799.7
cg22837251	0.03755246	0.10098939	0.0116381	0.87633374
cg18695750	0.03755807	195.700885	1.35481112	28268.7645
cg22364205	0.03757405	204.600393	1.35771306	30832.2295
cg11251349	0.03757449	72512822.3	2.82957606	1.8583E+15
cg11019437	0.03757478	5.2162E+10	4.12941746	6.59E+20
cg19334030	0.03757726	23.6223228	1.19923183	465.30964
cg02700626	0.03758323	3.62974233	1.07683889	12.2349124
cg10824915	0.03758551	26.5915071	1.2072563	585.715104
cg25065723	0.03759203	0.16806822	0.0312906	0.90272885
cg04709129	0.03759338	0.02301642	0.00065774	0.80542018
cg02197938	0.03759347	6.86751538	1.1168908	42.2268386
cg09869729	0.03759431	7.517239	1.12268913	50.3335077
cg05301609	0.03760638	7.33331443	1.12096174	47.97443
cg15445332	0.03760897	1136978014	3.30273645	3.9141E+17
cg05148093	0.03761797	393.665856	1.40792564	110071.727
cg08052882	0.03761967	402.059786	1.40955635	114682.943

cg15395193	0.03762832	2397327237	3.43986603	1.6708E+18
cg15283028	0.03763077	1.7352E+10	3.85115526	7.8187E+19
cg00262061	0.03763562	3150.54988	1.58485571	6263008.34
cg18261804	0.03763792	8.53E-05	1.24E-08	0.58535538
cg04446579	0.03763812	4.77881597	1.09351774	20.8840525
cg06963233	0.03764966	5.24553626	1.09925344	25.0312163
cg24247086	0.03765102	748.537171	1.45911574	384005.107
cg09527192	0.03766103	109.428273	1.30711509	9161.05019
cg26287080	0.03766234	0.20982361	0.04812673	0.9147919
cg08949408	0.03766807	3.56689019	1.07518685	11.8330183
cg07632934	0.03767053	0.22067472	0.05307712	0.91748248
cg04718145	0.03767612	5.39188409	1.10073991	26.4117016
cg01646147	0.03767712	5169.95995	1.62751928	16422838.2
cg03559686	0.03769029	5.62977792	1.10331737	28.7264575
cg10103631	0.03769167	6.7745E+14	6.97793027	6.58E+28
cg23858040	0.0376963	7.61857162	1.12240486	51.7127425
cg02180498	0.03769998	6508.08133	1.64736853	25710775.5
cg01179696	0.0377002	8.11867983	1.126425	58.5151804
cg09876101	0.03770398	3407226782	3.4811206	3.3349E+18
cg26466921	0.03770837	33.903201	1.22160069	940.918788
cg10953436	0.03771444	5.15793208	1.09762171	24.2380986
cg18147309	0.03771513	8.9321E+14	7.06039797	1.13E+29
cg00408964	0.03771825	1968583.65	2.27640312	1.7024E+12
cg01347596	0.03771833	47.3776785	1.24480985	1803.20265
cg12598803	0.03772517	7.35871687	1.119874	48.3542918
cg02407720	0.03772709	3.93625799	1.0808125	14.3356289
cg09526712	0.03772945	6.18535931	1.10885134	34.5029747
cg21634218	0.03774153	4.06927253	1.08274351	15.2935378
cg14975318	0.037742	30190.2935	1.79369248	508143862
cg26475097	0.0377445	1574751.86	2.24359912	1.1053E+12

cg09563216	0.03774965	4.34553614	1.08671568	17.3768398
cg02710004	0.03775199	0.1886414	0.03910819	0.9099266
cg05568384	0.03775406	236506.05	2.01402759	2.7773E+10
cg13405887	0.03775555	2.92737183	1.06265196	8.06426393
cg06807696	0.037756	3.60570401	1.07525274	12.0912051
cg01945183	0.03775931	8.26225207	1.12685483	60.5799498
cg07583490	0.0377619	7.27113144	1.11871204	47.2591252
cg14559139	0.03776214	444.180127	1.41156256	139771.337
cg22027947	0.03776955	8560114	2.46449566	2.9732E+13
cg14328457	0.0377753	3.33646424	1.07041973	10.3996529
cg16592713	0.03777663	53346887.8	2.7312422	1.042E+15
cg22375689	0.03778247	0.19226861	0.04057279	0.91113339
cg13716638	0.03778282	2712.27348	1.56235712	4708544.13
cg07095459	0.03778705	5405704431	3.54101386	8.2523E+18
cg19187185	0.03779162	23.2027228	1.19402384	450.884082
cg08445694	0.03779273	0.06764161	0.00532592	0.85907953
cg09551172	0.03779294	155792.179	1.96251495	1.2367E+10
cg24944820	0.03779663	7.46573749	1.11999891	49.7654377
cg16802592	0.03780047	4.45318484	1.08781525	18.2299846
cg23752923	0.03781174	53.3361359	1.25091748	2274.12554
cg05173382	0.03782171	5447720.18	2.39280866	1.2403E+13
cg04254487	0.03782711	4.82046611	1.09245915	21.2702631
cg17042098	0.03783103	6.5729294	1.11163176	38.8648492
cg06380459	0.03783234	7637515.92	2.4366993	2.3939E+13
cg23959605	0.03783374	5.76187209	1.10340829	30.0878382
cg22958605	0.03783406	741.592354	1.44967518	379367.204
cg01243251	0.03784577	184.070053	1.34009572	25283.1077
cg04617786	0.037846	27450.5694	1.77474234	424587695
cg16406225	0.03785179	4.02410385	1.08124172	14.9766805
cg09987240	0.0378531	8.0170063	1.12385565	57.1891862

cg03690837	0.03785935	36.1444246	1.22278562	1068.39614
cg20839219	0.03786239	624145.876	2.11262096	1.844E+11
cg09516144	0.03786305	1054.29421	1.47715094	752486.587
cg03979241	0.03786514	0.19942701	0.04353164	0.91361429
cg25673274	0.03786802	5.3081E+11	4.53784382	6.21E+22
cg05939324	0.0378747	0.21749121	0.0515204	0.91813007
cg16511708	0.03788035	35950916	2.64742972	4.882E+14
cg07697561	0.03788796	25480.5491	1.76364266	368134883
cg11639692	0.03788857	242470.212	2.00038064	2.939E+10
cg06602723	0.03789056	4.86989766	1.09254594	21.7070079
cg17895873	0.03789775	3623603305	3.42074123	3.8385E+18
cg04340651	0.03790213	5.31492667	1.09779639	25.7319534
cg17261676	0.03790929	6.44360064	1.10959485	37.4190536
cg22786741	0.03791685	1807751.15	2.2337381	1.463E+12
cg24292487	0.03791738	28.6940234	1.20590723	682.761459
cg19847038	0.0379217	8.12473182	1.12390291	58.7339589
cg05018361	0.03792438	3.421E+10	3.86563632	3.03E+20
cg08192350	0.03793065	4.88689045	1.09241714	21.8613362
cg11613545	0.03794498	4.28E-05	3.20E-09	0.57134645
cg05038216	0.037945	107.797153	1.2974796	8955.99913
cg00174845	0.03794787	0.223348	0.05422251	0.91999301
cg25022560	0.03794951	229583309	2.91771857	1.8065E+16
cg26836743	0.03795259	84.5519119	1.27985047	5585.82894
cg25289028	0.03795279	5.63469855	1.10091056	28.8396068
cg24361586	0.03795352	0.07953439	0.00728183	0.86869933
cg01068014	0.03796466	234.254338	1.35402861	40527.2787
cg24514884	0.03797221	12.9541809	1.15278746	145.569595
cg08044454	0.03797493	0.16337012	0.02951285	0.90434507
cg20700235	0.03797898	346.251373	1.38317016	86677.7038
cg08133931	0.03798089	7.75080758	1.12028679	53.6246779

cg16765160	0.03798429	116114917	2.80026087	4.8148E+15
cg24042517	0.03799263	4.75387574	1.09022114	20.7291288
cg17124509	0.0379941	299.383527	1.37147456	65353.3785
cg26340737	0.03799958	62.7105345	1.25755703	3127.18314
cg06288351	0.03800342	7.57500905	1.11861137	51.296423
cg27337894	0.0380126	17.9312847	1.17310955	274.084351
cg19630883	0.03802456	67580.5786	1.848668	2470500173
cg02721176	0.03802883	0.28276286	0.08573209	0.93261268
cg11799593	0.03803323	3.97132355	1.07911333	14.615157
cg04656882	0.03803525	625.693471	1.42678569	274387.614
cg13392052	0.03803526	1.9044E+10	3.69351653	9.8187E+19
cg13915028	0.03803687	1383.83159	1.49060436	1284707.01
cg10477603	0.03804153	40356.8841	1.79516357	907258882
cg02136628	0.03804313	54700576.3	2.67200112	1.1198E+15
cg02673986	0.0380435	10.4071038	1.13792927	95.1797377
cg02325300	0.03805522	1984.71902	1.51952959	2592321.74
cg07237214	0.03806161	14.5318468	1.15880281	182.235121
cg12039184	0.03806228	464155532	3.00087938	7.1792E+16
cg17266233	0.03806561	7.43346888	1.11675993	49.4792642
cg24081819	0.03807002	36.309425	1.2185592	1081.91243
cg27259408	0.03807395	12.8472456	1.15078854	143.424889
cg19052355	0.03807931	3.75575743	1.07547158	13.1158407
cg00930615	0.03808298	154.521305	1.31922558	18099.1288
cg22719334	0.03808417	1335780148	3.17357295	5.6224E+17
cg05934259	0.03808611	7651315.5	2.38925747	2.4502E+13
cg25341925	0.03808625	8.77030534	1.12672579	68.2670591
cg15447231	0.0380892	5.43442673	1.09745249	26.9104988
cg10172318	0.03809972	49.6750247	1.23905333	1991.52693
cg16107907	0.03810491	39.9374841	1.22418943	1302.90508
cg11121935	0.03810552	124.889311	1.3031817	11968.6611

cg19077271	0.03810825	36.403207	1.21791076	1088.0875
cg24216220	0.0381103	16.5973982	1.16653226	236.147458
cg16643473	0.03811093	11.9480074	1.14568839	124.601839
cg17207836	0.03811474	24900.2862	1.74162685	356002924
cg01591369	0.03813074	8668.41748	1.64258864	45745757.6
cg23341612	0.03813443	5.6266102	1.09912846	28.8034961
cg02131049	0.03813752	567888221	3.0119087	1.0707E+17
cg03785696	0.0381483	7.55864301	1.11687049	51.154619
cg08321129	0.03814858	8.77925448	1.12604097	68.4480505
cg14690220	0.03815139	8.2327E+10	3.94745736	1.72E+21
cg05663031	0.03816807	5.98329742	1.10250569	32.4713498
cg25057892	0.03817795	1559034.84	2.17525599	1.1174E+12
cg22597665	0.03817977	531.719518	1.40774908	200835.255
cg20171999	0.0381862	8.52136344	1.12376396	64.6164473
cg25983317	0.03818633	113813.377	1.88518856	6871187949
cg19540689	0.03821647	4.24956397	1.08174812	16.694084
cg11005899	0.03823674	665931.502	2.06870948	2.1437E+11
cg02024449	0.03823686	3957956334	3.31353955	4.7277E+18
cg23601416	0.03824211	19.9129424	1.17596541	337.191276
cg20451727	0.03824484	16297.0162	1.6911398	157049545
cg16751623	0.03824793	78.2212968	1.26630159	4831.84365
cg26142075	0.03824885	6899429.17	2.3460638	2.029E+13
cg22622425	0.0382499	1538.9441	1.4879222	1591715.57
cg02234820	0.03825427	10.2909069	1.13448872	93.3484511
cg06712722	0.03825488	163.838435	1.31780487	20369.505
cg02620694	0.03826193	6.27585001	1.10444735	35.6615398
cg18374708	0.03826461	1409562.16	2.15037801	9.2396E+11
cg23331156	0.0382691	23.7017876	1.18662025	473.424191
cg13259290	0.03826997	10.5520054	1.13582262	98.0301117
cg10742957	0.03827084	9.67970615	1.13052699	82.8787917

cg03314195	0.03827164	6.62043806	1.10754555	39.5741738
cg10899006	0.03827267	4824772.05	2.29693548	1.0135E+13
cg03591990	0.03827485	1597.31099	1.48958167	1712831.5
cg27431396	0.03829254	12056.1903	1.6601049	87555747.6
cg12878710	0.03829526	985.33433	1.45020834	669478.803
cg15004702	0.03829787	1185.63425	1.46462033	959790.425
cg05801246	0.03830184	6.17891876	1.10312536	34.6098807
cg20270172	0.03830231	164343858	2.77175692	9.7443E+15
cg20303850	0.03830304	7.27E-11	1.86E-20	0.28421712
cg11849086	0.03830434	1551748172	3.12759951	7.6989E+17
cg00415665	0.03830441	2297.2037	1.51741335	3477724.01
cg26942169	0.03830627	5.83179147	1.09965126	30.9277979
cg19522294	0.03830963	425606.94	2.00979226	9.0129E+10
cg14077270	0.03831116	82567.9625	1.83975096	3705647432
cg15929437	0.03831289	104088.595	1.86265793	5816653424
cg06632759	0.03832047	0.28410785	0.08637145	0.93453642
cg20422722	0.03832615	22.4519249	1.18212328	426.426699
cg25604984	0.03832871	9129343.15	2.36704281	3.5211E+13
cg09671109	0.03833138	5990374125	3.3537161	1.07E+19
cg15917704	0.03833243	112.319305	1.28883955	9788.36061
cg17777874	0.03833441	78135.8735	1.83194388	3332642872
cg18927077	0.03833684	7.03773835	1.11051867	44.6005657
cg24269840	0.0383387	6.06126332	1.10162499	33.3497457
cg17782954	0.03833968	4.43802292	1.08332653	18.181081
cg16523137	0.03834098	125.705999	1.29640056	12189.1325
cg16904330	0.0383419	5.13046962	1.09177601	24.1090831
cg20001810	0.03834383	5.33282463	1.09402889	25.9947602
cg07540542	0.03834391	487.157368	1.39410109	170233.208
cg15133313	0.03834643	1736.70598	1.49242617	2020969.42
cg26149131	0.03835836	0.01002037	0.00012852	0.7812927

cg19621160	0.03835912	45.0286618	1.22644679	1653.21513
cg04743859	0.03836285	7.23546589	1.111893	47.0836372
cg16416718	0.03836382	46187.226	1.77816623	1199696521
cg01755539	0.03837202	1.2389E+10	3.47123009	4.42E+19
cg08798701	0.03839419	0.00042555	2.74E-07	0.66045837
cg19720565	0.03839436	9.26132655	1.12631483	76.1529255
cg10944735	0.03840156	8.91893306	1.12396306	70.7740051
cg10297105	0.03841902	10.5990261	1.1341422	99.0522655
cg04834794	0.03842832	4443.83172	1.56434957	12623547
cg22711917	0.03842988	4512211.39	2.26183505	9.0016E+12
cg20585530	0.03844306	4.08773601	1.07778625	15.5036175
cg02084834	0.03844492	16267943	2.41878906	1.0941E+14
cg25723986	0.03845462	24.2028798	1.18453402	494.523062
cg24930634	0.03845585	234776325	2.78496344	1.9792E+16
cg07575519	0.03846053	11.1891046	1.13686634	110.123817
cg05132828	0.03846071	11.8033973	1.140096	122.2004
cg04805577	0.03848931	6.5997586	1.10513703	39.4130432
cg16696007	0.03849119	3.42E-06	2.28E-11	0.51342657
cg24404909	0.03849413	29.0501191	1.19530491	706.020205
cg27299660	0.03850238	0.00030092	1.39E-07	0.65112399
cg04319732	0.0385061	8.48978286	1.11978328	64.3663949
cg25518276	0.03850771	8.05774035	1.11667564	58.1432758
cg01860010	0.03850789	505895.442	2.00293373	1.2778E+11
cg04781638	0.03850978	9.34925605	1.1254645	77.6644564
cg14881284	0.03851606	2009806.38	2.15327206	1.8759E+12
cg09624525	0.03852463	7558.56746	1.6025047	35651653.3
cg05946827	0.0385308	342.172551	1.36064072	86049.2072
cg13611783	0.0385316	1015.11363	1.44097164	715111.705
cg18829186	0.03854371	1624.01073	1.4765048	1786252.81
cg23737062	0.03855226	7.02655564	1.1081479	44.5540565

cg12747487	0.038553	5707052.39	2.26899845	1.4355E+13
cg23678322	0.03855395	93.4972479	1.26994584	6883.54976
cg13235098	0.0385547	0.0057876	4.39E-05	0.76239146
cg00037314	0.03856301	0.0365298	0.00158827	0.84017624
cg12318400	0.03856385	1.3926E+10	3.41742001	5.6745E+19
cg09934977	0.03856607	25.8527674	1.18659152	563.265094
cg06289844	0.03856741	5.1354385	1.08986675	24.198122
cg00173435	0.03856763	47106.1182	1.7610808	1260013946
cg08905031	0.03857307	14269763	2.3773602	8.5652E+13
cg20313969	0.03857429	5.48304331	1.09356513	27.4915166
cg10115182	0.03857533	384903.133	1.96585067	7.5362E+10
cg22432024	0.03859062	0.06048035	0.00423811	0.86308959
cg06524337	0.03859156	169.763579	1.30923577	22012.592
cg14831166	0.03859187	21.8676719	1.17573163	406.721277
cg09242541	0.03859595	8.28714728	1.11731707	61.4658202
cg27332337	0.03859949	8.74837916	1.12045414	68.3063547
cg00328058	0.03860098	0.04287995	0.00216881	0.84778595
cg21420622	0.0386019	700934.404	2.02523307	2.4259E+11
cg12855522	0.03860194	4.19393695	1.07805884	16.3155354
cg11209279	0.0386095	169453436	2.69851418	1.0641E+16
cg10840064	0.03861451	1581.8005	1.47071047	1701281.71
cg03008269	0.03861727	4600.51975	1.55509789	13609935.5
cg17814457	0.03862268	2150284.23	2.14464864	2.1559E+12
cg00307214	0.03862609	6.60492257	1.10379143	39.5228671
cg14673936	0.03863049	9.02879693	1.12193632	72.6593595
cg14032725	0.03863253	2075.71208	1.49078825	2890135.9
cg24775616	0.03863507	0.00017123	4.61E-08	0.6355424
cg15377585	0.03863595	5.03846271	1.08818628	23.3288242
cg21773314	0.03864099	4121.61319	1.54468701	10997499.9
cg04309480	0.03864136	0.20556654	0.04589792	0.92068671

cg09693228	0.0386449	13796581.7	2.35952553	8.0671E+13
cg08282512	0.03865247	0.03914618	0.00181474	0.8444325
cg04082020	0.03865356	371.142172	1.36165725	101160.928
cg21410293	0.03865858	14.0219315	1.14764505	171.320012
cg10409799	0.03866847	802394.922	2.03066082	3.1706E+11
cg16391783	0.03867459	28.6030817	1.19080405	687.045268
cg14360865	0.03868201	6.92951208	1.10598143	43.4167668
cg21220708	0.03869687	309.705067	1.34722983	71195.8914
cg02002586	0.03869875	10.1692478	1.1280656	91.6733932
cg05797295	0.03869925	95181874.5	2.59723857	3.4882E+15
cg07571928	0.038703	10.1036988	1.1276327	90.5301243
cg04450855	0.03871283	14.6599935	1.14949552	186.964982
cg10951691	0.03871503	10.8539723	1.13167732	104.100976
cg22005150	0.03871535	74.1382763	1.25028243	4396.19393
cg09173265	0.03873137	1487104.67	2.08788994	1.0592E+12
cg21344215	0.0387327	10382095	2.30874375	4.6687E+13
cg07803811	0.03873371	1.174E+17	7.65525486	1.80E+33
cg12945194	0.03873496	7.37599145	1.10900921	49.0575275
cg11050622	0.03873521	191.636838	1.31275138	27975.3489
cg25206536	0.03873548	6.26261891	1.09964745	35.6663363
cg03775802	0.03874095	0.19264543	0.04041395	0.91830331
cg07286413	0.0387434	91028316.8	2.58103468	3.2104E+15
cg12619165	0.03874347	0.27850306	0.08286732	0.93600172
cg21097788	0.03875254	0.14174976	0.02222827	0.90393869
cg05708497	0.03876155	10.5470426	1.12939177	98.4955879
cg02670343	0.03876327	20.1360493	1.16772136	347.223658
cg23173307	0.03876449	7.21115188	1.10739761	46.9575797
cg25056994	0.03877547	5300375.24	2.2225646	1.264E+13
cg02007844	0.03877895	5.44466924	1.09131605	27.1639212
cg06957181	0.03878209	0.13579453	0.02043922	0.90219442

cg23169269	0.03878899	0.1173716	0.01538363	0.89550338
cg07109490	0.03878932	0.22890655	0.05653319	0.9268574
cg19784449	0.03879667	535.759521	1.38192558	207708.917
cg13421439	0.0387982	4.01710501	1.07419808	15.0224925
cg21667061	0.03879934	7.06006226	1.10582095	45.0746381
cg20502259	0.03881373	143.210166	1.29064997	15890.5605
cg17230002	0.03881516	0.01995934	0.00048713	0.817798
cg13554714	0.03881571	136.827679	1.2875685	14540.4411
cg06980169	0.03881903	6.08200246	1.09717696	33.7144828
cg06432479	0.03882079	36.8562924	1.20353503	1128.66369
cg20929143	0.03882158	350454.906	1.92646185	6.3753E+10
cg02924834	0.03882196	9.45171185	1.12227251	79.6017513
cg03339668	0.03882364	5.79067201	1.0943708	30.6403299
cg02019444	0.03882605	0.15177188	0.02537565	0.90774846
cg13444964	0.0388321	1062911.2	2.0379783	5.5436E+11
cg19257562	0.03883567	24.4003824	1.17803619	505.399296
cg22773661	0.03884585	2.53936367	1.04890956	6.14768718
cg17762617	0.03884633	378.474586	1.35547718	105677.185
cg24727290	0.03884897	12.2286407	1.13684332	131.53937
cg27301328	0.03885293	985.237051	1.42326395	682018.288
cg04410756	0.03886038	8.9030676	1.1183743	70.8748521
cg00971933	0.03887913	3.3355E+11	3.87775507	2.87E+22
cg01443318	0.03888411	8.35025666	1.1144391	62.5667085
cg17800649	0.03888621	227642.132	1.876954	2.7609E+10
cg20726001	0.03889326	19.8574461	1.16467908	338.563792
cg13010199	0.03889965	7026.81482	1.57072949	31435155.9
cg12178578	0.03890304	6.94199036	1.10378269	43.6600705
cg01154849	0.03890938	1082908.01	2.02930857	5.7788E+11
cg14264773	0.03891048	644.85897	1.39018948	299126.915
cg24776411	0.03892274	4.945E+10	3.49921899	6.99E+20

cg22753661	0.03892386	263.45838	1.32775888	52276.2976
cg14771938	0.03892594	681.022328	1.39337516	332854.658
cg25512791	0.03892849	19.4844202	1.16296168	326.444661
cg02372786	0.03893433	5.73586	1.09280909	30.1059812
cg03462943	0.03893557	14.6210163	1.14600191	186.539059
cg00012386	0.03893821	207.881696	1.31136686	32954.0121
cg01444765	0.03894731	4.158E+10	3.45839711	5.00E+20
cg01090821	0.03894917	18.9053916	1.16083667	307.8933
cg22999786	0.03895066	14.87199	1.14676637	192.869351
cg00871371	0.03895077	4.70504121	1.0817307	20.4648096
cg26434278	0.0389585	144005.055	1.82595934	1.1357E+10
cg04849129	0.03896558	1.3895E+10	3.26450575	5.9139E+19
cg14330641	0.03896877	4.39647762	1.07787527	17.9325159
cg02882968	0.03897146	0.12507455	0.01737992	0.90009853
cg13800542	0.03897287	310920.913	1.896937	5.0962E+10
cg22669260	0.0389779	930.381592	1.41321819	612509.742
cg20942223	0.03897906	6.23025298	1.09697353	35.3846752
cg19047292	0.03898683	3.75767032	1.06921482	13.2060331
cg13660163	0.03899047	29.719455	1.18698018	744.111842
cg17480669	0.0389941	89.3103175	1.2547548	6356.88567
cg04525496	0.03899877	7.45127949	1.10673852	50.1668326
cg26693601	0.03900788	7.73829464	1.10875248	54.0077295
cg11006995	0.03900996	9.90994588	1.12265037	87.477838
cg09789590	0.03901123	6.25494929	1.09687909	35.6688272
cg21359793	0.03901529	6.37807418	1.09791799	37.051793
cg19826026	0.03901749	4.04800221	1.07302212	15.2711875
cg16662919	0.03902242	1.68E+11	3.67752503	7.67E+21
cg03051392	0.03902349	16.5787272	1.15195998	238.597001
cg12339905	0.03902721	6.045843	1.09484684	33.3856902
cg06232807	0.03902733	0.23799066	0.06088569	0.93026045

cg06969845	0.03902781	0.13602076	0.02045684	0.90442334
cg01005506	0.0390359	22.2216402	1.16886808	422.4611
cg11782550	0.03903885	4.32536594	1.07644918	17.3800964
cg25893515	0.0390418	26717512.7	2.36308898	3.0207E+14
cg03882777	0.03904328	5.39131178	1.08840454	26.705367
cg23719318	0.03904795	3.16390467	1.05959626	9.44727075
cg20497205	0.03905134	776.39839	1.39702237	431485.189
cg24333845	0.03905371	7.62137845	1.107401	52.4520111
cg22168205	0.0390577	6.76661355	1.10076301	41.5957465
cg08832136	0.03905977	3271.24868	1.50121279	7128281.85
cg26463170	0.03906637	86.714142	1.25092738	6011.01432
cg14572436	0.03906863	45.6414209	1.21124022	1719.83993
cg12566415	0.03907804	115448.236	1.79344691	7431664221
cg16961545	0.0390783	76.5020055	1.24277665	4709.25878
cg13919148	0.03907846	3478562.26	2.12712138	5.6886E+12
cg09667013	0.0390805	7.70124257	1.10768725	53.5432155
cg15140562	0.03908412	264.64675	1.32231313	52966.2003
cg21008401	0.03908564	35725.3513	1.69041319	755022936
cg05092885	0.03909403	21995.2681	1.64918025	293352907
cg03536654	0.03909974	451.846271	1.35759032	150387.823
cg20232291	0.03910429	9.45426222	1.11883729	79.8892519
cg26523099	0.03910552	6.11568129	1.09472712	34.1651878
cg01244346	0.039114	104.561779	1.26137343	8667.66757
cg25418406	0.03911663	984349.491	1.99163605	4.8651E+11
cg03321133	0.03911786	7.97805112	1.10923136	57.3814461
cg02696790	0.03912364	4804.54373	1.52645019	15122432.8
cg01466164	0.03912527	0.19031324	0.03934419	0.92057124
cg15971518	0.03913009	0.18526163	0.03733183	0.91937293
cg23157190	0.03913212	33.6545033	1.19158086	950.523484
cg07196758	0.03914363	4415586.2	2.14231835	9.1011E+12

cg01566283	0.03914489	11.5446957	1.12952102	117.996918
cg16985259	0.0391533	8.569E+13	4.93322062	1.49E+27
cg18251245	0.03915797	1833347.47	2.04851855	1.6408E+12
cg21745091	0.03916207	413733588	2.6809909	6.3848E+16
cg12793610	0.03917217	8.66410583	1.11317596	67.4347383
cg26649219	0.03917281	1.12E-08	3.14E-16	0.40300525
cg08970330	0.03917541	594291276	2.72612604	1.2955E+17
cg11579905	0.03917602	15.878945	1.14711225	219.804901
cg10937776	0.03917911	1155.34272	1.41899121	940680.096
cg04856117	0.03918268	64.1674304	1.22928461	3349.47586
cg19511862	0.03919137	2.2413E+13	4.58874846	1.09E+26
cg00851532	0.03919619	4.8479063	1.08134007	21.7343241
cg00102996	0.03919623	124285.008	1.78803544	8638958061
cg24578679	0.03919832	5.09366242	1.08397409	23.93544
cg06452665	0.03920736	3.80440855	1.06835597	13.5474737
cg20945738	0.03921069	9.05461705	1.1151572	73.5197599
cg23375552	0.03921268	9.2792152	1.11648576	77.1204058
cg09455126	0.03921323	0.1819822	0.03602922	0.91918508
cg26551026	0.03921479	5.03637196	1.08322734	23.4161764
cg23481605	0.03921848	10.9691289	1.12568733	106.887397
cg13819787	0.03922089	5.04804955	1.08329962	23.5233207
cg06152533	0.03922164	8.98767898	1.11461809	72.4717949
cg07774251	0.03923456	6.8280758	1.09945152	42.4053431
cg09970396	0.03923917	264958.74	1.85154937	3.7916E+10
cg20499859	0.03923929	29556853.4	2.33634403	3.7392E+14
cg08209184	0.03924454	0.00312415	1.30E-05	0.75244655
cg20799547	0.03924924	3.7283E+11	3.7177505	3.74E+22
cg19112186	0.03926033	6.57892526	1.09717851	39.4486924
cg07489553	0.0392612	3.714E+12	4.156746	3.32E+24
cg16544736	0.03927572	22252.1556	1.6356668	302725732

cg12242338	0.03927711	0.25946212	0.07193582	0.93584248
cg26823630	0.03928192	47853.5703	1.69785496	1348739584
cg23188547	0.03928363	9001.67467	1.56395137	51811167.7
cg01119512	0.03928737	358.284429	1.33477814	96171.5866
cg25117523	0.03928926	19.221849	1.15616766	319.572577
cg10705060	0.03929401	33.215647	1.18753654	929.04864
cg20373747	0.03929507	19.7207357	1.15752589	335.981612
cg13560136	0.03930036	810612065	2.73432791	2.4031E+17
cg24036523	0.03930714	7.07299908	1.10060916	45.4542065
cg01758575	0.03931367	66.1109649	1.22783866	3559.63679
cg05585556	0.0393284	36.3821887	1.1921414	1110.32438
cg21741010	0.03932871	8.10897982	1.10776403	59.3588094
cg02585745	0.03933616	22.5763624	1.16450977	437.688162
cg25692259	0.0393443	4518577.88	2.11309623	9.6624E+12
cg26363065	0.03935545	14770.7264	1.59712639	136604315
cg16011258	0.03935851	548239956	2.66725935	1.1269E+17
cg26975459	0.03936253	461.526784	1.34846649	157962.377
cg19476445	0.03938108	30.8267345	1.181489	804.31351
cg03277051	0.03938283	338.856234	1.32755527	86492.4797
cg00978822	0.03938578	7.93424462	1.10595253	56.9212837
cg24616138	0.03939363	289.711249	1.31708646	63725.9667
cg02383154	0.03939705	8.53466846	1.10975268	65.636756
cg03711682	0.03939808	864.259908	1.388716	537867.49
cg09278980	0.03940142	3.80535965	1.06702998	13.571092
cg20038219	0.03940416	6.87159833	1.09805927	43.0021082
cg07708721	0.03940544	8.00595837	1.10621797	57.9409948
cg18427856	0.03941207	0.21943425	0.05182661	0.92908625
cg10598356	0.03941642	139635075	2.48214388	7.8553E+15
cg24334259	0.03942132	15.1305589	1.14068394	200.698725
cg16403932	0.03942177	0.2488941	0.06626614	0.93484052

cg12754854	0.03943204	13.6813601	1.13497934	164.918962
cg09935495	0.03943331	38.3759097	1.19304749	1234.41058
cg08359167	0.03943677	196.102514	1.29092905	29789.5505
cg09760963	0.03943717	4.6059596	1.07668294	19.70391
cg23042775	0.03943866	10.0447646	1.11804992	90.2439996
cg07199534	0.03944256	10.4436093	1.12010811	97.3736142
cg17601287	0.03944847	11.9029652	1.1271344	125.699812
cg01022780	0.03946858	6.51937238	1.09462006	38.8282818
cg01476047	0.03947487	43.2514466	1.19906598	1560.12068
cg17868052	0.03949298	26.3579579	1.17045092	593.567775
cg11390445	0.03950226	1.2273E+10	3.05406414	4.9318E+19
cg01640991	0.0395061	0.03661345	0.0015714	0.85308961
cg11530564	0.03950749	19.6382976	1.15375727	334.266784
cg14597214	0.03951466	4.10496004	1.07013646	15.7463067
cg10276465	0.03951551	23.1861554	1.16285643	462.307976
cg02533281	0.03951618	38.3446124	1.19126144	1234.24569
cg15095906	0.03951991	14.5111338	1.1369278	185.212294
cg10509187	0.03952062	5.47537133	1.08498317	27.6314803
cg11483915	0.03952157	19.2381959	1.15238553	321.16698
cg04064380	0.03952284	13.2937818	1.13211727	156.100997
cg09948350	0.03953149	0.05069225	0.00296443	0.86684545
cg07068756	0.03953204	2.77124866	1.05005381	7.31373866
cg01504836	0.03953351	20774032	2.24170992	1.9251E+14
cg12601909	0.03953513	6.01394807	1.08974058	33.1891571
cg04880506	0.03954436	11204062.1	2.17452157	5.7728E+13
cg13643509	0.03954784	110.16615	1.25225452	9691.78414
cg10327769	0.03954969	9.41616162	1.11322237	79.6463513
cg08390699	0.03955265	88.4997101	1.23907462	6321.00648
cg22171758	0.03955362	37.3253013	1.1889445	1171.77725
cg14597908	0.03955527	27.9563499	1.17259615	666.518903

cg09378756	0.03956468	0.09620779	0.01035098	0.8942086
cg25425705	0.03956576	32.2028125	1.18034251	878.576452
cg09339907	0.03957114	3413.24141	1.47445481	7901372.64
cg22416074	0.03957297	2759.92057	1.45947696	5219103.66
cg23562261	0.03957475	4.54147407	1.0748685	19.1883814
cg11120874	0.03957674	4.76348217	1.0773027	21.062569
cg25538340	0.03957942	41.8150416	1.19486386	1463.3447
cg22242926	0.03958176	41267740.6	2.30721835	7.3813E+14
cg25877553	0.03958445	5.33216067	1.0830464	26.2518185
cg20196910	0.03958944	21717.8135	1.60918309	293107372
cg02611085	0.03959169	9.14348815	1.11116064	75.2396839
cg22665276	0.03959345	33.4190113	1.18188015	944.960716
cg10917941	0.0395989	72.068368	1.22579562	4237.12532
cg03246824	0.03960054	2.8562E+10	3.14450917	2.59E+20
cg18048949	0.0396074	4969.08037	1.49889119	16473350.3
cg03379706	0.03961015	12.5204197	1.12766703	139.013472
cg13703021	0.03961799	0.02466066	0.00072509	0.83871908
cg07895169	0.03962315	5735186.77	2.09352623	1.5711E+13
cg19861329	0.03962442	4.4071E+10	3.20104732	6.07E+20
cg10376408	0.03962781	9.51821761	1.11285176	81.4092856
cg18832152	0.03962949	13.7656493	1.13248472	167.325084
cg20646950	0.03963418	47.0277157	1.20035633	1842.45794
cg06796435	0.03963696	6.99913851	1.09664056	44.6709174
cg06887580	0.03963715	13.7400415	1.13227515	166.733978
cg14105409	0.0396424	155.414504	1.27011502	19016.9141
cg05566582	0.03964432	0.10901477	0.01319989	0.9003274
cg27410595	0.03966108	0.14641145	0.02347545	0.91313743
cg04985097	0.03966113	356632.879	1.8305817	6.9479E+10
cg10682057	0.03966915	13312.7787	1.56637481	113146658
cg03137131	0.03966971	5.58133459	1.08464017	28.7203966

cg04505439	0.0396708	2321.38179	1.44219579	3736533.86
cg18909638	0.03968615	0.00090691	1.14E-06	0.7185818
cg16127617	0.03968619	12.8214781	1.12788699	145.750684
cg04948906	0.03969174	0.15318603	0.02563615	0.91534646
cg09490277	0.03969897	7.12031294	1.09689006	46.2205451
cg03572619	0.0397088	714.875532	1.36249227	375082.514
cg02492799	0.03970931	22.3932117	1.15754995	433.204575
cg15021531	0.03971075	25.2930824	1.16417671	549.5214
cg24296484	0.0397417	1988.7235	1.42798828	2769645.4
cg13164831	0.03975893	0.06263831	0.004467	0.8783421
cg04510564	0.03976078	3.94350016	1.06634077	14.5836997
cg01892327	0.03976409	1223520.46	1.9270494	7.7684E+11
cg06081199	0.03976602	5.23527064	1.0805349	25.3652693
cg20199347	0.03978484	8.12706671	1.10279038	59.8928086
cg25291004	0.03978914	5.4629933	1.08248524	27.5701643
cg10208594	0.03978917	53925764.6	2.29565092	1.2667E+15
cg23121993	0.03979198	2.62459625	1.0460571	6.58520984
cg19624799	0.03979402	748.728433	1.36176115	411668.571
cg27079322	0.03980138	7.01696024	1.09508276	44.9625661
cg03612039	0.03981731	5.082705	1.07860797	23.9511396
cg07835283	0.03982143	13.9881211	1.13058734	173.067153
cg15153018	0.0398225	30250.256	1.6159722	566270872
cg03553278	0.0398415	3.8689355	1.06482885	14.0573406
cg10512875	0.03984362	3.17684745	1.05511747	9.56515264
cg10788735	0.03984464	4.38883298	1.07105634	17.9839792
cg11609154	0.03985209	829.956139	1.36575505	504356.32
cg26337868	0.03985973	8.94336843	1.10685687	72.2621338
cg05172068	0.03986231	2000720.11	1.95844639	2.0439E+12
cg25492727	0.03986831	2.14E-08	1.04E-15	0.4414781
cg19949241	0.03986986	0.15869858	0.02742532	0.9183207

cg15928480	0.03988656	33.1415142	1.17559116	934.304379
cg02083999	0.03989138	4198.50045	1.47007332	11990834.7
cg01780109	0.03990533	2289.44872	1.42872964	3668696.53
cg03642503	0.03990879	11.0271057	1.11701948	108.858495
cg01965047	0.0399111	5.34144205	1.0802873	26.4105697
cg26894854	0.03991376	14.6899908	1.13181511	190.663501
cg24425171	0.03992148	5.24243879	1.07926702	25.464657
cg12811072	0.03992515	4.15438554	1.06774245	16.1639347
cg24720336	0.03992978	45.7860326	1.19233305	1758.20068
cg17838626	0.03993048	29.2574745	1.1680069	732.872226
cg25468863	0.03993445	1206.15115	1.38574273	1049834.56
cg15616946	0.03994403	1.73E-07	6.13E-14	0.4891125
cg11337261	0.03995108	1.8497E+10	2.9597882	1.16E+20
cg13628960	0.03995184	1.3636E+12	3.60527247	5.16E+23
cg25247520	0.03995664	5.86339565	1.08452003	31.700114
cg03660010	0.03996307	4.07695682	1.06654531	15.5845013
cg22661556	0.0399659	6736.25365	1.49779422	30295959.6
cg25019564	0.03997381	9.1366E+10	3.1761749	2.63E+21
cg09047573	0.03997761	7.81199999	1.09866202	55.5469677
cg02272993	0.03999665	6.7193902	1.09092153	41.3872158
cg22776856	0.04000513	10.4470849	1.11303173	98.0579253
cg19698570	0.04000861	1.2019E+13	3.95143135	3.66E+25
cg00994629	0.04000942	4.25627307	1.06830764	16.9575316
cg07318155	0.04001213	55698.0928	1.64605265	1884676976
cg09551160	0.04001293	9077.98087	1.51529442	54385296.7
cg00165360	0.04001531	1877.9901	1.41011149	2501112.03
cg07285708	0.04001917	8.81540129	1.10427629	70.3730587
cg24413235	0.04001939	412.403687	1.3157881	129258.504
cg03594790	0.04003069	6.01051194	1.08506184	33.294189
cg16370061	0.04003281	38912.1799	1.61765057	936022757

cg13049471	0.04003595	3.74460802	1.06190544	13.2046495
cg24937675	0.04003892	7.16349525	1.09367888	46.9202295
cg27246282	0.04003906	4.4290435	1.07002221	18.3327281
cg00419564	0.04004512	4465.09172	1.46514421	13607564.3
cg27144788	0.04004653	15255.7545	1.54918653	150232422
cg17695682	0.04005167	0.06382952	0.00461653	0.88252543
cg07826859	0.04005185	4.4500519	1.07015447	18.5047697
cg24917627	0.04005363	4880469557	2.75380607	8.6495E+18
cg08627125	0.04005644	5.25964973	1.07826982	25.6558374
cg09807148	0.04005682	23.7594409	1.15465671	488.899449
cg01668452	0.04006029	102.623237	1.23384998	8535.50195
cg06656027	0.04006829	28.700013	1.16438599	707.403519
cg07320850	0.04006849	11.1470872	1.11551356	111.390446
cg03516318	0.04007193	17.5493685	1.13864986	270.478526
cg19535896	0.04007377	4.19493557	1.06712809	16.4905082
cg19761115	0.04007761	4.69262051	1.07253234	20.5314902
cg07303577	0.04008173	0.22347975	0.05344886	0.93441091
cg25611569	0.04008373	0.11660238	0.01498508	0.90731018
cg15918224	0.04008401	44496967.5	2.21912149	8.9224E+14
cg01451391	0.04009055	3.38321895	1.05667664	10.8322358
cg07932328	0.04009327	147122.246	1.71266131	1.2638E+10
cg13264662	0.04009964	31864445.7	2.18300517	4.6511E+14
cg11786558	0.04009979	2636.34181	1.42752444	4868776.98
cg25981124	0.04010689	1319.17903	1.38321544	1258107.21
cg02593579	0.04010801	838.218014	1.35513155	518480.616
cg10168009	0.04011038	4.98269862	1.0751788	23.0913087
cg17702076	0.04011474	1424973369	2.58805716	7.8458E+17
cg09741298	0.04011724	103.93104	1.23299495	8760.50707
cg14796261	0.04011941	22.8737198	1.15158511	454.336422
cg09690326	0.0401308	51.1432487	1.19387948	2190.86763

cg00953309	0.04013122	0.09784894	0.01063099	0.90061396
cg20131013	0.04013682	48.6880638	1.1911035	1990.19444
cg07253870	0.04014161	3791.1925	1.4487572	9921014.04
cg15735240	0.04014187	198.98048	1.26885479	31203.9107
cg27236007	0.04014665	14.1644421	1.12657556	178.089627
cg04691829	0.04015002	8.04352529	1.09823714	58.9110463
cg13359765	0.04015355	34.5460711	1.17251596	1017.83777
cg20583141	0.04016502	7.37657698	1.09381587	49.7468445
cg04607699	0.04016694	0.18929265	0.03860991	0.92804421
cg24812891	0.04017078	196.838877	1.26730993	30573.0608
cg23538718	0.0401722	6.02028698	1.08382161	33.4407942
cg13582446	0.0401727	103389.613	1.67816896	6369687650
cg16092895	0.04017426	36.4377224	1.17490853	1130.05189
cg08769189	0.04018801	14.3187259	1.12653033	181.997685
cg08527546	0.04019021	205401833	2.35514476	1.7914E+16
cg04589156	0.04019064	317862077	2.40152426	4.2072E+16
cg12502785	0.04019123	1056300181	2.53397023	4.4032E+17
cg22755785	0.04019989	0.00373807	1.79E-05	0.77889706
cg10354495	0.04020891	5.75460913	1.08129775	30.6257239
cg16645539	0.04020923	214350.145	1.7302176	2.6555E+10
cg26243751	0.0402194	9.65805184	1.10646976	84.3023175
cg00327383	0.04022775	12.338487	1.11851449	136.107546
cg09125316	0.04022944	0.18937992	0.03862548	0.92852567
cg26163234	0.04023274	4.57363015	1.07007578	19.548235
cg18815765	0.04023591	1224663.36	1.86692898	8.0335E+11
cg15001364	0.0402445	2844.09551	1.42455196	5678191.79
cg05401069	0.04025757	5.87866996	1.08188188	31.943192
cg13981545	0.04026228	0.00033049	1.56E-07	0.70052132
cg24562149	0.04026284	12.1013303	1.11707956	131.093791
cg22545356	0.04026898	109.50779	1.23169523	9736.13909

cg22325715	0.04027068	0.08448161	0.0079642	0.8961532
cg17205313	0.04027119	6.29855078	1.08507313	36.561353
cg01579216	0.04027838	0.01146507	0.00016024	0.82029357
cg07469744	0.04027915	29.6560382	1.16213212	756.781944
cg25504222	0.04028276	5824876886	2.70834897	1.2528E+19
cg02081402	0.04028392	123.139476	1.23769891	12251.2272
cg22238209	0.04028485	8.00620129	1.09653443	58.4562213
cg25207447	0.04028662	103.926269	1.2283577	8792.77217
cg11558169	0.0402883	3714.54423	1.43909603	9587851.34
cg19016652	0.04028951	5.3973373	1.07750579	27.0358176
cg05740418	0.04029621	5.95499879	1.08214522	32.7701033
cg14840429	0.04029629	7.45140931	1.0929313	50.8023706
cg20092036	0.04029792	9.9706908	1.1070866	89.7984625
cg01308931	0.04030839	1.1658E+11	3.08326121	4.41E+21
cg11195360	0.04031051	0.28587434	0.08637247	0.94618275
cg06327965	0.04031251	0.03350965	0.00130461	0.86071499
cg10468369	0.04032064	55692.7837	1.61967953	1914999908
cg00396407	0.04032188	3.1406E+11	3.21578994	3.07E+22
cg07744751	0.0403234	71.5293198	1.20729316	4237.9463
cg09462826	0.04033243	238.062979	1.27276386	44528.2775
cg21197066	0.04033296	155976601	2.29652803	1.0594E+16
cg19458266	0.04033761	100.592179	1.22520662	8258.84083
cg15080939	0.04034558	20.1591317	1.14132665	356.068607
cg12406391	0.04035105	3.12123073	1.05133813	9.2663635
cg01909364	0.04035638	6.16069653	1.0832057	35.0387575
cg13078926	0.04035697	128.442238	1.23791176	13326.8048
cg00393376	0.04035933	0.00723178	6.49E-05	0.80524084
cg24034106	0.04036101	1.35E-07	3.65E-14	0.49907495
cg02034887	0.04037033	3.74695217	1.05969232	13.248799
cg04387010	0.04037552	32.1970599	1.16451047	890.202959

cg20382695	0.04037996	7.48999976	1.09230103	51.3595566
cg05774290	0.04039915	30.4490301	1.16121445	798.425678
cg16043990	0.04040233	8366797374	2.71645056	2.577E+19
cg00210994	0.04040728	7.91701394	1.09466407	57.2587621
cg23197860	0.04041324	1.4518E+10	2.77933503	7.5835E+19
cg21767036	0.04041485	0.02094543	0.00051941	0.84463041
cg06720722	0.0404224	4.06133877	1.06307568	15.5158027
cg02808338	0.04042492	5306857.4	1.9652216	1.4331E+13
cg02211646	0.04043261	6.47843454	1.08486441	38.6869676
cg08351100	0.04044126	4.11198679	1.0635154	15.8986277
cg09069694	0.04044186	13.014687	1.11823664	151.472481
cg11806890	0.04044313	235797674	2.3151234	2.4016E+16
cg26183265	0.04045044	54.3043106	1.18981978	2478.49146
cg13485746	0.04046407	205759.394	1.70150781	2.4882E+10
cg25479708	0.04046551	0.01364676	0.00022442	0.82983343
cg00442267	0.04046594	78.5484282	1.20869174	5104.57331
cg10027270	0.04048455	1.0965E+14	4.06052221	2.96E+27
cg02882624	0.04049	72368695.3	2.19017062	2.3912E+15
cg22049899	0.0404909	6.29103238	1.08292264	36.5465519
cg12850793	0.04049176	72071.5796	1.62330089	3199845829
cg10779981	0.04049191	2820648.77	1.90270855	4.1814E+12
cg01437411	0.04049275	15.6944592	1.12663802	218.62927
cg01296532	0.0404952	134.425742	1.23638477	14615.418
cg12778476	0.04049802	3.9670649	1.06145837	14.8263977
cg01044722	0.04050698	769999.994	1.79691516	3.2995E+11
cg03774463	0.04052686	5.70427486	1.0780175	30.1838807
cg07612928	0.04053435	7.2383146	1.08907515	48.1079735
cg06991565	0.04053974	10.18204	1.1051482	93.8099874
cg13653153	0.04054185	2763.32394	1.40680024	5427891.61
cg23848647	0.04054733	3149348.35	1.90426549	5.2085E+12

cg24884519	0.04055414	3.1472838	1.05055391	9.42873579
cg06277849	0.04055615	16.2040684	1.12724808	232.93172
cg03496709	0.04056983	8.17064079	1.09438911	61.0014941
cg07357346	0.04057382	6.27898415	1.08204609	36.4361948
cg23615572	0.04057523	63892.2196	1.60775408	2539079683
cg26987699	0.04057993	5.32131676	1.07433542	26.3571428
cg15819780	0.04057997	21.0975483	1.13971897	390.540614
cg25139493	0.04058098	8.54920632	1.09639448	66.6629849
cg04356968	0.04058838	895.485432	1.33813095	599264.337
cg23655816	0.04058951	54608402.1	2.14539732	1.39E+15
cg21721432	0.04060811	14823.0732	1.50777524	145726957
cg26484631	0.04061823	14.0070981	1.11933239	175.281981
cg05771157	0.04062113	0.23780988	0.06013005	0.94052037
cg11511443	0.04062114	4.18210284	1.06299286	16.4535293
cg10165864	0.04062259	5.85349575	1.07834908	31.7739526
cg09369490	0.04062307	20314445.2	2.0508718	2.0122E+14
cg21646955	0.04062897	4.08598076	1.06188235	15.7223056
cg27452691	0.04062999	18.54826	1.13265205	303.745488
cg08589721	0.04063291	74.073383	1.20148989	4566.71848
cg16843689	0.04063695	4.4787E+13	3.81773063	5.25E+26
cg23758931	0.0406377	1.2199E+12	3.27393391	4.55E+23
cg15597540	0.04064004	31647.2433	1.55501945	644074262
cg04912712	0.04064645	3.34905345	1.05280554	10.6535904
cg09611962	0.04064768	1.4698E+10	2.70896493	7.9743E+19
cg17374636	0.04065394	6202.61613	1.44987566	26534997.4
cg01542384	0.04065577	0.11894537	0.01548889	0.91342873
cg10687642	0.04066939	262500.762	1.69872501	4.0564E+10
cg11553177	0.04067083	973140.704	1.79575313	5.2736E+11
cg00023464	0.04067296	0.09290151	0.00954665	0.90405459
cg06422261	0.04067819	18.392029	1.1314866	298.957789

cg06276088	0.04068068	0.17575274	0.03325285	0.92891376
cg16682385	0.04068767	4855900389	2.57325949	9.1634E+18
cg16923670	0.04069922	121018974	2.19831466	6.6622E+15
cg10561067	0.04070347	6.38675106	1.081597	37.7132973
cg02192967	0.04070654	2.94625529	1.04675375	8.29270516
cg20689586	0.04070679	4286.50187	1.42426278	12900778.3
cg26884261	0.04070936	4016214.14	1.90183925	8.4813E+12
cg12680131	0.04071054	0.42505425	0.18732425	0.96448333
cg15646359	0.0407126	731.401468	1.32140779	404831.96
cg08951271	0.04071413	14.1924243	1.11860453	180.068026
cg15997635	0.04071453	24640298.2	2.05256539	2.958E+14
cg09495769	0.04071459	4.76319636	1.06817157	21.2400707
cg02545393	0.04071595	14206556.8	2.00513873	1.0065E+14
cg03851496	0.04072082	15.0429093	1.1212617	201.816507
cg13397649	0.04072133	5474.90347	1.43822044	20841428.2
cg20910746	0.04072267	7.35722415	1.08789025	49.7557057
cg09033857	0.04073012	9.8831E+13	3.8926541	2.51E+27
cg11758345	0.04073716	8.98378346	1.09693512	73.5762435
cg13354988	0.04074316	17.3806038	1.12777823	267.858857
cg11383961	0.04075097	26852258.4	2.05393079	3.5106E+14
cg06382770	0.0407549	23.3674601	1.14172317	478.25796
cg25390230	0.0407569	342875457	2.28501123	5.145E+16
cg08872703	0.04075711	6.4336424	1.08141761	38.2754584
cg20673255	0.04075879	1761.98805	1.36917762	2267493.9
cg14711866	0.04076265	5.17917339	1.07155361	25.0326597
cg15156836	0.04076393	87.1740224	1.20649391	6298.67263
cg20209308	0.04076533	3.91638529	1.05902462	14.4832078
cg23242944	0.04077015	22.2433218	1.13910176	434.346941
cg00606312	0.04077276	49.4926262	1.17794422	2079.48731
cg21171339	0.04077702	6.51679166	1.08180953	39.2569785

cg11056224	0.04077837	58.1199058	1.18578683	2848.67682
cg10981962	0.0407805	0.09226761	0.00940806	0.9048952
cg19895185	0.04078397	96.201235	1.21097177	7642.35619
cg16332577	0.04078537	174.522438	1.24154479	24532.4064
cg00212119	0.04078716	0.28846054	0.08765921	0.94923833
cg23526676	0.04079316	11.8993475	1.10927292	127.646198
cg19184415	0.0407963	20.7932922	1.13545359	380.78263
cg17910586	0.04079791	20.6195267	1.13502855	374.585188
cg13586425	0.04079981	11.8059096	1.10882016	125.700727
cg12104984	0.04080167	289633080	2.25945791	3.7127E+16
cg06240896	0.04080275	3057.26327	1.39891761	6681493.35
cg18095732	0.04080322	8523.22382	1.46018909	49750641.8
cg23103688	0.0408074	7.61842725	1.0886019	53.3164916
cg12978308	0.04080981	16.5794953	1.12453749	244.437973
cg06000635	0.04081292	23.9188602	1.14184265	501.042655
cg25404454	0.04082383	207.044236	1.24924938	34314.4581
cg04974751	0.040824	379651.679	1.70931815	8.4323E+10
cg15692031	0.04082705	3.7922E+10	2.76244875	5.21E+20
cg21887246	0.0408348	931226.082	1.77327856	4.8903E+11
cg16191009	0.04083694	16.4238475	1.12368841	240.051215
cg14671364	0.04083971	25.2026131	1.14386934	555.283446
cg16681199	0.04084177	10.2822475	1.10191489	95.9462615
cg21444002	0.04084366	0.00976295	0.00011558	0.82469802
cg07911225	0.04084708	17.6225048	1.12683565	275.597132
cg14283602	0.04085358	8466464.86	1.94138985	3.6923E+13
cg16532893	0.04086292	5766115.29	1.90930181	1.7414E+13
cg13455960	0.04086419	8.87035671	1.09490294	71.8631992
cg09137453	0.0408671	20.5076554	1.13364289	370.984492
cg01676959	0.04087036	799.703644	1.31976578	484575.313
cg07419459	0.04087809	270448421	2.23714995	3.2694E+16

cg27045356	0.04087954	3118585.54	1.85897117	5.2317E+12
cg11042320	0.04088455	9.593803	1.09823421	83.8082212
cg00566982	0.04088543	7.35229304	1.08618063	49.7672405
cg24699146	0.0408977	4.50923556	1.06430475	19.1046835
cg04656757	0.04089804	1.56E-06	4.23E-12	0.57507156
cg24093411	0.04089855	8.71362761	1.09370573	69.4220613
cg27636594	0.0409023	21.3493907	1.13495682	401.598085
cg19929126	0.04090565	4.47990112	1.06395705	18.8630867
cg09424275	0.04090997	46232.1773	1.55868321	1371294823
cg26284300	0.04091507	0.00396742	1.98E-05	0.79584282
cg03465028	0.04091612	2.98083539	1.04613086	8.49356421
cg14183922	0.04092682	6.2326585	1.07838215	36.0225102
cg18540492	0.04093216	11.4943379	1.10587979	119.470312
cg01268571	0.04094029	4.68286884	1.06563704	20.5785457
cg17255450	0.04094307	4.2223023	1.0610837	16.8015366
cg04626931	0.04094313	7.17421396	1.08449172	47.4594181
cg07758574	0.04094406	5.7038101	1.0742928	30.2835965
cg27277974	0.04094702	1789.51672	1.36091885	2353094.09
cg03054576	0.04095085	3690214.67	1.86242479	7.3118E+12
cg12649539	0.04095351	336.561644	1.27027252	89172.787
cg22476252	0.04095395	7.01844956	1.08340377	45.4665524
cg02112027	0.04095544	20.3871832	1.13193248	367.192605
cg00251405	0.04095993	9.31633718	1.09602303	79.1900685
cg18509435	0.04096639	0.26530815	0.07432889	0.94698587
cg14601444	0.04097634	7.98270593	1.0889124	58.5204044
cg05043716	0.04099053	0.0897868	0.00889771	0.90603862
cg00358323	0.0409938	3233.47684	1.39195138	7511305.78
cg16385583	0.04099664	0.11442661	0.01430766	0.91513539
cg26757722	0.04099678	0.19054991	0.03885716	0.93442921
cg20813773	0.04100079	7662.72362	1.44154929	40732102.3

cg00240569	0.04100102	76381.2579	1.58363871	3683982029
cg08293536	0.04100556	867.880089	1.31851429	571261.043
cg18324542	0.04101627	202181.451	1.64648556	2.4827E+10
cg27264388	0.04101792	0.02053599	0.00049419	0.85336986
cg23684878	0.04102279	3.14751502	1.0478751	9.45422869
cg20609803	0.04102861	0.01784047	0.00037504	0.84865695
cg27287305	0.04104544	6.45682114	1.07882107	38.6445357
cg01919895	0.04104573	27.5211648	1.14435255	661.871651
cg08132837	0.04104929	7.18064928	1.08345492	47.5900964
cg26771969	0.04105502	53.4964294	1.17550669	2434.58244
cg14240820	0.04106521	0.18198603	0.03549003	0.93318918
cg22940268	0.04107774	31.1407974	1.14951841	843.613512
cg17695512	0.04108284	0.22245484	0.05259208	0.94094301
cg21276549	0.0410857	0.03696917	0.00156193	0.87501804
cg11178810	0.04108672	3308527897	2.42867753	4.5071E+18
cg14190534	0.04108766	5.17826197	1.06882872	25.0876465
cg23094080	0.04109183	7467.08974	1.43448959	38869176.6
cg12384236	0.04109279	7.02604182	1.08206117	45.6215094
cg04302492	0.04110018	10935.633	1.4562666	82119626.1
cg07950803	0.04110031	0.24923424	0.06570566	0.9453935
cg20822606	0.0411061	66359628.1	2.06978051	2.1276E+15
cg19872537	0.04110909	44359.343	1.5403678	1277455497
cg01373705	0.04111022	46257.0814	1.54288649	1386827606
cg06074332	0.04112188	3978274.61	1.84529578	8.5768E+12
cg21111471	0.04112836	5.45488831	1.07073208	27.7901512
cg07650391	0.04112982	4.76030711	1.06486183	21.2802481
cg11586189	0.04113193	7.67859897	1.08554508	54.3145403
cg04522003	0.041135	10.1757857	1.09788617	94.3145268
cg26522057	0.04114334	2675815.62	1.81329422	3.9486E+12
cg20495404	0.04114881	10.1464383	1.09759211	93.7964195

cg09113530	0.04115006	17.6238438	1.12219926	276.777824
cg16564710	0.04115083	508.02606	1.28444683	200935.119
cg17805404	0.04115129	68.6183263	1.18516786	3972.83361
cg16175263	0.04115945	9.89865271	1.0963753	89.3702417
cg03784679	0.04116333	33869181.7	2.00485917	5.7217E+14
cg18117600	0.04116861	3.65601511	1.05335054	12.6894571
cg26934993	0.04117162	3.87694695	1.05581099	14.2361823
cg02630854	0.04117216	23.040687	1.13397678	468.151787
cg15604693	0.04117311	514.509041	1.28425503	206126.936
cg08244301	0.04117684	6.39066274	1.07712396	37.9163138
cg14386850	0.04118655	73261.9881	1.56543947	3428633947
cg10465680	0.04118778	1166.44202	1.32642465	1025755.2
cg16421340	0.04118922	10.6493354	1.09923003	103.170712
cg11785652	0.04119149	2.92195808	1.04380647	8.17952295
cg25104555	0.04119323	2.84910784	1.04274425	7.78466584
cg02017200	0.04119551	53354.6203	1.5449915	1842544445
cg13850354	0.04119799	1.08E-06	2.02E-12	0.5776092
cg23047335	0.04120568	124.467275	1.21235579	12778.5117
cg16719517	0.04120854	9.29054524	1.09302226	78.9684109
cg01342926	0.04120871	42854.3834	1.53049757	1199935374
cg14871584	0.04120906	59.44499	1.17703781	3002.20334
cg16760485	0.04121327	12.4142245	1.10567525	139.383576
cg08582356	0.04121767	4.74097116	1.06399794	21.12486
cg00821310	0.04122608	0.03410454	0.00133061	0.87412743
cg21037008	0.04123057	3.9235834	1.05591402	14.5793184
cg01409659	0.04124219	1165.17024	1.32395573	1025428.32
cg06836497	0.04124382	7.7814E+10	2.70882291	2.24E+21
cg27102995	0.04125444	78.2952988	1.18893099	5156.02158
cg01116067	0.04125613	9.86476677	1.09507618	88.8647064
cg21167563	0.04125772	32.3897937	1.14794231	913.895004

cg10775792	0.04126511	9.32253078	1.0925187	79.5497417
cg03057164	0.04127395	94353826.1	2.06898574	4.3029E+15
cg17703407	0.04127917	21434.1406	1.48382144	309621072
cg24921634	0.0412863	85347.4801	1.56660842	4649657356
cg13338877	0.04129397	14.0469202	1.11001627	177.759527
cg21561492	0.04129627	5.34568655	1.06843512	26.745999
cg23230554	0.04131165	0.12800703	0.01776876	0.92216895
cg03344031	0.04132688	4.12689027	1.05735593	16.1073701
cg19930885	0.04133114	244.834937	1.24148132	48284.3724
cg04946916	0.04133259	23197.3471	1.48469375	362443037
cg12237591	0.04133418	1385392.59	1.74350534	1.1008E+12
cg20777128	0.0413405	14.5630874	1.1109445	190.90379
cg01298678	0.04134165	5.97945664	1.07276077	33.3288677
cg10248148	0.04134616	4118.24794	1.38639965	12233100.4
cg05855489	0.04135326	81.558949	1.18841176	5597.27056
cg09313482	0.04135753	5.36840467	1.06809385	26.9824311
cg08996521	0.04136053	604.72716	1.28527395	284526.841
cg26541027	0.04136536	28244.852	1.49385643	534035033
cg10339200	0.04136539	46630.0611	1.52347344	1427240242
cg13052543	0.04136982	589.446491	1.28362591	270676.343
cg01022087	0.04137329	0.07429039	0.00610998	0.90328646
cg18398007	0.04137721	1.8591E+11	2.75867031	1.25E+22
cg06289138	0.04138067	4.73375029	1.06265842	21.0871071
cg00518698	0.04138249	83857598.6	2.0401502	3.4469E+15
cg19166626	0.0413848	394981.169	1.65447553	9.4296E+10
cg11943056	0.04139324	5.211086	1.06655202	25.4609402
cg11025037	0.04139409	0.19098243	0.03890873	0.93743189
cg05409131	0.0414005	149.63796	1.21567719	18418.9678
cg02442619	0.04140369	10.1949366	1.09473221	94.9426085
cg09015232	0.04140662	0.19510342	0.04056824	0.93830401

cg22234827	0.04141149	726228.417	1.69143346	3.1181E+11
cg17489534	0.04141662	23040.4801	1.47838646	359083188
cg10642449	0.04142039	17090.461	1.46104405	199914478
cg25318809	0.04142085	3.88904393	1.0542531	14.3463298
cg24496666	0.04142258	5.86575212	1.07122698	32.1192881
cg07485417	0.0414306	10.0865012	1.09395448	92.9997616
cg15134506	0.04143344	6.24678804	1.07375135	36.342083
cg15034464	0.04143411	8.1732E+11	2.90165444	2.30E+23
cg10905319	0.04143563	1.7741E+12	2.98971234	1.05E+24
cg14980255	0.04145134	15.4285097	1.11187149	214.08851
cg27284088	0.04145242	185.139382	1.22424249	27998.204
cg07264792	0.0414582	161310.588	1.59095993	1.6356E+10
cg18770763	0.04146301	28.6688231	1.13868341	721.799763
cg07836663	0.04146321	5.14038502	1.06540674	24.8013807
cg09961689	0.04146979	12.6285852	1.10303192	144.584361
cg04244056	0.04148517	187047.64	1.59763359	2.1899E+10
cg22752895	0.04148848	25264197.8	1.93015763	3.3069E+14
cg21058987	0.04148905	4271.02816	1.38056943	13213157.6
cg11213987	0.04149453	294578.64	1.62498192	5.3402E+10
cg00980538	0.04151869	5440920.94	1.81511961	1.6309E+13
cg06799321	0.04152462	19.1665498	1.12011457	327.963443
cg17391620	0.04152581	8.88277495	1.08749773	72.5552692
cg12411312	0.04152637	794.950217	1.29234331	488992.239
cg16581360	0.04152665	109883.442	1.56161352	7731984004
cg24927124	0.04152674	3836944299	2.33358869	6.3088E+18
cg11800672	0.04152963	4.37076293	1.05825087	18.0520225
cg14844619	0.04154048	839098.486	1.68690195	4.1738E+11
cg25882830	0.04154077	8.5253705	1.08561973	66.9497251
cg07185041	0.04154319	5.94142753	1.07067406	32.9704085
cg23730696	0.04154983	8.76716586	1.08668333	70.7319189

cg12849291	0.04155324	806295.015	1.6829471	3.8629E+11
cg13968099	0.04155872	170.956061	1.21732267	24008.4042
cg24494412	0.0415619	1094721.15	1.70179402	7.0421E+11
cg27061971	0.04156323	0.05004708	0.00280852	0.89182606
cg20612286	0.0415661	6.70326431	1.07541446	41.7827305
cg27370696	0.04156843	27.3586782	1.13475385	659.612011
cg16745967	0.04157425	2350.58509	1.34492256	4108229.31
cg04271677	0.04157432	0.00040546	2.22E-07	0.74217624
cg08101375	0.04157858	4.14942882	1.05579487	16.3078644
cg09807565	0.04159316	47.6092141	1.15850379	1956.52124
cg01875529	0.0415941	26248.6899	1.47329242	467655785
cg18246056	0.0415947	6.87862309	1.07619628	43.965452
cg12659494	0.04159852	6.42398418	1.07336164	38.4470354
cg11127732	0.04161424	4.90613035	1.06228053	22.6589063
cg10770023	0.04161608	6.9381614	1.07633925	44.7238952
cg16781647	0.04161731	2.96704508	1.04216241	8.44720213
cg24058132	0.04161785	39.7341843	1.15005989	1372.80277
cg15694117	0.04162506	0.19917715	0.04217578	0.94062355
cg06084073	0.04163824	13.0272835	1.10210748	153.9869
cg20559657	0.04164945	8.54793353	1.08453619	67.3718116
cg14452896	0.04165304	1275791662	2.20920964	7.3675E+17
cg26515676	0.04165751	95125.7616	1.54204101	5868138680
cg12754671	0.04166288	1776.48597	1.32646697	2379179.03
cg03853987	0.04166293	4.38454326	1.05739567	18.1807248
cg15717808	0.04166353	25.4428553	1.12996988	572.88154
cg16148454	0.04169101	3.71770773	1.05064688	13.1550866
cg07944724	0.04169213	75.3307324	1.17655371	4823.17057
cg08024097	0.04169906	26694.5854	1.46682306	485812440
cg06683487	0.0416994	0.12965183	0.01815115	0.92608975
cg22601577	0.04170041	443257.441	1.63007333	1.2053E+11

cg07077277	0.04170339	2.5217034	1.03535771	6.14182709
cg00770008	0.04171131	1739666.78	1.71475713	1.7649E+12
cg14390179	0.04171554	780.865813	1.28380721	474955.597
cg00920938	0.04171588	0.12164108	0.01601318	0.92402362
cg26002632	0.04171663	11.5918048	1.09625239	122.572082
cg00789347	0.04171881	29.7056873	1.13559414	777.062705
cg13327503	0.04172002	13.2257047	1.10164195	158.780504
cg02216481	0.0417208	9.77121166	1.08920134	87.6574181
cg24058604	0.04172635	3.42926681	1.04724433	11.2293479
cg21302562	0.0417276	3948072.06	1.76625737	8.825E+12
cg16362201	0.04175721	0.02305557	0.00061185	0.86877914
cg06213964	0.04175941	5.41405731	1.06503216	27.5221892
cg00990763	0.04176131	1783.13998	1.3220575	2405030.17
cg26954499	0.04176298	436465218	2.09967534	9.0729E+16
cg10285525	0.04176461	15.7951149	1.10835617	225.095201
cg05218653	0.04177057	0.17657221	0.03325809	0.93744847
cg14377982	0.04177393	7519886.88	1.80316009	3.1361E+13
cg24044028	0.04177832	7.51888092	1.07796654	52.4446431
cg10630880	0.04177833	289.297188	1.23480028	67778.4615
cg27339505	0.04180226	5.2957E+10	2.49961587	1.12E+21
cg01708273	0.04182324	5.03788168	1.06165912	23.9062155
cg10552592	0.04182352	22321.3157	1.44846764	343978090
cg09927483	0.04182367	2910.61866	1.34329056	6306677.94
cg02186542	0.04182887	6.79577508	1.07342841	43.0234177
cg07089892	0.0418445	8.04576277	1.07998544	59.9399736
cg04510815	0.04184761	11.078836	1.09277154	112.320465
cg16439198	0.04185068	5.67318295	1.06609603	30.1895926
cg14976964	0.04186021	696.263679	1.27261197	380935.528
cg17188367	0.04186063	90.9112953	1.18067332	7000.12737
cg04570419	0.04186907	1589.96006	1.3115049	1927536.07

cg03687765	0.04187371	64.046987	1.1652431	3520.30967
cg23337289	0.04187476	14.9768146	1.10460973	203.062646
cg24718465	0.04187619	10.482822	1.0902003	100.797585
cg03011535	0.04188106	3.28401419	1.04464315	10.3238596
cg12043747	0.0418822	1510.89348	1.30845441	1744653.16
cg26443922	0.04188516	4100.16839	1.3571611	12387166.8
cg05227350	0.04188709	10.2148114	1.08903336	95.8119151
cg18672998	0.04189033	0.06810994	0.0051195	0.90613678
cg16644457	0.04190477	6.90723684	1.07333364	44.450224
cg10273340	0.0419156	3.22632289	1.04376474	9.97270648
cg03671276	0.0419165	30.3199431	1.132865	811.481469
cg03203223	0.04191963	4.16436237	1.05352335	16.4608729
cg22040403	0.04192002	523.320186	1.25708447	217856.495
cg02264779	0.04193079	15.2536838	1.10456071	210.649236
cg01087645	0.04193391	5.5733E+10	2.46628515	1.26E+21
cg11201571	0.04193835	23.5526849	1.12208722	494.372412
cg21919602	0.04193883	100667944	1.95784881	5.1761E+15
cg13990746	0.04194481	3.62834883	1.0480713	12.5610874
cg18649459	0.04194535	17.7836098	1.11054569	284.776015
cg27249554	0.04195887	9.23281718	1.08418758	78.6256131
cg23912429	0.04196722	14.6110669	1.10232084	193.667096
cg21107197	0.04197504	10.3481205	1.08849988	98.3772256
cg07357021	0.04197754	0.12621757	0.0171731	0.92766466
cg23250439	0.04198624	4003.65124	1.35064202	11867854.6
cg25705508	0.04198971	0.20587443	0.04488139	0.94436214
cg03605784	0.04199606	1.1099E+12	2.72848821	4.51E+23
cg14377152	0.04199965	5.16114136	1.06116394	25.1020405
cg14252395	0.04200336	68.9961871	1.16542586	4084.7505
cg13658921	0.04200912	206363611	1.99709839	2.1324E+16
cg13245912	0.04202375	174.558606	1.20460623	25295.1595

cg04724058	0.04202816	674343.964	1.62207384	2.8034E+11
cg13179508	0.04203352	2775396.81	1.70628937	4.5144E+12
cg06080729	0.04203462	0.13842235	0.02057486	0.93126963
cg26070636	0.04203472	113165.515	1.52047131	8422673741
cg27563174	0.04205176	117959.616	1.52132313	9146295531
cg23609650	0.04205568	76.9506491	1.16878515	5066.28819
cg02004672	0.04206065	86.1573716	1.17341606	6326.05343
cg20387815	0.04206076	3.56002786	1.04662179	12.1092437
cg06090660	0.0420628	4.0992405	1.05191819	15.9744102
cg16236672	0.04206919	10.7381149	1.08881895	105.901088
cg09059767	0.04207081	5.0350355	1.0596425	23.9246562
cg19839026	0.04207365	13.5867275	1.09798161	168.125916
cg26740494	0.04207456	6.70015318	1.07051281	41.9350913
cg21307628	0.04207815	11.382763	1.09098531	118.761722
cg25257905	0.0420823	0.274048	0.07866308	0.9547339
cg12809031	0.0420864	8.24575223	1.07837575	63.0507778
cg05993265	0.04209055	13.5740556	1.0977179	167.852764
cg03100639	0.04209548	6.74050732	1.07054255	42.440573
cg18540455	0.04209664	5.9147E+10	2.4252362	1.44E+21
cg25250998	0.04209685	86.164145	1.17253139	6331.82184
cg01729401	0.04209773	345.079694	1.23207451	96649.995
cg21370778	0.0420988	812757.94	1.62567401	4.0634E+11
cg00907427	0.04210052	0.30143947	0.09484028	0.95809249
cg00411072	0.04210223	7.55295304	1.07483487	53.0752224
cg12789279	0.04210284	8.66055309	1.08009061	69.4434145
cg02434836	0.04210372	0.14579266	0.0227674	0.93359368
cg18729488	0.04210492	283188.107	1.56504385	5.1242E+10
cg05341097	0.0421052	54.5185399	1.15333787	2577.10361
cg08728761	0.04210536	773059.06	1.62209205	3.6843E+11
cg01353524	0.04211594	4753.97396	1.35208792	16715087.8

cg05943971	0.04211816	1752174961	2.13418009	1.4385E+18
cg08250787	0.0421188	0.09901541	0.01064567	0.92094293
cg11526413	0.04212486	3.12673897	1.04140164	9.38782529
cg26310240	0.04212977	22825545.9	1.82678006	2.852E+14
cg18146964	0.04213138	21488.9476	1.42571625	323889743
cg19309549	0.04213342	98971379.1	1.92400396	5.0911E+15
cg05027458	0.0421414	4.43703314	1.05433141	18.6727466
cg05783384	0.04214797	11.231934	1.0896009	115.782155
cg07891064	0.04214844	3.83672107	1.04885665	14.0347383
cg27600205	0.042154	3.16356457	1.04167169	9.60776887
cg25814383	0.04215646	39.4598203	1.13910564	1366.92977
cg01012308	0.04216085	6.4027E+15	3.6292336	1.13E+31
cg15835620	0.0421658	4.30808963	1.05305195	17.624616
cg27505984	0.04216946	2.8062E+11	2.54095452	3.10E+22
cg19651155	0.04217124	1125864905	2.08995033	6.0651E+17
cg23601586	0.0421797	208.926503	1.20770914	36143.0433
cg09939831	0.04218439	76665.0424	1.48750719	3951260720
cg16116203	0.04218454	9.16389671	1.08135229	77.6592451
cg18080370	0.04218702	5.6544218	1.06305335	30.0760878
cg09462575	0.04219202	18.6418423	1.10869315	313.448571
cg12654349	0.04219337	114668.143	1.50806227	8718992057
cg02151625	0.04219364	5.57132663	1.06244147	29.2154263
cg01486387	0.04219671	5855.51358	1.35769649	25253832.1
cg00585901	0.04220114	5.03385511	1.05858735	23.9372758
cg22624697	0.04220311	20.5601721	1.11235515	380.023121
cg23398241	0.04221052	34.640328	1.13284118	1059.24144
cg06390079	0.04221255	3.53433114	1.04540978	11.9488997
cg08726338	0.04221274	8.3562229	1.0775331	64.8021497
cg01269469	0.04221744	223.389245	1.20941071	41262.0417
ch.12.262555	0.0422186	0.00012508	2.15E-08	0.72917327

cg20704635	0.0422201	195.24582	1.20362047	31671.8857
cg26464934	0.04222621	12.2803167	1.09205098	138.094449
cg27434351	0.04222661	2992054641	2.15124735	4.1615E+18
cg02377073	0.04222719	137777.432	1.51501396	1.253E+10
cg26298967	0.04223967	6.62362641	1.06850726	41.0595494
cg05565809	0.04224715	5.74335144	1.06311534	31.0277583
cg14099293	0.04225667	102234584	1.90581066	5.4842E+15
cg02015348	0.04227056	28.6114343	1.1241845	728.184894
cg03810376	0.04227148	7.55777421	1.07313608	53.2271276
cg12740148	0.04227729	1088301.98	1.62371666	7.2944E+11
cg20532887	0.04227921	2297.53224	1.30973017	4030337.32
cg00479347	0.04228006	270.117628	1.21551497	60026.8487
cg23181776	0.04228226	21017.6142	1.41460208	312271637
cg10514594	0.04229077	260760.855	1.54358421	4.4051E+10
cg17135941	0.04229368	0.00091794	1.07E-06	0.78401064
cg16184495	0.04229418	5.30887712	1.0598016	26.5938231
cg23685529	0.04229608	2.9337E+13	2.94065531	2.93E+26
cg20425447	0.04230128	291356876	1.96886612	4.3116E+16
cg08180459	0.04231079	8.18E-06	1.00E-10	0.66587191
cg25141818	0.04231113	0.02912001	0.00095873	0.8844788
cg20949959	0.04231326	5401992.79	1.71253828	1.704E+13
cg21878918	0.04231466	109.823853	1.17707734	10246.8022
cg09386954	0.0423169	1427652.61	1.63485475	1.2467E+12
cg27109748	0.04231881	0.25213156	0.06668133	0.95334522
cg07834574	0.04232007	5.97197411	1.06391988	33.5217675
cg21961890	0.04232071	1307331.42	1.62946118	1.0489E+12
cg27660248	0.04232716	4303.87854	1.33619428	13862782.4
cg18773129	0.04233534	130.604532	1.183618	14411.3589
cg05496363	0.04234049	4.58711786	1.05407896	19.9621196
cg19878987	0.04234539	19.8439199	1.10875737	355.155391

cg02847589	0.0423516	2484243.52	1.66259064	3.712E+12
cg04271218	0.04235168	8.5724598	1.07699528	68.2334158
cg22448889	0.04235278	3.64230462	1.04562903	12.6874662
cg18000216	0.04235887	0.08092328	0.00714179	0.91693782
cg06904356	0.04236381	0.20492801	0.04435365	0.94683283
cg26188072	0.04236745	507760.368	1.57237701	1.6397E+11
cg20613442	0.04237301	85.2728859	1.16537347	6239.60066
cg05013728	0.04237604	4.56341366	1.05362402	19.7648722
cg08769300	0.04238441	10.6269828	1.08462057	104.121909
cg02070114	0.04238795	37125.6338	1.43543847	960203251
cg02216246	0.04239026	3.66499648	1.04561506	12.8462183
cg05718036	0.04239676	31.9470319	1.12621305	906.23426
cg00323631	0.042398	31155359.2	1.80749519	5.3702E+14
cg15407517	0.04239986	20209806.8	1.78059439	2.2938E+14
cg16721449	0.04241354	3.24E-10	2.22E-19	0.47331755
cg05534152	0.04241552	322.088917	1.21852498	85136.7612
cg22184096	0.04242677	7325.58345	1.35540622	39592686
cg07758902	0.04242794	6107.32185	1.34694345	27691867.9
cg24281422	0.0424389	79716261.3	1.86021621	3.4161E+15
cg26710686	0.04245857	798550.299	1.58787407	4.016E+11
cg21120786	0.04246361	11484.9659	1.37417965	95987771.5
cg26565099	0.04246407	5.84968432	1.0618928	32.2243515
cg02837212	0.04246541	79.5622643	1.16039981	5455.14904
cg13372488	0.04246552	8.1561313	1.07394522	61.9421517
cg00481081	0.04246678	1.6827E+10	2.22602528	1.27E+20
cg01374870	0.04246961	0.06751766	0.00499576	0.91250081
cg25753915	0.04247397	39497194.1	1.81098047	8.6143E+14
cg06824394	0.04247919	3.39921474	1.04238479	11.0848325
cg10096454	0.04248475	9.04969652	1.0775337	76.004126
cg23752380	0.04248638	1989415.38	1.63487733	2.4208E+12

cg00519002	0.04249094	3.66892256	1.04501392	12.8811612
cg20395773	0.04249542	85.5609102	1.16254488	6297.10685
cg17285947	0.0424994	92017756.9	1.85967967	4.5531E+15
cg01522296	0.04249984	14.8658776	1.09560943	201.709029
cg22149995	0.04250763	5.65630617	1.06030584	30.1741236
cg00375829	0.04250783	433.336232	1.22774066	152947.846
cg24424217	0.04252083	8.3689898	1.07429607	65.1961709
cg17446896	0.04252723	4.47687647	1.05181538	19.0550769
cg00358132	0.04253158	17.8551628	1.10195071	289.311343
cg14612733	0.04254429	4.43366122	1.05134678	18.6973053
cg16512144	0.04254476	9.674705	1.07928912	86.7236733
cg19039291	0.04254609	608.179865	1.24046272	298181.269
cg24498636	0.04254717	0.1346896	0.01940576	0.93484034
cg24274165	0.04256196	10929787.6	1.72217495	6.9366E+13
cg05116002	0.04256896	3.20165026	1.03976189	9.85856906
cg12111295	0.04258157	0.13848175	0.02048823	0.93601034
cg26328002	0.04258828	0.00239162	7.00E-06	0.81733972
cg03547631	0.04259079	2.2693E+10	2.21792419	2.32E+20
cg19396878	0.04259292	3.58104544	1.043522	12.2890428
cg20234121	0.04259803	24.9610013	1.11334404	559.621789
cg21232161	0.04259945	5.71075944	1.05985713	30.7709147
cg09788352	0.04260008	87.098746	1.16070923	6535.82425
cg16092018	0.04260216	15225.1424	1.37879471	168121447
cg12484593	0.04260702	400.497238	1.22108133	131357.375
cg15616111	0.0426076	6885530.14	1.69001267	2.8053E+13
cg02555579	0.04260894	28.1852292	1.1176767	710.766494
cg07891774	0.042612	21.8579258	1.10819971	431.121678
cg00487727	0.04261448	1.5215E+13	2.7473018	8.43E+25
cg06055873	0.04261689	6.35930761	1.06350766	38.0258599
cg13677149	0.04261746	4.1911002	1.04884637	16.7472772

cg00705992	0.04262234	8.45382405	1.07357499	66.5693051
cg14398731	0.04262815	0.18079343	0.03459793	0.94474621
cg13995497	0.04262905	5.03242736	1.05515943	24.0014205
cg10250177	0.04262987	8.68793117	1.0744692	70.2487778
cg07052390	0.04263304	5.81357976	1.06019583	31.8787423
cg08857194	0.04264678	0.00556331	3.68E-05	0.84191895
cg11046380	0.04264829	0.12030935	0.01552656	0.93223088
cg05478348	0.04264919	31303.6607	1.40913841	695403066
cg27359972	0.04266006	180588.371	1.4924666	2.1851E+10
cg26247841	0.04267046	3.70914601	1.0442522	13.1747524
cg02690256	0.04267488	12.6614755	1.08741859	147.425252
cg11775521	0.0426885	3.58649125	1.04298079	12.3328441
cg00201196	0.04269414	0.00531637	3.36E-05	0.84162587
cg05745142	0.04269482	0.00920177	9.88E-05	0.85697826
cg24030449	0.04270509	6.38660361	1.06284821	38.376793
cg20521696	0.04271444	7.61720327	1.0689279	54.2803546
cg15077012	0.04272115	6696.95492	1.33499565	33595019.7
cg14677983	0.0427266	0.01385981	0.00022101	0.86916301
cg11245681	0.04272698	3.83548774	1.04503801	14.0769676
cg19435284	0.04272908	6.77813956	1.06470118	43.1512395
cg24277128	0.0427338	409675.078	1.52666567	1.0993E+11
cg18998911	0.04273815	4.91894014	1.05350578	22.9670996
cg24806195	0.04274106	79.2465624	1.15373453	5443.20853
cg11331344	0.04274843	28.2340129	1.11531473	714.739493
cg21435190	0.04275337	10.7968277	1.080773	107.859364
cg19510318	0.04275632	151108.333	1.47577297	1.5472E+10
cg07596663	0.04276134	28295.9032	1.39692195	573158823
cg00574958	0.04276394	8.42224319	1.07193212	66.1741348
cg22553461	0.04277104	1.5896E+10	2.14885195	1.18E+20
cg09099965	0.04277123	565694397	1.92762009	1.6601E+17

cg00204465	0.04278065	4.52561722	1.050323	19.4999169
cg04359753	0.04279479	4.96894538	1.05340867	23.4385941
cg10536955	0.0427951	3.41516072	1.04066468	11.2075705
cg02622809	0.04280273	9.94019367	1.0772911	91.7184318
cg03871329	0.04280276	261838.831	1.49843634	4.5754E+10
cg06775930	0.04280734	3.14782519	1.03784758	9.54745533
cg07588964	0.04280958	26327.6183	1.39045722	498500404
cg23359394	0.04281209	6.36084964	1.06172716	38.1081031
cg01423843	0.04281704	260000877	1.87168079	3.6118E+16
cg08404201	0.04281743	734831731	1.93558655	2.7897E+17
cg03623264	0.04281865	0.24499292	0.06281507	0.95552748
cg06659926	0.04282034	31.1449059	1.11760775	867.929887
cg26267388	0.04282039	7.73512255	1.06838759	56.0022614
cg17066716	0.04282618	2136022.32	1.60139621	2.8491E+12
cg06198975	0.04283128	56.525159	1.13912221	2804.8734
cg14058998	0.04283408	336.17797	1.2065247	93670.3803
cg21770462	0.04284153	4.17946509	1.04718389	16.6808605
cg21172458	0.04284664	4.45952189	1.04933835	18.9522621
cg04117801	0.04284836	3.67837153	1.04283826	12.9746075
cg01978368	0.04286397	1146592.37	1.56568004	8.3968E+11
cg16621855	0.04286501	0.14615744	0.02272344	0.94008644
cg24153199	0.04286645	1592801770	1.97506202	1.2845E+18
cg19006008	0.04287088	3.50673341	1.04109765	11.8117443
cg14183329	0.0428773	4.31585954	1.04801334	17.7732886
cg01986605	0.04288741	14.9178778	1.09040015	204.093036
cg25092681	0.04289156	3.4615668	1.04054018	11.5156002
cg00201025	0.04289548	9.60795102	1.07505361	85.8680176
cg09481970	0.04290253	5076412.42	1.63780455	1.5734E+13
cg10331779	0.04290271	5.74111821	1.05742965	31.1703369
cg00904122	0.04290517	15388.8776	1.36063582	174049183

cg22259932	0.04291917	20571.7759	1.37242164	308358563
cg23893802	0.0429271	1.6978E+13	2.63767121	1.09E+26
cg00744739	0.04293265	0.24057466	0.06055973	0.9556874
cg00779858	0.04294343	11.2751359	1.0799861	117.713264
cg08144943	0.0429436	11.5926611	1.08093711	124.327115
cg10457397	0.04294479	1261766.4	1.56222812	1.0191E+12
cg07929082	0.04294488	0.09155814	0.00904412	0.92688906
cg06938101	0.04294565	165.279246	1.17607081	23227.5378
cg22256309	0.04294644	0.15498119	0.02548388	0.94252415
cg16430951	0.04295273	98727751	1.79299291	5.4363E+15
cg01137708	0.04296327	5642711.37	1.63614541	1.946E+13
cg20373432	0.04297291	5.47129718	1.0552187	28.3686147
cg07375367	0.04297716	1045.07276	1.24572581	876739.529
cg16271663	0.04299004	5.09440106	1.05270305	24.6536021
cg27291385	0.04299906	4.90125407	1.0513502	22.8489913
cg19053889	0.04299977	26.7757463	1.10911043	646.410462
cg11159295	0.04300145	243.514912	1.188936	49876.1182
cg24028120	0.04300475	1219580803	1.93201716	7.6986E+17
cg07213036	0.04300586	3.41154411	1.03937725	11.1976986
cg18235937	0.04301029	8.69317984	1.07038198	70.6022498
cg07261734	0.04301129	7.45735436	1.06522234	52.2070667
cg22419039	0.04301463	4.57185396	1.04893295	19.9267729
cg25196508	0.0430158	8.0339668	1.06767343	60.4535252
cg20273422	0.04301847	0.20983478	0.0462442	0.95213304
cg06866553	0.04302193	28.5149626	1.1109279	731.913469
cg03910004	0.04302415	7.89E-05	8.37E-09	0.74339676
cg08701937	0.04303352	452.194983	1.21123375	168819.853
cg00508024	0.04304277	0.07226565	0.00566996	0.92105098
cg14312538	0.04304554	3.71791202	1.04194184	13.2664505
cg10467371	0.04305069	5.96311975	1.05741239	33.6281259

cg14957359	0.04305717	7.09146974	1.06309432	47.3043099
cg13077519	0.04305973	0.13344219	0.01896254	0.93905254
cg15149938	0.04306356	0.22902443	0.05492095	0.95504889
cg10215884	0.04306423	1540.46188	1.25735572	1887312.22
cg11923320	0.04306669	5.85881028	1.05669031	32.484123
cg12891252	0.04306999	0.00325513	1.27E-05	0.83648076
cg22762180	0.04307812	19302.9601	1.35968898	274036399
cg02529547	0.04309081	18.2377068	1.09442992	303.915254
cg00484488	0.04309548	8.39814747	1.06832006	66.0184935
cg07339812	0.04309586	29252.1733	1.37623597	621760847
cg12011436	0.0430994	0.01475429	0.00024812	0.87733878
cg16208084	0.04311035	3.00733428	1.03470696	8.7406965
cg00541638	0.04312366	7.5077618	1.06432737	52.9597274
cg06739664	0.04313565	31510.0996	1.37678039	721165394
cg07724466	0.04313817	57.5820318	1.13323095	2925.87348
cg18380070	0.04313952	844.232065	1.23107837	578945.901
cg03805618	0.04313972	7.16047701	1.06261376	48.2512392
cg06214007	0.04314057	4.71238729	1.04897999	21.1697021
cg13213923	0.04314262	13.7463528	1.08417243	174.291662
cg15143070	0.04314566	129.176414	1.16164363	14364.5998
cg20152304	0.04314717	5.2947743	1.05270346	26.6310844
cg09941876	0.04314987	1532.89693	1.25350089	1874568.28
cg22867063	0.04315216	6.07249163	1.05711455	34.8828371
cg17829984	0.04315528	7741.57257	1.31732102	45495323.3
cg24550302	0.04315544	6091365.25	1.61739891	2.2941E+13
cg00334274	0.04316249	5.01737073	1.05083867	23.9561122
cg13217116	0.04316289	1569741.78	1.55052288	1.5892E+12
cg17366332	0.04316536	160451103	1.787157	1.4405E+16
cg12590430	0.04316539	0.29260357	0.08891217	0.96293736
cg11967894	0.04316688	11.1600741	1.07693382	115.649868

cg01940508	0.04317746	2018.44616	1.26293998	3225905.38
cg00853296	0.04318397	51114.4112	1.39410069	1874099229
cg12284802	0.04318643	8.14624445	1.06636539	62.2312947
cg25611476	0.04318863	86.1687884	1.14621806	6477.87746
cg13243329	0.04319131	169596618	1.78612458	1.6104E+16
cg18169128	0.04319966	55.53097	1.13066666	2727.31896
cg05194726	0.04320187	3.99686686	1.04325343	15.3126213
cg23806715	0.04320225	2553607173	1.93858947	3.3637E+18
cg24628676	0.04323038	5.8824065	1.05540096	32.786313
cg06912182	0.04323131	7.6546585	1.06388324	55.0754019
cg04287755	0.04324337	0.17453466	0.03212086	0.94836653
cg06933384	0.04324632	7.94772523	1.06494636	59.3141012
cg18501409	0.04324916	139.804148	1.16171905	16824.3775
cg08382220	0.04325449	10.9881285	1.07537214	112.276452
cg15342134	0.04325667	4.99289625	1.04994282	23.7432101
cg14473522	0.04327498	3.85910233	1.04165867	14.2970737
cg01518065	0.04327608	149521.26	1.43340542	1.5597E+10
cg16271954	0.04327747	4.74723455	1.04818104	21.5003278
cg27233566	0.04327855	88.8925557	1.145166	6900.2105
cg24827036	0.04328252	128931.023	1.42650081	1.1653E+10
cg17343873	0.04328294	0.12204758	0.01587203	0.93848171
cg27662875	0.04329331	4329.43589	1.28704935	14563555.9
cg04408225	0.04333063	36762355	1.68538289	8.0188E+14
cg02965250	0.04333159	38.6072336	1.11567518	1335.97889
cg07718808	0.04333498	1282504.19	1.52372248	1.0795E+12
cg05833318	0.04333732	420.903699	1.19826476	147847.062
cg09597022	0.04334315	0.22209672	0.05159734	0.9559979
cg05090725	0.0433443	11.508168	1.07578637	123.10802
cg07460992	0.04334998	4.33020396	1.04475879	17.9473641
cg25587223	0.04336247	4.0522223	1.04260548	15.7494909

cg20275889	0.04336289	7.60531989	1.06235868	54.4457271
cg02207686	0.04336522	7.37386194	1.06135724	51.2304791
cg10698419	0.04336602	260.445371	1.18029694	57470.1069
cg01535980	0.04336665	7.0833833	1.06007292	47.331007
cg03631076	0.04338035	2232233665	1.89663371	2.6272E+18
cg07647825	0.04338431	19.5986062	1.09244719	351.600855
cg13910573	0.04339282	2613611.83	1.55040523	4.4059E+12
cg03474795	0.04340597	147331.046	1.42254276	1.5259E+10
cg25402787	0.0434061	8.4882333	1.06538729	67.6280875
cg16012111	0.04340671	31.0056052	1.10704624	868.3897
cg00549566	0.04341361	3.11381335	1.03417018	9.375472
cg26104690	0.04342292	9.84353068	1.06988093	90.5662427
cg23445859	0.04342457	3.45548586	1.0372949	11.511078
cg15767406	0.0434259	23.8390979	1.09815349	517.507431
cg20293685	0.04343055	0.00789106	7.18E-05	0.86688269
cg00308618	0.04343982	522.766469	1.20248609	227266.481
cg17460200	0.04344214	55374853.1	1.69056224	1.8138E+15
cg23127998	0.04346012	9.68525582	1.06895089	87.7534984
cg23501962	0.04346718	10.9298194	1.07266855	111.368
cg26291655	0.04346742	8.70979619	1.06554589	71.194071
cg20863668	0.04346863	3491.80331	1.27029406	9598321.14
cg07207726	0.04347613	35.5250348	1.11024165	1136.71478
cg16818414	0.04347848	5.55842897	1.05150829	29.382681
cg04822759	0.04348116	2126.01286	1.25138813	3611933.46
cg12651664	0.0434895	1824941.94	1.52410254	2.1852E+12
cg15358657	0.04348957	58814.0235	1.37850642	2509302328
cg23261237	0.04349007	39686.8084	1.36271401	1155813140
cg07820696	0.0434988	2441551.88	1.53615348	3.8806E+12
cg09911554	0.04349999	3362104.95	1.55043721	7.2907E+12
cg23254528	0.0435068	18.4763312	1.08873312	313.552336

cg01101663	0.04351482	9.8317744	1.06880401	90.4410788
cg12395733	0.04352512	0.12860279	0.01755458	0.94212916
cg05493761	0.04352799	3.33160141	1.03558067	10.718207
cg14097855	0.04354949	686.179716	1.20815116	389721.601
cg07317601	0.04355155	83413.1264	1.3881421	5012274802
cg02351804	0.04356922	5.08860775	1.04807695	24.7061332
cg04670666	0.04357336	5.24935881	1.04898493	26.2689835
cg01916762	0.04357749	7.8047E+12	2.35284955	2.59E+25
cg20244273	0.04359052	10.860164	1.07100959	110.123348
cg17798749	0.04359188	79.6658115	1.13415796	5595.90615
cg02528389	0.04361109	30028566	1.63818404	5.5044E+14
cg02499214	0.04361364	0.2234189	0.05210643	0.95796255
cg21739584	0.04362456	20.2801597	1.08990635	377.357999
cg06650260	0.04363113	3.66520111	1.03781294	12.9442394
cg02864688	0.04363571	9.46627159	1.06628577	84.039664
cg14325123	0.04363766	57.0064288	1.12233496	2895.51074
cg01084740	0.04363825	23.253338	1.09396226	494.274573
cg13312337	0.04364717	39.318171	1.11031785	1392.32074
cg15993762	0.04366792	10.9263669	1.07028131	111.545902
cg08108311	0.04367512	3.70011049	1.03781821	13.1919228
cg19770715	0.04368055	4.09844133	1.04079629	16.1388175
cg03221702	0.04368254	45.9820916	1.1145863	1896.98433
cg13235059	0.04368386	6.77646309	1.05570732	43.4973322
cg05778739	0.04368942	160063.61	1.4038205	1.825E+10
cg13663300	0.04369771	4.66262116	1.0444809	20.8142016
cg00858015	0.04369843	2819.71566	1.25174904	6351749.57
cg08145617	0.04370028	4.70323272	1.04471779	21.1735631
cg24072474	0.04372207	7.04995493	1.05652734	47.0426678
cg00534253	0.04372463	47509325.8	1.64455981	1.3725E+15
cg14392392	0.04372587	519238491	1.7588499	1.5329E+17

cg08376992	0.04373088	72.1526292	1.12782994	4615.94583
cg00549412	0.04373164	12.1888829	1.07282142	138.484247
cg08417719	0.04373406	0.25693113	0.06858315	0.96253386
cg02417264	0.04373483	0.04143882	0.00187785	0.91443832
cg07354515	0.04373767	8.94073004	1.06345098	75.1672201
cg09736654	0.04374477	120601.046	1.38846001	1.0475E+10
cg09124213	0.04376072	1.7972E+10	1.93592953	1.67E+20
cg15676542	0.04376684	10.2314073	1.06715168	98.0944864
cg08790487	0.04377445	80.3402594	1.13024639	5710.75238
cg08441931	0.04378603	29.6259178	1.09900761	798.625043
cg24577417	0.04379239	0.19053662	0.03801855	0.9549076
cg06241803	0.04379767	3871487465	1.84757905	8.1125E+18
cg08755490	0.04380989	10.2193842	1.06662427	97.9124675
cg16057262	0.04381016	4.42883574	1.04215829	18.8211198
cg00662684	0.04382766	8.92341521	1.06242749	74.9484929
cg16399365	0.04382909	5.91536752	1.0503985	33.3126646
cg16291917	0.04382984	3.5113712	1.035349	11.9087647
cg00206407	0.04383363	11.2305771	1.06913755	117.969724
cg16555417	0.0438346	6.27881601	1.05208332	37.4718709
cg15458338	0.04383741	0.14705584	0.02280138	0.94842576
cg12806353	0.04385246	9.28227783	1.0633162	81.0301601
cg24378095	0.04385725	95122566.4	1.65826772	5.4565E+15
cg06150940	0.0438761	2146839.66	1.49201927	3.089E+12
cg18689240	0.04387622	4.66997551	1.04320286	20.9054942
cg15744134	0.04388477	5.125153	1.0458017	25.116801
cg27602828	0.04389874	9.2933559	1.06284569	81.259645
cg10579631	0.04390162	0.20798038	0.04515244	0.95799562
cg01067724	0.04390723	1.1647E+14	2.42117144	5.60E+27
cg09444979	0.04390824	56.0879678	1.11619023	2818.39067
cg22717014	0.04392009	4.30981764	1.04060027	17.8498206

cg04437233	0.04392026	38869.2823	1.33360297	1132886730
cg25376924	0.04392988	5.7199E+11	2.08816645	1.57E+23
cg27205688	0.04393376	1865.61152	1.22715066	2836250.23
cg27576271	0.04393496	7.43448042	1.05602681	52.3391061
cg12409211	0.04393663	185.363215	1.15242524	29814.9676
cg18633561	0.04394391	4.43619443	1.04124968	18.9001941
cg21292033	0.04394446	4.2511987	1.04004318	17.3768655
cg15207055	0.04394683	6.05359593	1.05004354	34.8995277
cg13116174	0.04394825	192406007	1.67727637	2.2072E+16
cg04149335	0.04395483	7.3468E+11	2.09583923	2.58E+23
cg21660130	0.04395788	5.397613	1.04669273	27.8345546
cg06374807	0.0439587	0.11792914	0.01473561	0.94378738
cg13411897	0.04396034	2415.32307	1.23461635	4725180.81
cg14041079	0.04396395	2.3235E+10	1.90678069	2.83E+20
cg13051028	0.04396694	2677.86979	1.23777104	5793467.76
cg09318120	0.04396863	998.602497	1.20514429	827458.551
cg26544435	0.04397364	14597.6924	1.295438	164494652
cg14503783	0.04398339	262916556	1.68623673	4.0994E+16
cg12082871	0.04399003	87.3462307	1.12786797	6764.41237
cg24855075	0.0439923	32.4478418	1.0981592	958.752099
cg03812837	0.04399573	6.85012261	1.05311314	44.5575865
cg26923490	0.04399987	3.45240614	1.03386029	11.5287416
cg01287088	0.04400305	6.4766914	1.05146052	39.8945377
cg04422150	0.04400483	0.02912858	0.00093298	0.90941929
cg10087374	0.04401844	568.778947	1.18523034	272950.733
cg17567560	0.0440299	2.97475519	1.02957599	8.59496384
cg03003689	0.04403745	5085.92213	1.25593307	20595527.3
cg11439192	0.04404289	2136313.2	1.47521272	3.0937E+12
cg25897951	0.04404792	14.0270678	1.07292845	183.384671
cg11358405	0.04405016	4.84405136	1.04293246	22.4989003

cg11083280	0.04405325	6.63037531	1.05166346	41.8022288
cg04897804	0.04405823	8.6232239	1.05899651	70.2174083
cg10045881	0.04405992	6.05062352	1.04904612	34.8984132
cg11444668	0.04406579	352.07485	1.16860452	106072.412
cg21188037	0.04407361	3.93701139	1.03703399	14.9465291
cg07890104	0.04408035	5.48576662	1.04614773	28.7661432
cg12072376	0.04408381	0.01899469	0.00040074	0.90033392
cg04013650	0.04408484	5.88364979	1.04805267	33.0301479
cg27295033	0.04409926	126497442	1.63695248	9.7752E+15
cg07776900	0.04410928	156.204727	1.14248541	21356.8737
cg20811145	0.04411374	23.6347017	1.08691154	513.932463
cg15246139	0.04412149	9463.59058	1.27242082	70385163
cg07951810	0.04412818	439641.663	1.40710296	1.3736E+11
cg16794070	0.04414269	4.68884754	1.0413431	21.1124375
cg09307279	0.04414404	1.2665E+10	1.83994551	8.7181E+19
cg12990093	0.04415212	303.676303	1.16138354	79404.6872
cg02905245	0.04415413	6.75503019	1.05125324	43.4057476
cg25635500	0.04415989	8.41455978	1.05725348	66.9705208
cg15704219	0.04416662	5.85629813	1.04722722	32.7495572
cg19245228	0.04417113	2287.84276	1.22359436	4277744.89
cg24643012	0.04417138	29.8343573	1.09262038	814.636895
cg14081218	0.04417433	5.95062548	1.04759803	33.8010788
cg14859317	0.04417438	1.49E-07	3.37E-14	0.66381479
cg05085336	0.04417626	0.20738258	0.04480762	0.95982642
cg07805603	0.04417788	5.29753174	1.04439906	26.8708042
cg19539664	0.04418428	0.2090774	0.0455307	0.96008541
cg07508400	0.04418593	3832.87884	1.23948112	11852508.2
cg17349352	0.04418728	7.36078873	1.05329781	51.4395932
cg18051889	0.04418819	65986.1822	1.33458888	3262559954
cg20844262	0.04419624	1997.19013	1.21819668	3274322.19

cg20996682	0.04419726	4.92360423	1.04226108	23.2589312
cg02829654	0.04419971	6.00061837	1.04760764	34.3710943
cg14366571	0.04420012	7.42836394	1.05342358	52.3821488
cg04646987	0.04420191	0.291301	0.0876157	0.96850532
cg14640066	0.04421495	4.67786426	1.04074583	21.0257042
cg23830855	0.04421682	12962902.8	1.52776501	1.0999E+14
cg14419393	0.04422384	3.78081528	1.03496968	13.8115777
cg02149708	0.04422578	6.81341168	1.05082599	44.1772272
cg09668344	0.04423012	4.85859215	1.04165282	22.6619822
cg23354735	0.04423032	23.7413338	1.08519776	519.399276
cg09663494	0.04423245	371.937182	1.16501618	118742.787
cg12293132	0.04423449	9.95234547	1.06106615	93.3487323
cg22104487	0.0442361	271.870287	1.15552352	63965.3382
cg08425065	0.04424561	7385679.41	1.50254092	3.6304E+13
cg08358115	0.04425195	11189492.1	1.51797992	8.2481E+13
cg22628500	0.04425209	5.0961691	1.04276611	24.9058146
cg03039008	0.04425311	749827.615	1.41595086	3.9708E+11
cg23027583	0.04425446	2.91860373	1.02791452	8.28692225
cg10923085	0.04425783	571220928	1.67861826	1.9438E+17
cg17583733	0.04426392	1.0319E+12	2.03366763	5.24E+23
cg02128572	0.04427622	0.05377057	0.00311597	0.92788969
cg18356785	0.04427836	7.84056847	1.05412095	58.3182734
cg10822582	0.0442927	419.252041	1.16667133	150661.346
cg24857620	0.04429365	2.75107018	1.02616692	7.37539572
cg07203561	0.04429482	5.13045508	1.04261145	25.2458088
cg08937573	0.04430137	14.3739605	1.07029975	193.040071
cg10913456	0.0443097	18.2438402	1.07670396	309.12648
cg06306636	0.04431547	6815.09565	1.25159295	37109132.7
cg00731062	0.04431992	0.18211776	0.03463335	0.95765728
cg17696847	0.04432034	13.0683864	1.06746634	159.98886

cg14300636	0.04432147	11.1363527	1.06312384	116.654661
cg12124386	0.044322	3.7482007	1.03412207	13.5854449
cg22710329	0.04432439	535.140526	1.17289093	244161.989
cg09662887	0.04432649	1153.15382	1.19589045	1111944.45
cg01763719	0.04432729	0.20007044	0.04169605	0.95999948
cg04839259	0.04432764	0.00027684	9.43E-08	0.81235343
cg05098432	0.04432943	7.92962003	1.05391406	59.6622405
cg17943647	0.04432986	5.84975077	1.04581106	32.7206179
cg15324453	0.04433764	4202.01105	1.23525075	14294180.2
cg09865015	0.04434083	9.93182513	1.05982143	93.0733685
cg04618532	0.04434122	7.529E+11	1.99780838	2.84E+23
cg23887396	0.04434277	4.22810074	1.03714828	17.2365285
cg14959291	0.04434944	31.0809345	1.09071916	885.676647
cg11437140	0.04434962	15.1703465	1.07112682	214.857296
cg26127778	0.04435079	4.48778332	1.03865609	19.3906331
cg27139424	0.04435567	5.71382214	1.04497207	31.2427138
cg19210712	0.04435928	28.7002682	1.08836019	756.831611
cg10481400	0.04436389	17022693.8	1.52137859	1.9047E+14
cg14074924	0.04437037	6178292.03	1.48232145	2.5751E+13
cg04678743	0.04437106	10.8953572	1.06195616	111.783154
cg04031129	0.04437676	1.0312E+13	2.1242044	5.01E+25
cg07818096	0.04437718	1.3418E+12	2.01792074	8.92E+23
cg18669588	0.04438004	14.5461066	1.06959055	197.822632
cg25306939	0.04438403	11.0346958	1.06214415	114.640287
cg00983637	0.04438792	19.5674417	1.07747458	355.353879
cg23733260	0.04439188	6.11710649	1.0464577	35.7577682
cg24041078	0.04439192	4.03121285	1.03557235	15.6924594
cg25756617	0.04439532	12.5953811	1.06553827	148.885901
cg09987651	0.04439729	0.24863088	0.06401044	0.96573793
cg11843238	0.04440262	4.29778706	1.0371621	17.8091484

cg20561908	0.04441573	678418.691	1.39823372	3.2917E+11
cg05877850	0.04442268	6.67E-08	6.72E-15	0.6623597
cg23818931	0.04442801	45000870.7	1.55105473	1.3056E+15
cg02831290	0.04443119	126.200439	1.12798382	14119.4852
cg21153898	0.04443128	3.51198549	1.03176431	11.9543212
cg23880581	0.04443474	4.57205726	1.03853654	20.1280426
cg05213414	0.04444298	42.4346451	1.0975688	1640.62526
cg14482308	0.04444452	3.22962089	1.02954061	10.1311701
cg00226923	0.04445529	12.5013389	1.06459872	146.800358
cg02701019	0.0444564	8.08800981	1.05316035	62.1139059
cg14267284	0.04445911	447247270	1.63770579	1.2214E+17
cg15205428	0.04445958	4.11012937	1.03562173	16.3120983
cg03103919	0.04446123	4.21975831	1.03628578	17.1828665
cg15518400	0.04446483	195.751795	1.13945028	33629.1683
cg23527902	0.04448178	7.79552696	1.05194837	57.7692235
cg06969125	0.04448547	346857.461	1.36941764	8.7855E+10
cg16126958	0.04449199	10.7062588	1.0600942	108.126218
cg20984085	0.04449483	3.56914359	1.03179671	12.3462168
cg15364537	0.04450232	52.206151	1.10204747	2473.10782
cg19006302	0.04450313	6.03794718	1.04515734	34.8816439
cg17829936	0.0445105	4.98225669	1.04017875	23.8640539
cg06796713	0.04451411	4.81193552	1.03926453	22.2799131
cg02376178	0.04452602	179.382988	1.13533862	28342.4311
cg22331740	0.04452674	10.595507	1.05942479	105.967662
cg06483978	0.04453566	452.088976	1.16098635	176043.795
cg14482569	0.04454688	6.49376048	1.04663511	40.2899965
cg13813391	0.04455134	51.5600542	1.10073562	2415.14778
cg20712415	0.04455722	5.99603255	1.04451503	34.4201907
cg10659652	0.04457117	52258673.2	1.53880437	1.7747E+15
cg18229710	0.04457352	0.0007378	6.48E-07	0.83960298

cg20410173	0.04458451	7639.90177	1.24147162	47015250.3
cg05192898	0.04458592	5.85418889	1.04366481	32.8376768
cg12320968	0.04458845	256373.2	1.35129441	4.864E+10
cg19539556	0.04459408	0.22321661	0.051663	0.96443595
cg08149078	0.04459624	1.2675E+11	1.8535253	8.67E+21
cg09173587	0.0445968	0.00044346	2.37E-07	0.82998793
cg10214045	0.04459752	1.8342E+13	2.08963531	1.61E+26
cg02144933	0.04461088	5.44452257	1.04163365	28.4580151
cg15731317	0.04461096	0.12671259	0.01687465	0.95149098
cg10667484	0.04461253	302.37296	1.14733296	79688.6432
cg14396066	0.04461583	256.846658	1.14274085	57729.8043
cg05819371	0.0446189	37.6582781	1.0911239	1299.71117
cg27386912	0.04462112	166.489256	1.13074846	24513.562
cg13760347	0.04462346	7108089.74	1.46060024	3.4592E+13
cg22315048	0.04462538	15.242146	1.06757434	217.617646
cg01716465	0.04462698	0.16897016	0.02979547	0.95823012
cg05161019	0.04463164	0.13868207	0.0201656	0.9537387
cg10316593	0.04463792	1488.23923	1.1911753	1859387.11
cg26013028	0.04464853	22.8642175	1.07765959	485.099792
cg10685559	0.04465427	9.95E-05	1.23E-08	0.80252095
cg07973470	0.0446572	8.96286252	1.05371737	76.2376202
cg24780185	0.04466263	107.75483	1.11799739	10385.6266
cg06476131	0.04466285	3.0961813	1.02730122	9.33157526
cg24272907	0.04466591	0.22425197	0.05211194	0.96501772
cg20693334	0.04467232	7.05857402	1.04758823	47.5601631
cg01610915	0.04467246	10.2005673	1.05680313	98.4588052
cg03721058	0.04467363	9.57871513	1.05521017	86.9511937
cg10597997	0.04467963	87.8503863	1.11218198	6939.23342
cg11354857	0.04468039	0.24030345	0.05973496	0.96669928
cg17176108	0.04468224	3411.16355	1.21307	9592221.97

cg11703722	0.04469332	861.139109	1.17365993	631835.975
cg11179625	0.04469481	0.03121451	0.00105773	0.92116256
cg19638477	0.04469859	3.87733377	1.03259554	14.5591536
cg02397499	0.04470575	1170235011	1.63811391	8.3599E+17
cg09618904	0.0447157	7.03409563	1.04709644	47.2530508
cg26147845	0.04471596	4.43982919	1.03578922	19.0309794
cg07574241	0.04471772	733.96812	1.16836427	461079.834
cg01427290	0.04471807	61733931.9	1.526523	2.4966E+15
cg14882265	0.04472226	7.45000054	1.0484532	52.9375159
cg08182191	0.0447313	5828.30428	1.22621119	27702512.5
cg14945297	0.04473404	232.883586	1.13669845	47712.5349
cg12850695	0.0447419	2.23E-05	6.42E-10	0.77775049
cg23753748	0.04474398	10.2988948	1.05623862	100.419766
cg19597031	0.04475908	17.1702367	1.06877197	275.846519
cg00615592	0.04476921	28.8996922	1.08170043	772.110456
cg02524475	0.0447743	4.8703801	1.03761519	22.8606929
cg25479916	0.04477507	3.4583613	1.02935788	11.6191493
cg07519822	0.04477935	591544.867	1.36297904	2.5674E+11
cg13848905	0.04477947	8.30923183	1.05057136	65.7197944
cg09802426	0.04478648	1103.10615	1.17704997	1033807.56
cg00889769	0.04478834	5.12727105	1.03875099	25.308191
cg16768376	0.0448136	4.97772662	1.03784386	23.8742679
cg21149967	0.04481589	1086.26859	1.17552428	1003789.94
cg13235717	0.04484195	8.88906867	1.0515681	75.1406794
cg03476195	0.04484219	3.94831807	1.03210868	15.1042384
cg05807768	0.04484543	20640658.2	1.47308562	2.8921E+14
cg12963312	0.04485374	8913529303	1.69222968	4.695E+19
cg13582435	0.04485582	6.33614581	1.0432846	38.4811045
cg07315372	0.04486795	1.1853E+10	1.70077911	8.2609E+19
cg03112433	0.04486868	3.9938111	1.032208	15.4528226

cg02271139	0.0448705	7.05915674	1.04573817	47.6521706
cg09144073	0.04487394	3689089.71	1.41310649	9.6308E+12
cg01755562	0.04487575	4.6313898	1.03566251	20.7111595
cg21857346	0.04488376	3.56847066	1.02946035	12.3695709
cg14427668	0.04488891	6.05779572	1.04192589	35.2202487
cg08203624	0.04490729	0.24130581	0.0601397	0.96822052
cg08370757	0.0449094	11.2691682	1.05653733	120.198453
cg04095257	0.04491367	61.2363526	1.09784765	3415.67509
cg26477856	0.04491792	6.14800436	1.04202658	36.2735061
cg13495347	0.04491862	3.3429E+10	1.73190545	6.45E+20
cg11145776	0.04492002	7.08067384	1.04534843	47.9609863
cg18592365	0.04492106	8.12753479	1.04860911	62.9947056
cg14749465	0.04492639	3.56110667	1.02915753	12.3221959
cg07589968	0.04493126	7.54259484	1.04673796	54.3505053
cg11692852	0.04493403	0.00422004	2.02E-05	0.88378357
cg24867524	0.04495357	4.91849235	1.03650099	23.3396467
cg12647497	0.04495628	7.00604726	1.04476109	46.9817442
cg05355225	0.04496889	3.46338985	1.02826209	11.6653811
cg04614997	0.04497018	4.29040358	1.03320503	17.8159826
cg19793804	0.04497144	468436.748	1.34015878	1.6374E+11
cg09662051	0.04497213	7293.31663	1.22069868	43575428.1
cg17465063	0.04497281	1164403371	1.59675332	8.4912E+17
cg17230773	0.044976	12.785752	1.05875076	154.404096
cg24840365	0.04497686	7.5809E+10	1.7526428	3.28E+21
cg05763263	0.0449819	2.23E-08	7.41E-16	0.67422764
cg16571983	0.04498889	5.05283103	1.03685921	24.6234987
cg03750606	0.04498925	7.22975493	1.04518907	50.0094745
cg12966875	0.0449904	21.235074	1.07063779	421.177331
cg25930229	0.04499326	6.31171962	1.04198765	38.2325111
cg14633020	0.04499871	272.740679	1.13322291	65642.4057

cg02267756	0.04500229	0.01249469	0.00017213	0.90696285
cg15883716	0.0450048	3.44974281	1.02796255	11.5770029
cg11659501	0.04500491	5.60088517	1.03911692	30.1890135
cg11831182	0.04500569	8196.61759	1.22221218	54969620.7
cg22837289	0.04502475	134222.035	1.29938527	1.3865E+10
cg09264282	0.04503352	14.2735108	1.06062523	192.087748
cg12733907	0.04503547	338238.112	1.32547288	8.6313E+10
cg07357279	0.04503704	3.70866795	1.02942257	13.3611
cg03485669	0.04503814	7.37923571	1.0452014	52.0982076
cg13007481	0.04504369	0.12530787	0.01643941	0.95514769
cg03077077	0.04504498	5.61548254	1.03884971	30.3543852
cg11912330	0.04504657	4.32793544	1.03287978	18.1347582
cg02992632	0.04506351	3.75743637	1.02955574	13.7130294
cg25037394	0.04506597	4.19544854	1.03204008	17.0553342
cg22438525	0.04506713	0.00306871	1.07E-05	0.88053104
cg08867933	0.04507199	0.25379924	0.06638365	0.97033003
cg19979107	0.04507201	3014.51664	1.19239889	7621032.36
cg10748355	0.04507628	4.62519464	1.0341815	20.6853686
cg13204435	0.04507832	2379.33316	1.18595258	4773568.85
cg09721423	0.04507923	66.9802771	1.09659733	4091.16217
cg11784697	0.04508204	2.7987E+10	1.69430618	4.62E+20
cg25203092	0.04508212	702421.215	1.34323547	3.6732E+11
cg21361134	0.04508558	25.6835677	1.07368323	614.376413
cg10297971	0.04508917	1560552.38	1.36631936	1.7824E+12
cg17588266	0.04510473	3.43025646	1.02725616	11.4544549
cg07901138	0.04510774	3.86445882	1.0299116	14.5003143
cg11698816	0.04511565	363000.886	1.32135366	9.9723E+10
cg09327035	0.04511806	57.0828699	1.09197708	2983.99489
cg08413402	0.04512472	80313.4645	1.27807682	5046842643
cg01596892	0.04512494	1934.97138	1.1786913	3176501.12

cg12377709	0.04513202	701198500	1.55554479	3.1608E+17
cg00489699	0.04513804	72252.7901	1.27427831	4096801796
cg15085431	0.04513971	24.2846141	1.07152405	550.377275
cg25385940	0.04514276	3.97407091	1.03031315	15.328582
cg22624022	0.04514697	0.03316834	0.00118423	0.92899294
cg06719628	0.04514998	10241.6992	1.22085636	85917071.2
cg01626459	0.04515316	4.03629638	1.03059153	15.8080948
cg24933925	0.04515566	3685497.91	1.38590314	9.8008E+12
cg10418044	0.04516624	0.01089413	0.00013081	0.90725387
cg22660542	0.04517735	6.50268122	1.0410455	40.6176897
cg07482220	0.04517801	23.0741547	1.06975266	497.700671
cg18094551	0.04517996	5752634410	1.62024059	2.0425E+19
cg19328711	0.04518098	749.366373	1.15270136	487159.971
cg06253072	0.04518413	10.4275645	1.05158588	103.400115
cg15978039	0.04519084	5.64189162	1.03776402	30.6726196
cg04822405	0.04520087	30.5066916	1.07580624	865.079782
cg03624316	0.04521709	0.1929207	0.03854628	0.96555102
cg03207661	0.04521935	83832.8229	1.27303715	5520610460
cg07533422	0.04522258	4.21624364	1.03109388	17.2406323
cg18182216	0.04522843	4.36438819	1.03181141	18.4606258
cg18167715	0.04522929	252331748	1.50846341	4.2209E+16
cg12114392	0.04523308	1225528.7	1.34668698	1.1153E+12
cg27201802	0.04523538	6.46973254	1.04041878	40.2313375
cg07185695	0.04523844	6.10316068	1.03910563	35.8467602
cg01563704	0.04523951	3.2629237	1.02539181	10.3830273
cg14947478	0.04524039	3.68291732	1.02802225	13.1941501
cg22247664	0.04524113	7.55602775	1.04379612	54.6979953
cg20417500	0.04524187	8.67962279	1.04686009	71.9636299
cg26727816	0.04524285	10.1500359	1.05032693	98.0868189
cg02099543	0.04524437	5.10157863	1.03511729	25.1431453

cg20740157	0.04524497	3.05138138	1.02390716	9.09352794
cg12982543	0.04524957	5.60866129	1.03715472	30.3301725
cg23528982	0.04525234	747985.893	1.33106561	4.2033E+11
cg22376415	0.04525376	11467947.7	1.41001068	9.3272E+13
cg24296478	0.04525425	9.92245778	1.04969752	93.7938473
cg15746415	0.04525761	7657.95321	1.20790798	48550260.7
cg26912602	0.04526266	20.8902363	1.06622126	409.297759
cg21082050	0.04526563	12.1595414	1.05408161	140.2685
cg02132760	0.04526634	120.044245	1.10620056	13027.1321
cg11055926	0.04526781	10273.0313	1.2149049	86867022.2
cg24817528	0.04527343	0.07707444	0.00626974	0.94748264
cg07086918	0.04527818	5.38736092	1.03604474	28.0139037
cg02494794	0.04528131	143.353128	1.10997097	18514.1053
cg05642379	0.045282	32.298349	1.07573928	969.736228
cg25836955	0.04528324	239.214142	1.12192439	51004.6901
cg02369140	0.04528573	262.457998	1.12404081	61282.6514
cg13597422	0.0452886	16.0107027	1.05990718	241.853822
cg11701377	0.04529006	4790735.91	1.38072361	1.6623E+13
cg25566552	0.04529044	5.03E-07	3.42E-13	0.73774799
cg24109980	0.0452921	3.79181957	1.02833515	13.9817215
cg10638439	0.04531114	1096530.98	1.33689434	8.9938E+11
cg16004427	0.04531866	6.26551344	1.03898861	37.7835314
cg20094837	0.04532145	8.25089944	1.04493869	65.1496034
cg07949433	0.04532524	5122199.78	1.37925015	1.9023E+13
cg12214534	0.04532811	1393286.59	1.3421306	1.4464E+12
cg15884014	0.04532931	0.29785998	0.09098335	0.97512976
cg15860013	0.04533342	306509.628	1.30012347	7.2261E+10
cg02707152	0.04533518	12.7065059	1.0542123	153.152541
cg00065570	0.04533651	55.5014878	1.08696276	2833.96567
cg07061500	0.04534011	3518.43424	1.1845954	10450301.9

cg20417723	0.04534225	0.2276496	0.05343935	0.96977857
cg25174840	0.04534404	5084733.1	1.37722058	1.8773E+13
cg17553286	0.04534557	17.2857136	1.06082923	281.662579
cg00494665	0.04534651	4.03905396	1.02934229	15.8489135
cg16385335	0.04534816	6.13102396	1.03826637	36.204057
cg12448934	0.0453557	30.6564786	1.07332934	875.611652
cg03975922	0.04536864	10.7883549	1.05025565	110.819306
cg03559133	0.04537727	1098.06614	1.15495563	1043978.85
cg11147919	0.04538082	13.6240025	1.0551681	175.908884
cg12346504	0.04538403	52.0303637	1.08458037	2496.04254
cg27522367	0.04539506	15.938823	1.05838849	240.03103
cg20299002	0.04540664	5504.15564	1.19253461	25404486.5
cg06931327	0.04540666	160.615645	1.10941189	23253.2079
cg07051728	0.04541209	72.3577577	1.09135861	4797.36452
cg05397514	0.04541366	2408860.41	1.34977065	4.299E+12
cg06403845	0.04541445	0.24686356	0.06270648	0.97185515
cg02641095	0.04541553	189148.028	1.28132964	2.7922E+10
cg13360404	0.04541943	403342165	1.49770455	1.0862E+17
cg10613925	0.04541964	2.0876E+11	1.70113769	2.56E+22
cg18404628	0.04542887	66561.7149	1.25347108	3534554522
cg27533754	0.04543654	651211.438	1.31238339	3.2313E+11
cg27313941	0.04544499	7.87766729	1.0427231	59.5149775
cg08402572	0.04547506	236.247285	1.1163016	49997.9391
cg09123773	0.04547902	1.5675E+12	1.75913661	1.40E+24
cg05576016	0.04548079	274.41691	1.11950901	67265.7745
cg07359803	0.04548692	1275453.08	1.3261506	1.2267E+12
cg06220958	0.04549387	20.7204817	1.0626492	404.026429
cg09240001	0.04550537	4.66475741	1.0312718	21.1001229
cg12665414	0.04551346	5.17778532	1.0333632	25.9438897
cg23054456	0.04553031	0.21872629	0.04930896	0.97023319

cg21525449	0.04553062	576066673	1.49334652	2.2222E+17
cg21553199	0.04553312	10.0422251	1.04690023	96.3284577
cg21656246	0.04554632	7.42435794	1.04051165	52.9749866
cg26572651	0.04555129	62.5953959	1.08529378	3610.25157
cg00534700	0.04555376	493.101999	1.13045606	215089.812
cg04274259	0.04555464	965.308078	1.14554133	813431.749
cg00636737	0.04556057	8.11100199	1.04219537	63.1247799
cg17929065	0.04556823	6.45450396	1.03743796	40.1572172
cg20733500	0.04557023	378936350	1.47571762	9.7304E+16
cg16243646	0.04557232	249.126048	1.11477763	55673.693
cg20142309	0.0455802	6.43765044	1.03727971	39.9538742
cg11908453	0.04558158	3.9925237	1.0275761	15.512472
cg16126178	0.04558853	351.179518	1.12185637	109931.233
cg15066489	0.04559825	5.78148853	1.03494159	32.2970977
cg24138433	0.0456031	6.60833215	1.03760988	42.0871607
cg00948900	0.04560805	0.16604126	0.02855358	0.96554269
cg13253791	0.04560919	71.9672309	1.0870728	4764.43006
cg26317056	0.04560934	49.1682206	1.07901451	2240.48323
cg11691214	0.04561052	0.00491949	2.68E-05	0.90146936
cg13152070	0.04561447	5570.62223	1.18316494	26227815.7
cg24789467	0.04562261	10.2963664	1.04642961	101.311316
cg13702181	0.04562398	5.75186054	1.03462639	31.9766632
cg11098493	0.04564186	4.40698692	1.02915513	18.8713375
cg07602200	0.04564524	7.71062035	1.04033829	57.1483973
cg07400063	0.04565232	15.4760251	1.05437312	227.156163
cg12385383	0.04565772	103.7769	1.09375209	9846.51371
cg03768001	0.04566825	4733855552	1.53570894	1.4592E+19
cg18977283	0.04567349	0.19008913	0.03730629	0.96857331
cg19695386	0.04567633	0.19981762	0.04118217	0.96952357
cg13229245	0.04568098	5.91968852	1.0347301	33.8665244

cg01869233	0.0456818	21.5444946	1.06070178	437.602027
cg20646018	0.0456864	15532274.3	1.37367947	1.7562E+14
cg26922546	0.04568845	8.45564248	1.04176222	68.6316788
cg01501819	0.0456932	3.28221923	1.023013	10.5306219
cg02852419	0.04569609	0.1670911	0.02889161	0.9663511
cg00473462	0.04570281	5705175.69	1.34599463	2.4182E+13
cg04988673	0.04570581	3020166759	1.51683294	6.0135E+18
cg25096282	0.04570614	6472.25872	1.18231404	35430631.4
cg06294637	0.04570791	2522483.15	1.32472414	4.8032E+12
cg11208222	0.04570854	3.95117511	1.02655411	15.2079511
cg26213155	0.04571148	0.2060012	0.04373383	0.97033574
cg22112841	0.04571964	10.0565968	1.04488902	96.7903163
cg23569090	0.04572484	0.00339182	1.28E-05	0.89759035
cg27657283	0.04573117	4682898.57	1.33828541	1.6386E+13
cg08027001	0.04573174	47.6711985	1.07605482	2111.92136
cg07796520	0.04573194	37975545.2	1.39240893	1.0357E+15
cg12910830	0.04574231	28.9151896	1.06572666	784.524045
cg01639898	0.04574744	8.64316511	1.0415999	71.7207279
cg07057320	0.04575196	9.81344336	1.04405368	92.2401527
cg23689441	0.0457532	905.795229	1.13711377	721532.9
cg17563229	0.04575752	6.49441749	1.03590047	40.7157442
cg25815054	0.04576379	329667.827	1.27019504	8.5562E+10
cg23099349	0.04576418	224935326	1.43615564	3.523E+16
cg13154413	0.04576533	3.61457941	1.02447365	12.7530701
cg06236987	0.04576776	22.8512427	1.06060876	492.339219
cg12377368	0.04577193	6.82504725	1.03674078	44.9304888
cg05198244	0.04577196	0.21570741	0.04788997	0.97159559
cg23520574	0.04578492	0.23541689	0.05694288	0.97327547
cg16277607	0.04578505	6.17503611	1.03468136	36.8529601
cg15246590	0.04579656	10.8378605	1.04550931	112.346412

cg04436646	0.04580139	10633647	1.35229675	8.3617E+13
cg03145963	0.0458143	12.2440944	1.04768346	143.094601
cg12744859	0.04581628	4.60042884	1.02877131	20.5720603
cg21065990	0.0458196	9051496.3	1.34646157	6.0848E+13
cg26836793	0.04582645	9035058.19	1.34574761	6.0659E+13
cg01615475	0.0458309	0.26592192	0.07247059	0.97576776
cg24780981	0.04583258	3.1602E+11	1.63262752	6.12E+22
cg25355076	0.04583406	1.1626E+10	1.53556251	8.8023E+19
cg08711724	0.04583991	9062252.06	1.34451077	6.1081E+13
cg00724111	0.04585392	8.830944	1.04092993	74.919137
cg26582085	0.04585586	0.09275469	0.00898835	0.95717572
cg15954031	0.04585925	3.75559115	1.0246353	13.7653513
cg22472290	0.04586116	4.96994756	1.02991429	23.9829461
cg23276878	0.04586882	17.2245789	1.05361363	281.589103
cg09991769	0.04587246	0.05434619	0.00311548	0.94801052
cg20712263	0.04587506	15.7243473	1.05177173	235.084375
cg01627785	0.04587596	368.511668	1.11431393	121869.471
cg21942576	0.04587721	78.5164359	1.08317274	5691.45667
cg09094674	0.04588266	57.4572518	1.07688941	3065.62193
cg01025690	0.04588624	7.99682797	1.03871407	61.5657948
cg24071007	0.04588781	6086.99129	1.17250013	31600391.4
cg14527280	0.04589287	10994.6588	1.1849771	102012539
cg05709162	0.04589645	8.20730781	1.03910619	64.8248484
cg00177496	0.04590454	45.9631675	1.07209706	1970.54244
cg02136620	0.04592033	7.65288323	1.03755465	56.4467829
cg12235988	0.04592034	21004.225	1.19756967	368393988
cg11908659	0.04592124	613.505198	1.12328886	335077.327
cg02921627	0.04592231	4.77845401	1.02872569	22.1960264
cg12498453	0.04592859	4.5775E+10	1.55856633	1.34E+21
cg07014308	0.04593653	0.00179493	3.61E-06	0.89218853

cg19637634	0.04594963	107.837052	1.08781631	10690.0676
cg14675357	0.04595128	5.14590424	1.02988561	25.7119142
cg06129556	0.04595293	10.1348415	1.04249207	98.5283397
cg11480267	0.04595721	0.13022502	0.01759054	0.96407261
cg19962750	0.0459622	4.90399728	1.0289139	23.373374
cg26995224	0.04597282	5.12399645	1.02964288	25.4994622
cg27009392	0.04597479	3.51061509	1.02269395	12.050935
cg07119434	0.04597809	4.65217377	1.02782856	21.0567419
cg08711320	0.04598187	0.11461894	0.01365505	0.96209849
cg05300440	0.04598875	0.11248311	0.01315441	0.96184104
cg22507887	0.04599142	0.34095554	0.11849795	0.98103537
cg11133895	0.04599345	2223.30445	1.14690358	4309937.42
cg21784917	0.04599588	4.490981	1.02705766	19.6375637
cg24404083	0.04600076	16855082.1	1.34366153	2.1143E+14
cg16911672	0.04600986	5.56200869	1.03085792	30.0098978
cg22054580	0.04601947	371.188289	1.11019321	124105.196
cg10003018	0.04602808	103344583	1.38446476	7.7142E+15
cg22733687	0.04602939	14.7529292	1.04857363	207.566654
cg06487247	0.0460303	25.6497399	1.05883002	621.354842
cg13862524	0.04603391	24474.5173	1.1946812	501390662
cg05128626	0.0460439	393185.296	1.25379331	1.233E+11
cg21332729	0.04604624	1754.14271	1.14004691	2699026.35
cg05245650	0.04604923	14.7762086	1.04834984	208.266678
cg02149136	0.04605359	980.313612	1.1282088	851805.782
cg14066905	0.04606665	0.14276113	0.02108511	0.9665939
cg24228707	0.04607111	4.79494358	1.02770645	22.3716451
cg10290107	0.04607533	19864766	1.3399828	2.9449E+14
cg17707347	0.04607951	1286287.97	1.27726649	1.2954E+12
cg27569321	0.04608163	14521128.3	1.33206585	1.583E+14
cg24341452	0.04608462	6.68472754	1.03355736	43.2347386

cg08210001	0.0460892	4398689.63	1.30400785	1.4838E+13
cg02606403	0.04608955	6.17109422	1.03208096	36.8986593
cg02102889	0.04609091	7.38447261	1.03528778	52.6717662
cg26306869	0.04609201	28.6401691	1.05989823	773.90382
cg10497692	0.0460979	4631.13401	1.1573536	18531417
cg21969477	0.04610016	7.01812207	1.03429034	47.6210938
cg24080793	0.04610052	3.196875	1.02031111	10.0165623
cg21817179	0.04610059	5.82748842	1.03096501	32.9396449
cg05512327	0.04610127	8.82404382	1.03838515	74.9854229
cg13263104	0.04610241	4616.71468	1.15709232	18420357.8
cg05136531	0.04610524	5.80458204	1.03085664	32.6846348
cg26337738	0.04610732	5.1167E+11	1.59303881	1.64E+23
cg23401756	0.04611987	24.6580012	1.05672442	575.378985
cg26085197	0.0461229	20193.5836	1.18591319	343853850
cg12985929	0.04612746	6.19024537	1.03181463	37.1376182
cg15564619	0.046131	4.90459053	1.02766991	23.4073295
cg09833538	0.04613222	61.5464298	1.07324877	3529.43616
cg22614265	0.04613497	5148311.26	1.30340693	2.0335E+13
cg15857427	0.04613828	34085.6174	1.19577365	971613073
cg03752087	0.04613845	0.22038883	0.04984604	0.97442509
cg09818930	0.04614359	165708.09	1.22825725	2.2356E+10
cg16214500	0.04614987	3831.4221	1.15133173	12750274.2
cg11948456	0.04615295	5.45106865	1.02936213	28.8665656
cg27468077	0.0461602	1177.64309	1.12799429	1229477.18
cg03082517	0.04618036	120651.604	1.21923964	1.1939E+10
cg21117673	0.04618112	7.47053627	1.03464876	53.9399595
cg12114584	0.04619339	5.73463459	1.02992522	31.9305064
cg23737055	0.04620671	57664566.2	1.35070232	2.4618E+15
cg08263387	0.04620795	10630.0954	1.16873195	96685068.5
cg00286388	0.04621414	107.320762	1.08166718	10648.1423

cg20438404	0.04621621	22.4995334	1.05363299	480.460471
cg12074252	0.04621692	27038767.7	1.33255254	5.4864E+14
cg01605945	0.04621695	223503.443	1.22954036	4.0628E+10
cg18546840	0.04621746	4.76506543	1.02653585	22.1189046
cg26867866	0.04622118	102199298	1.36212941	7.6679E+15
cg02764409	0.04622996	1186077.61	1.26341634	1.1135E+12
cg23717686	0.04623161	269774314	1.38320367	5.2616E+16
cg24645214	0.0462368	3.00876581	1.01855132	8.88779144
cg20979732	0.0462398	11720.5384	1.16907936	117503590
cg02675375	0.04624017	1.6023E+11	1.53746217	1.67E+22
cg17013303	0.04624143	0.21562368	0.04769773	0.97475444
cg20707345	0.0462481	29399.6349	1.18668681	728362803
cg24317255	0.04625297	86.2117982	1.07685667	6902.00877
cg03324464	0.04625654	8.20116438	1.03554421	64.9504837
cg00952134	0.04625743	1569812.09	1.26711111	1.9448E+12
cg06103243	0.04626731	5.11947328	1.02739486	25.5101594
cg00910297	0.04627061	88387728.6	1.35329189	5.7729E+15
cg05657581	0.04628115	1960.59199	1.13313678	3392283.27
cg12757705	0.04628237	14317.3859	1.17083566	175077978
cg13587740	0.04628335	67.8141741	1.07195189	4290.08265
cg21305021	0.04629205	19.5913389	1.05012052	365.501437
cg26344619	0.04629519	9.67528447	1.03797897	90.1859602
cg20566766	0.04630037	9.69236357	1.03795396	90.5068198
cg24842334	0.04630587	0.153214	0.02420686	0.96974719
cg13736939	0.04630749	0.14232569	0.02091347	0.96859102
cg12350309	0.04631886	6.071757	1.0298682	35.7970399
cg14170423	0.0463219	8.36253121	1.03523158	67.5519657
cg25034947	0.04632345	20905.0526	1.17599603	371617941
cg07205462	0.04633277	7486236.65	1.29341879	4.333E+13
cg27142680	0.04633762	12.3325315	1.0416239	146.013675

cg02176069	0.04634495	6.90503183	1.03179726	46.2101096
cg05020510	0.04634601	17.5296381	1.04747355	293.361311
cg07717763	0.04634812	0.01001922	0.00010815	0.92820157
cg04279588	0.04635212	8966593.87	1.2954101	6.2065E+13
cg04853708	0.04635296	12.9856299	1.04231215	161.781271
cg16734795	0.04635407	0.17746834	0.03238733	0.97244868
cg26271948	0.0463767	2206763.33	1.26433221	3.8517E+12
cg05020775	0.04637971	75.6239121	1.07186491	5335.5381
cg03161190	0.04638534	4.01305191	1.02250744	15.7500913
cg00046018	0.04638794	243808.377	1.2196268	4.8738E+10
cg10083572	0.04640324	9.30003937	1.03618003	83.4707578
cg24524044	0.0464044	30.191538	1.05579095	863.36123
cg06620654	0.0464047	74.2134449	1.07102216	5142.41029
cg00512404	0.04640611	3.5507804	1.02038409	12.3561721
cg20265830	0.04641444	155737.96	1.20918291	2.0058E+10
cg02845204	0.04641775	0.19165959	0.03770934	0.97411937
cg14222679	0.04641871	0.07601576	0.00601956	0.95993612
cg03592345	0.04642541	9.64440084	1.03654626	89.7349895
cg02643054	0.04642766	4.20281832	1.0229847	17.2668093
cg03506033	0.04643264	2.87784267	1.01684706	8.14476316
cg02616786	0.04643464	0.12698627	0.01665984	0.96792735
cg12661343	0.04643641	337.541204	1.09627066	103928.772
cg01577751	0.04644685	0.16011829	0.02638791	0.97157631
cg19917249	0.04645545	0.00119839	1.60E-06	0.89975969
cg21793358	0.04646583	7.99365151	1.03307832	61.8524881
cg25852200	0.04646611	245.707039	1.08998643	55387.7986
cg27366305	0.04647084	63.210869	1.06697039	3744.82177
cg02235894	0.0464726	1.2747E+14	1.66112658	9.78E+27
cg27621129	0.04647866	7.19966085	1.03127031	50.2633654
cg06139836	0.04648129	5.60217642	1.02722162	30.5526869

cg27333706	0.04648472	5.76494233	1.02765248	32.3402712
cg22910715	0.04649635	6276700365	1.41921234	2.776E+19
cg00040590	0.04649862	0.09702488	0.00976064	0.96446872
cg09251737	0.04650194	29.6413169	1.0539132	833.662266
cg17121120	0.04650284	265944.817	1.21346411	5.8285E+10
cg00591406	0.04650544	562.943373	1.10298854	287315.079
cg14505694	0.04650558	568.124264	1.10314058	292587.531
cg08478189	0.04650924	60772655	1.31929251	2.7995E+15
cg02147791	0.04652014	2523202.73	1.25506041	5.0727E+12
cg07537370	0.04652141	5.77787661	1.02739104	32.4938187
cg08274892	0.04652757	3313.2612	1.13275351	9691163.76
cg05800928	0.04653493	1508895.15	1.24397736	1.8302E+12
cg26755793	0.04653522	6.79331818	1.02983427	44.8122318
cg12422930	0.04653717	79486.5345	1.18890344	5314232403
cg20066782	0.04654718	10.8740178	1.03716183	114.007536
cg07474312	0.04656377	3554.3198	1.13246978	11155431.7
cg13264159	0.04656886	43414.4202	1.17613753	1602543779
cg17845761	0.04657567	13.3921403	1.04012467	172.430697
cg01143804	0.04657698	47.6475883	1.06030951	2141.16033
cg07073662	0.04658078	16781.2836	1.15867061	243047056
cg05525106	0.04658239	55.3254901	1.0626096	2880.55921
cg02809940	0.04658322	0.15473032	0.02462698	0.97216452
cg01454947	0.04658629	12029.4607	1.15257776	125551549
cg09312407	0.04658681	11.1772268	1.03715211	120.455234
cg26164735	0.04660507	3853799.19	1.25598885	1.1825E+13
cg10127573	0.04661745	1169.58507	1.11158283	1230613.85
cg16664523	0.0466201	4.28705987	1.02201826	17.9829295
cg18805066	0.04662146	3.97842759	1.02086779	15.5043447
cg11637682	0.04662673	5.63615458	1.02615785	30.9564834
cg18523666	0.04663966	0.23053213	0.05431778	0.97841

cg23733826	0.04664072	630446.064	1.21966311	3.2588E+11
cg19159961	0.04665803	6.41219615	1.02786801	40.0014973
cg08283206	0.04666461	4.77983526	1.02336339	22.325232
cg19945554	0.04667394	3.58811336	1.01898553	12.6346813
cg20646120	0.04667585	127.889568	1.07397941	15229.1017
cg01751433	0.04667731	705403.517	1.21900262	4.082E+11
cg06228542	0.04668102	5.03549396	1.02402889	24.7612149
cg08137352	0.04668578	189844005	1.32258525	2.725E+16
cg06126413	0.04669352	0.07149814	0.00531318	0.9621331
cg19119538	0.04669503	95.5354369	1.06896112	8538.21484
cg02418535	0.0466984	57.1793212	1.06090089	3081.79098
cg20717612	0.04670365	76.8614581	1.06538701	5545.10586
cg13411656	0.04670922	25.8841457	1.04852079	638.984945
cg08944563	0.04671578	103882.844	1.18278196	9123951504
cg26732808	0.0467182	0.17076824	0.02991997	0.9746597
cg08378505	0.04673137	58.7498554	1.06068142	3254.08311
cg19382808	0.04673375	0.00165753	3.01E-06	0.91162254
cg17638841	0.04673791	5.52283625	1.02497209	29.7585861
cg11327408	0.04674618	0.03253373	0.00111195	0.95188147
cg18396533	0.04674725	8.4261809	1.03114884	68.8557481
cg08462055	0.04674864	7.55862562	1.02952473	55.4943653
cg18237323	0.04674903	4.1212285	1.02057831	16.6420588
cg21298759	0.04675741	10.0297514	1.03362874	97.3230618
cg24952959	0.04675764	0.00887653	8.43E-05	0.93447447
cg02671204	0.04676404	6.10977472	1.02624964	36.3745289
cg16238618	0.04676523	25652308.6	1.27653283	5.1549E+14
cg03600270	0.04678063	1541442.05	1.22497669	1.9397E+12
cg19026976	0.04678575	0.15601211	0.02499131	0.97392962
cg13745957	0.04678629	27731.5979	1.15654805	664945587
cg03169018	0.04678989	5.01360413	1.02315726	24.5673146

cg21578457	0.04679203	0.18104432	0.03358168	0.97603947
cg18343556	0.04679557	20.7313305	1.04391001	411.709879
cg16303686	0.04681268	8580677578	1.38052648	5.3333E+19
cg26774156	0.04681887	6.02322547	1.02558707	35.374125
cg01528492	0.04681923	6.61183595	1.02693027	42.5699541
cg13572237	0.04681991	68.4225509	1.06123967	4411.48743
cg06351970	0.04682844	1118438429	1.33946004	9.3389E+17
cg13934106	0.04683385	38689260	1.27717693	1.172E+15
cg06189038	0.04683736	12.8233692	1.03633033	158.674114
cg16373176	0.04683943	9.81461754	1.03243961	93.3000985
cg01792145	0.04684612	15.8575806	1.03930068	241.953908
cg17014757	0.04684713	4.22949462	1.02031157	17.5325118
cg05727199	0.04684913	7.01044835	1.02750817	47.8306522
cg02614658	0.04685159	60.734028	1.05884415	3483.63084
cg17075352	0.04686075	5.58131148	1.02415739	30.4162603
cg06561044	0.04686829	593680.589	1.20214586	2.9319E+11
cg07234028	0.04687765	1417.84582	1.10538707	1818627.01
cg00078221	0.04687779	4.37780639	1.02059487	18.7784491
cg10896318	0.04687904	173.932382	1.07378866	28173.5827
cg07382149	0.04688208	6196186012	1.3645987	2.8135E+19
cg08450897	0.04688922	371.855247	1.08481524	127465.322
cg19272891	0.04690238	3.76E-08	1.79E-15	0.79125352
cg02382109	0.04690427	8.83696293	1.0302738	75.7972435
cg08303370	0.04691171	377579.9	1.19165411	1.1964E+11
cg25640615	0.04692466	8580684836	1.36476867	5.3949E+19
cg07312366	0.04692798	15763873.3	1.25243343	1.9841E+14
cg17279557	0.04692841	1144333.53	1.20857107	1.0835E+12
cg20720680	0.04692921	147.016632	1.07009922	20198.0244
cg03248196	0.04694346	4587821288	1.35066807	1.5583E+19
cg11992375	0.04695714	6.89351154	1.02630798	46.3023791

cg07476963	0.04696226	24.6509218	1.04397327	582.072323
cg09023593	0.04696642	76485917.2	1.27559644	4.5862E+15
cg04575343	0.04696998	18754824	1.25144321	2.8107E+14
cg18764192	0.04697068	321.260825	1.08035705	95531.8592
cg25244036	0.04697964	15.0153352	1.03682824	217.45192
cg21682902	0.0469905	0.0046649	2.34E-05	0.93109116
cg07713896	0.04699651	4.98469146	1.02155287	24.3229203
cg07842386	0.04699672	7.23416437	1.02661406	50.9764441
cg25485294	0.04700131	4.40389856	1.0198418	19.0169913
cg14230666	0.04701377	3.32921243	1.0159995	10.909115
cg07126559	0.0470172	6.96410676	1.02591315	47.2737705
cg16107483	0.04702611	19.5728881	1.03986036	368.412878
cg06996372	0.04702667	2.29E-05	6.01E-10	0.86900019
cg01894508	0.04703229	0.04948773	0.00254751	0.96134465
cg16215361	0.04703271	4.17569315	1.01891817	17.1126728
cg05551889	0.04704069	3.49983902	1.01651645	12.0498524
cg12564291	0.04704182	7.0707E+10	1.38618554	3.61E+21
cg23753351	0.04704675	4.61291337	1.02015149	20.8586371
cg07625430	0.04705445	55.042653	1.05355114	2875.69682
cg06685724	0.04705859	18.6679099	1.03877167	335.4836
cg06173216	0.04706687	4.53477616	1.01978533	20.1652193
cg00036328	0.04706703	1324.49645	1.09763649	1598243.91
cg05986680	0.04706791	4.3214E+11	1.41494498	1.32E+23
cg11977634	0.04707491	5086228602	1.33488106	1.938E+19
cg04706867	0.04707597	5.82152679	1.0230185	33.1276259
cg24619626	0.04708012	0.1346524	0.01860637	0.97446564
cg06491137	0.04708151	172.714494	1.0686821	27913.1621
cg13694291	0.0470837	102299.188	1.16024307	9019768430
cg03922571	0.04708538	4493061444	1.33135704	1.5163E+19
cg24260917	0.04708804	0.16953293	0.02940518	0.97742709

cg06052921	0.04709065	6.09586591	1.02350572	36.3061786
cg05263790	0.04709399	1800983.52	1.20312402	2.6959E+12
cg13733266	0.04709597	7.40683852	1.0260227	53.4698276
cg25189267	0.04709655	111.391243	1.06231849	11680.1215
cg21030939	0.04710122	3988411421	1.32723607	1.1985E+19
cg21989213	0.04710246	6.05207295	1.02331361	35.7931201
cg27601809	0.04710704	3.74887081	1.0170316	13.8186782
cg01938354	0.04711885	6665415.92	1.22137222	3.6375E+13
cg21663536	0.04712984	219.451614	1.0707387	44977.3702
cg25755953	0.04714008	70328.9823	1.15141047	4295744992
cg06182322	0.04714332	543935.781	1.18132583	2.5045E+11
cg01024247	0.04714606	10.9899137	1.03067624	117.183455
cg12871376	0.04715633	7.46261733	1.02556494	54.3024192
cg25170591	0.04715945	3.33261109	1.01521651	10.9398307
cg05468346	0.04716411	23.6270175	1.04040325	536.5573
cg18146843	0.04717231	3.30592936	1.01504435	10.7671837
cg00315391	0.04717498	697.543347	1.08511818	448399.752
cg21128569	0.04718184	4.83279256	1.0198005	22.9024049
cg03226554	0.04718634	0.00013014	1.89E-08	0.89478719
cg05802477	0.04718864	128193505	1.26084202	1.3034E+16
cg01434523	0.04719065	0.01825706	0.00035029	0.95154862
cg10768682	0.04719399	154.806576	1.06447206	22513.5793
cg07869548	0.04719845	7.8223719	1.02577424	59.6520169
cg20166027	0.04720161	4.22102792	1.01795465	17.5028197
cg01855674	0.04720171	17430.992	1.1282597	269299245
cg01535312	0.04720872	0.25200332	0.06459374	0.98315518
cg08386490	0.04721884	362.965722	1.07506733	122544.99
cg08757348	0.04721943	7.33642835	1.02476914	52.5222498
cg10852177	0.04722662	3.29026805	1.01469085	10.6691253
cg22610434	0.04723281	0.2649675	0.07135633	0.9839039

cg16994128	0.04723497	558023.008	1.17531942	2.6494E+11
cg08617683	0.0472404	4.57689943	1.01870478	20.563375
cg25938977	0.04724434	5.58038632	1.02113727	30.496107
cg24960291	0.04724615	0.19103557	0.0372365	0.98007577
cg15916160	0.04724859	3.68299477	1.01596281	13.3513258
cg27057288	0.04724889	29778.9233	1.13328724	782488540
cg26572856	0.04725065	644918.327	1.17629563	3.5358E+11
cg10593028	0.04725171	10.5640343	1.02901693	108.451881
cg13077262	0.04725746	4.19389721	1.0175095	17.2861029
cg27339291	0.04726016	927.436778	1.08615333	791913.035
cg09412882	0.04726262	8.27962928	1.02587356	66.8233042
cg26359174	0.0472631	6.64E-06	5.09E-11	0.86584026
cg12685200	0.04726576	30728564.3	1.2313437	7.6684E+14
cg19873719	0.04726634	3343.98386	1.10288679	10139053.4
cg04351402	0.04727436	27.155329	1.0405248	708.692275
cg06983052	0.04728324	0.17484305	0.03121615	0.97930378
cg12897782	0.04728518	448.215036	1.07590806	186722.942
cg19379572	0.04728603	317.494109	1.07144777	94080.656
cg11979743	0.04728704	36.2950819	1.04395096	1261.87247
cg00877620	0.04728851	3399126.76	1.19722034	9.6507E+12
cg19326232	0.04729155	5.2057E+18	1.67403218	1.62E+37
cg02129266	0.04729387	11281.8137	1.11790796	113854919
cg07126792	0.047294	12636716.6	1.21569722	1.3135E+14
cg13773678	0.04730638	28.4979967	1.04063074	780.426513
cg27459530	0.04731024	18.9768298	1.03555951	347.754106
cg19101460	0.04731208	264604299	1.25870507	5.5625E+16
cg00865356	0.04731974	199.888024	1.0646758	37528.0647
cg01755729	0.04732499	923083.911	1.17605194	7.2453E+11
cg04825775	0.04734886	65102.2053	1.13845874	3722837718
cg01508908	0.04734937	1.07E-05	1.30E-10	0.87467342

cg25418309	0.04735647	1739866078	1.28173013	2.3618E+18
cg06726167	0.04735765	0.00010656	1.26E-08	0.89883538
cg02497559	0.04735951	4.80043149	1.01844676	22.6267522
cg24853978	0.0473621	675972.057	1.16913176	3.9084E+11
cg05741490	0.04736345	7.04872594	1.0229805	48.568411
cg18668511	0.04736729	0.25941813	0.068361	0.98444673
cg09673200	0.04737065	283.825212	1.06772936	75446.7887
cg26438284	0.04737541	32.7086565	1.0412176	1027.50493
cg09853936	0.04737746	0.11008308	0.01243169	0.97478944
cg12446939	0.0473783	72.4172515	1.05078812	4990.78572
cg14732337	0.04737941	9.93400868	1.02690442	96.0990395
cg21112925	0.04738167	55905669.2	1.22887573	2.5433E+15
cg17067942	0.04738274	8548.95454	1.11021611	65829186.7
cg10828599	0.04738475	3.82028233	1.01558683	14.3705655
cg25163015	0.04739477	953.4438	1.08204748	840124.948
cg00777209	0.04740486	1.5463E+12	1.37900036	1.73E+24
cg25124300	0.04740761	0.21246547	0.04594845	0.9824396
cg13580783	0.04741003	9.08072096	1.02552945	80.4067529
cg10043760	0.04741602	4611.94517	1.10095153	19319686.4
cg14844588	0.04741763	8.13588539	1.02416999	64.6305122
cg16496965	0.04742469	141224663	1.23763844	1.6115E+16
cg23421262	0.04742515	8.83928119	1.0250631	76.2225193
cg13709054	0.04742714	679.872626	1.07683502	429245.685
cg10426076	0.04742957	0.12044457	0.01485928	0.97628481
cg14395298	0.04743058	3.22523541	1.01336187	10.2649841
cg04004578	0.04743486	14952660.5	1.20556019	1.8546E+14
cg14029489	0.04743937	2.87546459	1.0120023	8.17023501
cg06191536	0.04744618	4.86588409	1.0179846	23.2585327
cg26953749	0.04744693	10.486201	1.02682005	107.088298
cg15670863	0.04744927	68.982843	1.04879104	4537.25522

cg02119043	0.04745105	243932.48	1.14966978	5.1757E+10
cg02795515	0.04745233	4.48864673	1.01701771	19.8108147
cg07516307	0.04745326	4.30333104	1.01652974	18.2175271
cg26484001	0.04745379	7.48035121	1.02285832	54.7051858
cg21417843	0.04745554	1.6441E+11	1.33624041	2.02E+22
cg17758623	0.04745702	41446074	1.21743687	1.411E+15
cg23631538	0.0474696	4.80954905	1.01768409	22.7298061
cg01434121	0.04746984	906704379	1.25882406	6.5308E+17
cg26066361	0.04747368	4.16266358	1.01601838	17.0545813
cg05282518	0.04748603	0.20564318	0.04303727	0.98261626
cg06223477	0.04749727	37087317	1.21212309	1.1348E+15
cg09728459	0.04750156	3247159.36	1.17963461	8.9384E+12
cg02011430	0.04750636	4.80799145	1.0174186	22.7210135
cg00431427	0.04750678	0.05507265	0.00313124	0.9686262
cg07930752	0.0475078	26.076137	1.03649114	656.025792
cg22091798	0.04751031	29095969.4	1.20767464	7.01E+14
cg27565406	0.04751216	10279.4714	1.10666617	95482753.7
cg15074033	0.04751271	4.89496898	1.01757351	23.5469193
cg10930027	0.04751913	11.3970467	1.02697914	126.480343
cg05633748	0.04752129	22.0181788	1.03437374	468.689582
cg12419678	0.04752333	84.7568946	1.04968433	6843.7062
cg24791601	0.04752637	168149459	1.22949327	2.2997E+16
cg02360776	0.04752806	264397.989	1.14579088	6.1011E+10
cg09883798	0.04753822	0.24582927	0.06135952	0.98488428
cg18149485	0.047542	6.55139033	1.02058157	42.055154
cg13834500	0.0475524	3460396.86	1.1764419	1.0178E+13
cg26855280	0.0475574	0.05383478	0.00299083	0.96902248
cg10943458	0.04756079	3.46569905	1.01345685	11.8515849
cg05485968	0.04756833	3103.70453	1.09002577	8837389.03
cg17830174	0.04757156	3506143.51	1.17509657	1.0461E+13

cg05499148	0.04757585	12.1325771	1.02703404	143.32478
cg18953784	0.04758319	3.6014674	1.01374615	12.7946896
cg06976589	0.04759464	4.21338807	1.0153681	17.4839441
cg04160503	0.04760315	225271.21	1.13908636	4.4551E+10
cg05986933	0.04760656	6.12001967	1.01929736	36.7455488
cg04726784	0.04761625	4.68885512	1.01636886	21.6312829
cg27026926	0.04762177	2.30E-07	6.22E-14	0.85195004
cg20768239	0.04762648	40.551358	1.03949681	1581.93139
cg02537229	0.04763416	83679.9739	1.12546704	6221717570
cg22068044	0.04763749	9298.08049	1.09982433	78607372.1
cg16461538	0.04765975	6.55664477	1.01958457	42.1638303
cg21124756	0.04766066	576023554	1.23117494	2.695E+17
cg02952396	0.04766124	65441558.9	1.20381826	3.5575E+15
cg18213440	0.04766245	21829635.6	1.19016378	4.0039E+14
cg10096873	0.04766748	0.00337759	1.21E-05	0.94318109
cg18848419	0.04766989	15.5707337	1.02859329	235.70808
cg00435082	0.04767198	57433.0435	1.11899225	2947790287
cg25543401	0.04767264	4017.64198	1.08884144	14824423.9
cg27457323	0.04767757	167285262	1.21384868	2.3054E+16
cg09951650	0.0476781	1078698.82	1.15273831	1.0094E+12
cg00157359	0.04767998	5.33697808	1.01726912	27.999803
cg16963127	0.04768365	17.2645516	1.02950496	289.522397
cg20941820	0.04769229	3.21770826	1.01195536	10.2313272
cg11126068	0.04769892	2455870.5	1.16089993	5.1954E+12
cg12258454	0.04770282	7.32107378	1.02035559	52.5288651
cg00393825	0.04770453	0.20625184	0.04322454	0.98415913
cg06070625	0.0477073	6.34268131	1.01883741	39.4857959
cg19590063	0.0477103	23.3871115	1.03231376	529.835992
cg00159400	0.04771209	42603139.6	1.19374975	1.5204E+15
cg19308132	0.0477205	7.99121681	1.02109368	62.5403404

cg23584647	0.04773668	8.14747644	1.02113804	65.0072466
cg25975823	0.04773673	4627.55766	1.08779994	19685871.5
cg14666397	0.04774213	4.52537178	1.01513103	20.1737402
cg09427030	0.04774774	34974.6291	1.10939326	1102606917
cg25729257	0.04774876	6080.92996	1.0902584	33916463.5
cg13640145	0.04774877	4.78084162	1.01563884	22.5045022
cg07042289	0.04775068	58.4467038	1.0411365	3281.04643
cg10833037	0.04775648	8.48092569	1.02135432	70.4222808
cg01085125	0.04775795	6.34532197	1.01841772	39.5349671
cg08262764	0.04775937	69457.4148	1.11632793	4321608663
cg10105971	0.04776065	4.88048696	1.01576154	23.4495519
cg26477573	0.04776165	4.02742224	1.01383197	15.9988344
cg03265268	0.04776706	0.24815233	0.06242961	0.98638414
cg03045620	0.04777243	12.4130402	1.02502378	150.321943
cg01208563	0.04777371	34774213.9	1.18565294	1.0199E+15
cg27652925	0.04777545	6.68217607	1.01878756	43.828055
cg20279561	0.04777788	3.08577613	1.01109079	9.4175661
cg19405504	0.0477794	63.8237697	1.04149182	3911.19115
cg19466590	0.04777956	154687537	1.20254493	1.9898E+16
cg17031739	0.0477842	13.312174	1.02558844	172.792488
cg05335186	0.0477886	11.3581542	1.02395195	125.989963
cg23642078	0.04779077	41.8377499	1.0370024	1687.93951
cg00901493	0.04779598	28.1173135	1.03291998	765.386801
cg13616930	0.04779924	4.21753664	1.01404924	17.5411752
cg26488040	0.04779962	79120.5021	1.11551004	5611831016
cg12379294	0.04780441	7474459042	1.24589931	4.4841E+19
cg06228599	0.04780511	5.4359871	1.01650208	29.0702362
cg03470736	0.04782371	2.4216E+12	1.3143132	4.46E+24
cg05214460	0.04783219	15.6058946	1.02657909	237.238366
cg15526671	0.04783935	6.33485829	1.01772103	39.4316599

cg03547924	0.04784405	5.01131103	1.01541975	24.7318788
cg21244038	0.0478473	6.92067947	1.01850821	47.0254473
cg19813688	0.0478496	1.16E-07	1.58E-14	0.85968386
cg15416643	0.04785399	4.54440005	1.01440946	20.3582208
cg19189310	0.04785518	20185.1932	1.09814742	371026710
cg17859426	0.04786409	11.6058898	1.0233251	131.626478
cg15395329	0.04786492	6.18727244	1.01728205	37.6319824
cg02367316	0.04787255	3.49183478	1.01178256	12.0509194
cg12873037	0.04787259	190.661753	1.05041413	34607.2116
cg26841483	0.04787556	0.15452627	0.02429915	0.98268339
cg19139554	0.04787852	39374178.9	1.17746664	1.3167E+15
cg27038197	0.04788241	1.64E-05	2.98E-10	0.90235992
cg19448816	0.04788411	0.23181239	0.05447383	0.98647338
cg05978527	0.04788591	53.3162473	1.03770581	2739.33344
cg24548564	0.04788611	26.4155873	1.03094132	676.840898
cg18341924	0.04789305	56782.8705	1.10688727	2912938347
cg08971171	0.04789361	95.2751243	1.04316607	8701.72982
cg23130254	0.04790449	7.04301137	1.01817235	48.7186764
cg05319515	0.04790975	29.4450838	1.0316172	840.440586
cg20346388	0.04791143	6.36625448	1.01716574	39.8452234
cg23166970	0.04791912	92.8577699	1.04238204	8271.98195
cg27648075	0.04792525	3.58153446	1.01172081	12.6787835
cg10401367	0.0479285	114284367	1.18435646	1.1028E+16
cg10770742	0.04793013	3.14591271	1.010498	9.79394993
cg01201932	0.04793063	0.23872156	0.05773651	0.98703545
cg19705910	0.04793209	4.14417006	1.01302624	16.9533075
cg20939662	0.04793238	5.65728342	1.01589827	31.5039968
cg00143729	0.0479373	5.07732466	1.01486267	25.4016888
cg03973663	0.04793765	37.8142483	1.03352961	1383.52821
cg06952384	0.04794008	28.1501345	1.03072694	768.806984

cg08502873	0.04794093	20522221.7	1.16487198	3.6155E+14
cg03774988	0.04794518	12.5099793	1.02311592	152.963688
cg10494773	0.04794937	0.00018539	3.71E-08	0.9253664
cg19386159	0.04796093	146685.667	1.11267818	1.9338E+10
cg10322124	0.04796649	3.58370291	1.01148999	12.6970377
cg24413842	0.04797285	0.21738797	0.04790539	0.98647618
cg08525207	0.04797806	0.00294278	9.12E-06	0.94945413
cg02082342	0.04797816	6.66745575	1.01702641	43.7107295
cg17385737	0.0479795	12625.6834	1.08760625	146567640
cg10042437	0.04797959	66.293413	1.03799973	4233.92848
cg11478273	0.04798216	339.495421	1.05311634	109443.882
cg11802985	0.04798293	0.11672454	0.01388691	0.98111213
cg20256097	0.04798433	3665.17963	1.07551965	12490280.1
cg19599045	0.04798619	5.7468828	1.01561929	32.5187421
cg13450057	0.04798864	32037.9412	1.09618848	936362403
cg10684802	0.04798953	5.27407262	1.01482174	27.4095843
cg12253828	0.0479922	83617.3337	1.10533916	6325532234
cg13649519	0.04799985	32.1147707	1.03100972	1000.33829
cg10056203	0.04800311	9.44315956	1.01992764	87.4309697
cg23835812	0.04800395	2496.51056	1.07113239	5818669.16
cg20885078	0.04801367	10.8200658	1.02103442	114.661975
cg01704105	0.04802748	26.5248149	1.02886204	683.829101
cg08847991	0.04802787	29399.9066	1.09339456	790523878
cg18226096	0.04802926	16.5538614	1.02463754	267.441232
cg03124974	0.04803069	29.8673354	1.02987261	866.182589
cg03294496	0.04803781	5.85291299	1.01537279	33.737944
cg05153861	0.04804251	17030814.9	1.15420787	2.513E+14
cg14151259	0.04804677	4.57381877	1.01315209	20.6482504
cg12006072	0.04804811	6.32815555	1.01597183	39.416007
cg25150440	0.04805369	0.1830413	0.03399487	0.98556385

cg21483700	0.04805654	0.0717206	0.00526106	0.97772035
cg01890417	0.04805835	4.56304919	1.01305253	20.5531474
cg12059258	0.04805926	0.26174655	0.0692999	0.98861992
cg27395450	0.04806817	1772446.4	1.13007941	2.78E+12
cg08304059	0.04806975	5.39553673	1.01441741	28.6980648
cg09139491	0.04807001	5.6304286	1.0147826	31.2399189
cg07419575	0.04807683	11.8504803	1.02113891	137.526718
cg01337047	0.04808518	6.26443117	1.01557708	38.6411811
cg10277175	0.0480888	5.84690348	1.01495833	33.682447
cg12707693	0.04809349	3.56955843	1.01072924	12.6064893
cg26615130	0.04809454	34672542.2	1.1566537	1.0394E+15
cg12154261	0.04809541	17.8139436	1.02442365	309.770852
cg23547181	0.04809599	6058.72948	1.07567562	34125718.2
cg12223253	0.04809773	0.01485106	0.00022846	0.96538552
cg24573719	0.04809822	6.40829189	1.01566223	40.4329349
cg03264884	0.04810096	1519.23386	1.06311237	2171051.33
cg13726191	0.04810322	97.3816161	1.03894308	9127.71775
cg03531100	0.04810806	0.19560315	0.0387837	0.98651225
cg17016559	0.04810906	0.01721902	0.00030668	0.96677866
cg16764147	0.04811077	0.14954161	0.02271863	0.98433262
cg13177214	0.0481135	1.3985E+11	1.23734418	1.58E+22
cg15621458	0.04811781	9.99650178	1.01924358	98.0433425
cg10500167	0.04812443	0.14708793	0.02197969	0.98431154
cg15734706	0.04812862	14222.0355	1.08189321	186955877
cg15791348	0.04813072	2727563.47	1.12957538	6.5862E+12
cg03790378	0.04813651	8.05642265	1.01724853	63.8053966
cg09902913	0.04814853	0.00186598	3.66E-06	0.9501162
cg00057324	0.04815299	128.432252	1.04023012	15856.9175
cg12973168	0.04815352	547320926	1.1775058	2.544E+17
cg22783598	0.04815462	110032.897	1.09879699	1.1019E+10

cg08941457	0.04815808	6.25689497	1.01496509	38.5715084
cg19270251	0.04816169	18.7101739	1.02396345	341.87803
cg12647587	0.04816931	0.08400469	0.00719892	0.9802562
cg14352682	0.04818162	14.9099173	1.02184173	217.55388
cg12192818	0.04818593	11.1853539	1.01944884	122.725277
cg21477861	0.04818913	26.8971206	1.02656146	704.736269
cg14573676	0.04819824	1252255.79	1.11766304	1.4031E+12
cg15978899	0.04822943	6.99141652	1.01525378	48.1455038
cg10746615	0.04824151	6606195.44	1.12908304	3.8652E+13
cg07468655	0.04824158	0.14466695	0.02124368	0.985165
cg08830818	0.0482418	32.0041301	1.02715278	997.18792
cg23979631	0.04824321	3.63559424	1.01001929	13.0864288
cg25160915	0.04824616	10.2719478	1.01812329	103.634709
cg04638014	0.04825467	4.57672574	1.01173864	20.7033889
cg01575614	0.04825839	51386202.3	1.14561108	2.3049E+15
cg00365945	0.04826387	8.43439376	1.01640721	69.9906468
cg13841742	0.04827313	31.3357401	1.0264945	956.584388
cg23036508	0.04827451	957.376257	1.05344411	870069.223
cg24740531	0.04827537	3.12428085	1.00867395	9.67719138
cg27638778	0.04827596	2404.86912	1.06077661	5452039.01
cg00953403	0.04829543	2.72E-06	8.13E-12	0.90844634
cg01193217	0.04829571	5.56954261	1.01294774	30.6233024
cg04940435	0.04830156	23.4974892	1.02384687	539.272051
cg23531257	0.04830286	7.90177306	1.01553893	61.4826431
cg17035770	0.04830297	9.90778561	1.01725309	96.4993046
cg27253887	0.04830342	293698.749	1.09843463	7.8529E+10
cg07954885	0.04831026	109.460677	1.03548788	11571.0092
cg16740067	0.04831709	619.630583	1.04870187	366111.734
cg14552404	0.04831969	0.16758342	0.02845713	0.98689495
cg10397322	0.04833057	18.7796886	1.02175059	345.169075

cg15038286	0.04833466	5.65501118	1.01276078	31.5762142
cg15648905	0.04833468	98027.8212	1.08775219	8834230666
cg08460435	0.04833496	7.61276533	1.01496396	57.0997573
cg27558095	0.0483547	3.48087015	1.00905873	12.0076826
cg02205739	0.04835506	7.28375697	1.01445669	52.2970732
cg16417118	0.04835519	10.7793189	1.01733379	114.213956
cg15680518	0.04835819	508149.48	1.09942755	2.3486E+11
cg05312338	0.0483587	2.8075E+10	1.18948041	6.63E+20
cg15662902	0.04836476	14.8735731	1.01958748	216.973217
cg23322523	0.04837178	3.88060791	1.0097487	14.9137283
cg09937438	0.04837318	7.57338035	1.01457803	56.5319652
cg08109136	0.04837699	35.5448313	1.02579203	1231.66781
cg19554255	0.0483814	6.22475474	1.01308936	38.2469434
cg18665732	0.0483838	1.0001E+11	1.19705816	8.36E+21
cg21347053	0.04838545	2.79490092	1.0073179	7.75472288
cg09914675	0.04838713	1.6957E+10	1.18165694	2.43E+20
cg27476810	0.04839	4.41E-08	2.20E-15	0.88709556
cg20439473	0.04839082	6.43666362	1.01325201	40.8887803
cg04237075	0.04839154	169039.423	1.08879661	2.6244E+10
cg00815931	0.04839364	6.77E-05	4.90E-09	0.93448492
cg06445533	0.04840951	11074.0886	1.06723532	114909464
cg11884792	0.04841926	3.10E-05	1.04E-09	0.93045075
cg17333211	0.04842282	6.63086366	1.01319366	43.3958033
cg12058064	0.04842759	6.70272781	1.01322872	44.3399987
cg24815885	0.04843474	19008486.5	1.12214935	3.2199E+14
cg02377114	0.04843508	1117.24379	1.04943303	1189436.25
cg04630810	0.04843548	10176647	1.11727977	9.2693E+13
cg22974452	0.04843797	31753099.1	1.12583693	8.9556E+14
cg16216311	0.04844049	82.322306	1.03067681	6575.25423
cg26518431	0.04844909	0.01028537	0.00010914	0.96929956

cg01376829	0.04845104	14.6759544	1.01844507	211.482822
cg03948207	0.04845346	6744.20051	1.06172272	42840036.7
cg06154570	0.04846293	36.6666171	1.02461584	1312.14135
cg03126490	0.04846768	50267269.4	1.12676427	2.2425E+15
cg24867946	0.04846769	659904.7	1.09437825	3.9792E+11
cg02764093	0.04847238	5.53819126	1.01155092	30.3213232
cg01761478	0.04847256	579.063626	1.04360156	321305.271
cg27299725	0.04847372	43825949.3	1.1251956	1.707E+15
cg09196146	0.04847448	5.86422366	1.01192264	33.9839408
cg06568815	0.04847919	63.3639179	1.02810029	3905.24752
cg08961621	0.04848069	6.71508575	1.01278876	44.5229829
cg13509954	0.04848355	8374357573	1.16436693	6.023E+19
cg23244095	0.0484846	17.2668405	1.01914145	292.544063
cg18101225	0.04848671	11.0580118	1.01610037	120.342073
cg01315067	0.04848852	4.75457444	1.01040364	22.3732152
cg12453687	0.04848996	4.47880299	1.00999326	19.8611982
cg23197992	0.04849615	66.6784853	1.02812691	4324.3887
cg26406131	0.04850027	9453.71192	1.0621478	84143345
cg21970438	0.04850455	0.19475235	0.0383382	0.98931294
cg27262137	0.0485105	922694592	1.14456924	7.4383E+17
cg20702204	0.04851137	0.00466963	2.26E-05	0.96552441
cg03785807	0.04851682	396496.529	1.08758438	1.4455E+11
cg25347337	0.04852017	64072677.5	1.12390972	3.6527E+15
cg26763394	0.04852488	9.17040506	1.01445787	82.8978036
cg06490744	0.04854595	14016.2177	1.06285629	184836238
cg27022535	0.04855526	0.02706037	0.00074922	0.97736229
cg14485377	0.04855753	7420.45507	1.05806696	52041274.7
cg18332146	0.04855768	9751.62477	1.05989262	89720585
cg10976961	0.04855893	5.7673629	1.01114834	32.8957419
cg08575330	0.04855899	3.09677828	1.00717743	9.52169445

cg09863772	0.04856114	11.2796114	1.01542498	125.296931
cg15945287	0.04856411	4.66808849	1.00976079	21.5804083
cg10243292	0.04856434	81509.7789	1.07388307	6186748133
cg03584535	0.04856633	5.64915871	1.01095865	31.5670617
cg20259594	0.04857388	0.00117109	1.43E-06	0.95861844
cg18773937	0.04857739	0.20520701	0.04252852	0.9901572
cg04645421	0.04858054	4256560769	1.14817015	1.578E+19
cg22898783	0.04858774	5.49944972	1.01062486	29.9259878
cg05414026	0.04859078	179951.951	1.07773364	3.0047E+10
cg08381596	0.04859084	2092.91697	1.04843929	4177925.7
cg04400030	0.04859195	59453.0603	1.07031405	3302457229
cg02970551	0.04859308	4.79410133	1.00972789	22.7619816
cg01294194	0.04860796	0.05696374	0.00330218	0.98264307
cg16044565	0.04861069	941722958	1.13430288	7.8184E+17
cg03609847	0.04862121	4.9148E+11	1.17695501	2.05E+23
cg16066354	0.04862393	7.57854873	1.01230855	56.7360621
cg10800003	0.04864452	49740.8218	1.06645451	2319976466
cg14610403	0.04864572	4.21984405	1.00859495	17.6553371
cg02928278	0.04864717	0.21154505	0.04516596	0.99081936
cg03330710	0.04865075	9.52339626	1.01343582	89.4926691
cg07048608	0.04866145	0.28095341	0.07952573	0.99256952
cg16819803	0.04866199	6.38781802	1.01094894	40.3622945
cg00765921	0.04866976	0.00023904	6.00E-08	0.95248398
cg07906941	0.04869388	1219527731	1.12739947	1.3192E+18
cg06019229	0.04869748	727.890406	1.0383852	510238.824
cg09661370	0.04870307	5.07986051	1.00929237	25.567401
cg11811341	0.04870345	0.1528227	0.02360571	0.98936968
cg19379845	0.04870634	2125.16716	1.04445117	4324123.12
cg08616234	0.04870739	0.14948657	0.02258843	0.98927817
cg10645113	0.04871146	45.2834394	1.02179223	2006.85602

cg21704448	0.04871204	6.62198224	1.01074067	43.3846682
cg24814117	0.04872005	83.3866722	1.02515383	6782.7256
cg06615912	0.04872422	115573.729	1.06743229	1.2513E+10
cg00302587	0.04872621	4.01055182	1.00779278	15.960152
cg15088822	0.0487276	1.1479E+10	1.13805233	1.16E+20
cg04055835	0.0487342	8.32846299	1.01184155	68.5515395
cg13727946	0.04873496	20.8497369	1.01700115	427.444481
cg11948341	0.04874364	3693837.28	1.0869267	1.2553E+13
cg22729335	0.04874436	23.0468518	1.01743446	522.055619
cg08172935	0.04874944	2.447E+10	1.14024171	5.25E+20
cg07681419	0.04874994	33349493.6	1.09966034	1.0114E+15
cg12356261	0.04875144	17.0261349	1.01564883	285.422739
cg17864619	0.04875682	7277.12728	1.04969404	50449540
cg07097863	0.04875973	1038163.58	1.07828858	9.9953E+11
cg20865068	0.04877867	1757126.72	1.08008235	2.8586E+12
cg04446777	0.04878344	816105.81	1.07534587	6.1936E+11
cg24348448	0.04878856	0.16111601	0.02621143	0.99034556
cg13614659	0.04878988	1385.05416	1.03914326	1846112.19
cg21205305	0.04879423	7.71082823	1.01086198	58.8179918
cg20119464	0.04879675	5.44014204	1.00897971	29.3317548
cg20277416	0.04879752	9.22817217	1.01179022	84.1668165
cg02192204	0.04880004	5.70142739	1.00920409	32.2098124
cg12207922	0.04880015	1.3034E+13	1.17225733	1.45E+26
cg21080336	0.0488094	8.5276181	1.01125544	71.9108815
cg24863255	0.04881205	4.43605676	1.00779249	19.52644
cg19059861	0.04881224	0.17092784	0.02948644	0.99083944
cg04324917	0.04882023	126.555612	1.02536389	15620.1356
cg14602697	0.0488282	100.171997	1.02395851	9799.6442
cg03288621	0.04882871	11300.3689	1.04910912	121720738
cg06909469	0.04882927	5.24620862	1.00854686	27.2894655

cg20837456	0.04883209	0.00299835	9.26E-06	0.97068055
cg16512615	0.0488457	3.56264677	1.00645218	12.611083
cg17161421	0.0488461	4.58191166	1.0077322	20.8328309
cg21237687	0.04884613	5.59736826	1.00875327	31.0586664
cg16655905	0.04885042	7853083149	1.12171898	5.4979E+19
cg00973653	0.04885352	4.25461075	1.00730665	17.9704091
cg05350938	0.04885684	22438.058	1.05150564	478805276
cg00679681	0.0488575	113531.853	1.06005143	1.2159E+10
cg26234644	0.04886686	0.15709801	0.02490781	0.99084532
cg15439862	0.04886892	5.4091861	1.00840782	29.0153387
cg12567545	0.04887105	8.0227907	1.01036153	63.7050881
cg11806528	0.04887122	4.5091822	1.00748266	20.1817113
cg08260245	0.04887585	11.5593296	1.01213753	132.015756
cg00482226	0.04888078	0.06489328	0.00426804	0.98666735
cg10755961	0.0488849	16.5355143	1.01381187	269.698197
cg00026374	0.04888551	141752.099	1.05967855	1.8962E+10
cg17144305	0.04888735	5.00673047	1.00788937	24.8711325
cg04087789	0.048888	11381.2244	1.04659104	123765888
cg24543526	0.04889182	210958.737	1.061378	4.193E+10
cg06759097	0.04889353	12.85446	1.01246609	163.202643
cg18385671	0.04889395	236.806694	1.02686849	54610.1191
cg08097581	0.04890117	13.9282185	1.0127704	191.54911
cg21187597	0.04890345	5.09020843	1.00785441	25.7082983
cg13711238	0.04890894	7.90640127	1.00993986	61.8959441
cg01515913	0.04890958	4521513.7	1.07601163	1.9E+13
cg08199563	0.04890964	0.11530491	0.01343323	0.98972654
cg12914657	0.04893606	0.21256208	0.04551015	0.99280355
cg00664406	0.04893872	9.47839673	1.01051827	88.9048791
cg06928741	0.04893878	0.0017745	3.24E-06	0.97096216
cg24028828	0.04894104	1745.03999	1.0352591	2941451.62

cg20643029	0.04894629	5.53101123	1.00793161	30.3513501
cg17857094	0.04894739	0.09574091	0.00926609	0.98923289
cg27083087	0.04895548	0.18898687	0.03598953	0.9924007
cg05042697	0.04897297	0.06893995	0.00481028	0.98803251
cg12303084	0.04897867	492.557087	1.02814146	235971.889
cg20701850	0.04898418	626594.653	1.061231	3.6997E+11
cg11253514	0.04898689	6.09695326	1.00805951	36.8756395
cg18719665	0.04899329	6.07133985	1.00798949	36.569
cg02310229	0.04899502	105.909252	1.02074907	10988.763
cg03809994	0.04899872	2613.69282	1.03513257	6599531.64
cg03446876	0.04900059	573.342916	1.02821043	319703.137
cg02647878	0.04900063	3.75085466	1.00580694	13.987685
cg16034458	0.04901172	24.382558	1.01392905	586.34195
cg12434312	0.04901676	8.03693144	1.00902044	64.0148253
cg00187606	0.04901761	1650.83297	1.03241145	2639693.23
cg13547250	0.04902325	13005201.4	1.07263261	1.5768E+14
cg10825332	0.0490242	3616696.88	1.06670541	1.2263E+13
cg25538450	0.04902582	358.202039	1.02542453	125127.395
cg13797775	0.04903888	1.9656E+12	1.12661357	3.43E+24
cg11391462	0.04904338	0.14601367	0.02149264	0.99196721
cg19189012	0.04904908	1.3838E+10	1.102187	1.74E+20
cg10134204	0.04905341	4.73607568	1.00647137	22.2861907
cg25228510	0.04906514	1655.42879	1.03082483	2658496.79
cg09158314	0.04906765	10.6066565	1.00969345	111.421107
cg03163184	0.04907502	4.58957297	1.00619444	20.9345026
cg08584381	0.04908233	532711551	1.0841382	2.6176E+17
cg27277403	0.04908308	2637.53137	1.03215179	6739872.76
cg18238973	0.04908321	4.69227733	1.00622867	21.8811758
cg23627301	0.04909457	15.6340581	1.01096577	241.772549
cg15174949	0.04910284	2922002044	1.08943936	7.8371E+18

cg08495027	0.04910607	1291887.77	1.05665234	1.5795E+12
cg14995433	0.0491066	61480.35	1.04409992	3620183612
cg16548780	0.04910685	6.41360133	1.00729786	40.8362646
cg21545902	0.04912903	4.31931301	1.00559752	18.5526163
cg22160377	0.04912984	640.261092	1.02493582	399960.913
cg06813260	0.04913356	5.81909303	1.00670635	33.6362671
cg05597836	0.04913523	6.48121054	1.00710432	41.7097704
cg22438763	0.04914304	4.39040754	1.00556853	19.1689356
cg20770175	0.04914419	5.79868448	1.00661029	33.4039321
cg11606466	0.04914464	0.00062271	3.99E-07	0.972724
cg18353550	0.04914557	9.9512E+12	1.11851963	8.85E+25
cg08371050	0.04915289	4.44309976	1.00554868	19.6322027
cg25041172	0.0491556	2226283	1.05554209	4.6955E+12
cg20412726	0.04916493	81999.7747	1.04224904	6451397662
cg06852305	0.0491683	0.13596618	0.01862166	0.99275821
cg17513943	0.04917131	279815976	1.07314057	7.2961E+16
cg00658394	0.04917242	45.4711647	1.01393092	2039.21863
cg25054416	0.04917276	9.95469364	1.00836049	98.274304
cg22534097	0.04918291	0.23989809	0.05784583	0.99490476
cg02931001	0.04918415	8.62214673	1.0077269	73.7713904
cg01985396	0.0491845	7.65430725	1.00729519	58.1641012
cg00220769	0.0491953	6000.91372	1.03113166	34923731.8
cg14856679	0.0492128	6.37761337	1.00640707	40.4150106
cg16170919	0.04921417	0.18321945	0.03376598	0.99417736
cg13296916	0.04921679	8.96E-07	8.42E-13	0.95336418
cg21233517	0.04921891	38.5007215	1.01256421	1463.91265
cg13394083	0.04924005	1077976.32	1.04730345	1.1095E+12
cg03836184	0.04924014	4.61781543	1.00510296	21.2159552
cg27613455	0.0492405	51.1875143	1.01317292	2586.0952
cg23629792	0.04924256	51.7323226	1.01317239	2641.43913

cg16925970	0.04924871	144323.754	1.03984991	2.0031E+10
cg06959340	0.04925136	2105.0538	1.02539821	4321493.31
cg22093805	0.04925424	44.3136002	1.01245555	1939.53715
cg08391287	0.04925546	5991714790	1.07614633	3.336E+19
cg00590039	0.0492567	4.48635116	1.00489669	20.0292697
cg23923856	0.04925938	3.55934326	1.00412502	12.6168795
cg20944895	0.04926978	5.327E+12	1.09820595	2.58E+25
cg01629435	0.04927074	4.83377111	1.00504295	23.2481042
cg20106260	0.04927176	179.400626	1.01668263	31656.4712
cg06191810	0.04927376	8.76459093	1.00692528	76.2897268
cg11686214	0.04927891	12564273.3	1.05295457	1.4992E+14
cg15714196	0.04927892	2409565.2	1.04747953	5.5428E+12
cg23548719	0.04928718	31365987.9	1.0553382	9.3224E+14
cg01316923	0.0493005	36.1835734	1.0110486	1294.94366
cg21380181	0.04931411	0.04882919	0.002406	0.9909762
cg03874868	0.04931454	0.00191193	3.72E-06	0.98139472
cg27203983	0.04931603	0.13975279	0.01964624	0.99412602
cg17366544	0.04932908	5.58075022	1.0050615	30.9879275
cg27492942	0.04933021	17.4105199	1.00841064	300.59798
cg05964918	0.04933089	99.2788632	1.01355625	9724.46535
cg00937681	0.04933508	24.5256709	1.00935508	595.93353
cg09133836	0.04934304	0.01934906	0.00037866	0.98872119
cg13611499	0.04935256	9087.77237	1.02616206	80482030.9
cg06126494	0.04935392	6.84859236	1.00545498	46.6487492
cg10208370	0.04935836	4.61321681	1.00430244	21.190598
cg15793258	0.04935954	5.53505081	1.00480738	30.4902093
cg10888878	0.04936573	15.88971	1.00770604	250.552118
cg14558880	0.0493748	3.55012546	1.00347228	12.5597797
cg05457264	0.04938322	3.3425E+10	1.06758766	1.05E+21
cg21910650	0.04938383	6.83249707	1.00519475	46.4417627

cg11933779	0.04938685	4.61747352	1.004113	21.2337274
cg19725972	0.04938771	9.62270556	1.00608455	92.0364619
cg01682784	0.04938799	0.17948519	0.03236346	0.9954107
cg13934277	0.0493884	113443.366	1.03163858	1.2475E+10
cg02396253	0.049397	23.7982812	1.00839792	561.64157
cg03278924	0.04940589	234710510	1.0513777	5.2397E+16
cg01077764	0.04940667	12.0781371	1.00648873	144.940912
cg05647602	0.04940838	19.4489534	1.00771182	375.367025
cg12718426	0.04942412	347645.146	1.03266862	1.1703E+11
cg17216414	0.04942454	3880.92605	1.02102369	14751456.9
cg00619097	0.04942619	35846.1151	1.02667663	1251556653
cg04857420	0.04942737	3.81490759	1.00335996	14.5047844
cg21149807	0.04943044	436454202	1.05082178	1.8128E+17
cg17300047	0.04943637	0.08974131	0.00810152	0.994073
cg02084087	0.04944043	4.4628422	1.00366841	19.8441639
cg06512974	0.04944452	8.72635576	1.00527821	75.7494634
cg16856286	0.04944906	0.31183384	0.09751383	0.99719542
cg09408143	0.04945315	0.12097314	0.01470863	0.99495989
cg14677130	0.04945361	13.2421401	1.00619397	174.274821
cg01817464	0.04945584	768403.889	1.03278583	5.717E+11
cg07279963	0.04945836	9.42E-05	9.07E-09	0.97827462
cg10104451	0.0494585	11.8470268	1.00587301	139.532569
cg10277546	0.04945931	356240443	1.04767534	1.2113E+17
cg10094907	0.04946101	124.200352	1.01143373	15251.3476
cg05922057	0.04946969	190.170484	1.01224818	35727.2196
cg10911971	0.04947252	1186397302	1.04939072	1.3413E+18
cg18476690	0.04947639	8.64597472	1.00495275	74.3844714
cg11721566	0.0494806	0.07063985	0.00502012	0.99399718
cg10010922	0.04948806	774935164	1.04689864	5.7362E+17
cg14240963	0.04949117	253355.418	1.02807861	6.2436E+10

cg24860534	0.04949591	8.21319339	1.00465354	67.1440883
cg02376018	0.04950151	54.1048388	1.00873915	2901.97281
cg08230472	0.04950541	1602.08859	1.01609028	2526043.12
cg23151421	0.04950705	12.9245181	1.00553264	166.124064
cg16961218	0.04950743	1097299.86	1.03041626	1.1685E+12
cg27616378	0.04950947	5060.59445	1.01846662	25145268.2
cg22981993	0.04951192	5.21512978	1.00353168	27.1018636
cg06051154	0.04951434	126273.855	1.02526318	1.5552E+10
cg14513281	0.04952128	2514.53061	1.01652782	6220060.16
cg07886701	0.04952158	4.65287202	1.00322202	21.5796878
cg11904960	0.04952355	1.3245E+11	1.054811	1.66E+22
cg08196359	0.04952866	4.48E-05	2.05E-09	0.97956966
cg03478969	0.04953713	5.23697576	1.00335714	27.3341505
cg14878320	0.04954031	1.927E+12	1.0585108	3.51E+24
cg02512505	0.04954306	3.69677123	1.00261597	13.6304606
cg20271517	0.04954381	3.8731005	1.00270482	14.9604421
cg01966878	0.04955117	5.351166	1.0032974	28.5408669
cg18180874	0.04955733	59096860	1.03524469	3.3735E+15
cg08655788	0.0495644	20.3866905	1.00575893	413.237347
cg25020279	0.04956583	4600137120	1.04314328	2.0286E+19
cg03428881	0.04956642	11672596	1.03133169	1.3211E+14
cg25207721	0.04956664	320973.421	1.02431655	1.0058E+11
cg17971306	0.04956882	126989.332	1.02240363	1.5773E+10
cg05058516	0.0495711	2.32E-09	5.59E-18	0.96340122
cg00378234	0.04957445	0.2103351	0.04436938	0.99710331
cg16822666	0.04958121	4.68371571	1.00283133	21.8752567
cg27221266	0.04958258	46709.4964	1.01981659	2139381802
cg16673929	0.04958564	5.97893315	1.00324497	35.6320169
cg01940235	0.04958836	7.17E-06	5.25E-11	0.97890687
cg26427777	0.04959139	83.8366244	1.00794345	6973.18845

cg11725331	0.0495938	1794.065	1.01339462	3176126.22
cg16918905	0.04959383	3.26177855	1.00210167	10.6168862
cg11865553	0.04959667	4.47539643	1.00264608	19.9763143
cg01144344	0.04960064	43.9498423	1.00662704	1918.87219
cg16995385	0.04960462	5126.67825	1.01487535	25897594
cg14753321	0.0496119	4.05881689	1.00237969	16.4348845
cg08939418	0.04961215	0.27941321	0.07824072	0.99784034
cg24680852	0.04961454	9.9836E+10	1.04360177	9.55E+21
cg13676449	0.04963104	9.13787506	1.00357472	83.2033317
cg21573692	0.0496318	7.2193E+10	1.04106311	5.01E+21
cg08517562	0.04963193	7927307361	1.0373548	6.0579E+19
cg11547878	0.0496378	98.1748927	1.00728846	9568.56942
cg04871873	0.04964387	5187881	1.02436183	2.6274E+13
cg17789012	0.04965005	28142.989	1.015795	779712278
cg25922808	0.04965246	977.147246	1.01051346	944882.753
cg02602601	0.04966354	319251.142	1.01881285	1.0004E+11
cg01565314	0.04967054	4.9593516	1.00230844	24.5385226
cg24198953	0.04967377	1.4388E+11	1.03731217	2.00E+22
cg21589375	0.0496785	0.14700389	0.02166844	0.99730956
cg13067194	0.0496876	46296.198	1.01477536	2112130471
cg01185682	0.04968992	4.07474807	1.00190557	16.5719928
cg13447915	0.04969165	435.277993	1.00822143	187921.944
cg08133669	0.04969321	19.8468621	1.00401431	392.32303
cg25342397	0.04969525	173.162011	1.00688817	29779.9528
cg13115617	0.04970402	7.2768E+12	1.03905059	5.10E+25
cg09284708	0.04970641	6.07046402	1.00231646	36.7653678
cg08704217	0.04970692	89297.3458	1.01470748	7858438113
cg00013441	0.04970924	105.776028	1.00594046	11122.4953
cg15551144	0.04971169	1056552844	1.02652483	1.0875E+18
cg18870532	0.04971596	225.469898	1.00674794	50495.9309

cg02284939	0.04971663	54.2397935	1.00495735	2927.44283
cg24493649	0.0497233	4.9519294	1.00193618	24.4742184
cg27141863	0.04972669	14593135.6	1.01989635	2.0881E+14
cg16519772	0.04973135	17.6239773	1.00337393	309.560142
cg25715035	0.04973303	14.6127879	1.00313342	212.866571
cg05815275	0.04974168	6.77731981	1.00216222	45.832963
cg00441434	0.0497418	10.8837068	1.00269692	118.13647
cg10177147	0.04975109	7.24E-11	5.37E-21	0.97492618
cg16292132	0.04975286	4.71862231	1.00167676	22.2281253
cg24101459	0.04975767	0.17121673	0.02937	0.99813315
cg17978274	0.04975854	0.24978375	0.06248329	0.99853766
cg26213561	0.04976904	114173544	1.01889756	1.2794E+16
cg03824617	0.04976922	13.5614561	1.00263219	183.430266
cg13073564	0.04977025	15.0313944	1.0027241	225.328997
cg26130726	0.04977132	0.00271452	7.41E-06	0.99411373
cg02988727	0.04977494	6.13388465	1.00178509	37.5574975
cg19877419	0.04978207	12.3905059	1.00239916	153.157187
cg09382800	0.0497849	42.7261029	1.00353457	1819.09017
cg09164920	0.04978917	19245369.2	1.01556827	3.6471E+14
cg03087937	0.04979452	4.62045037	1.0013748	21.319252
cg01241758	0.04979591	2.036E+12	1.02559031	4.04E+24
cg04222409	0.0497978	154269.687	1.01060807	2.3549E+10
cg05382097	0.04980246	5.0982971	1.00140664	25.9561223
cg01973862	0.04980444	74815.1479	1.00963331	5543900240
cg24212855	0.04981383	6.94E-06	4.87E-11	0.99038733
cg01477546	0.049818	0.01245479	0.00015566	0.99651944
cg26715571	0.04982493	5.01189139	1.00123335	25.0881128
cg26147668	0.0498279	0.0001978	3.94E-08	0.99360963
cg21810188	0.04982832	3.56383542	1.00095344	12.6888249
cg18449964	0.04982895	6.11644503	1.00135392	37.3603171

cg00019495	0.04982995	40.7962447	1.00275832	1659.75545
cg26104206	0.04983202	7.94853496	1.00152209	63.0831894
cg17689707	0.0498321	3.28103935	1.00087174	10.7558429
cg16131766	0.04984233	0.26648934	0.07108127	0.99908974
cg16704246	0.04984368	4.96304723	1.00109435	24.6049115
cg24603926	0.04984394	3.95326144	1.00093733	15.6136409
cg24249542	0.04984934	8.17656117	1.00138359	66.763779
cg27391511	0.04986	0.12430541	0.01547155	0.99872596
cg17658213	0.04986249	23.7917326	1.0019052	564.970158
cg16141056	0.04986922	5.28057252	1.00095087	27.8579568
cg09988291	0.04987124	170.35199	1.00289333	28936.0788
cg25212146	0.04987266	3266.73296	1.00450991	10623632.6
cg21616089	0.04987338	5.95288673	1.0009869	35.4019224
cg20541120	0.04987948	54.9276731	1.0021106	3010.69489
cg03759613	0.04988523	27178928.8	1.0086164	7.3238E+14
cg22816534	0.0498924	2586841.65	1.00696241	6.6455E+12
cg00590620	0.04989605	6.68578189	1.00086282	44.6611451
cg25983531	0.04989798	0.24060832	0.05792911	0.99936557
cg08786829	0.04990157	5.11980253	1.00070216	26.1939856
cg09268877	0.04990197	1.5691E+12	1.01209355	2.43E+24
cg00856204	0.04990895	221.7282	1.00214973	49057.9333
cg00711072	0.04991039	5.64470518	1.0006774	31.8411274
cg14930269	0.049916	2968888.63	1.00548124	8.7662E+12
cg18120358	0.04992927	0.23881957	0.05706002	0.99955786
cg02915785	0.0499394	5.66417664	1.00045896	32.068179
cg19955956	0.04994188	0.03097934	0.00096057	0.99911875
cg00699945	0.04994358	4.13818664	1.00034989	17.118599
cg16989018	0.04994929	0.07974336	0.00636257	0.99944023
cg11905617	0.04995242	160149.757	1.00249258	2.5584E+10
cg25589890	0.04996321	11.3216245	1.00038979	128.129239

cg05883932	0.04997607	211634975	1.00200481	4.47E+16
cg10527285	0.04997786	2650443895	1.00209958	7.0101E+18
cg12431087	0.0499867	5.52560683	1.00009921	30.5293019
