# Supplementary material for: Mining the Prognostic Role of DNA Methylation Heterogeneity in Lung Adenocarcinoma
Source: Dis Markers. 2022 May 28;2022:9389372. doi: 10.1155/2022/9389372 (PMC9168807; doi:10.1155/2022/9389372)
Supplement: Supplementary 8 — Supplementary Table 2: methylation sites and annotated genes in each subgroup. [file 9389372.f8.pdf]

Table S2

CpG	Chrom	Start	End	GeneSymbol	Feature_Type	Cluster
cg04781638	chr15	82572041	82572042	CPEB1	.	C5
cg08932256	chr12	115570453	115570454	RP11-1028N23.3	.	C2
cg27274426	chr2	86563964	86563965	CHMP3	S_Shore	C4
cg02721176	chr10	116325075	116325076	CCDC172	.	C4
cg24874612	chr17	4786470	4786471	VMO1	S_Shore	C4
cg24041078	chr11	111513164	111513165	BTG4	Island	C5
cg09937190	chr20	15196863	15196864	MACROD2	.	C4
cg12778476	chr11	22337799	22337800	SLC17A6	N_Shelf	C5
cg26942121	chr11	8810656	8810657	ST5	.	C4
cg21401879	chr2	44934897	44934898	RP11-89K21.1	Island	C1
cg14273450	chr2	26402066	26402067	DRC1	Island	C5
cg10879207	chr12	66235616	66235617	AC078889.1	Island	C5
cg06289844	chr6	125750392	125750393	RP11-624M8.1	S_Shore	C5
cg07139165	chr11	133953230	133953231	IGSF9B	N_Shelf	C4
cg21211321	chr11	133946347	133946348	IGSF9B	.	C4
cg01645998	chr16	82011617	82011618	SDR42E1	Island	C4
cg02703870	chr16	82011535	82011536	SDR42E1	Island	C4
cg04886060	chr16	82011183	82011184	SDR42E1	N_Shore	C4
cg10147797	chr16	82011473	82011474	SDR42E1	Island	C4
cg15242360	chr6	39886754	39886755	RP11-61I13.3	.	C4
cg01250407	chr7	117324748	117324749	WNT2	S_Shore	C5
cg17161421	chr6	32152300	32152301	PPT2	S_Shore	C1
cg21037008	chr6	32152162	32152163	PPT2	S_Shore	C1
cg07517893	chr19	45385906	45385907	PPP1R13L	Island	C1
cg05031016	chr14	24334947	24334948	ADCY4	Island	C1
cg07485357	chr14	24334473	24334474	ADCY4	N_Shore	C1
cg12265829	chr14	24334816	24334817	ADCY4	Island	C1
cg13631572	chr14	24334697	24334698	ADCY4	Island	C1
cg14287235	chr14	24335133	24335134	ADCY4	Island	C1
cg16215203	chr14	24334711	24334712	ADCY4	Island	C1
cg16761581	chr14	24334601	24334602	ADCY4	Island	C1
cg19646112	chr14	24335136	24335137	ADCY4	Island	C1
cg23179456	chr14	24334667	24334668	ADCY4	Island	C1
cg25556905	chr14	24334719	24334720	ADCY4	Island	C1
cg20184247	chr17	48594690	48594691	HOXB-AS3	S_Shore	C1
cg26279336	chr17	48597076	48597077	HOXB-AS3	S_Shore	C4
cg01501819	chr19	49072943	49072944	KCNA7	Island	C5
cg00987461	chr12	104050228	104050229	GLT8D2	.	C5
cg14849798	chr12	104050239	104050240	GLT8D2	.	C5
cg16721177	chr12	104050233	104050234	GLT8D2	.	C5
cg02409878	chr8	98948270	98948271	OSR2	Island	C4
cg02469095	chr8	98948693	98948694	OSR2	Island	C4
cg26570179	chr8	98948272	98948273	OSR2	Island	C4
cg20034091	chr18	33578563	33578564	ASXL3	Island	C5

cg24880056	chr18	33578270	33578271	ASXL3	S_Shore	C5
cg27109877	chr18	33578585	33578586	ASXL3	Island	C5
cg00225623	chr13	24271753	24271754	SPATA13	.	C4
cg04579966	chr13	24272083	24272084	SPATA13	.	C4
cg00813746	chr3	50238126	50238127	XXcos-LUCA16.1	Island	C4
cg05060704	chr3	50238262	50238263	XXcos-LUCA16.1	S_Shore	C4
cg12996903	chr3	50238143	50238144	XXcos-LUCA16.1	Island	C4
cg15726260	chr6	30007123	30007124	HLA-J	Island	C5
cg11354594	chr4	183905551	183905552	STOX2	Island	C4
cg27614319	chr17	36722750	36722751	CTD-3194G12.2	.	C4
cg02535060	chr10	132337186	132337187	LRRC27	.	C4
cg05951994	chr10	132337256	132337257	LRRC27	.	C4
cg07119315	chr10	132337063	132337064	LRRC27	.	C4
cg10530568	chr10	132337002	132337003	LRRC27	.	C4
cg21370255	chr10	132336985	132336986	LRRC27	.	C4
cg22586726	chr10	132337038	132337039	LRRC27	.	C4
cg24214699	chr10	132336947	132336948	LRRC27	.	C4
cg11279444	chr1	52633381	52633382	FAM159A	Island	C5
cg18146843	chr1	52633576	52633577	FAM159A	Island	C5
cg25074426	chr14	88551432	88551433	RP11-507K2.3	Island	C4
cg08886727	chr17	48723722	48723723	PRAC2	Island	C5
cg08886727	chr17	48723722	48723723	PRAC1	Island	C5
cg10978355	chr5	81233521	81233522	CKMT2	.	C5
cg12827530	chr5	81233302	81233303	CKMT2	.	C5
cg20929545	chr11	119087336	119087337	HMBS	S_Shelf	C1
cg22027897	chr17	62808495	62808496	Mar-10	Island	C5
cg00700007	chr6	27867585	27867586	HIST1H1B	Island	C5
cg18798248	chr6	27867682	27867683	HIST1H1B	Island	C5
cg03929531	chr15	52112965	52112966	BCL2L10	S_Shore	C4
cg15190747	chr15	52112936	52112937	BCL2L10	S_Shore	C4
cg26631984	chr2	17538108	17538109	VSNL1	N_Shore	C5
cg02000318	chr7	27158094	27158095	HOXA7	N_Shore	C1
cg02384857	chr7	27157995	27157996	HOXA7	N_Shore	C1
cg03232620	chr7	27158260	27158261	HOXA7	N_Shore	C1
cg06911354	chr7	27157836	27157837	HOXA7	N_Shore	C1
cg15372603	chr7	27157620	27157621	HOXA7	S_Shore	C1
cg19147218	chr7	27157936	27157937	HOXA7	N_Shore	C1
cg13694927	chr7	27145093	27145094	HOXA5	Island	C1
cg06888121	chr7	27129449	27129450	HOXA4	N_Shore	C1
cg14042889	chr7	27129589	27129590	HOXA4	N_Shore	C1
cg05250768	chr7	27169651	27169652	HOXA9	Island	C4
cg08964780	chr7	27169844	27169845	HOXA9	Island	C4
cg24884519	chr7	27170171	27170172	HOXA9	S_Shore	C4
cg25644556	chr7	27169963	27169964	HOXA9	Island	C4
cg26259537	chr7	27169628	27169629	HOXA9	Island	C4
cg23679724	chr20	59006423	59006424	CTSZ	N_Shore	C4

cg21819468	chr1	13584074	13584075	PDPN	Island	C5
cg08781140	chr17	75588536	75588537	MYO15B	Island	C6
cg22287064	chr17	75587989	75587990	MYO15B	Island	C6
cg04796763	chr14	21301017	21301018	RPGRIP1	Island	C5
cg05639912	chr17	49993527	49993528	DLX3	Island	C5
cg05129081	chr3	189631147	189631148	TP63	.	C4
cg18881978	chr11	133900918	133900919	MIR4697HG	.	C4
cg23971226	chr11	133900869	133900870	MIR4697HG	.	C4
cg21361094	chr12	57628134	57628135	B4GALNT1	Island	C4
cg00415978	chr4	118888770	118888771	SYNPO2	.	C5
cg02519806	chr4	118888881	118888882	SYNPO2	.	C5
cg02819335	chr4	118888937	118888938	SYNPO2	.	C5
cg05682719	chr4	118888816	118888817	SYNPO2	.	C5
cg11566061	chr4	118888911	118888912	SYNPO2	.	C5
cg24326232	chr4	118888820	118888821	SYNPO2	.	C5
cg06080729	chr16	3017625	3017626	TNFRSF12A	Island	C4
cg04528060	chr4	56505702	56505703	ARL9	Island	C5
cg07800892	chr19	37211233	37211234	CTC-454I21.3	.	C2
cg10879207	chr12	66235616	66235617	RBMS1P1	Island	C5
cg20162652	chr12	66235238	66235239	RBMS1P1	Island	C5
cg08718097	chr1	23342299	23342300	HNRNPR	N_Shore	C1
cg13338827	chr7	87475616	87475617	ABCB4	Island	C5
cg13570585	chr20	8132926	8132927	PLCB1	Island	C4
cg08101193	chr11	6269721	6269722	CCKBR	N_Shore	C4
cg10139651	chr22	30822722	30822723	OSBP2	Island	C5
cg03506033	chr11	75668819	75668820	MAP6	Island	C5
cg10684802	chr11	75668446	75668447	MAP6	Island	C5
cg18935660	chr11	75668822	75668823	MAP6	Island	C5
cg25074426	chr14	88551432	88551433	PTPN21	Island	C4
cg13851466	chr1	37754301	37754302	EPHA10	Island	C5
cg06917617	chr1	207645473	207645474	CD46P1	Island	C6
cg23401796	chr8	140238568	140238569	TRAPPC9	.	C4
cg24129356	chr6	32952958	32952959	XXbac-BPG181M17.5	.	C4
cg20053110	chr6	37650088	37650089	MDGA1	Island	C5
cg20601919	chr2	218782108	218782109	CYP27A1	Island	C5
cg24389730	chr17	48037249	48037250	MIR152	Island	C5
cg23275064	chr2	3702389	3702390	DCDC2C	N_Shore	C4
cg05085336	chr3	73474578	73474579	PDZRN3	.	C4
cg01038149	chr22	21014476	21014477	P2RX6	Island	C5
cg19789653	chr22	21014348	21014349	P2RX6	Island	C5
cg21014483	chr22	21014418	21014419	P2RX6	Island	C5
cg06997549	chr12	2834997	2834998	NRIP2	.	C4
cg14826425	chr7	27193218	27193219	RP1-170O19.14	Island	C1
cg15971010	chr17	19533587	19533588	SLC47A1	Island	C5
cg02970696	chr3	125357528	125357529	ZNF148	Island	C4
cg10794285	chr18	31042058	31042059	DSC3	Island	C7

cg14719951	chr18	31042508	31042509	DSC3	Island	C6
cg06917617	chr1	207645473	207645474	CR1L	Island	C6
cg13050390	chr7	143958558	143958559	OR2F1	.	C7
cg01554060	chr5	115602305	115602306	AC010226.4	Island	C4
cg08870042	chr5	115602451	115602452	AC010226.4	Island	C4
cg18098750	chr5	115601951	115601952	AC010226.4	Island	C4
cg20733436	chr5	115602087	115602088	AC010226.4	Island	C4
cg22173752	chr5	115602222	115602223	AC010226.4	Island	C4
cg00002719	chr1	169427468	169427469	CCDC181	Island	C1
cg08104202	chr1	169427474	169427475	CCDC181	Island	C5
cg25464921	chr1	169460460	169460461	CCDC181	.	C5
cg01085803	chr6	11233312	11233313	NEDD9	.	C4
cg26844213	chr6	11233359	11233360	NEDD9	.	C4
cg15457058	chr1	47416650	47416651	FOXE3	Island	C5
cg18815943	chr1	47416642	47416643	FOXE3	Island	C5
cg19809499	chr1	47416593	47416594	FOXE3	Island	C5
cg20034091	chr18	33578563	33578564	RP11-258B16.1	Island	C5
cg24880056	chr18	33578270	33578271	RP11-258B16.1	S_Shore	C5
cg27109877	chr18	33578585	33578586	RP11-258B16.1	Island	C5
cg26552774	chr15	43800200	43800201	RP11-296A16.1	N_Shore	C4
cg16713947	chr12	6451523	6451524	CD27-AS1	.	C4
cg08081156	chr8	100649748	100649749	SNX31	S_Shore	C5
cg10715223	chr8	100649560	100649561	SNX31	Island	C5
cg13277380	chr8	100649793	100649794	SNX31	S_Shore	C5
cg18582180	chr8	100649763	100649764	SNX31	S_Shore	C5
cg24903183	chr8	100649690	100649691	SNX31	S_Shore	C5
cg26440289	chr8	100649693	100649694	SNX31	S_Shore	C5
cg27043726	chr8	100649539	100649540	SNX31	Island	C5
cg19595886	chr7	57654679	57654680	RP11-368M16.8	.	C2
cg19595886	chr7	57654679	57654680	RP11-368M16.7	.	C2
cg21746532	chr15	24953646	24953647	SNURF	N_Shore	C4
cg05945782	chr17	2051692	2051693	MIR212	Island	C5
cg25479916	chr6	27873614	27873615	HIST1H4L	S_Shore	C5
cg26552774	chr15	43800200	43800201	SERINC4	N_Shore	C4
cg15642759	chr10	70284048	70284049	NPFFR1	Island	C5
cg23609985	chr1	119327716	119327717	RP5-834N19.1	Island	C5
cg05211836	chr19	16888765	16888766	F2RL3	N_Shore	C4
cg23599104	chr1	109113698	109113699	KIAA1324	Island	C5
cg16131766	chr15	23565133	23565134	MKRN3	.	C4
cg26286839	chr5	80083132	80083133	CTD-2201I18.1	.	C7
cg19224164	chr4	2964657	2964658	NOP14	S_Shore	C4
cg23609985	chr1	119327716	119327717	RP11-418J17.3	Island	C5
cg06148736	chr22	18998178	18998179	DGCR5	Island	C4
cg09619624	chr22	18998119	18998120	DGCR5	Island	C4
cg16489547	chr16	30586128	30586129	ZNF785	S_Shore	C4
cg08729318	chr12	29783906	29783907	TMTC1	Island	C5

cg10512875	chr12	29783241	29783242	TMTC1	Island	C5
cg01940297	chr8	128048945	128048946	MIR1207	.	C4
cg22492271	chr7	36919884	36919885	MIR1200	.	C4
cg10453019	chr3	16174708	16174709	GALNT15	.	C1
cg18789887	chr3	16174593	16174594	GALNT15	.	C1
cg23067317	chr3	16174587	16174588	GALNT15	.	C1
cg04022379	chr5	111073042	111073043	TSLP	Island	C5
cg24994173	chr5	111073299	111073300	TSLP	Island	C5
cg04614997	chr17	29006167	29006168	SEZ6	Island	C5
cg24041078	chr11	111513164	111513165	MIR34C	Island	C5
cg25446604	chr2	241932761	241932762	LINC01237	Island	C4
cg01527459	chr10	80356815	80356816	DYDC2	Island	C5
cg01527459	chr10	80356815	80356816	DYDC1	Island	C5
cg14240820	chr17	74262676	74262677	CTD-2514K5.4	.	C4
cg03081691	chr1	226000305	226000306	SDE2	S_Shore	C1
cg04117530	chr1	226000151	226000152	SDE2	S_Shore	C4
cg01348086	chr5	177358295	177358296	RGS14	.	C4
cg04466840	chr5	177357718	177357719	RGS14	.	C4
cg25461508	chr5	177357714	177357715	RGS14	.	C4
cg12351660	chr10	73911035	73911036	PLAU	Island	C5
cg23599104	chr1	109113698	109113699	C1orf194	Island	C5
cg14346208	chr11	35526000	35526001	PAMR1	.	C5
cg24740531	chr18	63318499	63318500	BCL2	Island	C5
cg06641593	chr3	37992744	37992745	VILL	N_Shore	C4
cg23585979	chr4	74365460	74365461	EREG	.	C5
cg07182669	chr6	2903301	2903302	SERPINB9	Island	C5
cg07844977	chr6	2903465	2903466	SERPINB9	Island	C5
cg13722431	chr6	2903299	2903300	SERPINB9	Island	C5
cg22336806	chr6	2903470	2903471	SERPINB9	Island	C5
cg20470734	chr12	129700471	129700472	TMEM132D	S_Shore	C4
cg01597066	chr19	48624249	48624250	AC022154.7	Island	C4
cg02583309	chr19	48624070	48624071	AC022154.7	N_Shore	C4
cg11376305	chr19	48624253	48624254	AC022154.7	Island	C4
cg22325530	chr12	110689541	110689542	HVCN1	Island	C5
cg14780632	chr11	66049050	66049051	GAL3ST3	Island	C1
cg24960947	chr11	66048992	66048993	GAL3ST3	Island	C1
cg25717844	chr7	100168195	100168196	GAL3ST4	N_Shelf	C5
cg10324701	chr12	131493324	131493325	RP11-897M7.1	S_Shore	C4
cg13997645	chr11	15114929	15114930	INSC	Island	C5
cg07664976	chr15	25099341	25099342	SNORD116-26	.	C4
cg16150571	chr15	25089841	25089842	SNORD116-22	.	C4
cg18488946	chr15	24985148	24985149	SNORD64	.	C4
cg26410550	chr15	24984109	24984110	SNORD64	.	C4
cg10082589	chr2	191848194	191848195	SDPR	.	C4
cg14022778	chr1	15327105	15327106	FHAD1	.	C4
cg21576886	chr1	158930298	158930299	PYHIN1	.	C3

cg01910527	chr17	74249439	74249440	TTYH2	.	C4
cg24101049	chr14	51846919	51846920	GNG2	.	C4
cg00243313	chr5	1883299	1883300	CTD-2194D22.3	Island	C5
cg00264591	chr5	1883400	1883401	CTD-2194D22.3	Island	C5
cg07882671	chr5	1882891	1882892	CTD-2194D22.3	Island	C4
cg24876960	chr5	1883100	1883101	CTD-2194D22.3	Island	C4
cg12421755	chr15	52788736	52788737	ONECUT1	Island	C5
cg01627847	chr17	45262145	45262146	SPATA32	Island	C4
cg03048083	chr17	45262130	45262131	SPATA32	Island	C4
cg04992638	chr17	45261961	45261962	SPATA32	Island	C4
cg08124910	chr17	45262148	45262149	SPATA32	Island	C4
cg08247376	chr17	45261991	45261992	SPATA32	Island	C4
cg11933779	chr17	45261673	45261674	SPATA32	N_Shore	C4
cg13001868	chr17	45261856	45261857	SPATA32	Island	C4
cg17222164	chr17	45262109	45262110	SPATA32	Island	C4
cg19539667	chr17	45262464	45262465	SPATA32	Island	C4
cg24542751	chr17	45262222	45262223	SPATA32	Island	C4
cg26532627	chr17	45262377	45262378	SPATA32	Island	C4
cg26742995	chr17	45262227	45262228	SPATA32	Island	C4
cg09838956	chr15	25080566	25080567	SNHG14	.	C4
cg26582085	chr15	24980782	24980783	SNHG14	.	C4
cg25035631	chr1	35120757	35120758	EFCAB14P1	Island	C5
cg24550644	chr17	32519186	32519187	RP11-466A19.7	Island	C4
cg00531137	chr16	57610020	57610021	GPR56	.	C4
cg08739433	chr1	112509303	112509304	WNT2B	Island	C5
cg11806528	chr1	112509355	112509356	WNT2B	Island	C7
cg13939859	chr1	90717962	90717963	BARHL2	Island	C4
cg20311863	chr1	90718569	90718570	BARHL2	Island	C4
cg08864344	chr1	33307396	33307397	RP11-415J8.3	S_Shore	C1
cg20384898	chr6	1624876	1624877	GMDS	Island	C1
cg21347053	chr6	1624743	1624744	GMDS	Island	C1
cg24079361	chr13	44373903	44373904	SERP2	Island	C5
cg16410706	chr1	212700621	212700622	BATF3	Island	C5
cg25479916	chr6	27873614	27873615	HIST1H3I	S_Shore	C5
cg22507887	chr1	157925710	157925711	RP11-451O13.1	.	C4
cg24716879	chr1	234970125	234970126	RP11-443B7.1	.	C7
cg06976589	chr4	1310260	1310261	MAEA	S_Shore	C1
cg06722639	chr14	23155168	23155169	SLC7A8	.	C4
cg18701685	chr14	23155154	23155155	SLC7A8	.	C4
cg26582085	chr15	24980782	24980783	SNORD107	.	C4
cg04110544	chr5	42424840	42424841	GHR	Island	C5
cg07229186	chr16	56632663	56632664	MT1M	Island	C5
cg07602841	chr16	56668232	56668233	MT1G	N_Shore	C5
cg23039189	chr11	7512797	7512798	PPFIBP2	N_Shore	C4
cg19507527	chr3	192727518	192727519	FGF12	.	C4
cg18488946	chr15	24985148	24985149	RP11-701H24.5	.	C4

cg26410550	chr15	24984109	24984110	RP11-701H24.5	.	C4
cg01059295	chr4	188631523	188631524	RP11-756P10.1	Island	C4
cg10810752	chr1	47225488	47225489	RP1-18D14.7	Island	C5
cg22381196	chr16	72007477	72007478	DHODH	N_Shore	C4
cg05413277	chr2	74414960	74414961	AC005041.11	Island	C4
cg01554060	chr5	115602305	115602306	TICAM2	Island	C4
cg08870042	chr5	115602451	115602452	TICAM2	Island	C4
cg18098750	chr5	115601951	115601952	TICAM2	Island	C4
cg20733436	chr5	115602087	115602088	TICAM2	Island	C4
cg22173752	chr5	115602222	115602223	TICAM2	Island	C4
cg00994629	chr14	22226652	22226653	TRAV36DV7	.	C4
cg00356183	chr7	712196	712197	PRKAR1B	Island	C1
cg12441126	chr7	712325	712326	PRKAR1B	Island	C1
cg27331241	chr7	712193	712194	PRKAR1B	Island	C1
cg24129356	chr6	32952958	32952959	HLA-DMA	.	C4
cg14159026	chr6	105136676	105136677	BVES-AS1	Island	C5
cg08095637	chr3	169812739	169812740	LRRC34	S_Shore	C4
cg12358524	chr3	169812679	169812680	LRRC34	S_Shore	C4
cg12397426	chr3	169812646	169812647	LRRC34	Island	C4
cg27536453	chr3	169812629	169812630	LRRC34	Island	C4
cg17102205	chr2	207227237	207227238	AC007879.4	.	C4
cg24550644	chr17	32519186	32519187	RP11-466A19.6	Island	C4
cg24550644	chr17	32519186	32519187	RP11-466A19.3	Island	C4
cg09374838	chr7	149881295	149881296	ATP6V0E2-AS1	.	C4
cg27195224	chr22	32457160	32457161	BPIFC	.	C4
cg08196842	chr14	64549799	64549800	PPP1R36	Island	C5
cg12281657	chr14	64549877	64549878	PPP1R36	Island	C5
cg17930034	chr14	64549884	64549885	PPP1R36	Island	C5
cg23265500	chr14	64549873	64549874	PPP1R36	Island	C5
cg15841063	chr1	63323829	63323830	FOXD3-AS1	Island	C4
cg08911275	chr2	176092734	176092735	HOXD13	Island	C4
cg15128200	chr1	107140052	107140053	NTNG1	N_Shore	C5
cg13866214	chr18	12304804	12304805	RNU6-170P	N_Shelf	C4
cg01826863	chr2	47570814	47570815	KCNK12	Island	C5
cg04981611	chr2	47571338	47571339	KCNK12	Island	C5
cg13442432	chr11	43579229	43579230	HSD17B12	Island	C5
cg23589617	chr11	43579178	43579179	HSD17B12	Island	C5
cg07068756	chr4	41256893	41256894	UCHL1	Island	C6
cg27494647	chr7	150341809	150341810	RARRES2	S_Shore	C5
cg01038149	chr22	21014476	21014477	TUBA3FP	Island	C5
cg19789653	chr22	21014348	21014349	TUBA3FP	Island	C5
cg21014483	chr22	21014418	21014419	TUBA3FP	Island	C5
cg01356198	chr3	188211725	188211726	LPP	.	C4
cg16754467	chr6	10426355	10426356	RP1-290I10.3	Island	C4
cg10273340	chr16	56190881	56190882	GNAO1	Island	C5
cg04100696	chr11	12008721	12008722	DKK3	Island	C5

cg22573528	chr2	51027032	51027033	AC007682.4	N_Shore	C4
cg17689707	chr6	134317733	134317734	SGK1	Island	C5
cg16489586	chr14	50561246	50561247	MAP4K5	S_Shore	C5
cg21107197	chr14	50561143	50561144	MAP4K5	S_Shore	C5
cg09838956	chr15	25080566	25080567	SNORD116-15	.	C4
cg03665761	chr16	54932017	54932018	IRX5	Island	C4
cg16642791	chr16	54932559	54932560	IRX5	Island	C4
cg00243313	chr5	1883299	1883300	IRX4	Island	C5
cg00264591	chr5	1883400	1883401	IRX4	Island	C5
cg07882671	chr5	1882891	1882892	IRX4	Island	C4
cg24876960	chr5	1883100	1883101	IRX4	Island	C4
cg08881680	chr11	95232305	95232306	SESN3	Island	C5
cg07748159	chr11	83286867	83286868	CCDC90B	S_Shore	C4
cg01791407	chr2	74002697	74002698	TET3	Island	C4
cg19527084	chr2	74002648	74002649	TET3	Island	C4
cg23793040	chr2	74002868	74002869	TET3	Island	C4
cg15726260	chr6	30007123	30007124	HCG4P3	Island	C5
cg20941820	chr15	100342129	100342130	ADAMTS17	Island	C5
cg02927327	chr6	35297982	35297983	DEF6	.	C4
cg03355690	chr6	35297839	35297840	DEF6	.	C4
cg05583848	chr6	35297634	35297635	DEF6	.	C4
cg07294541	chr6	35297778	35297779	DEF6	.	C4
cg19403104	chr19	49527989	49527990	RCN3	Island	C7
cg19808978	chr19	49528016	49528017	RCN3	S_Shore	C7
cg17635080	chr12	108636339	108636340	RP11-689B22.2	.	C4
cg24398450	chr12	108635323	108635324	RP11-689B22.2	.	C4
cg04915044	chr2	86036772	86036773	POLR1A	.	C4
cg26279336	chr17	48597076	48597077	HOXB6	S_Shore	C4
cg20184247	chr17	48594690	48594691	HOXB5	S_Shore	C1
cg22156128	chr3	10285769	10285770	GHRLOS	.	C1
cg06500727	chr2	1413392	1413393	TPO	.	C4
cg12680131	chr2	1413381	1413382	TPO	.	C4
cg09374838	chr7	149881295	149881296	RP11-445N20.2	.	C4
cg23817893	chr11	86374890	86374891	CCDC81	Island	C7
cg27377289	chr7	7772642	7772643	UMAD1	.	C6
cg18120358	chr15	25062275	25062276	SNORD116-5	.	C4
cg18154158	chr15	25062022	25062023	SNORD116-5	.	C4
cg24738356	chr15	25062337	25062338	SNORD116-5	.	C4
cg02029242	chr15	25067014	25067015	SNORD116-7	.	C4
cg01350077	chr6	41638579	41638580	MDFI	Island	C1
cg27200446	chr6	41638701	41638702	MDFI	Island	C5
cg05021846	chr4	48483276	48483277	SLC10A4	N_Shore	C5
cg19065831	chr4	48483272	48483273	SLC10A4	N_Shore	C5
cg19247841	chr4	48483284	48483285	SLC10A4	N_Shore	C5
cg05497345	chr2	96325176	96325177	ITPRIPL1	Island	C5
cg08983217	chr2	96325411	96325412	ITPRIPL1	Island	C5

cg11270393	chr2	96325279	96325280	ITPRIPL1	Island	C5
cg13997864	chr2	96325244	96325245	ITPRIPL1	Island	C5
cg04517429	chr4	154492466	154492467	DCHS2	S_Shore	C5
cg06888121	chr7	27129449	27129450	HOXA-AS3	N_Shore	C1
cg14042889	chr7	27129589	27129590	HOXA-AS3	N_Shore	C1
cg01175550	chr7	27107587	27107588	HOXA-AS2	N_Shore	C1
cg01957751	chr8	142280225	142280226	TSNARE1	Island	C4
cg00393798	chr11	8594147	8594148	STK33	Island	C5
cg11596902	chr11	123454126	123454127	LINC01059	.	C4
cg16823292	chr1	71007055	71007056	RP3-333A15.2	.	C4
cg14638883	chr6	166168713	166168714	T	Island	C5
cg17188046	chr6	166168709	166168710	T	Island	C5
cg19675288	chr6	166168718	166168719	T	Island	C5
cg09975039	chr9	129320678	129320679	C9orf106	Island	C4
cg23363971	chr12	56021487	56021488	IKZF4	.	C4
cg26607758	chr8	90985325	90985326	C8orf88	Island	C1
cg04672495	chr15	75844534	75844535	UBE2Q2	S_Shore	C5
cg05044743	chr17	47852291	47852292	SP6	S_Shore	C6
cg09965384	chr17	47852317	47852318	SP6	S_Shore	C4
cg01597066	chr19	48624249	48624250	SPHK2	Island	C4
cg02583309	chr19	48624070	48624071	SPHK2	N_Shore	C4
cg11376305	chr19	48624253	48624254	SPHK2	Island	C4
cg22573528	chr2	51027032	51027033	NRXN1	N_Shore	C4
cg07983905	chr20	3092915	3092916	RN7SL555P	Island	C6
cg16489547	chr16	30586128	30586129	RP11-146F11.5	S_Shore	C4
cg07826859	chr7	44980487	44980488	MYO1G	.	C4
cg17558126	chr1	206506917	206506918	RASSF5	Island	C5
cg25706502	chr1	3242644	3242645	PRDM16	S_Shore	C4
cg11970289	chr12	122204422	122204423	B3GNT4	Island	C5
cg23445859	chr12	122204240	122204241	B3GNT4	Island	C1
cg16849024	chr19	41428305	41428306	B3GNT8	.	C4
cg20557104	chr19	41428408	41428409	B3GNT8	.	C4
cg15242360	chr6	39886754	39886755	DAAM2	.	C4
cg02000318	chr7	27158094	27158095	RP1-170O19.24	N_Shore	C1
cg02384857	chr7	27157995	27157996	RP1-170O19.24	N_Shore	C1
cg03232620	chr7	27158260	27158261	RP1-170O19.24	N_Shore	C1
cg06911354	chr7	27157836	27157837	RP1-170O19.24	N_Shore	C1
cg15372603	chr7	27157620	27157621	RP1-170O19.24	S_Shore	C1
cg19147218	chr7	27157936	27157937	RP1-170O19.24	N_Shore	C1
cg04283162	chr15	29570313	29570314	FAM189A1	Island	C5
cg26552774	chr15	43800200	43800201	HYPK	N_Shore	C4
cg01262913	chr21	37208185	37208186	DSCR9	.	C4
cg11399508	chr21	37208183	37208184	DSCR9	.	C4
cg27372920	chr5	53482915	53482916	FST	S_Shore	C5
cg23273694	chr7	151440076	151440077	CRYGN	Island	C5
cg05861291	chr2	207122814	207122815	KLF7-IT1	.	C4

cg20633478	chr10	129768399	129768400	RP11-109A6.3	.	C4
cg24389730	chr17	48037249	48037250	COPZ2	Island	C5
cg13050981	chr17	9576648	9576649	STX8	S_Shore	C4
cg23980468	chr17	9576622	9576623	STX8	S_Shore	C4
cg01815671	chr3	126394820	126394821	CCDC37	Island	C5
cg13567541	chr7	129188462	129188463	SMO	Island	C5
cg01659099	chr15	40405843	40405844	IVD	.	C4
cg02378269	chr15	40405390	40405391	IVD	.	C4
cg06731484	chr15	40405513	40405514	IVD	.	C4
cg09655862	chr15	40405433	40405434	IVD	.	C4
cg11934304	chr15	40406138	40406139	IVD	.	C4
cg13463731	chr15	40406166	40406167	IVD	.	C4
cg26738606	chr15	40406191	40406192	IVD	.	C4
cg27148665	chr15	40405581	40405582	IVD	.	C4
cg27529930	chr15	40405758	40405759	IVD	.	C4
cg14539231	chr13	42992126	42992127	EPSTI1	Island	C5
cg03770912	chr17	48817312	48817313	TTLL6	Island	C5
cg05257472	chr17	48817294	48817295	TTLL6	Island	C5
cg06830702	chr17	48817124	48817125	TTLL6	Island	C5
cg07528363	chr17	48817101	48817102	TTLL6	Island	C5
cg12027420	chr17	48817322	48817323	TTLL6	Island	C5
cg13342722	chr17	48817128	48817129	TTLL6	Island	C5
cg22330492	chr17	48817103	48817104	TTLL6	Island	C5
cg25120284	chr17	48817309	48817310	TTLL6	Island	C5
cg19870284	chr1	175044901	175044902	SDCCAG3P2	.	C4
cg14383422	chr2	200586239	200586240	AOX1	Island	C5
cg05361096	chr12	49955524	49955525	RP11-469H8.8	Island	C4
cg06734406	chr12	49955546	49955547	RP11-469H8.8	Island	C4
cg09577511	chr12	49955390	49955391	RP11-469H8.8	Island	C4
cg02595219	chr11	74467716	74467717	KCNE3	Island	C5
cg11775521	chr11	74467750	74467751	KCNE3	Island	C5
cg05927190	chr1	53737875	53737876	GLIS1	Island	C5
cg18949300	chr6	166862290	166862291	RPS6KA2	Island	C5
cg21760146	chr6	166862293	166862294	RPS6KA2	Island	C5
cg08881680	chr11	95232305	95232306	RP11-712B9.2	Island	C5
cg12778476	chr11	22337799	22337800	CTD-2140G10.2	N_Shelf	C5
cg03656968	chr19	55063057	55063058	RDH13	Island	C4
cg18372896	chr14	75430673	75430674	JDP2	S_Shelf	C1
cg17567560	chr10	103277106	103277107	INA	Island	C4
cg24680586	chr10	103276970	103276971	INA	Island	C4
cg22381196	chr16	72007477	72007478	ATP5A1P3	N_Shore	C4
cg04377289	chr17	81018165	81018166	CTD-2561B21.11	.	C4
cg09022720	chr1	92483890	92483891	GFI1	Island	C5
cg11785652	chr1	92483780	92483781	GFI1	Island	C5
cg22628500	chr1	92483895	92483896	GFI1	Island	C5
cg16017089	chr11	73342785	73342786	ARHGEF17	Island	C5

cg01030534	chr7	143882605	143882606	TCAF1	N_Shelf	C5
cg03514404	chr7	143882572	143882573	TCAF1	N_Shelf	C5
cg12749863	chr21	25562264	25562265	MIR155HG	Island	C5
cg14315558	chr21	25562370	25562371	MIR155HG	Island	C5
cg17297071	chr21	25562112	25562113	MIR155HG	Island	C5
cg16818414	chr22	45503086	45503087	FBLN1	Island	C5
cg10810752	chr1	47225488	47225489	TAL1	Island	C5
cg15694117	chr19	2717424	2717425	DIRAS1	Island	C4
cg08886727	chr17	48723722	48723723	HOXB13-AS1_2	Island	C5
cg09937039	chr14	75522860	75522861	BATF	.	C4
cg14424070	chr14	75522477	75522478	BATF	.	C4
cg15645309	chr14	75522404	75522405	BATF	.	C4
cg21531300	chr14	75522457	75522458	BATF	.	C4
cg05799507	chr16	56643311	56643312	MT1DP	Island	C5
cg05003322	chr1	31704105	31704106	COL16A1	Island	C4
cg24577417	chr6	167977879	167977880	RP3-470B24.5	N_Shore	C4
cg07702750	chr7	28958598	28958599	TRIL	Island	C5
cg19929126	chr7	28958579	28958580	TRIL	Island	C5
cg10348922	chr22	20965015	20965016	AIFM3	Island	C4
cg16590005	chr22	20964957	20964958	AIFM3	Island	C4
cg24848890	chr3	9915783	9915784	IL17RC	Island	C4
cg02249490	chr17	43927510	43927511	RP11-527L4.5	.	C5
cg02249490	chr17	43927510	43927511	RP11-527L4.6	.	C5
cg24547885	chr2	179050141	179050142	CCDC141	.	C1
cg01181415	chr12	16605020	16605021	LMO3	.	C4
cg01787574	chr12	16605123	16605124	LMO3	.	C4
cg10143811	chr12	16605051	16605052	LMO3	.	C4
cg02884176	chr2	88452701	88452702	FOXI3	Island	C5
cg08871855	chr2	88452767	88452768	FOXI3	Island	C5
cg19544662	chr2	88452538	88452539	FOXI3	Island	C5
cg01815671	chr3	126394820	126394821	CCDC37-AS1	Island	C5
cg08432727	chr2	5691590	5691591	LINC01248	Island	C5
cg08526991	chr2	5691262	5691263	LINC01248	Island	C1
cg26827247	chr11	93330042	93330043	CCDC67	N_Shore	C5
cg10037049	chr12	47882850	47882851	VDR	.	C4
cg21844669	chr12	119333330	119333331	CCDC60	N_Shore	C4
cg16713947	chr12	6451523	6451524	TAPBPL	.	C4
cg22256433	chr17	8039425	8039426	ALOX15B	.	C4
cg06305609	chr19	17523363	17523364	FAM129C	S_Shelf	C4
cg17942851	chr1	36320684	36320685	SH3D21	N_Shore	C4
cg19969694	chr15	40893602	40893603	VPS18	N_Shore	C1
cg08307816	chr14	100159855	100159856	DEGS2	Island	C5
cg07028533	chr7	146116347	146116348	CNTNAP2	Island	C5
cg08432727	chr2	5691590	5691591	SOX11	Island	C5
cg08526991	chr2	5691262	5691263	SOX11	Island	C1
cg17246140	chr2	42793124	42793125	HAAO	Island	C5

cg16306898	chr1	1540295	1540296	TMEM240	Island	C1
cg16601494	chr1	1540357	1540358	TMEM240	Island	C1
cg06824394	chr7	87217219	87217220	TMEM243	N_Shelf	C4
cg23732024	chr8	73991566	73991567	LY96	.	C4
cg02288341	chr5	138274537	138274538	GFRA3	Island	C5
cg09350274	chr5	138274707	138274708	GFRA3	Island	C5
cg18442524	chr5	138274683	138274684	GFRA3	Island	C5
cg19670286	chr5	138274438	138274439	GFRA3	Island	C5
cg21225170	chr5	138274702	138274703	GFRA3	Island	C5
cg23511432	chr5	138274566	138274567	GFRA3	Island	C5
cg04299389	chr20	25148660	25148661	RP4-738P15.1	Island	C5
cg05446860	chr20	25148490	25148491	RP4-738P15.1	Island	C5
cg05235884	chr6	30164029	30164030	TRIM15	.	C5
cg23778358	chr6	30163972	30163973	TRIM15	.	C1
cg11253514	chr1	228416539	228416540	TRIM17	Island	C5
cg23540518	chr1	228416865	228416866	TRIM17	Island	C5
cg19217130	chr2	88128062	88128063	FABP1	.	C4
cg14159026	chr6	105136676	105136677	BVES	Island	C5
cg08432727	chr2	5691590	5691591	AC108025.2	Island	C5
cg08526991	chr2	5691262	5691263	AC108025.2	Island	C1
cg10428938	chr7	99393214	99393215	ARPC1B	Island	C1
cg01056889	chr3	27369539	27369540	NEK10	Island	C5
cg05435286	chr3	27369572	27369573	NEK10	Island	C5
cg17191919	chr3	27369136	27369137	NEK10	Island	C5
cg22415969	chr3	27369574	27369575	NEK10	Island	C5
cg10508349	chr16	68908328	68908329	TANGO6	.	C1
cg07478122	chr15	24985274	24985275	PWAR5	.	C4
cg18488946	chr15	24985148	24985149	PWAR5	.	C4
cg26410550	chr15	24984109	24984110	PWAR5	.	C4
cg01481989	chr2	182866441	182866442	FRZB	Island	C4
cg04037038	chr2	182867334	182867335	FRZB	Island	C5
cg06830784	chr2	182867096	182867097	FRZB	Island	C5
cg20701689	chr2	182867104	182867105	FRZB	Island	C5
cg20474425	chr12	54352283	54352284	RP11-753H16.3	.	C4
cg06299833	chr10	124448140	124448141	RP13-238F13.3	Island	C4
cg21401879	chr2	44934897	44934898	Six3os1_3	Island	C1
cg21401879	chr2	44934897	44934898	Six3os1_4	Island	C1
cg02818811	chr12	75334678	75334679	RP11-585P4.6	.	C4
cg07684809	chr12	75334432	75334433	RP11-585P4.6	.	C4
cg19430489	chr12	75334324	75334325	RP11-585P4.6	S_Shelf	C4
cg21087137	chr12	75334689	75334690	RP11-585P4.6	.	C4
cg24234651	chr12	75334691	75334692	RP11-585P4.6	.	C4
cg24353213	chr1	89843061	89843062	LRRC8D	N_Shore	C4
cg22027897	chr17	62808495	62808496	RP11-453A12.1	Island	C5
cg12296552	chr3	11017157	11017158	SLC6A1	Island	C4
cg21611775	chr3	11017150	11017151	SLC6A1	Island	C4

cg05184938	chr17	77373857	77373858		Sep-09	Island	C4
cg06848185	chr17	77372820	77372821		Sep-09	Island	C4
cg12783819	chr17	77373575	77373576		Sep-09	Island	C4
cg19554255	chr17	77372969	77372970		Sep-09	Island	C4
cg20275528	chr17	77373402	77373403		Sep-09	Island	C4
cg20955894	chr17	77451573	77451574		Sep-09	Island	C4
cg21292033	chr17	77466107	77466108		Sep-09	.	C1
cg00174845	chr18	78796804	78796805	RP11-715C4.1		Island	C4
cg16576694	chr14	105605478	105605479	RP11-731F5.1		.	C4
cg24398450	chr12	108635323	108635324	SELPLG		.	C4
cg18261462	chr17	48430343	48430344	SKAP1		S_Shore	C5
cg19585103	chr17	48430187	48430188	SKAP1		Island	C5
cg02083559	chr12	23950424	23950425	SOX5		.	C5
cg26067203	chr1	2003850	2003851	CFAP74		.	C5
cg13296579	chr11	133065067	133065068	AP004782.1		.	C4
cg20417723	chr11	133065225	133065226	AP004782.1		.	C4
cg15571373	chr15	25072199	25072200	SNORD116-9		.	C4
cg25114752	chr15	25070236	25070237	SNORD116-8		.	C4
cg16601489	chr15	25059327	25059328	SNORD116-4		.	C4
cg02534163	chr8	119638754	119638755	ENPP2		.	C5
cg06998282	chr8	119639158	119639159	ENPP2		.	C5
cg15841063	chr1	63323829	63323830	FOXD3		Island	C4
cg07201835	chr12	32678435	32678436	DNM1L		N_Shore	C4
cg20829550	chr12	92735671	92735672	PLEKHG7		.	C4
cg04900427	chr1	43147896	43147897	FAM183A		Island	C4
cg05125578	chr1	43147894	43147895	FAM183A		Island	C4
cg15961211	chr1	43147769	43147770	FAM183A		Island	C4
cg15997906	chr1	43147762	43147763	FAM183A		Island	C4
cg22608128	chr1	43147823	43147824	FAM183A		Island	C4
cg16522557	chr13	110506764	110506765	COL4A2		.	C4
cg23027583	chr1	220879216	220879217	HLX		Island	C5
cg10054857	chr18	64149309	64149310	LINC00305		.	C4
cg14280533	chr14	73239819	73239820	PAPLN		Island	C4
cg22638491	chr14	73239968	73239969	PAPLN		Island	C4
cg23425316	chr14	73239741	73239742	PAPLN		Island	C4
cg05862007	chr2	48723014	48723015	LHCGR		.	C4
cg01431908	chr1	46203210	46203211	LURAP1		Island	C4
cg06787764	chr1	46203198	46203199	LURAP1		Island	C4
cg08160980	chr1	46203168	46203169	LURAP1		Island	C4
cg15696662	chr1	46203549	46203550	LURAP1		Island	C4
cg23431851	chr1	46203150	46203151	LURAP1		Island	C4
cg14145801	chr2	186848984	186848985	ZSWIM2		N_Shore	C5
cg22289434	chr20	35601018	35601019	FER1L4		Island	C1
cg13702222	chr3	152299451	152299452	MBNL1		.	C4
cg16802592	chr15	36887402	36887403	RP11-122D10.1		Island	C5
cg20034372	chr15	36887409	36887410	RP11-122D10.1		Island	C5

cg17504394	chr17	72343154	72343155	RP11-1124B17.1	.	C1
cg03004714	chr18	61893132	61893133	RNF152	N_Shore	C5
cg11870261	chr3	36945188	36945189	TRANK1	Island	C5
cg07706558	chr11	12161899	12161900	MICAL2	.	C1
cg23584743	chr4	13544533	13544534	NKX3-2	Island	C5
cg27438798	chr4	13544527	13544528	NKX3-2	Island	C5
cg01029638	chr7	120988820	120988821	CPED1	.	C5
cg21746532	chr15	24953646	24953647	SNRPN	N_Shore	C4
cg02399524	chr11	63491037	63491038	HRASLS5	Island	C5
cg07213036	chr13	27968590	27968591	CDX2	Island	C4
cg16201038	chr13	27970623	27970624	CDX2	Island	C7
cg00813746	chr3	50238126	50238127	GNAI2	Island	C4
cg05060704	chr3	50238262	50238263	GNAI2	S_Shore	C4
cg06508783	chr3	50238014	50238015	GNAI2	Island	C4
cg12691534	chr3	50237962	50237963	GNAI2	Island	C4
cg12996903	chr3	50238143	50238144	GNAI2	Island	C4
cg20918957	chr3	50237680	50237681	GNAI2	N_Shore	C4
cg01926269	chr2	25168801	25168802	POMC	Island	C5
cg24425171	chr2	25168842	25168843	POMC	Island	C5
cg00731944	chr17	6080773	6080774	WSCD1	.	C4
cg27201457	chr17	6080971	6080972	WSCD1	.	C4
cg05766064	chr18	21682615	21682616	MIR320C1	.	C4
cg11314310	chr10	1074891	1074892	WDR37	.	C4
cg27201679	chr10	1074854	1074855	WDR37	.	C4
cg10282491	chr16	3012367	3012368	CLDN9	N_Shore	C4
cg06080729	chr16	3017625	3017626	CLDN6	Island	C4
cg09275980	chr15	25105778	25105779	SNORD116-29	.	C4
cg10109500	chr3	172448094	172448095	GHSR	Island	C7
cg19785066	chr20	46118016	46118017	CD40	N_Shore	C7
cg17202086	chr11	77411794	77411795	PAK1	Island	C4
cg18309286	chr11	77412002	77412003	PAK1	Island	C4
cg26996201	chr11	77411819	77411820	PAK1	Island	C4
cg10273340	chr16	56190881	56190882	RP11-461O7.1	Island	C5
cg18722881	chr11	22666593	22666594	GAS2	N_Shore	C5
cg01047631	chr16	54938661	54938662	CTD-3032H12.1	Island	C4
cg06892501	chr11	5323875	5323876	OR51B2	.	C4
cg23039189	chr11	7512797	7512798	CTD-2516F10.2	N_Shore	C4
cg02748089	chr16	78506275	78506276	RP11-264L1.4	.	C4
cg05368724	chr2	96895932	96895933	FAM178B	.	C7
cg00836482	chr3	119810109	119810110	NR1I2	Island	C3
cg21041775	chr12	54031634	54031635	HOXC5	S_Shore	C7
cg00506343	chr12	54027249	54027250	HOXC6	N_Shelf	C1
cg05412137	chr12	53990960	53990961	HOXC6	N_Shelf	C4
cg11930554	chr4	73944195	73944196	CXCL1P	Island	C5
cg24591969	chr1	6045799	6045800	KCNAB2	.	C4
cg01366985	chr6	25167467	25167468	CMAHP	.	C4

cg01627847	chr17	45262145	45262146	MAP3K14-AS1	Island	C4
cg03048083	chr17	45262130	45262131	MAP3K14-AS1	Island	C4
cg04992638	chr17	45261961	45261962	MAP3K14-AS1	Island	C4
cg08124910	chr17	45262148	45262149	MAP3K14-AS1	Island	C4
cg08247376	chr17	45261991	45261992	MAP3K14-AS1	Island	C4
cg11933779	chr17	45261673	45261674	MAP3K14-AS1	N_Shore	C4
cg13001868	chr17	45261856	45261857	MAP3K14-AS1	Island	C4
cg17222164	chr17	45262109	45262110	MAP3K14-AS1	Island	C4
cg19539667	chr17	45262464	45262465	MAP3K14-AS1	Island	C4
cg24542751	chr17	45262222	45262223	MAP3K14-AS1	Island	C4
cg26532627	chr17	45262377	45262378	MAP3K14-AS1	Island	C4
cg26742995	chr17	45262227	45262228	MAP3K14-AS1	Island	C4
cg27380724	chr19	55304731	55304732	BRSK1	Island	C4
cg16212145	chr14	24634524	24634525	GZMB	.	C4
cg09636214	chr14	73245783	73245784	RP4-647C14.2	Island	C6
cg14183329	chr7	131558203	131558204	PODXL	Island	C5
cg07371589	chr1	151131891	151131892	RP11-68I18.10	Island	C5
cg16399136	chr1	151131527	151131528	RP11-68I18.10	Island	C5
cg17161421	chr6	32152300	32152301	PRRT1	S_Shore	C1
cg21037008	chr6	32152162	32152163	PRRT1	S_Shore	C1
cg11391462	chr1	103570439	103570440	AMY2B	.	C4
cg08863777	chr11	94545291	94545292	PIWIL4	Island	C4
cg13300301	chr11	94545902	94545903	PIWIL4	S_Shore	C4
cg18023065	chr11	94545437	94545438	PIWIL4	Island	C4
cg05250768	chr7	27169651	27169652	MIR196B	Island	C4
cg08964780	chr7	27169844	27169845	MIR196B	Island	C4
cg24884519	chr7	27170171	27170172	MIR196B	S_Shore	C4
cg25644556	chr7	27169963	27169964	MIR196B	Island	C4
cg26259537	chr7	27169628	27169629	MIR196B	Island	C4
cg11948456	chr2	178413605	178413606	AC009948.5	Island	C5
cg21693907	chr1	203625933	203625934	ATP2B4	N_Shelf	C4
cg10020934	chr17	66877476	66877477	CACNG5	.	C4
cg02818811	chr12	75334678	75334679	GLIPR1L1	.	C4
cg07684809	chr12	75334432	75334433	GLIPR1L1	.	C4
cg19430489	chr12	75334324	75334325	GLIPR1L1	S_Shelf	C4
cg21087137	chr12	75334689	75334690	GLIPR1L1	.	C4
cg24234651	chr12	75334691	75334692	GLIPR1L1	.	C4
cg10296301	chr6	163193482	163193483	RP1-301L19.1	.	C4
cg01733284	chr1	158421329	158421330	OR10K2	.	C7
cg05250768	chr7	27169651	27169652	HOXA10-AS	Island	C4
cg08964780	chr7	27169844	27169845	HOXA10-AS	Island	C4
cg26259537	chr7	27169628	27169629	HOXA10-AS	Island	C4
cg03905819	chr12	53991491	53991492	MIR196A2	N_Shelf	C4
cg05412137	chr12	53990960	53990961	MIR196A2	N_Shelf	C4
cg27494647	chr7	150341809	150341810	RP4-584D14.7	S_Shore	C5
cg01698298	chr11	61295492	61295493	VWCE	Island	C5

cg12110327	chr10	37975653	37975654	RP11-393J16.4	N_Shore	C4
cg17695512	chr11	55967983	55967984	OR10AG1	.	C4
cg03781098	chr12	7695752	7695753	GDF3	.	C4
cg18552861	chr2	20666085	20666086	GDF7	Island	C5
cg10037049	chr12	47882850	47882851	RP11-89H19.1	.	C4
cg23044884	chr8	30387629	30387630	RBPMS	S_Shelf	C7
cg13050981	chr17	9576648	9576649	CFAP52	S_Shore	C4
cg23980468	chr17	9576622	9576623	CFAP52	S_Shore	C4
cg14798020	chr15	25075020	25075021	SNORD116-11	.	C4
cg21844656	chr15	25078648	25078649	SNORD116-13	.	C4
cg01620672	chr15	25076981	25076982	SNORD116-12	.	C4
cg21844656	chr15	25078648	25078649	SNORD116-14	.	C4
cg03942855	chr1	109668303	109668304	GSTM2	Island	C5
cg07234865	chr1	156420428	156420429	C1orf61	N_Shore	C5
cg14862722	chr3	2866595	2866596	CNTN4	.	C4
cg14328457	chr17	39225222	39225223	STAC2	Island	C6
cg15742848	chr2	168912991	168912992	SPC25	.	C5
cg07234865	chr1	156420428	156420429	MIR9-1	N_Shore	C5
cg05335944	chr10	13707010	13707011	FRMD4A	.	C1
cg16674492	chr3	128492843	128492844	GATA2	Island	C1
cg01916115	chr6	128883194	128883195	LAMA2	.	C5
cg19966212	chr5	150413220	150413221	CD74	.	C4
cg24874612	chr17	4786470	4786471	RP11-81A22.4	S_Shore	C4
cg15377585	chr7	19773456	19773457	TMEM196	.	C5
cg05951609	chr4	76435354	76435355	SHROOM3	.	C4
cg13050390	chr7	143958558	143958559	RP4-669B10.3	.	C7
cg10454568	chr14	96424295	96424296	PEBP1P1	Island	C4
cg07068756	chr4	41256893	41256894	UCHL1-AS1	Island	C6
cg11168687	chr4	184805628	184805629	ACSL1	S_Shelf	C4
cg07800892	chr19	37211233	37211234	ZNF585B	.	C2
cg01950845	chr15	93089501	93089502	RGMA	Island	C5
cg02918577	chr15	93089916	93089917	RGMA	Island	C6
cg10959198	chr15	93089667	93089668	RGMA	Island	C5
cg12595281	chr15	93089943	93089944	RGMA	Island	C5
cg09168604	chr10	86367431	86367432	GRID1	Island	C5
cg11832404	chr12	64824478	64824479	TBC1D30	Island	C4
cg23684878	chr12	64824480	64824481	TBC1D30	Island	C4
cg14080475	chr9	114388215	114388216	AKNA	.	C4
cg04822330	chr6	125748026	125748027	HEY2	Island	C5
cg00679711	chr17	3813199	3813200	C17orf85	.	C4
cg02317397	chr17	3812985	3812986	C17orf85	.	C4
cg25382652	chr17	3813132	3813133	C17orf85	.	C4
cg06148736	chr22	18998178	18998179	AC007326.9	Island	C4
cg09619624	chr22	18998119	18998120	AC007326.9	Island	C4
cg18098433	chr11	115192204	115192205	CADM1	.	C4
cg21913319	chr1	86156945	86156946	COL24A1	Island	C5

cg26121931	chr6	29927427	29927428	HCG4B	S_Shore	C5
cg10334121	chr3	138010153	138010154	CLDN18	Island	C1
cg10510478	chr3	138009968	138009969	CLDN18	N_Shore	C1
cg23008404	chr3	138010155	138010156	CLDN18	Island	C1
cg20950465	chr1	52602509	52602510	GPX7	Island	C5
cg13939204	chr9	127904778	127904779	ST6GALNAC6	.	C1
cg22773661	chr4	48490208	48490209	ZAR1	Island	C5
cg05855588	chr13	33016135	33016136	KL	Island	C5
cg21545902	chr13	33015945	33015946	KL	Island	C5
cg03811891	chr6	158516254	158516255	CACYBPP3	Island	C4
cg06214007	chr1	89363749	89363750	GBP6	.	C4
cg26866325	chr4	74054842	74054843	PPBPP2	.	C4
cg07748159	chr11	83286867	83286868	RP11-727A23.10	S_Shore	C4
cg04525496	chr5	83471707	83471708	VCAN	N_Shore	C5
cg19540689	chr5	83471478	83471479	VCAN	N_Shore	C5
cg21810188	chr5	83471631	83471632	VCAN	N_Shore	C5
cg13883696	chr1	61054216	61054217	NFIA	Island	C5
