# Supplementary material for: Mining the Prognostic Role of DNA Methylation Heterogeneity in Lung Adenocarcinoma
Source: Dis Markers. 2022 May 28;2022:9389372. doi: 10.1155/2022/9389372 (PMC9168807; doi:10.1155/2022/9389372)
Supplement: Supplementary 9 — Supplementary Table 3: functional analysis of methylation-regulated genes. [file 9389372.f9.pdf]

Table S3

GO.ID	Description	p.Val	FDR	Phenotype
GO:0032501	multicellular organismal process	3.07E-02	3.07E-02	1
GO:0048856	anatomical structure development	2.65E-02	2.65E-02	1
GO:0009653	anatomical structure morphogenesis	3.48E-04	3.48E-04	1
GO:0007275	multicellular organism development	1.40E-03	1.40E-03	1
GO:0007389	pattern specification process	6.60E-04	6.60E-04	1
GO:0009790	embryo development	2.01E-04	2.01E-04	1
GO:0009792	embryo development ending in birth or egg hatching	4.91E-02	4.91E-02	1
GO:0043009	chordate embryonic development	4.14E-02	4.14E-02	1
GO:0048598	embryonic morphogenesis	6.19E-04	6.19E-04	1
GO:0048731	system development	1.47E-03	1.47E-03	1
GO:0055123	digestive system development	8.31E-03	8.31E-03	1
GO:0001501	skeletal system development	1.04E-02	1.04E-02	1
GO:0048706	embryonic skeletal system development	1.81E-03	1.81E-03	1
GO:0061458	reproductive system development	1.73E-02	1.73E-02	1
GO:0035295	tube development	3.52E-04	3.52E-04	1
GO:0035239	tube morphogenesis	4.80E-03	4.80E-03	1
GO:0048565	digestive tract development	3.28E-03	3.28E-03	1
GO:0048513	animal organ development	1.49E-03	1.49E-03	1
GO:0048568	embryonic organ development	9.60E-04	9.60E-04	1
GO:0048608	reproductive structure development	1.50E-02	1.50E-02	1
GO:0051239	regulation of multicellular organismal process	5.35E-03	5.35E-03	1
GO:0050793	regulation of developmental process	3.53E-02	3.53E-02	1
GO:0030154	cell differentiation	3.18E-02	3.18E-02	1
GO:0045165	cell fate commitment	4.13E-02	4.13E-02	1
GO:0045596	negative regulation of cell differentiation	2.22E-02	2.22E-02	1
GO:0048518	positive regulation of biological process	1.07E-03	1.07E-03	1
GO:0048522	positive regulation of cellular process	2.56E-02	2.56E-02	1
GO:0009952	anterior/posterior pattern specification	1.26E-02	1.26E-02	1

GO:0043565	sequence-specific DNA binding	4.30E-03	4.30E-03	1
GO:0000981	RNA polymerase II transcription factor activity, sequence-specific DNA binding	2.85E-02	2.85E-02	1
KEGG:04514	Cell adhesion molecules (CAMs)	1.21E-03	1.21E-03	1
TF:M00649	Factor: MAZ; motif: GGGGAGGG	2.93E-02	2.93E-02	1
TF:M01587	Factor: FPM315; motif: SRGGGAGGAGGN	2.44E-02	2.44E-02	1
TF:M07040_1	Factor: GKLF; motif: NNRRGRRNGNSNNN; match class: 1	3.96E-04	3.96E-04	1
